# Assessing functional annotation transfers with inter-species conserved coexpression: application to *Plasmodium falciparum*

**DOI:** 10.1186/1471-2164-11-35

**Published:** 2010-01-15

**Authors:** Laurent Bréhélin, Isabelle Florent, Olivier Gascuel, Éric Maréchal

**Affiliations:** 1 Méthodes et algorithmes pour la Bioinformatique, LIRMM, Univ. Montpellier 2, CNRS; 161 rue Ada, 34392 MONTPELLIER, France; 2 FRE3206 CNRS/MNHN, USM504, Biologie Fonctionnelle des Protozoaires, RDDM, Muséum National d'Histoire Naturelle, Paris, France; 3 UMR 5168 CNRS-CEA-INRA-Grenoble Université, Institut de Recherches en Technologies et Sciences pour le Vivant, CEA-Grenoble; 17, rue des Martyrs, 38054 Grenoble, France

## Abstract

**Background:**

*Plasmodium falciparum *is the main causative agent of malaria. Of the 5 484 predicted genes of *P. falciparum*, about 57% do not have sufficient sequence similarity to characterized genes in other species to warrant functional assignments. Non-homology methods are thus needed to obtain functional clues for these uncharacterized genes. Gene expression data have been widely used in the recent years to help functional annotation in an intra-species way via the so-called *Guilt By Association *(GBA) principle.

**Results:**

We propose a new method that uses gene expression data to assess inter-species annotation transfers. Our approach starts from a set of likely orthologs between a reference species (here *S. cerevisiae *and *D. melanogaster*) and a query species (*P. falciparum*). It aims at identifying clusters of coexpressed genes in the query species whose coexpression has been conserved in the reference species. These conserved clusters of coexpressed genes are then used to assess annotation transfers between genes with low sequence similarity, enabling reliable transfers of annotations from the reference to the query species. The approach was used with transcriptomic data sets of *P. falciparum*, *S. cerevisiae *and *D. melanogaster*, and enabled us to propose with high confidence new/refined annotations for several dozens hypothetical/putative *P. falciparum *genes. Notably, we revised the annotation of genes involved in ribosomal proteins and ribosome biogenesis and assembly, thus highlighting several potential drug targets.

**Conclusions:**

Our approach uses both sequence similarity and gene expression data to help inter-species gene annotation transfers. Experiments show that this strategy improves the accuracy achieved when using solely sequence similarity and outperforms the accuracy of the GBA approach. In addition, our experiments with *P. falciparum *show that it can infer a function for numerous hypothetical genes.

## Background

Malaria is one of the deadliest infectious diseases, threatening half a billion humans worldwide with a yearly death toll of 1 to 2 million people, mainly in developing countries (World Malaria Report 2005, Geneva, World health Organization, WHO/UNICEF; 2005). Malaria is due to infections by protozoan parasites of the *Plasmodium *genus, transmitted by bites of female Anopheles mosquitoes. Of the four species that infect humans, *P. falciparum *causes the greatest incidence of illness and death [1]. Despite sustained efforts to combat the disease, safe and affordable new drugs, and new drug targets, are still required to circumvent drug resistance outbreaks triggered by the use of existing drugs, as anti-malarial vaccines are not yet available [2,3]. The *P. falciparum *genome was published in 2002, initially revealing 5 268 protein coding genes, of which about 60% did not have sufficient similarity to characterized genes in other species to justify provision of functional assignments [4]. At the date of writing, the PlasmoDB 5.4 database includes 5 484 coding genes, 3 155 of which (57%) are still annotated as hypothetical. Thus, more than half of *P. falciparum *genes lack a functional annotation, which is a high proportion compared to that usually observed in other eukaryotic genomes [4]. Although this situation may be explained by the existence of genes that are unique to the *P. falciparum *species, the *Plasmodium *genus or even the Apicomplexan phylum to which these organisms belong [5], it is certainly further exacerbated by the high evolutionary distance between *P. falciparum *and other sequenced organisms [6], which makes homology detection particularly difficult. The extreme AT bias (above 80%), the high amino-acid bias (six amino acids account for more than 50% of the protein composition), and the presence of a large number of low-complexity regions that are believed to form unstructured segments [7], can impede the efficacy of standard methods of sequence comparison based on BLAST [8,9] or HMMER [10]. Thus, *P. falciparum *is a typical organism for which new approaches are needed to help detection of distant homologs.

Usually, a low (stringent) e-value threshold is used to determine whether a measured BLAST sequence similarity allows functional annotation transfer. However, even if they have high sequence similarity, homologous proteins are not necessarily functionally equivalent [11]. Moreover, in the case of highly divergent proteins like those of *P. falciparum*, this strategy can fail to annotate many proteins. In this latter case, increasing the e-value threshold might enable annotation of a larger proportion of proteins, but with the risk of more spurious annotations. Here, we propose to use the conservation of coexpression between species to decide whether to transfer, or not, the functional annotations from homologs with potentially weak sequence similarity.

Gene expression data have been widely used in recent years to help gene annotation *via *the so-called *Guilt by Association *(GBA) principle. Contrary to sequence homology which involves inter-species annotation transfers—*i.e*. genes characterized in other species are used to annotate genes of the newly sequenced genome—GBA approaches involve intra-species annotation transfers: annotations of an already characterized gene in the organism are transferred to an uncharacterized gene if the two genes share similar transcriptomic profiles [12,13]. This approach has been successfully applied to *P. falciparum *[14-16] and provides functional clues for many uncharacterized genes. However, depending on the data sets and on the type of function, the accuracy of the GBA predictions can be low [16].

Several studies report the conservation of coexpression between species [17-19]. In particular, these analyses show that if two genes are coexpressed in one organism, then their orthologs also tend to be coexpressed in other organisms, *i.e*. coexpression is sometimes conserved. In this paper, we propose a method that uses this property to help for annotation transfer in an *inter-species *way. The principle is as follows: we have (1) a query species (*P. falciparum*) and a reference species (for example *S. cerevisiae*), (2) two microarray data sets that monitor the level of expression of the genes of the two species, and (3) a set of likely orthologs between the two species. The *coexpression context *of a gene in one of the species is defined as the set of genes that appear to be coexpressed with this gene in the microarray data of the species. Then, if two homologs with potentially weak sequence similarity have similar coexpression context—*i.e*., if their coexpression contexts share a sufficiently high number of orthologs—, they likely have the same function.

We show that considering conserved coexpression (or *co-coexpression *for short) between species improves the accuracy of functional annotation transfers by sequence similarity alone, and outperforms the accuracy of the classical GBA approaches. Moreover, it also allows reliable annotation transfers when the homology is doubtful, a useful property for the highly divergent proteins of *P. falciparum*. The method was applied on two different reference species (*S. cerevisiae *and *D. melanogaster*), and with different gene expression data screening different biological conditions for each species. These studies enabled us to provide a function for 74 previously hypothetical genes and to confidently propose refined annotations of previously incomplete or wrong original functions for 58 additional genes. Notably, we revised the annotation of genes involved in ribosomal proteins and ribosome biogenesis and assembly, thus highlighting several potential drug targets.

## Results

### Gene expression data

For the *P. falciparum *query species, we used two series of transcriptomic profiles produced during the parasite life cycle. The first series, referred to as the Bozdech data set (BO), measures the expression level of *P. falciparum *genes once an hour during one complete 48-hours erythrocytic cycle [20]. The second data set, referred to as the Le Roch data set (LR), measures the expression level of *P. falciparum *genes in 9 different developmental stages during the entire life cycle both in its human host and in the insect vector, in two experimental conditions (a total of 16 expression measurements) [14]. For the reference species, we selected two *S. cerevisiae *and one *D. melanogaster *transcriptomic studies. The first *S. cerevisiae *data set, referred to as the Gasch data set (GA), is a compendium of 176 transcriptomic measurements in a variety of stress conditions [21] and the second one, the Spellman data set (SP) measures gene expression levels during a little more than two yeast cell cycles (77 expression measurements) [22]. Finally, the *D. melanogaster *data set (PI) corresponds to a transcriptomic study of 15 time points in the early development of the fly [23]. One advantage of our approach is that it can deal with transcriptomic data monitoring different conditions in the two organisms. Even if better results can be expected from data produced by identical assays, in practice this is often impossible. Rather, the aim here is to find gene clusters likely involved in analogous pathways.

### The approach

Our approach involves the detection of clusters of coexpressed genes in the query species whose coexpression is conserved in the reference species. We implemented this approach with a probabilistic model specially designed for modeling the cluster of coexpressed genes in each species as well as the conservation of coexpression between the two species.

#### The model

**Figure 1:**
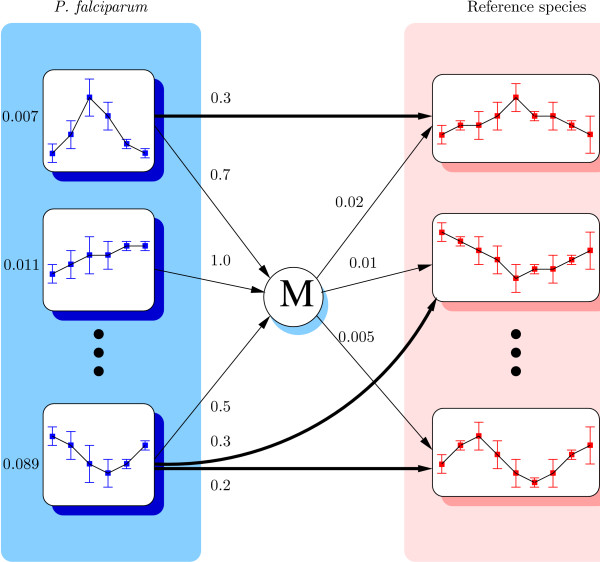
**The probabilistic model**. For each species, only three prototypes are represented here, while several dozens are usually used. Prototypes are modeled with multivariate Gaussian models represented here by series of means and standard deviations. *P. falciparum *prototypes are labeled with their prior probabilities. Prior probabilities of the prototype of the reference species are on the outgoing transitions from the mute state *M*. Direct transitions corresponding to evolutionary conservation between prototypes are in bold.

In each species, coexpression is modeled with *prototypes *of gene expression profiles. This is an approach similar to that used in classical clustering algorithms such as SOM [24] or K-MEANS [25,26]. The probabilistic model has the following structure (see Figure 1). We use *K *prototypes of gene expression profiles for *P. falciparum*, and *K' *prototypes for the reference species. In the experiments below, we use 100 prototypes for each species. Each prototype (of *P. falciparum *and the reference species) is modeled with a probabilistic, multivariate Gaussian distribution defining a probability function over the gene expression profiles of the species. As all prototypes are different, the profile of a given gene has different probabilities depending on the prototype concerned, and hence some prototypes are more likely than others for the gene. Genes are associated with their most likely prototype, and all genes associated with the same prototype are considered to be coexpressed. There is a prior-probability distribution on the prototypes of each species, which reflects the proportion of genes that are associated with each prototype in the species.

When the coexpression of a set of genes associated with prototype *k *of *P. falciparum *is conserved, the homologs of these genes in the reference species also tend to share a common prototype *k' *in this species. This is modeled by a transition between *k *and *k'*, labeled with a probability *P*(*k' *|*k*) representing the proportion of genes associated with *k *whose homologs are associated with *k'*. Finally, an additional mute state *M *is used for modeling all non-conserved coexpressions: it is possible to reach the *M *state from every prototype of *P. falciparum*, and reach every prototype of the reference species from the *M *state. Prototypes of *P. falciparum *are labeled with their prior probability *P*(*k*), *k *= 1 ... *K*. The prior probabilities of the prototypes of the reference *P*(*k' *), *k' *= 1 ... *K' *are on the outgoing transitions from the *M *state.

Usually, one prototype is linked with zero or with one prototype of the other species. However, as illustrated by the last *P. falciparum *prototype in Figure 1, one prototype can sometimes be linked with two or more prototypes. This may occur for several reasons. For biological reasons first: since some genes are part of several pathways, the same pathway is not necessarily active in the two species—either due to the species' specificities, or because the microarray data monitor different conditions in the two species. For experimental reasons next: the experimental noise in microarray data can artificially break down a set of coregulated genes into two (or more) different clusters. Conversely, two clusters with a specific but close signature of expression profiles may appear to be similar and associated with the same prototype.

This model defines a probabilistic model of pairs of gene expression profiles. From a computational standpoint, it is a Hidden Markov Model (HMM) [27,28], therefore we benefit from all classical algorithms designed for using and training these models (see reference [27] for a description of these algorithms). Given the expression profiles of a pair of query-reference genes, this HMM can be used to compute the probability density of the profiles under two different hypotheses. Under the hypothesis of dependence of the expression profiles—that is, homologous genes belonging to a group of conserved coexpression— this is the probability of generating the two profiles by a path in the HMM that uses only direct transitions between prototypes. Under the independence assumption of the expression profiles, this is the probability of generating the two profiles by a path that uses the *M *state: this way, the density only depends on the prototype's prior probabilities and not on conditional probabilities between prototypes.

#### Learning the model

Learning the model involves 1) building the structure (*i.e*. deciding on the direct transitions between prototypes); 2) training the model to estimate the numerical parameters of the HMM (*i.e*. Gaussian distributions, transition probabilities, and prototype prior probabilities). Building the structure involves identifying the conserved coexpressed clusters between the two species. For this purpose, we designed an algorithm which takes as input a set of gene pairs with high sequence similarity selected with the Reciprocal Best Hit procedure (RBH) between the two species (see Methods). An initial gene clustering of each species in *K *and *K' *clusters is computed with the K-MEANS algorithm [25]. Next, a statistical procedure computes, for each cluster pair (*k*, *k'*), the number of gene pairs with high sequence similarity between *k *and *k'*, and tests if this can be expected by chance. When this is not the case, a transition between *k *- *k' *is added to the model. By the end of the process, the structure of the HMM has been built, and the classical *Baum-Welch algorithm *[29] is then run to train the model.

#### Assessing functional conservation

Once this model is built, it can be used for assessing the functional conservation between two putative homologs. Given the gene expression profiles of the two genes, the procedure involves searching for the prototypes *k *and *k' *that have the highest probability of generating the two profiles. This can be done with the classical Viterbi algorithm of the HMMs [27]. If these prototypes share a direct transition, then *P*(*k' *|*k*) reflects the conservation of coexpression between *k *and *k'*. *P*(*k*)·*P*(*k' *|*k*) is the prior probability of these prototypes under the hypothesis of dependence of the two profiles. In a similar way, *P*(*k*)·*P*(*k' *) is the prior probability of these prototypes for genes that are conditionally independent. The greater the difference between these probabilities, the more likely the functional conservation between genes. Thus, if the ratio of these probabilities, *i.e. P*(*k' *|*k*)/*P*(*k' *) is higher than a given threshold ξ, the annotations of the reference gene are transferred to the query gene. Otherwise, the annotations are not transferred.

### Estimate of accuracy with the Gene Ontology

We first investigated the global accuracy of our approach using genes already annotated in the Gene Ontology (GO, http://www.geneontology.org), which is a systematic and standardized nomenclature to annotate genes in terms of their associated biological processes (BP), cellular components (CC) and molecular functions (MF), in a species-independent manner. The validation procedure is as follows. First, an HMM is learned from a set of gene pairs selected by RBH. Next, given several (putative) homologous genes, the HMM is used to select from among these pairs those authorizing annotation transfers from the reference to the query species. Transfers involving gene pairs already annotated in both species are used to compute an estimate of the accuracy of the method. Namely, the proportion of annotations of the reference gene shared by the query gene is computed, and the global accuracy is then estimated by averaging all results (see Methods).

Most available *P. falciparum *annotations have an IEA code only, indicating that they have not been reviewed by a curator. In the BP ontology for example, of the 1799 annotated genes (35%), 1067 (68%) have an IEA code. In these conditions, it is recommended to first check the method on curated annotations of better annotated organisms. To this end, we estimated the method accuracy using *D. melanogaster *as the query species and *S. cerevisiae *as the reference species. The procedure was applied to the Pilot (PI) and Spellman (SP) data sets. We first investigated the method accuracy on genes with high sequence similarity, by applying it to the RBH pairs used to learn the HMM.

**Figure 2:**
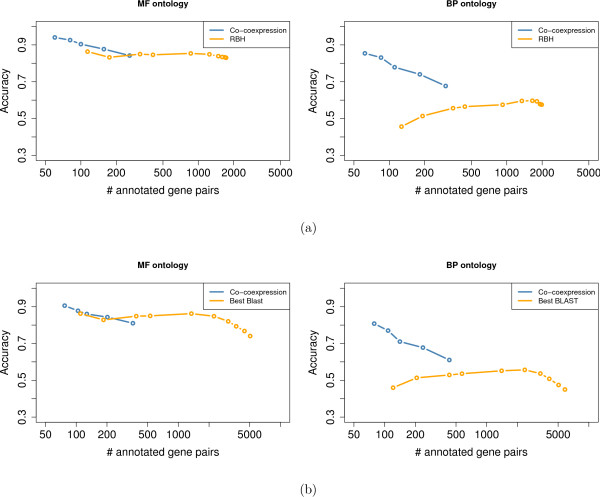
**Estimate of the method accuracy with GO: *D. melanogaster *vs. *S. cerevisiae***. Accuracy achieved on the MF (left) and BP (right) ontologies when using sequence information alone (orange curves, for different e-value cutoffs) and when exploiting the expression context (blue curves, for different ξ thresholds). The x-axis shows the number of gene pairs authorizing annotation transfers. (a) Results achieved on RBH gene pairs. (b) Results achieved on gene pairs with potentially low sequence similarity.

Figure 2(a) summarizes the accuracy achieved on the BP and MF ontologies, when varying the ξ threshold to decide on the annotation transfer. In addition, the accuracy of the RBH method alone was also computed. For this method, a BLAST e-value cutoff was introduced to control the accuracy and number of functional transfers—*i.e*. all RBH pairs above the cutoff are not considered.

Accuracies achieved on the BP and MF ontologies strongly differ. For the MF ontology, sequence information alone (RBH) is accurate (around 80%) and no improvement is observed using coexpression context. However, for the BP ontology, the RBH accuracy is below 60%, and using coexpression context clearly improves the results. As expected, the number of gene pairs authorizing a functional transfer (*x*-axis) increases with the ξ threshold, while the accuracy of the method decreases. Surprisingly, the e-value cutoff introduced in the RBH approach fails to control the accuracy, which tends to prove that the RBH strategy already captures most of the sequence information relevant for the annotation transfer.

We next investigated the potential of the approach to assess functional conservation between gene pairs with less stringent sequence similarity. To this end, all gene pairs with BLAST sequence similarity below a loose e-value threshold (0.1) were considered. This list contains all pairs selected by the RBH strategy, but also many others. Figure 2(b) summarizes the accuracy achieved for the MF and BP ontologies. For the purpose of comparison, we also estimated the accuracy of the approach that associates with each *D. melanogaster *gene its best BLAST hit in *S. cerevisiae*, using different e-value cutoff to control the number and accuracy of functional transfers. Here again we observe for the BP ontology that using expression context greatly improves the accuracy achieved using only sequence similarity.

**Figure 3:**
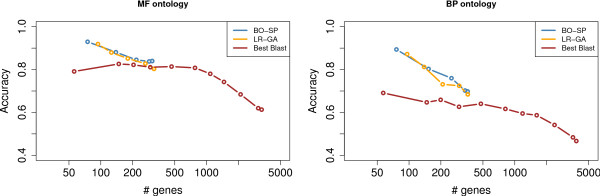
**Estimate of the method accuracy with GO: *P. falciparum *vs. *S. cerevisiae***. Accuracies achieved on the Le Roch-Gasch (orange curve) and Bozdech-Spellman (blue curve) comparisons, estimated on the MF (left) and BP (right) ontologies with different ξ thresholds—from left to right on each curve: 40, 20, 10, 5, 2, 1. The x-axis shows the number of gene pairs authorizing annotation transfers. The brown curves show the accuracy achieve when associating each gene of *P. falciparum *with its best BLAST hit in *S. cerevisiae*, for different e-value thresholds.

Next, the same experiments were conducted on *P. falciparum *using *S. cerevisiae *as reference species (transcriptomic studies Le Roch vs. Gash and Bozdech vs. Spellman) and with all available GO annotations (IEA included). In each experiment, the HMM was learned using the *P. falciparum*-yeast orthologs selected with the RBH procedure, and was then used to assess the functional annotation transfers of all gene pairs with BLAST sequence similarity below 0.1. Results were compared with the accuracy of the method that associates with each gene *P. falciparum *of its best BLAST hit in *S. cerevisiae*, using different e-value cutoffs (see Figure 3). Like for *D. melanogaster*, taking coexpression context into account outperforms the accuracy achieved when using only sequence information. Table 1 is an estimate of the method potential on genes that have no, or only non-curated GO annotations. This table shows that, depending on the threshold chosen, several dozen genes with no BP annotation can be associated with a *S. cerevisiae *gene annotated in this ontology. Moreover, even more genes that only possess non-curated annotations (mainly derived from sequence similarity) can be associated with a *S. cerevisiae *gene with curated annotations. This reveals the potential of our method for improving annotations based on sequence similarity only, by using information derived from gene expression.

**Table 1:** Number of uncharacterized or poorly characterized genes of P.falciparum that can be annotated by the LR-GA or BO-SP comparisons with different ratio thresholds.

ξ ratio threshold		40	20	10	5	2
LR-GA	# unchar. genes	4	47	80	142	183
	# IEA char. genes	74	90	127	163	214

BO-SP	# unchar. genes	1	31	92	129	167
	# IEA char. genes	77	106	115	176	230

Lines "# unchar. genes" show the number of genes without any annotation in the BP ontology that can be associated with an *S. cerevisiae *gene annotated in this ontology. Lines "# IEA char. genes" show the number of genes with only electronic non-curated annotations (GO Evidence Code IEA) in the BP ontology that can be associated with an *S. cerevisiae *gene with a curated annotation in this ontology.

### Appraisal of the proposed annotation transfers

We next investigated the potential of co-coexpression analysis to improve simple homology-based annotation transfers in a more careful and systematic way. The method was applied to the *P. falciparum *vs. *S. cerevisiae *and *P. falciparum *vs. *D. melanogaster *studies LR-GA, LR-SP, BO-GA, BO-SP, and BO-PI. For each analysis, a model was learned using gene pairs selected by RBH, and was then used to assess functional transfers for gene pairs with BLAST sequence similarity below 0.1. For each experiment, our method outputs the gene pairs authorizing a functional transfer in an organized way, by presenting in cluster pairs the coexpressed genes whose coexpression has been conserved. Outputs of all above analyses along with useful links to appropriate databases and BLAST alignments can be browsed in the Supplementary Data files (see Additional Files 1, 2, 3, 4 and 5).

All pairs were analyzed in all clusters to validate (or invalidate) the identified pairs and, in addition, we checked that *P. falciparum *genes were associated with the same yeast genes independently of the data sets considered (for example in LR-GA versus BO-GA or BO-GA versus BO-SP). In cases where *P. falciparum *genes were paired both with yeast and *Drosophila *genes, we checked that the two latter genes had concordant annotations, further supporting the inter-species annotation transfer. When both genes of the output pairs had a previously inferred function, we observed nearly no annotation conflict. All discrepancies could be attributed to an obvious error, or incomplete analysis of the *Plasmodium *gene. We did not detect any *Plasmodium *gene with known function, paired with hypothetical *Saccharomyces *or *Drosophila *genes. However, on several occasions, hypothetical *Plasmodium *genes were paired with hypothetical genes in the reference species—mostly *Drosophila*, which is not yet as extensively annotated as yeast.

Several hypothetical *P. falciparum *genes were paired with annotated *S. cerevisiae*/*D. melanogaster *genes. In some of these pairs, the *P. falciparum *gene is only partially annotated, as for example in the LR-GA co-coexpression analysis, the gene pairs (*PFE1240w*; *YPL207W*) and (*PF11_0090*; *YGR103W*) in the first cluster pair (cluster pair #0), or the gene pair (*PF14_0661*; *YOR145C*) in cluster pair #1 (see Additional File 1). In other pairs, the *P. falciparum *gene has absolutely no GO data. See, for example, the pairs (*PF11_0471*; *YCR072C*), (*PF07_0121*; *YHR170W*), or (*PF10_0200*; *YNL132W*) in the cluster pair #0 of the LR-GA analysis. The comparative analysis therefore allows correction of incomplete annotations and proposes annotations for completely unannotated *P. falciparum *genes. Several pairs associating hypothetical *P. falciparum *genes have surprisingly good or even excellent BLAST matches, with e-values lower than 1.e-40. For the others, with moderate or poor BLAST matches, careful examination was crucial to assess the proposed annotation transfer. This led to the identification of several homologs with probably the same function, generally with amino acid compositional biases and the occurrence of low complexity inserts in the *Plasmodium *genes. See for example the pairs (*PF10_0278*; *YKR081C*), e-value = 2e-05, and (*PF14_0072*; *YNR046W*), e-value = 2e-09, in the cluster pair #0 of the LR-GA co-coexpression analysis. For some other pairs, however, the sequence alignments are clearly irrelevant, aligning repeated domains, low complexity segments, or very short proportions of one gene—*e.g*. in the LR-GA analysis, (*PFI0635c*; *YML093W*) and (*PF13_0256*; *YCL037C*) in the cluster pair #0. Thus, the co-coexpression analyses are very interesting indicators to identify hypothetical *Plasmodium *genes that have sequence and expression similarities with a yeast and/or *Drosophila *functional gene, but they cannot entirely replace an expert examination of the sequences to support or reject the possible annotation transfers they propose.

Three tables summarize the most interesting annotation transfers proposed by the co-coexpression analysis:

• Additional File 6 shows the results obtained when focusing on the cytosolic and organellar (mitochondrial and apicoplast) ribosomal proteins (GO:0003735) based on comparative analysis between *P. falciparum *and *S. cerevisiae *expression profiles (LR-GA; BO-GA and BO-SP) and between *P. falciparum *and *D. melanogaster *expression profiles (BO-PI). For genes encoding ribosomal proteins of the cytosol, the co-coexpression analyses enable the recovery of 57 functional genes previously available in the PlasmoDB v5.4 resource, the refinement of 8 incomplete or wrong annotations, an inference of a function for 2 hypothetical genes, and provide no information on 9 already known genes. For genes encoding mitochondrial ribosomal proteins, 6 could be annotated on the basis of the co-coexpression analysis (4 confirmed PlasmoDB v5.4 annotation, 1 refinement of an incomplete annotation and 1 functional inference of hypothetical genes). None of the 30 apicoplast ribosomal proteins could be annotated according to the co-coexpression analysis, which is consistent with the lack of plastid in yeast and *Drosophila*, and consequently provides a negative control for this procedure.

• Additional File 7 shows the pairing of *P. falciparum *genes with *S. cerevisiae *or *D. melanogaster *genes functionally annotated as factors of the ribosome assembly and biogenesis (GO:0042254) in the nucleolus and/or nucleus or cytoplasm, factors involved in the rRNA metabolic process (GO:0016072) and tRNA processing (GO:0008033). In these 102 pairs, co-coexpression analysis confirmed the functional inference of 7 *Plasmodium *genes, a proposed refinement or correction of 46 genes and inference of a function for 49 hypothetical genes. In this analysis, careful examination was critical for reviewing gene pairs when BLAST matches were questionable—see, for example, (*PFL2295w*; *YKL099C*), e-value = 0.095 found in BO-GA and BO-SP and predicting a nucleolar rRNA processing protein associated with U3 snoRNA. A few unexpected situations were discovered, such as *PFA0330w *known as the *PfAARP2 *protein [30] that turned out to correspond to a nucleolar small ribosomal subunit assembling protein similar to *YPL217C*, with an e-value = 1.4e-45. Likewise, the pairing of two contiguous *P. falciparum *genes, *PF14_0436 *and *PF14_0437*, with distinct parts of the same yeast gene *YNL112W *involved in ribosomal biogenesis and assembly, allowed us to propose a revised gene structure at the corresponding *P. falciparum *locus (Additional File 8).

• Additional File 9 is a series of other remarkable pairings between *P. falciparum *and *S. cerevisiae *or *P. falciparum *and *D. melanogaster *that were discovered. Apart from the pair (*PFL1345c*; *YPL095C*) that allowed us to propose a refined annotation as a "histone S-adenosyl methyltransferase, putative" for *PFL1345c*, and the pair (*PF14_0178*; *YGR048W*), that led to a new annotation as a "polyubiquitinated protein - 26S proteasome guiding protein, putative" for *PF14_0178*, most of these pairs were only found in only one co-coexpression analysis. For example, see the *P. falciparum *gene *PFC0100c*, now proposed to be involved in "Golgi organisation and biogenesis" (pair (*PFC0100c*; *FBgn0030365*) in BO-PI analysis).

On the whole, the biological functions attributed to the gene of the reference species in the output gene pairs show that a limited number of cellular processes are represented. In particular, genes coding for ribosome constituents are extremely abundant, for example in cluster pairs #13, #14 and #18 of the LR-GA analysis, or in cluster pairs #14 and #24 of the BO-GA analysis. Proteins involved in ribosomal biogenesis and assembly and rRNA metabolism and processing, most of which are annotated as nucleolar proteins in Yeast and/or Drosophila, are also frequently returned in co-coexpressed clusters (such as in cluster pair #2 of the BO-GA analysis). To a lesser extent, genes encoding proteins playing roles in histone modifications, cytoskeleton dynamics, Golgi organization and biogenesis, proteasome, and cell cycle regulation (see Discussion below) are also discovered.

## Discussion

### Ribosomal proteins and potential drug targets

Many gene pairs highlighted in the present study comprise genes coding for ribosomal proteins or involved in ribosome biogenesis and assembly. Whereas the structure of malarial ribosomal RNAs has attracted considerable attention and has been analysed in many comparative studies, no census of ribosomal proteins was undertaken in the first large scale expert annotation of *P. falciparum *[4,31] and annotations have not been seriously revised since. The results of the present study, combined with other published data [32] and an analysis by Akhil Vaidya group (Philadelphia, USA), were therefore used to propose a revised annotation for the future versions of the GeneDB and PlasmoDB public databases. The annotation presented here was transferred to the PlasmoDB database before the submission of this article and was used in a recent study by Mishra et al. (2009) [33].

This re-annotation potentially involves promising drug targets. Many of the drugs used in clinical medicine to treat infectious diseases interfere with protein synthesis by targeting the pathogen ribosomes, *e.g*. macrolides, ketolides, lincosamides, oxazolidinones, aminoglycosides and tetracyclines [34]. Many teams have studied *Plasmodium *ribosomal RNAs as targets for antimalarial drugs, including thiostrepton, known to bind the apicoplast large ribosomal subunit rRNA [35,36], clindamycin, shown to also act on the large ribosomal subunit rRNA in *Toxoplasma *[37] and tetracycline, whose antimalarial effect is likely due to its binding to the mitochondrial and/or apicoplast small subunit rRNA [38]. Whereas recent studies focused on the ribonucleic constituents of ribosomes, *i.e*. rRNAs, as a promising target for innovative drugs [39], ribosomal proteins are also candidates of choice in the search for antimalarial drugs. Sidhu et al. (2007) [40] reported, for instance, that azithromycin, a broad spectrum antibiotic macrolide, could bind the apicoplast ribosomal protein *L4 *(*PFCOMPIRB-rpl4*; see Add. File 6), a polypeptide suspected to interact with the apicoplast ribosomal protein *L22 *(*PF14_0642*, annotated from our analysis as an organelle ribosomal protein *L22/L17 *precursor, putative; see Add. File 6) and the apicoplast large RNA subunit. In general, the main problem in contemporary drug development is toxicity [34]. Concerning the uses of ribosomes as drug targets, the characterization of structural domains that diverge between a pathogen and its host, and the identification of residues whose polymorphism might be related to drug resistance, are crucial in the search for new drug candidates. The present study, which allows prediction of ribosomal proteins with low or very low sequence conservation, points to regions of the ribosomes that likely bind specific small molecules that would not interfere with the host ribosomes.

### Proteins involved in cell cycle regulation

Molecular mechanisms and proteins involved in the control of *P. falciparum *cell cycle are largely unknown [41]. We focused on this biological process as a case study, and attempted to uncover some related malarial genes, by paying special attention to the Bozdech data sets in which the cell cycle was synchronized. 38 genes were proposed to be involved in cell cycle regulation by Tienda-Luna et al. (2008) [42], and 97 *P. falciparum *kinase genes were reported by Ward et al. (2004) [43] as representative of the malarial kinome. From these genes, 24 and 30 have expression measurements in the Bozdech data, respectively, while 9 and 4 genes are actually retrieved by co-coexpression analysis. These genes code for three putative DNA replication licensing factors (*PF07_0023*, *PF13_0291 *and *PFL0580w*), two putative minichromosome maintenance proteins (*PFE1345c *and *PFL0560c*), the proliferating cell nuclear antigen (*PF13_0328*), the putative karyopherin alpha (*PF08_0087*) and beta (*PFE1195w*) homologs, the Myb2 protein (*PF10_0327*), the NIMA-related protein kinase (*PFL1370w*), the ROI kinase like protein (*PFD0975w*), casein kinase I (*PF11_0377*) and casein kinase II regulatory subunit (*PF13_0232*). Interestingly, most of these genes were found in the Bozdech versus Spellman comparison (see cluster pairs #24, #27 and #28 in Additional File 4). A few other *P. falciparum *genes are paired with *S. cerevisiae *genes with annotations related to cell cycle control (see Add. File 9). For example, analysis of cluster pair #24 of the BO-SP output allowed us to refine the annotations of a ROI kinase like protein (*PFD0975w*); analysis of cluster pair #28 allowed refinement of the annotation of a putative serine/threonine kinase similar to RAD53 (*PF11_0488*); in cluster pair #20, several hypothetical *P. falciparum *genes could be pinpointed as putatively involved in a cell cycle related process. In cluster pair #11 of the LR-GA output, which contains the casein kinase I gene (*PF11_0377*) [43], a functional annotation of a chromosome condensation protein involved in sister chromatid exchange is proposed for the hypothetical gene *MAL13P1.21*, paired with a significant e-value = 4e-07 to *YBL097W*.

By contrast with the genes involved in ribosomal biogenesis and assembly, very few *P. falciparum *genes involved in cell cycle regulation can be eventually identified. This is possibly due to the lack of some of the marker genes in the data sets compared, to the highly atypical way in which the parasite divides, *i.e*. schizogony [44], and to the evolutionary distance between *P. falciparum *and the model organisms in which the cell cycle has been studied in depth, *i.e*. animals and fungi [45].

### High and low representation of certain functions in co-coexpression analysis

The high representation of biological functions such as ribosome biogenesis and assembly, as well as the apparent lack of other biological functions such as cell cycle regulation might be a bias of the present method for different biological reasons. First, we expected to find the conservation of housekeeping genes throughout eukaryote biodiversity, including genes involved in protein biosynthetic processes. Secondly, the biological specificities of the published studies we selected as data sets for our analyses might also explain this fact. Intuitively, outputs of co-coexpression analysis and therefore usefulness in terms of gain to transfer annotations are likely highly dependent on the type of transcriptomic data sets compared. In particular, co-coexpression based annotations are expected to depend both on (1) the organisms that are compared (*i.e*. their phylogenetic distance), and (2) on the biological conditions of the assays used to produce the transcriptomic profiles.

Concerning the choice of organisms, we obtained better results in terms of the capacity to transfer annotations by comparing *P. falciparum *to *S. cerevisiae *than by comparing *P. falciparum *to *D. melanogaster*. The reasons for the better performance of the *P. falciparum *vs. *S. cerevisiae *comparisons may be due to the better annotation status of the *S. cerevisiae *genome compared to that of *D. melanogaster *and/or the relative genetic distance between the species we compared. Undoubtedly, improvements in *D. melanogaster *genome annotations will improve our ability to transfer annotations from the fly to *P. falciparum*, which were sometimes limiting in the present study.

Concerning the biological conditions, the transcriptomic data we selected in published studies (for review, [46]) for our analyses focused on proliferating and differentiating stages of *Plasmodium*, mobilizing the transcriptional/translational machinery and possibly adding weight to this particular cellular process in the final output. This is further supported by the presence in the results of several clusters involved in subcellular targeting of proteins soon after their biosynthesis. Moreover, querying *P. falciparum *genes involved in cell cycle regulation using the Spellman data set [22] that directly deals with this question, gave more results than using the Gasch data set [21], a compendium of 176 stress conditions, which gave no results at all when compared to the Bozdech data set, and very few when compared to the Le Roch data set. Thus, while it is possible that the potential of the co-coexpression analysis as a strategy to assist in the functional inference of hypothetical genes may be exhausted after covering a limited number of conserved eukaryotic housekeeping processes, it may also benefit from two improvements: 1) using species that are phylogenetically less distant, ideally, other *Apicomplexans*, and 2) comparing profiling studies of biological situations specifically chosen to answer specific questions. For example, comparing *Arabidopsis thaliana *to *P. falciparum *transcriptomic data could help uncover genes inherited from the ancestral algae [9].

### Comparison with the GBA approach

**Figure 4:**
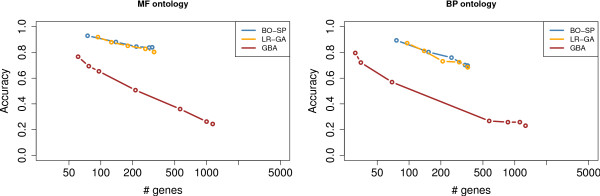
**Comparison with a GBA approach**. Accuracy achieved by the OPI approach (brown curves) on the MF (left) and BP (right) ontologies when using different FDR thresholds. The orange and blue curves show the accuracy achieved on the Le Roch-Gasch and Bozdech-Spellman experiments, respectively.

During the writing of this article, Zhou et al. (2008) [15] published a database of GO functional predictions for several *P. falciparum *genes based on a Guilt By Association method named OPI. Their predictions are based on a transcriptomic data set covering all life cycle stages of the parasite and combining gene expression measurements from both *P. falciparum *and *P. yoelii*. The OPI approach provides us with an interesting reference to compare annotation transfers allowed by co-coexpression and GBA. To this end, we first estimated the global accuracy of the OPI method using GO. OPI provides for each GO term an estimate of the False Discovery Rate (FDR) associated with the term. For example, a GO term with a FDR of 20% means that all predictions in this term have an error probability of 20%. We used this FDR as a cutoff to control the accuracy and the number of OPI annotation predictions. Namely, all GO terms with FDR below the cutoff are considered, and the already annotated genes predicted in these terms by OPI are used to estimate the global accuracy of the method in a way similar to that used to assess the accuracy of our approach (see Methods). Figure 4 shows the OPI results achieved when varying the FDR threshold, as well as the accuracy of the already described LR-GA and BO-SP experiments. As we can see on this figure, the results obtained with expression data alone are not as accurate as those obtained when also using sequence information.

Note that in reference [15], the authors also further corroborated some of their predictions by applying the OPI method to known orthologous genes of *P. falciparum *in *S. cerevisiae *and *Homo sapiens *(using yeast and *Homo sapiens *microarray gene expression data sets), and checking that these genes are predicted in the same GO terms as their *P. falciparum *orthologs. This approach to combine sequence and gene expression data for purpose of annotation transfer is different from ours. Zhou et al. rely on the GBA principle to transfer the annotations in an intra-species way; they use another organism to validate some of their predictions by running a second independent GBA procedure. On the other hand, we use conserved co-expression to assess inter-species annotation transfers suggested by borderline sequence similarity.

Finally, we investigated whether the results, collected here by co-coexpression analysis between *P. falciparum*, *S. cerevisiae *and *D. melanogaster *overlapped the results obtained by Zhou et al. (2008) [15], but also the results of previous GBA studies on *P. falciparum *[14,47]. Our work allowed a systematic revision of the annotation of ribosomal proteins, and of proteins involved in ribosome biogenesis. From the "hypothetical" genes of this series, OPI proposes consistent annotations for one of the genes coding for ribosomal proteins (*PF07_121*) and 3 genes involved in ribosome biogenesis and assembly (*PF14_0055*, *PF11_090 *and *PF11_0471*). These annotations were further corroborated by applying OPI on the same yeast genes as those presented here. In the case of *PF07_121*, this gene was reported in one of the expression clusters in the study of Le Roch et al (2003), although it was not annotated at that time. Of the other 43 hypothetical genes that have been functionally annotated here, none was attributed any function in previous studies by Le Roch et al. (2003) [14], Young et al (2005) [47] and Zhou et al (2008) [15], either because they were not part of expression clusters in their analyses or because the homology searches were not conclusive.

## Conclusions

We propose a method that uses coexpression to annotate genes in an inter-species way. Classical GBA approaches work in an intra-species way, by transferring annotations between coexpressed genes. Our approach searches for conserved coexpression between a query species and a reference species, and uses this information to increase confidence of annotation transfers between genes with borderline sequence similarity. Our analyses show that this strategy improves the accuracy achieved when using sequence similarity alone. It also outperforms the accuracy of classical GBA approaches. Results achieved on *P. falciparum *allowed us to pool and review series of genes involved in particular processes such as ribosomal complex elaboration and biogenesis. Notably, it allowed us to highlight potential drug targets. We observed high representation of certain biological functions and an apparent lack of other functions. The potential of such an analysis appears to depend on the choice of both the species compared and of the biological conditions screened by the microarray data. Nonetheless, results on *P. falciparum *show that the approach has high accuracy and potentially allows a function to be inferred for several unknown genes.

## Methods

### The model

HMMs are probabilistic models that are widely used in biological sequence modeling to recognize protein families and predict gene models [28]. Here they are used for modeling pairs of gene expression profiles. Our HMM has a predefined structure with a mute state *M*, as depicted in Figure 1, and a set of numerical parameters Θ. Each prototype is parameterized by a multivariate normal distribution of dimension equal to the profile sizes—*i.e*., the number of time points (or experiments) of the gene expression profiles. In our experiments, we restricted the covariance matrices to be diagonal and equal for all prototypes of one species (homoscedastic assumption). Moreover, there is a prior probability distribution on the prototypes of the query species, and a probability distribution on the outgoing transitions from each of these prototypes and from the *M *state.

### Assessing functional conservation

We assessed the transfer of function of numerous gene pairs between *S. cerevisiae*/*D. melanogaster *and *P. falciparum*. We used the BLASTP software of NCBI with default parameters to measure sequence similarity between genes. All gene pairs between the query and the reference species with an e-value below 0.1 were considered. For query genes possessing several homologs in the reference species, the BLAST bit scores (used to compute the e-value) were compared, and gene pairs with scores under 95% of the largest one were removed. This allowed us to associate at least one gene in *S. cerevisiae *and *D. melanogaster *with 3 664 and 3 115 genes of *P. falciparum*, respectively. As some of these *P. falciparum *genes are actually associated with more than one gene in each reference species, the total number gene pairs considered was 5 345 between *P. falciparum *and *S. cerevisiae*, and 3 904 between *P. falciparum *and *D. melanogaster*. For each gene pair, the classical Viterbi algorithm [27] was used to search the prototypes *k *and *k' *that have the highest probability of generating the associated gene expression profiles. Then, each pair for which the ratio *P*(*k' *|*k*)/*P*(*k' *) was higher than the threshold ξ = 10 was considered eligible for a functional transfer.

### Learning the model

The learning algorithm takes the number of prototypes *K *and *K' *for the two species as parameters—in the experiments we used *K *= *K' *= 100. It is applied to a learning set ℒ of gene pairs selected by Reciprocal Best Hit (RBH). The procedure involves associating with each gene of the query (respectively reference) species the gene in the reference (resp. query) species that most resembles it, and identifying the gene pairs *g *- *g' *such that *g *is the best hit for *g'*, and *g' *is the best hit for *g*. We obtained 1 427 and 1 855 aligned pairs of sequences between *P. falciparum *and *S. cerevisiae*, and *P. falciparum *and *D. melanogaster*, respectively.

Learning the HMM involves to learn both its structure (*i.e*. the presence/absence of direct transitions between prototypes), and parameters Θ (*i.e*. parameters of the Gaussian models, prior probabilities of the prototypes, and transition probabilities). Given a predefined structure, the second problem can be handled with the classical *Baum-Welch algorithm *[29] used for training the HMMs. Solving the former problem involves deciding, for each prototype pair (*k*, *k' *), if it involves conserved coexpressed genes. To this end, an initial clustering in *K *and *K' *clusters is computed by running the K-MEANS algorithm [25] on the expression data of each species. Then, for each cluster pair (*k*, *k' *), the number of gene pairs of ℒ involving genes assigned to clusters *k *and *k' *by the K-MEANS algorithm is computed, and a statistical test is applied to test if this number can be expected by chance (see below). When this is not the case (the null hypothesis is rejected), the two clusters are considered to reveal conserved coexpression between the two species, and a transition is added in the HMM from prototype *k *to prototype *k'*. At the end of the process, the HMM structure has been built.

The Baum-Welch algorithm is then run to train this structure. It is applied to the pairs of expression profiles in ℒ and aims at maximizing the model likelihood:(1)

with *P*(*g*, *g' *|Θ) being the probability of generating the expression profiles associated with genes *g *and *g' *with the HMM with parameters Θ. Thus, the Baum-Welch algorithm searches for the parameters Θ that maximizes Expression (1). It is an iterative algorithm, which starts from an initial set of parameter Θ^0^, and iteratively reestimates the parameters at each step of the process. We use the initial K-MEANS clusterings to set parameters Θ^0^. Namely, means and covariance matrices of the Gaussian distributions are estimated from the profiles of the genes associated with each prototype. Prior probabilities of the prototypes are estimated by the proportion of genes associated with, and probability transitions between prototypes are estimated by the proportion of genes whose coexpression has been conserved.

### Testing for coexpression conservation

Let *k *and *k' *be two gene clusters for which we want to know if they reveal conserved coexpression. For this purpose, we designed a statistical test that uses the number *n*_*kk*' _of gene pairs of ℒ between the two clusters. More precisely, we compute the probability of observing *n*_*kk*' _or more gene pairs of ℒ between cluster *k *and a randomly composed cluster of size #*k'*. This p-value can be written as(2)

with *P*(*r *= *u*|ℒ, *k*, #*k'*) being the probability of observing *u *gene pairs of ℒ between cluster *k *and a randomly composed cluster of size #*k'*. Let *G' *be the number of genes of the reference species. We can mark the genes of the reference species that are paired with a gene of *k *in ℒ. The probability *P*(*r *= *u*|ℒ, *k*, #*k' *) is actually the probability of finding *u *marked genes when picking #*k' *genes among the *G' *genes, and thus follows an hypergeometric distribution. In our experiments, all cluster pairs that get a p-value below 10^-3^, as computed by Expression (2), are considered to reveal conserved coexpression, and a direct transition is added in the HMM between the associated prototypes.

### Accuracy estimate on the Gene Ontology

Each ontology (MF, BP or CC) is a pseudo-hierarchy describing generalization relationships between hundreds of terms. The most general term is at the top of the ontology, while the terms at the bottom are the most specific ones. The specificity of a term can be assessed by its depth in the ontology, *i.e*. the number of levels that separate this term from the top. In the following, we consider a term as "specific" if it is deeper than the 4th level of the ontology. Note that a gene may be annotated with several GO terms of the same ontology. Moreover, due to the generalization relationship, when a gene is annotated with a term *t*, it is also annotated with all the upper level terms that generalize *t *(a principle known as the *"true path rule" *in GO context). Let *g *be a gene of the query species already annotated in a given ontology (for example BP), and let **A **be the "specific" annotations associated with *g *in this ontology. Now, let **A' **be the set of specific annotations transferred to *g *by one of the methods assessed in the experiments. Four different methods are tested: the RBH procedure, the best BLAST procedure, the co-coexpression approach, and OPI. For the first three approaches, **A' **is the set of specific annotations associated with the reference gene used for the transfer; for the OPI approach, **A' **is the set of specific annotations (plus the specific annotations of the upper levels) that have been predicted for *g *with a FDR below the cutoff. The transfer accuracy is estimated by the proportion of annotations of **A' **that are in **A**, *i.e*. , with |·| denoting the cardinal number of a set. Transfers involving the same specific annotations have accuracy 1, while those involving completely different specific annotations have accuracy 0. The global accuracy of a method is then estimated by averaging the accuracy of all transfers involving a query gene already annotated in the ontology. We used annotations available on the GO website and provided by GeneDB http://www.genedb.org/, the *Saccharomyces Genome Database *http://www.yeastgenome.org/, and *FlyBase *http://flybase.bio.indiana.edu/ for *P. falciparum*, *S. cerevisiae*, and *D. melanogaster*, respectively.

## Authors' contributions

LB conceived, designed and implemented the method, carried out the experiments, and drafted the manuscript. IF and EM designed and analyzed the experiments and drafted the manuscript. OG revised the manuscript and initiated the project. All authors read and approved the final manuscript.

## Supplementary Material

Additional file 1 **Le Roch - Gasch analysis**. This file presents the cluster pairs identified as revealing a conservation of coexpression when comparing the Le Roch and Gasch data. This file also provide additional information on the available functional annotations, as well as links to the BLAST alignments and the different databases (click on the '?'s to access PlasmoDB, SGD, and Amigo databases). Gene functional annotations are as follows. The short description immediately following each *P. falciparum *gene comes from PlasmoDB (red = functional gene, blue = putative gene, black = hypothetical gene). Other annotations are Gene Ontology annotations (red = Molecular Function, green = Biological Process, blue = Cellular Component). Click here for file

Additional file 2 **Le Roch - Spellman analysis**. This file presents the cluster pairs identified as revealing a conservation of coexpression when comparing the Le Roch and Spellman data. This file also provide additional information on the available functional annotations, as well as links to the BLAST alignments and the different databases (click on the '?'s to access PlasmoDB, SGD, and Amigo databases). Gene functional annotations are as follows. The short description immediately following each *P. falciparum *gene comes from PlasmoDB (red = functional gene, blue = putative gene, black = hypothetical gene). Other annotations are Gene Ontology annotations (red = Molecular Function, green = Biological Process, blue = Cellular Component). Click here for file

Additional file 3 **Bozdech - Gasch analysis**. This file presents the cluster pairs identified as revealing a conservation of coexpression when comparing the Bozdech and Gasch data. This file also provide additional information on the available functional annotations, as well as links to the BLAST alignments and the different databases (click on the '?'s to access PlasmoDB, SGD, and Amigo databases). Gene functional annotations are as follows. The short description immediately following each *P. falciparum *gene comes from PlasmoDB (red = functional gene, blue = putative gene, black = hypothetical gene). Other annotations are Gene Ontology annotations (red = Molecular Function, green = Biological Process, blue = Cellular Component). Click here for file

Additional file 4 **Bozdech - Spellman analysis**. This file presents the cluster pairs identified as revealing a conservation of coexpression when comparing the Bozdech and Spellman data. This file also provide additional information on the available functional annotations, as well as links to the BLAST alignments and the different databases (click on the '?'s to access PlasmoDB, SGD, and Amigo databases). Gene functional annotations are as follows. The short description immediately following each *P. falciparum *gene comes from PlasmoDB (red = functional gene, blue = putative gene, black = hypothetical gene). Other annotations are Gene Ontology annotations (red = Molecular Function, green = Biological Process, blue = Cellular Component). Click here for file

Additional file 5 **Bozdech - Pilot analysis**. This file presents the cluster pairs identified as revealing a conservation of coexpression when comparing the Bozdech and Pilot data. This file also provide additional information on the available functional annotations, as well as links to the BLAST alignments and the different databases (click on the '?'s to access PlasmoDB, FlyBase, and Amigo databases). Gene functional annotations are as follows. The short description immediately following each *P. falciparum *gene comes from PlasmoDB (red = functional gene, blue = putative gene, black = hypothetical gene). Other annotations are Gene Ontology annotations (red = Molecular Function, green = Biological Process, blue = Cellular Component). Click here for file

Additional file 6 **Predicted annotations of *P. falciparum *gene coding for putative ribosomal proteins (structural constituent of ribosomes, GO:0003735)**. Prediction gained by co-coexpression analyses compared to PlasmoDB 5.4: (0) confirmed annotation; (1) refined annotation of an incomplete or wrong original functional inference; (2) previously hypothetical; (N): no advance based on co-coexpression analyses; (+) functional inference of hypothetical gene products by Smits et al. (2007) [32]; (*) correction of Smits et al. (2007) annotation; (Z) pair also identified in Zhou et al. (2008) [15]; (x): pairing of *P. falciparum *and *S. cerevisiae *or *P. falciparum *and *D. melanogaster *genes in co-coexpression analyses. Click here for file

Additional file 7 **Predicted annotations of *P. falciparum *gene products involved in ribosome biogenesis and assembly (GO:0042254), rRNA metabolic process (GO:0016072) and tRNA processing (GO:0008033)**. Prediction gained by co-coexpression analyses when compared to PlasmoDB 5.4: (0) confirmed annotation; (1) refined annotation of an incomplete or wrong original functional inference; (2) previously hypothetical; (Z) pair also identified in Zhou et al. (2008) [15]. (x): pairing of *P. falciparum *and *S. cerevisiae *or *P. falciparum *and *D. melanogaster *genes in co-coexpression analyses. (§): pairing of *P. falciparum *and *S. cerevisiae *genes in co-coexpression with different clustering parameters. (*): Correction of the gene models of *PF14_0436 *and *PF14_0437*, forming a unique gene coding for a putative nucleolar DEAD/DEAH box ATP-dependent RNA helicase (see Add. File 8). Click here for file

Additional file 8 **Correction of the gene structures and functional inference of *PF14_0436 *and *PF14_0437 *based on *P. falciparum *vs. *S. cerevisiae *co-coexpression analyses**. In co-coxpression analyses, both *PF14_0436 *and *PF14_0437 *were in the same expression cluster and with the same yeast sequence, *YNL112W*, involved in ribosome biogenesis and assembly (GO:0042254, see Add. File 7). Sequence comparison of these contiguous predicted open reading frames shows that they align with both extremities of *YNL112W*, and that their overlapping region is an error of the gene model, corrected in updated versions of PlasmoDB. Click here for file

Additional file 9 **Other annotations of *P. falciparum *gene products based on co-coexpression analyses**. Prediction gained by co-coexpression analyses when compared to PlasmoDB 5.4: (0) confirmed annotation; (1) refined annotation of an incomplete or wrong original functional inference; (2) previously hypothetical; (Z) pair also identified in Zhou et al. (2008) [15]. (x): pairing of *P. falciparum *and *S. cerevisiae *or *P. falciparum *and *D. melanogaster *genes in co-coexpressed analyses. (§): pairing of *P. falciparum *and *S. cerevisiae *genes in co-coexpression with different clustering parameters. This table summarizes important functional annotation predicted for *P. falciparum *genes previously reported as "hypotheticals" or with little indication of a putative function. Click here for file
