# Supplementary material for: Assessing functional annotation transfers with inter-species conserved coexpression: application to Plasmodium falciparum
Source: BMC Genomics. 2010 Jan 15;11:35. doi: 10.1186/1471-2164-11-35 (PMC2826313; doi:10.1186/1471-2164-11-35)
Supplement: Additional file 1 — Le Roch - Gasch analysis. This file presents the cluster pairs identified as revealing a conservation of coexpression when comparing the Le Roch and Gasch data. This file also provide additional information on the available functional annotations, as well as links to the BLAST alignments and the different databases (click on the '?'s to access PlasmoDB, SGD, and Amigo databases). Gene functional annotations are as follows. The short description immediately following each P. falciparum gene comes from PlasmoDB (red = functional gene, blue = putative gene, black = hypothetical gene). Other annotations are Gene Ontology annotations (red = Molecular Function, green = Biological Process, blue = Cellular Component). [file 1471-2164-11-35-S1.HTML]

# Leroch-Gasch co-coexpression analysis

# 24 cluster pairs

## Cluster Pair #0: 30 gene pairs.

P.falciparum | S.cerevisiae | Blast evalue || PFB0860c ? RNA helicase, putative  nucleic acid binding (IEA) ? ATP-dependent RNA helicase activity ? helicase activity (IEA) ? ATP binding (IEA) ? ATP-dependent helicase activity (IEA) ? | YHR065C ? ATP-dependent RNA helicase activity ? nucleolus ? 35S primary transcript processing ? ribosome biogenesis and assembly ? | 0  BLAST |
| PF11\_0471 ? hypothetical protein | YCR072C ? ribosomal large subunit assembly and maintenance ? nucleolus ? ribosome ? ribosome biogenesis and assembly ? | 2.8026e-45  BLAST |
| PFE1240w ? hypothetical protein, conserved  catalytic activity (IEA) ? iron ion binding (IEA) ? | YPL207W ? endoplasmic reticulum ? | 0  BLAST |
| PFF1500c ? DEAD%2FDEAH box ATP-dependent RNA helicase, putative  nucleic acid binding (IEA) ? ATP-dependent RNA helicase activity ? helicase activity (IEA) ? ATP binding (IEA) ? ATP-dependent helicase activity (IEA) ? RNA metabolism ? | YMR290C ? RNA binding ? ATP-dependent RNA helicase activity ? nuclear membrane ? nucleolus ? rRNA processing ? RNA-dependent ATPase activity ? ribosome biogenesis and assembly ? | 0  BLAST |
| PF13\_0109 ? N2,N2-dimethylguanosine tRNA methyltransferase, putative  RNA binding (IEA) ? tRNA (guanine-N2-)-methyltransferase activity ? tRNA modification ? tRNA processing (IEA) ? apicoplast ? | YDR120C ? tRNA (guanine-N2-)-methyltransferase activity ? nuclear membrane ? nuclear inner membrane ? mitochondrion ? tRNA methylation ? ribosome biogenesis and assembly ? | 3e-13  BLAST |
| PF11\_0445 ? DNA-directed RNA polymerase I, putative  DNA binding (IEA) ? DNA-directed RNA polymerase activity (IEA) ? transcription (IEA) ? protein dimerization activity (IEA) ? | YPR110C ? DNA-directed RNA polymerase activity ? DNA-directed RNA polymerase III complex ? DNA-directed RNA polymerase I complex ? transcription from RNA polymerase I promoter ? transcription from RNA polymerase III promoter ? ribosome biogenesis and assembly ? | 9.94922e-44  BLAST |
| PF07\_0121 ? hypothetical protein, conserved | YHR170W ? ribosomal large subunit assembly and maintenance ? ribosomal large subunit-nucleus export ? RNA binding ? protein binding ? cytosol ? cytosolic large ribosomal subunit (sensu Eukaryota) ? ribosome biogenesis and assembly ? | 2.94273e-44  BLAST |
| PF11\_0358 ? DNA-directed RNA polymerase, beta subunit, putative  DNA binding ? DNA-directed RNA polymerase activity ? DNA-directed RNA polymerase I complex ? transcription (IEA) ? transcription from RNA polymerase I promoter ? | YPR010C ? DNA-directed RNA polymerase activity ? DNA-directed RNA polymerase I complex ? transcription from RNA polymerase I promoter ? ribosome biogenesis and assembly ? | 0  BLAST |
| PF10\_0200 ? hypothetical protein, conserved | YNL132W ? nucleolus ? ribosome biogenesis and assembly ? | 2e-39  BLAST |
| PF08\_0123 ? hypothetical protein | YPL212C ? nucleus ? tRNA modification ? tRNA-pseudouridine synthase activity ? ribosome biogenesis and assembly ? | 2e-17  BLAST |
| PF13\_0261 ? ATP binding protein, putative  ATP binding ? | YLR243W ? signal sequence binding ? | 2e-24  BLAST |
| PF14\_0185 ? ATP-dependent RNA helicase, putative  nucleic acid binding (IEA) ? ATP-dependent RNA helicase activity ? helicase activity (IEA) ? ATP binding (IEA) ? ATP-dependent helicase activity (IEA) ? \*\* also with: YFL002C, clust.pair #0 \*\* also with: YHR065C, clust.pair #0 \*\* also with: YGL078C, clust.pair #0 \*\* also with: YJL033W, clust.pair #0 | YMR290C ? RNA binding ? ATP-dependent RNA helicase activity ? nuclear membrane ? nucleolus ? rRNA processing ? RNA-dependent ATPase activity ? ribosome biogenesis and assembly ? | 5e-10  BLAST |
| PF14\_0185 ? ATP-dependent RNA helicase, putative  nucleic acid binding (IEA) ? ATP-dependent RNA helicase activity ? helicase activity (IEA) ? ATP binding (IEA) ? ATP-dependent helicase activity (IEA) ? \*\* also with: YMR290C, clust.pair #0 \*\* also with: YHR065C, clust.pair #0 \*\* also with: YGL078C, clust.pair #0 \*\* also with: YJL033W, clust.pair #0 | YFL002C ? ribosomal large subunit assembly and maintenance ? ATP-dependent RNA helicase activity ? nucleolus ? 35S primary transcript processing ? ribosome biogenesis and assembly ? | 6e-10  BLAST |
| PF14\_0185 ? ATP-dependent RNA helicase, putative  nucleic acid binding (IEA) ? ATP-dependent RNA helicase activity ? helicase activity (IEA) ? ATP binding (IEA) ? ATP-dependent helicase activity (IEA) ? \*\* also with: YMR290C, clust.pair #0 \*\* also with: YFL002C, clust.pair #0 \*\* also with: YGL078C, clust.pair #0 \*\* also with: YJL033W, clust.pair #0 | YHR065C ? ATP-dependent RNA helicase activity ? nucleolus ? 35S primary transcript processing ? ribosome biogenesis and assembly ? | 1e-09  BLAST |
| PF14\_0185 ? ATP-dependent RNA helicase, putative  nucleic acid binding (IEA) ? ATP-dependent RNA helicase activity ? helicase activity (IEA) ? ATP binding (IEA) ? ATP-dependent helicase activity (IEA) ? \*\* also with: YMR290C, clust.pair #0 \*\* also with: YFL002C, clust.pair #0 \*\* also with: YHR065C, clust.pair #0 \*\* also with: YJL033W, clust.pair #0 | YGL078C ? ribosomal large subunit assembly and maintenance ? ATP-dependent RNA helicase activity ? nucleolus ? 35S primary transcript processing ? | 2e-08  BLAST |
| PF14\_0185 ? ATP-dependent RNA helicase, putative  nucleic acid binding (IEA) ? ATP-dependent RNA helicase activity ? helicase activity (IEA) ? ATP binding (IEA) ? ATP-dependent helicase activity (IEA) ? \*\* also with: YMR290C, clust.pair #0 \*\* also with: YFL002C, clust.pair #0 \*\* also with: YHR065C, clust.pair #0 \*\* also with: YGL078C, clust.pair #0 | YJL033W ? ATP-dependent RNA helicase activity ? nucleolus ? 35S primary transcript processing ? ribosome biogenesis and assembly ? | 2e-08  BLAST |
| PF13\_0177 ? ATP-dependent RNA helicase, putative  nucleic acid binding (IEA) ? helicase activity (IEA) ? ATP binding (IEA) ? ATP-dependent helicase activity (IEA) ? \*\* also with: YHR169W, clust.pair #5 | YHR065C ? ATP-dependent RNA helicase activity ? nucleolus ? 35S primary transcript processing ? ribosome biogenesis and assembly ? | 0  BLAST |
| PF11\_0090 ? hypothetical protein  intracellular (IEA) ? nucleolus (IEA) ? cell proliferation (IEA) ? | YGR103W ? nucleus ? nucleolus ? cell cycle ? cell proliferation ? processing of 20S pre-rRNA ? ribosome biogenesis and assembly ? ribosomal large subunit biogenesis ? | 0  BLAST |
| MAL8P1.19 ? hypothetical protein, conserved  nucleic acid binding (IEA) ? helicase activity (IEA) ? ATP binding (IEA) ? ATP-dependent helicase activity (IEA) ? apicoplast ? \*\* also with: YLL008W, clust.pair #0 \*\* also with: YMR290C, clust.pair #0 \*\* also with: YGL078C, clust.pair #0 \*\* also with: YDL031W, clust.pair #0 | YGL171W ? ATP-dependent RNA helicase activity ? nucleolus ? 35S primary transcript processing ? ATPase activity ? | 8e-34  BLAST |
| MAL8P1.19 ? hypothetical protein, conserved  nucleic acid binding (IEA) ? helicase activity (IEA) ? ATP binding (IEA) ? ATP-dependent helicase activity (IEA) ? apicoplast ? \*\* also with: YGL171W, clust.pair #0 \*\* also with: YMR290C, clust.pair #0 \*\* also with: YGL078C, clust.pair #0 \*\* also with: YDL031W, clust.pair #0 | YLL008W ? ribosomal large subunit assembly and maintenance ? ATP-dependent RNA helicase activity ? nucleolus ? 35S primary transcript processing ? ribosome biogenesis and assembly ? | 7e-32  BLAST |
| MAL8P1.19 ? hypothetical protein, conserved  nucleic acid binding (IEA) ? helicase activity (IEA) ? ATP binding (IEA) ? ATP-dependent helicase activity (IEA) ? apicoplast ? \*\* also with: YGL171W, clust.pair #0 \*\* also with: YLL008W, clust.pair #0 \*\* also with: YGL078C, clust.pair #0 \*\* also with: YDL031W, clust.pair #0 | YMR290C ? RNA binding ? ATP-dependent RNA helicase activity ? nuclear membrane ? nucleolus ? rRNA processing ? RNA-dependent ATPase activity ? ribosome biogenesis and assembly ? | 2e-31  BLAST |
| MAL8P1.19 ? hypothetical protein, conserved  nucleic acid binding (IEA) ? helicase activity (IEA) ? ATP binding (IEA) ? ATP-dependent helicase activity (IEA) ? apicoplast ? \*\* also with: YGL171W, clust.pair #0 \*\* also with: YLL008W, clust.pair #0 \*\* also with: YMR290C, clust.pair #0 \*\* also with: YDL031W, clust.pair #0 | YGL078C ? ribosomal large subunit assembly and maintenance ? ATP-dependent RNA helicase activity ? nucleolus ? 35S primary transcript processing ? | 1e-30  BLAST |
| MAL8P1.19 ? hypothetical protein, conserved  nucleic acid binding (IEA) ? helicase activity (IEA) ? ATP binding (IEA) ? ATP-dependent helicase activity (IEA) ? apicoplast ? \*\* also with: YGL171W, clust.pair #0 \*\* also with: YLL008W, clust.pair #0 \*\* also with: YMR290C, clust.pair #0 \*\* also with: YGL078C, clust.pair #0 | YDL031W ? ribosomal large subunit assembly and maintenance ? ATP-dependent RNA helicase activity ? nucleolus ? 35S primary transcript processing ? ribosome biogenesis and assembly ? | 5e-30  BLAST |
| PF10\_0278 ? hypothetical protein, conserved | YKR081C ? ribosomal large subunit assembly and maintenance ? nucleolus ? 5S rRNA binding ? 7S RNA binding ? rRNA binding ? processing of 27S pre-rRNA ? ribosome biogenesis and assembly ? | 2e-05  BLAST |
| MAL8P1.92 ? ATPase, putative  nucleotide binding (IEA) ? ATP binding (IEA) ? ATPase activity ? nucleoside-triphosphatase activity (IEA) ? | YLL034C ? ribosomal large subunit-nucleus export ? nucleus ? nucleolus ? ATPase activity ? | 0  BLAST |
| PF13\_0341 ? DNA-directed RNA polymerase 2, putative  DNA binding ? DNA-directed RNA polymerase activity ? DNA-directed RNA polymerase II, core complex ? transcription (IEA) ? transcription from RNA polymerase II promoter ? | YBR154C ? DNA-directed RNA polymerase activity ? DNA-directed RNA polymerase II, core complex ? DNA-directed RNA polymerase III complex ? DNA-directed RNA polymerase I complex ? transcription from RNA polymerase I promoter ? transcription from RNA polymerase II promoter ? transcription from RNA polymerase III promoter ? | 3e-37  BLAST |
| PF14\_0183 ? RNA helicase, putative  nucleic acid binding (IEA) ? ATP-dependent RNA helicase activity ? helicase activity (IEA) ? ATP binding (IEA) ? ATP-dependent helicase activity (IEA) ? | YJL033W ? ATP-dependent RNA helicase activity ? nucleolus ? 35S primary transcript processing ? ribosome biogenesis and assembly ? | 0  BLAST |
| PF14\_0072 ? hypothetical protein, conserved | YNR046W ? tRNA (guanine-N2-)-methyltransferase activity ? nucleus ? nucleolus ? cytoplasm ? zinc ion binding ? tRNA methylation ? | 2e-09  BLAST |
| PFI0635c ? hypothetical protein | YML093W ? small nucleolar ribonucleoprotein complex ? processing of 20S pre-rRNA ? snoRNA binding ? ribosome biogenesis and assembly ? | 0.01  BLAST |
| PF13\_0256 ? hypothetical protein | YCL037C ? RNA binding ? polysome ? protein biosynthesis ? ribosome biogenesis and assembly ? | 0.092  BLAST |

## Cluster Pair #1: 7 gene pairs.

| P.falciparum | S.cerevisiae | Blast evalue |
| --- | --- | --- |
| PF14\_0473 ? 3%27-5%27 exonuclease, putative  nucleic acid binding (IEA) ? intracellular (IEA) ? 3'-5' exonuclease activity (IEA) ? | YOR001W ? 3'-5'-exoribonuclease activity ? nuclear exosome (RNase complex) ? 35S primary transcript processing ? mRNA catabolism ? ribosome biogenesis and assembly ? | 1e-12  BLAST |
| PF11\_0353 ? hypothetical protein | YNL227C ? ATPase stimulator activity ? cytoplasm ? mitochondrion ? cytosol ? cytosolic large ribosomal subunit (sensu Eukaryota) ? endocytosis ? regulation of cell size ? Hsp70/Hsc70 protein regulator activity ? ribosomal large subunit biogenesis ? | 0.038  BLAST |
| PF14\_0677 ? RNA 3%27-Terminal Phosphate Cyclase-like protein, putative  RNA-3'-phosphate cyclase activity (IEA) ? ribosome biogenesis ? ribosome biogenesis and assembly ? | YOL010W ? RNA-3'-phosphate cyclase activity ? nucleolus ? 35S primary transcript processing ? ribosome biogenesis and assembly ? | 1e-11  BLAST |
| PF14\_0436 ? helicase, truncated, putative  nucleic acid binding (IEA) ? helicase activity ? ATP binding (IEA) ? ATP-dependent helicase activity (IEA) ? | YNL112W ? mRNA catabolism, nonsense-mediated decay ? RNA helicase activity ? nucleus ? cytoplasm ? mitochondrion ? 35S primary transcript processing ? | 0  BLAST |
| PF14\_0661 ? hypothetical protein, conserved  nucleic acid binding (IEA) ? | YOR145C ? nucleus ? nucleolus ? rRNA processing ? 35S primary transcript processing ? protein complex assembly ? ribosome biogenesis and assembly ? unfolded protein binding ? | 0  BLAST |
| PF08\_0053 ? hypothetical protein  \*\* also with: YHR066W, clust.pair #1 | YDR312W ? ribosomal large subunit assembly and maintenance ? conjugation with cellular fusion ? nucleolus ? rRNA binding ? ribosome biogenesis and assembly ? | 0.001  BLAST |
| PF08\_0053 ? hypothetical protein  \*\* also with: YDR312W, clust.pair #1 | YHR066W ? ribosomal large subunit assembly and maintenance ? conjugation with cellular fusion ? nucleolus ? regulation of cell size ? rRNA binding ? | 0.003  BLAST |

## Cluster Pair #2: 10 gene pairs.

| P.falciparum | S.cerevisiae | Blast evalue |
| --- | --- | --- |
| MAL13P1.93 ? hypothetical protein | YPR144C ? nucleus ? nucleolus ? small nucleolar ribonucleoprotein complex ? 35S primary transcript processing ? Noc4p-Nop14p complex ? ribosome biogenesis and assembly ? ribosomal small subunit biogenesis ? | 0.009  BLAST |
| PF10\_0087 ? diphthine synthase  diphthine synthase activity ? metabolism (IEA) ? methyltransferase activity (IEA) ? peptidyl-diphthamide biosynthesis from peptidyl-histidine (IEA) ? | YLR172C ? diphthine synthase activity ? cytoplasm ? peptidyl-diphthamide biosynthesis from peptidyl-histidine ? | 0  BLAST |
| PF14\_0429 ? RNA helicase, putative  nucleic acid binding (IEA) ? helicase activity ? ATP binding (IEA) ? ATP-dependent helicase activity (IEA) ? \*\* also with: YJL033W, clust.pair #2 \*\* also with: YMR290C, clust.pair #2 \*\* also with: YHR065C, clust.pair #2 | YLL008W ? ribosomal large subunit assembly and maintenance ? ATP-dependent RNA helicase activity ? nucleolus ? 35S primary transcript processing ? ribosome biogenesis and assembly ? | 4e-30  BLAST |
| PF14\_0429 ? RNA helicase, putative  nucleic acid binding (IEA) ? helicase activity ? ATP binding (IEA) ? ATP-dependent helicase activity (IEA) ? \*\* also with: YLL008W, clust.pair #2 \*\* also with: YMR290C, clust.pair #2 \*\* also with: YHR065C, clust.pair #2 | YJL033W ? ATP-dependent RNA helicase activity ? nucleolus ? 35S primary transcript processing ? ribosome biogenesis and assembly ? | 2e-29  BLAST |
| PF14\_0429 ? RNA helicase, putative  nucleic acid binding (IEA) ? helicase activity ? ATP binding (IEA) ? ATP-dependent helicase activity (IEA) ? \*\* also with: YLL008W, clust.pair #2 \*\* also with: YJL033W, clust.pair #2 \*\* also with: YHR065C, clust.pair #2 | YMR290C ? RNA binding ? ATP-dependent RNA helicase activity ? nuclear membrane ? nucleolus ? rRNA processing ? RNA-dependent ATPase activity ? ribosome biogenesis and assembly ? | 8e-29  BLAST |
| PF14\_0429 ? RNA helicase, putative  nucleic acid binding (IEA) ? helicase activity ? ATP binding (IEA) ? ATP-dependent helicase activity (IEA) ? \*\* also with: YLL008W, clust.pair #2 \*\* also with: YJL033W, clust.pair #2 \*\* also with: YMR290C, clust.pair #2 | YHR065C ? ATP-dependent RNA helicase activity ? nucleolus ? 35S primary transcript processing ? ribosome biogenesis and assembly ? | 1e-28  BLAST |
| PFA0330w ? pfAARP2 protein  plasma membrane ? | YPL217C ? GTP binding ? nucleus ? nucleolus ? cytoplasm ? mitochondrion ? rRNA processing ? 35S primary transcript processing ? ribosome assembly ? | 1.4013e-45  BLAST |
| PFF0695w ? hypothetical protein, conserved | YPR112C ? nucleolus ? 35S primary transcript processing ? snoRNA binding ? rRNA primary transcript binding ? | 0.056  BLAST |
| PF14\_0663 ? hypothetical protein  metalloendopeptidase activity (IEA) ? proteolysis and peptidolysis (IEA) ? pathogenesis (IEA) ? metal ion binding (IEA) ? | YDL167C ? cytoplasm ? ribosome biogenesis and assembly ? | 3e-05  BLAST |
| PFE1215c ? developmentally regulated GTP-binding protein 1, putative  GTP binding (IEA) ? | YAL036C ? GTP binding ? cytoplasm ? ribosome biogenesis and assembly ? | 0  BLAST |

## Cluster Pair #3: 27 gene pairs.

| P.falciparum | S.cerevisiae | Blast evalue |
| --- | --- | --- |
| PFF0940c ? cell division cycle protein 48 homologue, putative  regulation of progression through cell cycle ? nucleotide binding (IEA) ? ATP-dependent peptidase activity (IEA) ? serine-type endopeptidase activity (IEA) ? ATP binding (IEA) ? microsome ? proteolysis and peptidolysis (IEA) ? hydrolase activity (IEA) ? ATPase activity ? nucleoside-triphosphatase activity (IEA) ? | YDL126C ? nucleus ? endoplasmic reticulum membrane ? microsome ? cytosol ? ubiquitin-dependent protein catabolism ? vesicle fusion ? apoptosis ? cell cycle ? protein transport ? ATPase activity ? ER-associated protein catabolism ? | 0  BLAST |
| PFL2060c ? rabGDI protein  Rab GDP-dissociation inhibitor activity ? Rab GTPase activator activity ? protein transport (IEA) ? vesicle-mediated transport ? regulation of GTPase activity (IEA) ? | YER136W ? Rab GDP-dissociation inhibitor activity ? membrane fraction ? soluble fraction ? vesicle-mediated transport ? | 0  BLAST |
| PF13\_0282 ? proteasome subunit, putative  endopeptidase activity ? threonine endopeptidase activity (IEA) ? proteasome core complex (sensu Eukaryota) ? ubiquitin-dependent protein catabolism ? | YGR135W ? endopeptidase activity ? ubiquitin-dependent protein catabolism ? proteasome core complex, alpha-subunit complex (sensu Eukaryota) ? filamentous growth ? | 0  BLAST |
| PFL2345c ? tat-binding protein homolog  nucleotide binding (IEA) ? proteasome complex (sensu Eukaryota) ? transcription cofactor activity ? ATP binding (IEA) ? nucleus (IEA) ? cytoplasm (IEA) ? ubiquitin-dependent protein catabolism ? hydrolase activity (IEA) ? ATPase activity ? nucleoside-triphosphatase activity (IEA) ? nucleotide kinase activity (IEA) ? protein catabolism (IEA) ? | YGL048C ? endopeptidase activity ? nucleus ? ubiquitin-dependent protein catabolism ? proteasome regulatory particle, base subcomplex (sensu Eukaryota) ? ATPase activity ? | 0  BLAST |
| PFE0915c ? proteasome subunit beta type 1  endopeptidase activity ? threonine endopeptidase activity (IEA) ? proteasome core complex (sensu Eukaryota) ? ubiquitin-dependent protein catabolism ? | YBL041W ? endopeptidase activity ? ubiquitin-dependent protein catabolism ? proteasome core complex, beta-subunit complex (sensu Eukaryota) ? | 9e-35  BLAST |
| PF07\_0112 ? proteasome subunit alpha type 5, putative  endopeptidase activity ? threonine endopeptidase activity (IEA) ? proteasome core complex (sensu Eukaryota) (IEA) ? ubiquitin-dependent protein catabolism ? proteasome core complex, alpha-subunit complex (sensu Eukaryota) ? | YGR253C ? endopeptidase activity ? ubiquitin-dependent protein catabolism ? response to stress ? proteasome core complex, alpha-subunit complex (sensu Eukaryota) ? sporulation (sensu Fungi) ? | 0  BLAST |
| PF14\_0716 ? Proteosome subunit alpha type 1, putative  endopeptidase activity ? threonine endopeptidase activity (IEA) ? proteasome core complex (sensu Eukaryota) ? ubiquitin-dependent protein catabolism ? | YMR314W ? endopeptidase activity ? ubiquitin-dependent protein catabolism ? proteasome core complex, alpha-subunit complex (sensu Eukaryota) ? | 3.00018e-42  BLAST |
| PF13\_0033 ? 26S proteasome regulatory subunit, putative  nucleotide binding (IEA) ? nucleic acid binding (IEA) ? endopeptidase activity ? ATP binding (IEA) ? nucleus (IEA) ? cytoplasm (IEA) ? proteasome regulatory particle (sensu Eukaryota) ? ubiquitin-dependent protein catabolism ? hydrolase activity (IEA) ? ATPase activity (IEA) ? nucleoside-triphosphatase activity (IEA) ? protein catabolism (IEA) ? | YOR259C ? endopeptidase activity ? nucleus ? ubiquitin-dependent protein catabolism ? proteasome regulatory particle, base subcomplex (sensu Eukaryota) ? ATPase activity ? | 0  BLAST |
| MAL13P1.343 ? proteasome regulatory subunit, putative  proteasome regulatory particle (sensu Eukaryota) ? ubiquitin-dependent protein catabolism ? | YFR004W ? endopeptidase activity ? nucleus ? ubiquitin-dependent protein catabolism ? proteasome regulatory particle, lid subcomplex (sensu Eukaryota) ? | 0  BLAST |
| MAL8P1.142 ? proteasome beta-subunit  endopeptidase activity ? threonine endopeptidase activity (IEA) ? proteasome core complex (sensu Eukaryota) ? ubiquitin-dependent protein catabolism (IEA) ? | YFR050C ? endopeptidase activity ? ubiquitin-dependent protein catabolism ? proteasome core complex, beta-subunit complex (sensu Eukaryota) ? | 5e-26  BLAST |
| PFF0420c ? proteasome subunit alpha type 2, putative  endopeptidase activity ? threonine endopeptidase activity (IEA) ? proteasome core complex (sensu Eukaryota) ? ubiquitin-dependent protein catabolism ? | YML092C ? endopeptidase activity ? ubiquitin-dependent protein catabolism ? proteasome core complex, alpha-subunit complex (sensu Eukaryota) ? | 0  BLAST |
| PF13\_0063 ? 26S proteasome regulatory subunit 7, putative  nucleotide binding (IEA) ? endopeptidase activity ? ATP binding (IEA) ? nucleus (IEA) ? cytoplasm (IEA) ? proteasome regulatory particle (sensu Eukaryota) ? ubiquitin-dependent protein catabolism ? hydrolase activity (IEA) ? ATPase activity (IEA) ? nucleoside-triphosphatase activity (IEA) ? protein catabolism (IEA) ? | YKL145W ? endopeptidase activity ? ubiquitin-dependent protein catabolism ? proteasome regulatory particle, base subcomplex (sensu Eukaryota) ? ATPase activity ? | 0  BLAST |
| PF14\_0025 ? proteosome subunit, putative  endopeptidase activity ? proteasome regulatory particle (sensu Eukaryota) ? proteolysis and peptidolysis ? | YDL097C ? structural molecule activity ? ubiquitin-dependent protein catabolism ? proteasome regulatory particle, lid subcomplex (sensu Eukaryota) ? | 8e-21  BLAST |
| PF13\_0156 ? proteasome subunit beta type 7 precursor, putative  endopeptidase activity ? threonine endopeptidase activity (IEA) ? proteasome core complex (sensu Eukaryota) ? ubiquitin-dependent protein catabolism ? | YOR157C ? endopeptidase activity ? ubiquitin-dependent protein catabolism ? proteasome core complex, beta-subunit complex (sensu Eukaryota) ? | 0  BLAST |
| PFC0745c ? proteasome component C8, putative  endopeptidase activity ? threonine endopeptidase activity (IEA) ? proteasome core complex (sensu Eukaryota) ? ubiquitin-dependent protein catabolism ? | YOR362C ? endopeptidase activity ? ubiquitin-dependent protein catabolism ? proteasome core complex, alpha-subunit complex (sensu Eukaryota) ? | 1e-32  BLAST |
| PFD0665c ? 26s proteasome aaa-ATPase subunit Rpt3, putative  nucleotide binding (IEA) ? ATP binding (IEA) ? nucleus (IEA) ? cytoplasm (IEA) ? proteasome regulatory particle (sensu Eukaryota) ? ubiquitin-dependent protein catabolism ? hydrolase activity (IEA) ? ATPase activity ? nucleoside-triphosphatase activity (IEA) ? protein catabolism (IEA) ? | YDR394W ? endopeptidase activity ? ubiquitin-dependent protein catabolism ? proteasome regulatory particle, base subcomplex (sensu Eukaryota) ? ATPase activity ? | 0  BLAST |
| MAL13P1.270 ? proteasome subunit, putative  endopeptidase activity ? threonine endopeptidase activity (IEA) ? proteasome core complex (sensu Eukaryota) ? ubiquitin-dependent protein catabolism ? | YOL038W ? endopeptidase activity ? mitochondrion ? cytosol ? ubiquitin-dependent protein catabolism ? proteasome core complex, alpha-subunit complex (sensu Eukaryota) ? | 0  BLAST |
| PFI1545c ? proteasome precursor, putative  threonine endopeptidase activity (IEA) ? proteasome core complex (sensu Eukaryota) (IEA) ? ubiquitin-dependent protein catabolism (IEA) ? | YJL001W ? endopeptidase activity ? ubiquitin-dependent protein catabolism ? response to stress ? proteasome core complex, beta-subunit complex (sensu Eukaryota) ? sporulation (sensu Fungi) ? | 2e-15  BLAST |
| PFA0400c ? beta3 proteasome subunit, putative  endopeptidase activity ? threonine endopeptidase activity (IEA) ? proteasome core complex (sensu Eukaryota) ? ubiquitin-dependent protein catabolism ? | YER094C ? endopeptidase activity ? ubiquitin-dependent protein catabolism ? proteasome core complex, beta-subunit complex (sensu Eukaryota) ? | 0  BLAST |
| PF11\_0314 ? 26S protease subunit regulatory subunit 6a, putative  nucleotide binding (IEA) ? proteasome complex (sensu Eukaryota) ? endopeptidase activity ? ATP binding (IEA) ? nucleus (IEA) ? cytoplasm (IEA) ? proteolysis and peptidolysis ? hydrolase activity (IEA) ? ATPase activity ? nucleoside-triphosphatase activity (IEA) ? protein catabolism (IEA) ? | YOR117W ? endopeptidase activity ? ubiquitin-dependent protein catabolism ? proteasome regulatory particle, base subcomplex (sensu Eukaryota) ? ATPase activity ? | 0  BLAST |
| PF10\_0111 ? 20S proteasome beta subunit, putative  endopeptidase activity ? threonine endopeptidase activity (IEA) ? proteasome core complex (sensu Eukaryota) ? ubiquitin-dependent protein catabolism ? | YPR103W ? endopeptidase activity ? ubiquitin-dependent protein catabolism ? proteasome core complex, beta-subunit complex (sensu Eukaryota) ? | 0  BLAST |
| MAL13P1.190 ? proteasome regulatory component, putative  endopeptidase activity ? proteasome regulatory particle (sensu Eukaryota) ? ubiquitin-dependent protein catabolism ? | YER021W ? ubiquitin-dependent protein catabolism ? proteasome regulatory particle, lid subcomplex (sensu Eukaryota) ? | 0  BLAST |
| PFC0520w ? 26S proteasome regulatory subunit S14, putative  proteasome regulatory particle (sensu Eukaryota) ? ubiquitin-dependent protein catabolism ? | YFR052W ? endopeptidase activity ? ubiquitin-dependent protein catabolism ? proteasome regulatory particle, lid subcomplex (sensu Eukaryota) ? | 4e-14  BLAST |
| MAL8P1.128 ? proteasome subunit alpha, putative  endopeptidase activity ? threonine endopeptidase activity (IEA) ? proteasome core complex (sensu Eukaryota) ? ubiquitin-dependent protein catabolism ? | YGL011C ? endopeptidase activity ? mitochondrion ? ubiquitin-dependent protein catabolism ? proteasome core complex, alpha-subunit complex (sensu Eukaryota) ? | 1e-34  BLAST |
| PF11\_0303 ? 26S proteasome regulatory complex subunit, putative  proteasome complex (sensu Eukaryota) ? endopeptidase activity ? proteolysis and peptidolysis ? | YPR108W ? structural molecule activity ? ubiquitin-dependent protein catabolism ? proteasome regulatory particle, lid subcomplex (sensu Eukaryota) ? | 0  BLAST |
| PF10\_0174 ? 26s proteasome subunit p55, putative  endopeptidase activity ? proteasome regulatory particle (sensu Eukaryota) ? ubiquitin-dependent protein catabolism ? | YDL147W ? ubiquitin-dependent protein catabolism ? proteasome regulatory particle, lid subcomplex (sensu Eukaryota) ? | 0  BLAST |
| PF14\_0178 ? hypothetical protein  ubiquitin-dependent protein catabolism (IEA) ? membrane ? | YGR048W ? protein binding ? endoplasmic reticulum ? mRNA processing ? ubiquitin-dependent protein catabolism ? protein transport ? | 7e-34  BLAST |

## Cluster Pair #4: 11 gene pairs.

| P.falciparum | S.cerevisiae | Blast evalue |
| --- | --- | --- |
| PF14\_0620 ? hypothetical protein | YKR079C ? nucleus ? cytoplasm ? mitochondrion ? purine nucleotide binding ? removal of tRNA 3'-trailer sequence ? 3'-tRNA processing endoribonuclease activity ? | 9e-23  BLAST |
| PFF0100w ? putative ATP dependent RNA helicase  nucleic acid binding (IEA) ? ATP-dependent RNA helicase activity ? helicase activity (IEA) ? ATP binding (IEA) ? ATP-dependent helicase activity (IEA) ? | YJL050W ? ATP-dependent RNA helicase activity ? nucleus ? nucleolus ? 35S primary transcript processing ? mRNA-nucleus export ? rRNA catabolism ? tRNA catabolism ? TRAMP complex ? ribosome biogenesis and assembly ? | 0  BLAST |
| PFF0625w ? nucleolar GTP-binding protein 1, putative  GTP binding ? | YPL093W ? ribosome-nucleus export ? GTP binding ? nucleolus ? ribosome biogenesis and assembly ? ribosomal large subunit biogenesis ? | 0  BLAST |
| MAL13P1.243 ? elongation factor Tu, putative  translation elongation factor activity ? GTP binding ? protein biosynthesis (IEA) ? translational elongation ? | YNL163C ? GTPase activity ? cytoplasm ? processing of 27S pre-rRNA ? ribosomal large subunit biogenesis ? | 5e-39  BLAST |
| PF10\_0128 ? hypothetical protein | YLR222C ? small nucleolar ribonucleoprotein complex ? processing of 20S pre-rRNA ? snoRNA binding ? | 5e-16  BLAST |
| PFE1310c ? hypothetical protein  \*\* also with: YCR057C, clust.pair #4 \*\* also with: YPR169W, clust.pair #5 | YLR129W ? small nucleolar ribonucleoprotein complex ? processing of 20S pre-rRNA ? snoRNA binding ? ribosome biogenesis and assembly ? | 0.0006  BLAST |
| PFE1310c ? hypothetical protein  \*\* also with: YLR129W, clust.pair #4 \*\* also with: YPR169W, clust.pair #5 | YCR057C ? cytokinesis ? small nucleolar ribonucleoprotein complex ? cytoplasm ? 35S primary transcript processing ? establishment of cell polarity (sensu Fungi) ? processing of 20S pre-rRNA ? snoRNA binding ? 90S preribosome ? ribosome biogenesis and assembly ? | 0.001  BLAST |
| PF11\_0116 ? hypothetical protein | YBL024W ? nucleus ? tRNA (cytosine-5-)-methyltransferase activity ? tRNA methylation ? ribosome biogenesis and assembly ? | 2e-09  BLAST |
| PFL1820w ? hypothetical protein | YLR196W ? nucleus ? nucleolus ? cytoplasm ? rRNA processing ? | 3e-15  BLAST |
| PFL1230w ? hypothetical protein | YLR186W ? nucleus ? nucleolus ? small nucleolar ribonucleoprotein complex ? cytoplasm ? nuclear microtubule ? 35S primary transcript processing ? ribosome biogenesis and assembly ? ribosomal small subunit biogenesis ? | 3e-12  BLAST |
| PF14\_0194 ? spliceosome-associated protein, putative  nucleic acid binding (IEA) ? spliceosome complex ? RNA splicing ? RNA splicing factor activity, transesterification mechanism ? | YER165W ? nucleus ? cytoplasm ? ribosome ? regulation of translational initiation ? poly(A) binding ? | 2e-19  BLAST |

## Cluster Pair #5: 7 gene pairs.

| P.falciparum | S.cerevisiae | Blast evalue |
| --- | --- | --- |
| PFB0175c ? hypothetical protein | YAL025C ? nucleolus ? processing of 27S pre-rRNA ? ribosome biogenesis and assembly ? ribosomal large subunit biogenesis ? | 1e-25  BLAST |
| MAL8P1.67 ? hypothetical protein, conserved | YDR339C ? nucleolus ? 35S primary transcript processing ? mitochondrion organization and biogenesis ? | 0  BLAST |
| MAL13P1.170 ? hypothetical protein  nucleic acid binding (IEA) ? nucleotidyltransferase activity (IEA) ? | YNL299W ? DNA-directed DNA polymerase activity ? polynucleotide adenylyltransferase activity ? nucleus ? nucleolus ? mitotic sister chromatid cohesion ? rRNA catabolism ? snRNA catabolism ? snoRNA catabolism ? | 5e-19  BLAST |
| PF13\_0177 ? ATP-dependent RNA helicase, putative  nucleic acid binding (IEA) ? helicase activity (IEA) ? ATP binding (IEA) ? ATP-dependent helicase activity (IEA) ? \*\* also with: YHR065C, clust.pair #0 | YHR169W ? ATP-dependent RNA helicase activity ? nucleolus ? 35S primary transcript processing ? ribosome biogenesis and assembly ? | 0  BLAST |
| PFE1310c ? hypothetical protein  \*\* also with: YLR129W, clust.pair #4 \*\* also with: YCR057C, clust.pair #4 | YPR169W ? nucleus ? nucleolus ? protein monoubiquitination ? | 0.0001  BLAST |
| PF11\_0259 ? hypothetical protein  nucleus (IEA) ? ribosome biogenesis and assembly (IEA) ? | YOR294W ? nucleus ? rRNA processing ? ribosome biogenesis and assembly ? | 2e-06  BLAST |
| MAL13P1.334 ? hypothetical protein | YJR002W ? nucleus ? nucleolus ? small nucleolar ribonucleoprotein complex ? 35S primary transcript processing ? processing of 20S pre-rRNA ? ribosome biogenesis and assembly ? | 0.082  BLAST |

## Cluster Pair #6: 39 gene pairs.

| P.falciparum | S.cerevisiae | Blast evalue |
| --- | --- | --- |
| PF07\_0067 ? hypothetical protein | YGR245C ? nucleus ? traversing start control point of mitotic cell cycle ? actin cytoskeleton organization and biogenesis ? ribosome biogenesis and assembly ? ribosome assembly ? | 1e-09  BLAST |
| PF10\_0123 ? GMP synthetase  catalytic activity (IEA) ? GMP synthase activity ? GMP synthase (glutamine-hydrolyzing) activity (IEA) ? ATP binding (IEA) ? purine nucleotide biosynthesis ? GMP biosynthesis (IEA) ? glutamine metabolism (IEA) ? | YMR217W ? GMP synthase (glutamine-hydrolyzing) activity ? GMP metabolism ? | 0  BLAST |
| PFL2475w ? DEAD%2FDEAH box helicase, putative  nucleic acid binding (IEA) ? ATP-dependent RNA helicase activity ? helicase activity (IEA) ? ATP binding (IEA) ? ATP-dependent helicase activity (IEA) ? RNA metabolism ? | YLL008W ? ribosomal large subunit assembly and maintenance ? ATP-dependent RNA helicase activity ? nucleolus ? 35S primary transcript processing ? ribosome biogenesis and assembly ? | 0  BLAST |
| PFL1170w ? polyadenylate-binding protein, putative  nucleic acid binding (IEA) ? RNA binding (IEA) ? mRNA binding ? RNA processing ? poly(A) binding ? | YER165W ? nucleus ? cytoplasm ? ribosome ? regulation of translational initiation ? poly(A) binding ? | 0  BLAST |
| PF14\_0068 ? fibrillarin, putative  RNA binding (IEA) ? nucleus (IEA) ? small nucleolar ribonucleoprotein complex ? mitochondrion ? rRNA processing ? | YDL014W ? ribosomal large subunit assembly and maintenance ? rRNA modification ? RNA methylation ? nucleolus ? small nucleolar ribonucleoprotein complex ? ribosome ? 35S primary transcript processing ? methyltransferase activity ? processing of 20S pre-rRNA ? snoRNA 3'-end processing ? ribosome biogenesis and assembly ? | 0  BLAST |
| PF14\_0550 ? hypothetical protein | YDR060W ? ribosomal large subunit assembly and maintenance ? Noc1p-Noc2p complex ? ribosome biogenesis and assembly ? | 0.011  BLAST |
| PFL0330c ? DNA-directed RNA polymerase III subunit, putative  DNA binding (IEA) ? DNA-directed RNA polymerase activity ? DNA-directed RNA polymerase III complex ? transcription (IEA) ? transcription from RNA polymerase III promoter ? | YOR207C ? DNA-directed RNA polymerase activity ? DNA-directed RNA polymerase III complex ? transcription from RNA polymerase III promoter ? | 0  BLAST |
| MAL7P1.24 ? hypothetical protein, conserved  intracellular (IEA) ? | YER126C ? nucleus ? ribosome biogenesis and assembly ? ribosomal large subunit biogenesis ? | 0  BLAST |
| PF13\_0178 ? translation initiation factor 6, putative  translation initiation factor activity (IEA) ? translational initiation (IEA) ? | YPR016C ? nucleus ? cytoplasm ? processing of 27S pre-rRNA ? ribosomal large subunit biogenesis ? ribosomal large subunit binding ? | 0  BLAST |
| PF14\_0100 ? cytidine triphosphate synthetase  catalytic activity (IEA) ? CTP synthase activity ? pyrimidine base metabolism ? pyrimidine nucleotide biosynthesis (IEA) ? | YBL039C ? CTP synthase activity ? cytosol ? CTP biosynthesis ? phospholipid biosynthesis ? pyrimidine base biosynthesis ? | 0  BLAST |
| PFF1030w ? hypothetical protein, conserved  RNA binding (IEA) ? | YDR496C ? mRNA binding ? nucleus ? nucleolus ? regulation of transcription, mating-type specific ? specific transcriptional repressor activity ? ribosome biogenesis and assembly ? | 0.0006  BLAST |
| PF14\_0456 ? hypothetical protein, conserved | YLR129W ? small nucleolar ribonucleoprotein complex ? processing of 20S pre-rRNA ? snoRNA binding ? ribosome biogenesis and assembly ? | 5.04467e-44  BLAST |
| PF13\_0309 ? hypothetical protein | YLR409C ? nucleus ? nucleolus ? small nucleolar ribonucleoprotein complex ? 35S primary transcript processing ? snoRNA binding ? ribosome biogenesis and assembly ? | 1e-17  BLAST |
| PFL1345c ? hypothetical protein, conserved  catalytic activity (IEA) ? iron ion binding (IEA) ? membrane ? | YPL086C ? histone acetyltransferase activity ? nucleus ? cytoplasm ? regulation of transcription from RNA polymerase II promoter ? tRNA modification ? transcription elongation factor complex ? | 0  BLAST |
| PFE1435c ? hypothetical protein | YER006W ? GTPase activity ? nucleus ? nucleolus ? rRNA processing ? ribosome biogenesis and assembly ? | 4e-09  BLAST |
| PF08\_0065 ? hypothetical protein, conserved | YMR131C ? nucleolus ? ribosome biogenesis and assembly ? | 5e-33  BLAST |
| PFB0375w ? hypothetical protein  metalloendopeptidase activity (IEA) ? proteolysis and peptidolysis (IEA) ? pathogenesis (IEA) ? membrane ? metal ion binding (IEA) ? | YDL167C ? cytoplasm ? ribosome biogenesis and assembly ? | 0.0004  BLAST |
| PFI1235w ? hypothetical protein | YDR083W ? telomere maintenance ? nucleolus ? rRNA processing ? methyltransferase activity ? ribosome biogenesis and assembly ? | 6e-24  BLAST |
| PF13\_0286 ? methyltransferase, putative  rRNA processing ? RNA methyltransferase activity ? | YCL054W ? nucleus ? nucleolus ? rRNA (uridine-2'-O-)-methyltransferase activity ? rRNA (guanine) methyltransferase activity ? processing of 27S pre-rRNA ? rRNA methylation ? | 0  BLAST |
| PFI0865w ? hypothetical protein, conserved  ATP binding (IEA) ? | YLR243W ? signal sequence binding ? | 1e-34  BLAST |
| PF14\_0292 ? hypothetical protein, conserved  GTP binding (IEA) ? | YGL099W ? ribosome-nucleus export ? conjugation with cellular fusion ? GTPase activity ? cytoplasm ? sporulation (sensu Fungi) ? ribosome biogenesis and assembly ? | 2e-30  BLAST |
| PF11\_0275 ? hypothetical protein | YLR409C ? nucleus ? nucleolus ? small nucleolar ribonucleoprotein complex ? 35S primary transcript processing ? snoRNA binding ? ribosome biogenesis and assembly ? | 0.083  BLAST |
| PFE1390w ? RNA helicase-1  nucleic acid binding (IEA) ? ATP-dependent RNA helicase activity ? helicase activity (IEA) ? ATP binding (IEA) ? ATP-dependent helicase activity (IEA) ? | YNL112W ? mRNA catabolism, nonsense-mediated decay ? RNA helicase activity ? nucleus ? cytoplasm ? mitochondrion ? 35S primary transcript processing ? | 0  BLAST |
| PF14\_0494 ? hypothetical protein, conserved  mitochondrion ? | YDL060W ? nucleolus ? cytoplasm ? rRNA processing ? ribosome biogenesis and assembly ? ribonucleoprotein binding ? | 2e-18  BLAST |
| PFL1175w ? hypothetical protein | YJL069C ? nucleolus ? small nucleolar ribonucleoprotein complex ? 35S primary transcript processing ? ribosome biogenesis and assembly ? | 7e-05  BLAST |
| PF10\_0197 ? hypothetical protein  apicoplast ? | YNL061W ? nucleolus ? rRNA processing ? RNA methyltransferase activity ? S-adenosylmethionine-dependent methyltransferase activity ? ribosome biogenesis and assembly ? | 1e-06  BLAST |
| PF14\_0174 ? hypothetical protein, conserved  RNA binding (IEA) ? pseudouridylate synthase activity (IEA) ? RNA processing (IEA) ? | YLR175W ? pseudouridylate synthase activity ? nucleolus ? 35S primary transcript processing ? rRNA pseudouridine synthesis ? box H/ACA snoRNP complex ? ribosome biogenesis and assembly ? | 0  BLAST |
| MAL13P1.341 ? hypothetical protein, conserved | YKL009W ? telomere maintenance ? nucleus ? nucleolus ? rRNA processing ? mRNA catabolism ? ribosome biogenesis and assembly ? ribosomal large subunit biogenesis ? | 4e-06  BLAST |
| MAL7P1.113 ? DEAD box helicase, putative  nucleic acid binding (IEA) ? RNA binding ? ATP-dependent RNA helicase activity ? helicase activity (IEA) ? ATP binding ? ATP-dependent helicase activity (IEA) ? RNA metabolism ? | YMR290C ? RNA binding ? ATP-dependent RNA helicase activity ? nuclear membrane ? nucleolus ? rRNA processing ? RNA-dependent ATPase activity ? ribosome biogenesis and assembly ? | 1e-24  BLAST |
| PF10\_0085 ? nucleolar protein NOP5, putative  RNA binding ? rRNA processing ? | YOR310C ? rRNA modification ? small nucleolar ribonucleoprotein complex ? 35S primary transcript processing ? processing of 20S pre-rRNA ? box C/D snoRNP complex ? ribosome biogenesis and assembly ? | 0  BLAST |
| PF07\_0122 ? hypothetical protein, conserved | YOL077C ? ribosomal large subunit assembly and maintenance ? nucleolus ? 5S rRNA binding ? rRNA primary transcript binding ? ribosome biogenesis and assembly ? | 1e-35  BLAST |
| PFB0370c ? RNA-binding protein, putative | YCL059C ? nucleolus ? small nucleolar ribonucleoprotein complex ? rRNA processing ? 35S primary transcript processing ? ribosome biogenesis and assembly ? | 0  BLAST |
| MAL13P1.14 ? ATP-dependent DEAD box helicase, putative  nucleic acid binding (IEA) ? ATP-dependent RNA helicase activity ? helicase activity (IEA) ? ATP binding (IEA) ? ATP-dependent helicase activity (IEA) ? | YMR128W ? RNA helicase activity ? nucleolus ? small nucleolar ribonucleoprotein complex ? mitochondrion ? processing of 20S pre-rRNA ? ribosome biogenesis and assembly ? | 9.94922e-44  BLAST |
| PF14\_0150 ? RNA polymerase small subunit, putative  DNA binding (IEA) ? DNA-directed RNA polymerase activity ? transcription ? protein dimerization activity (IEA) ? | YNL113W ? DNA-directed RNA polymerase activity ? DNA-directed RNA polymerase III complex ? DNA-directed RNA polymerase I complex ? transcription from RNA polymerase I promoter ? transcription from RNA polymerase III promoter ? ribosome biogenesis and assembly ? | 1e-18  BLAST |
| PF13\_0051 ? snornp protein gar1 homologue, putative  small nucleolar ribonucleoprotein complex (IEA) ? rRNA processing (IEA) ? RNA modification ? rRNA binding (IEA) ? ribosome biogenesis and assembly (IEA) ? | YHR089C ? RNA binding ? nucleolus ? small nucleolar ribonucleoprotein complex ? 35S primary transcript processing ? box H/ACA snoRNP complex ? ribosome biogenesis and assembly ? | 8e-17  BLAST |
| PF13\_0013 ? PBS lyase HEAT-like repeat domain protein | YJR070C ? microtubule cytoskeleton organization and biogenesis ? protein binding ? nucleus ? cytoplasm ? deoxyhypusine monooxygenase activity ? ribosome biogenesis and assembly ? hypusine biosynthesis ? | 8e-15  BLAST |
| PFL2010c ? DEAD%2FDEAH box helicase, putative  nucleic acid binding (IEA) ? ATP-dependent RNA helicase activity ? helicase activity (IEA) ? ATP binding (IEA) ? ATP-dependent helicase activity (IEA) ? RNA metabolism ? | YLR276C ? ribosomal large subunit assembly and maintenance ? ATP-dependent RNA helicase activity ? nucleolus ? 35S primary transcript processing ? ribosome biogenesis and assembly ? | 3e-25  BLAST |
| PF14\_0156 ? dimethyladenosine transferase, putative  rRNA modification ? rRNA (adenine-N6,N6-)-dimethyltransferase activity (IEA) ? mitochondrion ? rRNA processing (IEA) ? rRNA methyltransferase activity ? S-adenosylmethionine-dependent methyltransferase activity (IEA) ? rRNA (adenine) methyltransferase activity (IEA) ? | YPL266W ? rRNA modification ? rRNA (adenine-N6,N6-)-dimethyltransferase activity ? nucleolus ? 35S primary transcript processing ? ribosome biogenesis and assembly ? | 0  BLAST |
| PF07\_0015 ? hypothetical protein  metalloendopeptidase activity (IEA) ? proteolysis and peptidolysis (IEA) ? pathogenesis (IEA) ? metal ion binding (IEA) ? | YBL024W ? nucleus ? tRNA (cytosine-5-)-methyltransferase activity ? tRNA methylation ? ribosome biogenesis and assembly ? | 3e-39  BLAST |

## Cluster Pair #7: 3 gene pairs.

| P.falciparum | S.cerevisiae | Blast evalue |
| --- | --- | --- |
| PFL0380c ? tRNA delta%282%29-isopentenylpyrophosphate transferase, putative  tRNA isopentenyltransferase activity ? ATP binding ? tRNA processing ? apicoplast ? | YOR274W ? tRNA isopentenyltransferase activity ? nucleus ? nucleolus ? mitochondrion ? cytosol ? tRNA modification ? | 6e-29  BLAST |
| PF14\_0416 ? hypothetical protein  ubiquitin ligase complex (IEA) ? nucleic acid binding (IEA) ? ubiquitin-protein ligase activity (IEA) ? zinc ion binding (IEA) ? protein ubiquitination (IEA) ? | YLR323C ? spliceosome complex ? | 9e-17  BLAST |
| PF07\_0011 ? hypothetical protein, conserved | YHR122W ? nucleus ? cytoplasm ? transcription ? | 1e-23  BLAST |

## Cluster Pair #8: 6 gene pairs.

| P.falciparum | S.cerevisiae | Blast evalue |
| --- | --- | --- |
| PF14\_0324 ? hypothetical protein, conserved | YOR027W ? cytoplasm ? protein folding ? chaperone activator activity ? chaperone inhibitor activity ? Hsp70 protein binding ? | 0  BLAST |
| PFL0740c ? 10 kd chaperonin, putative  ATP binding (IEA) ? protein folding (IEA) ? response to unfolded protein ? response to heat ? unfolded protein binding ? | YOR020C ? mitochondrion ? mitochondrial matrix ? protein folding ? unfolded protein binding ? | 4e-17  BLAST |
| PF11\_0351 ? heat shock protein hsp70 homologue  ATP binding (IEA) ? response to unfolded protein ? response to heat ? | YJR045C ? presequence translocase-associated import motor ? mitochondrion ? mitochondrial inner membrane ? protein folding ? protein transporter activity ? ATPase activity ? mitochondrial matrix protein import ? enzyme regulator activity ? protein refolding ? mitochondrial nucleoid ? | 0  BLAST |
| PF10\_0153 ? hsp60  protein binding (IEA) ? ATP binding (IEA) ? mitochondrion ? protein folding ? protein targeting to mitochondrion ? response to unfolded protein ? ATPase activity, coupled ? cellular protein metabolism (IEA) ? unfolded protein binding (IEA) ? | YLR259C ? single-stranded DNA binding ? mitochondrion ? protein folding ? mitochondrial matrix protein import ? mitochondrial nucleoid ? | 0  BLAST |
| PF07\_0029 ? heat shock protein 86  ATP binding (IEA) ? protein folding (IEA) ? response to unfolded protein ? response to heat ? unfolded protein binding (IEA) ? \*\* also with: YPL240C, clust.pair #8 | YMR186W ? telomere maintenance ? cytoplasm ? mitochondrion ? protein folding ? 'de novo' protein folding ? response to stress ? protein refolding ? ATPase activity, coupled ? proteasome assembly ? unfolded protein binding ? | 0  BLAST |
| PF07\_0029 ? heat shock protein 86  ATP binding (IEA) ? protein folding (IEA) ? response to unfolded protein ? response to heat ? unfolded protein binding (IEA) ? \*\* also with: YMR186W, clust.pair #8 | YPL240C ? cytoplasm ? 'de novo' protein folding ? response to stress ? response to osmotic stress ? protein refolding ? ATPase activity, coupled ? proteasome assembly ? unfolded protein binding ? | 0  BLAST |

## Cluster Pair #9: 8 gene pairs.

| P.falciparum | S.cerevisiae | Blast evalue |
| --- | --- | --- |
| PFF0160c ? dihydroorotate dehydrogenase, mitochondrial precursor  dihydroorotate dehydrogenase activity ? dihydroorotate oxidase activity (IEA) ? mitochondrial inner membrane ? 'de novo' pyrimidine base biosynthesis ? membrane (IEA) ? | YKL216W ? dihydroorotate dehydrogenase activity ? cytoplasm ? 'de novo' pyrimidine base biosynthesis ? extrinsic to membrane ? | 5e-08  BLAST |
| MAL13P1.73 ? hypothetical protein | YNL016W ? mRNA catabolism, nonsense-mediated decay ? nucleic acid binding ? mRNA binding ? nucleus ? cytoplasm ? heterogeneous nuclear ribonucleoprotein complex ? regulation of mRNA stability ? | 0.097  BLAST |
| PF11\_0071 ? RuvB DNA helicase, putative  nucleotide binding ? ATP binding (IEA) ? nucleus (IEA) ? DNA repair ? DNA recombination ? ATPase activity ? nucleoside-triphosphatase activity (IEA) ? ATP-dependent 5' to 3' DNA helicase activity (IEA) ? | YDR190C ? SWR1 complex ? nucleus ? chromatin remodeling ? regulation of transcription from RNA polymerase II promoter ? chromatin remodeling complex ? ATPase activity ? INO80 complex ? ATP-dependent 5' to 3' DNA helicase activity ? | 0  BLAST |
| PFE0630c ? orotate phosphoribosyltransferase, putative  orotate phosphoribosyltransferase activity ? nucleoside metabolism (IEA) ? | YML106W ? orotate phosphoribosyltransferase activity ? nucleus ? cytoplasm ? 'de novo' pyrimidine base biosynthesis ? | 3e-16  BLAST |
| PFC0465c ? hypothetical protein  mRNA processing (IEA) ? | YAL035W ? translation initiation factor activity ? GTPase activity ? mitochondrion ? cytosolic small ribosomal subunit (sensu Eukaryota) ? translational initiation ? | 0.026  BLAST |
| PFL0665c ? RNA polymerase subunit 8c, putative  nucleic acid binding (IEA) ? DNA-directed RNA polymerase activity ? transcription (IEA) ? transcription initiation ? | YOR224C ? DNA-directed RNA polymerase activity ? DNA-directed RNA polymerase II, core complex ? DNA-directed RNA polymerase III complex ? DNA-directed RNA polymerase I complex ? transcription from RNA polymerase I promoter ? transcription from RNA polymerase II promoter ? transcription from RNA polymerase III promoter ? | 1e-14  BLAST |
| PFE1360c ? methionine aminopeptidase, putative  methionyl aminopeptidase activity ? proteolysis and peptidolysis ? metalloexopeptidase activity (IEA) ? | YLR244C ? methionyl aminopeptidase activity ? cytosolic ribosome (sensu Eukaryota) ? proteolysis and peptidolysis ? | 0  BLAST |
| PFL0335c ? eukaryotic translation initiation factor 5, putative  translation initiation factor activity ? translational initiation (IEA) ? regulation of translational initiation ? | YPR041W ? translation initiation factor activity ? GTPase activator activity ? cytosolic small ribosomal subunit (sensu Eukaryota) ? regulation of translational initiation ? mature ribosome assembly ? | 6e-33  BLAST |

## Cluster Pair #10: 6 gene pairs.

| P.falciparum | S.cerevisiae | Blast evalue |
| --- | --- | --- |
| PFC0350c ? T-complex protein eta subunit, putative  protein binding (IEA) ? ATP binding (IEA) ? chaperonin-containing T-complex ? protein folding ? cellular protein metabolism (IEA) ? unfolded protein binding ? | YJL111W ? cytoplasm ? chaperonin-containing T-complex ? cytoskeleton ? protein folding ? cytoskeleton organization and biogenesis ? unfolded protein binding ? | 0  BLAST |
| PFL1425w ? t-complex protein 1, gamma subunit, putative  protein binding (IEA) ? ATP binding ? chaperonin-containing T-complex ? protein folding ? cellular protein metabolism (IEA) ? unfolded protein binding ? | YJL014W ? cytoplasm ? chaperonin-containing T-complex ? cytoskeleton ? protein folding ? cytoskeleton organization and biogenesis ? unfolded protein binding ? | 0  BLAST |
| PFI0965w ? conserved protein, putative | YBL036C ? intracellular ? amino acid metabolism ? alanine racemase activity ? pyridoxal phosphate binding ? | 3e-24  BLAST |
| PFC0900w ? T-complex protein 1 epsilon subunit, putative  protein binding (IEA) ? ATP binding (IEA) ? chaperonin-containing T-complex ? protein folding ? cellular protein metabolism (IEA) ? unfolded protein binding ? | YJR064W ? cytoplasm ? chaperonin-containing T-complex ? cytoskeleton ? protein folding ? cytoskeleton organization and biogenesis ? unfolded protein binding ? | 0  BLAST |
| PF14\_0335 ? hypothetical protein, conserved  membrane ? | YGL231C ? endoplasmic reticulum ? | 0.0009  BLAST |
| PFE1470w ? cell cycle regulator protein, putative  regulation of progression through cell cycle ? RNA binding ? | YER007C-A ? RNA binding ? cytoplasm ? ribosome ? ribosome biogenesis and assembly ? | 4.99983e-42  BLAST |

## Cluster Pair #11: 3 gene pairs.

| P.falciparum | S.cerevisiae | Blast evalue |
| --- | --- | --- |
| PFI0355c ? ATP-dependent heat shock protein, putative  nucleotide binding (IEA) ? protein binding (IEA) ? ATP binding (IEA) ? cytoplasm (IEA) ? HslUV protease complex (IEA) ? HslUV protease activity (IEA) ? ATPase activity (IEA) ? nucleoside-triphosphatase activity (IEA) ? | YBR227C ? ATP binding ? mitochondrion ? mitochondrial matrix ? unfolded protein binding ? | 4e-14  BLAST |
| MAL13P1.21 ? hypothetical protein | YBL097W ? mitotic sister chromatid segregation ? nuclear condensin complex ? nucleus ? mitotic chromosome condensation ? | 4e-07  BLAST |
| PF11\_0377 ? casein kinase 1  protein kinase activity (IEA) ? protein serine/threonine kinase activity (IEA) ? casein kinase I activity ? protein-tyrosine kinase activity (IEA) ? ATP binding (IEA) ? protein amino acid phosphorylation ? | YPL204W ? nuclear division ? casein kinase activity ? nucleus ? plasma membrane ? DNA repair ? chromosome segregation ? mitosis ? meiosis ? ribosomal small subunit biogenesis ? | 0  BLAST |

## Cluster Pair #12: 8 gene pairs.

| P.falciparum | S.cerevisiae | Blast evalue |
| --- | --- | --- |
| PFL0210c ? eukaryotic initiation factor 5a, putative  nucleic acid binding (IEA) ? translation initiation factor activity ? translational initiation ? \*\* also with: YJR047C, clust.pair #12 | YEL034W ? translation initiation factor activity ? protein binding ? cytoplasm ? mitochondrion ? ribosome ? translational initiation ? | 0  BLAST |
| PFL0210c ? eukaryotic initiation factor 5a, putative  nucleic acid binding (IEA) ? translation initiation factor activity ? translational initiation ? \*\* also with: YEL034W, clust.pair #12 | YJR047C ? translation initiation factor activity ? ribosome ? translational initiation ? | 1.96182e-44  BLAST |
| PF10\_0068 ? hypothetical protein  nucleic acid binding (IEA) ? | YCL011C ? telomere maintenance ? RNA binding ? nucleus ? poly(A)+ mRNA-nucleus export ? telomeric DNA binding ? | 1e-11  BLAST |
| PFI0645w ? EF-1B  translation elongation factor activity (IEA) ? eukaryotic translation elongation factor 1 complex (IEA) ? translational elongation (IEA) ? | YAL003W ? translation elongation factor activity ? ribosome ? eukaryotic translation elongation factor 1 complex ? translational elongation ? | 7e-06  BLAST |
| PFI0740c ? ubiquitin conjugating enzyme, putative  protein modification (IEA) ? ubiquitin cycle (IEA) ? small protein activating enzyme activity (IEA) ? | YDL064W ? mitotic spindle elongation ? G2/M transition of mitotic cell cycle ? nucleus ? protein sumoylation ? SUMO conjugating enzyme activity ? | 0  BLAST |
| PF14\_0411 ? small nuclear ribonuclear protein, putative  nucleus (IEA) ? small nucleolar ribonucleoprotein complex ? mRNA processing (IEA) ? RNA splicing ? ribonucleoprotein complex (IEA) ? | YER146W ? nuclear mRNA splicing, via spliceosome ? RNA binding ? snRNP U6 ? nucleolus ? small nucleolar ribonucleoprotein complex ? mRNA catabolism ? RNA splicing factor activity, transesterification mechanism ? U4/U6 x U5 tri-snRNP complex ? | 2e-08  BLAST |
| PFC0870w ? elongation factor 1 %28EF-1%29, putative  translation elongation factor activity ? eukaryotic translation elongation factor 1 complex ? translational elongation ? | YAL003W ? translation elongation factor activity ? ribosome ? eukaryotic translation elongation factor 1 complex ? translational elongation ? | 3e-15  BLAST |
| PF13\_0023 ? DNA-directed RNA polymerase 2, putative  DNA binding (IEA) ? DNA-directed RNA polymerase activity ? transcription (IEA) ? transcription from RNA polymerase II promoter ? DNA-directed RNA polymerase II, holoenzyme ? | YOL005C ? DNA-directed RNA polymerase activity ? DNA-directed RNA polymerase II, core complex ? transcription from RNA polymerase II promoter ? | 1e-12  BLAST |

## Cluster Pair #13: 15 gene pairs.

| P.falciparum | S.cerevisiae | Blast evalue |
| --- | --- | --- |
| PFE0975c ? 40S ribosomal subunit protein S24, putative  structural constituent of ribosome (IEA) ? intracellular (IEA) ? ribosome (IEA) ? protein biosynthesis (IEA) ? | YER074W ? structural constituent of ribosome ? mitochondrion ? cytosolic small ribosomal subunit (sensu Eukaryota) ? protein biosynthesis ? | 3e-33  BLAST |
| PF14\_0627 ? ribosomal protein S3, putative  nucleic acid binding (IEA) ? structural constituent of ribosome ? intracellular (IEA) ? ribosome (IEA) ? cytosolic small ribosomal subunit (sensu Eukaryota) ? protein biosynthesis ? small ribosomal subunit (IEA) ? | YNL178W ? structural constituent of ribosome ? cytosolic small ribosomal subunit (sensu Eukaryota) ? protein biosynthesis ? response to DNA damage stimulus ? nucleolar preribosome, small subunit precursor ? | 0  BLAST |
| PFL1470c ? hypothetical protein | YMR116C ? telomere maintenance ? cytoplasm ? cytosolic small ribosomal subunit (sensu Eukaryota) ? negative regulation of protein biosynthesis ? | 0.1  BLAST |
| PF11\_0447 ? translation initiation factor eIF-1A, putative  nucleic acid binding (IEA) ? RNA binding (IEA) ? translation initiation factor activity ? translational initiation ? eukaryotic 43S preinitiation complex ? | YMR260C ? translation initiation factor activity ? ribosome ? translational initiation ? | 2.00386e-43  BLAST |
| PF13\_0132 ? 60S ribosomal protein L23a, putative  structural constituent of ribosome ? cytosolic large ribosomal subunit (sensu Eukaryota) ? protein biosynthesis ? rRNA binding ? | YOL127W ? ribosomal large subunit assembly and maintenance ? RNA binding ? structural constituent of ribosome ? cytosolic large ribosomal subunit (sensu Eukaryota) ? protein biosynthesis ? | 2e-21  BLAST |
| PFC0300c ? 60S ribosomal protein L7, putative  structural constituent of ribosome ? intracellular (IEA) ? ribosome (IEA) ? cytosolic large ribosomal subunit (sensu Eukaryota) ? protein biosynthesis ? large ribosomal subunit (IEA) ? transcription regulator activity (IEA) ? \*\* also with: YPL198W, clust.pair #13 | YGL076C ? structural constituent of ribosome ? cytosolic large ribosomal subunit (sensu Eukaryota) ? protein biosynthesis ? | 1.00053e-42  BLAST |
| PFC0300c ? 60S ribosomal protein L7, putative  structural constituent of ribosome ? intracellular (IEA) ? ribosome (IEA) ? cytosolic large ribosomal subunit (sensu Eukaryota) ? protein biosynthesis ? large ribosomal subunit (IEA) ? transcription regulator activity (IEA) ? \*\* also with: YGL076C, clust.pair #13 | YPL198W ? structural constituent of ribosome ? cytosolic large ribosomal subunit (sensu Eukaryota) ? protein biosynthesis ? | 1.00053e-42  BLAST |
| PFD1055w ? ribosomal protein S19s, putative  structural constituent of ribosome ? intracellular (IEA) ? ribosome ? protein biosynthesis ? \*\* also with: YOL121C, clust.pair #13 | YNL302C ? ribosomal small subunit-nucleus export ? structural constituent of ribosome ? cytosolic small ribosomal subunit (sensu Eukaryota) ? rRNA processing ? protein biosynthesis ? ribosomal small subunit biogenesis ? | 3e-20  BLAST |
| PFD1055w ? ribosomal protein S19s, putative  structural constituent of ribosome ? intracellular (IEA) ? ribosome ? protein biosynthesis ? \*\* also with: YNL302C, clust.pair #13 | YOL121C ? ribosomal small subunit-nucleus export ? telomere maintenance ? structural constituent of ribosome ? cytosolic small ribosomal subunit (sensu Eukaryota) ? rRNA processing ? protein biosynthesis ? ribosomal small subunit biogenesis ? | 3e-20  BLAST |
| PFE1005w ? 40S ribosomal subunit protein S9, putative  RNA binding (IEA) ? structural constituent of ribosome (IEA) ? intracellular (IEA) ? ribosome (IEA) ? protein biosynthesis (IEA) ? small ribosomal subunit (IEA) ? \*\* also with: YPL081W, clust.pair #13 | YBR189W ? structural constituent of ribosome ? small nucleolar ribonucleoprotein complex ? cytosolic small ribosomal subunit (sensu Eukaryota) ? protein biosynthesis ? regulation of translational fidelity ? | 0  BLAST |
| PFE1005w ? 40S ribosomal subunit protein S9, putative  RNA binding (IEA) ? structural constituent of ribosome (IEA) ? intracellular (IEA) ? ribosome (IEA) ? protein biosynthesis (IEA) ? small ribosomal subunit (IEA) ? \*\* also with: YBR189W, clust.pair #13 | YPL081W ? structural constituent of ribosome ? small nucleolar ribonucleoprotein complex ? cytoplasm ? cytosolic small ribosomal subunit (sensu Eukaryota) ? protein biosynthesis ? regulation of translational fidelity ? | 0  BLAST |
| PFC0200w ? 60S Ribosomal protein L44, putative  structural constituent of ribosome ? intracellular (IEA) ? ribosome (IEA) ? cytosolic large ribosomal subunit (sensu Eukaryota) ? protein biosynthesis ? \*\* also with: YNL162W, clust.pair #13 | YHR141C ? structural constituent of ribosome ? cytosolic large ribosomal subunit (sensu Eukaryota) ? protein biosynthesis ? | 6e-30  BLAST |
| PFC0200w ? 60S Ribosomal protein L44, putative  structural constituent of ribosome ? intracellular (IEA) ? ribosome (IEA) ? cytosolic large ribosomal subunit (sensu Eukaryota) ? protein biosynthesis ? \*\* also with: YHR141C, clust.pair #13 | YNL162W ? structural constituent of ribosome ? cytosolic large ribosomal subunit (sensu Eukaryota) ? protein biosynthesis ? | 6e-30  BLAST |
| PF14\_0585 ? ribosomal protein S28e, putative  structural constituent of ribosome ? intracellular (IEA) ? ribosome (IEA) ? cytosolic small ribosomal subunit (sensu Eukaryota) ? protein biosynthesis ? \*\* also with: YOR167C, clust.pair #13 | YLR264W ? telomere maintenance ? structural constituent of ribosome ? cytosolic small ribosomal subunit (sensu Eukaryota) ? protein biosynthesis ? | 5e-12  BLAST |
| PF14\_0585 ? ribosomal protein S28e, putative  structural constituent of ribosome ? intracellular (IEA) ? ribosome (IEA) ? cytosolic small ribosomal subunit (sensu Eukaryota) ? protein biosynthesis ? \*\* also with: YLR264W, clust.pair #13 | YOR167C ? structural constituent of ribosome ? cytosolic small ribosomal subunit (sensu Eukaryota) ? protein biosynthesis ? | 5e-12  BLAST |

## Cluster Pair #14: 74 gene pairs.

| P.falciparum | S.cerevisiae | Blast evalue |
| --- | --- | --- |
| PF08\_0019 ? guanine nucleotide-binding protein, putative  protein kinase C binding ? heterotrimeric G-protein complex ? G-protein coupled receptor protein signaling pathway ? | YMR116C ? telomere maintenance ? cytoplasm ? cytosolic small ribosomal subunit (sensu Eukaryota) ? negative regulation of protein biosynthesis ? | 0  BLAST |
| PF10\_0264 ? 40S ribosomal protein, putative  structural constituent of ribosome ? intracellular (IEA) ? ribosome (IEA) ? cytosolic small ribosomal subunit (sensu Eukaryota) ? protein biosynthesis ? small ribosomal subunit (IEA) ? \*\* also with: YLR048W, clust.pair #14 | YGR214W ? ribosomal small subunit assembly and maintenance ? structural constituent of ribosome ? cytosolic small ribosomal subunit (sensu Eukaryota) ? protein biosynthesis ? | 0  BLAST |
| PF10\_0264 ? 40S ribosomal protein, putative  structural constituent of ribosome ? intracellular (IEA) ? ribosome (IEA) ? cytosolic small ribosomal subunit (sensu Eukaryota) ? protein biosynthesis ? small ribosomal subunit (IEA) ? \*\* also with: YGR214W, clust.pair #14 | YLR048W ? ribosomal small subunit assembly and maintenance ? structural constituent of ribosome ? cytosolic small ribosomal subunit (sensu Eukaryota) ? protein biosynthesis ? | 0  BLAST |
| PF11\_0313 ? ribosomal phosphoprotein P0  structural constituent of ribosome ? intracellular (IEA) ? mitochondrion ? ribosome (IEA) ? cytosolic large ribosomal subunit (sensu Eukaryota) ? protein biosynthesis ? translational elongation (IEA) ? ribosome biogenesis and assembly (IEA) ? | YLR340W ? ribosomal large subunit assembly and maintenance ? structural constituent of ribosome ? cytosolic large ribosomal subunit (sensu Eukaryota) ? protein biosynthesis ? translational elongation ? | 0  BLAST |
| PF14\_0240 ? ribosomal protein L21e, putative  structural constituent of ribosome ? mitochondrion ? cytosolic large ribosomal subunit (sensu Eukaryota) ? protein biosynthesis ? \*\* also with: YPL079W, clust.pair #14 | YBR191W ? structural constituent of ribosome ? cytosolic large ribosomal subunit (sensu Eukaryota) ? protein biosynthesis ? | 4e-28  BLAST |
| PF14\_0240 ? ribosomal protein L21e, putative  structural constituent of ribosome ? mitochondrion ? cytosolic large ribosomal subunit (sensu Eukaryota) ? protein biosynthesis ? \*\* also with: YBR191W, clust.pair #14 | YPL079W ? structural constituent of ribosome ? cytosolic large ribosomal subunit (sensu Eukaryota) ? protein biosynthesis ? | 5e-28  BLAST |
| MAL13P1.209 ? 60S ribosomal subunit porotein L18, putative  structural constituent of ribosome ? intracellular (IEA) ? ribosome (IEA) ? cytosolic large ribosomal subunit (sensu Eukaryota) ? protein biosynthesis ? \*\* also with: YOL120C, clust.pair #14 | YNL301C ? structural constituent of ribosome ? cytosolic large ribosomal subunit (sensu Eukaryota) ? protein biosynthesis ? | 8.00001e-42  BLAST |
| MAL13P1.209 ? 60S ribosomal subunit porotein L18, putative  structural constituent of ribosome ? intracellular (IEA) ? ribosome (IEA) ? cytosolic large ribosomal subunit (sensu Eukaryota) ? protein biosynthesis ? \*\* also with: YNL301C, clust.pair #14 | YOL120C ? structural constituent of ribosome ? cytosolic large ribosomal subunit (sensu Eukaryota) ? protein biosynthesis ? | 8.00001e-42  BLAST |
| PF14\_0141 ? ribosomal protein L10, putative  structural constituent of ribosome ? intracellular (IEA) ? ribosome (IEA) ? cytosolic large ribosomal subunit (sensu Eukaryota) ? protein biosynthesis ? | YLR075W ? ribosomal large subunit assembly and maintenance ? structural constituent of ribosome ? cytosolic large ribosomal subunit (sensu Eukaryota) ? protein biosynthesis ? | 0  BLAST |
| PF11\_0065 ? ribosomal protein S4, putative  RNA binding ? structural constituent of ribosome ? intracellular (IEA) ? mitochondrion ? ribosome (IEA) ? protein biosynthesis ? small ribosomal subunit ? \*\* also with: YJR145C, clust.pair #14 | YHR203C ? telomere maintenance ? structural constituent of ribosome ? cytosolic small ribosomal subunit (sensu Eukaryota) ? protein biosynthesis ? | 0  BLAST |
| PF11\_0065 ? ribosomal protein S4, putative  RNA binding ? structural constituent of ribosome ? intracellular (IEA) ? mitochondrion ? ribosome (IEA) ? protein biosynthesis ? small ribosomal subunit ? \*\* also with: YHR203C, clust.pair #14 | YJR145C ? telomere maintenance ? structural constituent of ribosome ? cytoplasm ? cytosolic small ribosomal subunit (sensu Eukaryota) ? protein biosynthesis ? processing of 20S pre-rRNA ? | 0  BLAST |
| PF10\_0272 ? ribosomal protein L3, putative  structural constituent of ribosome ? intracellular (IEA) ? mitochondrion ? ribosome (IEA) ? cytosolic large ribosomal subunit (sensu Eukaryota) ? protein biosynthesis ? | YOR063W ? ribosomal large subunit assembly and maintenance ? structural constituent of ribosome ? cytosolic large ribosomal subunit (sensu Eukaryota) ? protein biosynthesis ? | 0  BLAST |
| PF14\_0083 ? ribosomal protein S8e, putative  structural constituent of ribosome ? intracellular (IEA) ? mitochondrion ? cytosolic small ribosomal subunit (sensu Eukaryota) ? protein biosynthesis ? \*\* also with: YER102W, clust.pair #14 | YBL072C ? structural constituent of ribosome ? cytosolic small ribosomal subunit (sensu Eukaryota) ? protein biosynthesis ? | 0  BLAST |
| PF14\_0083 ? ribosomal protein S8e, putative  structural constituent of ribosome ? intracellular (IEA) ? mitochondrion ? cytosolic small ribosomal subunit (sensu Eukaryota) ? protein biosynthesis ? \*\* also with: YBL072C, clust.pair #14 | YER102W ? structural constituent of ribosome ? cytosolic small ribosomal subunit (sensu Eukaryota) ? protein biosynthesis ? | 0  BLAST |
| PFB0885w ? 40S ribosomal protein S30, putative  structural constituent of ribosome ? intracellular (IEA) ? mitochondrion ? ribosome (IEA) ? cytosolic small ribosomal subunit (sensu Eukaryota) ? protein biosynthesis ? | YOR182C ? telomere maintenance ? structural constituent of ribosome ? cytosolic small ribosomal subunit (sensu Eukaryota) ? protein biosynthesis ? | 4e-13  BLAST |
| PF14\_0391 ? ribosomal protein L1, putative  structural constituent of ribosome ? intracellular (IEA) ? ribosome (IEA) ? cytosolic large ribosomal subunit (sensu Eukaryota) ? protein biosynthesis ? \*\* also with: YPL220W, clust.pair #14 | YGL135W ? telomere maintenance ? structural constituent of ribosome ? cytosolic large ribosomal subunit (sensu Eukaryota) ? protein biosynthesis ? | 0  BLAST |
| PF14\_0391 ? ribosomal protein L1, putative  structural constituent of ribosome ? intracellular (IEA) ? ribosome (IEA) ? cytosolic large ribosomal subunit (sensu Eukaryota) ? protein biosynthesis ? \*\* also with: YGL135W, clust.pair #14 | YPL220W ? structural constituent of ribosome ? cytosolic large ribosomal subunit (sensu Eukaryota) ? protein biosynthesis ? | 0  BLAST |
| PFE0350c ? 60S ribosomal subunit protein L4%2FL1, putative  RNA binding ? structural constituent of ribosome ? intracellular (IEA) ? ribosome (IEA) ? protein biosynthesis ? large ribosomal subunit ? \*\* also with: YDR012W, clust.pair #14 | YBR031W ? structural constituent of ribosome ? cytosolic large ribosomal subunit (sensu Eukaryota) ? protein biosynthesis ? | 0  BLAST |
| PFE0350c ? 60S ribosomal subunit protein L4%2FL1, putative  RNA binding ? structural constituent of ribosome ? intracellular (IEA) ? ribosome (IEA) ? protein biosynthesis ? large ribosomal subunit ? \*\* also with: YBR031W, clust.pair #14 | YDR012W ? structural constituent of ribosome ? cytoplasm ? cytosolic large ribosomal subunit (sensu Eukaryota) ? protein biosynthesis ? | 0  BLAST |
| PF13\_0268 ? ribosomal protein L17, putative  structural constituent of ribosome ? intracellular (IEA) ? ribosome (IEA) ? cytosolic large ribosomal subunit (sensu Eukaryota) ? protein biosynthesis ? large ribosomal subunit (IEA) ? \*\* also with: YKL180W, clust.pair #14 | YJL177W ? structural constituent of ribosome ? cytosolic large ribosomal subunit (sensu Eukaryota) ? protein biosynthesis ? | 3e-40  BLAST |
| PF13\_0268 ? ribosomal protein L17, putative  structural constituent of ribosome ? intracellular (IEA) ? ribosome (IEA) ? cytosolic large ribosomal subunit (sensu Eukaryota) ? protein biosynthesis ? large ribosomal subunit (IEA) ? \*\* also with: YJL177W, clust.pair #14 | YKL180W ? structural constituent of ribosome ? cytoplasm ? cytosolic large ribosomal subunit (sensu Eukaryota) ? protein biosynthesis ? | 4.00001e-40  BLAST |
| PFC1020c ? 40S ribosomal protein S3A, putative  structural constituent of ribosome ? intracellular (IEA) ? ribosome (IEA) ? cytosolic small ribosomal subunit (sensu Eukaryota) ? protein biosynthesis ? \*\* also with: YML063W, clust.pair #14 | YLR441C ? structural constituent of ribosome ? cytosolic small ribosomal subunit (sensu Eukaryota) ? protein biosynthesis ? | 0  BLAST |
| PFC1020c ? 40S ribosomal protein S3A, putative  structural constituent of ribosome ? intracellular (IEA) ? ribosome (IEA) ? cytosolic small ribosomal subunit (sensu Eukaryota) ? protein biosynthesis ? \*\* also with: YLR441C, clust.pair #14 | YML063W ? structural constituent of ribosome ? cytosolic small ribosomal subunit (sensu Eukaryota) ? protein biosynthesis ? | 0  BLAST |
| PF07\_0088 ? 40S ribosomal protein S5, putative  structural constituent of ribosome ? intracellular (IEA) ? ribosome (IEA) ? cytosolic small ribosomal subunit (sensu Eukaryota) ? protein biosynthesis ? small ribosomal subunit (IEA) ? | YJR123W ? structural constituent of ribosome ? cytosolic small ribosomal subunit (sensu Eukaryota) ? protein biosynthesis ? | 0  BLAST |
| PF14\_0579 ? ribosomal protein L27, putative  structural constituent of ribosome ? intracellular (IEA) ? ribosome (IEA) ? cytosolic large ribosomal subunit (sensu Eukaryota) ? protein biosynthesis ? \*\* also with: YDR471W, clust.pair #14 | YHR010W ? structural constituent of ribosome ? cytosolic large ribosomal subunit (sensu Eukaryota) ? protein biosynthesis ? | 1e-16  BLAST |
| PF14\_0579 ? ribosomal protein L27, putative  structural constituent of ribosome ? intracellular (IEA) ? ribosome (IEA) ? cytosolic large ribosomal subunit (sensu Eukaryota) ? protein biosynthesis ? \*\* also with: YHR010W, clust.pair #14 | YDR471W ? structural constituent of ribosome ? cytosolic large ribosomal subunit (sensu Eukaryota) ? protein biosynthesis ? | 5e-16  BLAST |
| PFC0290w ? 40S ribosomal protein S23, putative  nucleic acid binding (IEA) ? structural constituent of ribosome ? intracellular (IEA) ? ribosome (IEA) ? cytosolic small ribosomal subunit (sensu Eukaryota) ? protein biosynthesis ? small ribosomal subunit (IEA) ? \*\* also with: YPR132W, clust.pair #14 | YGR118W ? telomere maintenance ? structural constituent of ribosome ? cytosolic small ribosomal subunit (sensu Eukaryota) ? protein biosynthesis ? regulation of translational fidelity ? | 0  BLAST |
| PFC0290w ? 40S ribosomal protein S23, putative  nucleic acid binding (IEA) ? structural constituent of ribosome ? intracellular (IEA) ? ribosome (IEA) ? cytosolic small ribosomal subunit (sensu Eukaryota) ? protein biosynthesis ? small ribosomal subunit (IEA) ? \*\* also with: YGR118W, clust.pair #14 | YPR132W ? telomere maintenance ? structural constituent of ribosome ? cytosolic small ribosomal subunit (sensu Eukaryota) ? protein biosynthesis ? regulation of translational fidelity ? | 0  BLAST |
| PFD0770c ? ribosomal protein l15, putative  structural constituent of ribosome ? intracellular (IEA) ? ribosome ? protein biosynthesis ? \*\* also with: YLR029C, clust.pair #14 | YMR121C ? RNA binding ? structural constituent of ribosome ? cytosolic large ribosomal subunit (sensu Eukaryota) ? protein biosynthesis ? | 0  BLAST |
| PFD0770c ? ribosomal protein l15, putative  structural constituent of ribosome ? intracellular (IEA) ? ribosome ? protein biosynthesis ? \*\* also with: YMR121C, clust.pair #14 | YLR029C ? RNA binding ? structural constituent of ribosome ? cytosolic large ribosomal subunit (sensu Eukaryota) ? protein biosynthesis ? | 0  BLAST |
| PF11\_0272 ? ribosomal protein S18, putative  RNA binding (IEA) ? structural constituent of ribosome ? intracellular (IEA) ? ribosome (IEA) ? protein biosynthesis ? small ribosomal subunit ? \*\* also with: YML026C, clust.pair #14 | YDR450W ? telomere maintenance ? structural constituent of ribosome ? mitochondrion ? cytosolic small ribosomal subunit (sensu Eukaryota) ? protein biosynthesis ? | 0  BLAST |
| PF11\_0272 ? ribosomal protein S18, putative  RNA binding (IEA) ? structural constituent of ribosome ? intracellular (IEA) ? ribosome (IEA) ? protein biosynthesis ? small ribosomal subunit ? \*\* also with: YDR450W, clust.pair #14 | YML026C ? structural constituent of ribosome ? mitochondrion ? cytosolic small ribosomal subunit (sensu Eukaryota) ? protein biosynthesis ? | 0  BLAST |
| PF13\_0213 ? 60S ribosomal subunit protein L6e, putative  structural constituent of ribosome ? intracellular (IEA) ? ribosome (IEA) ? cytosolic large ribosomal subunit (sensu Eukaryota) ? protein biosynthesis ? \*\* also with: YML073C, clust.pair #14 | YLR448W ? ribosomal large subunit assembly and maintenance ? RNA binding ? structural constituent of ribosome ? cytosolic large ribosomal subunit (sensu Eukaryota) ? protein biosynthesis ? | 7e-16  BLAST |
| PF13\_0213 ? 60S ribosomal subunit protein L6e, putative  structural constituent of ribosome ? intracellular (IEA) ? ribosome (IEA) ? cytosolic large ribosomal subunit (sensu Eukaryota) ? protein biosynthesis ? \*\* also with: YLR448W, clust.pair #14 | YML073C ? ribosomal large subunit assembly and maintenance ? RNA binding ? structural constituent of ribosome ? cytosolic large ribosomal subunit (sensu Eukaryota) ? protein biosynthesis ? | 1e-14  BLAST |
| PF08\_0075 ? 60S ribosomal protein L13, putative  structural constituent of ribosome ? intracellular (IEA) ? ribosome (IEA) ? cytosolic large ribosomal subunit (sensu Eukaryota) ? protein biosynthesis ? \*\* also with: YMR142C, clust.pair #14 | YDL082W ? structural constituent of ribosome ? cytosolic large ribosomal subunit (sensu Eukaryota) ? protein biosynthesis ? | 3e-18  BLAST |
| PF08\_0075 ? 60S ribosomal protein L13, putative  structural constituent of ribosome ? intracellular (IEA) ? ribosome (IEA) ? cytosolic large ribosomal subunit (sensu Eukaryota) ? protein biosynthesis ? \*\* also with: YDL082W, clust.pair #14 | YMR142C ? telomere maintenance ? structural constituent of ribosome ? cytosolic large ribosomal subunit (sensu Eukaryota) ? protein biosynthesis ? | 3e-18  BLAST |
| PF11\_0043 ? 60S acidic ribosomal protein p1, putative  structural constituent of ribosome ? intracellular (IEA) ? ribosome (IEA) ? protein biosynthesis ? translational elongation (IEA) ? large ribosomal subunit ? | YDL081C ? telomere maintenance ? structural constituent of ribosome ? cytosolic large ribosomal subunit (sensu Eukaryota) ? protein biosynthesis ? translational elongation ? | 2e-06  BLAST |
| PFF0700c ? 60S ribosomal protein L19, putative  structural constituent of ribosome ? intracellular (IEA) ? ribosome (IEA) ? cytosolic large ribosomal subunit (sensu Eukaryota) ? protein biosynthesis ? \*\* also with: YBR084C-A, clust.pair #14 | YBL027W ? structural constituent of ribosome ? cytosolic large ribosomal subunit (sensu Eukaryota) ? protein biosynthesis ? | 7e-39  BLAST |
| PFF0700c ? 60S ribosomal protein L19, putative  structural constituent of ribosome ? intracellular (IEA) ? ribosome (IEA) ? cytosolic large ribosomal subunit (sensu Eukaryota) ? protein biosynthesis ? \*\* also with: YBL027W, clust.pair #14 | YBR084C-A ? structural constituent of ribosome ? cytosolic large ribosomal subunit (sensu Eukaryota) ? protein biosynthesis ? | 7e-39  BLAST |
| PFE0185c ? 60S ribosomal subunit protein L31, putative  structural constituent of ribosome ? intracellular (IEA) ? ribosome (IEA) ? protein biosynthesis ? large ribosomal subunit ? \*\* also with: YDL075W, clust.pair #14 | YLR406C ? structural constituent of ribosome ? cytosolic large ribosomal subunit (sensu Eukaryota) ? protein biosynthesis ? | 5e-21  BLAST |
| PFE0185c ? 60S ribosomal subunit protein L31, putative  structural constituent of ribosome ? intracellular (IEA) ? ribosome (IEA) ? protein biosynthesis ? large ribosomal subunit ? \*\* also with: YLR406C, clust.pair #14 | YDL075W ? structural constituent of ribosome ? cytosolic large ribosomal subunit (sensu Eukaryota) ? protein biosynthesis ? | 9e-21  BLAST |
| PFL2055w ? 40S ribosomal protein S17, putative  structural constituent of ribosome ? intracellular (IEA) ? mitochondrion ? ribosome (IEA) ? cytosolic small ribosomal subunit (sensu Eukaryota) ? protein biosynthesis ? \*\* also with: YML024W, clust.pair #14 | YDR447C ? ribosomal small subunit assembly and maintenance ? telomere maintenance ? structural constituent of ribosome ? cytosolic small ribosomal subunit (sensu Eukaryota) ? protein biosynthesis ? | 4e-33  BLAST |
| PFL2055w ? 40S ribosomal protein S17, putative  structural constituent of ribosome ? intracellular (IEA) ? mitochondrion ? ribosome (IEA) ? cytosolic small ribosomal subunit (sensu Eukaryota) ? protein biosynthesis ? \*\* also with: YDR447C, clust.pair #14 | YML024W ? ribosomal small subunit assembly and maintenance ? telomere maintenance ? structural constituent of ribosome ? cytosolic small ribosomal subunit (sensu Eukaryota) ? protein biosynthesis ? | 4e-33  BLAST |
| PF10\_0043 ? ribosomal protein L13, putative  structural constituent of ribosome ? intracellular (IEA) ? ribosome (IEA) ? cytosolic large ribosomal subunit (sensu Eukaryota) ? protein biosynthesis ? large ribosomal subunit (IEA) ? \*\* also with: YNL069C, clust.pair #14 | YIL133C ? RNA binding ? structural constituent of ribosome ? cytosolic large ribosomal subunit (sensu Eukaryota) ? protein biosynthesis ? | 0  BLAST |
| PF10\_0043 ? ribosomal protein L13, putative  structural constituent of ribosome ? intracellular (IEA) ? ribosome (IEA) ? cytosolic large ribosomal subunit (sensu Eukaryota) ? protein biosynthesis ? large ribosomal subunit (IEA) ? \*\* also with: YIL133C, clust.pair #14 | YNL069C ? RNA binding ? structural constituent of ribosome ? cytosolic large ribosomal subunit (sensu Eukaryota) ? protein biosynthesis ? | 0  BLAST |
| PF10\_0187 ? ribosomal protein L30e, putative  structural constituent of ribosome ? cytosolic large ribosomal subunit (sensu Eukaryota) ? protein biosynthesis ? | YGL030W ? structural constituent of ribosome ? cytoplasm ? cytosolic large ribosomal subunit (sensu Eukaryota) ? rRNA processing ? protein biosynthesis ? negative regulation of protein biosynthesis ? negative regulation of nuclear mRNA splicing, via spliceosome ? | 2e-26  BLAST |
| PFI0190w ? ribosomal protein L32, putative  structural constituent of ribosome (IEA) ? intracellular (IEA) ? ribosome (IEA) ? protein biosynthesis (IEA) ? | YBL092W ? structural constituent of ribosome ? cytosolic large ribosomal subunit (sensu Eukaryota) ? protein biosynthesis ? | 6e-22  BLAST |
| PF13\_0129 ? ribosomal protein L6 homologue, putative  RNA binding ? structural constituent of ribosome ? intracellular (IEA) ? ribosome (IEA) ? protein biosynthesis ? large ribosomal subunit ? \*\* also with: YNL067W, clust.pair #14 | YGL147C ? structural constituent of ribosome ? cytosolic large ribosomal subunit (sensu Eukaryota) ? protein biosynthesis ? | 9.99995e-41  BLAST |
| PF13\_0129 ? ribosomal protein L6 homologue, putative  RNA binding ? structural constituent of ribosome ? intracellular (IEA) ? ribosome (IEA) ? protein biosynthesis ? large ribosomal subunit ? \*\* also with: YGL147C, clust.pair #14 | YNL067W ? structural constituent of ribosome ? cytosolic large ribosomal subunit (sensu Eukaryota) ? protein biosynthesis ? | 9.99995e-41  BLAST |
| MAL7P1.81 ? eukaryotic translation initiation factor 3 37.28 kDa subunit, putative  translation initiation factor activity ? eukaryotic translation initiation factor 3 complex ? regulation of translational initiation ? | YMR146C ? translation initiation factor activity ? eukaryotic translation initiation factor 3 complex ? translational initiation ? | 0  BLAST |
| PF10\_0103 ? eukaryotic translation initiation factor 2, beta, putative  RNA binding ? translation initiation factor activity ? eukaryotic translation initiation factor 2 complex ? translational initiation ? | YPL237W ? translation initiation factor activity ? ribosome ? eukaryotic translation initiation factor 2 complex ? translational initiation ? | 6e-32  BLAST |
| PF13\_0228 ? 40S ribosomal subunit protein S6, putative  structural constituent of ribosome ? intracellular (IEA) ? ribosome (IEA) ? cytosolic small ribosomal subunit (sensu Eukaryota) ? protein biosynthesis ? \*\* also with: YPL090C, clust.pair #14 | YBR181C ? structural constituent of ribosome ? small nucleolar ribonucleoprotein complex ? cytoplasm ? cytosolic small ribosomal subunit (sensu Eukaryota) ? protein biosynthesis ? | 0  BLAST |
| PF13\_0228 ? 40S ribosomal subunit protein S6, putative  structural constituent of ribosome ? intracellular (IEA) ? ribosome (IEA) ? cytosolic small ribosomal subunit (sensu Eukaryota) ? protein biosynthesis ? \*\* also with: YBR181C, clust.pair #14 | YPL090C ? structural constituent of ribosome ? small nucleolar ribonucleoprotein complex ? cytosolic small ribosomal subunit (sensu Eukaryota) ? protein biosynthesis ? | 0  BLAST |
| PFC0735w ? 40S ribosomal protein S15A, putative  structural constituent of ribosome ? intracellular (IEA) ? ribosome (IEA) ? cytosolic small ribosomal subunit (sensu Eukaryota) ? protein biosynthesis ? \*\* also with: YLR367W, clust.pair #14 | YJL190C ? telomere maintenance ? structural constituent of ribosome ? cytosolic small ribosomal subunit (sensu Eukaryota) ? protein biosynthesis ? | 0  BLAST |
| PFC0735w ? 40S ribosomal protein S15A, putative  structural constituent of ribosome ? intracellular (IEA) ? ribosome (IEA) ? cytosolic small ribosomal subunit (sensu Eukaryota) ? protein biosynthesis ? \*\* also with: YJL190C, clust.pair #14 | YLR367W ? structural constituent of ribosome ? cytosolic small ribosomal subunit (sensu Eukaryota) ? protein biosynthesis ? | 0  BLAST |
| PF14\_0448 ? ribosomal protein S2, putative  structural constituent of ribosome ? intracellular (IEA) ? ribosome (IEA) ? cytosolic small ribosomal subunit (sensu Eukaryota) ? protein biosynthesis ? small ribosomal subunit (IEA) ? | YGL123W ? structural constituent of ribosome ? small nucleolar ribonucleoprotein complex ? cytosolic small ribosomal subunit (sensu Eukaryota) ? protein biosynthesis ? regulation of translational fidelity ? | 0  BLAST |
| PF14\_0231 ? ribosomal protein L7a, putative  structural constituent of ribosome ? intracellular (IEA) ? ribosome (IEA) ? cytosolic large ribosomal subunit (sensu Eukaryota) ? protein biosynthesis ? ribonucleoprotein complex (IEA) ? ribosome biogenesis and assembly (IEA) ? \*\* also with: YLL045C, clust.pair #14 | YHL033C ? structural constituent of ribosome ? cytosolic large ribosomal subunit (sensu Eukaryota) ? protein biosynthesis ? | 2.00386e-43  BLAST |
| PF14\_0231 ? ribosomal protein L7a, putative  structural constituent of ribosome ? intracellular (IEA) ? ribosome (IEA) ? cytosolic large ribosomal subunit (sensu Eukaryota) ? protein biosynthesis ? ribonucleoprotein complex (IEA) ? ribosome biogenesis and assembly (IEA) ? \*\* also with: YHL033C, clust.pair #14 | YLL045C ? structural constituent of ribosome ? cytosolic large ribosomal subunit (sensu Eukaryota) ? protein biosynthesis ? | 2.99878e-43  BLAST |
| PF08\_0076 ? 40S ribosomal protein S16, putative  structural constituent of ribosome ? intracellular (IEA) ? ribosome (IEA) ? cytosolic small ribosomal subunit (sensu Eukaryota) ? protein biosynthesis ? \*\* also with: YMR143W, clust.pair #14 | YDL083C ? telomere maintenance ? structural constituent of ribosome ? cytosolic small ribosomal subunit (sensu Eukaryota) ? protein biosynthesis ? | 9.80909e-45  BLAST |
| PF08\_0076 ? 40S ribosomal protein S16, putative  structural constituent of ribosome ? intracellular (IEA) ? ribosome (IEA) ? cytosolic small ribosomal subunit (sensu Eukaryota) ? protein biosynthesis ? \*\* also with: YDL083C, clust.pair #14 | YMR143W ? telomere maintenance ? structural constituent of ribosome ? cytosolic small ribosomal subunit (sensu Eukaryota) ? protein biosynthesis ? | 9.80909e-45  BLAST |
| PF13\_0014 ? 40S ribosomal protein S7 homologue, putative  structural constituent of ribosome ? intracellular (IEA) ? ribosome (IEA) ? cytosolic small ribosomal subunit (sensu Eukaryota) ? protein biosynthesis ? \*\* also with: YOR096W, clust.pair #14 | YNL096C ? structural constituent of ribosome ? small nucleolar ribonucleoprotein complex ? cytosolic small ribosomal subunit (sensu Eukaryota) ? protein biosynthesis ? | 5e-31  BLAST |
| PF13\_0014 ? 40S ribosomal protein S7 homologue, putative  structural constituent of ribosome ? intracellular (IEA) ? ribosome (IEA) ? cytosolic small ribosomal subunit (sensu Eukaryota) ? protein biosynthesis ? \*\* also with: YNL096C, clust.pair #14 | YOR096W ? structural constituent of ribosome ? small nucleolar ribonucleoprotein complex ? cytosolic small ribosomal subunit (sensu Eukaryota) ? protein biosynthesis ? | 6e-29  BLAST |
| PF14\_0296 ? ribosomal protein L14, putative  structural constituent of ribosome ? intracellular (IEA) ? ribosome (IEA) ? cytosolic small ribosomal subunit (sensu Eukaryota) ? protein biosynthesis ? apicoplast ? \*\* also with: YHL001W, clust.pair #14 | YKL006W ? RNA binding ? structural constituent of ribosome ? cytosolic large ribosomal subunit (sensu Eukaryota) ? protein biosynthesis ? | 0.004  BLAST |
| PF14\_0296 ? ribosomal protein L14, putative  structural constituent of ribosome ? intracellular (IEA) ? ribosome (IEA) ? cytosolic small ribosomal subunit (sensu Eukaryota) ? protein biosynthesis ? apicoplast ? \*\* also with: YKL006W, clust.pair #14 | YHL001W ? RNA binding ? structural constituent of ribosome ? cytosolic large ribosomal subunit (sensu Eukaryota) ? protein biosynthesis ? | 0.006  BLAST |
| PF14\_0027 ? ribosomal S27a, putative  structural constituent of ribosome ? intracellular (IEA) ? ribosome (IEA) ? cytosolic small ribosomal subunit (sensu Eukaryota) ? protein biosynthesis (IEA) ? protein modification (IEA) ? | YLR167W ? ribosomal small subunit assembly and maintenance ? structural constituent of ribosome ? cytoplasm ? cytosolic small ribosomal subunit (sensu Eukaryota) ? protein biosynthesis ? protein ubiquitination ? protein tag ? ribosome biogenesis and assembly ? | 1e-12  BLAST |
| PF11\_0250 ? high mobility group-like protein NHP2, putative  structural constituent of ribosome (IEA) ? intracellular (IEA) ? nucleus ? ribosome (IEA) ? protein biosynthesis (IEA) ? ribonucleoprotein complex (IEA) ? ribosome biogenesis and assembly (IEA) ? | YEL026W ? nuclear mRNA splicing, via spliceosome ? RNA binding ? nucleolus ? small nucleolar ribonucleoprotein complex ? processing of 20S pre-rRNA ? RNA splicing factor activity, transesterification mechanism ? ribosome biogenesis and assembly ? U4/U6 x U5 tri-snRNP complex ? | 3e-39  BLAST |
| MAL13P1.92 ? 40S ribosomal protein S15, putative  structural constituent of ribosome ? intracellular (IEA) ? ribosome (IEA) ? cytosolic small ribosomal subunit (sensu Eukaryota) ? protein biosynthesis ? small ribosomal subunit (IEA) ? | YOL040C ? ribosomal small subunit-nucleus export ? structural constituent of ribosome ? cytosolic small ribosomal subunit (sensu Eukaryota) ? protein biosynthesis ? | 5e-36  BLAST |
| PF08\_0039 ? ribosomal protein, putative  structural constituent of ribosome ? intracellular (IEA) ? ribosome (IEA) ? cytosolic large ribosomal subunit (sensu Eukaryota) ? protein biosynthesis ? | YLR061W ? structural constituent of ribosome ? cytosolic large ribosomal subunit (sensu Eukaryota) ? protein biosynthesis ? | 1e-07  BLAST |
| PF07\_0043 ? 60S ribosomal protein L34-a, putative  structural constituent of ribosome (IEA) ? intracellular (IEA) ? ribosome (IEA) ? protein biosynthesis (IEA) ? \*\* also with: YIL052C, clust.pair #14 | YER056C-A ? structural constituent of ribosome ? cytosolic large ribosomal subunit (sensu Eukaryota) ? protein biosynthesis ? | 1e-30  BLAST |
| PF07\_0043 ? 60S ribosomal protein L34-a, putative  structural constituent of ribosome (IEA) ? intracellular (IEA) ? ribosome (IEA) ? protein biosynthesis (IEA) ? \*\* also with: YER056C-A, clust.pair #14 | YIL052C ? telomere maintenance ? structural constituent of ribosome ? cytosolic large ribosomal subunit (sensu Eukaryota) ? protein biosynthesis ? | 1e-30  BLAST |
| PF13\_0049 ? 60S ribosomal protein L24, putative  structural constituent of ribosome ? intracellular (IEA) ? ribosome (IEA) ? cytosolic large ribosomal subunit (sensu Eukaryota) ? protein biosynthesis ? \*\* also with: YGL031C, clust.pair #14 | YGR148C ? RNA binding ? structural constituent of ribosome ? cytosolic large ribosomal subunit (sensu Eukaryota) ? protein biosynthesis ? | 3e-10  BLAST |
| PF13\_0049 ? 60S ribosomal protein L24, putative  structural constituent of ribosome ? intracellular (IEA) ? ribosome (IEA) ? cytosolic large ribosomal subunit (sensu Eukaryota) ? protein biosynthesis ? \*\* also with: YGR148C, clust.pair #14 | YGL031C ? RNA binding ? structural constituent of ribosome ? cytosolic large ribosomal subunit (sensu Eukaryota) ? protein biosynthesis ? | 4e-10  BLAST |
| PFC0400w ? 60S Acidic ribosomal protein P2  structural constituent of ribosome (IEA) ? intracellular (IEA) ? ribosome (IEA) ? cytosolic large ribosomal subunit (sensu Eukaryota) ? translational elongation ? large ribosomal subunit ? \*\* also with: YOL039W, clust.pair #14 | YDR382W ? structural constituent of ribosome ? cytosolic large ribosomal subunit (sensu Eukaryota) ? protein biosynthesis ? translational elongation ? | 9e-11  BLAST |
| PFC0400w ? 60S Acidic ribosomal protein P2  structural constituent of ribosome (IEA) ? intracellular (IEA) ? ribosome (IEA) ? cytosolic large ribosomal subunit (sensu Eukaryota) ? translational elongation ? large ribosomal subunit ? \*\* also with: YDR382W, clust.pair #14 | YOL039W ? structural constituent of ribosome ? cytosolic large ribosomal subunit (sensu Eukaryota) ? protein biosynthesis ? translational elongation ? | 2e-09  BLAST |

## Cluster Pair #15: 26 gene pairs.

| P.falciparum | S.cerevisiae | Blast evalue |
| --- | --- | --- |
| PF14\_0486 ? elongation factor 2  translation elongation factor activity ? GTP binding ? protein biosynthesis (IEA) ? translational elongation ? \*\* also with: YOR133W, clust.pair #15 | YDR385W ? translation elongation factor activity ? ribosome ? translational elongation ? | 0  BLAST |
| PF14\_0486 ? elongation factor 2  translation elongation factor activity ? GTP binding ? protein biosynthesis (IEA) ? translational elongation ? \*\* also with: YDR385W, clust.pair #15 | YOR133W ? translation elongation factor activity ? ribosome ? translational elongation ? | 0  BLAST |
| PF14\_0589 ? valine - tRNA ligase, putative  tRNA ligase activity (IEA) ? valine-tRNA ligase activity ? ATP binding (IEA) ? tRNA aminoacylation for protein translation (IEA) ? valyl-tRNA aminoacylation ? | YGR094W ? valine-tRNA ligase activity ? cytoplasm ? mitochondrion ? valyl-tRNA aminoacylation ? | 0  BLAST |
| PF13\_0214 ? elongation factor 1-gamma, putative  translation elongation factor activity ? glutathione transferase activity ? eukaryotic translation elongation factor 1 complex ? translational elongation ? | YKL081W ? translation elongation factor activity ? mitochondrion ? ribosome ? eukaryotic translation elongation factor 1 complex ? translational elongation ? | 4e-24  BLAST |
| PF11\_0270 ? threonine -- tRNA ligase, putative  tRNA ligase activity (IEA) ? threonine-tRNA ligase activity ? ATP binding ? mitochondrion ? protein biosynthesis (IEA) ? tRNA aminoacylation for protein translation (IEA) ? threonyl-tRNA aminoacylation ? apicoplast ? | YIL078W ? threonine-tRNA ligase activity ? cytoplasm ? mitochondrion ? protein biosynthesis ? | 0  BLAST |
| PFL0900c ? arginyl-tRNA synthetase, putative  arginine-tRNA ligase activity ? ATP binding (IEA) ? arginyl-tRNA aminoacylation ? apicoplast ? | YDR341C ? arginine-tRNA ligase activity ? cytoplasm ? mitochondrion ? protein biosynthesis ? | 3.9937e-43  BLAST |
| PF10\_0150 ? methionine aminopeptidase, putative  methionyl aminopeptidase activity ? regulation of protein biosynthesis ? regulation of translation ? protein modification ? proteolysis and peptidolysis ? metalloexopeptidase activity (IEA) ? | YLR244C ? methionyl aminopeptidase activity ? cytosolic ribosome (sensu Eukaryota) ? proteolysis and peptidolysis ? | 0  BLAST |
| PF10\_0149 ? cysteine -- tRNA ligase, putative  cysteine-tRNA ligase activity ? ATP binding (IEA) ? translational elongation ? cysteinyl-tRNA aminoacylation ? apicoplast ? | YNL247W ? cysteine-tRNA ligase activity ? cytoplasm ? ribosome ? cysteinyl-tRNA aminoacylation ? cysteine metabolism ? ribosome biogenesis and assembly ? | 0  BLAST |
| PF13\_0330 ? ATP-dependent DNA helicase, putative  nucleotide binding (IEA) ? ATP-dependent DNA helicase activity ? ATP binding (IEA) ? nucleus (IEA) ? nucleoside-triphosphatase activity (IEA) ? ATP-dependent 5' to 3' DNA helicase activity (IEA) ? | YPL235W ? SWR1 complex ? nucleus ? chromatin remodeling ? regulation of transcription from RNA polymerase II promoter ? 35S primary transcript processing ? snoRNA metabolism ? chromatin remodeling complex ? ATPase activity ? INO80 complex ? ATP-dependent 5' to 3' DNA helicase activity ? | 0  BLAST |
| PF13\_0170 ? glutaminyl-tRNA synthetase, putative  glutamate-tRNA ligase activity (IEA) ? glutamine-tRNA ligase activity (IEA) ? ATP binding (IEA) ? glutamyl-tRNA aminoacylation (IEA) ? | YOR168W ? glutamine-tRNA ligase activity ? cytoplasm ? glutaminyl-tRNA aminoacylation ? | 0  BLAST |
| PF14\_0230 ? Ribosomal protein family L5, putative  structural constituent of ribosome ? intracellular (IEA) ? mitochondrion ? ribosome (IEA) ? cytosolic large ribosomal subunit (sensu Eukaryota) ? protein biosynthesis ? 5S rRNA binding (IEA) ? | YPL131W ? ribosomal large subunit assembly and maintenance ? RNA binding ? structural constituent of ribosome ? cytosolic large ribosomal subunit (sensu Eukaryota) ? protein biosynthesis ? | 0  BLAST |
| PFE1195w ? karyopherin beta  nuclear pore ? | YMR308C ? nucleus ? cytoplasm ? mRNA-nucleus export ? protein-nucleus import ? protein carrier activity ? | 0  BLAST |
| PF14\_0198 ? glycine -- tRNA ligase, putative  tRNA ligase activity (IEA) ? glycine-tRNA ligase activity ? ATP binding (IEA) ? protein biosynthesis (IEA) ? tRNA aminoacylation for protein translation (IEA) ? glycyl-tRNA aminoacylation ? apicoplast ? | YBR121C ? glycine-tRNA ligase activity ? cytoplasm ? mitochondrion ? transcription termination ? glycyl-tRNA aminoacylation ? | 0  BLAST |
| PFE0885w ? eukaryotic translation initiation factor 3 subunit, putative  nucleic acid binding (IEA) ? | YOR361C ? translation initiation factor activity ? cytoplasm ? eukaryotic translation initiation factor 3 complex ? translational initiation ? | 0  BLAST |
| PFF0345w ? translation initiation factor IF-2, putative  translation initiation factor activity ? GTP binding (IEA) ? protein biosynthesis (IEA) ? translational initiation ? | YAL035W ? translation initiation factor activity ? GTPase activity ? mitochondrion ? cytosolic small ribosomal subunit (sensu Eukaryota) ? translational initiation ? | 0  BLAST |
| PF11\_0051 ? phenylalanine -- tRNA ligase, putative  phenylalanine-tRNA ligase activity ? ATP binding (IEA) ? cytoplasm (IEA) ? phenylalanyl-tRNA aminoacylation ? phenylalanine-tRNA ligase complex ? | YLR060W ? phenylalanine-tRNA ligase activity ? cytoplasm ? phenylalanyl-tRNA aminoacylation ? phenylalanine-tRNA ligase complex ? | 0  BLAST |
| PF13\_0179 ? isoleucine--tRNA ligase, putative  tRNA ligase activity (IEA) ? isoleucine-tRNA ligase activity (IEA) ? ATP binding (IEA) ? tRNA aminoacylation for protein translation (IEA) ? isoleucyl-tRNA aminoacylation (IEA) ? | YBL076C ? isoleucine-tRNA ligase activity ? cytosol ? protein biosynthesis ? | 0  BLAST |
| PF14\_0028 ? hypothetical protein, conserved  nucleic acid binding (IEA) ? RNA binding (IEA) ? RNA processing (IEA) ? ATP biosynthesis (IEA) ? ATP synthesis coupled proton transport (IEA) ? proton-transporting two-sector ATPase complex (IEA) ? hydrogen-transporting ATP synthase activity, rotational mechanism (IEA) ? hydrogen-transporting ATPase activity, rotational mechanism (IEA) ? \*\* also with: YER165W, clust.pair #17 \*\* also with: YGR159C, clust.pair #17 | YNL016W ? mRNA catabolism, nonsense-mediated decay ? nucleic acid binding ? mRNA binding ? nucleus ? cytoplasm ? heterogeneous nuclear ribonucleoprotein complex ? regulation of mRNA stability ? | 1e-06  BLAST |
| PF08\_0111 ? hypothetical protein  nucleic acid binding (IEA) ? helicase activity (IEA) ? ATP binding (IEA) ? ATP-dependent helicase activity (IEA) ? | YDL084W ? transcription export complex ? U2-type nuclear mRNA branch site recognition ? nuclear mRNA splicing, via spliceosome ? chromosome, telomeric region ? RNA binding ? ATP-dependent RNA helicase activity ? protein binding ? nucleus ? spliceosome complex ? chromatin silencing at telomere ? mRNA-nucleus export ? RNA splicing factor activity, transesterification mechanism ? | 3e-18  BLAST |
| PF13\_0354 ? alanine--tRNA ligase, putative  alanine-tRNA ligase activity ? ATP binding (IEA) ? alanyl-tRNA aminoacylation ? apicoplast ? | YOR335C ? alanine-tRNA ligase activity ? cytoplasm ? mitochondrion ? alanyl-tRNA aminoacylation ? | 0  BLAST |
| PFB0445c ? helicase, putative  nucleic acid binding (IEA) ? ATP-dependent RNA helicase activity ? helicase activity (IEA) ? ATP binding (IEA) ? ATP-dependent helicase activity (IEA) ? | YDL084W ? transcription export complex ? U2-type nuclear mRNA branch site recognition ? nuclear mRNA splicing, via spliceosome ? chromosome, telomeric region ? RNA binding ? ATP-dependent RNA helicase activity ? protein binding ? nucleus ? spliceosome complex ? chromatin silencing at telomere ? mRNA-nucleus export ? RNA splicing factor activity, transesterification mechanism ? | 0  BLAST |
| PFL0815w ? DNA-binding chaperone, putative  DNA binding (IEA) ? nucleus (IEA) ? protein folding (IEA) ? heat shock protein binding (IEA) ? unfolded protein binding (IEA) ? | YGR285C ? cytoplasm ? mitochondrion ? ribosome ? regulation of translational fidelity ? protein folding ? unfolded protein binding ? | 8e-17  BLAST |
| PFD1070w ? eukaryotic initiation factor, putative  nucleic acid binding (IEA) ? translation initiation factor activity ? ATP-dependent RNA helicase activity ? helicase activity (IEA) ? ATP binding (IEA) ? regulation of translational initiation ? ATP-dependent helicase activity (IEA) ? eukaryotic translation initiation factor 4F complex ? \*\* also with: YKR059W, clust.pair #15 | YJL138C ? RNA helicase activity ? translation initiation factor activity ? cytoplasm ? ribosome ? translational initiation ? regulation of translational initiation ? eukaryotic translation initiation factor 4F complex ? | 0  BLAST |
| PFD1070w ? eukaryotic initiation factor, putative  nucleic acid binding (IEA) ? translation initiation factor activity ? ATP-dependent RNA helicase activity ? helicase activity (IEA) ? ATP binding (IEA) ? regulation of translational initiation ? ATP-dependent helicase activity (IEA) ? eukaryotic translation initiation factor 4F complex ? \*\* also with: YJL138C, clust.pair #15 | YKR059W ? telomere maintenance ? translation initiation factor activity ? ATP-dependent RNA helicase activity ? ribosome ? translational initiation ? eukaryotic translation initiation factor 4F complex ? | 0  BLAST |
| PF13\_0262 ? lysine--tRNA ligase  nucleic acid binding (IEA) ? tRNA ligase activity (IEA) ? lysine-tRNA ligase activity ? ATP binding ? cytoplasm (IEA) ? tRNA aminoacylation for protein translation (IEA) ? lysyl-tRNA aminoacylation ? | YDR037W ? lysine-tRNA ligase activity ? cytoplasm ? lysyl-tRNA aminoacylation ? | 0  BLAST |
| PF14\_0125 ? deoxyhypusine synthase  protein biosynthesis ? hypusine biosynthesis from peptidyl-lysine ? membrane ? spermidine catabolism to deoxyhypusine, using deoxyhypusine synthase ? | YHR068W ? cytoplasm ? hypusine biosynthesis from peptidyl-lysine ? transferase activity, transferring alkyl or aryl (other than methyl) groups ? | 9.80909e-45  BLAST |

## Cluster Pair #16: 5 gene pairs.

| P.falciparum | S.cerevisiae | Blast evalue |
| --- | --- | --- |
| PFI1105w ? Phosphoglycerate kinase  phosphoglycerate kinase activity (IEA) ? glycolysis (IEA) ? | YCR012W ? phosphoglycerate kinase activity ? cytoplasm ? mitochondrion ? gluconeogenesis ? glycolysis ? | 0  BLAST |
| PF11\_0208 ? phosphoglycerate mutase, putative  phosphoglycerate mutase activity ? cytosol ? glycolysis ? intramolecular transferase activity, phosphotransferases (IEA) ? | YKL152C ? phosphoglycerate mutase activity ? mitochondrion ? cytosol ? gluconeogenesis ? glycolysis ? | 0  BLAST |
| PF10\_0155 ? enolase  phosphopyruvate hydratase complex ? phosphopyruvate hydratase activity ? gluconeogenesis ? glycolysis ? \*\* also with: YHR174W, clust.pair #16 | YGR254W ? phosphopyruvate hydratase complex ? phosphopyruvate hydratase activity ? cytoplasm ? gluconeogenesis ? glycolysis ? | 0  BLAST |
| PF10\_0155 ? enolase  phosphopyruvate hydratase complex ? phosphopyruvate hydratase activity ? gluconeogenesis ? glycolysis ? \*\* also with: YGR254W, clust.pair #16 | YHR174W ? phosphopyruvate hydratase complex ? phosphopyruvate hydratase activity ? soluble fraction ? gluconeogenesis ? glycolysis ? | 0  BLAST |
| PFB0200c ? aspartate aminotransferase, putative  amino acid metabolism ? transaminase activity ? biosynthesis (IEA) ? transferase activity, transferring nitrogenous groups (IEA) ? | YLR027C ? aspartate transaminase activity ? cytoplasm ? peroxisome ? aspartate biosynthesis ? aspartate catabolism ? glutamate metabolism ? nitrogen compound metabolism ? asparagine biosynthesis from oxaloacetate ? | 0  BLAST |

## Cluster Pair #17: 28 gene pairs.

| P.falciparum | S.cerevisiae | Blast evalue |
| --- | --- | --- |
| PF11\_0305 ? hypothetical protein | YNL061W ? nucleolus ? rRNA processing ? RNA methyltransferase activity ? S-adenosylmethionine-dependent methyltransferase activity ? ribosome biogenesis and assembly ? | 0  BLAST |
| PF14\_0261 ? proliferation-associated protein 2g4, putative  methionyl aminopeptidase activity ? proteolysis and peptidolysis (IEA) ? cell cycle arrest ? metalloexopeptidase activity (IEA) ? cell proliferation ? | YDR101C ? nucleoplasm ? cytoplasm ? cytosolic large ribosomal subunit (sensu Eukaryota) ? ribosome biogenesis and assembly ? ribosomal large subunit biogenesis ? | 8e-14  BLAST |
| PF14\_0104 ? eukaryotic translation initiation factor 2 gamma subunit, putative  GTP binding ? eukaryotic translation initiation factor 2 complex ? protein biosynthesis (IEA) ? translational initiation ? | YER025W ? translation initiation factor activity ? ribosome ? eukaryotic translation initiation factor 2 complex ? translational initiation ? | 0  BLAST |
| PF11\_0245 ? translation elongation factor EF-1, subunit alpha, putative  translation elongation factor activity ? translation release factor activity (IEA) ? GTP binding ? eukaryotic translation elongation factor 1 complex ? protein biosynthesis (IEA) ? translational elongation ? translational termination (IEA) ? | YDR172W ? mRNA catabolism, deadenylylation-dependent decay ? translation release factor activity ? cytosol ? translational termination ? translation release factor complex ? | 0  BLAST |
| PF13\_0157 ? ribose-phosphate pyrophosphokinase, putative  histidine biosynthesis ? tryptophan biosynthesis ? ribose phosphate diphosphokinase activity ? nucleoside metabolism ? nucleotide biosynthesis (IEA) ? apicoplast ? | YKL181W ? histidine biosynthesis ? tryptophan biosynthesis ? ribose phosphate diphosphokinase activity ? cytoplasm ? purine ribonucleoside salvage ? 'de novo' IMP biosynthesis ? 'de novo' pyrimidine base biosynthesis ? | 1e-35  BLAST |
| PF13\_0143 ? phosphoribosylpyrophosphate synthetase  ribose phosphate diphosphokinase activity ? pentose-phosphate shunt ? purine ribonucleoside salvage ? nucleoside metabolism (IEA) ? nucleotide biosynthesis (IEA) ? \*\* also with: YHL011C, clust.pair #17 | YBL068W ? histidine biosynthesis ? tryptophan biosynthesis ? ribose phosphate diphosphokinase activity ? cytoplasm ? purine ribonucleoside salvage ? 'de novo' IMP biosynthesis ? 'de novo' pyrimidine base biosynthesis ? ribosome biogenesis and assembly ? | 0  BLAST |
| PF13\_0143 ? phosphoribosylpyrophosphate synthetase  ribose phosphate diphosphokinase activity ? pentose-phosphate shunt ? purine ribonucleoside salvage ? nucleoside metabolism (IEA) ? nucleotide biosynthesis (IEA) ? \*\* also with: YBL068W, clust.pair #17 | YHL011C ? histidine biosynthesis ? tryptophan biosynthesis ? telomere maintenance ? ribose phosphate diphosphokinase activity ? cytoplasm ? purine ribonucleoside salvage ? 'de novo' IMP biosynthesis ? 'de novo' pyrimidine base biosynthesis ? regulation of cell size ? ribosome biogenesis and assembly ? | 0  BLAST |
| PFD0150w ? hypothetical protein | YKR092C ? nucleolus ? nucleocytoplasmic transport ? ribosome biogenesis and assembly ? | 0.001  BLAST |
| PFL0310c ? eukaryotic translation initiation factor 3 subunit 8, putative  translation initiation factor activity ? eukaryotic translation initiation factor 3 complex ? translational initiation (IEA) ? regulation of translational initiation ? | YMR309C ? translation initiation factor activity ? cytoplasm ? eukaryotic translation initiation factor 3 complex ? translational initiation ? ribosome biogenesis and assembly ? | 9.99995e-41  BLAST |
| PFL1745c ? clustered-asparagine-rich protein  nucleic acid binding (IEA) ? RNA binding ? | YER165W ? nucleus ? cytoplasm ? ribosome ? regulation of translational initiation ? poly(A) binding ? | 4e-08  BLAST |
| MAL13P1.294 ? GTP-binding protein, putative  GTP binding ? signal transduction ? \*\* also with: YGR173W, clust.pair #17 | YAL036C ? GTP binding ? cytoplasm ? ribosome biogenesis and assembly ? | 0  BLAST |
| MAL13P1.294 ? GTP-binding protein, putative  GTP binding ? signal transduction ? \*\* also with: YAL036C, clust.pair #17 | YGR173W ? GTP binding ? cytoplasm ? | 0  BLAST |
| MAL13P1.289 ? mitotic control protein dis3 homologue, putative  exosome (RNase complex) ? RNA binding ? exonuclease activity ? ribonuclease activity (IEA) ? | YOL021C ? 3'-5'-exoribonuclease activity ? nuclear exosome (RNase complex) ? cytoplasmic exosome (RNase complex) ? mitochondrion ? 35S primary transcript processing ? mRNA catabolism ? | 0  BLAST |
| PF14\_0207 ? RNA polymerase subunit, putative  DNA binding (IEA) ? DNA-directed RNA polymerase activity ? nucleus (IEA) ? transcription (IEA) ? regulation of transcription, DNA-dependent ? membrane ? | YNR003C ? DNA-directed RNA polymerase activity ? nucleus ? DNA-directed RNA polymerase III complex ? cytoplasm ? transcription from RNA polymerase III promoter ? ribosome biogenesis and assembly ? | 3e-11  BLAST |
| PFC0365w ? conserved protein, putative  ubiquitin ligase complex (IEA) ? ubiquitin-protein ligase activity (IEA) ? nuclear matrix ? protein ubiquitination (IEA) ? | YCR057C ? cytokinesis ? small nucleolar ribonucleoprotein complex ? cytoplasm ? 35S primary transcript processing ? establishment of cell polarity (sensu Fungi) ? processing of 20S pre-rRNA ? snoRNA binding ? 90S preribosome ? ribosome biogenesis and assembly ? | 2e-19  BLAST |
| PFL2125c ? hypothetical protein | YPL122C ? nucleotide excision repair factor 3 complex ? nucleotide-excision repair, DNA duplex unwinding ? transcription factor TFIIH complex ? nucleotide-excision repair ? transcription initiation from RNA polymerase II promoter ? negative regulation of transcription from RNA polymerase II promoter, mitotic ? general RNA polymerase II transcription factor activity ? | 3e-16  BLAST |
| PF14\_0028 ? hypothetical protein, conserved  nucleic acid binding (IEA) ? RNA binding (IEA) ? RNA processing (IEA) ? ATP biosynthesis (IEA) ? ATP synthesis coupled proton transport (IEA) ? proton-transporting two-sector ATPase complex (IEA) ? hydrogen-transporting ATP synthase activity, rotational mechanism (IEA) ? hydrogen-transporting ATPase activity, rotational mechanism (IEA) ? \*\* also with: YNL016W, clust.pair #15 \*\* also with: YGR159C, clust.pair #17 | YER165W ? nucleus ? cytoplasm ? ribosome ? regulation of translational initiation ? poly(A) binding ? | 6e-06  BLAST |
| PF14\_0028 ? hypothetical protein, conserved  nucleic acid binding (IEA) ? RNA binding (IEA) ? RNA processing (IEA) ? ATP biosynthesis (IEA) ? ATP synthesis coupled proton transport (IEA) ? proton-transporting two-sector ATPase complex (IEA) ? hydrogen-transporting ATP synthase activity, rotational mechanism (IEA) ? hydrogen-transporting ATPase activity, rotational mechanism (IEA) ? \*\* also with: YNL016W, clust.pair #15 \*\* also with: YER165W, clust.pair #17 | YGR159C ? ribosomal small subunit assembly and maintenance ? telomere maintenance ? single-stranded DNA binding ? RNA binding ? nucleus ? nucleolus ? mitochondrion ? rRNA processing ? | 7e-06  BLAST |
| PFE0655w ? hypothetical protein | YDL167C ? cytoplasm ? ribosome biogenesis and assembly ? | 0.003  BLAST |
| MAL13P1.52 ? hypothetical protein | YNL313C ? karyogamy during conjugation with cellular fusion ? nucleus ? cytoplasm ? | 1e-13  BLAST |
| PFI0625c ? hypothetical protein  translation initiation factor activity (IEA) ? regulation of translational initiation (IEA) ? | YNL062C ? tRNA binding ? nucleus ? translational initiation ? tRNA (adenine-N1-)-methyltransferase activity ? tRNA methylation ? ribosome biogenesis and assembly ? | 5e-07  BLAST |
| PF13\_0315 ? RNA binding protein, putative  nucleic acid binding (IEA) ? RNA binding ? \*\* also with: YOL123W, clust.pair #17 | YGR159C ? ribosomal small subunit assembly and maintenance ? telomere maintenance ? single-stranded DNA binding ? RNA binding ? nucleus ? nucleolus ? mitochondrion ? rRNA processing ? | 7e-14  BLAST |
| PF13\_0315 ? RNA binding protein, putative  nucleic acid binding (IEA) ? RNA binding ? \*\* also with: YGR159C, clust.pair #17 | YOL123W ? RNA binding ? nucleus ? cytoplasm ? mRNA cleavage factor complex ? mRNA polyadenylylation ? mRNA cleavage ? | 1e-13  BLAST |
| PFL0670c ? Bi-functional aminoacyl-tRNA synthetase, putative  tRNA ligase activity (IEA) ? glutamate-tRNA ligase activity ? proline-tRNA ligase activity ? ATP binding (IEA) ? cytoplasm (IEA) ? protein biosynthesis (IEA) ? tRNA aminoacylation for protein translation ? prolyl-tRNA aminoacylation (IEA) ? | YHR020W ? proline-tRNA ligase activity ? ribosome ? tRNA aminoacylation for protein translation ? | 0  BLAST |
| PFE1335c ? hypothetical protein | YGR162W ? translation initiation factor activity ? mitochondrion ? ribosome ? translational initiation ? eukaryotic translation initiation factor 4F complex ? ribosome biogenesis and assembly ? | 0.004  BLAST |
| PFI0860c ? ATP-dependant RNA helicase, putative  nucleic acid binding (IEA) ? helicase activity (IEA) ? ATP binding (IEA) ? ATP-dependent helicase activity (IEA) ? | YGL120C ? U2-type spliceosome disassembly ? ATP-dependent RNA helicase activity ? spliceosome complex ? mitochondrion ? rRNA processing ? 35S primary transcript processing ? processing of 27S pre-rRNA ? processing of 20S pre-rRNA ? RNA splicing factor activity, transesterification mechanism ? ribosome biogenesis and assembly ? ribosomal large subunit biogenesis ? | 0  BLAST |
| PFD0460c ? hypothetical protein | YBR034C ? nucleus ? mRNA-nucleus export ? protein-arginine N-methyltransferase activity ? peptidyl-arginine modification ? ribosome biogenesis and assembly ? | 0.1  BLAST |
| PFE0515w ? hypothetical protein | YOL022C ? cytoplasm ? | 0.005  BLAST |

## Cluster Pair #18: 24 gene pairs.

| P.falciparum | S.cerevisiae | Blast evalue |
| --- | --- | --- |
| PFE0845c ? 60S ribosomal subunit protein L8, putative  nucleic acid binding (IEA) ? structural constituent of ribosome ? intracellular (IEA) ? ribosome (IEA) ? protein biosynthesis ? large ribosomal subunit ? \*\* also with: YIL018W, clust.pair #18 | YFR031C-A ? structural constituent of ribosome ? cytosolic large ribosomal subunit (sensu Eukaryota) ? protein biosynthesis ? | 0  BLAST |
| PFE0845c ? 60S ribosomal subunit protein L8, putative  nucleic acid binding (IEA) ? structural constituent of ribosome ? intracellular (IEA) ? ribosome (IEA) ? protein biosynthesis ? large ribosomal subunit ? \*\* also with: YFR031C-A, clust.pair #18 | YIL018W ? structural constituent of ribosome ? cytosolic large ribosomal subunit (sensu Eukaryota) ? protein biosynthesis ? response to drug ? | 0  BLAST |
| PF13\_0224 ? 60S ribosomal subunit protein L18, putative  structural constituent of ribosome ? intracellular (IEA) ? ribosome (IEA) ? cytosolic large ribosomal subunit (sensu Eukaryota) ? protein biosynthesis ? \*\* also with: YOR312C, clust.pair #18 | YMR242C ? structural constituent of ribosome ? cytosolic large ribosomal subunit (sensu Eukaryota) ? protein biosynthesis ? | 4e-34  BLAST |
| PF13\_0224 ? 60S ribosomal subunit protein L18, putative  structural constituent of ribosome ? intracellular (IEA) ? ribosome (IEA) ? cytosolic large ribosomal subunit (sensu Eukaryota) ? protein biosynthesis ? \*\* also with: YMR242C, clust.pair #18 | YOR312C ? structural constituent of ribosome ? cytosolic large ribosomal subunit (sensu Eukaryota) ? protein biosynthesis ? ribosome biogenesis and assembly ? | 4e-34  BLAST |
| PFE0810c ? 40S ribosomal subunit protein S14, putative  RNA binding ? structural constituent of ribosome (IEA) ? intracellular (IEA) ? ribosome (IEA) ? cytosolic small ribosomal subunit (sensu Eukaryota) ? protein biosynthesis ? \*\* also with: YJL191W, clust.pair #18 | YCR031C ? ribosomal small subunit assembly and maintenance ? telomere maintenance ? RNA binding ? structural constituent of ribosome ? small nucleolar ribonucleoprotein complex ? cytosolic small ribosomal subunit (sensu Eukaryota) ? protein biosynthesis ? processing of 20S pre-rRNA ? | 5.60519e-45  BLAST |
| PFE0810c ? 40S ribosomal subunit protein S14, putative  RNA binding ? structural constituent of ribosome (IEA) ? intracellular (IEA) ? ribosome (IEA) ? cytosolic small ribosomal subunit (sensu Eukaryota) ? protein biosynthesis ? \*\* also with: YCR031C, clust.pair #18 | YJL191W ? ribosomal small subunit assembly and maintenance ? RNA binding ? structural constituent of ribosome ? small nucleolar ribonucleoprotein complex ? cytosolic small ribosomal subunit (sensu Eukaryota) ? protein biosynthesis ? processing of 20S pre-rRNA ? | 5.60519e-45  BLAST |
| PFB0830w ? Ribosomal protein S26e, putative  structural constituent of ribosome ? intracellular (IEA) ? mitochondrion ? ribosome (IEA) ? cytosolic small ribosomal subunit (sensu Eukaryota) ? protein biosynthesis ? \*\* also with: YGL189C, clust.pair #18 | YER131W ? structural constituent of ribosome ? cytosolic small ribosomal subunit (sensu Eukaryota) ? protein biosynthesis ? | 2e-26  BLAST |
| PFB0830w ? Ribosomal protein S26e, putative  structural constituent of ribosome ? intracellular (IEA) ? mitochondrion ? ribosome (IEA) ? cytosolic small ribosomal subunit (sensu Eukaryota) ? protein biosynthesis ? \*\* also with: YER131W, clust.pair #18 | YGL189C ? structural constituent of ribosome ? cytosolic small ribosomal subunit (sensu Eukaryota) ? protein biosynthesis ? | 2e-26  BLAST |
| PF07\_0080 ? 40S ribosomal protein S10, putative  structural constituent of ribosome ? cytosolic small ribosomal subunit (sensu Eukaryota) ? protein biosynthesis ? \*\* also with: YOR293W, clust.pair #18 | YMR230W ? structural constituent of ribosome ? cytosolic small ribosomal subunit (sensu Eukaryota) ? protein biosynthesis ? | 1e-20  BLAST |
| PF07\_0080 ? 40S ribosomal protein S10, putative  structural constituent of ribosome ? cytosolic small ribosomal subunit (sensu Eukaryota) ? protein biosynthesis ? \*\* also with: YMR230W, clust.pair #18 | YOR293W ? structural constituent of ribosome ? cytosolic small ribosomal subunit (sensu Eukaryota) ? protein biosynthesis ? | 2e-20  BLAST |
| PFB0455w ? ribosomal L37ae protein, putative  structural constituent of ribosome ? intracellular (IEA) ? mitochondrion ? ribosome (IEA) ? cytosolic large ribosomal subunit (sensu Eukaryota) ? protein biosynthesis ? \*\* also with: YPR043W, clust.pair #18 | YJR094W-A ? structural constituent of ribosome ? cytosolic large ribosomal subunit (sensu Eukaryota) ? protein biosynthesis ? | 9e-26  BLAST |
| PFB0455w ? ribosomal L37ae protein, putative  structural constituent of ribosome ? intracellular (IEA) ? mitochondrion ? ribosome (IEA) ? cytosolic large ribosomal subunit (sensu Eukaryota) ? protein biosynthesis ? \*\* also with: YJR094W-A, clust.pair #18 | YPR043W ? structural constituent of ribosome ? cytosolic large ribosomal subunit (sensu Eukaryota) ? protein biosynthesis ? | 9e-26  BLAST |
| PF11\_0454 ? Ribosomal protein, 40S subunit, putative  structural constituent of ribosome ? intracellular (IEA) ? ribosome (IEA) ? cytosolic small ribosomal subunit (sensu Eukaryota) ? protein biosynthesis ? \*\* also with: YJL136C, clust.pair #18 | YKR057W ? telomere maintenance ? structural constituent of ribosome ? cytosolic small ribosomal subunit (sensu Eukaryota) ? protein biosynthesis ? | 4e-17  BLAST |
| PF11\_0454 ? Ribosomal protein, 40S subunit, putative  structural constituent of ribosome ? intracellular (IEA) ? ribosome (IEA) ? cytosolic small ribosomal subunit (sensu Eukaryota) ? protein biosynthesis ? \*\* also with: YKR057W, clust.pair #18 | YJL136C ? structural constituent of ribosome ? cytosolic small ribosomal subunit (sensu Eukaryota) ? protein biosynthesis ? | 6e-17  BLAST |
| PF13\_0045 ? 40S ribosomal protein S27, putative  structural constituent of ribosome ? intracellular (IEA) ? ribosome (IEA) ? cytosolic small ribosomal subunit (sensu Eukaryota) ? protein biosynthesis ? \*\* also with: YKL156W, clust.pair #18 | YHR021C ? telomere maintenance ? structural constituent of ribosome ? cytosolic small ribosomal subunit (sensu Eukaryota) ? protein biosynthesis ? | 5e-27  BLAST |
| PF13\_0045 ? 40S ribosomal protein S27, putative  structural constituent of ribosome ? intracellular (IEA) ? ribosome (IEA) ? cytosolic small ribosomal subunit (sensu Eukaryota) ? protein biosynthesis ? \*\* also with: YHR021C, clust.pair #18 | YKL156W ? structural constituent of ribosome ? cytoplasm ? cytosolic small ribosomal subunit (sensu Eukaryota) ? protein biosynthesis ? | 5e-27  BLAST |
| PFC0775w ? 40S ribosomal protein S11, putative  nucleic acid binding (IEA) ? structural constituent of ribosome ? intracellular (IEA) ? ribosome (IEA) ? cytosolic small ribosomal subunit (sensu Eukaryota) ? protein biosynthesis ? \*\* also with: YDR025W, clust.pair #18 | YBR048W ? ribosomal small subunit assembly and maintenance ? telomere maintenance ? structural constituent of ribosome ? cytosolic small ribosomal subunit (sensu Eukaryota) ? protein biosynthesis ? regulation of translational fidelity ? | 0  BLAST |
| PFC0775w ? 40S ribosomal protein S11, putative  nucleic acid binding (IEA) ? structural constituent of ribosome ? intracellular (IEA) ? ribosome (IEA) ? cytosolic small ribosomal subunit (sensu Eukaryota) ? protein biosynthesis ? \*\* also with: YBR048W, clust.pair #18 | YDR025W ? ribosomal small subunit assembly and maintenance ? structural constituent of ribosome ? cytosolic small ribosomal subunit (sensu Eukaryota) ? protein biosynthesis ? regulation of translational fidelity ? | 0  BLAST |
| PF11\_0438 ? Ribosomal protein, putative  structural constituent of ribosome ? intracellular (IEA) ? ribosome (IEA) ? protein biosynthesis ? large ribosomal subunit ? \*\* also with: YPL143W, clust.pair #18 | YOR234C ? structural constituent of ribosome ? cytosolic large ribosomal subunit (sensu Eukaryota) ? protein biosynthesis ? | 1e-30  BLAST |
| PF11\_0438 ? Ribosomal protein, putative  structural constituent of ribosome ? intracellular (IEA) ? ribosome (IEA) ? protein biosynthesis ? large ribosomal subunit ? \*\* also with: YOR234C, clust.pair #18 | YPL143W ? structural constituent of ribosome ? cytosolic large ribosomal subunit (sensu Eukaryota) ? protein biosynthesis ? | 1e-30  BLAST |
| PFC0535w ? 60S ribosomal protein L26, putative  structural constituent of ribosome ? intracellular (IEA) ? ribosome (IEA) ? cytosolic large ribosomal subunit (sensu Eukaryota) ? protein biosynthesis ? large ribosomal subunit (IEA) ? \*\* also with: YLR344W, clust.pair #18 | YGR034W ? RNA binding ? structural constituent of ribosome ? cytosolic large ribosomal subunit (sensu Eukaryota) ? protein biosynthesis ? | 2e-28  BLAST |
| PFC0535w ? 60S ribosomal protein L26, putative  structural constituent of ribosome ? intracellular (IEA) ? ribosome (IEA) ? cytosolic large ribosomal subunit (sensu Eukaryota) ? protein biosynthesis ? large ribosomal subunit (IEA) ? \*\* also with: YGR034W, clust.pair #18 | YLR344W ? RNA binding ? structural constituent of ribosome ? cytosolic large ribosomal subunit (sensu Eukaryota) ? protein biosynthesis ? | 1e-28  BLAST |
| PF13\_0171 ? 60S ribosomal protein L23, putative  structural constituent of ribosome (IEA) ? intracellular (IEA) ? ribosome (IEA) ? protein biosynthesis (IEA) ? \*\* also with: YER117W, clust.pair #18 | YBL087C ? structural constituent of ribosome ? cytosolic large ribosomal subunit (sensu Eukaryota) ? protein biosynthesis ? | 0  BLAST |
| PF13\_0171 ? 60S ribosomal protein L23, putative  structural constituent of ribosome (IEA) ? intracellular (IEA) ? ribosome (IEA) ? protein biosynthesis (IEA) ? \*\* also with: YBL087C, clust.pair #18 | YER117W ? structural constituent of ribosome ? cytosolic large ribosomal subunit (sensu Eukaryota) ? protein biosynthesis ? response to drug ? | 0  BLAST |

## Cluster Pair #19: 5 gene pairs.

| P.falciparum | S.cerevisiae | Blast evalue |
| --- | --- | --- |
| PF10\_0114 ? DNA repair protein RAD23, putative  damaged DNA binding ? nucleus (IEA) ? nucleotide-excision repair ? protein modification (IEA) ? | YEL037C ? repairosome ? nucleotide excision repair factor 2 complex ? proteasome complex (sensu Eukaryota) ? nucleotide-excision repair, DNA damage recognition ? damaged DNA binding ? mitochondrion ? ER-associated protein catabolism ? protein binding, bridging ? negative regulation of protein catabolism ? | 1e-06  BLAST |
| PF14\_0520 ? 6-phosphogluconate dehydrogenase, decarboxylating, putative  phosphogluconate dehydrogenase (decarboxylating) activity ? pentose-phosphate shunt ? | YHR183W ? phosphogluconate dehydrogenase (decarboxylating) activity ? cytoplasm ? mitochondrion ? response to oxidative stress ? pentose-phosphate shunt, oxidative branch ? | 0  BLAST |
| PFE0285c ? ubiquitin-like protein, putative  protein modification (IEA) ? ubiquitin cycle ? modification-dependent protein catabolism ? protein ubiquitination during ubiquitin-dependent protein catabolism ? | YDR510W ? nucleus ? septin ring ? protein sumoylation ? protein tag ? | 1e-18  BLAST |
| PF14\_0137 ? hypothetical protein  protein folding (IEA) ? membrane ? heat shock protein binding (IEA) ? unfolded protein binding (IEA) ? | YMR214W ? endoplasmic reticulum lumen ? protein folding ? ER-associated protein catabolism ? unfolded protein response ? chaperone binding ? | 2e-09  BLAST |
| PFI1505c ? hypothetical protein  GTP binding (IEA) ? protein biosynthesis (IEA) ? | YKR084C ? cytoplasm ? protein biosynthesis ? | 1e-10  BLAST |

## Cluster Pair #20: 3 gene pairs.

| P.falciparum | S.cerevisiae | Blast evalue |
| --- | --- | --- |
| PFI1070c ? hypothetical protein | YHR088W ? ribosomal large subunit assembly and maintenance ? nucleolus ? processing of 27S pre-rRNA ? rRNA primary transcript binding ? ribosome biogenesis and assembly ? | 5e-18  BLAST |
| PF13\_0310 ? hypothetical protein | YDL153C ? nucleus ? small nucleolar ribonucleoprotein complex ? establishment and/or maintenance of chromatin architecture ? processing of 20S pre-rRNA ? snoRNA binding ? ribosome biogenesis and assembly ? | 2e-06  BLAST |
| PF07\_0083 ? hypothetical protein, conserved | YNR054C ? nucleolus ? small nucleolar ribonucleoprotein complex ? cytoplasm ? 35S primary transcript processing ? transcription regulator activity ? ribosome biogenesis and assembly ? | 6e-15  BLAST |

## Cluster Pair #21: 5 gene pairs.

| P.falciparum | S.cerevisiae | Blast evalue |
| --- | --- | --- |
| PF10\_0086 ? adenylate kinase, putative  adenylate kinase activity ? ATP binding (IEA) ? mitochondrion ? nucleobase, nucleoside, nucleotide and nucleic acid metabolism (IEA) ? phosphotransferase activity, phosphate group as acceptor (IEA) ? nucleotide kinase activity (IEA) ? | YDR226W ? adenylate kinase activity ? cytoplasm ? mitochondrion ? mitochondrial intermembrane space ? ADP biosynthesis ? nucleotide metabolism ? | 0  BLAST |
| PF08\_0087 ? importin alpha, putative  nuclear pore ? NLS-bearing substrate-nucleus import ? | YNL189W ? nucleus ? cytoplasm ? nucleocytoplasmic transport ? protein carrier activity ? | 0  BLAST |
| MAL8P1.156 ? hypothetical protein | YER003C ? cell wall mannoprotein biosynthesis ? mannose-6-phosphate isomerase activity ? nucleus ? cytoplasm ? protein amino acid glycosylation ? GDP-mannose biosynthesis ? | 1e-08  BLAST |
| PFA0300c ? vacuolar ATP synthase, putative  ATP binding (IEA) ? proton-transporting ATP synthase complex (sensu Eukaryota) ? ATP synthesis coupled proton transport ? proton-transporting two-sector ATPase complex (IEA) ? hydrolase activity, acting on acid anhydrides, catalyzing transmembrane movement of substances (IEA) ? hydrogen-transporting ATPase activity, rotational mechanism ? | YKL080W ? hydrogen-transporting ATPase V1 domain ? vacuolar membrane (sensu Fungi) ? vacuolar acidification ? hydrogen-transporting ATPase activity, rotational mechanism ? | 9e-19  BLAST |
| PFF0395c ? hypothetical protein, conserved | YBR246W ? | 2e-12  BLAST |

## Cluster Pair #22: 2 gene pairs.

| P.falciparum | S.cerevisiae | Blast evalue |
| --- | --- | --- |
| PFE0795c ? nif-like protein, putative  phosphoric monoester hydrolase activity (IEA) ? | YLL010C ? phosphoprotein phosphatase activity ? plasma membrane ? response to stress ? | 4e-25  BLAST |
| PFI1265w ? hypothetical protein | YHR077C ? mRNA catabolism, nonsense-mediated decay ? telomere maintenance ? protein binding ? cytoplasm ? polysome ? mRNA catabolism ? translational frameshifting ? | 7e-12  BLAST |

## Cluster Pair #23: 3 gene pairs.

| P.falciparum | S.cerevisiae | Blast evalue |
| --- | --- | --- |
| PFE0625w ? GTPase, putative  GTP binding (IEA) ? intracellular protein transport (IEA) ? small GTPase mediated signal transduction (IEA) ? protein transport (IEA) ? | YFL038C ? Golgi membrane ? GTPase activity ? mitochondrion ? endoplasmic reticulum membrane ? protein complex assembly ? ER to Golgi transport ? | 0  BLAST |
| PFA0335w ? P. falciparum GTP binding protein RAB5  GTPase activity ? GTP binding (IEA) ? intracellular protein transport (IEA) ? small GTPase mediated signal transduction ? protein transport (IEA) ? | YOR089C ? GTPase activity ? mitochondrion ? mitochondrial outer membrane ? late endosome ? protein targeting to vacuole ? endocytosis ? | 0  BLAST |
| PF07\_0102 ? hypothetical protein | YMR028W ? protein binding ? signal transduction ? | 0.008  BLAST |
