# Supplementary material for: Assessing functional annotation transfers with inter-species conserved coexpression: application to Plasmodium falciparum
Source: BMC Genomics. 2010 Jan 15;11:35. doi: 10.1186/1471-2164-11-35 (PMC2826313; doi:10.1186/1471-2164-11-35)
Supplement: Additional file 2 — Le Roch - Spellman analysis. This file presents the cluster pairs identified as revealing a conservation of coexpression when comparing the Le Roch and Spellman data. This file also provide additional information on the available functional annotations, as well as links to the BLAST alignments and the different databases (click on the '?'s to access PlasmoDB, SGD, and Amigo databases). Gene functional annotations are as follows. The short description immediately following each P. falciparum gene comes from PlasmoDB (red = functional gene, blue = putative gene, black = hypothetical gene). Other annotations are Gene Ontology annotations (red = Molecular Function, green = Biological Process, blue = Cellular Component). [file 1471-2164-11-35-S2.HTML]

# Leroch - Spellman co-coexpression analysis

# 33 cluster pairs

## Cluster Pair #0: 11 gene pairs.

|  |  |  |  |  |  |  |  |  |  |  |  |  |  |  |  |  |  |  |  |  |  |  |  |  |  |  |  |  |  |  |  |  |  |  |  |
| --- | --- | --- | --- | --- | --- | --- | --- | --- | --- | --- | --- | --- | --- | --- | --- | --- | --- | --- | --- | --- | --- | --- | --- | --- | --- | --- | --- | --- | --- | --- | --- | --- | --- | --- | --- |
| P. falciparum S. cerevisiae Blast evalue|  |  |  |  |  |  |  |  |  |  |  |  |  |  |  |  |  |  |  |  |  |  |  |  |  |  |  |  |  |  |  |  |  | | --- | --- | --- | --- | --- | --- | --- | --- | --- | --- | --- | --- | --- | --- | --- | --- | --- | --- | --- | --- | --- | --- | --- | --- | --- | --- | --- | --- | --- | --- | --- | --- | --- | | PF14\_0254 ? DNA mismatch repair protein Msh2p, putative  damaged DNA binding ? ATP binding (IEA) ? DNA metabolic process (IEA) ? mismatch repair ?  YOL090W ? nuclear chromosome ? four-way junction DNA binding ? Y-form DNA binding ? loop DNA binding ? double-strand/single-strand DNA junction binding ? meiotic mismatch repair ? removal of nonhomologous ends ? ATP binding ? nucleus ? mismatch repair ? DNA recombination ? meiotic gene conversion ? mitotic recombination ? ATPase activity ? chromatin silencing at silent mating-type cassette ? DNA insertion or deletion binding ? guanine/thymine mispair binding ? single base insertion or deletion binding ?  0  BLAST| PF13\_0328 ? proliferating cell nuclear antigen  DNA binding (IEA) ? nucleus (IEA) ? DNA replication factor C complex (IEA) ? regulation of DNA replication (IEA) ? DNA polymerase processivity factor activity (IEA) ? PCNA complex (IEA) ?  YBR088C ? nucleus ? replication fork ? leading strand elongation ? lagging strand elongation ? mutagenesis ? base-excision repair ? nucleotide-excision repair ? mismatch repair ? postreplication repair ? chromatin silencing at telomere ? DNA polymerase processivity factor activity ? chromatin silencing at silent mating-type cassette ? PCNA complex ?  2e-37  BLAST| PFE0270c ? DNA repair protein, putative  damaged DNA binding ? ATP binding (IEA) ? DNA metabolic process (IEA) ? DNA repair ? mismatch repair (IEA) ?  YDR097C ? four-way junction DNA binding ? meiotic mismatch repair ? ATP binding ? nucleus ? mismatch repair ? ATPase activity ? guanine/thymine mispair binding ? single base insertion or deletion binding ?  0  BLAST| PF08\_0125 ? tubulin gamma chain  microtubule cytoskeleton organization and biogenesis ? GTPase activity (IEA) ? structural molecule activity (IEA) ? structural constituent of cytoskeleton ? GTP binding (IEA) ? microtubule organizing center ? microtubule (IEA) ? microtubule-based movement (IEA) ? protein complex (IEA) ? protein polymerization (IEA) ?  YLR212C ? gamma-tubulin small complex, spindle pole body ? structural constituent of cytoskeleton ? inner plaque of spindle pole body ? outer plaque of spindle pole body ? microtubule nucleation ? mitotic spindle organization and biogenesis in nucleus ?  0  BLAST| PFI0235w ? replication factor A-related protein, putative  nucleic acid binding (IEA) ?  YAR007C ? double-strand break repair via homologous recombination ? chromosome, telomeric region ? damaged DNA binding ? single-stranded DNA binding ? nucleus ? DNA replication factor A complex ? cytoplasm ? DNA unwinding during replication ? DNA replication, synthesis of RNA primer ? DNA strand elongation during DNA replication ? nucleotide-excision repair ? postreplication repair ? DNA recombination ?  1e-28  BLAST| PFI0530c ? DNA primase, large subunit, putative  DNA primase activity (IEA) ? alpha DNA polymerase:primase complex (IEA) ? DNA replication, synthesis of RNA primer (IEA) ?  YKL045W ? DNA synthesis during DNA repair ? DNA primase activity ? nucleus ? nuclear envelope ? alpha DNA polymerase:primase complex ? DNA replication ? DNA replication, synthesis of RNA primer ? DNA replication initiation ? lagging strand elongation ?  9.80909e-45  BLAST| PFL2330w ? hypothetical protein  membrane ? apicoplast ?  YCL024W ? septin checkpoint ? septin ring assembly ? protein kinase activity ? cellular bud neck ? protein amino acid phosphorylation ? budding cell bud growth ? axial cellular bud site selection ?  0.28  BLAST| PFD0470c ? replication factor a protein, putative  nucleic acid binding (IEA) ? DNA replication factor A complex ? DNA replication ? DNA-dependent DNA replication ?  YAR007C ? double-strand break repair via homologous recombination ? chromosome, telomeric region ? damaged DNA binding ? single-stranded DNA binding ? nucleus ? DNA replication factor A complex ? cytoplasm ? DNA unwinding during replication ? DNA replication, synthesis of RNA primer ? DNA strand elongation during DNA replication ? nucleotide-excision repair ? postreplication repair ? DNA recombination ?  9.94922e-44  BLAST| PF14\_0680 ? hypothetical protein  membrane ?  YGR221C ? cellular bud tip ? cellular bud neck ? budding cell bud growth ?  3.2  BLAST| PF11\_0249 ? hypothetical protein  ATP binding (IEA) ? chromosome (IEA) ? chromosome organization and biogenesis (IEA) ?  YOL034W ? nucleus ? DNA repair ? cell proliferation ? Smc5-Smc6 complex ?  1e-14  BLAST| PFE1255w ? hypothetical protein  ATP binding (IEA) ? chromosome (IEA) ? chromosome organization and biogenesis (IEA) ?  YLR383W ? nucleus ? mitochondrion ? DNA repair ? cell proliferation ? Smc5-Smc6 complex ?  3e-11  BLAST | | | | | | | | | | | | | | | | | | | | | | | | | | | | | | | | | | | |

## Cluster Pair #1: 13 gene pairs.

|  |  |  |  |  |  |  |  |  |  |  |  |  |  |  |  |  |  |  |  |  |  |  |  |  |  |  |  |  |  |  |  |  |  |  |  |  |  |  |  |  |  |
| --- | --- | --- | --- | --- | --- | --- | --- | --- | --- | --- | --- | --- | --- | --- | --- | --- | --- | --- | --- | --- | --- | --- | --- | --- | --- | --- | --- | --- | --- | --- | --- | --- | --- | --- | --- | --- | --- | --- | --- | --- | --- |
| P. falciparum S. cerevisiae Blast evalue|  |  |  |  |  |  |  |  |  |  |  |  |  |  |  |  |  |  |  |  |  |  |  |  |  |  |  |  |  |  |  |  |  |  |  |  |  |  |  | | --- | --- | --- | --- | --- | --- | --- | --- | --- | --- | --- | --- | --- | --- | --- | --- | --- | --- | --- | --- | --- | --- | --- | --- | --- | --- | --- | --- | --- | --- | --- | --- | --- | --- | --- | --- | --- | --- | --- | | PFF1070c ? hypothetical protein, conserved  catalytic activity (IEA) ? iron ion binding (IEA) ?  YBR142W ? ribosomal large subunit assembly and maintenance ? ATP-dependent RNA helicase activity ? nucleolus ? rRNA processing ? ribosome biogenesis and assembly ?  2.8  BLAST| PF14\_0473 ? 3%27-5%27 exonuclease, putative  nucleic acid binding (IEA) ? intracellular (IEA) ? 3'-5' exonuclease activity (IEA) ?  YOR001W ? 3'-5'-exoribonuclease activity ? nuclear exosome (RNase complex) ? mRNA catabolic process ? ribosome biogenesis and assembly ? polyadenylation-dependent ncRNA catabolic process ?  1e-12  BLAST| MAL13P1.93 ? hypothetical protein   YPR144C ? nucleus ? nucleolus ? small nucleolar ribonucleoprotein complex ? Noc4p-Nop14p complex ? ribosome biogenesis and assembly ? ribosomal small subunit biogenesis and assembly ?  0.009  BLAST| PF11\_0353 ? hypothetical protein   YNL227C ? ATPase activator activity ? cytoplasm ? mitochondrion ? cytosol ? endocytosis ? regulation of cell size ? Hsp70/Hsc70 protein regulator activity ? ribosomal large subunit biogenesis and assembly ?  0.038  BLAST| PFE1240w ? hypothetical protein, conserved  catalytic activity (IEA) ? iron ion binding (IEA) ?  YPL207W ? endoplasmic reticulum ?  0  BLAST| PFF1500c ? DEAD%2FDEAH box ATP-dependent RNA helicase, putative  nucleic acid binding (IEA) ? ATP-dependent RNA helicase activity ? helicase activity (IEA) ? ATP binding (IEA) ? ATP-dependent helicase activity (IEA) ? RNA metabolic process ?  YMR290C ? RNA binding ? ATP-dependent RNA helicase activity ? nuclear envelope ? nucleolus ? rRNA processing ? RNA-dependent ATPase activity ? ribosome biogenesis and assembly ?  0  BLAST| PF13\_0109 ? N2,N2-dimethylguanosine tRNA methyltransferase, putative  RNA binding (IEA) ? tRNA (guanine-N2-)-methyltransferase activity ? tRNA modification ? tRNA processing (IEA) ? apicoplast ?  YDR120C ? tRNA (guanine-N2-)-methyltransferase activity ? nuclear envelope ? nuclear inner membrane ? mitochondrion ? tRNA methylation ? ribosome biogenesis and assembly ?  3e-13  BLAST| PF07\_0121 ? hypothetical protein, conserved   YHR170W ? ribosomal large subunit assembly and maintenance ? ribosomal large subunit export from nucleus ? RNA binding ? protein binding ? cytosol ? ribosome biogenesis and assembly ?  2.94273e-44  BLAST| PF10\_0200 ? hypothetical protein, conserved   YNL132W ? nucleolus ? ribosome biogenesis and assembly ?  2e-39  BLAST| PF14\_0185 ? ATP-dependent RNA helicase, putative  nucleic acid binding (IEA) ? ATP-dependent RNA helicase activity ? helicase activity (IEA) ? ATP binding (IEA) ? ATP-dependent helicase activity (IEA) ? also with YHR065C, clust.pair #2 YMR290C ? RNA binding ? ATP-dependent RNA helicase activity ? nuclear envelope ? nucleolus ? rRNA processing ? RNA-dependent ATPase activity ? ribosome biogenesis and assembly ?  5e-10  BLAST| PF13\_0177 ? ATP-dependent RNA helicase, putative  nucleic acid binding (IEA) ? helicase activity (IEA) ? ATP binding (IEA) ? ATP-dependent helicase activity (IEA) ?  YHR169W ? ATP-dependent RNA helicase activity ? nucleolus ? ribosome biogenesis and assembly ?  0  BLAST| PF14\_0055 ? hypothetical protein, conserved   YMR049C ? nucleus ? nucleolus ? rRNA processing ?  2e-24  BLAST| PF10\_0278 ? hypothetical protein, conserved   YKR081C ? ribosomal large subunit assembly and maintenance ? nucleolus ? 5S rRNA binding ? 7S RNA binding ? rRNA binding ? processing of 27S pre-rRNA ? ribosome biogenesis and assembly ?  2e-05  BLAST | | | | | | | | | | | | | | | | | | | | | | | | | | | | | | | | | | | | | | | | | |

## Cluster Pair #2: 11 gene pairs.

|  |  |  |  |  |  |  |  |  |  |  |  |  |  |  |  |  |  |  |  |  |  |  |  |  |  |  |  |  |  |  |  |  |  |  |  |
| --- | --- | --- | --- | --- | --- | --- | --- | --- | --- | --- | --- | --- | --- | --- | --- | --- | --- | --- | --- | --- | --- | --- | --- | --- | --- | --- | --- | --- | --- | --- | --- | --- | --- | --- | --- |
| P. falciparum S. cerevisiae Blast evalue|  |  |  |  |  |  |  |  |  |  |  |  |  |  |  |  |  |  |  |  |  |  |  |  |  |  |  |  |  |  |  |  |  | | --- | --- | --- | --- | --- | --- | --- | --- | --- | --- | --- | --- | --- | --- | --- | --- | --- | --- | --- | --- | --- | --- | --- | --- | --- | --- | --- | --- | --- | --- | --- | --- | --- | | PFB0860c ? RNA helicase, putative  nucleic acid binding (IEA) ? ATP-dependent RNA helicase activity ? helicase activity (IEA) ? ATP binding (IEA) ? ATP-dependent helicase activity (IEA) ?  YHR065C ? ATP-dependent RNA helicase activity ? nucleolus ? ribosome biogenesis and assembly ?  0  BLAST| PF11\_0471 ? hypothetical protein   YCR072C ? ribosomal large subunit assembly and maintenance ? nucleolus ? ribosome ? ribosome biogenesis and assembly ?  2.8026e-45  BLAST| PF14\_0185 ? ATP-dependent RNA helicase, putative  nucleic acid binding (IEA) ? ATP-dependent RNA helicase activity ? helicase activity (IEA) ? ATP binding (IEA) ? ATP-dependent helicase activity (IEA) ? also with YMR290C, clust.pair #1 YHR065C ? ATP-dependent RNA helicase activity ? nucleolus ? ribosome biogenesis and assembly ?  1e-09  BLAST| PF11\_0090 ? hypothetical protein  intracellular (IEA) ? nucleolus (IEA) ? cell proliferation (IEA) ?  YGR103W ? nucleus ? nucleolus ? cell cycle ? cell proliferation ? maturation of SSU-rRNA ? ribosome biogenesis and assembly ? ribosomal large subunit biogenesis and assembly ?  0  BLAST| MAL8P1.19 ? hypothetical protein, conserved  nucleic acid binding (IEA) ? helicase activity (IEA) ? ATP binding (IEA) ? ATP-dependent helicase activity (IEA) ? apicoplast ?  YLL008W ? ribosomal large subunit assembly and maintenance ? ATP-dependent RNA helicase activity ? nucleolus ? ribosome biogenesis and assembly ?  7e-32  BLAST| MAL8P1.92 ? ATPase, putative  nucleotide binding (IEA) ? ATP binding (IEA) ? ATPase activity ? nucleoside-triphosphatase activity (IEA) ?  YLL034C ? ribosomal large subunit export from nucleus ? nucleus ? nucleolus ? ATPase activity ?  0  BLAST| PF14\_0661 ? hypothetical protein, conserved  nucleic acid binding (IEA) ?  YOR145C ? nucleus ? nucleolus ? rRNA processing ? protein complex assembly ? ribosome biogenesis and assembly ? unfolded protein binding ?  0  BLAST| PF14\_0183 ? RNA helicase, putative  nucleic acid binding (IEA) ? ATP-dependent RNA helicase activity ? helicase activity (IEA) ? ATP binding (IEA) ? ATP-dependent helicase activity (IEA) ?  YJL033W ? ATP-dependent RNA helicase activity ? nucleolus ? ribosome biogenesis and assembly ?  0  BLAST| PF14\_0072 ? hypothetical protein, conserved   YNR046W ? tRNA (guanine-N2-)-methyltransferase activity ? nucleus ? nucleolus ? cytoplasm ? zinc ion binding ? tRNA methylation ?  2e-09  BLAST| PFI0635c ? hypothetical protein   YML093W ? small nucleolar ribonucleoprotein complex ? maturation of SSU-rRNA ? snoRNA binding ? ribosome biogenesis and assembly ?  0.01  BLAST| MAL13P1.334 ? hypothetical protein   YJR002W ? nucleus ? nucleolus ? small nucleolar ribonucleoprotein complex ? maturation of SSU-rRNA ? small subunit processome ? ribosome biogenesis and assembly ?  0.082  BLAST | | | | | | | | | | | | | | | | | | | | | | | | | | | | | | | | | | | |

## Cluster Pair #3: 4 gene pairs.

|  |  |  |  |  |  |  |  |  |  |  |  |  |  |  |
| --- | --- | --- | --- | --- | --- | --- | --- | --- | --- | --- | --- | --- | --- | --- |
| P. falciparum S. cerevisiae Blast evalue|  |  |  |  |  |  |  |  |  |  |  |  | | --- | --- | --- | --- | --- | --- | --- | --- | --- | --- | --- | --- | | PF11\_0189 ? hypothetical protein   YOL098C ? cytoplasm ?  6.02558e-44  BLAST| PF11\_0225 ? PfGCN20  nucleotide binding (IEA) ? ATP binding ? regulation of translation ? transport ? membrane ? ATPase activity (IEA) ? nucleoside-triphosphatase activity (IEA) ? ATPase activity, coupled to transmembrane movement of substances ?  YFR009W ? cytosol ? regulation of translational elongation ? ATPase activity ?  0  BLAST| PF13\_0261 ? ATP binding protein, putative  ATP binding ?  YLR243W ? signal sequence binding ?  2e-24  BLAST| PFE1320w ? hypothetical protein  intracellular (IEA) ? RNA processing (IEA) ?  YMR061W ? RNA binding ? nucleus ? mitochondrion ? mRNA cleavage factor complex ? mRNA polyadenylation ? mRNA cleavage ? protein heterodimerization activity ?  6e-05  BLAST | | | | | | | | | | | | | | |

## Cluster Pair #4: 11 gene pairs.

|  |  |  |  |  |  |  |  |  |  |  |  |  |  |  |  |  |  |  |  |  |  |  |  |  |  |  |  |  |  |  |  |  |  |  |  |
| --- | --- | --- | --- | --- | --- | --- | --- | --- | --- | --- | --- | --- | --- | --- | --- | --- | --- | --- | --- | --- | --- | --- | --- | --- | --- | --- | --- | --- | --- | --- | --- | --- | --- | --- | --- |
| P. falciparum S. cerevisiae Blast evalue|  |  |  |  |  |  |  |  |  |  |  |  |  |  |  |  |  |  |  |  |  |  |  |  |  |  |  |  |  |  |  |  |  | | --- | --- | --- | --- | --- | --- | --- | --- | --- | --- | --- | --- | --- | --- | --- | --- | --- | --- | --- | --- | --- | --- | --- | --- | --- | --- | --- | --- | --- | --- | --- | --- | --- | | PF14\_0716 ? Proteosome subunit alpha type 1, putative  endopeptidase activity ? threonine endopeptidase activity (IEA) ? proteasome core complex ? ubiquitin-dependent protein catabolic process ?  YMR314W ? endopeptidase activity ? ubiquitin-dependent protein catabolic process ? proteasome core complex, alpha-subunit complex ?  3.00018e-42  BLAST| PF13\_0033 ? 26S proteasome regulatory subunit, putative  nucleotide binding (IEA) ? nucleic acid binding (IEA) ? endopeptidase activity ? ATP binding (IEA) ? nucleus (IEA) ? cytoplasm (IEA) ? proteasome regulatory particle ? ubiquitin-dependent protein catabolic process ? hydrolase activity (IEA) ? ATPase activity (IEA) ? nucleoside-triphosphatase activity (IEA) ? protein catabolic process (IEA) ?  YOR259C ? endopeptidase activity ? nucleus ? ubiquitin-dependent protein catabolic process ? proteasome regulatory particle, base subcomplex ? ATPase activity ?  0  BLAST| MAL8P1.142 ? proteasome beta-subunit  endopeptidase activity ? threonine endopeptidase activity (IEA) ? proteasome core complex ? ubiquitin-dependent protein catabolic process (IEA) ?  YFR050C ? endopeptidase activity ? ubiquitin-dependent protein catabolic process ? proteasome core complex, beta-subunit complex ?  5e-26  BLAST| PF13\_0063 ? 26S proteasome regulatory subunit 7, putative  nucleotide binding (IEA) ? endopeptidase activity ? ATP binding (IEA) ? nucleus (IEA) ? cytoplasm (IEA) ? proteasome regulatory particle ? ubiquitin-dependent protein catabolic process ? hydrolase activity (IEA) ? ATPase activity (IEA) ? nucleoside-triphosphatase activity (IEA) ? protein catabolic process (IEA) ?  YKL145W ? endopeptidase activity ? ubiquitin-dependent protein catabolic process ? proteasome regulatory particle, base subcomplex ? ATPase activity ?  0  BLAST| PF14\_0025 ? proteosome subunit, putative  endopeptidase activity ? proteasome regulatory particle ? proteolysis ?  YDL097C ? structural molecule activity ? ubiquitin-dependent protein catabolic process ? proteasome regulatory particle, lid subcomplex ?  8e-21  BLAST| PF07\_0113 ? hypothetical protein  apicoplast ?  YJL036W ? pre-autophagosomal structure ? cytoplasm ? protein targeting to vacuole ? lipid binding ? phosphatidylinositol 3-phosphate binding ?  0.008  BLAST| MAL13P1.270 ? proteasome subunit, putative  endopeptidase activity ? threonine endopeptidase activity (IEA) ? proteasome core complex ? ubiquitin-dependent protein catabolic process ?  YOL038W ? endopeptidase activity ? mitochondrion ? cytosol ? ubiquitin-dependent protein catabolic process ? proteasome core complex, alpha-subunit complex ?  0  BLAST| MAL13P1.190 ? proteasome regulatory component, putative  endopeptidase activity ? proteasome regulatory particle ? ubiquitin-dependent protein catabolic process ?  YER021W ? ubiquitin-dependent protein catabolic process ? proteasome regulatory particle, lid subcomplex ?  0  BLAST| PFC0520w ? 26S proteasome regulatory subunit S14, putative  proteasome regulatory particle ? ubiquitin-dependent protein catabolic process ?  YFR052W ? endopeptidase activity ? ubiquitin-dependent protein catabolic process ? proteasome regulatory particle, lid subcomplex ?  4e-14  BLAST| MAL8P1.128 ? proteasome subunit alpha, putative  endopeptidase activity ? threonine endopeptidase activity (IEA) ? proteasome core complex ? ubiquitin-dependent protein catabolic process ?  YGL011C ? endopeptidase activity ? mitochondrion ? ubiquitin-dependent protein catabolic process ? proteasome core complex, alpha-subunit complex ?  1e-34  BLAST| PF11\_0303 ? 26S proteasome regulatory complex subunit, putative  proteasome complex ? endopeptidase activity ? proteolysis ?  YPR108W ? structural molecule activity ? ubiquitin-dependent protein catabolic process ? proteasome regulatory particle, lid subcomplex ?  0  BLAST | | | | | | | | | | | | | | | | | | | | | | | | | | | | | | | | | | | |

## Cluster Pair #5: 12 gene pairs.

|  |  |  |  |  |  |  |  |  |  |  |  |  |  |  |  |  |  |  |  |  |  |  |  |  |  |  |  |  |  |  |  |  |  |  |  |  |  |  |
| --- | --- | --- | --- | --- | --- | --- | --- | --- | --- | --- | --- | --- | --- | --- | --- | --- | --- | --- | --- | --- | --- | --- | --- | --- | --- | --- | --- | --- | --- | --- | --- | --- | --- | --- | --- | --- | --- | --- |
| P. falciparum S. cerevisiae Blast evalue|  |  |  |  |  |  |  |  |  |  |  |  |  |  |  |  |  |  |  |  |  |  |  |  |  |  |  |  |  |  |  |  |  |  |  |  | | --- | --- | --- | --- | --- | --- | --- | --- | --- | --- | --- | --- | --- | --- | --- | --- | --- | --- | --- | --- | --- | --- | --- | --- | --- | --- | --- | --- | --- | --- | --- | --- | --- | --- | --- | --- | | PFF0940c ? cell division cycle protein 48 homologue, putative  nucleotide binding (IEA) ? ATP-dependent peptidase activity (IEA) ? serine-type endopeptidase activity (IEA) ? ATP binding (IEA) ? microsome ? proteolysis (IEA) ? hydrolase activity (IEA) ? ATPase activity ? nucleoside-triphosphatase activity (IEA) ?  YDL126C ? nucleus ? endoplasmic reticulum membrane ? microsome ? cytosol ? ubiquitin-dependent protein catabolic process ? vesicle fusion ? apoptosis ? cell cycle ? protein transport ? ATPase activity ? ER-associated protein catabolic process ?  0  BLAST| PF13\_0282 ? proteasome subunit, putative  endopeptidase activity ? threonine endopeptidase activity (IEA) ? proteasome core complex ? ubiquitin-dependent protein catabolic process ?  YGR135W ? endopeptidase activity ? ubiquitin-dependent protein catabolic process ? proteasome core complex, alpha-subunit complex ? filamentous growth ?  0  BLAST| PFE0915c ? proteasome subunit beta type 1  endopeptidase activity ? threonine endopeptidase activity (IEA) ? proteasome core complex ? ubiquitin-dependent protein catabolic process ?  YBL041W ? endopeptidase activity ? ubiquitin-dependent protein catabolic process ? proteasome core complex, beta-subunit complex ?  9e-35  BLAST| PF07\_0112 ? proteasome subunit alpha type 5, putative  endopeptidase activity ? threonine endopeptidase activity (IEA) ? proteasome core complex (IEA) ? ubiquitin-dependent protein catabolic process ? proteasome core complex, alpha-subunit complex ?  YGR253C ? endopeptidase activity ? ubiquitin-dependent protein catabolic process ? response to stress ? proteasome core complex, alpha-subunit complex ? ascospore formation ?  0  BLAST| PFE0150c ? 4-diphosphocytidyl-2c-methyl-D-erythritol kinase %28CMK%29, putative  ATP binding (IEA) ? isoprenoid biosynthetic process ? kinase activity (IEA) ? phosphorylation (IEA) ? apicoplast ? 4-(cytidine 5'-diphospho)-2-C-methyl-D-erythritol kinase activity ?  YLR149C ?  0.086  BLAST| PFL1300c ? hypothetical protein   YEL052W ? mitochondrion ? ATPase activity ?  0.23  BLAST| MAL13P1.343 ? proteasome regulatory subunit, putative  proteasome regulatory particle ? ubiquitin-dependent protein catabolic process ?  YFR004W ? endopeptidase activity ? nucleus ? ubiquitin-dependent protein catabolic process ? proteasome regulatory particle, lid subcomplex ?  0  BLAST| PFA0450c ? mRNA cleavage factor-like protein, putative   YNL173C ? pheromone-dependent signal transduction during conjugation with cellular fusion ? plasma membrane ?  0.017  BLAST| PF14\_0632 ? 26S proteasome subunit, putative  endopeptidase activity ? proteasome regulatory particle ? ubiquitin-dependent protein catabolic process ?  YIL075C ? endopeptidase activity ? nucleus ? ubiquitin-dependent protein catabolic process ? proteasome regulatory particle, base subcomplex ? protein binding, bridging ?  0  BLAST| PFL1245w ? ubiquitin-activating enzyme e1, putative  catalytic activity (IEA) ? ubiquitin activating enzyme activity ? protein modification process (IEA) ? ubiquitin cycle ? small protein activating enzyme activity (IEA) ?  YKL210W ? ubiquitin activating enzyme activity ? nucleus ? cytoplasm ? ubiquitin cycle ?  0  BLAST| PFA0400c ? beta3 proteasome subunit, putative  endopeptidase activity ? threonine endopeptidase activity (IEA) ? proteasome core complex ? ubiquitin-dependent protein catabolic process ?  YER094C ? endopeptidase activity ? ubiquitin-dependent protein catabolic process ? proteasome core complex, beta-subunit complex ?  0  BLAST| PF11\_0314 ? 26S protease subunit regulatory subunit 6a, putative  nucleotide binding (IEA) ? proteasome complex ? endopeptidase activity ? ATP binding (IEA) ? nucleus (IEA) ? cytoplasm (IEA) ? proteolysis ? hydrolase activity (IEA) ? ATPase activity ? nucleoside-triphosphatase activity (IEA) ? protein catabolic process (IEA) ?  YOR117W ? endopeptidase activity ? ubiquitin-dependent protein catabolic process ? proteasome regulatory particle, base subcomplex ? ATPase activity ?  0  BLAST | | | | | | | | | | | | | | | | | | | | | | | | | | | | | | | | | | | | | | |

## Cluster Pair #6: 6 gene pairs.

|  |  |  |  |  |  |  |  |  |  |  |  |  |  |  |  |  |  |  |  |  |
| --- | --- | --- | --- | --- | --- | --- | --- | --- | --- | --- | --- | --- | --- | --- | --- | --- | --- | --- | --- | --- |
| P. falciparum S. cerevisiae Blast evalue|  |  |  |  |  |  |  |  |  |  |  |  |  |  |  |  |  |  | | --- | --- | --- | --- | --- | --- | --- | --- | --- | --- | --- | --- | --- | --- | --- | --- | --- | --- | | PFB0175c ? hypothetical protein   YAL025C ? nucleolus ? processing of 27S pre-rRNA ? ribosome biogenesis and assembly ? ribosomal large subunit biogenesis and assembly ?  1e-25  BLAST| PF11\_0445 ? DNA-directed RNA polymerase I, putative  DNA binding (IEA) ? DNA-directed RNA polymerase activity (IEA) ? transcription (IEA) ? protein dimerization activity (IEA) ?  YPR110C ? DNA-directed RNA polymerase activity ? DNA-directed RNA polymerase III complex ? DNA-directed RNA polymerase I complex ? transcription from RNA polymerase I promoter ? transcription from RNA polymerase III promoter ? ribosome biogenesis and assembly ?  9.94922e-44  BLAST| PFE1310c ? hypothetical protein   YPR169W ? nucleus ? nucleolus ? protein monoubiquitination ?  0.0001  BLAST| PF13\_0136 ? hypothetical protein   YDR361C ? ribosomal large subunit export from nucleus ? nucleus ? cytoplasm ? protein export from nucleus ?  0.28  BLAST| PF07\_0027 ? DNA-directed RNA polymerase 2 8.2 kDa polypeptide, putative  DNA-directed RNA polymerase activity ? DNA-directed RNA polymerase II, core complex ? transcription (IEA) ? transcription from RNA polymerase II promoter ?  YOR210W ? DNA-directed RNA polymerase activity ? DNA-directed RNA polymerase II, core complex ? DNA-directed RNA polymerase III complex ? DNA-directed RNA polymerase I complex ? transcription from RNA polymerase I promoter ? transcription from RNA polymerase II promoter ? transcription from RNA polymerase III promoter ?  2e-17  BLAST| PFI0475w ? small nuclear ribonucleoprotein %28snRNP%29, putative  nucleus (IEA) ? small nucleolar ribonucleoprotein complex (IEA) ? mRNA processing (IEA) ? ribonucleoprotein complex (IEA) ?  YLR147C ? commitment complex ? nuclear mRNA splicing, via spliceosome ? mRNA binding ? snRNP U5 ? snRNP U1 ? RNA splicing factor activity, transesterification mechanism ? U4/U6 x U5 tri-snRNP complex ?  4e-16  BLAST | | | | | | | | | | | | | | | | | | | | |

## Cluster Pair #7: 18 gene pairs.

|  |  |  |  |  |  |  |  |  |  |  |  |  |  |  |  |  |  |  |  |  |  |  |  |  |  |  |  |  |  |  |  |  |  |  |  |  |  |  |  |  |  |  |  |  |  |  |  |  |  |  |  |  |  |  |  |  |
| --- | --- | --- | --- | --- | --- | --- | --- | --- | --- | --- | --- | --- | --- | --- | --- | --- | --- | --- | --- | --- | --- | --- | --- | --- | --- | --- | --- | --- | --- | --- | --- | --- | --- | --- | --- | --- | --- | --- | --- | --- | --- | --- | --- | --- | --- | --- | --- | --- | --- | --- | --- | --- | --- | --- | --- | --- |
| P. falciparum S. cerevisiae Blast evalue|  |  |  |  |  |  |  |  |  |  |  |  |  |  |  |  |  |  |  |  |  |  |  |  |  |  |  |  |  |  |  |  |  |  |  |  |  |  |  |  |  |  |  |  |  |  |  |  |  |  |  |  |  |  | | --- | --- | --- | --- | --- | --- | --- | --- | --- | --- | --- | --- | --- | --- | --- | --- | --- | --- | --- | --- | --- | --- | --- | --- | --- | --- | --- | --- | --- | --- | --- | --- | --- | --- | --- | --- | --- | --- | --- | --- | --- | --- | --- | --- | --- | --- | --- | --- | --- | --- | --- | --- | --- | --- | | MAL7P1.24 ? hypothetical protein, conserved  intracellular (IEA) ?  YER126C ? nucleus ? ribosome biogenesis and assembly ? ribosomal large subunit biogenesis and assembly ?  0  BLAST| PF14\_0429 ? RNA helicase, putative  nucleic acid binding (IEA) ? helicase activity ? ATP binding (IEA) ? ATP-dependent helicase activity (IEA) ? also with YMR290C, clust.pair #7 also with YLL008W, clust.pair #8 also with YJL033W, clust.pair #8 also with YHR065C, clust.pair #8 YHR169W ? ATP-dependent RNA helicase activity ? nucleolus ? ribosome biogenesis and assembly ?  3e-29  BLAST| PF14\_0429 ? RNA helicase, putative  nucleic acid binding (IEA) ? helicase activity ? ATP binding (IEA) ? ATP-dependent helicase activity (IEA) ? also with YHR169W, clust.pair #7 also with YLL008W, clust.pair #8 also with YJL033W, clust.pair #8 also with YHR065C, clust.pair #8 YMR290C ? RNA binding ? ATP-dependent RNA helicase activity ? nuclear envelope ? nucleolus ? rRNA processing ? RNA-dependent ATPase activity ? ribosome biogenesis and assembly ?  8e-29  BLAST| PF14\_0100 ? cytidine triphosphate synthetase  catalytic activity (IEA) ? CTP synthase activity ? pyrimidine base metabolic process ? pyrimidine nucleotide biosynthetic process (IEA) ?  YBL039C ? CTP synthase activity ? cytosol ? CTP biosynthetic process ? phospholipid biosynthetic process ? pyrimidine base biosynthetic process ?  0  BLAST| PF13\_0309 ? hypothetical protein   YLR409C ? nucleus ? nucleolus ? small nucleolar ribonucleoprotein complex ? snoRNA binding ? ribosome biogenesis and assembly ?  1e-17  BLAST| PFL1345c ? hypothetical protein, conserved  catalytic activity (IEA) ? iron ion binding (IEA) ? membrane ?  YPL086C ? histone acetyltransferase activity ? nucleus ? cytoplasm ? regulation of transcription from RNA polymerase II promoter ? tRNA modification ? transcription elongation factor complex ?  0  BLAST| PF08\_0065 ? hypothetical protein, conserved   YMR131C ? nucleolus ? ribosome biogenesis and assembly ?  5e-33  BLAST| PF14\_0436 ? helicase, truncated, putative  nucleic acid binding (IEA) ? helicase activity ? ATP binding (IEA) ? ATP-dependent helicase activity (IEA) ?  YNL112W ? mRNA catabolic process, nonsense-mediated decay ? RNA helicase activity ? nucleus ? cytoplasm ? mitochondrion ?  0  BLAST| PF13\_0310 ? hypothetical protein   YDL153C ? nucleus ? small nucleolar ribonucleoprotein complex ? establishment and/or maintenance of chromatin architecture ? maturation of SSU-rRNA ? snoRNA binding ? ribosome biogenesis and assembly ?  2e-06  BLAST| PF14\_0221 ? hypothetical protein, conserved  GTP binding (IEA) ?  YNR053C ? ribosomal large subunit export from nucleus ? GTPase activity ? nucleus ? nucleoplasm ? nucleolus ? ribosome biogenesis and assembly ? ribosome assembly ?  0  BLAST| PF10\_0054 ? hypothetical protein   YIL091C ? RNA helicase activity ? nucleus ? nucleolus ?  2e-19  BLAST| PF11\_0275 ? hypothetical protein   YLR409C ? nucleus ? nucleolus ? small nucleolar ribonucleoprotein complex ? snoRNA binding ? ribosome biogenesis and assembly ?  0.083  BLAST| PFE1390w ? RNA helicase-1  nucleic acid binding (IEA) ? ATP-dependent RNA helicase activity ? helicase activity (IEA) ? ATP binding (IEA) ? ATP-dependent helicase activity (IEA) ?  YNL112W ? mRNA catabolic process, nonsense-mediated decay ? RNA helicase activity ? nucleus ? cytoplasm ? mitochondrion ?  0  BLAST| PF14\_0494 ? hypothetical protein, conserved  mitochondrion ?  YDL060W ? nucleolus ? cytoplasm ? rRNA processing ? ribosome biogenesis and assembly ? ribonucleoprotein binding ?  2e-18  BLAST| PFL1175w ? hypothetical protein   YJL069C ? nucleolus ? small nucleolar ribonucleoprotein complex ? ribosome biogenesis and assembly ?  7e-05  BLAST| PF10\_0197 ? hypothetical protein  apicoplast ?  YNL061W ? nucleolus ? rRNA processing ? RNA methyltransferase activity ? S-adenosylmethionine-dependent methyltransferase activity ? ribosome biogenesis and assembly ?  1e-06  BLAST| MAL7P1.113 ? DEAD box helicase, putative  nucleic acid binding (IEA) ? RNA binding ? ATP-dependent RNA helicase activity ? helicase activity (IEA) ? ATP binding ? ATP-dependent helicase activity (IEA) ? RNA metabolic process ? also with YMR290C, clust.pair #7 YKR024C ? ribosomal large subunit assembly and maintenance ? ATP-dependent RNA helicase activity ? nucleolus ? ribosome biogenesis and assembly ?  5e-26  BLAST| MAL7P1.113 ? DEAD box helicase, putative  nucleic acid binding (IEA) ? RNA binding ? ATP-dependent RNA helicase activity ? helicase activity (IEA) ? ATP binding ? ATP-dependent helicase activity (IEA) ? RNA metabolic process ? also with YKR024C, clust.pair #7 YMR290C ? RNA binding ? ATP-dependent RNA helicase activity ? nuclear envelope ? nucleolus ? rRNA processing ? RNA-dependent ATPase activity ? ribosome biogenesis and assembly ?  1e-24  BLAST | | | | | | | | | | | | | | | | | | | | | | | | | | | | | | | | | | | | | | | | | | | | | | | | | | | | | | | | |

## Cluster Pair #8: 17 gene pairs.

|  |  |  |  |  |  |  |  |  |  |  |  |  |  |  |  |  |  |  |  |  |  |  |  |  |  |  |  |  |  |  |  |  |  |  |  |  |  |  |  |  |  |  |  |  |  |  |  |  |  |  |  |  |  |
| --- | --- | --- | --- | --- | --- | --- | --- | --- | --- | --- | --- | --- | --- | --- | --- | --- | --- | --- | --- | --- | --- | --- | --- | --- | --- | --- | --- | --- | --- | --- | --- | --- | --- | --- | --- | --- | --- | --- | --- | --- | --- | --- | --- | --- | --- | --- | --- | --- | --- | --- | --- | --- | --- |
| P. falciparum S. cerevisiae Blast evalue|  |  |  |  |  |  |  |  |  |  |  |  |  |  |  |  |  |  |  |  |  |  |  |  |  |  |  |  |  |  |  |  |  |  |  |  |  |  |  |  |  |  |  |  |  |  |  |  |  |  |  | | --- | --- | --- | --- | --- | --- | --- | --- | --- | --- | --- | --- | --- | --- | --- | --- | --- | --- | --- | --- | --- | --- | --- | --- | --- | --- | --- | --- | --- | --- | --- | --- | --- | --- | --- | --- | --- | --- | --- | --- | --- | --- | --- | --- | --- | --- | --- | --- | --- | --- | --- | | PF07\_0067 ? hypothetical protein   YGR245C ? nucleus ? traversing start control point of mitotic cell cycle ? actin cytoskeleton organization and biogenesis ? ribosome biogenesis and assembly ? ribosome assembly ?  1e-09  BLAST| PFL2475w ? DEAD%2FDEAH box helicase, putative  nucleic acid binding (IEA) ? ATP-dependent RNA helicase activity ? helicase activity (IEA) ? ATP binding (IEA) ? ATP-dependent helicase activity (IEA) ? RNA metabolic process ?  YLL008W ? ribosomal large subunit assembly and maintenance ? ATP-dependent RNA helicase activity ? nucleolus ? ribosome biogenesis and assembly ?  0  BLAST| PF14\_0068 ? fibrillarin, putative  RNA binding (IEA) ? nucleus (IEA) ? small nucleolar ribonucleoprotein complex ? mitochondrion ? rRNA processing ?  YDL014W ? ribosomal large subunit assembly and maintenance ? rRNA modification ? RNA methylation ? nucleolus ? small nucleolar ribonucleoprotein complex ? ribosome ? methyltransferase activity ? maturation of SSU-rRNA ? snoRNA 3'-end processing ? ribosome biogenesis and assembly ?  0  BLAST| PF14\_0429 ? RNA helicase, putative  nucleic acid binding (IEA) ? helicase activity ? ATP binding (IEA) ? ATP-dependent helicase activity (IEA) ? also with YHR169W, clust.pair #7 also with YMR290C, clust.pair #7 also with YJL033W, clust.pair #8 also with YHR065C, clust.pair #8 YLL008W ? ribosomal large subunit assembly and maintenance ? ATP-dependent RNA helicase activity ? nucleolus ? ribosome biogenesis and assembly ?  4e-30  BLAST| PF14\_0429 ? RNA helicase, putative  nucleic acid binding (IEA) ? helicase activity ? ATP binding (IEA) ? ATP-dependent helicase activity (IEA) ? also with YHR169W, clust.pair #7 also with YMR290C, clust.pair #7 also with YLL008W, clust.pair #8 also with YHR065C, clust.pair #8 YJL033W ? ATP-dependent RNA helicase activity ? nucleolus ? ribosome biogenesis and assembly ?  2e-29  BLAST| PF14\_0429 ? RNA helicase, putative  nucleic acid binding (IEA) ? helicase activity ? ATP binding (IEA) ? ATP-dependent helicase activity (IEA) ? also with YHR169W, clust.pair #7 also with YMR290C, clust.pair #7 also with YLL008W, clust.pair #8 also with YJL033W, clust.pair #8 YHR065C ? ATP-dependent RNA helicase activity ? nucleolus ? ribosome biogenesis and assembly ?  1e-28  BLAST| PFF1030w ? hypothetical protein, conserved  RNA binding (IEA) ?  YDR496C ? mRNA binding ? nucleus ? nucleolus ? regulation of transcription, mating-type specific ? specific transcriptional repressor activity ? ribosome biogenesis and assembly ?  0.0006  BLAST| PF14\_0456 ? hypothetical protein, conserved   YLR129W ? small nucleolar ribonucleoprotein complex ? maturation of SSU-rRNA ? snoRNA binding ? ribosome biogenesis and assembly ?  5.04467e-44  BLAST| PFE1115c ? s-adenosylmethionine-dependent methyltransferase, putative  S-adenosylmethionine-dependent methyltransferase activity ?  YCR047C ? cellular bud site selection ? nucleus ? cytoplasm ? S-adenosylmethionine-dependent methyltransferase activity ?  0  BLAST| PFI1235w ? hypothetical protein   YDR083W ? telomere maintenance ? nucleolus ? rRNA processing ? methyltransferase activity ? ribosome biogenesis and assembly ?  6e-24  BLAST| PF14\_0292 ? hypothetical protein, conserved  GTP binding (IEA) ?  YGL099W ? ribosome export from nucleus ? conjugation with cellular fusion ? GTPase activity ? cytoplasm ? ascospore formation ? ribosome biogenesis and assembly ?  2e-30  BLAST| PF14\_0174 ? hypothetical protein, conserved  RNA binding (IEA) ? pseudouridylate synthase activity (IEA) ? RNA processing (IEA) ?  YLR175W ? pseudouridylate synthase activity ? nucleolus ? rRNA pseudouridine synthesis ? box H/ACA snoRNP complex ? ribosome biogenesis and assembly ?  0  BLAST| MAL13P1.341 ? hypothetical protein, conserved   YKL009W ? telomere maintenance ? nucleus ? nucleolus ? rRNA processing ? mRNA catabolic process ? ribosome biogenesis and assembly ? ribosomal large subunit biogenesis and assembly ?  4e-06  BLAST| MAL13P1.14 ? ATP-dependent DEAD box helicase, putative  nucleic acid binding (IEA) ? ATP-dependent RNA helicase activity ? helicase activity (IEA) ? ATP binding (IEA) ? ATP-dependent helicase activity (IEA) ?  YMR128W ? RNA helicase activity ? nucleolus ? small nucleolar ribonucleoprotein complex ? mitochondrion ? maturation of SSU-rRNA ? ribosome biogenesis and assembly ?  9.94922e-44  BLAST| PF14\_0150 ? RNA polymerase small subunit, putative  DNA binding (IEA) ? DNA-directed RNA polymerase activity ? transcription ? protein dimerization activity (IEA) ?  YNL113W ? DNA-directed RNA polymerase activity ? DNA-directed RNA polymerase III complex ? DNA-directed RNA polymerase I complex ? transcription from RNA polymerase I promoter ? transcription from RNA polymerase III promoter ? ribosome biogenesis and assembly ?  1e-18  BLAST| PFL2010c ? DEAD%2FDEAH box helicase, putative  nucleic acid binding (IEA) ? ATP-dependent RNA helicase activity ? helicase activity (IEA) ? ATP binding (IEA) ? ATP-dependent helicase activity (IEA) ? RNA metabolic process ?  YLR276C ? ribosomal large subunit assembly and maintenance ? ATP-dependent RNA helicase activity ? nucleolus ? ribosome biogenesis and assembly ?  3e-25  BLAST| PF14\_0156 ? dimethyladenosine transferase, putative  rRNA modification ? rRNA (adenine-N6,N6-)-dimethyltransferase activity (IEA) ? mitochondrion ? rRNA processing (IEA) ? rRNA methyltransferase activity ? S-adenosylmethionine-dependent methyltransferase activity (IEA) ? rRNA (adenine) methyltransferase activity (IEA) ?  YPL266W ? rRNA modification ? rRNA (adenine-N6,N6-)-dimethyltransferase activity ? nucleolus ? ribosome biogenesis and assembly ?  0  BLAST | | | | | | | | | | | | | | | | | | | | | | | | | | | | | | | | | | | | | | | | | | | | | | | | | | | | | |

## Cluster Pair #9: 5 gene pairs.

|  |  |  |  |  |  |  |  |  |  |  |  |  |  |  |  |  |  |
| --- | --- | --- | --- | --- | --- | --- | --- | --- | --- | --- | --- | --- | --- | --- | --- | --- | --- |
| P. falciparum S. cerevisiae Blast evalue|  |  |  |  |  |  |  |  |  |  |  |  |  |  |  | | --- | --- | --- | --- | --- | --- | --- | --- | --- | --- | --- | --- | --- | --- | --- | | PF14\_0282 ? hypothetical protein, conserved  acid phosphatase activity (IEA) ?  YLR410W ? cytoplasm ? actin cytoskeleton organization and biogenesis ?  0  BLAST| PF07\_0053 ? hypothetical protein  metalloendopeptidase activity (IEA) ? proteolysis (IEA) ? pathogenesis (IEA) ? metal ion binding (IEA) ?  YDL035C ? replicative cell aging ? G-protein coupled receptor activity ? plasma membrane ? pseudohyphal growth ? G-protein coupled receptor protein signaling pathway ?  5e-06  BLAST| PF10\_0314 ? asparagine-rich antigen   YDL035C ? replicative cell aging ? G-protein coupled receptor activity ? plasma membrane ? pseudohyphal growth ? G-protein coupled receptor protein signaling pathway ?  0.004  BLAST| PF14\_0127 ? N-myristoyltransferase  glycylpeptide N-tetradecanoyltransferase activity ? N-terminal protein myristoylation ?  YLR195C ? glycylpeptide N-tetradecanoyltransferase activity ? cytosol ? N-terminal peptidyl-glycine N-myristoylation ?  0  BLAST| PFF0320c ? polypyrimidine tract binding protein, putative  nucleic acid binding (IEA) ? poly-pyrimidine tract binding ? RNA splicing ?  YOR319W ? nuclear mRNA splicing, via spliceosome ? RNA binding ? snRNP U2 ?  5e-05  BLAST | | | | | | | | | | | | | | | | | |

## Cluster Pair #10: 6 gene pairs.

|  |  |  |  |  |  |  |  |  |  |  |  |  |  |  |  |  |  |  |  |  |
| --- | --- | --- | --- | --- | --- | --- | --- | --- | --- | --- | --- | --- | --- | --- | --- | --- | --- | --- | --- | --- |
| P. falciparum S. cerevisiae Blast evalue|  |  |  |  |  |  |  |  |  |  |  |  |  |  |  |  |  |  | | --- | --- | --- | --- | --- | --- | --- | --- | --- | --- | --- | --- | --- | --- | --- | --- | --- | --- | | PF14\_0177 ? DNA replication licensing factor MCM2  nucleotide binding (IEA) ? nucleic acid binding (IEA) ? DNA binding (IEA) ? ATP binding (IEA) ? nucleus (IEA) ? DNA replication ? DNA replication initiation (IEA) ? DNA-dependent ATPase activity (IEA) ? photosynthesis (IEA) ? chlorophyll biosynthetic process (IEA) ? magnesium chelatase activity (IEA) ? nucleoside-triphosphatase activity (IEA) ?  YBL023C ? chromatin binding ? ATP-dependent DNA helicase activity ? nucleus ? pre-replicative complex ? cytoplasm ? pre-replicative complex assembly ? DNA unwinding during replication ? DNA replication initiation ? MCM complex ?  0  BLAST| PFL0580w ? DNA replication licensing factor mcm5, putative  nucleic acid binding (IEA) ? DNA binding (IEA) ? ATP-dependent DNA helicase activity ? ATP binding ? nucleus (IEA) ? pre-replicative complex ? DNA replication initiation ? DNA strand elongation during DNA replication ? DNA-dependent ATPase activity ?  YLR274W ? chromatin binding ? ATP-dependent DNA helicase activity ? pre-replicative complex ? replication fork ? cytoplasm ? pre-replicative complex assembly ? DNA unwinding during replication ? DNA replication initiation ? establishment of chromatin silencing ? MCM complex ?  0  BLAST| PF13\_0095 ? DNA replication licensing factor mcm4-related  nucleic acid binding (IEA) ? DNA binding (IEA) ? ATP binding (IEA) ? nucleus (IEA) ? DNA unwinding during replication ? DNA replication initiation ? DNA-dependent ATPase activity (IEA) ?  YPR019W ? chromatin binding ? ATP-dependent DNA helicase activity ? nucleus ? pre-replicative complex ? cytoplasm ? pre-replicative complex assembly ? DNA unwinding during replication ? DNA replication initiation ? MCM complex ?  0  BLAST| PFE1345c ? minichromosome maintenance protein 3, putative  nucleotide binding (IEA) ? nucleic acid binding (IEA) ? DNA binding (IEA) ? ATP binding (IEA) ? nucleus (IEA) ? DNA replication initiation (IEA) ? DNA-dependent ATPase activity (IEA) ? photosynthesis (IEA) ? chlorophyll biosynthetic process (IEA) ? magnesium chelatase activity (IEA) ? nucleoside-triphosphatase activity (IEA) ?  YEL032W ? chromatin binding ? ATP-dependent DNA helicase activity ? nucleus ? pre-replicative complex ? cytoplasm ? pre-replicative complex assembly ? DNA unwinding during replication ? DNA replication initiation ? MCM complex ?  0  BLAST| PFL1370w ? NIMA-related protein kinase %28Pfnek-1%29  protein kinase activity (IEA) ? protein serine/threonine kinase activity ? protein-tyrosine kinase activity (IEA) ? ATP binding (IEA) ? protein amino acid phosphorylation ? mitosis ?  YAR018C ? protein kinase activity ? chromosome segregation ?  1e-38  BLAST| PF07\_0023 ? DNA replication licensing factor mcm7 homologue, putative  nucleotide binding (IEA) ? nucleic acid binding (IEA) ? DNA binding (IEA) ? ATP binding (IEA) ? nucleus (IEA) ? DNA replication initiation ? DNA-dependent ATPase activity ? photosynthesis (IEA) ? chlorophyll biosynthetic process (IEA) ? magnesium chelatase activity (IEA) ? ATPase activity (IEA) ? nucleoside-triphosphatase activity (IEA) ?  YBR202W ? chromatin binding ? ATP-dependent DNA helicase activity ? ATP binding ? nucleus ? pre-replicative complex ? cytoplasm ? pre-replicative complex assembly ? DNA unwinding during replication ? DNA replication initiation ? MCM complex ?  0  BLAST | | | | | | | | | | | | | | | | | | | | |

## Cluster Pair #11: 11 gene pairs.

|  |  |  |  |  |  |  |  |  |  |  |  |  |  |  |  |  |  |  |  |  |  |  |  |  |  |  |  |  |  |  |  |  |  |  |  |
| --- | --- | --- | --- | --- | --- | --- | --- | --- | --- | --- | --- | --- | --- | --- | --- | --- | --- | --- | --- | --- | --- | --- | --- | --- | --- | --- | --- | --- | --- | --- | --- | --- | --- | --- | --- |
| P. falciparum S. cerevisiae Blast evalue|  |  |  |  |  |  |  |  |  |  |  |  |  |  |  |  |  |  |  |  |  |  |  |  |  |  |  |  |  |  |  |  |  | | --- | --- | --- | --- | --- | --- | --- | --- | --- | --- | --- | --- | --- | --- | --- | --- | --- | --- | --- | --- | --- | --- | --- | --- | --- | --- | --- | --- | --- | --- | --- | --- | --- | | PF10\_0348 ? hypothetical protein  receptor activity (IEA) ? pathogenesis (IEA) ? integral to membrane (IEA) ?  YDL058W ? soluble fraction ? protein complex assembly ? ER to Golgi vesicle-mediated transport ?  0.052  BLAST| PFD1130w ? hypothetical protein   YBR163W ? mitochondrion ?  0.014  BLAST| PFI1475w ? merozoite surface protein 1, precursor  pathogenesis (IEA) ? membrane (IEA) ?  YDL058W ? soluble fraction ? protein complex assembly ? ER to Golgi vesicle-mediated transport ?  4e-05  BLAST| PF14\_0172 ? hypothetical protein  metalloendopeptidase activity (IEA) ? proteolysis (IEA) ? pathogenesis (IEA) ? metal ion binding (IEA) ?  YDL058W ? soluble fraction ? protein complex assembly ? ER to Golgi vesicle-mediated transport ?  4e-05  BLAST| PF13\_0233 ? myosin a  motor activity ? actin binding ? ATP binding ? plasma membrane ? myosin complex ? pellicle ?  YAL029C ? microfilament motor activity ? mitochondrion ? cellular bud ? gene conversion at mating-type locus ? intracellular mRNA localization ? myosin V complex ? filamentous actin ? endoplasmic reticulum inheritance ? actin filament binding ?  0  BLAST| PFL1090w ? hypothetical protein   YDL058W ? soluble fraction ? protein complex assembly ? ER to Golgi vesicle-mediated transport ?  0.45  BLAST| PF13\_0173 ? hypothetical protein   YBR215W ? G1/S-specific transcription in mitotic cell cycle ? HIR complex ? nucleus ? DNA replication-independent nucleosome assembly ? RNA elongation from RNA polymerase II promoter ? transcription regulator activity ?  0.13  BLAST| PF11\_0477 ? CCAAT-box DNA binding protein subunit B  DNA binding ? nucleus (IEA) ? regulation of transcription, DNA-dependent ?  YBL021C ? nucleus ? regulation of carbohydrate metabolic process ? transcription ? transcription activator activity ? CCAAT-binding factor complex ?  3e-25  BLAST| PF11\_0278 ? hypothetical protein  membrane ? apicoplast ?  YDL058W ? soluble fraction ? protein complex assembly ? ER to Golgi vesicle-mediated transport ?  0.001  BLAST| PFL1340c ? hypothetical protein   YDL058W ? soluble fraction ? protein complex assembly ? ER to Golgi vesicle-mediated transport ?  0.0008  BLAST| PFB0670c ? hypothetical protein  membrane ?  YDL058W ? soluble fraction ? protein complex assembly ? ER to Golgi vesicle-mediated transport ?  2e-10  BLAST | | | | | | | | | | | | | | | | | | | | | | | | | | | | | | | | | | | |

## Cluster Pair #12: 4 gene pairs.

|  |  |  |  |  |  |  |  |  |  |  |  |  |  |  |
| --- | --- | --- | --- | --- | --- | --- | --- | --- | --- | --- | --- | --- | --- | --- |
| P. falciparum S. cerevisiae Blast evalue|  |  |  |  |  |  |  |  |  |  |  |  | | --- | --- | --- | --- | --- | --- | --- | --- | --- | --- | --- | --- | | PF11\_0331 ? t-complex protein 1, alpha subunit, putative  protein binding (IEA) ? ATP binding ? chaperonin-containing T-complex ? protein folding ? cytoskeleton organization and biogenesis ? cellular protein metabolic process (IEA) ? unfolded protein binding ?  YDR212W ? cytoplasm ? chaperonin-containing T-complex ? cytoskeleton ? protein folding ? cytoskeleton organization and biogenesis ? unfolded protein binding ?  0  BLAST| PFB0245c ? DNA-directed RNA polymerase II 16 kDa subunit, putative  DNA-directed RNA polymerase activity ? transcription (IEA) ? transcription from RNA polymerase II promoter ?  YJL140W ? mRNA catabolic process, deadenylation-dependent decay ? telomere maintenance ? cytoplasmic mRNA processing body ? DNA-directed RNA polymerase activity ? nucleus ? DNA-directed RNA polymerase II, core complex ? cytoplasm ? transcription from RNA polymerase II promoter ? mRNA export from nucleus ?  0.0003  BLAST| PFI1130c ? DNA-directed RNA polymerase II, putative  DNA binding (IEA) ? DNA-directed RNA polymerase activity (IEA) ? transcription (IEA) ? protein dimerization activity (IEA) ?  YIL021W ? DNA-directed RNA polymerase activity ? DNA-directed RNA polymerase II, core complex ? transcription from RNA polymerase II promoter ?  2e-22  BLAST| PF08\_0028 ? hypothetical protein   YGL112C ? G1-specific transcription in mitotic cell cycle ? SAGA complex ? transcription factor TFIID complex ? establishment and/or maintenance of chromatin architecture ? transcription initiation from RNA polymerase II promoter ? protein amino acid acetylation ? general RNA polymerase II transcription factor activity ? chromatin modification ? histone acetylation ? SLIK (SAGA-like) complex ?  0.1  BLAST | | | | | | | | | | | | | | |

## Cluster Pair #13: 5 gene pairs.

|  |  |  |  |  |  |  |  |  |  |  |  |  |  |  |  |  |  |
| --- | --- | --- | --- | --- | --- | --- | --- | --- | --- | --- | --- | --- | --- | --- | --- | --- | --- |
| P. falciparum S. cerevisiae Blast evalue|  |  |  |  |  |  |  |  |  |  |  |  |  |  |  | | --- | --- | --- | --- | --- | --- | --- | --- | --- | --- | --- | --- | --- | --- | --- | | PF14\_0324 ? hypothetical protein, conserved   YOR027W ? cytoplasm ? protein folding ? chaperone activator activity ? chaperone inhibitor activity ? Hsp70 protein binding ? Hsp90 protein binding ?  0  BLAST| PF10\_0153 ? hsp60  protein binding (IEA) ? ATP binding (IEA) ? mitochondrion ? protein folding ? protein targeting to mitochondrion ? response to unfolded protein ? ATPase activity, coupled ? cellular protein metabolic process (IEA) ? unfolded protein binding (IEA) ?  YLR259C ? single-stranded DNA binding ? mitochondrion ? protein folding ? protein import into mitochondrial matrix ? mitochondrial nucleoid ?  0  BLAST| PF07\_0029 ? heat shock protein 86  ATP binding (IEA) ? protein folding (IEA) ? response to unfolded protein ? response to heat ? unfolded protein binding (IEA) ? also with YPL240C, clust.pair #13 YMR186W ? telomere maintenance ? cytoplasm ? mitochondrion ? protein folding ? 'de novo' protein folding ? response to stress ? protein refolding ? ATPase activity, coupled ? proteasome assembly ? unfolded protein binding ?  0  BLAST| PF07\_0029 ? heat shock protein 86  ATP binding (IEA) ? protein folding (IEA) ? response to unfolded protein ? response to heat ? unfolded protein binding (IEA) ? also with YMR186W, clust.pair #13 YPL240C ? cytoplasm ? 'de novo' protein folding ? response to stress ? response to osmotic stress ? protein refolding ? ATPase activity, coupled ? proteasome assembly ? unfolded protein binding ?  0  BLAST| PFB0953w ? hypothetical protein  membrane ?  YBR169C ? adenyl-nucleotide exchange factor activity ? cytoplasm ? protein folding ? protein refolding ?  0.059  BLAST | | | | | | | | | | | | | | | | | |

## Cluster Pair #14: 5 gene pairs.

|  |  |  |  |  |  |  |  |  |  |  |  |  |  |  |  |  |  |
| --- | --- | --- | --- | --- | --- | --- | --- | --- | --- | --- | --- | --- | --- | --- | --- | --- | --- |
| P. falciparum S. cerevisiae Blast evalue|  |  |  |  |  |  |  |  |  |  |  |  |  |  |  | | --- | --- | --- | --- | --- | --- | --- | --- | --- | --- | --- | --- | --- | --- | --- | | PFE0975c ? 40S ribosomal subunit protein S24, putative  structural constituent of ribosome (IEA) ? intracellular (IEA) ? ribosome (IEA) ? translation (IEA) ? also with YIL069C, clust.pair #14 YER074W ? structural constituent of ribosome ? mitochondrion ? translation ?  3e-33  BLAST| PFE0975c ? 40S ribosomal subunit protein S24, putative  structural constituent of ribosome (IEA) ? intracellular (IEA) ? ribosome (IEA) ? translation (IEA) ? also with YER074W, clust.pair #14 YIL069C ? structural constituent of ribosome ? translation ?  3e-33  BLAST| PF14\_0627 ? ribosomal protein S3, putative  nucleic acid binding (IEA) ? structural constituent of ribosome ? intracellular (IEA) ? ribosome (IEA) ? translation ? small ribosomal subunit (IEA) ?  YNL178W ? structural constituent of ribosome ? translation ? response to DNA damage stimulus ? nucleolar preribosome, small subunit precursor ?  0  BLAST| MAL7P1.122 ? conserved GTP-binding protein, putative  GTP binding ? signal transduction ?  YBR025C ? cytoplasm ?  0  BLAST| PFE0630c ? orotate phosphoribosyltransferase, putative  orotate phosphoribosyltransferase activity ? nucleoside metabolic process (IEA) ?  YML106W ? orotate phosphoribosyltransferase activity ? nucleus ? cytoplasm ? 'de novo' pyrimidine base biosynthetic process ?  3e-16  BLAST | | | | | | | | | | | | | | | | | |

## Cluster Pair #15: 4 gene pairs.

|  |  |  |  |  |  |  |  |  |  |  |  |  |  |  |
| --- | --- | --- | --- | --- | --- | --- | --- | --- | --- | --- | --- | --- | --- | --- |
| P. falciparum S. cerevisiae Blast evalue|  |  |  |  |  |  |  |  |  |  |  |  | | --- | --- | --- | --- | --- | --- | --- | --- | --- | --- | --- | --- | | PFC0300c ? 60S ribosomal protein L7, putative  structural constituent of ribosome ? intracellular (IEA) ? ribosome (IEA) ? translation ? large ribosomal subunit (IEA) ? transcription regulator activity (IEA) ?  YPL198W ? structural constituent of ribosome ? translation ?  1.00053e-42  BLAST| PFD1055w ? ribosomal protein S19s, putative  structural constituent of ribosome ? intracellular (IEA) ? ribosome ? translation ? also with YOL121C, clust.pair #15 YNL302C ? ribosomal small subunit export from nucleus ? structural constituent of ribosome ? rRNA processing ? translation ? ribosomal small subunit biogenesis and assembly ?  3e-20  BLAST| PFD1055w ? ribosomal protein S19s, putative  structural constituent of ribosome ? intracellular (IEA) ? ribosome ? translation ? also with YNL302C, clust.pair #15 YOL121C ? ribosomal small subunit export from nucleus ? telomere maintenance ? structural constituent of ribosome ? rRNA processing ? translation ? ribosomal small subunit biogenesis and assembly ?  3e-20  BLAST| PFC0200w ? 60S Ribosomal protein L44, putative  structural constituent of ribosome ? intracellular (IEA) ? ribosome (IEA) ? translation ?  YNL162W ? structural constituent of ribosome ? translation ?  6e-30  BLAST | | | | | | | | | | | | | | |

## Cluster Pair #16: 4 gene pairs.

|  |  |  |  |  |  |  |  |  |  |  |  |  |  |  |
| --- | --- | --- | --- | --- | --- | --- | --- | --- | --- | --- | --- | --- | --- | --- |
| P. falciparum S. cerevisiae Blast evalue|  |  |  |  |  |  |  |  |  |  |  |  | | --- | --- | --- | --- | --- | --- | --- | --- | --- | --- | --- | --- | | PF07\_0117 ? eukaryotic translation initiation factor 2 alpha subunit, putative  nucleic acid binding (IEA) ? RNA binding (IEA) ? translation initiation factor activity ? eukaryotic translation initiation factor 2 complex ? translation (IEA) ? translational initiation ?  YJR007W ? translation initiation factor activity ? cytoplasm ? ribosome ? eukaryotic translation initiation factor 2 complex ? translational initiation ? multi-eIF complex ?  0  BLAST| MAL13P1.344 ? RNAse L inhibitor protein, putative  nucleotide binding (IEA) ? iron ion binding (IEA) ? ATP binding (IEA) ? electron transport (IEA) ? electron carrier activity (IEA) ? ATPase activity (IEA) ? nucleoside-triphosphatase activity (IEA) ?  YDR091C ? ribosome export from nucleus ? iron ion binding ? nucleus ? cytoplasm ? translational initiation ? ATPase activity ? ribosomal large subunit biogenesis and assembly ?  0  BLAST| PF14\_0411 ? small nuclear ribonuclear protein, putative  nucleus (IEA) ? small nucleolar ribonucleoprotein complex ? mRNA processing (IEA) ? RNA splicing ? ribonucleoprotein complex (IEA) ?  YER146W ? nuclear mRNA splicing, via spliceosome ? RNA binding ? snRNP U6 ? nucleolus ? small nucleolar ribonucleoprotein complex ? mRNA catabolic process ? RNA splicing factor activity, transesterification mechanism ? U4/U6 x U5 tri-snRNP complex ?  2e-08  BLAST| PFL2105c ? hypothetical protein   YKL018W ? telomere maintenance ? mRNA cleavage and polyadenylation specificity factor complex ? histone methylation ? termination of RNA polymerase II transcription, poly(A)-coupled ? termination of RNA polymerase II transcription, poly(A)-independent ? mRNA 3'-end processing ? snoRNA 3'-end processing ? histone lysine N-methyltransferase activity (H3-K4 specific) ? COMPASS complex ?  3e-14  BLAST | | | | | | | | | | | | | | |

## Cluster Pair #17: 3 gene pairs.

|  |  |  |  |  |  |  |  |  |  |  |  |
| --- | --- | --- | --- | --- | --- | --- | --- | --- | --- | --- | --- |
| P. falciparum S. cerevisiae Blast evalue|  |  |  |  |  |  |  |  |  | | --- | --- | --- | --- | --- | --- | --- | --- | --- | | PFL0960w ? D-ribulose-5-phosphate 3-epimerase, putative  ribulose-phosphate 3-epimerase activity ? carbohydrate metabolic process (IEA) ? pentose-phosphate shunt, non-oxidative branch ?  YJL121C ? ribulose-phosphate 3-epimerase activity ? cytosol ? pentose-phosphate shunt ?  0  BLAST| PF11\_0198 ? hypothetical protein, conserved  RNA binding (IEA) ? tRNA processing (IEA) ? tRNA methyltransferase activity (IEA) ?  YOL093W ? nucleus ? cytoplasm ? tRNA (guanine) methyltransferase activity ? tRNA methylation ?  5e-17  BLAST| PF07\_0011 ? hypothetical protein, conserved   YHR122W ? nucleus ? cytoplasm ? transcription ?  1e-23  BLAST | | | | | | | | | | | |

## Cluster Pair #18: 11 gene pairs.

|  |  |  |  |  |  |  |  |  |  |  |  |  |  |  |  |  |  |  |  |  |  |  |  |  |  |  |  |  |  |  |  |  |  |  |  |
| --- | --- | --- | --- | --- | --- | --- | --- | --- | --- | --- | --- | --- | --- | --- | --- | --- | --- | --- | --- | --- | --- | --- | --- | --- | --- | --- | --- | --- | --- | --- | --- | --- | --- | --- | --- |
| P. falciparum S. cerevisiae Blast evalue|  |  |  |  |  |  |  |  |  |  |  |  |  |  |  |  |  |  |  |  |  |  |  |  |  |  |  |  |  |  |  |  |  | | --- | --- | --- | --- | --- | --- | --- | --- | --- | --- | --- | --- | --- | --- | --- | --- | --- | --- | --- | --- | --- | --- | --- | --- | --- | --- | --- | --- | --- | --- | --- | --- | --- | | MAL13P1.209 ? 60S ribosomal subunit porotein L18, putative  structural constituent of ribosome ? intracellular (IEA) ? ribosome (IEA) ? translation ? also with YNL301C, clust.pair #23 YOL120C ? structural constituent of ribosome ? translation ?  8.00001e-42  BLAST| PF11\_0065 ? ribosomal protein S4, putative  RNA binding ? structural constituent of ribosome ? intracellular (IEA) ? mitochondrion ? ribosome (IEA) ? translation ? small ribosomal subunit ? also with YHR203C, clust.pair #22 YJR145C ? telomere maintenance ? structural constituent of ribosome ? cytoplasm ? translation ? maturation of SSU-rRNA ?  0  BLAST| PF13\_0268 ? ribosomal protein L17, putative  structural constituent of ribosome ? intracellular (IEA) ? ribosome (IEA) ? translation ? large ribosomal subunit (IEA) ? also with YJL177W, clust.pair #22 YKL180W ? structural constituent of ribosome ? cytoplasm ? translation ?  4.00001e-40  BLAST| PFC0290w ? 40S ribosomal protein S23, putative  nucleic acid binding (IEA) ? structural constituent of ribosome ? intracellular (IEA) ? ribosome (IEA) ? translation ? small ribosomal subunit (IEA) ? also with YGR118W, clust.pair #22 YPR132W ? telomere maintenance ? structural constituent of ribosome ? translation ? regulation of translational fidelity ?  0  BLAST| PFF0700c ? 60S ribosomal protein L19, putative  structural constituent of ribosome ? intracellular (IEA) ? ribosome (IEA) ? translation ? also with YBR084C-A, clust.pair #18 YBL027W ? structural constituent of ribosome ? translation ?  7e-39  BLAST| PFF0700c ? 60S ribosomal protein L19, putative  structural constituent of ribosome ? intracellular (IEA) ? ribosome (IEA) ? translation ? also with YBL027W, clust.pair #18 YBR084C-A ? structural constituent of ribosome ? translation ?  7e-39  BLAST| PFE0185c ? 60S ribosomal subunit protein L31, putative  structural constituent of ribosome ? intracellular (IEA) ? ribosome (IEA) ? translation ? large ribosomal subunit ? also with YLR406C, clust.pair #21 YDL075W ? structural constituent of ribosome ? translation ?  9e-21  BLAST| PFL2055w ? 40S ribosomal protein S17, putative  structural constituent of ribosome ? intracellular (IEA) ? mitochondrion ? ribosome (IEA) ? translation ?  YDR447C ? ribosomal small subunit assembly and maintenance ? telomere maintenance ? structural constituent of ribosome ? translation ?  4e-33  BLAST| PF13\_0228 ? 40S ribosomal subunit protein S6, putative  structural constituent of ribosome ? intracellular (IEA) ? ribosome (IEA) ? translation ? also with YPL090C, clust.pair #21 YBR181C ? structural constituent of ribosome ? small nucleolar ribonucleoprotein complex ? cytoplasm ? translation ?  0  BLAST| PFC0735w ? 40S ribosomal protein S15A, putative  structural constituent of ribosome ? intracellular (IEA) ? ribosome (IEA) ? translation ? also with YJL190C, clust.pair #21 YLR367W ? structural constituent of ribosome ? translation ?  0  BLAST| PF14\_0296 ? ribosomal protein L14, putative  structural constituent of ribosome ? intracellular (IEA) ? ribosome (IEA) ? translation ? apicoplast ? also with YKL006W, clust.pair #22 YHL001W ? RNA binding ? structural constituent of ribosome ? translation ?  0.006  BLAST | | | | | | | | | | | | | | | | | | | | | | | | | | | | | | | | | | | |

## Cluster Pair #19: 13 gene pairs.

|  |  |  |  |  |  |  |  |  |  |  |  |  |  |  |  |  |  |  |  |  |  |  |  |  |  |  |  |  |  |  |  |  |  |  |  |  |  |  |  |  |  |
| --- | --- | --- | --- | --- | --- | --- | --- | --- | --- | --- | --- | --- | --- | --- | --- | --- | --- | --- | --- | --- | --- | --- | --- | --- | --- | --- | --- | --- | --- | --- | --- | --- | --- | --- | --- | --- | --- | --- | --- | --- | --- |
| P. falciparum S. cerevisiae Blast evalue|  |  |  |  |  |  |  |  |  |  |  |  |  |  |  |  |  |  |  |  |  |  |  |  |  |  |  |  |  |  |  |  |  |  |  |  |  |  |  | | --- | --- | --- | --- | --- | --- | --- | --- | --- | --- | --- | --- | --- | --- | --- | --- | --- | --- | --- | --- | --- | --- | --- | --- | --- | --- | --- | --- | --- | --- | --- | --- | --- | --- | --- | --- | --- | --- | --- | | PF11\_0208 ? phosphoglycerate mutase, putative  phosphoglycerate mutase activity ? cytosol ? glycolysis ? intramolecular transferase activity, phosphotransferases (IEA) ?  YKL152C ? phosphoglycerate mutase activity ? mitochondrion ? cytosol ? gluconeogenesis ? glycolysis ?  0  BLAST| PF14\_0083 ? ribosomal protein S8e, putative  structural constituent of ribosome ? intracellular (IEA) ? mitochondrion ? translation ? also with YBL072C, clust.pair #21 YER102W ? structural constituent of ribosome ? translation ?  0  BLAST| PFB0885w ? 40S ribosomal protein S30, putative  structural constituent of ribosome ? intracellular (IEA) ? mitochondrion ? ribosome (IEA) ? translation ?  YOR182C ? telomere maintenance ? structural constituent of ribosome ? translation ?  4e-13  BLAST| PFC1020c ? 40S ribosomal protein S3A, putative  structural constituent of ribosome ? intracellular (IEA) ? ribosome (IEA) ? translation ? also with YML063W, clust.pair #22 YLR441C ? structural constituent of ribosome ? translation ?  0  BLAST| PF14\_0579 ? ribosomal protein L27, putative  structural constituent of ribosome ? intracellular (IEA) ? ribosome (IEA) ? translation ? also with YDR471W, clust.pair #23 YHR010W ? structural constituent of ribosome ? translation ?  1e-16  BLAST| PFD0770c ? ribosomal protein l15, putative  structural constituent of ribosome ? intracellular (IEA) ? ribosome ? translation ? also with YMR121C, clust.pair #23 YLR029C ? RNA binding ? structural constituent of ribosome ? translation ?  0  BLAST| PF08\_0076 ? 40S ribosomal protein S16, putative  structural constituent of ribosome ? intracellular (IEA) ? ribosome (IEA) ? translation ?  YDL083C ? telomere maintenance ? structural constituent of ribosome ? translation ?  9.80909e-45  BLAST| PF14\_0027 ? ribosomal S27a, putative  structural constituent of ribosome ? intracellular (IEA) ? ribosome (IEA) ? translation (IEA) ? protein modification process (IEA) ?  YLR167W ? ribosomal small subunit assembly and maintenance ? structural constituent of ribosome ? cytoplasm ? translation ? protein ubiquitination ? protein tag ? ribosome biogenesis and assembly ?  1e-12  BLAST| MAL13P1.92 ? 40S ribosomal protein S15, putative  structural constituent of ribosome ? intracellular (IEA) ? ribosome (IEA) ? translation ? small ribosomal subunit (IEA) ?  YOL040C ? ribosomal small subunit export from nucleus ? structural constituent of ribosome ? translation ?  5e-36  BLAST| PF08\_0039 ? ribosomal protein, putative  structural constituent of ribosome ? intracellular (IEA) ? ribosome (IEA) ? translation ?  YLR061W ? structural constituent of ribosome ? translation ?  1e-07  BLAST| PF07\_0043 ? 60S ribosomal protein L34-a, putative  structural constituent of ribosome (IEA) ? intracellular (IEA) ? ribosome (IEA) ? translation (IEA) ? also with YIL052C, clust.pair #22 YER056C-A ? structural constituent of ribosome ? translation ?  1e-30  BLAST| PF13\_0049 ? 60S ribosomal protein L24, putative  structural constituent of ribosome ? intracellular (IEA) ? ribosome (IEA) ? translation ? also with YGL031C, clust.pair #19 YGR148C ? RNA binding ? structural constituent of ribosome ? translation ?  3e-10  BLAST| PF13\_0049 ? 60S ribosomal protein L24, putative  structural constituent of ribosome ? intracellular (IEA) ? ribosome (IEA) ? translation ? also with YGR148C, clust.pair #19 YGL031C ? RNA binding ? structural constituent of ribosome ? translation ?  4e-10  BLAST | | | | | | | | | | | | | | | | | | | | | | | | | | | | | | | | | | | | | | | | | |

## Cluster Pair #20: 7 gene pairs.

|  |  |  |  |  |  |  |  |  |  |  |  |  |  |  |  |  |  |  |  |  |  |  |  |
| --- | --- | --- | --- | --- | --- | --- | --- | --- | --- | --- | --- | --- | --- | --- | --- | --- | --- | --- | --- | --- | --- | --- | --- |
| P. falciparum S. cerevisiae Blast evalue|  |  |  |  |  |  |  |  |  |  |  |  |  |  |  |  |  |  |  |  |  | | --- | --- | --- | --- | --- | --- | --- | --- | --- | --- | --- | --- | --- | --- | --- | --- | --- | --- | --- | --- | --- | | PF14\_0104 ? eukaryotic translation initiation factor 2 gamma subunit, putative  GTP binding ? eukaryotic translation initiation factor 2 complex ? translation (IEA) ? translational initiation ?  YER025W ? translation initiation factor activity ? ribosome ? eukaryotic translation initiation factor 2 complex ? translational initiation ? multi-eIF complex ?  0  BLAST| PF13\_0157 ? ribose-phosphate pyrophosphokinase, putative  histidine biosynthetic process ? tryptophan biosynthetic process ? ribose phosphate diphosphokinase activity ? nucleoside metabolic process ? nucleotide biosynthetic process (IEA) ? apicoplast ?  YKL181W ? histidine biosynthetic process ? tryptophan biosynthetic process ? ribose phosphate diphosphokinase activity ? cytoplasm ? purine ribonucleoside salvage ? 'de novo' IMP biosynthetic process ? 'de novo' pyrimidine base biosynthetic process ?  1e-35  BLAST| PFL0310c ? eukaryotic translation initiation factor 3 subunit 8, putative  translation initiation factor activity ? eukaryotic translation initiation factor 3 complex ? translational initiation (IEA) ? regulation of translational initiation ?  YMR309C ? translation initiation factor activity ? cytoplasm ? eukaryotic translation initiation factor 3 complex ? translational initiation ? ribosome biogenesis and assembly ? multi-eIF complex ?  9.99995e-41  BLAST| PF10\_0225 ? orotidine-monophosphate-decarboxylase, putative  orotidine-5'-phosphate decarboxylase activity ? 'de novo' pyrimidine base biosynthetic process ? pyrimidine nucleotide biosynthetic process (IEA) ?  YGR090W ? nucleus ? nucleolus ? small nucleolar ribonucleoprotein complex ? snoRNA binding ?  1.1  BLAST| PFF0345w ? translation initiation factor IF-2, putative  translation initiation factor activity ? GTP binding (IEA) ? translation (IEA) ? translational initiation ?  YAL035W ? translation initiation factor activity ? GTPase activity ? mitochondrion ? translational initiation ?  0  BLAST| PFE1335c ? hypothetical protein   YGR162W ? translation initiation factor activity ? mitochondrion ? ribosome ? translational initiation ? eukaryotic translation initiation factor 4F complex ? ribosome biogenesis and assembly ?  0.004  BLAST| PFE1140c ? G10 protein, putative  nucleus (IEA) ?  YCR063W ? cellular bud site selection ? nucleus ? RNA splicing ?  9.94922e-44  BLAST | | | | | | | | | | | | | | | | | | | | | | | |

## Cluster Pair #21: 18 gene pairs.

|  |  |  |  |  |  |  |  |  |  |  |  |  |  |  |  |  |  |  |  |  |  |  |  |  |  |  |  |  |  |  |  |  |  |  |  |  |  |  |  |  |  |  |  |  |  |  |  |  |  |  |  |  |  |  |  |  |
| --- | --- | --- | --- | --- | --- | --- | --- | --- | --- | --- | --- | --- | --- | --- | --- | --- | --- | --- | --- | --- | --- | --- | --- | --- | --- | --- | --- | --- | --- | --- | --- | --- | --- | --- | --- | --- | --- | --- | --- | --- | --- | --- | --- | --- | --- | --- | --- | --- | --- | --- | --- | --- | --- | --- | --- | --- |
| P. falciparum S. cerevisiae Blast evalue|  |  |  |  |  |  |  |  |  |  |  |  |  |  |  |  |  |  |  |  |  |  |  |  |  |  |  |  |  |  |  |  |  |  |  |  |  |  |  |  |  |  |  |  |  |  |  |  |  |  |  |  |  |  | | --- | --- | --- | --- | --- | --- | --- | --- | --- | --- | --- | --- | --- | --- | --- | --- | --- | --- | --- | --- | --- | --- | --- | --- | --- | --- | --- | --- | --- | --- | --- | --- | --- | --- | --- | --- | --- | --- | --- | --- | --- | --- | --- | --- | --- | --- | --- | --- | --- | --- | --- | --- | --- | --- | | PF08\_0019 ? guanine nucleotide-binding protein, putative  protein kinase C binding ? heterotrimeric G-protein complex ? G-protein coupled receptor protein signaling pathway ?  YMR116C ? telomere maintenance ? cytoplasm ? negative regulation of translation ?  0  BLAST| PF10\_0264 ? 40S ribosomal protein, putative  structural constituent of ribosome ? intracellular (IEA) ? ribosome (IEA) ? translation ? small ribosomal subunit (IEA) ? also with YGR214W, clust.pair #23 YLR048W ? ribosomal small subunit assembly and maintenance ? structural constituent of ribosome ? translation ?  0  BLAST| PF11\_0313 ? ribosomal phosphoprotein P0  structural constituent of ribosome ? intracellular (IEA) ? mitochondrion ? ribosome (IEA) ? translation ? translational elongation (IEA) ? ribosome biogenesis and assembly (IEA) ?  YLR340W ? ribosomal large subunit assembly and maintenance ? structural constituent of ribosome ? translation ? translational elongation ?  0  BLAST| PF14\_0240 ? ribosomal protein L21e, putative  structural constituent of ribosome ? mitochondrion ? translation ? also with YPL079W, clust.pair #22 YBR191W ? structural constituent of ribosome ? translation ?  4e-28  BLAST| PF14\_0083 ? ribosomal protein S8e, putative  structural constituent of ribosome ? intracellular (IEA) ? mitochondrion ? translation ? also with YER102W, clust.pair #19 YBL072C ? structural constituent of ribosome ? translation ?  0  BLAST| PF14\_0391 ? ribosomal protein L1, putative  structural constituent of ribosome ? intracellular (IEA) ? ribosome (IEA) ? translation ? also with YGL135W, clust.pair #23 YPL220W ? structural constituent of ribosome ? translation ?  0  BLAST| PFE0350c ? 60S ribosomal subunit protein L4%2FL1, putative  RNA binding ? structural constituent of ribosome ? intracellular (IEA) ? ribosome (IEA) ? translation ? large ribosomal subunit ? also with YDR012W, clust.pair #21 YBR031W ? structural constituent of ribosome ? translation ?  0  BLAST| PFE0350c ? 60S ribosomal subunit protein L4%2FL1, putative  RNA binding ? structural constituent of ribosome ? intracellular (IEA) ? ribosome (IEA) ? translation ? large ribosomal subunit ? also with YBR031W, clust.pair #21 YDR012W ? structural constituent of ribosome ? cytoplasm ? translation ?  0  BLAST| PF11\_0272 ? ribosomal protein S18, putative  RNA binding (IEA) ? structural constituent of ribosome ? intracellular (IEA) ? ribosome (IEA) ? translation ? small ribosomal subunit ?  YDR450W ? telomere maintenance ? structural constituent of ribosome ? mitochondrion ? translation ?  0  BLAST| PF11\_0043 ? 60S acidic ribosomal protein p1, putative  structural constituent of ribosome ? intracellular (IEA) ? ribosome (IEA) ? translation ? translational elongation (IEA) ? large ribosomal subunit ?  YDL081C ? telomere maintenance ? structural constituent of ribosome ? translation ? translational elongation ?  2e-06  BLAST| PFE0185c ? 60S ribosomal subunit protein L31, putative  structural constituent of ribosome ? intracellular (IEA) ? ribosome (IEA) ? translation ? large ribosomal subunit ? also with YDL075W, clust.pair #18 YLR406C ? structural constituent of ribosome ? translation ?  5e-21  BLAST| PFI0645w ? EF-1B  translation elongation factor activity (IEA) ? eukaryotic translation elongation factor 1 complex (IEA) ? translational elongation (IEA) ?  YAL003W ? translation elongation factor activity ? ribosome ? eukaryotic translation elongation factor 1 complex ? translational elongation ?  7e-06  BLAST| PF13\_0304 ? elongation factor 1 alpha  translation elongation factor activity ? GTP binding (IEA) ? cytoplasm (IEA) ? eukaryotic translation elongation factor 1 complex ? translation (IEA) ? translational elongation ? also with YPR080W, clust.pair #26 YBR118W ? translation elongation factor activity ? ribosome ? eukaryotic translation elongation factor 1 complex ? tRNA export from nucleus ? translational elongation ?  0  BLAST| PF13\_0228 ? 40S ribosomal subunit protein S6, putative  structural constituent of ribosome ? intracellular (IEA) ? ribosome (IEA) ? translation ? also with YBR181C, clust.pair #18 YPL090C ? structural constituent of ribosome ? small nucleolar ribonucleoprotein complex ? translation ?  0  BLAST| PFC0735w ? 40S ribosomal protein S15A, putative  structural constituent of ribosome ? intracellular (IEA) ? ribosome (IEA) ? translation ? also with YLR367W, clust.pair #18 YJL190C ? telomere maintenance ? structural constituent of ribosome ? translation ?  0  BLAST| PFE1005w ? 40S ribosomal subunit protein S9, putative  RNA binding (IEA) ? structural constituent of ribosome (IEA) ? intracellular (IEA) ? ribosome (IEA) ? translation (IEA) ? small ribosomal subunit (IEA) ? also with YPL081W, clust.pair #22 YBR189W ? structural constituent of ribosome ? small nucleolar ribonucleoprotein complex ? translation ? regulation of translational fidelity ?  0  BLAST| PF14\_0585 ? ribosomal protein S28e, putative  structural constituent of ribosome ? intracellular (IEA) ? ribosome (IEA) ? translation ? also with YLR264W, clust.pair #23 YOR167C ? structural constituent of ribosome ? translation ?  5e-12  BLAST| PFC0400w ? 60S Acidic ribosomal protein P2  structural constituent of ribosome (IEA) ? intracellular (IEA) ? ribosome (IEA) ? translational elongation ? large ribosomal subunit ?  YDR382W ? structural constituent of ribosome ? translation ? translational elongation ?  9e-11  BLAST | | | | | | | | | | | | | | | | | | | | | | | | | | | | | | | | | | | | | | | | | | | | | | | | | | | | | | | | |

## Cluster Pair #22: 23 gene pairs.

|  |  |  |  |  |  |  |  |  |  |  |  |  |  |  |  |  |  |  |  |  |  |  |  |  |  |  |  |  |  |  |  |  |  |  |  |  |  |  |  |  |  |  |  |  |  |  |  |  |  |  |  |  |  |  |  |  |  |  |  |  |  |  |  |  |  |  |  |  |  |  |  |
| --- | --- | --- | --- | --- | --- | --- | --- | --- | --- | --- | --- | --- | --- | --- | --- | --- | --- | --- | --- | --- | --- | --- | --- | --- | --- | --- | --- | --- | --- | --- | --- | --- | --- | --- | --- | --- | --- | --- | --- | --- | --- | --- | --- | --- | --- | --- | --- | --- | --- | --- | --- | --- | --- | --- | --- | --- | --- | --- | --- | --- | --- | --- | --- | --- | --- | --- | --- | --- | --- | --- | --- |
| P. falciparum S. cerevisiae Blast evalue|  |  |  |  |  |  |  |  |  |  |  |  |  |  |  |  |  |  |  |  |  |  |  |  |  |  |  |  |  |  |  |  |  |  |  |  |  |  |  |  |  |  |  |  |  |  |  |  |  |  |  |  |  |  |  |  |  |  |  |  |  |  |  |  |  |  |  |  |  | | --- | --- | --- | --- | --- | --- | --- | --- | --- | --- | --- | --- | --- | --- | --- | --- | --- | --- | --- | --- | --- | --- | --- | --- | --- | --- | --- | --- | --- | --- | --- | --- | --- | --- | --- | --- | --- | --- | --- | --- | --- | --- | --- | --- | --- | --- | --- | --- | --- | --- | --- | --- | --- | --- | --- | --- | --- | --- | --- | --- | --- | --- | --- | --- | --- | --- | --- | --- | --- | | PF13\_0214 ? elongation factor 1-gamma, putative  translation elongation factor activity ? glutathione transferase activity ? eukaryotic translation elongation factor 1 complex ? translational elongation ?  YKL081W ? translation elongation factor activity ? mitochondrion ? ribosome ? eukaryotic translation elongation factor 1 complex ? translational elongation ?  4e-24  BLAST| PF11\_0270 ? threonine -- tRNA ligase, putative  aminoacyl-tRNA ligase activity (IEA) ? threonine-tRNA ligase activity ? ATP binding ? mitochondrion ? translation (IEA) ? tRNA aminoacylation for protein translation (IEA) ? threonyl-tRNA aminoacylation ? apicoplast ?  YIL078W ? threonine-tRNA ligase activity ? cytoplasm ? mitochondrion ? translation ?  0  BLAST| PF14\_0240 ? ribosomal protein L21e, putative  structural constituent of ribosome ? mitochondrion ? translation ? also with YBR191W, clust.pair #21 YPL079W ? structural constituent of ribosome ? translation ?  5e-28  BLAST| PF14\_0141 ? ribosomal protein L10, putative  structural constituent of ribosome ? intracellular (IEA) ? ribosome (IEA) ? translation ?  YLR075W ? ribosomal large subunit assembly and maintenance ? structural constituent of ribosome ? translation ?  0  BLAST| PF11\_0065 ? ribosomal protein S4, putative  RNA binding ? structural constituent of ribosome ? intracellular (IEA) ? mitochondrion ? ribosome (IEA) ? translation ? small ribosomal subunit ? also with YJR145C, clust.pair #18 YHR203C ? telomere maintenance ? structural constituent of ribosome ? translation ?  0  BLAST| PF10\_0272 ? ribosomal protein L3, putative  structural constituent of ribosome ? intracellular (IEA) ? mitochondrion ? ribosome (IEA) ? translation ?  YOR063W ? ribosomal large subunit assembly and maintenance ? structural constituent of ribosome ? translation ?  0  BLAST| PF13\_0268 ? ribosomal protein L17, putative  structural constituent of ribosome ? intracellular (IEA) ? ribosome (IEA) ? translation ? large ribosomal subunit (IEA) ? also with YKL180W, clust.pair #18 YJL177W ? structural constituent of ribosome ? translation ?  3e-40  BLAST| PFC1020c ? 40S ribosomal protein S3A, putative  structural constituent of ribosome ? intracellular (IEA) ? ribosome (IEA) ? translation ? also with YLR441C, clust.pair #19 YML063W ? structural constituent of ribosome ? translation ?  0  BLAST| PF11\_0051 ? phenylalanine -- tRNA ligase, putative  phenylalanine-tRNA ligase activity ? ATP binding (IEA) ? cytoplasm (IEA) ? phenylalanyl-tRNA aminoacylation ? phenylalanine-tRNA ligase complex ?  YLR060W ? phenylalanine-tRNA ligase activity ? cytoplasm ? phenylalanyl-tRNA aminoacylation ? phenylalanine-tRNA ligase complex ?  0  BLAST| PFC0290w ? 40S ribosomal protein S23, putative  nucleic acid binding (IEA) ? structural constituent of ribosome ? intracellular (IEA) ? ribosome (IEA) ? translation ? small ribosomal subunit (IEA) ? also with YPR132W, clust.pair #18 YGR118W ? telomere maintenance ? structural constituent of ribosome ? translation ? regulation of translational fidelity ?  0  BLAST| PF13\_0213 ? 60S ribosomal subunit protein L6e, putative  structural constituent of ribosome ? intracellular (IEA) ? ribosome (IEA) ? translation ? also with YML073C, clust.pair #22 YLR448W ? ribosomal large subunit assembly and maintenance ? RNA binding ? structural constituent of ribosome ? translation ?  7e-16  BLAST| PF13\_0213 ? 60S ribosomal subunit protein L6e, putative  structural constituent of ribosome ? intracellular (IEA) ? ribosome (IEA) ? translation ? also with YLR448W, clust.pair #22 YML073C ? ribosomal large subunit assembly and maintenance ? RNA binding ? structural constituent of ribosome ? translation ?  1e-14  BLAST| PF10\_0043 ? ribosomal protein L13, putative  structural constituent of ribosome ? intracellular (IEA) ? ribosome (IEA) ? translation ? large ribosomal subunit (IEA) ? also with YNL069C, clust.pair #23 YIL133C ? RNA binding ? structural constituent of ribosome ? translation ?  0  BLAST| PF10\_0187 ? ribosomal protein L30e, putative  structural constituent of ribosome ? translation ?  YGL030W ? structural constituent of ribosome ? cytoplasm ? rRNA processing ? translation ? negative regulation of translation ? negative regulation of nuclear mRNA splicing, via spliceosome ?  2e-26  BLAST| PF13\_0129 ? ribosomal protein L6 homologue, putative  RNA binding ? structural constituent of ribosome ? intracellular (IEA) ? ribosome (IEA) ? translation ? large ribosomal subunit ? also with YNL067W, clust.pair #22 YGL147C ? structural constituent of ribosome ? translation ?  9.99995e-41  BLAST| PF13\_0129 ? ribosomal protein L6 homologue, putative  RNA binding ? structural constituent of ribosome ? intracellular (IEA) ? ribosome (IEA) ? translation ? large ribosomal subunit ? also with YGL147C, clust.pair #22 YNL067W ? structural constituent of ribosome ? translation ?  9.99995e-41  BLAST| PF10\_0103 ? eukaryotic translation initiation factor 2, beta, putative  RNA binding ? translation initiation factor activity ? eukaryotic translation initiation factor 2 complex ? translational initiation ?  YPL237W ? translation initiation factor activity ? ribosome ? eukaryotic translation initiation factor 2 complex ? translational initiation ? multi-eIF complex ?  6e-32  BLAST| PF14\_0448 ? ribosomal protein S2, putative  structural constituent of ribosome ? intracellular (IEA) ? ribosome (IEA) ? translation ? small ribosomal subunit (IEA) ?  YGL123W ? structural constituent of ribosome ? small nucleolar ribonucleoprotein complex ? translation ? regulation of translational fidelity ?  0  BLAST| PF13\_0014 ? 40S ribosomal protein S7 homologue, putative  structural constituent of ribosome ? intracellular (IEA) ? ribosome (IEA) ? translation ?  YNL096C ? structural constituent of ribosome ? small nucleolar ribonucleoprotein complex ? translation ?  5e-31  BLAST| PFE1005w ? 40S ribosomal subunit protein S9, putative  RNA binding (IEA) ? structural constituent of ribosome (IEA) ? intracellular (IEA) ? ribosome (IEA) ? translation (IEA) ? small ribosomal subunit (IEA) ? also with YBR189W, clust.pair #21 YPL081W ? structural constituent of ribosome ? small nucleolar ribonucleoprotein complex ? cytoplasm ? translation ? regulation of translational fidelity ?  0  BLAST| PF14\_0296 ? ribosomal protein L14, putative  structural constituent of ribosome ? intracellular (IEA) ? ribosome (IEA) ? translation ? apicoplast ? also with YHL001W, clust.pair #18 YKL006W ? RNA binding ? structural constituent of ribosome ? translation ?  0.004  BLAST| PF07\_0043 ? 60S ribosomal protein L34-a, putative  structural constituent of ribosome (IEA) ? intracellular (IEA) ? ribosome (IEA) ? translation (IEA) ? also with YER056C-A, clust.pair #19 YIL052C ? telomere maintenance ? structural constituent of ribosome ? translation ?  1e-30  BLAST| PF11\_0312 ? ribosomal protein L38e  structural constituent of ribosome ? intracellular (IEA) ? ribosome (IEA) ? translation ?  YLR325C ? structural constituent of ribosome ? translation ?  0.11  BLAST | | | | | | | | | | | | | | | | | | | | | | | | | | | | | | | | | | | | | | | | | | | | | | | | | | | | | | | | | | | | | | | | | | | | | | | |

## Cluster Pair #23: 30 gene pairs.

|  |  |  |  |  |  |  |  |  |  |  |  |  |  |  |  |  |  |  |  |  |  |  |  |  |  |  |  |  |  |  |  |  |  |  |  |  |  |  |  |  |  |  |  |  |  |  |  |  |  |  |  |  |  |  |  |  |  |  |  |  |  |  |  |  |  |  |  |  |  |  |  |  |  |  |  |  |  |  |  |  |  |  |  |  |  |  |  |  |  |  |  |  |
| --- | --- | --- | --- | --- | --- | --- | --- | --- | --- | --- | --- | --- | --- | --- | --- | --- | --- | --- | --- | --- | --- | --- | --- | --- | --- | --- | --- | --- | --- | --- | --- | --- | --- | --- | --- | --- | --- | --- | --- | --- | --- | --- | --- | --- | --- | --- | --- | --- | --- | --- | --- | --- | --- | --- | --- | --- | --- | --- | --- | --- | --- | --- | --- | --- | --- | --- | --- | --- | --- | --- | --- | --- | --- | --- | --- | --- | --- | --- | --- | --- | --- | --- | --- | --- | --- | --- | --- | --- | --- | --- | --- | --- |
| P. falciparum S. cerevisiae Blast evalue|  |  |  |  |  |  |  |  |  |  |  |  |  |  |  |  |  |  |  |  |  |  |  |  |  |  |  |  |  |  |  |  |  |  |  |  |  |  |  |  |  |  |  |  |  |  |  |  |  |  |  |  |  |  |  |  |  |  |  |  |  |  |  |  |  |  |  |  |  |  |  |  |  |  |  |  |  |  |  |  |  |  |  |  |  |  |  |  |  |  | | --- | --- | --- | --- | --- | --- | --- | --- | --- | --- | --- | --- | --- | --- | --- | --- | --- | --- | --- | --- | --- | --- | --- | --- | --- | --- | --- | --- | --- | --- | --- | --- | --- | --- | --- | --- | --- | --- | --- | --- | --- | --- | --- | --- | --- | --- | --- | --- | --- | --- | --- | --- | --- | --- | --- | --- | --- | --- | --- | --- | --- | --- | --- | --- | --- | --- | --- | --- | --- | --- | --- | --- | --- | --- | --- | --- | --- | --- | --- | --- | --- | --- | --- | --- | --- | --- | --- | --- | --- | --- | | PF14\_0486 ? elongation factor 2  translation elongation factor activity ? GTP binding ? translation (IEA) ? translational elongation ? also with YOR133W, clust.pair #23 YDR385W ? translation elongation factor activity ? ribosome ? translational elongation ?  0  BLAST| PF14\_0486 ? elongation factor 2  translation elongation factor activity ? GTP binding ? translation (IEA) ? translational elongation ? also with YDR385W, clust.pair #23 YOR133W ? translation elongation factor activity ? ribosome ? translational elongation ?  0  BLAST| PFE0660c ? uridine phosphorylase, putative  catalytic activity (IEA) ? uridine phosphorylase activity ? nucleoside metabolic process (IEA) ? also with YNL209W, clust.pair #23 YDL229W ? soluble fraction ? polysome ? translation ? regulation of translational fidelity ? ATPase activity ? unfolded protein binding ? 'de novo' cotranslational protein folding ?  1.3  BLAST| PFE0660c ? uridine phosphorylase, putative  catalytic activity (IEA) ? uridine phosphorylase activity ? nucleoside metabolic process (IEA) ? also with YDL229W, clust.pair #23 YNL209W ? cytoplasm ? polysome ? translation ? regulation of translational fidelity ? ATPase activity ? unfolded protein binding ? 'de novo' cotranslational protein folding ?  1.3  BLAST| PF10\_0264 ? 40S ribosomal protein, putative  structural constituent of ribosome ? intracellular (IEA) ? ribosome (IEA) ? translation ? small ribosomal subunit (IEA) ? also with YLR048W, clust.pair #21 YGR214W ? ribosomal small subunit assembly and maintenance ? structural constituent of ribosome ? translation ?  0  BLAST| MAL13P1.209 ? 60S ribosomal subunit porotein L18, putative  structural constituent of ribosome ? intracellular (IEA) ? ribosome (IEA) ? translation ? also with YOL120C, clust.pair #18 YNL301C ? structural constituent of ribosome ? translation ?  8.00001e-42  BLAST| PF13\_0143 ? phosphoribosylpyrophosphate synthetase  ribose phosphate diphosphokinase activity ? pentose-phosphate shunt ? purine ribonucleoside salvage ? nucleoside metabolic process (IEA) ? nucleotide biosynthetic process (IEA) ?  YBL068W ? histidine biosynthetic process ? tryptophan biosynthetic process ? ribose phosphate diphosphokinase activity ? cytoplasm ? purine ribonucleoside salvage ? 'de novo' IMP biosynthetic process ? 'de novo' pyrimidine base biosynthetic process ? ribosome biogenesis and assembly ?  0  BLAST| PFL0900c ? arginyl-tRNA synthetase, putative  arginine-tRNA ligase activity ? ATP binding (IEA) ? arginyl-tRNA aminoacylation ? apicoplast ?  YDR341C ? arginine-tRNA ligase activity ? cytoplasm ? mitochondrion ? translation ?  3.9937e-43  BLAST| PFL1745c ? clustered-asparagine-rich protein  nucleic acid binding (IEA) ? RNA binding ?  YER165W ? nucleus ? cytoplasm ? ribosome ? regulation of translational initiation ? poly(A) binding ?  4e-08  BLAST| PF14\_0391 ? ribosomal protein L1, putative  structural constituent of ribosome ? intracellular (IEA) ? ribosome (IEA) ? translation ? also with YPL220W, clust.pair #21 YGL135W ? telomere maintenance ? structural constituent of ribosome ? translation ?  0  BLAST| PF14\_0230 ? Ribosomal protein family L5, putative  structural constituent of ribosome ? intracellular (IEA) ? mitochondrion ? ribosome (IEA) ? translation ? 5S rRNA binding (IEA) ?  YPL131W ? ribosomal large subunit assembly and maintenance ? RNA binding ? structural constituent of ribosome ? translation ?  0  BLAST| PF07\_0088 ? 40S ribosomal protein S5, putative  structural constituent of ribosome ? intracellular (IEA) ? ribosome (IEA) ? translation ? small ribosomal subunit (IEA) ?  YJR123W ? structural constituent of ribosome ? translation ?  0  BLAST| PF14\_0579 ? ribosomal protein L27, putative  structural constituent of ribosome ? intracellular (IEA) ? ribosome (IEA) ? translation ? also with YHR010W, clust.pair #19 YDR471W ? structural constituent of ribosome ? translation ?  5e-16  BLAST| PFD0770c ? ribosomal protein l15, putative  structural constituent of ribosome ? intracellular (IEA) ? ribosome ? translation ? also with YLR029C, clust.pair #19 YMR121C ? RNA binding ? structural constituent of ribosome ? translation ?  0  BLAST| PF13\_0179 ? isoleucine--tRNA ligase, putative  aminoacyl-tRNA ligase activity (IEA) ? isoleucine-tRNA ligase activity (IEA) ? ATP binding (IEA) ? tRNA aminoacylation for protein translation (IEA) ? isoleucyl-tRNA aminoacylation (IEA) ?  YBL076C ? isoleucine-tRNA ligase activity ? cytosol ? translation ?  0  BLAST| PF14\_0028 ? hypothetical protein, conserved  nucleic acid binding (IEA) ? RNA binding (IEA) ? RNA processing (IEA) ? ATP biosynthetic process (IEA) ? ATP synthesis coupled proton transport (IEA) ? proton-transporting two-sector ATPase complex (IEA) ? hydrogen ion transporting ATP synthase activity, rotational mechanism (IEA) ? hydrogen ion transporting ATPase activity, rotational mechanism (IEA) ?  YER165W ? nucleus ? cytoplasm ? ribosome ? regulation of translational initiation ? poly(A) binding ?  6e-06  BLAST| PF13\_0354 ? alanine--tRNA ligase, putative  alanine-tRNA ligase activity ? ATP binding (IEA) ? alanyl-tRNA aminoacylation ? apicoplast ?  YOR335C ? alanine-tRNA ligase activity ? cytoplasm ? mitochondrion ? alanyl-tRNA aminoacylation ?  0  BLAST| PFB0445c ? helicase, putative  nucleic acid binding (IEA) ? ATP-dependent RNA helicase activity ? helicase activity (IEA) ? ATP binding (IEA) ? ATP-dependent helicase activity (IEA) ?  YDL084W ? transcription export complex ? U2-type nuclear mRNA branch site recognition ? nuclear mRNA splicing, via spliceosome ? chromosome, telomeric region ? RNA binding ? ATP-dependent RNA helicase activity ? protein binding ? nucleus ? spliceosome ? chromatin silencing at telomere ? mRNA export from nucleus ? RNA splicing factor activity, transesterification mechanism ?  0  BLAST| PF08\_0075 ? 60S ribosomal protein L13, putative  structural constituent of ribosome ? intracellular (IEA) ? ribosome (IEA) ? translation ?  YDL082W ? structural constituent of ribosome ? translation ?  3e-18  BLAST| PF11\_0200 ? U2 snRNP auxiliary factor, small subunit, putative  nuclear mRNA splicing, via spliceosome ? nucleic acid binding (IEA) ? RNA binding ? nucleus (IEA) ? snRNP U2 ?  YER165W ? nucleus ? cytoplasm ? ribosome ? regulation of translational initiation ? poly(A) binding ?  0.024  BLAST| PFL0670c ? Bi-functional aminoacyl-tRNA synthetase, putative  aminoacyl-tRNA ligase activity (IEA) ? glutamate-tRNA ligase activity ? proline-tRNA ligase activity ? ATP binding (IEA) ? cytoplasm (IEA) ? translation (IEA) ? tRNA aminoacylation for protein translation ? prolyl-tRNA aminoacylation (IEA) ?  YHR020W ? proline-tRNA ligase activity ? ribosome ? tRNA aminoacylation for protein translation ?  0  BLAST| PFD1070w ? eukaryotic initiation factor, putative  nucleic acid binding (IEA) ? translation initiation factor activity ? ATP-dependent RNA helicase activity ? helicase activity (IEA) ? ATP binding (IEA) ? regulation of translational initiation ? ATP-dependent helicase activity (IEA) ? eukaryotic translation initiation factor 4F complex ? also with YKR059W, clust.pair #23 YJL138C ? RNA helicase activity ? translation initiation factor activity ? cytoplasm ? ribosome ? translational initiation ? regulation of translational initiation ? eukaryotic translation initiation factor 4F complex ?  0  BLAST| PFD1070w ? eukaryotic initiation factor, putative  nucleic acid binding (IEA) ? translation initiation factor activity ? ATP-dependent RNA helicase activity ? helicase activity (IEA) ? ATP binding (IEA) ? regulation of translational initiation ? ATP-dependent helicase activity (IEA) ? eukaryotic translation initiation factor 4F complex ? also with YJL138C, clust.pair #23 YKR059W ? telomere maintenance ? translation initiation factor activity ? ATP-dependent RNA helicase activity ? ribosome ? translational initiation ? eukaryotic translation initiation factor 4F complex ?  0  BLAST| PF10\_0043 ? ribosomal protein L13, putative  structural constituent of ribosome ? intracellular (IEA) ? ribosome (IEA) ? translation ? large ribosomal subunit (IEA) ? also with YIL133C, clust.pair #22 YNL069C ? RNA binding ? structural constituent of ribosome ? translation ?  0  BLAST| PFI0190w ? ribosomal protein L32, putative  structural constituent of ribosome (IEA) ? intracellular (IEA) ? ribosome (IEA) ? translation (IEA) ?  YBL092W ? structural constituent of ribosome ? translation ?  6e-22  BLAST| PF14\_0125 ? deoxyhypusine synthase  translation ? peptidyl-lysine modification to hypusine ? membrane ? spermidine catabolic process to deoxyhypusine, using deoxyhypusine synthase ?  YHR068W ? cytoplasm ? peptidyl-lysine modification to hypusine ? transferase activity, transferring alkyl or aryl (other than methyl) groups ?  9.80909e-45  BLAST| PF14\_0231 ? ribosomal protein L7a, putative  structural constituent of ribosome ? intracellular (IEA) ? ribosome (IEA) ? translation ? ribonucleoprotein complex (IEA) ? ribosome biogenesis and assembly (IEA) ? also with YLL045C, clust.pair #23 YHL033C ? structural constituent of ribosome ? translation ?  2.00386e-43  BLAST| PF14\_0231 ? ribosomal protein L7a, putative  structural constituent of ribosome ? intracellular (IEA) ? ribosome (IEA) ? translation ? ribonucleoprotein complex (IEA) ? ribosome biogenesis and assembly (IEA) ? also with YHL033C, clust.pair #23 YLL045C ? structural constituent of ribosome ? translation ?  2.99878e-43  BLAST| PF11\_0250 ? high mobility group-like protein NHP2, putative  structural constituent of ribosome (IEA) ? intracellular (IEA) ? nucleus ? ribosome (IEA) ? translation (IEA) ? ribonucleoprotein complex (IEA) ? ribosome biogenesis and assembly (IEA) ?  YEL026W ? nuclear mRNA splicing, via spliceosome ? RNA binding ? nucleolus ? small nucleolar ribonucleoprotein complex ? maturation of SSU-rRNA ? RNA splicing factor activity, transesterification mechanism ? ribosome biogenesis and assembly ? U4/U6 x U5 tri-snRNP complex ?  3e-39  BLAST| PF14\_0585 ? ribosomal protein S28e, putative  structural constituent of ribosome ? intracellular (IEA) ? ribosome (IEA) ? translation ? also with YOR167C, clust.pair #21 YLR264W ? telomere maintenance ? structural constituent of ribosome ? translation ?  5e-12  BLAST | | | | | | | | | | | | | | | | | | | | | | | | | | | | | | | | | | | | | | | | | | | | | | | | | | | | | | | | | | | | | | | | | | | | | | | | | | | | | | | | | | | | | | | | | | | | |

## Cluster Pair #24: 7 gene pairs.

|  |  |  |  |  |  |  |  |  |  |  |  |  |  |  |  |  |  |  |  |  |  |  |  |
| --- | --- | --- | --- | --- | --- | --- | --- | --- | --- | --- | --- | --- | --- | --- | --- | --- | --- | --- | --- | --- | --- | --- | --- |
| P. falciparum S. cerevisiae Blast evalue|  |  |  |  |  |  |  |  |  |  |  |  |  |  |  |  |  |  |  |  |  | | --- | --- | --- | --- | --- | --- | --- | --- | --- | --- | --- | --- | --- | --- | --- | --- | --- | --- | --- | --- | --- | | PF13\_0224 ? 60S ribosomal subunit protein L18, putative  structural constituent of ribosome ? intracellular (IEA) ? ribosome (IEA) ? translation ? also with YMR242C, clust.pair #27 YOR312C ? structural constituent of ribosome ? translation ? ribosome biogenesis and assembly ?  4e-34  BLAST| PF11\_0454 ? Ribosomal protein, 40S subunit, putative  structural constituent of ribosome ? intracellular (IEA) ? ribosome (IEA) ? translation ? also with YJL136C, clust.pair #26 YKR057W ? telomere maintenance ? structural constituent of ribosome ? translation ?  4e-17  BLAST| PFC0775w ? 40S ribosomal protein S11, putative  nucleic acid binding (IEA) ? structural constituent of ribosome ? intracellular (IEA) ? ribosome (IEA) ? translation ? also with YDR025W, clust.pair #24 YBR048W ? ribosomal small subunit assembly and maintenance ? telomere maintenance ? structural constituent of ribosome ? translation ? regulation of translational fidelity ?  0  BLAST| PFC0775w ? 40S ribosomal protein S11, putative  nucleic acid binding (IEA) ? structural constituent of ribosome ? intracellular (IEA) ? ribosome (IEA) ? translation ? also with YBR048W, clust.pair #24 YDR025W ? ribosomal small subunit assembly and maintenance ? structural constituent of ribosome ? translation ? regulation of translational fidelity ?  0  BLAST| PF11\_0438 ? Ribosomal protein, putative  structural constituent of ribosome ? intracellular (IEA) ? ribosome (IEA) ? translation ? large ribosomal subunit ? also with YPL143W, clust.pair #24 YOR234C ? structural constituent of ribosome ? translation ?  1e-30  BLAST| PF11\_0438 ? Ribosomal protein, putative  structural constituent of ribosome ? intracellular (IEA) ? ribosome (IEA) ? translation ? large ribosomal subunit ? also with YOR234C, clust.pair #24 YPL143W ? structural constituent of ribosome ? translation ?  1e-30  BLAST| PF13\_0171 ? 60S ribosomal protein L23, putative  structural constituent of ribosome (IEA) ? intracellular (IEA) ? ribosome (IEA) ? translation (IEA) ? also with YER117W, clust.pair #27 YBL087C ? structural constituent of ribosome ? translation ?  0  BLAST | | | | | | | | | | | | | | | | | | | | | | | |

## Cluster Pair #25: 4 gene pairs.

|  |  |  |  |  |  |  |  |  |  |  |  |  |  |  |
| --- | --- | --- | --- | --- | --- | --- | --- | --- | --- | --- | --- | --- | --- | --- |
| P. falciparum S. cerevisiae Blast evalue|  |  |  |  |  |  |  |  |  |  |  |  | | --- | --- | --- | --- | --- | --- | --- | --- | --- | --- | --- | --- | | PFB0830w ? Ribosomal protein S26e, putative  structural constituent of ribosome ? intracellular (IEA) ? mitochondrion ? ribosome (IEA) ? translation ? also with YGL189C, clust.pair #25 YER131W ? structural constituent of ribosome ? translation ?  2e-26  BLAST| PFB0830w ? Ribosomal protein S26e, putative  structural constituent of ribosome ? intracellular (IEA) ? mitochondrion ? ribosome (IEA) ? translation ? also with YER131W, clust.pair #25 YGL189C ? structural constituent of ribosome ? translation ?  2e-26  BLAST| PF07\_0080 ? 40S ribosomal protein S10, putative  structural constituent of ribosome ? translation ?  YOR293W ? structural constituent of ribosome ? translation ?  2e-20  BLAST| PFC0535w ? 60S ribosomal protein L26, putative  structural constituent of ribosome ? intracellular (IEA) ? ribosome (IEA) ? translation ? large ribosomal subunit (IEA) ? also with YGR034W, clust.pair #26 YLR344W ? RNA binding ? structural constituent of ribosome ? translation ?  1e-28  BLAST | | | | | | | | | | | | | | |

## Cluster Pair #26: 8 gene pairs.

|  |  |  |  |  |  |  |  |  |  |  |  |  |  |  |  |  |  |  |  |  |  |  |  |  |  |  |
| --- | --- | --- | --- | --- | --- | --- | --- | --- | --- | --- | --- | --- | --- | --- | --- | --- | --- | --- | --- | --- | --- | --- | --- | --- | --- | --- |
| P. falciparum S. cerevisiae Blast evalue|  |  |  |  |  |  |  |  |  |  |  |  |  |  |  |  |  |  |  |  |  |  |  |  | | --- | --- | --- | --- | --- | --- | --- | --- | --- | --- | --- | --- | --- | --- | --- | --- | --- | --- | --- | --- | --- | --- | --- | --- | | PFE0845c ? 60S ribosomal subunit protein L8, putative  nucleic acid binding (IEA) ? structural constituent of ribosome ? intracellular (IEA) ? ribosome (IEA) ? translation ? large ribosomal subunit ? also with YIL018W, clust.pair #27 YFR031C-A ? structural constituent of ribosome ? translation ?  0  BLAST| PF13\_0305 ? elongation factor 1 alpha  translation elongation factor activity ? GTP binding (IEA) ? cytoplasm (IEA) ? eukaryotic translation elongation factor 1 complex ? translation (IEA) ? translational elongation ?  YPR080W ? translation elongation factor activity ? ribosome ? eukaryotic translation elongation factor 1 complex ? tRNA export from nucleus ? translational elongation ?  0  BLAST| PF13\_0304 ? elongation factor 1 alpha  translation elongation factor activity ? GTP binding (IEA) ? cytoplasm (IEA) ? eukaryotic translation elongation factor 1 complex ? translation (IEA) ? translational elongation ? also with YBR118W, clust.pair #21 YPR080W ? translation elongation factor activity ? ribosome ? eukaryotic translation elongation factor 1 complex ? tRNA export from nucleus ? translational elongation ?  0  BLAST| PF11\_0454 ? Ribosomal protein, 40S subunit, putative  structural constituent of ribosome ? intracellular (IEA) ? ribosome (IEA) ? translation ? also with YKR057W, clust.pair #24 YJL136C ? structural constituent of ribosome ? translation ?  6e-17  BLAST| PF13\_0045 ? 40S ribosomal protein S27, putative  structural constituent of ribosome ? intracellular (IEA) ? ribosome (IEA) ? translation ? also with YKL156W, clust.pair #26 YHR021C ? telomere maintenance ? structural constituent of ribosome ? translation ?  5e-27  BLAST| PF13\_0045 ? 40S ribosomal protein S27, putative  structural constituent of ribosome ? intracellular (IEA) ? ribosome (IEA) ? translation ? also with YHR021C, clust.pair #26 YKL156W ? structural constituent of ribosome ? cytoplasm ? translation ?  5e-27  BLAST| PF10\_0038 ? ribosomal protein S20e, putative  structural constituent of ribosome ? intracellular (IEA) ? ribosome (IEA) ? translation ? small ribosomal subunit (IEA) ?  YHL015W ? structural constituent of ribosome ? translation ?  3e-25  BLAST| PFC0535w ? 60S ribosomal protein L26, putative  structural constituent of ribosome ? intracellular (IEA) ? ribosome (IEA) ? translation ? large ribosomal subunit (IEA) ? also with YLR344W, clust.pair #25 YGR034W ? RNA binding ? structural constituent of ribosome ? translation ?  2e-28  BLAST | | | | | | | | | | | | | | | | | | | | | | | | | | |

## Cluster Pair #27: 7 gene pairs.

|  |  |  |  |  |  |  |  |  |  |  |  |  |  |  |  |  |  |  |  |  |  |  |  |
| --- | --- | --- | --- | --- | --- | --- | --- | --- | --- | --- | --- | --- | --- | --- | --- | --- | --- | --- | --- | --- | --- | --- | --- |
| P. falciparum S. cerevisiae Blast evalue|  |  |  |  |  |  |  |  |  |  |  |  |  |  |  |  |  |  |  |  |  | | --- | --- | --- | --- | --- | --- | --- | --- | --- | --- | --- | --- | --- | --- | --- | --- | --- | --- | --- | --- | --- | | PFE0845c ? 60S ribosomal subunit protein L8, putative  nucleic acid binding (IEA) ? structural constituent of ribosome ? intracellular (IEA) ? ribosome (IEA) ? translation ? large ribosomal subunit ? also with YFR031C-A, clust.pair #26 YIL018W ? structural constituent of ribosome ? translation ? response to drug ?  0  BLAST| PF14\_0655 ? RNA helicase-1, putative  RNA cap binding ? nucleic acid binding (IEA) ? mRNA binding ? translation initiation factor activity ? helicase activity (IEA) ? ATP binding (IEA) ? regulation of translational initiation ? ATP-dependent helicase activity ? eukaryotic translation initiation factor 4F complex ? also with YKR059W, clust.pair #27 YJL138C ? RNA helicase activity ? translation initiation factor activity ? cytoplasm ? ribosome ? translational initiation ? regulation of translational initiation ? eukaryotic translation initiation factor 4F complex ?  0  BLAST| PF14\_0655 ? RNA helicase-1, putative  RNA cap binding ? nucleic acid binding (IEA) ? mRNA binding ? translation initiation factor activity ? helicase activity (IEA) ? ATP binding (IEA) ? regulation of translational initiation ? ATP-dependent helicase activity ? eukaryotic translation initiation factor 4F complex ? also with YJL138C, clust.pair #27 YKR059W ? telomere maintenance ? translation initiation factor activity ? ATP-dependent RNA helicase activity ? ribosome ? translational initiation ? eukaryotic translation initiation factor 4F complex ?  0  BLAST| PF13\_0224 ? 60S ribosomal subunit protein L18, putative  structural constituent of ribosome ? intracellular (IEA) ? ribosome (IEA) ? translation ? also with YOR312C, clust.pair #24 YMR242C ? structural constituent of ribosome ? translation ?  4e-34  BLAST| PFE0810c ? 40S ribosomal subunit protein S14, putative  RNA binding ? structural constituent of ribosome (IEA) ? intracellular (IEA) ? ribosome (IEA) ? translation ?  YJL191W ? ribosomal small subunit assembly and maintenance ? RNA binding ? structural constituent of ribosome ? small nucleolar ribonucleoprotein complex ? translation ? maturation of SSU-rRNA ?  5.60519e-45  BLAST| PFB0455w ? ribosomal L37ae protein, putative  structural constituent of ribosome ? intracellular (IEA) ? mitochondrion ? ribosome (IEA) ? translation ?  YPR043W ? structural constituent of ribosome ? translation ?  9e-26  BLAST| PF13\_0171 ? 60S ribosomal protein L23, putative  structural constituent of ribosome (IEA) ? intracellular (IEA) ? ribosome (IEA) ? translation (IEA) ? also with YBL087C, clust.pair #24 YER117W ? structural constituent of ribosome ? translation ? response to drug ?  0  BLAST | | | | | | | | | | | | | | | | | | | | | | | |

## Cluster Pair #28: 6 gene pairs.

|  |  |  |  |  |  |  |  |  |  |  |  |  |  |  |  |  |  |  |  |  |
| --- | --- | --- | --- | --- | --- | --- | --- | --- | --- | --- | --- | --- | --- | --- | --- | --- | --- | --- | --- | --- |
| P. falciparum S. cerevisiae Blast evalue|  |  |  |  |  |  |  |  |  |  |  |  |  |  |  |  |  |  | | --- | --- | --- | --- | --- | --- | --- | --- | --- | --- | --- | --- | --- | --- | --- | --- | --- | --- | | PF10\_0155 ? enolase  phosphopyruvate hydratase complex ? phosphopyruvate hydratase activity ? gluconeogenesis ? glycolysis ?  YHR174W ? phosphopyruvate hydratase complex ? phosphopyruvate hydratase activity ? soluble fraction ? gluconeogenesis ? glycolysis ?  0  BLAST| PF14\_0378 ? triose-phosphate isomerase  triose-phosphate isomerase activity ? gluconeogenesis ? glycolysis ? pentose-phosphate shunt ? fatty acid biosynthetic process ? metabolic process (IEA) ?  YDR050C ? triose-phosphate isomerase activity ? cytoplasm ? glycolysis ?  0  BLAST| PFL0210c ? eukaryotic initiation factor 5a, putative  nucleic acid binding (IEA) ? translation initiation factor activity ? translational initiation ?  YEL034W ? translation initiation factor activity ? protein binding ? cytoplasm ? mitochondrion ? ribosome ? translational initiation ?  0  BLAST| PF14\_0023 ? hypothetical protein, conserved  membrane ?  YDR380W ? pyruvate decarboxylase activity ? cytoplasm ? leucine catabolic process ? L-phenylalanine catabolic process ? carboxy-lyase activity ? phenylpyruvate decarboxylase activity ?  1.7  BLAST| PF14\_0520 ? 6-phosphogluconate dehydrogenase, decarboxylating, putative  phosphogluconate dehydrogenase (decarboxylating) activity ? pentose-phosphate shunt ?  YHR183W ? phosphogluconate dehydrogenase (decarboxylating) activity ? cytoplasm ? mitochondrion ? response to oxidative stress ? pentose-phosphate shunt, oxidative branch ?  0  BLAST| PFL0705c ? adrenodoxin-type ferredoxin, putative  iron ion binding (IEA) ? mitochondrion ? electron transport ? electron carrier activity ? membrane ?  YPL252C ? mitochondrial matrix ? heme a biosynthetic process ? oxidoreductase activity, acting on NADH or NADPH, heme protein as acceptor ?  2e-33  BLAST | | | | | | | | | | | | | | | | | | | | |

## Cluster Pair #29: 6 gene pairs.

|  |  |  |  |  |  |  |  |  |  |  |  |  |  |  |  |  |  |  |  |  |
| --- | --- | --- | --- | --- | --- | --- | --- | --- | --- | --- | --- | --- | --- | --- | --- | --- | --- | --- | --- | --- |
| P. falciparum S. cerevisiae Blast evalue|  |  |  |  |  |  |  |  |  |  |  |  |  |  |  |  |  |  | | --- | --- | --- | --- | --- | --- | --- | --- | --- | --- | --- | --- | --- | --- | --- | --- | --- | --- | | PFD0305c ? vacuolar ATP synthase subunit b  ATP binding (IEA) ? cytoplasm (IEA) ? ATP biosynthetic process (IEA) ? vacuolar acidification ? hydrogen-exporting ATPase activity, phosphorylative mechanism (IEA) ? ATP synthesis coupled proton transport (IEA) ? energy coupled proton transport, against electrochemical gradient (IEA) ? proton-transporting two-sector ATPase complex (IEA) ? hydrogen ion transporting ATP synthase activity, rotational mechanism (IEA) ? hydrogen ion transporting ATPase activity, rotational mechanism ?  YBR127C ? vacuolar proton-transporting V-type ATPase, V1 domain ? cytoplasm ? cellular calcium ion homeostasis ? vacuolar acidification ? hydrogen ion transporting ATPase activity, rotational mechanism ?  0  BLAST| PF10\_0086 ? adenylate kinase, putative  adenylate kinase activity ? ATP binding (IEA) ? mitochondrion ? nucleobase, nucleoside, nucleotide and nucleic acid metabolic process (IEA) ? phosphotransferase activity, phosphate group as acceptor (IEA) ? nucleotide kinase activity (IEA) ?  YDR226W ? adenylate kinase activity ? cytoplasm ? mitochondrion ? mitochondrial intermembrane space ? ADP biosynthetic process ? nucleotide metabolic process ?  0  BLAST| PFE0285c ? ubiquitin-like protein, putative  protein modification process (IEA) ? ubiquitin cycle ? modification-dependent protein catabolic process ? protein ubiquitination during ubiquitin-dependent protein catabolic process ?  YDR510W ? nucleus ? septin ring ? protein sumoylation ? protein tag ?  1e-18  BLAST| MAL13P1.185 ? CDK-related protein kinase 6   YLR113W ? MAP kinase activity ? nucleus ? cytoplasm ? protein amino acid phosphorylation ? hyperosmotic response ? osmosensory signaling pathway ? positive regulation of transcription from RNA polymerase II promoter ? negative regulation of transcription from RNA polymerase II promoter by pheromones ? response to arsenic ?  6.02558e-44  BLAST| PFA0300c ? vacuolar ATP synthase, putative  ATP binding (IEA) ? mitochondrial proton-transporting ATP synthase complex ? ATP synthesis coupled proton transport ? proton-transporting two-sector ATPase complex (IEA) ? hydrolase activity, acting on acid anhydrides, catalyzing transmembrane movement of substances (IEA) ? hydrogen ion transporting ATPase activity, rotational mechanism ?  YKL080W ? vacuolar proton-transporting V-type ATPase, V1 domain ? membrane of vacuole with cell cycle-correlated morphology ? vacuolar acidification ? hydrogen ion transporting ATPase activity, rotational mechanism ?  9e-19  BLAST| PF11\_0203 ? hypothetical protein  nucleotide binding (IEA) ? metalloendopeptidase activity (IEA) ? ATP binding (IEA) ? proteolysis (IEA) ? zinc ion binding (IEA) ? membrane ? integral to membrane (IEA) ? nucleoside-triphosphatase activity (IEA) ? protein catabolic process (IEA) ?  YMR089C ? cytoplasm ? mitochondrion ? mitochondrial inner membrane ? m-AAA complex ? translation ? protein complex assembly ? signal peptide processing ? proteolysis ? metallopeptidase activity ? ATPase activity ? protein import into mitochondrial intermembrane space ?  0  BLAST | | | | | | | | | | | | | | | | | | | | |

## Cluster Pair #30: 7 gene pairs.

|  |  |  |  |  |  |  |  |  |  |  |  |  |  |  |  |  |  |  |  |  |  |  |  |
| --- | --- | --- | --- | --- | --- | --- | --- | --- | --- | --- | --- | --- | --- | --- | --- | --- | --- | --- | --- | --- | --- | --- | --- |
| P. falciparum S. cerevisiae Blast evalue|  |  |  |  |  |  |  |  |  |  |  |  |  |  |  |  |  |  |  |  |  | | --- | --- | --- | --- | --- | --- | --- | --- | --- | --- | --- | --- | --- | --- | --- | --- | --- | --- | --- | --- | --- | | PF13\_0164 ? hypothetical protein, conserved   YDR255C ? cytosol ? negative regulation of gluconeogenesis ?  0.004  BLAST| PF10\_0186 ? hypothetical protein  nucleic acid binding (IEA) ? metalloendopeptidase activity (IEA) ? proteolysis (IEA) ? pathogenesis (IEA) ? apicoplast ? metal ion binding (IEA) ?  YMR173W ? GTPase activity ? cytoplasm ? DNA repair ? ATPase activity ?  5e-05  BLAST| MAL7P1.155 ? hypothetical protein  ubiquitin ligase complex (IEA) ? ubiquitin-protein ligase activity (IEA) ? zinc ion binding (IEA) ? protein ubiquitination (IEA) ?  YDR255C ? cytosol ? negative regulation of gluconeogenesis ?  2e-08  BLAST| PF10\_0311 ? hypothetical protein, conserved   YFR003C ? protein phosphatase inhibitor activity ? nucleus ? glycogen metabolic process ? protein amino acid dephosphorylation ?  2e-07  BLAST| PFI1680w ? hypothetical protein   YDL091C ? cytoplasm ?  3e-06  BLAST| PFF1280w ? hypothetical protein   YMR173W ? GTPase activity ? cytoplasm ? DNA repair ? ATPase activity ?  4e-08  BLAST| PFF1275c ? 3-oxoacyl-%28acyl-carrier-protein%29 synthase i%2Fii  catalytic activity (IEA) ? fatty-acid synthase activity ? 3-oxoacyl-[acyl-carrier-protein] synthase activity ? fatty acid biosynthetic process (IEA) ? apicoplast ?  YER061C ? fatty-acid synthase activity ? mitochondrion ? fatty acid biosynthetic process ?  0  BLAST | | | | | | | | | | | | | | | | | | | | | | | |

## Cluster Pair #31: 5 gene pairs.

|  |  |  |  |  |  |  |  |  |  |  |  |  |  |  |  |  |  |
| --- | --- | --- | --- | --- | --- | --- | --- | --- | --- | --- | --- | --- | --- | --- | --- | --- | --- |
| P. falciparum S. cerevisiae Blast evalue|  |  |  |  |  |  |  |  |  |  |  |  |  |  |  | | --- | --- | --- | --- | --- | --- | --- | --- | --- | --- | --- | --- | --- | --- | --- | | PFL1655c ? hypothetical protein  DNA binding (IEA) ? DNA-directed DNA polymerase activity (IEA) ? nucleus (IEA) ? DNA replication (IEA) ?  YPR175W ? nucleus ? replication fork ? cytoplasm ? leading strand elongation ? lagging strand elongation ? nucleotide-excision repair ? mismatch repair ? epsilon DNA polymerase complex ?  1e-09  BLAST| PFF0520w ? calcium-dependent protein kinase  protein kinase activity (IEA) ? protein serine/threonine kinase activity (IEA) ? protein-tyrosine kinase activity (IEA) ? calcium ion binding (IEA) ? calmodulin binding (IEA) ? ATP binding (IEA) ? phosphorylase kinase complex (IEA) ? glycogen biosynthetic process (IEA) ? protein amino acid phosphorylation ? also with YDL101C, clust.pair #31 YPL153C ? DNA replication origin binding ? protein serine/threonine/tyrosine kinase activity ? nucleus ? nucleobase, nucleoside, nucleotide and nucleic acid metabolic process ? DNA replication initiation ? DNA repair ? deoxyribonucleoside triphosphate biosynthetic process ?  0  BLAST| PFF0520w ? calcium-dependent protein kinase  protein kinase activity (IEA) ? protein serine/threonine kinase activity (IEA) ? protein-tyrosine kinase activity (IEA) ? calcium ion binding (IEA) ? calmodulin binding (IEA) ? ATP binding (IEA) ? phosphorylase kinase complex (IEA) ? glycogen biosynthetic process (IEA) ? protein amino acid phosphorylation ? also with YPL153C, clust.pair #31 YDL101C ? cell cycle checkpoint ? DNA damage checkpoint ? telomere maintenance ? protein kinase activity ? nucleus ? protein amino acid phosphorylation ?  0  BLAST| PF14\_0602 ? DNA polymerase alpha subunit, putative  DNA binding (IEA) ? DNA-directed DNA polymerase activity (IEA) ? nucleus (IEA) ? alpha DNA polymerase:primase complex ? DNA replication (IEA) ? DNA replication, synthesis of RNA primer ?  YBL035C ? nucleus ? nuclear envelope ? alpha DNA polymerase:primase complex ? DNA replication initiation ? lagging strand elongation ? telomere capping ?  2e-16  BLAST| PFD0830w ? bifunctional dihydrofolate reductase-thymidylate synthase  dihydrofolate reductase activity ? thymidylate synthase activity ? dTMP biosynthetic process ? glycine biosynthetic process ? nucleotide biosynthetic process ?  YOR074C ? thymidylate synthase activity ? nucleus ? dTMP biosynthetic process ? DNA-dependent DNA replication ?  0  BLAST | | | | | | | | | | | | | | | | | |

## Cluster Pair #32: 13 gene pairs.

|  |  |  |  |  |  |  |  |  |  |  |  |  |  |  |  |  |  |  |  |  |  |  |  |  |  |  |  |  |  |  |  |  |  |  |  |  |  |  |  |  |  |
| --- | --- | --- | --- | --- | --- | --- | --- | --- | --- | --- | --- | --- | --- | --- | --- | --- | --- | --- | --- | --- | --- | --- | --- | --- | --- | --- | --- | --- | --- | --- | --- | --- | --- | --- | --- | --- | --- | --- | --- | --- | --- |
| P. falciparum S. cerevisiae Blast evalue|  |  |  |  |  |  |  |  |  |  |  |  |  |  |  |  |  |  |  |  |  |  |  |  |  |  |  |  |  |  |  |  |  |  |  |  |  |  |  | | --- | --- | --- | --- | --- | --- | --- | --- | --- | --- | --- | --- | --- | --- | --- | --- | --- | --- | --- | --- | --- | --- | --- | --- | --- | --- | --- | --- | --- | --- | --- | --- | --- | --- | --- | --- | --- | --- | --- | | PFL2115c ? glucose inhibited division protein A homologue, putative  membrane ? apicoplast ?  YGL236C ? mitochondrion ? tRNA modification ? translation ? response to drug ?  0  BLAST| PFF0115c ? elongation factor G, putative  translation elongation factor activity ? GTP binding (IEA) ? translation (IEA) ? translational elongation ? apicoplast ?  YLR069C ? translation elongation factor activity ? mitochondrion ? translational elongation ?  0  BLAST| PF07\_0062 ? GTP-binding translation elongation factor tu family protein, putative  translation elongation factor activity ? GTP binding ? translation (IEA) ? translational elongation ?  YLR069C ? translation elongation factor activity ? mitochondrion ? translational elongation ?  5e-21  BLAST| PFI1625c ? organelle processing peptidase, putative   YLR163C ? mitochondrial processing peptidase activity ? mitochondrion ? mitochondrial protein processing during import ? mitochondrial processing peptidase complex ?  0  BLAST| PFL1590c ? elongation factor g, putative  translation elongation factor activity ? GTP binding ? mitochondrion ? translation (IEA) ? translational elongation ?  YLR069C ? translation elongation factor activity ? mitochondrion ? translational elongation ?  0  BLAST| PF08\_0071 ? Fe-superoxide dismutase  superoxide dismutase activity (IEA) ? superoxide metabolic process ? response to oxidative stress ? metal ion binding (IEA) ?  YHR008C ? replicative cell aging ? age-dependent response to reactive oxygen species during chronological cell aging ? age-dependent response to oxidative stress during chronological cell aging ? mitochondrion ? mitochondrial matrix ? oxygen and reactive oxygen species metabolic process ?  6e-30  BLAST| PFF0155w ? bcs1-like protein, putative  nucleotide binding (IEA) ? ATP binding (IEA) ? mitochondrial inner membrane ? protein complex assembly ? ATPase activity ? nucleoside-triphosphatase activity (IEA) ?  YDR375C ? mitochondrion ? mitochondrial inner membrane ? aerobic respiration ? ATPase activity ? chaperone-mediated protein complex assembly ?  0  BLAST| PFI0570w ? GTP-binding protein, putative  GTP binding (IEA) ? translation (IEA) ? small GTPase mediated signal transduction (IEA) ? apicoplast ?  YLR289W ? GTPase activity ? mitochondrion ?  0  BLAST| MAL8P1.14 ? hypothetical protein  integral to membrane (IEA) ? protein insertion into membrane (IEA) ?  YER154W ? mitochondrion ? mitochondrial inner membrane ? protein transporter activity ? protein import into mitochondrial inner membrane ?  5e-05  BLAST| PF14\_0451 ? mitochondrial ribosomal protein S14 precursor, putative  structural constituent of ribosome ? mitochondrial small ribosomal subunit ? translation ?  YPR166C ? structural constituent of ribosome ? mitochondrion ? mitochondrial small ribosomal subunit ? translation ?  5e-06  BLAST| PF14\_0289 ? ribosomal protein L17, putative  organellar large ribosomal subunit ? structural constituent of ribosome ? intracellular (IEA) ? mitochondrion ? ribosome (IEA) ? translation ?  YJL063C ? mitochondrial genome maintenance ? structural constituent of ribosome ? mitochondrion ? mitochondrial large ribosomal subunit ? translation ?  4e-06  BLAST| PF10\_0252 ? hypothetical protein  copper ion binding (IEA) ? mitochondrial intermembrane space (IEA) ? copper ion transport (IEA) ? copper chaperone activity (IEA) ?  YLL009C ? mitochondrion ? mitochondrial intermembrane space ? cytosol ? thioredoxin peroxidase activity ? respiratory chain complex IV assembly ? intracellular copper ion transport ? copper chaperone activity ?  3e-07  BLAST| PFL0490c ? hypothetical protein, conserved   YNL315C ? mitochondrion ? mitochondrial matrix ? protein complex assembly ? unfolded protein binding ?  0.003  BLAST | | | | | | | | | | | | | | | | | | | | | | | | | | | | | | | | | | | | | | | | | |
