# Supplementary material for: Assessing functional annotation transfers with inter-species conserved coexpression: application to Plasmodium falciparum
Source: BMC Genomics. 2010 Jan 15;11:35. doi: 10.1186/1471-2164-11-35 (PMC2826313; doi:10.1186/1471-2164-11-35)
Supplement: Additional file 3 — Bozdech - Gasch analysis. This file presents the cluster pairs identified as revealing a conservation of coexpression when comparing the Bozdech and Gasch data. This file also provide additional information on the available functional annotations, as well as links to the BLAST alignments and the different databases (click on the '?'s to access PlasmoDB, SGD, and Amigo databases). Gene functional annotations are as follows. The short description immediately following each P. falciparum gene comes from PlasmoDB (red = functional gene, blue = putative gene, black = hypothetical gene). Other annotations are Gene Ontology annotations (red = Molecular Function, green = Biological Process, blue = Cellular Component). [file 1471-2164-11-35-S3.HTML]

# Bozdech - Gasch co-coexpression analysis

# 26 cluster pairs

## Cluster Pair #0: 4 gene pairs.

|  |  |  |  |  |  |  |  |  |  |  |  |  |  |  |
| --- | --- | --- | --- | --- | --- | --- | --- | --- | --- | --- | --- | --- | --- | --- |
| P.falciparum S.cerevisiae Blast evalue|  |  |  |  |  |  |  |  |  |  |  |  | | --- | --- | --- | --- | --- | --- | --- | --- | --- | --- | --- | --- | | PFI0630w ? 26S proteasome regulatory subunit, putative   YOR261C ? ubiquitin-dependent protein catabolism ? proteasome regulatory particle, lid subcomplex (sensu Eukaryota) ?  0  BLAST| PFE0380c ? hypothetical protein, conserved  protein modification (IEA) ?  YBR170C ? endoplasmic reticulum ? ER-associated protein catabolism ? nuclear envelope-endoplasmic reticulum network ?  7e-23  BLAST| PF14\_0716 ? Proteosome subunit alpha type 1, putative  endopeptidase activity ? threonine endopeptidase activity (IEA) ? proteasome core complex (sensu Eukaryota) ? ubiquitin-dependent protein catabolism ?  YMR314W ? endopeptidase activity ? ubiquitin-dependent protein catabolism ? proteasome core complex, alpha-subunit complex (sensu Eukaryota) ?  3.00018e-42  BLAST| MAL13P1.270 ? proteasome subunit, putative  endopeptidase activity ? threonine endopeptidase activity (IEA) ? proteasome core complex (sensu Eukaryota) ? ubiquitin-dependent protein catabolism ?  YOL038W ? endopeptidase activity ? mitochondrion ? cytosol ? ubiquitin-dependent protein catabolism ? proteasome core complex, alpha-subunit complex (sensu Eukaryota) ?  0  BLAST | | | | | | | | | | | | | | |

## Cluster Pair #1: 12 gene pairs.

|  |  |  |  |  |  |  |  |  |  |  |  |  |  |  |  |  |  |  |  |  |  |  |  |  |  |  |  |  |  |  |  |  |  |  |  |  |  |  |
| --- | --- | --- | --- | --- | --- | --- | --- | --- | --- | --- | --- | --- | --- | --- | --- | --- | --- | --- | --- | --- | --- | --- | --- | --- | --- | --- | --- | --- | --- | --- | --- | --- | --- | --- | --- | --- | --- | --- |
| P.falciparum S.cerevisiae Blast evalue|  |  |  |  |  |  |  |  |  |  |  |  |  |  |  |  |  |  |  |  |  |  |  |  |  |  |  |  |  |  |  |  |  |  |  |  | | --- | --- | --- | --- | --- | --- | --- | --- | --- | --- | --- | --- | --- | --- | --- | --- | --- | --- | --- | --- | --- | --- | --- | --- | --- | --- | --- | --- | --- | --- | --- | --- | --- | --- | --- | --- | | PF10\_0169 ? phosphomannomutase, putative  phosphomannomutase activity ? cytoplasm (IEA) ? GDP-mannose biosynthesis ? mannose biosynthesis (IEA) ?  YFL045C ? phosphomannomutase activity ? cytosol ? protein targeting to ER ?  0  BLAST| PF07\_0098 ? dynactin 4, putative  dynactin complex ? microtubule-based movement ? \*\* also with: YDR211W, clust.pair #2 YDL055C ? cell wall mannoprotein biosynthesis ? mannose-1-phosphate guanylyltransferase activity ? cytoplasm ? protein amino acid glycosylation ? GDP-mannose biosynthesis ?  0.081  BLAST| PFA0480w ? phenylalanyl-tRNA synthetase beta chain, putative  tRNA ligase activity (IEA) ? phenylalanine-tRNA ligase activity ? ATP binding (IEA) ? cytoplasm (IEA) ? tRNA aminoacylation for protein translation (IEA) ? phenylalanyl-tRNA aminoacylation ? phenylalanine-tRNA ligase complex ?  YFL022C ? phenylalanine-tRNA ligase activity ? cytoplasm ? phenylalanyl-tRNA aminoacylation ? phenylalanine-tRNA ligase complex ?  0  BLAST| MAL13P1.204 ? exoribonuclease PH, putative  3'-5'-exoribonuclease activity ? RNA binding (IEA) ? RNA processing (IEA) ? tRNA processing ?  YDL111C ? 3'-5'-exoribonuclease activity ? nuclear exosome (RNase complex) ? cytoplasmic exosome (RNase complex) ? 35S primary transcript processing ? mRNA catabolism ?  1e-07  BLAST| PFE1085w ? DEAD-box subfamily ATP-dependant helicase, putative  nucleic acid binding (IEA) ? helicase activity (IEA) ? ATP binding (IEA) ? ATP-dependent helicase activity (IEA) ? \*\* also with: YDL031W, clust.pair #2 \*\* also with: YGL078C, clust.pair #2 YDL084W ? transcription export complex ? U2-type nuclear mRNA branch site recognition ? nuclear mRNA splicing, via spliceosome ? chromosome, telomeric region ? RNA binding ? ATP-dependent RNA helicase activity ? protein binding ? nucleus ? spliceosome complex ? chromatin silencing at telomere ? mRNA-nucleus export ? RNA splicing factor activity, transesterification mechanism ?  9e-12  BLAST| PFI1140w ? NADPH-cytochrome p450 reductase  electron transport (IEA) ? electron carrier activity (IEA) ? FMN binding (IEA) ? oxidoreductase activity (IEA) ?  YHR042W ? mitochondrion ? mitochondrial outer membrane ? microsome ? electron transport ? ergosterol biosynthesis ? electron carrier activity ?  5e-22  BLAST| PFC0395w ? asparagine synthetase, putative  asparagine synthase (glutamine-hydrolyzing) activity ? asparagine biosynthesis ? metabolism (IEA) ?  YGR124W ? asparagine synthase (glutamine-hydrolyzing) activity ? cytoplasm ? asparagine biosynthesis ?  0  BLAST| PF13\_0354 ? alanine--tRNA ligase, putative  alanine-tRNA ligase activity ? ATP binding (IEA) ? alanyl-tRNA aminoacylation ? apicoplast ?  YOR335C ? alanine-tRNA ligase activity ? cytoplasm ? mitochondrion ? alanyl-tRNA aminoacylation ?  0  BLAST| PFE0885w ? eukaryotic translation initiation factor 3 subunit, putative  nucleic acid binding (IEA) ?  YOR361C ? translation initiation factor activity ? cytoplasm ? eukaryotic translation initiation factor 3 complex ? translational initiation ?  0  BLAST| PFL0665c ? RNA polymerase subunit 8c, putative  nucleic acid binding (IEA) ? DNA-directed RNA polymerase activity ? transcription (IEA) ? transcription initiation ?  YOR224C ? DNA-directed RNA polymerase activity ? DNA-directed RNA polymerase II, core complex ? DNA-directed RNA polymerase III complex ? DNA-directed RNA polymerase I complex ? transcription from RNA polymerase I promoter ? transcription from RNA polymerase II promoter ? transcription from RNA polymerase III promoter ?  1e-14  BLAST| PFL0335c ? eukaryotic translation initiation factor 5, putative  translation initiation factor activity ? translational initiation (IEA) ? regulation of translational initiation ?  YPR041W ? translation initiation factor activity ? GTPase activator activity ? cytosolic small ribosomal subunit (sensu Eukaryota) ? regulation of translational initiation ? mature ribosome assembly ?  6e-33  BLAST| PFL0900c ? arginyl-tRNA synthetase, putative  arginine-tRNA ligase activity ? ATP binding (IEA) ? arginyl-tRNA aminoacylation ? apicoplast ?  YDR341C ? arginine-tRNA ligase activity ? cytoplasm ? mitochondrion ? protein biosynthesis ?  3.9937e-43  BLAST | | | | | | | | | | | | | | | | | | | | | | | | | | | | | | | | | | | | | | |

## Cluster Pair #2: 30 gene pairs.

|  |  |  |  |  |  |  |  |  |  |  |  |  |  |  |  |  |  |  |  |  |  |  |  |  |  |  |  |  |  |  |  |  |  |  |  |  |  |  |  |  |  |  |  |  |  |  |  |  |  |  |  |  |  |  |  |  |  |  |  |  |  |  |  |  |  |  |  |  |  |  |  |  |  |  |  |  |  |  |  |  |  |  |  |  |  |  |  |  |  |  |  |  |
| --- | --- | --- | --- | --- | --- | --- | --- | --- | --- | --- | --- | --- | --- | --- | --- | --- | --- | --- | --- | --- | --- | --- | --- | --- | --- | --- | --- | --- | --- | --- | --- | --- | --- | --- | --- | --- | --- | --- | --- | --- | --- | --- | --- | --- | --- | --- | --- | --- | --- | --- | --- | --- | --- | --- | --- | --- | --- | --- | --- | --- | --- | --- | --- | --- | --- | --- | --- | --- | --- | --- | --- | --- | --- | --- | --- | --- | --- | --- | --- | --- | --- | --- | --- | --- | --- | --- | --- | --- | --- | --- | --- | --- |
| P.falciparum S.cerevisiae Blast evalue|  |  |  |  |  |  |  |  |  |  |  |  |  |  |  |  |  |  |  |  |  |  |  |  |  |  |  |  |  |  |  |  |  |  |  |  |  |  |  |  |  |  |  |  |  |  |  |  |  |  |  |  |  |  |  |  |  |  |  |  |  |  |  |  |  |  |  |  |  |  |  |  |  |  |  |  |  |  |  |  |  |  |  |  |  |  |  |  |  |  | | --- | --- | --- | --- | --- | --- | --- | --- | --- | --- | --- | --- | --- | --- | --- | --- | --- | --- | --- | --- | --- | --- | --- | --- | --- | --- | --- | --- | --- | --- | --- | --- | --- | --- | --- | --- | --- | --- | --- | --- | --- | --- | --- | --- | --- | --- | --- | --- | --- | --- | --- | --- | --- | --- | --- | --- | --- | --- | --- | --- | --- | --- | --- | --- | --- | --- | --- | --- | --- | --- | --- | --- | --- | --- | --- | --- | --- | --- | --- | --- | --- | --- | --- | --- | --- | --- | --- | --- | --- | --- | | PFL1820w ? hypothetical protein   YLR196W ? nucleus ? nucleolus ? cytoplasm ? rRNA processing ?  3e-15  BLAST| PF14\_0663 ? hypothetical protein  metalloendopeptidase activity (IEA) ? proteolysis and peptidolysis (IEA) ? pathogenesis (IEA) ? metal ion binding (IEA) ?  YDL167C ? cytoplasm ? ribosome biogenesis and assembly ?  3e-05  BLAST| PF13\_0315 ? RNA binding protein, putative  nucleic acid binding (IEA) ? RNA binding ? \*\* also with: YOL123W, clust.pair #2 YGR159C ? ribosomal small subunit assembly and maintenance ? telomere maintenance ? single-stranded DNA binding ? RNA binding ? nucleus ? nucleolus ? mitochondrion ? rRNA processing ?  7e-14  BLAST| PF13\_0315 ? RNA binding protein, putative  nucleic acid binding (IEA) ? RNA binding ? \*\* also with: YGR159C, clust.pair #2 YOL123W ? RNA binding ? nucleus ? cytoplasm ? mRNA cleavage factor complex ? mRNA polyadenylylation ? mRNA cleavage ?  1e-13  BLAST| PF11\_0445 ? DNA-directed RNA polymerase I, putative  DNA binding (IEA) ? DNA-directed RNA polymerase activity (IEA) ? transcription (IEA) ? protein dimerization activity (IEA) ?  YPR110C ? DNA-directed RNA polymerase activity ? DNA-directed RNA polymerase III complex ? DNA-directed RNA polymerase I complex ? transcription from RNA polymerase I promoter ? transcription from RNA polymerase III promoter ? ribosome biogenesis and assembly ?  9.94922e-44  BLAST| PF14\_0104 ? eukaryotic translation initiation factor 2 gamma subunit, putative  GTP binding ? eukaryotic translation initiation factor 2 complex ? protein biosynthesis (IEA) ? translational initiation ?  YER025W ? translation initiation factor activity ? ribosome ? eukaryotic translation initiation factor 2 complex ? translational initiation ?  0  BLAST| PF13\_0051 ? snornp protein gar1 homologue, putative  small nucleolar ribonucleoprotein complex (IEA) ? rRNA processing (IEA) ? RNA modification ? rRNA binding (IEA) ? ribosome biogenesis and assembly (IEA) ?  YHR089C ? RNA binding ? nucleolus ? small nucleolar ribonucleoprotein complex ? 35S primary transcript processing ? box H/ACA snoRNP complex ? ribosome biogenesis and assembly ?  8e-17  BLAST| MAL13P1.243 ? elongation factor Tu, putative  translation elongation factor activity ? GTP binding ? protein biosynthesis (IEA) ? translational elongation ?  YNL163C ? GTPase activity ? cytoplasm ? processing of 27S pre-rRNA ? ribosomal large subunit biogenesis ?  5e-39  BLAST| PFI0865w ? hypothetical protein, conserved  ATP binding (IEA) ?  YLR243W ? signal sequence binding ?  1e-34  BLAST| PF13\_0278 ? hypothetical protein  nucleic acid binding (IEA) ?  YDL167C ? cytoplasm ? ribosome biogenesis and assembly ?  5e-05  BLAST| PF11\_0316 ? hypothetical protein   YOL125W ? nucleus ? cytoplasm ? tRNA methyltransferase activity ? tRNA methylation ?  2e-06  BLAST| PF07\_0098 ? dynactin 4, putative  dynactin complex ? microtubule-based movement ? \*\* also with: YDL055C, clust.pair #1 YDR211W ? translation initiation factor activity ? guanyl-nucleotide exchange factor activity ? eukaryotic translation initiation factor 2B complex ? regulation of translational initiation ?  0.007  BLAST| PF10\_0123 ? GMP synthetase  catalytic activity (IEA) ? GMP synthase activity ? GMP synthase (glutamine-hydrolyzing) activity (IEA) ? ATP binding (IEA) ? purine nucleotide biosynthesis ? GMP biosynthesis (IEA) ? glutamine metabolism (IEA) ?  YMR217W ? GMP synthase (glutamine-hydrolyzing) activity ? GMP metabolism ?  0  BLAST| PF14\_0072 ? hypothetical protein, conserved   YNR046W ? tRNA (guanine-N2-)-methyltransferase activity ? nucleus ? nucleolus ? cytoplasm ? zinc ion binding ? tRNA methylation ?  2e-09  BLAST| PFE1085w ? DEAD-box subfamily ATP-dependant helicase, putative  nucleic acid binding (IEA) ? helicase activity (IEA) ? ATP binding (IEA) ? ATP-dependent helicase activity (IEA) ? \*\* also with: YDL084W, clust.pair #1 \*\* also with: YGL078C, clust.pair #2 YDL031W ? ribosomal large subunit assembly and maintenance ? ATP-dependent RNA helicase activity ? nucleolus ? 35S primary transcript processing ? ribosome biogenesis and assembly ?  2e-13  BLAST| PFE1085w ? DEAD-box subfamily ATP-dependant helicase, putative  nucleic acid binding (IEA) ? helicase activity (IEA) ? ATP binding (IEA) ? ATP-dependent helicase activity (IEA) ? \*\* also with: YDL084W, clust.pair #1 \*\* also with: YDL031W, clust.pair #2 YGL078C ? ribosomal large subunit assembly and maintenance ? ATP-dependent RNA helicase activity ? nucleolus ? 35S primary transcript processing ?  4e-12  BLAST| PFI1235w ? hypothetical protein   YDR083W ? telomere maintenance ? nucleolus ? rRNA processing ? methyltransferase activity ? ribosome biogenesis and assembly ?  6e-24  BLAST| PFC0155c ? DNA-directed RNA polymerase subunit I, putative  DNA binding (IEA) ? DNA-directed RNA polymerase activity ? DNA-directed RNA polymerase II, core complex ? transcription, DNA-dependent (IEA) ? transcription from RNA polymerase II promoter ?  YPR187W ? DNA-directed RNA polymerase activity ? DNA-directed RNA polymerase II, core complex ? DNA-directed RNA polymerase III complex ? DNA-directed RNA polymerase I complex ? transcription from RNA polymerase I promoter ? transcription from RNA polymerase II promoter ? transcription from RNA polymerase III promoter ?  4e-23  BLAST| PFI0625c ? hypothetical protein  translation initiation factor activity (IEA) ? regulation of translational initiation (IEA) ?  YNL062C ? tRNA binding ? nucleus ? translational initiation ? tRNA (adenine-N1-)-methyltransferase activity ? tRNA methylation ? ribosome biogenesis and assembly ?  5e-07  BLAST| PFI0415c ? ribosomal RNA methyltransferase, putative   YBR061C ? cytoplasm ? protein biosynthesis ? tRNA methyltransferase activity ? tRNA methylation ?  6.00036e-42  BLAST| PF14\_0620 ? hypothetical protein   YKR079C ? nucleus ? cytoplasm ? mitochondrion ? purine nucleotide binding ? removal of tRNA 3'-trailer sequence ? 3'-tRNA processing endoribonuclease activity ?  9e-23  BLAST| PF10\_0209 ? RNA helicase, putative  nucleic acid binding (IEA) ? RNA helicase activity ? helicase activity (IEA) ? ATP binding (IEA) ? ATP-dependent helicase activity (IEA) ?  YGL171W ? ATP-dependent RNA helicase activity ? nucleolus ? 35S primary transcript processing ? ATPase activity ?  2e-36  BLAST| PF13\_0157 ? ribose-phosphate pyrophosphokinase, putative  histidine biosynthesis ? tryptophan biosynthesis ? ribose phosphate diphosphokinase activity ? nucleoside metabolism ? nucleotide biosynthesis (IEA) ? apicoplast ?  YKL181W ? histidine biosynthesis ? tryptophan biosynthesis ? ribose phosphate diphosphokinase activity ? cytoplasm ? purine ribonucleoside salvage ? 'de novo' IMP biosynthesis ? 'de novo' pyrimidine base biosynthesis ?  1e-35  BLAST| PFB0375w ? hypothetical protein  metalloendopeptidase activity (IEA) ? proteolysis and peptidolysis (IEA) ? pathogenesis (IEA) ? membrane ? metal ion binding (IEA) ?  YDL167C ? cytoplasm ? ribosome biogenesis and assembly ?  0.0004  BLAST| PF10\_0197 ? hypothetical protein  apicoplast ?  YNL061W ? nucleolus ? rRNA processing ? RNA methyltransferase activity ? S-adenosylmethionine-dependent methyltransferase activity ? ribosome biogenesis and assembly ?  1e-06  BLAST| PF08\_0123 ? hypothetical protein   YPL212C ? nucleus ? tRNA modification ? tRNA-pseudouridine synthase activity ? ribosome biogenesis and assembly ?  2e-17  BLAST| PF14\_0636 ? hypothetical protein   YNR038W ? ribosomal large subunit assembly and maintenance ? ATP-dependent RNA helicase activity ? nucleolus ? 35S primary transcript processing ? nucleolar preribosome, large subunit precursor ? ribosome biogenesis and assembly ?  0.069  BLAST| PF14\_0494 ? hypothetical protein, conserved  mitochondrion ?  YDL060W ? nucleolus ? cytoplasm ? rRNA processing ? ribosome biogenesis and assembly ? ribonucleoprotein binding ?  2e-18  BLAST| PF14\_0156 ? dimethyladenosine transferase, putative  rRNA modification ? rRNA (adenine-N6,N6-)-dimethyltransferase activity (IEA) ? mitochondrion ? rRNA processing (IEA) ? rRNA methyltransferase activity ? S-adenosylmethionine-dependent methyltransferase activity (IEA) ? rRNA (adenine) methyltransferase activity (IEA) ?  YPL266W ? rRNA modification ? rRNA (adenine-N6,N6-)-dimethyltransferase activity ? nucleolus ? 35S primary transcript processing ? ribosome biogenesis and assembly ?  0  BLAST| PFL0330c ? DNA-directed RNA polymerase III subunit, putative  DNA binding (IEA) ? DNA-directed RNA polymerase activity ? DNA-directed RNA polymerase III complex ? transcription (IEA) ? transcription from RNA polymerase III promoter ?  YOR207C ? DNA-directed RNA polymerase activity ? DNA-directed RNA polymerase III complex ? transcription from RNA polymerase III promoter ?  0  BLAST | | | | | | | | | | | | | | | | | | | | | | | | | | | | | | | | | | | | | | | | | | | | | | | | | | | | | | | | | | | | | | | | | | | | | | | | | | | | | | | | | | | | | | | | | | | | |

## Cluster Pair #3: 2 gene pairs.

|  |  |  |  |  |  |  |  |  |
| --- | --- | --- | --- | --- | --- | --- | --- | --- |
| P.falciparum S.cerevisiae Blast evalue|  |  |  |  |  |  | | --- | --- | --- | --- | --- | --- | | PF10\_0111 ? 20S proteasome beta subunit, putative  endopeptidase activity ? threonine endopeptidase activity (IEA) ? proteasome core complex (sensu Eukaryota) ? ubiquitin-dependent protein catabolism ?  YPR103W ? endopeptidase activity ? ubiquitin-dependent protein catabolism ? proteasome core complex, beta-subunit complex (sensu Eukaryota) ?  0  BLAST| PF14\_0676 ? 20S proteasome beta 4 subunit, putative  endopeptidase activity ? threonine endopeptidase activity (IEA) ? proteasome core complex (sensu Eukaryota) ? ubiquitin-dependent protein catabolism ?  YER012W ? endopeptidase activity ? nucleus ? ubiquitin-dependent protein catabolism ? response to stress ? proteasome core complex, beta-subunit complex (sensu Eukaryota) ? sporulation (sensu Fungi) ?  9e-21  BLAST | | | | | | | | |

## Cluster Pair #4: 16 gene pairs.

|  |  |  |  |  |  |  |  |  |  |  |  |  |  |  |  |  |  |  |  |  |  |  |  |  |  |  |  |  |  |  |  |  |  |  |  |  |  |  |  |  |  |  |  |  |  |  |  |  |  |  |
| --- | --- | --- | --- | --- | --- | --- | --- | --- | --- | --- | --- | --- | --- | --- | --- | --- | --- | --- | --- | --- | --- | --- | --- | --- | --- | --- | --- | --- | --- | --- | --- | --- | --- | --- | --- | --- | --- | --- | --- | --- | --- | --- | --- | --- | --- | --- | --- | --- | --- | --- |
| P.falciparum S.cerevisiae Blast evalue|  |  |  |  |  |  |  |  |  |  |  |  |  |  |  |  |  |  |  |  |  |  |  |  |  |  |  |  |  |  |  |  |  |  |  |  |  |  |  |  |  |  |  |  |  |  |  |  | | --- | --- | --- | --- | --- | --- | --- | --- | --- | --- | --- | --- | --- | --- | --- | --- | --- | --- | --- | --- | --- | --- | --- | --- | --- | --- | --- | --- | --- | --- | --- | --- | --- | --- | --- | --- | --- | --- | --- | --- | --- | --- | --- | --- | --- | --- | --- | --- | | PF14\_0589 ? valine - tRNA ligase, putative  tRNA ligase activity (IEA) ? valine-tRNA ligase activity ? ATP binding (IEA) ? tRNA aminoacylation for protein translation (IEA) ? valyl-tRNA aminoacylation ?  YGR094W ? valine-tRNA ligase activity ? cytoplasm ? mitochondrion ? valyl-tRNA aminoacylation ?  0  BLAST| PF14\_0697 ? dihydroorotase, putative  dihydroorotase activity ? 'de novo' pyrimidine base biosynthesis ? hydrolase activity (IEA) ?  YLR420W ? dihydroorotase activity ? nucleus ? cytoplasm ? 'de novo' pyrimidine base biosynthesis ? pyrimidine nucleotide biosynthesis ?  9.94922e-44  BLAST| PF14\_0486 ? elongation factor 2  translation elongation factor activity ? GTP binding ? protein biosynthesis (IEA) ? translational elongation ? \*\* also with: YOR133W, clust.pair #4 YDR385W ? translation elongation factor activity ? ribosome ? translational elongation ?  0  BLAST| PF14\_0486 ? elongation factor 2  translation elongation factor activity ? GTP binding ? protein biosynthesis (IEA) ? translational elongation ? \*\* also with: YDR385W, clust.pair #4 YOR133W ? translation elongation factor activity ? ribosome ? translational elongation ?  0  BLAST| PF14\_0028 ? hypothetical protein, conserved  nucleic acid binding (IEA) ? RNA binding (IEA) ? RNA processing (IEA) ? ATP biosynthesis (IEA) ? ATP synthesis coupled proton transport (IEA) ? proton-transporting two-sector ATPase complex (IEA) ? hydrogen-transporting ATP synthase activity, rotational mechanism (IEA) ? hydrogen-transporting ATPase activity, rotational mechanism (IEA) ? \*\* also with: YER165W, clust.pair #5 \*\* also with: YGR159C, clust.pair #5 YNL016W ? mRNA catabolism, nonsense-mediated decay ? nucleic acid binding ? mRNA binding ? nucleus ? cytoplasm ? heterogeneous nuclear ribonucleoprotein complex ? regulation of mRNA stability ?  1e-06  BLAST| PFB0550w ? peptide chain release factor subunit 1, putative  translation release factor activity ? cytoplasm (IEA) ? translational termination (IEA) ? regulation of translational termination ? translation release factor activity, codon specific (IEA) ?  YBR143C ? cytokinesis ? cytosol ? translational termination ? translation release factor activity, codon specific ? translation release factor complex ?  0  BLAST| PF10\_0149 ? cysteine -- tRNA ligase, putative  cysteine-tRNA ligase activity ? ATP binding (IEA) ? translational elongation ? cysteinyl-tRNA aminoacylation ? apicoplast ?  YNL247W ? cysteine-tRNA ligase activity ? cytoplasm ? ribosome ? cysteinyl-tRNA aminoacylation ? cysteine metabolism ? ribosome biogenesis and assembly ?  0  BLAST| PF10\_0150 ? methionine aminopeptidase, putative  methionyl aminopeptidase activity ? regulation of protein biosynthesis ? regulation of translation ? protein modification ? proteolysis and peptidolysis ? metalloexopeptidase activity (IEA) ?  YLR244C ? methionyl aminopeptidase activity ? cytosolic ribosome (sensu Eukaryota) ? proteolysis and peptidolysis ?  0  BLAST| PFE0670w ? hypothetical protein  apicoplast ?  YLR008C ? presequence translocase-associated import motor ? ATPase stimulator activity ? mitochondrion ? mitochondrial inner membrane presequence translocase complex ? protein transporter activity ? mitochondrial matrix protein import ? unfolded protein binding ?  0.0006  BLAST| PFC0350c ? T-complex protein eta subunit, putative  protein binding (IEA) ? ATP binding (IEA) ? chaperonin-containing T-complex ? protein folding ? cellular protein metabolism (IEA) ? unfolded protein binding ?  YJL111W ? cytoplasm ? chaperonin-containing T-complex ? cytoskeleton ? protein folding ? cytoskeleton organization and biogenesis ? unfolded protein binding ?  0  BLAST| PFE0135w ? hypothetical protein  protein folding (IEA) ? heat shock protein binding (IEA) ? unfolded protein binding (IEA) ?  YOR254C ? mitochondrion ? endoplasmic reticulum membrane ? SRP-dependent cotranslational protein-membrane targeting ? posttranslational protein-membrane targeting ? protein transporter activity ? Sec62/Sec63 complex ? cytosol to ER transport ?  5e-06  BLAST| PF13\_0205 ? tryptophan--tRNA ligase, putative  tRNA ligase activity (IEA) ? tryptophan-tRNA ligase activity ? ATP binding (IEA) ? tRNA aminoacylation for protein translation (IEA) ? tryptophanyl-tRNA aminoacylation ? apicoplast ?  YOL097C ? tryptophan-tRNA ligase activity ? cytoplasm ? tryptophanyl-tRNA aminoacylation ?  0  BLAST| PF13\_0304 ? elongation factor 1 alpha  translation elongation factor activity ? GTP binding (IEA) ? cytoplasm (IEA) ? eukaryotic translation elongation factor 1 complex ? protein biosynthesis (IEA) ? translational elongation ? \*\* also with: YPR080W, clust.pair #4 YBR118W ? translation elongation factor activity ? ribosome ? eukaryotic translation elongation factor 1 complex ? tRNA-nucleus export ? translational elongation ?  0  BLAST| PF13\_0304 ? elongation factor 1 alpha  translation elongation factor activity ? GTP binding (IEA) ? cytoplasm (IEA) ? eukaryotic translation elongation factor 1 complex ? protein biosynthesis (IEA) ? translational elongation ? \*\* also with: YBR118W, clust.pair #4 YPR080W ? translation elongation factor activity ? ribosome ? eukaryotic translation elongation factor 1 complex ? tRNA-nucleus export ? translational elongation ?  0  BLAST| PF13\_0330 ? ATP-dependent DNA helicase, putative  nucleotide binding (IEA) ? ATP-dependent DNA helicase activity ? ATP binding (IEA) ? nucleus (IEA) ? nucleoside-triphosphatase activity (IEA) ? ATP-dependent 5' to 3' DNA helicase activity (IEA) ?  YPL235W ? SWR1 complex ? nucleus ? chromatin remodeling ? regulation of transcription from RNA polymerase II promoter ? 35S primary transcript processing ? snoRNA metabolism ? chromatin remodeling complex ? ATPase activity ? INO80 complex ? ATP-dependent 5' to 3' DNA helicase activity ?  0  BLAST| PF14\_0198 ? glycine -- tRNA ligase, putative  tRNA ligase activity (IEA) ? glycine-tRNA ligase activity ? ATP binding (IEA) ? protein biosynthesis (IEA) ? tRNA aminoacylation for protein translation (IEA) ? glycyl-tRNA aminoacylation ? apicoplast ?  YBR121C ? glycine-tRNA ligase activity ? cytoplasm ? mitochondrion ? transcription termination ? glycyl-tRNA aminoacylation ?  0  BLAST | | | | | | | | | | | | | | | | | | | | | | | | | | | | | | | | | | | | | | | | | | | | | | | | | | |

## Cluster Pair #5: 16 gene pairs.

|  |  |  |  |  |  |  |  |  |  |  |  |  |  |  |  |  |  |  |  |  |  |  |  |  |  |  |  |  |  |  |  |  |  |  |  |  |  |  |  |  |  |  |  |  |  |  |  |  |  |  |
| --- | --- | --- | --- | --- | --- | --- | --- | --- | --- | --- | --- | --- | --- | --- | --- | --- | --- | --- | --- | --- | --- | --- | --- | --- | --- | --- | --- | --- | --- | --- | --- | --- | --- | --- | --- | --- | --- | --- | --- | --- | --- | --- | --- | --- | --- | --- | --- | --- | --- | --- |
| P.falciparum S.cerevisiae Blast evalue|  |  |  |  |  |  |  |  |  |  |  |  |  |  |  |  |  |  |  |  |  |  |  |  |  |  |  |  |  |  |  |  |  |  |  |  |  |  |  |  |  |  |  |  |  |  |  |  | | --- | --- | --- | --- | --- | --- | --- | --- | --- | --- | --- | --- | --- | --- | --- | --- | --- | --- | --- | --- | --- | --- | --- | --- | --- | --- | --- | --- | --- | --- | --- | --- | --- | --- | --- | --- | --- | --- | --- | --- | --- | --- | --- | --- | --- | --- | --- | --- | | PFL0670c ? Bi-functional aminoacyl-tRNA synthetase, putative  tRNA ligase activity (IEA) ? glutamate-tRNA ligase activity ? proline-tRNA ligase activity ? ATP binding (IEA) ? cytoplasm (IEA) ? protein biosynthesis (IEA) ? tRNA aminoacylation for protein translation ? prolyl-tRNA aminoacylation (IEA) ?  YHR020W ? proline-tRNA ligase activity ? ribosome ? tRNA aminoacylation for protein translation ?  0  BLAST| PFD1235w ? erythrocyte membrane protein 1 %28PfEMP1%29  receptor activity (IEA) ? glycosaminoglycan binding (IEA) ? pathogenesis (IEA) ? integral to membrane (IEA) ? cell-cell adhesion ? host cell plasma membrane ? rosetting ? infected host cell surface knob ? antigenic variation ? cytoadherence to microvasculature ? cell adhesion molecule binding ?  YGR245C ? nucleus ? traversing start control point of mitotic cell cycle ? actin cytoskeleton organization and biogenesis ? ribosome biogenesis and assembly ? ribosome assembly ?  0.076  BLAST| PFD0460c ? hypothetical protein   YBR034C ? nucleus ? mRNA-nucleus export ? protein-arginine N-methyltransferase activity ? peptidyl-arginine modification ? ribosome biogenesis and assembly ?  0.1  BLAST| PF14\_0028 ? hypothetical protein, conserved  nucleic acid binding (IEA) ? RNA binding (IEA) ? RNA processing (IEA) ? ATP biosynthesis (IEA) ? ATP synthesis coupled proton transport (IEA) ? proton-transporting two-sector ATPase complex (IEA) ? hydrogen-transporting ATP synthase activity, rotational mechanism (IEA) ? hydrogen-transporting ATPase activity, rotational mechanism (IEA) ? \*\* also with: YNL016W, clust.pair #4 \*\* also with: YGR159C, clust.pair #5 YER165W ? nucleus ? cytoplasm ? ribosome ? regulation of translational initiation ? poly(A) binding ?  6e-06  BLAST| PF14\_0028 ? hypothetical protein, conserved  nucleic acid binding (IEA) ? RNA binding (IEA) ? RNA processing (IEA) ? ATP biosynthesis (IEA) ? ATP synthesis coupled proton transport (IEA) ? proton-transporting two-sector ATPase complex (IEA) ? hydrogen-transporting ATP synthase activity, rotational mechanism (IEA) ? hydrogen-transporting ATPase activity, rotational mechanism (IEA) ? \*\* also with: YNL016W, clust.pair #4 \*\* also with: YER165W, clust.pair #5 YGR159C ? ribosomal small subunit assembly and maintenance ? telomere maintenance ? single-stranded DNA binding ? RNA binding ? nucleus ? nucleolus ? mitochondrion ? rRNA processing ?  7e-06  BLAST| MAL13P1.289 ? mitotic control protein dis3 homologue, putative  exosome (RNase complex) ? RNA binding ? exonuclease activity ? ribonuclease activity (IEA) ?  YOL021C ? 3'-5'-exoribonuclease activity ? nuclear exosome (RNase complex) ? cytoplasmic exosome (RNase complex) ? mitochondrion ? 35S primary transcript processing ? mRNA catabolism ?  0  BLAST| PF11\_0116 ? hypothetical protein   YBL024W ? nucleus ? tRNA (cytosine-5-)-methyltransferase activity ? tRNA methylation ? ribosome biogenesis and assembly ?  2e-09  BLAST| PF11\_0071 ? RuvB DNA helicase, putative  nucleotide binding ? ATP binding (IEA) ? nucleus (IEA) ? DNA repair ? DNA recombination ? ATPase activity ? nucleoside-triphosphatase activity (IEA) ? ATP-dependent 5' to 3' DNA helicase activity (IEA) ?  YDR190C ? SWR1 complex ? nucleus ? chromatin remodeling ? regulation of transcription from RNA polymerase II promoter ? chromatin remodeling complex ? ATPase activity ? INO80 complex ? ATP-dependent 5' to 3' DNA helicase activity ?  0  BLAST| PFE0730c ? ribose 5-phosphate epimerase, putative  ribose-5-phosphate isomerase activity ? pentose-phosphate shunt, non-oxidative branch ?  YOR095C ? ribose-5-phosphate isomerase activity ? nucleus ? cytoplasm ? pentose-phosphate shunt ? pyridoxine biosynthesis ? ribosome biogenesis and assembly ?  2e-25  BLAST| PFE1240w ? hypothetical protein, conserved  catalytic activity (IEA) ? iron ion binding (IEA) ?  YPL207W ? endoplasmic reticulum ?  0  BLAST| PF14\_0087 ? hypothetical protein   YLL011W ? rRNA modification ? nucleolus ? small nucleolar ribonucleoprotein complex ? 35S primary transcript processing ? processing of 20S pre-rRNA ? snoRNA binding ? small nuclear ribonucleoprotein complex ?  0.054  BLAST| PFE1335c ? hypothetical protein   YGR162W ? translation initiation factor activity ? mitochondrion ? ribosome ? translational initiation ? eukaryotic translation initiation factor 4F complex ? ribosome biogenesis and assembly ?  0.004  BLAST| PF11\_0245 ? translation elongation factor EF-1, subunit alpha, putative  translation elongation factor activity ? translation release factor activity (IEA) ? GTP binding ? eukaryotic translation elongation factor 1 complex ? protein biosynthesis (IEA) ? translational elongation ? translational termination (IEA) ?  YDR172W ? mRNA catabolism, deadenylylation-dependent decay ? translation release factor activity ? cytosol ? translational termination ? translation release factor complex ?  0  BLAST| MAL13P1.344 ? RNAse L inhibitor protein, putative  nucleotide binding (IEA) ? iron ion binding (IEA) ? ATP binding (IEA) ? electron transport (IEA) ? electron carrier activity (IEA) ? ATPase activity (IEA) ? nucleoside-triphosphatase activity (IEA) ?  YDR091C ? ribosome-nucleus export ? iron ion binding ? nucleus ? cytoplasm ? cytosolic ribosome (sensu Eukaryota) ? translational initiation ? ATPase activity ? ribosomal large subunit biogenesis ?  0  BLAST| PF07\_0015 ? hypothetical protein  metalloendopeptidase activity (IEA) ? proteolysis and peptidolysis (IEA) ? pathogenesis (IEA) ? metal ion binding (IEA) ?  YBL024W ? nucleus ? tRNA (cytosine-5-)-methyltransferase activity ? tRNA methylation ? ribosome biogenesis and assembly ?  3e-39  BLAST| PFL2125c ? hypothetical protein   YPL122C ? nucleotide excision repair factor 3 complex ? nucleotide-excision repair, DNA duplex unwinding ? transcription factor TFIIH complex ? nucleotide-excision repair ? transcription initiation from RNA polymerase II promoter ? negative regulation of transcription from RNA polymerase II promoter, mitotic ? general RNA polymerase II transcription factor activity ?  3e-16  BLAST | | | | | | | | | | | | | | | | | | | | | | | | | | | | | | | | | | | | | | | | | | | | | | | | | | |

## Cluster Pair #6: 9 gene pairs.

|  |  |  |  |  |  |  |  |  |  |  |  |  |  |  |  |  |  |  |  |  |  |  |  |  |  |  |  |  |  |
| --- | --- | --- | --- | --- | --- | --- | --- | --- | --- | --- | --- | --- | --- | --- | --- | --- | --- | --- | --- | --- | --- | --- | --- | --- | --- | --- | --- | --- | --- |
| P.falciparum S.cerevisiae Blast evalue|  |  |  |  |  |  |  |  |  |  |  |  |  |  |  |  |  |  |  |  |  |  |  |  |  |  |  | | --- | --- | --- | --- | --- | --- | --- | --- | --- | --- | --- | --- | --- | --- | --- | --- | --- | --- | --- | --- | --- | --- | --- | --- | --- | --- | --- | | PF14\_0207 ? RNA polymerase subunit, putative  DNA binding (IEA) ? DNA-directed RNA polymerase activity ? nucleus (IEA) ? transcription (IEA) ? regulation of transcription, DNA-dependent ? membrane ?  YNR003C ? DNA-directed RNA polymerase activity ? nucleus ? DNA-directed RNA polymerase III complex ? cytoplasm ? transcription from RNA polymerase III promoter ? ribosome biogenesis and assembly ?  3e-11  BLAST| PFA0530c ? hypothetical protein, conserved   YDL166C ? ribosomal small subunit-nucleus export ? nucleus ? cytoplasm ? response to oxidative stress ? nucleoside-triphosphatase activity ? processing of 20S pre-rRNA ? preribosome ?  3e-13  BLAST| PF11\_0284 ? methyltransferase, putative  methyltransferase activity ? S-adenosylmethionine-dependent methyltransferase activity (IEA) ? membrane ?  YDL201W ? protein binding ? nucleus ? tRNA (guanine-N7-)-methyltransferase activity ? tRNA methylation ? ribosome biogenesis and assembly ?  5.60519e-45  BLAST| PF13\_0087 ? hypothetical protein, conserved  S-adenosylmethionine-dependent methyltransferase activity (IEA) ?  YJL125C ? nucleus ? tRNA (adenine-N1-)-methyltransferase activity ? tRNA methylation ? ribosome biogenesis and assembly ?  2e-35  BLAST| PF11\_0101 ? hypothetical protein   YBR155W ? cytoplasm ? protein folding ? Hsp70 protein binding ?  0.025  BLAST| PFE1370w ? hsp70 interacting protein, putative   YBR155W ? cytoplasm ? protein folding ? Hsp70 protein binding ?  1e-06  BLAST| PF14\_0086 ? hypothetical protein  tRNA processing (IEA) ? oxidoreductase activity (IEA) ? FAD binding (IEA) ?  YML080W ? nucleus ? tRNA modification ? tRNA dihydrouridine synthase activity ?  0  BLAST| PF14\_0677 ? RNA 3%27-Terminal Phosphate Cyclase-like protein, putative  RNA-3'-phosphate cyclase activity (IEA) ? ribosome biogenesis ? ribosome biogenesis and assembly ?  YOL010W ? RNA-3'-phosphate cyclase activity ? nucleolus ? 35S primary transcript processing ? ribosome biogenesis and assembly ?  1e-11  BLAST| PFL2150c ? hypothetical protein, conserved   YOR091W ? cytoplasm ? ribosome ? ribosome biogenesis and assembly ?  3e-36  BLAST | | | | | | | | | | | | | | | | | | | | | | | | | | | | | |

## Cluster Pair #7: 12 gene pairs.

|  |  |  |  |  |  |  |  |  |  |  |  |  |  |  |  |  |  |  |  |  |  |  |  |  |  |  |  |  |  |  |  |  |  |  |  |  |  |  |
| --- | --- | --- | --- | --- | --- | --- | --- | --- | --- | --- | --- | --- | --- | --- | --- | --- | --- | --- | --- | --- | --- | --- | --- | --- | --- | --- | --- | --- | --- | --- | --- | --- | --- | --- | --- | --- | --- | --- |
| P.falciparum S.cerevisiae Blast evalue|  |  |  |  |  |  |  |  |  |  |  |  |  |  |  |  |  |  |  |  |  |  |  |  |  |  |  |  |  |  |  |  |  |  |  |  | | --- | --- | --- | --- | --- | --- | --- | --- | --- | --- | --- | --- | --- | --- | --- | --- | --- | --- | --- | --- | --- | --- | --- | --- | --- | --- | --- | --- | --- | --- | --- | --- | --- | --- | --- | --- | | MAL8P1.142 ? proteasome beta-subunit  endopeptidase activity ? threonine endopeptidase activity (IEA) ? proteasome core complex (sensu Eukaryota) ? ubiquitin-dependent protein catabolism (IEA) ?  YFR050C ? endopeptidase activity ? ubiquitin-dependent protein catabolism ? proteasome core complex, beta-subunit complex (sensu Eukaryota) ?  5e-26  BLAST| PFC0520w ? 26S proteasome regulatory subunit S14, putative  proteasome regulatory particle (sensu Eukaryota) ? ubiquitin-dependent protein catabolism ?  YFR052W ? endopeptidase activity ? ubiquitin-dependent protein catabolism ? proteasome regulatory particle, lid subcomplex (sensu Eukaryota) ?  4e-14  BLAST| PF14\_0178 ? hypothetical protein  ubiquitin-dependent protein catabolism (IEA) ? membrane ?  YGR048W ? protein binding ? endoplasmic reticulum ? mRNA processing ? ubiquitin-dependent protein catabolism ? protein transport ?  7e-34  BLAST| PFA0400c ? beta3 proteasome subunit, putative  endopeptidase activity ? threonine endopeptidase activity (IEA) ? proteasome core complex (sensu Eukaryota) ? ubiquitin-dependent protein catabolism ?  YER094C ? endopeptidase activity ? ubiquitin-dependent protein catabolism ? proteasome core complex, beta-subunit complex (sensu Eukaryota) ?  0  BLAST| PF07\_0112 ? proteasome subunit alpha type 5, putative  endopeptidase activity ? threonine endopeptidase activity (IEA) ? proteasome core complex (sensu Eukaryota) (IEA) ? ubiquitin-dependent protein catabolism ? proteasome core complex, alpha-subunit complex (sensu Eukaryota) ?  YGR253C ? endopeptidase activity ? ubiquitin-dependent protein catabolism ? response to stress ? proteasome core complex, alpha-subunit complex (sensu Eukaryota) ? sporulation (sensu Fungi) ?  0  BLAST| PFE0915c ? proteasome subunit beta type 1  endopeptidase activity ? threonine endopeptidase activity (IEA) ? proteasome core complex (sensu Eukaryota) ? ubiquitin-dependent protein catabolism ?  YBL041W ? endopeptidase activity ? ubiquitin-dependent protein catabolism ? proteasome core complex, beta-subunit complex (sensu Eukaryota) ?  9e-35  BLAST| PF10\_0174 ? 26s proteasome subunit p55, putative  endopeptidase activity ? proteasome regulatory particle (sensu Eukaryota) ? ubiquitin-dependent protein catabolism ?  YDL147W ? ubiquitin-dependent protein catabolism ? proteasome regulatory particle, lid subcomplex (sensu Eukaryota) ?  0  BLAST| PF14\_0025 ? proteosome subunit, putative  endopeptidase activity ? proteasome regulatory particle (sensu Eukaryota) ? proteolysis and peptidolysis ?  YDL097C ? structural molecule activity ? ubiquitin-dependent protein catabolism ? proteasome regulatory particle, lid subcomplex (sensu Eukaryota) ?  8e-21  BLAST| PFI1545c ? proteasome precursor, putative  threonine endopeptidase activity (IEA) ? proteasome core complex (sensu Eukaryota) (IEA) ? ubiquitin-dependent protein catabolism (IEA) ?  YJL001W ? endopeptidase activity ? ubiquitin-dependent protein catabolism ? response to stress ? proteasome core complex, beta-subunit complex (sensu Eukaryota) ? sporulation (sensu Fungi) ?  2e-15  BLAST| PFC0745c ? proteasome component C8, putative  endopeptidase activity ? threonine endopeptidase activity (IEA) ? proteasome core complex (sensu Eukaryota) ? ubiquitin-dependent protein catabolism ?  YOR362C ? endopeptidase activity ? ubiquitin-dependent protein catabolism ? proteasome core complex, alpha-subunit complex (sensu Eukaryota) ?  1e-32  BLAST| MAL8P1.128 ? proteasome subunit alpha, putative  endopeptidase activity ? threonine endopeptidase activity (IEA) ? proteasome core complex (sensu Eukaryota) ? ubiquitin-dependent protein catabolism ?  YGL011C ? endopeptidase activity ? mitochondrion ? ubiquitin-dependent protein catabolism ? proteasome core complex, alpha-subunit complex (sensu Eukaryota) ?  1e-34  BLAST| PF13\_0282 ? proteasome subunit, putative  endopeptidase activity ? threonine endopeptidase activity (IEA) ? proteasome core complex (sensu Eukaryota) ? ubiquitin-dependent protein catabolism ?  YGR135W ? endopeptidase activity ? ubiquitin-dependent protein catabolism ? proteasome core complex, alpha-subunit complex (sensu Eukaryota) ? filamentous growth ?  0  BLAST | | | | | | | | | | | | | | | | | | | | | | | | | | | | | | | | | | | | | | |

## Cluster Pair #8: 6 gene pairs.

|  |  |  |  |  |  |  |  |  |  |  |  |  |  |  |  |  |  |  |  |  |
| --- | --- | --- | --- | --- | --- | --- | --- | --- | --- | --- | --- | --- | --- | --- | --- | --- | --- | --- | --- | --- |
| P.falciparum S.cerevisiae Blast evalue|  |  |  |  |  |  |  |  |  |  |  |  |  |  |  |  |  |  | | --- | --- | --- | --- | --- | --- | --- | --- | --- | --- | --- | --- | --- | --- | --- | --- | --- | --- | | PFD0665c ? 26s proteasome aaa-ATPase subunit Rpt3, putative  nucleotide binding (IEA) ? ATP binding (IEA) ? nucleus (IEA) ? cytoplasm (IEA) ? proteasome regulatory particle (sensu Eukaryota) ? ubiquitin-dependent protein catabolism ? hydrolase activity (IEA) ? ATPase activity ? nucleoside-triphosphatase activity (IEA) ? protein catabolism (IEA) ?  YDR394W ? endopeptidase activity ? ubiquitin-dependent protein catabolism ? proteasome regulatory particle, base subcomplex (sensu Eukaryota) ? ATPase activity ?  0  BLAST| PF10\_0081 ? 26S proteasome regulatory subunit 4, putative  nucleotide binding (IEA) ? endopeptidase activity ? ATP binding (IEA) ? nucleus (IEA) ? cytoplasm (IEA) ? proteasome regulatory particle (sensu Eukaryota) ? proteolysis and peptidolysis ? hydrolase activity (IEA) ? ATPase activity (IEA) ? nucleoside-triphosphatase activity (IEA) ? protein catabolism (IEA) ?  YDL007W ? endopeptidase activity ? nucleus ? ubiquitin-dependent protein catabolism ? proteasome regulatory particle, base subcomplex (sensu Eukaryota) ? ATPase activity ?  0  BLAST| PF11\_0314 ? 26S protease subunit regulatory subunit 6a, putative  nucleotide binding (IEA) ? proteasome complex (sensu Eukaryota) ? endopeptidase activity ? ATP binding (IEA) ? nucleus (IEA) ? cytoplasm (IEA) ? proteolysis and peptidolysis ? hydrolase activity (IEA) ? ATPase activity ? nucleoside-triphosphatase activity (IEA) ? protein catabolism (IEA) ?  YOR117W ? endopeptidase activity ? ubiquitin-dependent protein catabolism ? proteasome regulatory particle, base subcomplex (sensu Eukaryota) ? ATPase activity ?  0  BLAST| PF13\_0063 ? 26S proteasome regulatory subunit 7, putative  nucleotide binding (IEA) ? endopeptidase activity ? ATP binding (IEA) ? nucleus (IEA) ? cytoplasm (IEA) ? proteasome regulatory particle (sensu Eukaryota) ? ubiquitin-dependent protein catabolism ? hydrolase activity (IEA) ? ATPase activity (IEA) ? nucleoside-triphosphatase activity (IEA) ? protein catabolism (IEA) ?  YKL145W ? endopeptidase activity ? ubiquitin-dependent protein catabolism ? proteasome regulatory particle, base subcomplex (sensu Eukaryota) ? ATPase activity ?  0  BLAST| PFL2345c ? tat-binding protein homolog  nucleotide binding (IEA) ? proteasome complex (sensu Eukaryota) ? transcription cofactor activity ? ATP binding (IEA) ? nucleus (IEA) ? cytoplasm (IEA) ? ubiquitin-dependent protein catabolism ? hydrolase activity (IEA) ? ATPase activity ? nucleoside-triphosphatase activity (IEA) ? nucleotide kinase activity (IEA) ? protein catabolism (IEA) ?  YGL048C ? endopeptidase activity ? nucleus ? ubiquitin-dependent protein catabolism ? proteasome regulatory particle, base subcomplex (sensu Eukaryota) ? ATPase activity ?  0  BLAST| MAL13P1.343 ? proteasome regulatory subunit, putative  proteasome regulatory particle (sensu Eukaryota) ? ubiquitin-dependent protein catabolism ?  YFR004W ? endopeptidase activity ? nucleus ? ubiquitin-dependent protein catabolism ? proteasome regulatory particle, lid subcomplex (sensu Eukaryota) ?  0  BLAST | | | | | | | | | | | | | | | | | | | | |

## Cluster Pair #9: 3 gene pairs.

|  |  |  |  |  |  |  |  |  |  |  |  |
| --- | --- | --- | --- | --- | --- | --- | --- | --- | --- | --- | --- |
| P.falciparum S.cerevisiae Blast evalue|  |  |  |  |  |  |  |  |  | | --- | --- | --- | --- | --- | --- | --- | --- | --- | | PF13\_0096 ? Ubiquitin Carboxyl-terminal Hydrolase-like zinc finger protein  cysteine-type endopeptidase activity (IEA) ? ubiquitin thiolesterase activity ? ubiquitin-dependent protein catabolism ?  YFR005C ? nuclear mRNA splicing, via spliceosome ? nucleus ? RNA splicing factor activity, transesterification mechanism ?  6e-32  BLAST| PFB0450w ? Sec61-gamma subunit of protein translocation complex, putative  protein targeting (IEA) ? SRP-dependent cotranslational protein-membrane targeting, translocation ? intracellular protein transport (IEA) ? protein transporter activity ? protein transport (IEA) ? protein translocase activity (IEA) ? membrane ?  YDR086C ? translocon complex ? endoplasmic reticulum membrane ? SRP-dependent cotranslational protein-membrane targeting, translocation ? posttranslational protein-membrane targeting ? protein transporter activity ? protein secretion ? posttranslational protein membrane targeting, translocation ? Sec complex-associated translocon complex ?  8e-11  BLAST| PFI0740c ? ubiquitin conjugating enzyme, putative  protein modification (IEA) ? ubiquitin cycle (IEA) ? small protein activating enzyme activity (IEA) ?  YDL064W ? mitotic spindle elongation ? G2/M transition of mitotic cell cycle ? nucleus ? protein sumoylation ? SUMO conjugating enzyme activity ?  0  BLAST | | | | | | | | | | | |

## Cluster Pair #10: 5 gene pairs.

|  |  |  |  |  |  |  |  |  |  |  |  |  |  |  |  |  |  |
| --- | --- | --- | --- | --- | --- | --- | --- | --- | --- | --- | --- | --- | --- | --- | --- | --- | --- |
| P.falciparum S.cerevisiae Blast evalue|  |  |  |  |  |  |  |  |  |  |  |  |  |  |  | | --- | --- | --- | --- | --- | --- | --- | --- | --- | --- | --- | --- | --- | --- | --- | | MAL13P1.144 ? hypothetical protein   YOR260W ? translation initiation factor activity ? guanyl-nucleotide exchange factor activity ? eukaryotic translation initiation factor 2B complex ? regulation of translational initiation ?  9e-06  BLAST| MAL8P1.125 ? tyrosyl-tRNA synthetase, putative  tRNA ligase activity (IEA) ? tyrosine-tRNA ligase activity ? ATP binding (IEA) ? tRNA aminoacylation for protein translation (IEA) ? tyrosyl-tRNA aminoacylation ?  YGR185C ? tyrosine-tRNA ligase activity ? nucleus ? cytoplasm ? tyrosyl-tRNA aminoacylation ?  1e-27  BLAST| PF13\_0179 ? isoleucine--tRNA ligase, putative  tRNA ligase activity (IEA) ? isoleucine-tRNA ligase activity (IEA) ? ATP binding (IEA) ? tRNA aminoacylation for protein translation (IEA) ? isoleucyl-tRNA aminoacylation (IEA) ?  YBL076C ? isoleucine-tRNA ligase activity ? cytosol ? protein biosynthesis ?  0  BLAST| PF11\_0051 ? phenylalanine -- tRNA ligase, putative  phenylalanine-tRNA ligase activity ? ATP binding (IEA) ? cytoplasm (IEA) ? phenylalanyl-tRNA aminoacylation ? phenylalanine-tRNA ligase complex ?  YLR060W ? phenylalanine-tRNA ligase activity ? cytoplasm ? phenylalanyl-tRNA aminoacylation ? phenylalanine-tRNA ligase complex ?  0  BLAST| PF13\_0262 ? lysine--tRNA ligase  nucleic acid binding (IEA) ? tRNA ligase activity (IEA) ? lysine-tRNA ligase activity ? ATP binding ? cytoplasm (IEA) ? tRNA aminoacylation for protein translation (IEA) ? lysyl-tRNA aminoacylation ?  YDR037W ? lysine-tRNA ligase activity ? cytoplasm ? lysyl-tRNA aminoacylation ?  0  BLAST | | | | | | | | | | | | | | | | | |

## Cluster Pair #11: 8 gene pairs.

|  |  |  |  |  |  |  |  |  |  |  |  |  |  |  |  |  |  |  |  |  |  |  |  |  |  |  |
| --- | --- | --- | --- | --- | --- | --- | --- | --- | --- | --- | --- | --- | --- | --- | --- | --- | --- | --- | --- | --- | --- | --- | --- | --- | --- | --- |
| P.falciparum S.cerevisiae Blast evalue|  |  |  |  |  |  |  |  |  |  |  |  |  |  |  |  |  |  |  |  |  |  |  |  | | --- | --- | --- | --- | --- | --- | --- | --- | --- | --- | --- | --- | --- | --- | --- | --- | --- | --- | --- | --- | --- | --- | --- | --- | | PF14\_0218 ? actin, putative  structural constituent of cytoskeleton ? protein binding (IEA) ? actin filament ? exocytosis ? endocytosis ? cytoskeleton organization and biogenesis ?  YFL039C ? mitochondrion inheritance ? vacuole inheritance ? histone acetyltransferase complex ? establishment of mitotic spindle orientation ? contractile ring (sensu Saccharomyces) ? SWR1 complex ? cytokinesis ? cytokinesis, contractile ring contraction ? chronological cell aging ? structural constituent of cytoskeleton ? actin filament ? DNA repair ? regulation of transcription from RNA polymerase II promoter ? exocytosis ? endocytosis ? response to osmotic stress ? response to oxidative stress ? cell wall organization and biogenesis ? budding cell isotropic bud growth ? protein secretion ? histone acetylation ? actin filament reorganization during cell cycle ? vesicle transport along actin filament ? sporulation (sensu Fungi) ? establishment of cell polarity (sensu Fungi) ? actin cortical patch ? actin cable ? INO80 complex ? TIP60 histone acetyltransferase complex ?  7e-28  BLAST| PF13\_0214 ? elongation factor 1-gamma, putative  translation elongation factor activity ? glutathione transferase activity ? eukaryotic translation elongation factor 1 complex ? translational elongation ? \*\* also with: YKL081W, clust.pair #25 YPL048W ? translation elongation factor activity ? cytosolic ribosome (sensu Eukaryota) ? regulation of translational elongation ?  9e-26  BLAST| PF10\_0086 ? adenylate kinase, putative  adenylate kinase activity ? ATP binding (IEA) ? mitochondrion ? nucleobase, nucleoside, nucleotide and nucleic acid metabolism (IEA) ? phosphotransferase activity, phosphate group as acceptor (IEA) ? nucleotide kinase activity (IEA) ?  YDR226W ? adenylate kinase activity ? cytoplasm ? mitochondrion ? mitochondrial intermembrane space ? ADP biosynthesis ? nucleotide metabolism ?  0  BLAST| PFI0755c ? 6-phosphofructokinase, putative  catalytic activity (IEA) ? 6-phosphofructokinase activity (IEA) ? ATP binding (IEA) ? 6-phosphofructokinase complex (IEA) ? glycolysis (IEA) ? metabolism (IEA) ? diphosphate-fructose-6-phosphate 1-phosphotransferase activity (IEA) ? \*\* also with: YMR205C, clust.pair #11 YGR240C ? 6-phosphofructokinase activity ? cytoplasm ? 6-phosphofructokinase complex ? glycolysis ?  0.008  BLAST| PFI0755c ? 6-phosphofructokinase, putative  catalytic activity (IEA) ? 6-phosphofructokinase activity (IEA) ? ATP binding (IEA) ? 6-phosphofructokinase complex (IEA) ? glycolysis (IEA) ? metabolism (IEA) ? diphosphate-fructose-6-phosphate 1-phosphotransferase activity (IEA) ? \*\* also with: YGR240C, clust.pair #11 YMR205C ? 6-phosphofructokinase activity ? cytoplasm ? 6-phosphofructokinase complex ? glycolysis ?  0.03  BLAST| MAL8P1.105 ? hypothetical protein   YCL045C ? endoplasmic reticulum ?  4e-08  BLAST| PF07\_0077 ? hypothetical protein  protein binding (IEA) ?  YFL039C ? mitochondrion inheritance ? vacuole inheritance ? histone acetyltransferase complex ? establishment of mitotic spindle orientation ? contractile ring (sensu Saccharomyces) ? SWR1 complex ? cytokinesis ? cytokinesis, contractile ring contraction ? chronological cell aging ? structural constituent of cytoskeleton ? actin filament ? DNA repair ? regulation of transcription from RNA polymerase II promoter ? exocytosis ? endocytosis ? response to osmotic stress ? response to oxidative stress ? cell wall organization and biogenesis ? budding cell isotropic bud growth ? protein secretion ? histone acetylation ? actin filament reorganization during cell cycle ? vesicle transport along actin filament ? sporulation (sensu Fungi) ? establishment of cell polarity (sensu Fungi) ? actin cortical patch ? actin cable ? INO80 complex ? TIP60 histone acetyltransferase complex ?  1e-09  BLAST| PFL2305w ? hypothetical protein, conserved  methyltransferase activity (IEA) ? S-adenosylmethionine-dependent methyltransferase activity (IEA) ?  YOR239W ? actin filament ? S-adenosylmethionine-dependent methyltransferase activity ? actin cytoskeleton organization and biogenesis ? protein binding, bridging ? actin filament binding ?  2e-26  BLAST | | | | | | | | | | | | | | | | | | | | | | | | | | |

## Cluster Pair #12: 5 gene pairs.

|  |  |  |  |  |  |  |  |  |  |  |  |  |  |  |  |  |  |
| --- | --- | --- | --- | --- | --- | --- | --- | --- | --- | --- | --- | --- | --- | --- | --- | --- | --- |
| P.falciparum S.cerevisiae Blast evalue|  |  |  |  |  |  |  |  |  |  |  |  |  |  |  | | --- | --- | --- | --- | --- | --- | --- | --- | --- | --- | --- | --- | --- | --- | --- | | PF14\_0378 ? triose-phosphate isomerase  triose-phosphate isomerase activity ? gluconeogenesis ? glycolysis ? pentose-phosphate shunt ? fatty acid biosynthesis ? metabolism (IEA) ?  YDR050C ? triose-phosphate isomerase activity ? cytoplasm ? glycolysis ?  0  BLAST| PFI1105w ? Phosphoglycerate kinase  phosphoglycerate kinase activity (IEA) ? glycolysis (IEA) ?  YCR012W ? phosphoglycerate kinase activity ? cytoplasm ? mitochondrion ? gluconeogenesis ? glycolysis ?  0  BLAST| PF14\_0598 ? glyceraldehyde-3-phosphate dehydrogenase  glyceraldehyde-3-phosphate dehydrogenase (phosphorylating) activity ? mitochondrion ? glucose metabolism (IEA) ? gluconeogenesis ? glycolysis ? glyceraldehyde-3-phosphate dehydrogenase activity (IEA) ? NAD binding (IEA) ? \*\* also with: YGR192C, clust.pair #12 \*\* also with: YJR009C, clust.pair #12 YJL052W ? glyceraldehyde-3-phosphate dehydrogenase (phosphorylating) activity ? cytoplasm ? lipid particle ? gluconeogenesis ? glycolysis ? cell wall (sensu Fungi) ?  0  BLAST| PF14\_0598 ? glyceraldehyde-3-phosphate dehydrogenase  glyceraldehyde-3-phosphate dehydrogenase (phosphorylating) activity ? mitochondrion ? glucose metabolism (IEA) ? gluconeogenesis ? glycolysis ? glyceraldehyde-3-phosphate dehydrogenase activity (IEA) ? NAD binding (IEA) ? \*\* also with: YJL052W, clust.pair #12 \*\* also with: YJR009C, clust.pair #12 YGR192C ? glyceraldehyde-3-phosphate dehydrogenase (phosphorylating) activity ? cytoplasm ? mitochondrion ? lipid particle ? gluconeogenesis ? glycolysis ? cell wall (sensu Fungi) ?  0  BLAST| PF14\_0598 ? glyceraldehyde-3-phosphate dehydrogenase  glyceraldehyde-3-phosphate dehydrogenase (phosphorylating) activity ? mitochondrion ? glucose metabolism (IEA) ? gluconeogenesis ? glycolysis ? glyceraldehyde-3-phosphate dehydrogenase activity (IEA) ? NAD binding (IEA) ? \*\* also with: YJL052W, clust.pair #12 \*\* also with: YGR192C, clust.pair #12 YJR009C ? glyceraldehyde-3-phosphate dehydrogenase (phosphorylating) activity ? cytoplasm ? lipid particle ? gluconeogenesis ? glycolysis ? cell wall (sensu Fungi) ?  0  BLAST | | | | | | | | | | | | | | | | | |

## Cluster Pair #13: 6 gene pairs.

|  |  |  |  |  |  |  |  |  |  |  |  |  |  |  |  |  |  |  |  |  |
| --- | --- | --- | --- | --- | --- | --- | --- | --- | --- | --- | --- | --- | --- | --- | --- | --- | --- | --- | --- | --- |
| P.falciparum S.cerevisiae Blast evalue|  |  |  |  |  |  |  |  |  |  |  |  |  |  |  |  |  |  | | --- | --- | --- | --- | --- | --- | --- | --- | --- | --- | --- | --- | --- | --- | --- | --- | --- | --- | | PFL0740c ? 10 kd chaperonin, putative  ATP binding (IEA) ? protein folding (IEA) ? response to unfolded protein ? response to heat ? unfolded protein binding ?  YOR020C ? mitochondrion ? mitochondrial matrix ? protein folding ? unfolded protein binding ?  4e-17  BLAST| PF10\_0153 ? hsp60  protein binding (IEA) ? ATP binding (IEA) ? mitochondrion ? protein folding ? protein targeting to mitochondrion ? response to unfolded protein ? ATPase activity, coupled ? cellular protein metabolism (IEA) ? unfolded protein binding (IEA) ?  YLR259C ? single-stranded DNA binding ? mitochondrion ? protein folding ? mitochondrial matrix protein import ? mitochondrial nucleoid ?  0  BLAST| PFB0125c ? hypothetical protein  nucleic acid binding (IEA) ?  YJL020C ? myosin I binding ? actin cytoskeleton organization and biogenesis ? actin cortical patch ?  0.0004  BLAST| PF11\_0188 ? heat shock protein 90, putative  ATP binding ? mitochondrion ? protein folding (IEA) ? response to unfolded protein ? response to heat ? ATPase activity ? unfolded protein binding (IEA) ? \*\* also with: YMR186W, clust.pair #13 YPL240C ? cytoplasm ? 'de novo' protein folding ? response to stress ? response to osmotic stress ? protein refolding ? ATPase activity, coupled ? proteasome assembly ? unfolded protein binding ?  7e-37  BLAST| PF11\_0188 ? heat shock protein 90, putative  ATP binding ? mitochondrion ? protein folding (IEA) ? response to unfolded protein ? response to heat ? ATPase activity ? unfolded protein binding (IEA) ? \*\* also with: YPL240C, clust.pair #13 YMR186W ? telomere maintenance ? cytoplasm ? mitochondrion ? protein folding ? 'de novo' protein folding ? response to stress ? protein refolding ? ATPase activity, coupled ? proteasome assembly ? unfolded protein binding ?  5e-35  BLAST| PF11\_0351 ? heat shock protein hsp70 homologue  ATP binding (IEA) ? response to unfolded protein ? response to heat ?  YJR045C ? presequence translocase-associated import motor ? mitochondrion ? mitochondrial inner membrane ? protein folding ? protein transporter activity ? ATPase activity ? mitochondrial matrix protein import ? enzyme regulator activity ? protein refolding ? mitochondrial nucleoid ?  0  BLAST | | | | | | | | | | | | | | | | | | | | |

## Cluster Pair #14: 57 gene pairs.

|  |  |  |  |  |  |  |  |  |  |  |  |  |  |  |  |  |  |  |  |  |  |  |  |  |  |  |  |  |  |  |  |  |  |  |  |  |  |  |  |  |  |  |  |  |  |  |  |  |  |  |  |  |  |  |  |  |  |  |  |  |  |  |  |  |  |  |  |  |  |  |  |  |  |  |  |  |  |  |  |  |  |  |  |  |  |  |  |  |  |  |  |  |  |  |  |  |  |  |  |  |  |  |  |  |  |  |  |  |  |  |  |  |  |  |  |  |  |  |  |  |  |  |  |  |  |  |  |  |  |  |  |  |  |  |  |  |  |  |  |  |  |  |  |  |  |  |  |  |  |  |  |  |  |  |  |  |  |  |  |  |  |  |  |  |  |  |  |  |  |  |  |  |  |
| --- | --- | --- | --- | --- | --- | --- | --- | --- | --- | --- | --- | --- | --- | --- | --- | --- | --- | --- | --- | --- | --- | --- | --- | --- | --- | --- | --- | --- | --- | --- | --- | --- | --- | --- | --- | --- | --- | --- | --- | --- | --- | --- | --- | --- | --- | --- | --- | --- | --- | --- | --- | --- | --- | --- | --- | --- | --- | --- | --- | --- | --- | --- | --- | --- | --- | --- | --- | --- | --- | --- | --- | --- | --- | --- | --- | --- | --- | --- | --- | --- | --- | --- | --- | --- | --- | --- | --- | --- | --- | --- | --- | --- | --- | --- | --- | --- | --- | --- | --- | --- | --- | --- | --- | --- | --- | --- | --- | --- | --- | --- | --- | --- | --- | --- | --- | --- | --- | --- | --- | --- | --- | --- | --- | --- | --- | --- | --- | --- | --- | --- | --- | --- | --- | --- | --- | --- | --- | --- | --- | --- | --- | --- | --- | --- | --- | --- | --- | --- | --- | --- | --- | --- | --- | --- | --- | --- | --- | --- | --- | --- | --- | --- | --- | --- | --- | --- | --- | --- | --- | --- | --- | --- | --- |
| P.falciparum S.cerevisiae Blast evalue|  |  |  |  |  |  |  |  |  |  |  |  |  |  |  |  |  |  |  |  |  |  |  |  |  |  |  |  |  |  |  |  |  |  |  |  |  |  |  |  |  |  |  |  |  |  |  |  |  |  |  |  |  |  |  |  |  |  |  |  |  |  |  |  |  |  |  |  |  |  |  |  |  |  |  |  |  |  |  |  |  |  |  |  |  |  |  |  |  |  |  |  |  |  |  |  |  |  |  |  |  |  |  |  |  |  |  |  |  |  |  |  |  |  |  |  |  |  |  |  |  |  |  |  |  |  |  |  |  |  |  |  |  |  |  |  |  |  |  |  |  |  |  |  |  |  |  |  |  |  |  |  |  |  |  |  |  |  |  |  |  |  |  |  |  |  |  |  |  |  |  | | --- | --- | --- | --- | --- | --- | --- | --- | --- | --- | --- | --- | --- | --- | --- | --- | --- | --- | --- | --- | --- | --- | --- | --- | --- | --- | --- | --- | --- | --- | --- | --- | --- | --- | --- | --- | --- | --- | --- | --- | --- | --- | --- | --- | --- | --- | --- | --- | --- | --- | --- | --- | --- | --- | --- | --- | --- | --- | --- | --- | --- | --- | --- | --- | --- | --- | --- | --- | --- | --- | --- | --- | --- | --- | --- | --- | --- | --- | --- | --- | --- | --- | --- | --- | --- | --- | --- | --- | --- | --- | --- | --- | --- | --- | --- | --- | --- | --- | --- | --- | --- | --- | --- | --- | --- | --- | --- | --- | --- | --- | --- | --- | --- | --- | --- | --- | --- | --- | --- | --- | --- | --- | --- | --- | --- | --- | --- | --- | --- | --- | --- | --- | --- | --- | --- | --- | --- | --- | --- | --- | --- | --- | --- | --- | --- | --- | --- | --- | --- | --- | --- | --- | --- | --- | --- | --- | --- | --- | --- | --- | --- | --- | --- | --- | --- | --- | --- | --- | --- | --- | --- | | PF13\_0014 ? 40S ribosomal protein S7 homologue, putative  structural constituent of ribosome ? intracellular (IEA) ? ribosome (IEA) ? cytosolic small ribosomal subunit (sensu Eukaryota) ? protein biosynthesis ? \*\* also with: YOR096W, clust.pair #14 YNL096C ? structural constituent of ribosome ? small nucleolar ribonucleoprotein complex ? cytosolic small ribosomal subunit (sensu Eukaryota) ? protein biosynthesis ?  5e-31  BLAST| PF13\_0014 ? 40S ribosomal protein S7 homologue, putative  structural constituent of ribosome ? intracellular (IEA) ? ribosome (IEA) ? cytosolic small ribosomal subunit (sensu Eukaryota) ? protein biosynthesis ? \*\* also with: YNL096C, clust.pair #14 YOR096W ? structural constituent of ribosome ? small nucleolar ribonucleoprotein complex ? cytosolic small ribosomal subunit (sensu Eukaryota) ? protein biosynthesis ?  6e-29  BLAST| PF13\_0224 ? 60S ribosomal subunit protein L18, putative  structural constituent of ribosome ? intracellular (IEA) ? ribosome (IEA) ? cytosolic large ribosomal subunit (sensu Eukaryota) ? protein biosynthesis ? \*\* also with: YOR312C, clust.pair #14 YMR242C ? structural constituent of ribosome ? cytosolic large ribosomal subunit (sensu Eukaryota) ? protein biosynthesis ?  4e-34  BLAST| PF13\_0224 ? 60S ribosomal subunit protein L18, putative  structural constituent of ribosome ? intracellular (IEA) ? ribosome (IEA) ? cytosolic large ribosomal subunit (sensu Eukaryota) ? protein biosynthesis ? \*\* also with: YMR242C, clust.pair #14 YOR312C ? structural constituent of ribosome ? cytosolic large ribosomal subunit (sensu Eukaryota) ? protein biosynthesis ? ribosome biogenesis and assembly ?  4e-34  BLAST| PF14\_0027 ? ribosomal S27a, putative  structural constituent of ribosome ? intracellular (IEA) ? ribosome (IEA) ? cytosolic small ribosomal subunit (sensu Eukaryota) ? protein biosynthesis (IEA) ? protein modification (IEA) ?  YLR167W ? ribosomal small subunit assembly and maintenance ? structural constituent of ribosome ? cytoplasm ? cytosolic small ribosomal subunit (sensu Eukaryota) ? protein biosynthesis ? protein ubiquitination ? protein tag ? ribosome biogenesis and assembly ?  1e-12  BLAST| PF10\_0038 ? ribosomal protein S20e, putative  structural constituent of ribosome ? intracellular (IEA) ? ribosome (IEA) ? cytosolic small ribosomal subunit (sensu Eukaryota) ? protein biosynthesis ? small ribosomal subunit (IEA) ?  YHL015W ? structural constituent of ribosome ? cytosolic small ribosomal subunit (sensu Eukaryota) ? protein biosynthesis ?  3e-25  BLAST| PF13\_0132 ? 60S ribosomal protein L23a, putative  structural constituent of ribosome ? cytosolic large ribosomal subunit (sensu Eukaryota) ? protein biosynthesis ? rRNA binding ?  YOL127W ? ribosomal large subunit assembly and maintenance ? RNA binding ? structural constituent of ribosome ? cytosolic large ribosomal subunit (sensu Eukaryota) ? protein biosynthesis ?  2e-21  BLAST| PFD1055w ? ribosomal protein S19s, putative  structural constituent of ribosome ? intracellular (IEA) ? ribosome ? protein biosynthesis ? \*\* also with: YOL121C, clust.pair #14 YNL302C ? ribosomal small subunit-nucleus export ? structural constituent of ribosome ? cytosolic small ribosomal subunit (sensu Eukaryota) ? rRNA processing ? protein biosynthesis ? ribosomal small subunit biogenesis ?  3e-20  BLAST| PFD1055w ? ribosomal protein S19s, putative  structural constituent of ribosome ? intracellular (IEA) ? ribosome ? protein biosynthesis ? \*\* also with: YNL302C, clust.pair #14 YOL121C ? ribosomal small subunit-nucleus export ? telomere maintenance ? structural constituent of ribosome ? cytosolic small ribosomal subunit (sensu Eukaryota) ? rRNA processing ? protein biosynthesis ? ribosomal small subunit biogenesis ?  3e-20  BLAST| PFE0810c ? 40S ribosomal subunit protein S14, putative  RNA binding ? structural constituent of ribosome (IEA) ? intracellular (IEA) ? ribosome (IEA) ? cytosolic small ribosomal subunit (sensu Eukaryota) ? protein biosynthesis ? \*\* also with: YJL191W, clust.pair #14 YCR031C ? ribosomal small subunit assembly and maintenance ? telomere maintenance ? RNA binding ? structural constituent of ribosome ? small nucleolar ribonucleoprotein complex ? cytosolic small ribosomal subunit (sensu Eukaryota) ? protein biosynthesis ? processing of 20S pre-rRNA ?  5.60519e-45  BLAST| PFE0810c ? 40S ribosomal subunit protein S14, putative  RNA binding ? structural constituent of ribosome (IEA) ? intracellular (IEA) ? ribosome (IEA) ? cytosolic small ribosomal subunit (sensu Eukaryota) ? protein biosynthesis ? \*\* also with: YCR031C, clust.pair #14 YJL191W ? ribosomal small subunit assembly and maintenance ? RNA binding ? structural constituent of ribosome ? small nucleolar ribonucleoprotein complex ? cytosolic small ribosomal subunit (sensu Eukaryota) ? protein biosynthesis ? processing of 20S pre-rRNA ?  5.60519e-45  BLAST| PFI0190w ? ribosomal protein L32, putative  structural constituent of ribosome (IEA) ? intracellular (IEA) ? ribosome (IEA) ? protein biosynthesis (IEA) ?  YBL092W ? structural constituent of ribosome ? cytosolic large ribosomal subunit (sensu Eukaryota) ? protein biosynthesis ?  6e-22  BLAST| PF13\_0213 ? 60S ribosomal subunit protein L6e, putative  structural constituent of ribosome ? intracellular (IEA) ? ribosome (IEA) ? cytosolic large ribosomal subunit (sensu Eukaryota) ? protein biosynthesis ? \*\* also with: YML073C, clust.pair #14 YLR448W ? ribosomal large subunit assembly and maintenance ? RNA binding ? structural constituent of ribosome ? cytosolic large ribosomal subunit (sensu Eukaryota) ? protein biosynthesis ?  7e-16  BLAST| PF13\_0213 ? 60S ribosomal subunit protein L6e, putative  structural constituent of ribosome ? intracellular (IEA) ? ribosome (IEA) ? cytosolic large ribosomal subunit (sensu Eukaryota) ? protein biosynthesis ? \*\* also with: YLR448W, clust.pair #14 YML073C ? ribosomal large subunit assembly and maintenance ? RNA binding ? structural constituent of ribosome ? cytosolic large ribosomal subunit (sensu Eukaryota) ? protein biosynthesis ?  1e-14  BLAST| PFB0455w ? ribosomal L37ae protein, putative  structural constituent of ribosome ? intracellular (IEA) ? mitochondrion ? ribosome (IEA) ? cytosolic large ribosomal subunit (sensu Eukaryota) ? protein biosynthesis ? \*\* also with: YPR043W, clust.pair #14 YJR094W-A ? structural constituent of ribosome ? cytosolic large ribosomal subunit (sensu Eukaryota) ? protein biosynthesis ?  9e-26  BLAST| PFB0455w ? ribosomal L37ae protein, putative  structural constituent of ribosome ? intracellular (IEA) ? mitochondrion ? ribosome (IEA) ? cytosolic large ribosomal subunit (sensu Eukaryota) ? protein biosynthesis ? \*\* also with: YJR094W-A, clust.pair #14 YPR043W ? structural constituent of ribosome ? cytosolic large ribosomal subunit (sensu Eukaryota) ? protein biosynthesis ?  9e-26  BLAST| PF10\_0187 ? ribosomal protein L30e, putative  structural constituent of ribosome ? cytosolic large ribosomal subunit (sensu Eukaryota) ? protein biosynthesis ?  YGL030W ? structural constituent of ribosome ? cytoplasm ? cytosolic large ribosomal subunit (sensu Eukaryota) ? rRNA processing ? protein biosynthesis ? negative regulation of protein biosynthesis ? negative regulation of nuclear mRNA splicing, via spliceosome ?  2e-26  BLAST| PFB0830w ? Ribosomal protein S26e, putative  structural constituent of ribosome ? intracellular (IEA) ? mitochondrion ? ribosome (IEA) ? cytosolic small ribosomal subunit (sensu Eukaryota) ? protein biosynthesis ? \*\* also with: YGL189C, clust.pair #14 YER131W ? structural constituent of ribosome ? cytosolic small ribosomal subunit (sensu Eukaryota) ? protein biosynthesis ?  2e-26  BLAST| PFB0830w ? Ribosomal protein S26e, putative  structural constituent of ribosome ? intracellular (IEA) ? mitochondrion ? ribosome (IEA) ? cytosolic small ribosomal subunit (sensu Eukaryota) ? protein biosynthesis ? \*\* also with: YER131W, clust.pair #14 YGL189C ? structural constituent of ribosome ? cytosolic small ribosomal subunit (sensu Eukaryota) ? protein biosynthesis ?  2e-26  BLAST| PF07\_0080 ? 40S ribosomal protein S10, putative  structural constituent of ribosome ? cytosolic small ribosomal subunit (sensu Eukaryota) ? protein biosynthesis ? \*\* also with: YOR293W, clust.pair #14 YMR230W ? structural constituent of ribosome ? cytosolic small ribosomal subunit (sensu Eukaryota) ? protein biosynthesis ?  1e-20  BLAST| PF07\_0080 ? 40S ribosomal protein S10, putative  structural constituent of ribosome ? cytosolic small ribosomal subunit (sensu Eukaryota) ? protein biosynthesis ? \*\* also with: YMR230W, clust.pair #14 YOR293W ? structural constituent of ribosome ? cytosolic small ribosomal subunit (sensu Eukaryota) ? protein biosynthesis ?  2e-20  BLAST| PFC0295c ? 40S ribosomal protein S12, putative  structural constituent of ribosome ? intracellular (IEA) ? ribosome (IEA) ? cytosolic small ribosomal subunit (sensu Eukaryota) ? protein biosynthesis ?  YOR369C ? structural constituent of ribosome ? cytosolic small ribosomal subunit (sensu Eukaryota) ? protein biosynthesis ?  9e-19  BLAST| PFC0300c ? 60S ribosomal protein L7, putative  structural constituent of ribosome ? intracellular (IEA) ? ribosome (IEA) ? cytosolic large ribosomal subunit (sensu Eukaryota) ? protein biosynthesis ? large ribosomal subunit (IEA) ? transcription regulator activity (IEA) ? \*\* also with: YPL198W, clust.pair #14 YGL076C ? structural constituent of ribosome ? cytosolic large ribosomal subunit (sensu Eukaryota) ? protein biosynthesis ?  1.00053e-42  BLAST| PFC0300c ? 60S ribosomal protein L7, putative  structural constituent of ribosome ? intracellular (IEA) ? ribosome (IEA) ? cytosolic large ribosomal subunit (sensu Eukaryota) ? protein biosynthesis ? large ribosomal subunit (IEA) ? transcription regulator activity (IEA) ? \*\* also with: YGL076C, clust.pair #14 YPL198W ? structural constituent of ribosome ? cytosolic large ribosomal subunit (sensu Eukaryota) ? protein biosynthesis ?  1.00053e-42  BLAST| PFE0185c ? 60S ribosomal subunit protein L31, putative  structural constituent of ribosome ? intracellular (IEA) ? ribosome (IEA) ? protein biosynthesis ? large ribosomal subunit ? \*\* also with: YDL075W, clust.pair #14 YLR406C ? structural constituent of ribosome ? cytosolic large ribosomal subunit (sensu Eukaryota) ? protein biosynthesis ?  5e-21  BLAST| PFE0185c ? 60S ribosomal subunit protein L31, putative  structural constituent of ribosome ? intracellular (IEA) ? ribosome (IEA) ? protein biosynthesis ? large ribosomal subunit ? \*\* also with: YLR406C, clust.pair #14 YDL075W ? structural constituent of ribosome ? cytosolic large ribosomal subunit (sensu Eukaryota) ? protein biosynthesis ?  9e-21  BLAST| PF13\_0045 ? 40S ribosomal protein S27, putative  structural constituent of ribosome ? intracellular (IEA) ? ribosome (IEA) ? cytosolic small ribosomal subunit (sensu Eukaryota) ? protein biosynthesis ? \*\* also with: YKL156W, clust.pair #14 YHR021C ? telomere maintenance ? structural constituent of ribosome ? cytosolic small ribosomal subunit (sensu Eukaryota) ? protein biosynthesis ?  5e-27  BLAST| PF13\_0045 ? 40S ribosomal protein S27, putative  structural constituent of ribosome ? intracellular (IEA) ? ribosome (IEA) ? cytosolic small ribosomal subunit (sensu Eukaryota) ? protein biosynthesis ? \*\* also with: YHR021C, clust.pair #14 YKL156W ? structural constituent of ribosome ? cytoplasm ? cytosolic small ribosomal subunit (sensu Eukaryota) ? protein biosynthesis ?  5e-27  BLAST| PF13\_0171 ? 60S ribosomal protein L23, putative  structural constituent of ribosome (IEA) ? intracellular (IEA) ? ribosome (IEA) ? protein biosynthesis (IEA) ? \*\* also with: YER117W, clust.pair #14 YBL087C ? structural constituent of ribosome ? cytosolic large ribosomal subunit (sensu Eukaryota) ? protein biosynthesis ?  0  BLAST| PF13\_0171 ? 60S ribosomal protein L23, putative  structural constituent of ribosome (IEA) ? intracellular (IEA) ? ribosome (IEA) ? protein biosynthesis (IEA) ? \*\* also with: YBL087C, clust.pair #14 YER117W ? structural constituent of ribosome ? cytosolic large ribosomal subunit (sensu Eukaryota) ? protein biosynthesis ? response to drug ?  0  BLAST| PF14\_0231 ? ribosomal protein L7a, putative  structural constituent of ribosome ? intracellular (IEA) ? ribosome (IEA) ? cytosolic large ribosomal subunit (sensu Eukaryota) ? protein biosynthesis ? ribonucleoprotein complex (IEA) ? ribosome biogenesis and assembly (IEA) ? \*\* also with: YLL045C, clust.pair #14 YHL033C ? structural constituent of ribosome ? cytosolic large ribosomal subunit (sensu Eukaryota) ? protein biosynthesis ?  2.00386e-43  BLAST| PF14\_0231 ? ribosomal protein L7a, putative  structural constituent of ribosome ? intracellular (IEA) ? ribosome (IEA) ? cytosolic large ribosomal subunit (sensu Eukaryota) ? protein biosynthesis ? ribonucleoprotein complex (IEA) ? ribosome biogenesis and assembly (IEA) ? \*\* also with: YHL033C, clust.pair #14 YLL045C ? structural constituent of ribosome ? cytosolic large ribosomal subunit (sensu Eukaryota) ? protein biosynthesis ?  2.99878e-43  BLAST| PF11\_0272 ? ribosomal protein S18, putative  RNA binding (IEA) ? structural constituent of ribosome ? intracellular (IEA) ? ribosome (IEA) ? protein biosynthesis ? small ribosomal subunit ? \*\* also with: YML026C, clust.pair #14 YDR450W ? telomere maintenance ? structural constituent of ribosome ? mitochondrion ? cytosolic small ribosomal subunit (sensu Eukaryota) ? protein biosynthesis ?  0  BLAST| PF11\_0272 ? ribosomal protein S18, putative  RNA binding (IEA) ? structural constituent of ribosome ? intracellular (IEA) ? ribosome (IEA) ? protein biosynthesis ? small ribosomal subunit ? \*\* also with: YDR450W, clust.pair #14 YML026C ? structural constituent of ribosome ? mitochondrion ? cytosolic small ribosomal subunit (sensu Eukaryota) ? protein biosynthesis ?  0  BLAST| PF14\_0579 ? ribosomal protein L27, putative  structural constituent of ribosome ? intracellular (IEA) ? ribosome (IEA) ? cytosolic large ribosomal subunit (sensu Eukaryota) ? protein biosynthesis ? \*\* also with: YDR471W, clust.pair #14 YHR010W ? structural constituent of ribosome ? cytosolic large ribosomal subunit (sensu Eukaryota) ? protein biosynthesis ?  1e-16  BLAST| PF14\_0579 ? ribosomal protein L27, putative  structural constituent of ribosome ? intracellular (IEA) ? ribosome (IEA) ? cytosolic large ribosomal subunit (sensu Eukaryota) ? protein biosynthesis ? \*\* also with: YHR010W, clust.pair #14 YDR471W ? structural constituent of ribosome ? cytosolic large ribosomal subunit (sensu Eukaryota) ? protein biosynthesis ?  5e-16  BLAST| PF08\_0076 ? 40S ribosomal protein S16, putative  structural constituent of ribosome ? intracellular (IEA) ? ribosome (IEA) ? cytosolic small ribosomal subunit (sensu Eukaryota) ? protein biosynthesis ? \*\* also with: YMR143W, clust.pair #14 YDL083C ? telomere maintenance ? structural constituent of ribosome ? cytosolic small ribosomal subunit (sensu Eukaryota) ? protein biosynthesis ?  9.80909e-45  BLAST| PF08\_0076 ? 40S ribosomal protein S16, putative  structural constituent of ribosome ? intracellular (IEA) ? ribosome (IEA) ? cytosolic small ribosomal subunit (sensu Eukaryota) ? protein biosynthesis ? \*\* also with: YDL083C, clust.pair #14 YMR143W ? telomere maintenance ? structural constituent of ribosome ? cytosolic small ribosomal subunit (sensu Eukaryota) ? protein biosynthesis ?  9.80909e-45  BLAST| PF14\_0240 ? ribosomal protein L21e, putative  structural constituent of ribosome ? mitochondrion ? cytosolic large ribosomal subunit (sensu Eukaryota) ? protein biosynthesis ? \*\* also with: YPL079W, clust.pair #14 YBR191W ? structural constituent of ribosome ? cytosolic large ribosomal subunit (sensu Eukaryota) ? protein biosynthesis ?  4e-28  BLAST| PF14\_0240 ? ribosomal protein L21e, putative  structural constituent of ribosome ? mitochondrion ? cytosolic large ribosomal subunit (sensu Eukaryota) ? protein biosynthesis ? \*\* also with: YBR191W, clust.pair #14 YPL079W ? structural constituent of ribosome ? cytosolic large ribosomal subunit (sensu Eukaryota) ? protein biosynthesis ?  5e-28  BLAST| PFL2055w ? 40S ribosomal protein S17, putative  structural constituent of ribosome ? intracellular (IEA) ? mitochondrion ? ribosome (IEA) ? cytosolic small ribosomal subunit (sensu Eukaryota) ? protein biosynthesis ? \*\* also with: YML024W, clust.pair #14 YDR447C ? ribosomal small subunit assembly and maintenance ? telomere maintenance ? structural constituent of ribosome ? cytosolic small ribosomal subunit (sensu Eukaryota) ? protein biosynthesis ?  4e-33  BLAST| PFL2055w ? 40S ribosomal protein S17, putative  structural constituent of ribosome ? intracellular (IEA) ? mitochondrion ? ribosome (IEA) ? cytosolic small ribosomal subunit (sensu Eukaryota) ? protein biosynthesis ? \*\* also with: YDR447C, clust.pair #14 YML024W ? ribosomal small subunit assembly and maintenance ? telomere maintenance ? structural constituent of ribosome ? cytosolic small ribosomal subunit (sensu Eukaryota) ? protein biosynthesis ?  4e-33  BLAST| PF11\_0438 ? Ribosomal protein, putative  structural constituent of ribosome ? intracellular (IEA) ? ribosome (IEA) ? protein biosynthesis ? large ribosomal subunit ? \*\* also with: YPL143W, clust.pair #14 YOR234C ? structural constituent of ribosome ? cytosolic large ribosomal subunit (sensu Eukaryota) ? protein biosynthesis ?  1e-30  BLAST| PF11\_0438 ? Ribosomal protein, putative  structural constituent of ribosome ? intracellular (IEA) ? ribosome (IEA) ? protein biosynthesis ? large ribosomal subunit ? \*\* also with: YOR234C, clust.pair #14 YPL143W ? structural constituent of ribosome ? cytosolic large ribosomal subunit (sensu Eukaryota) ? protein biosynthesis ?  1e-30  BLAST| MAL13P1.92 ? 40S ribosomal protein S15, putative  structural constituent of ribosome ? intracellular (IEA) ? ribosome (IEA) ? cytosolic small ribosomal subunit (sensu Eukaryota) ? protein biosynthesis ? small ribosomal subunit (IEA) ?  YOL040C ? ribosomal small subunit-nucleus export ? structural constituent of ribosome ? cytosolic small ribosomal subunit (sensu Eukaryota) ? protein biosynthesis ?  5e-36  BLAST| PF13\_0228 ? 40S ribosomal subunit protein S6, putative  structural constituent of ribosome ? intracellular (IEA) ? ribosome (IEA) ? cytosolic small ribosomal subunit (sensu Eukaryota) ? protein biosynthesis ? \*\* also with: YPL090C, clust.pair #14 YBR181C ? structural constituent of ribosome ? small nucleolar ribonucleoprotein complex ? cytoplasm ? cytosolic small ribosomal subunit (sensu Eukaryota) ? protein biosynthesis ?  0  BLAST| PF13\_0228 ? 40S ribosomal subunit protein S6, putative  structural constituent of ribosome ? intracellular (IEA) ? ribosome (IEA) ? cytosolic small ribosomal subunit (sensu Eukaryota) ? protein biosynthesis ? \*\* also with: YBR181C, clust.pair #14 YPL090C ? structural constituent of ribosome ? small nucleolar ribonucleoprotein complex ? cytosolic small ribosomal subunit (sensu Eukaryota) ? protein biosynthesis ?  0  BLAST| PFC0535w ? 60S ribosomal protein L26, putative  structural constituent of ribosome ? intracellular (IEA) ? ribosome (IEA) ? cytosolic large ribosomal subunit (sensu Eukaryota) ? protein biosynthesis ? large ribosomal subunit (IEA) ? \*\* also with: YLR344W, clust.pair #14 YGR034W ? RNA binding ? structural constituent of ribosome ? cytosolic large ribosomal subunit (sensu Eukaryota) ? protein biosynthesis ?  2e-28  BLAST| PFC0535w ? 60S ribosomal protein L26, putative  structural constituent of ribosome ? intracellular (IEA) ? ribosome (IEA) ? cytosolic large ribosomal subunit (sensu Eukaryota) ? protein biosynthesis ? large ribosomal subunit (IEA) ? \*\* also with: YGR034W, clust.pair #14 YLR344W ? RNA binding ? structural constituent of ribosome ? cytosolic large ribosomal subunit (sensu Eukaryota) ? protein biosynthesis ?  1e-28  BLAST| PF13\_0268 ? ribosomal protein L17, putative  structural constituent of ribosome ? intracellular (IEA) ? ribosome (IEA) ? cytosolic large ribosomal subunit (sensu Eukaryota) ? protein biosynthesis ? large ribosomal subunit (IEA) ? \*\* also with: YKL180W, clust.pair #14 YJL177W ? structural constituent of ribosome ? cytosolic large ribosomal subunit (sensu Eukaryota) ? protein biosynthesis ?  3e-40  BLAST| PF13\_0268 ? ribosomal protein L17, putative  structural constituent of ribosome ? intracellular (IEA) ? ribosome (IEA) ? cytosolic large ribosomal subunit (sensu Eukaryota) ? protein biosynthesis ? large ribosomal subunit (IEA) ? \*\* also with: YJL177W, clust.pair #14 YKL180W ? structural constituent of ribosome ? cytoplasm ? cytosolic large ribosomal subunit (sensu Eukaryota) ? protein biosynthesis ?  4.00001e-40  BLAST| PFC0735w ? 40S ribosomal protein S15A, putative  structural constituent of ribosome ? intracellular (IEA) ? ribosome (IEA) ? cytosolic small ribosomal subunit (sensu Eukaryota) ? protein biosynthesis ? \*\* also with: YLR367W, clust.pair #14 YJL190C ? telomere maintenance ? structural constituent of ribosome ? cytosolic small ribosomal subunit (sensu Eukaryota) ? protein biosynthesis ?  0  BLAST| PFC0735w ? 40S ribosomal protein S15A, putative  structural constituent of ribosome ? intracellular (IEA) ? ribosome (IEA) ? cytosolic small ribosomal subunit (sensu Eukaryota) ? protein biosynthesis ? \*\* also with: YJL190C, clust.pair #14 YLR367W ? structural constituent of ribosome ? cytosolic small ribosomal subunit (sensu Eukaryota) ? protein biosynthesis ?  0  BLAST| PFC0775w ? 40S ribosomal protein S11, putative  nucleic acid binding (IEA) ? structural constituent of ribosome ? intracellular (IEA) ? ribosome (IEA) ? cytosolic small ribosomal subunit (sensu Eukaryota) ? protein biosynthesis ? \*\* also with: YDR025W, clust.pair #14 YBR048W ? ribosomal small subunit assembly and maintenance ? telomere maintenance ? structural constituent of ribosome ? cytosolic small ribosomal subunit (sensu Eukaryota) ? protein biosynthesis ? regulation of translational fidelity ?  0  BLAST| PFC0775w ? 40S ribosomal protein S11, putative  nucleic acid binding (IEA) ? structural constituent of ribosome ? intracellular (IEA) ? ribosome (IEA) ? cytosolic small ribosomal subunit (sensu Eukaryota) ? protein biosynthesis ? \*\* also with: YBR048W, clust.pair #14 YDR025W ? ribosomal small subunit assembly and maintenance ? structural constituent of ribosome ? cytosolic small ribosomal subunit (sensu Eukaryota) ? protein biosynthesis ? regulation of translational fidelity ?  0  BLAST| PFC1020c ? 40S ribosomal protein S3A, putative  structural constituent of ribosome ? intracellular (IEA) ? ribosome (IEA) ? cytosolic small ribosomal subunit (sensu Eukaryota) ? protein biosynthesis ? \*\* also with: YML063W, clust.pair #14 YLR441C ? structural constituent of ribosome ? cytosolic small ribosomal subunit (sensu Eukaryota) ? protein biosynthesis ?  0  BLAST| PFC1020c ? 40S ribosomal protein S3A, putative  structural constituent of ribosome ? intracellular (IEA) ? ribosome (IEA) ? cytosolic small ribosomal subunit (sensu Eukaryota) ? protein biosynthesis ? \*\* also with: YLR441C, clust.pair #14 YML063W ? structural constituent of ribosome ? cytosolic small ribosomal subunit (sensu Eukaryota) ? protein biosynthesis ?  0  BLAST | | | | | | | | | | | | | | | | | | | | | | | | | | | | | | | | | | | | | | | | | | | | | | | | | | | | | | | | | | | | | | | | | | | | | | | | | | | | | | | | | | | | | | | | | | | | | | | | | | | | | | | | | | | | | | | | | | | | | | | | | | | | | | | | | | | | | | | | | | | | | | | | | | | | | | | | | | | | | | | | | | | | | | | | | | | | | |

## Cluster Pair #15: 53 gene pairs.

|  |  |  |  |  |  |  |  |  |  |  |  |  |  |  |  |  |  |  |  |  |  |  |  |  |  |  |  |  |  |  |  |  |  |  |  |  |  |  |  |  |  |  |  |  |  |  |  |  |  |  |  |  |  |  |  |  |  |  |  |  |  |  |  |  |  |  |  |  |  |  |  |  |  |  |  |  |  |  |  |  |  |  |  |  |  |  |  |  |  |  |  |  |  |  |  |  |  |  |  |  |  |  |  |  |  |  |  |  |  |  |  |  |  |  |  |  |  |  |  |  |  |  |  |  |  |  |  |  |  |  |  |  |  |  |  |  |  |  |  |  |  |  |  |  |  |  |  |  |  |  |  |  |  |  |  |  |  |  |  |  |  |
| --- | --- | --- | --- | --- | --- | --- | --- | --- | --- | --- | --- | --- | --- | --- | --- | --- | --- | --- | --- | --- | --- | --- | --- | --- | --- | --- | --- | --- | --- | --- | --- | --- | --- | --- | --- | --- | --- | --- | --- | --- | --- | --- | --- | --- | --- | --- | --- | --- | --- | --- | --- | --- | --- | --- | --- | --- | --- | --- | --- | --- | --- | --- | --- | --- | --- | --- | --- | --- | --- | --- | --- | --- | --- | --- | --- | --- | --- | --- | --- | --- | --- | --- | --- | --- | --- | --- | --- | --- | --- | --- | --- | --- | --- | --- | --- | --- | --- | --- | --- | --- | --- | --- | --- | --- | --- | --- | --- | --- | --- | --- | --- | --- | --- | --- | --- | --- | --- | --- | --- | --- | --- | --- | --- | --- | --- | --- | --- | --- | --- | --- | --- | --- | --- | --- | --- | --- | --- | --- | --- | --- | --- | --- | --- | --- | --- | --- | --- | --- | --- | --- | --- | --- | --- | --- | --- | --- | --- | --- | --- | --- | --- |
| P.falciparum S.cerevisiae Blast evalue|  |  |  |  |  |  |  |  |  |  |  |  |  |  |  |  |  |  |  |  |  |  |  |  |  |  |  |  |  |  |  |  |  |  |  |  |  |  |  |  |  |  |  |  |  |  |  |  |  |  |  |  |  |  |  |  |  |  |  |  |  |  |  |  |  |  |  |  |  |  |  |  |  |  |  |  |  |  |  |  |  |  |  |  |  |  |  |  |  |  |  |  |  |  |  |  |  |  |  |  |  |  |  |  |  |  |  |  |  |  |  |  |  |  |  |  |  |  |  |  |  |  |  |  |  |  |  |  |  |  |  |  |  |  |  |  |  |  |  |  |  |  |  |  |  |  |  |  |  |  |  |  |  |  |  |  |  |  |  | | --- | --- | --- | --- | --- | --- | --- | --- | --- | --- | --- | --- | --- | --- | --- | --- | --- | --- | --- | --- | --- | --- | --- | --- | --- | --- | --- | --- | --- | --- | --- | --- | --- | --- | --- | --- | --- | --- | --- | --- | --- | --- | --- | --- | --- | --- | --- | --- | --- | --- | --- | --- | --- | --- | --- | --- | --- | --- | --- | --- | --- | --- | --- | --- | --- | --- | --- | --- | --- | --- | --- | --- | --- | --- | --- | --- | --- | --- | --- | --- | --- | --- | --- | --- | --- | --- | --- | --- | --- | --- | --- | --- | --- | --- | --- | --- | --- | --- | --- | --- | --- | --- | --- | --- | --- | --- | --- | --- | --- | --- | --- | --- | --- | --- | --- | --- | --- | --- | --- | --- | --- | --- | --- | --- | --- | --- | --- | --- | --- | --- | --- | --- | --- | --- | --- | --- | --- | --- | --- | --- | --- | --- | --- | --- | --- | --- | --- | --- | --- | --- | --- | --- | --- | --- | --- | --- | --- | --- | --- | | PFL2010c ? DEAD%2FDEAH box helicase, putative  nucleic acid binding (IEA) ? ATP-dependent RNA helicase activity ? helicase activity (IEA) ? ATP binding (IEA) ? ATP-dependent helicase activity (IEA) ? RNA metabolism ?  YLR276C ? ribosomal large subunit assembly and maintenance ? ATP-dependent RNA helicase activity ? nucleolus ? 35S primary transcript processing ? ribosome biogenesis and assembly ?  3e-25  BLAST| PFL1470c ? hypothetical protein  \*\* also with: YLR222C, clust.pair #15 YCR057C ? cytokinesis ? small nucleolar ribonucleoprotein complex ? cytoplasm ? 35S primary transcript processing ? establishment of cell polarity (sensu Fungi) ? processing of 20S pre-rRNA ? snoRNA binding ? 90S preribosome ? ribosome biogenesis and assembly ?  0.012  BLAST| PFL1470c ? hypothetical protein  \*\* also with: YCR057C, clust.pair #15 YLR222C ? small nucleolar ribonucleoprotein complex ? processing of 20S pre-rRNA ? snoRNA binding ?  0.012  BLAST| PF14\_0550 ? hypothetical protein   YDR060W ? ribosomal large subunit assembly and maintenance ? Noc1p-Noc2p complex ? ribosome biogenesis and assembly ?  0.011  BLAST| PFL1230w ? hypothetical protein   YLR186W ? nucleus ? nucleolus ? small nucleolar ribonucleoprotein complex ? cytoplasm ? nuclear microtubule ? 35S primary transcript processing ? ribosome biogenesis and assembly ? ribosomal small subunit biogenesis ?  3e-12  BLAST| MAL13P1.14 ? ATP-dependent DEAD box helicase, putative  nucleic acid binding (IEA) ? ATP-dependent RNA helicase activity ? helicase activity (IEA) ? ATP binding (IEA) ? ATP-dependent helicase activity (IEA) ?  YMR128W ? RNA helicase activity ? nucleolus ? small nucleolar ribonucleoprotein complex ? mitochondrion ? processing of 20S pre-rRNA ? ribosome biogenesis and assembly ?  9.94922e-44  BLAST| PF07\_0122 ? hypothetical protein, conserved   YOL077C ? ribosomal large subunit assembly and maintenance ? nucleolus ? 5S rRNA binding ? rRNA primary transcript binding ? ribosome biogenesis and assembly ?  1e-35  BLAST| PF11\_0105 ? hypothetical protein   YBR247C ? nucleus ? nucleolus ? rRNA processing ? 35S primary transcript processing ? snoRNA binding ? nucleolar preribosome, small subunit precursor ? ribosome biogenesis and assembly ?  1e-37  BLAST| PFD0360w ? hypothetical protein  DNA binding (IEA) ? transcription factor activity (IEA) ? RNA elongation (IEA) ? regulation of transcription, DNA-dependent (IEA) ?  YJR063W ? DNA-directed RNA polymerase activity ? DNA-directed RNA polymerase I complex ? transcription from RNA polymerase I promoter ? ribosome biogenesis and assembly ?  1e-07  BLAST| MAL13P1.213 ? transcription activator, putative  DNA binding (IEA) ? transcription factor activity ? DNA-directed RNA polymerase activity (IEA) ? transcription ?  YHR143W-A ? DNA-directed RNA polymerase activity ? DNA-directed RNA polymerase II, core complex ? DNA-directed RNA polymerase III complex ? DNA-directed RNA polymerase I complex ? transcription from RNA polymerase I promoter ? transcription from RNA polymerase II promoter ? transcription from RNA polymerase III promoter ?  5e-07  BLAST| PF10\_0194 ? hypothetical protein  nucleic acid binding (IEA) ?  YOL041C ? RNA binding ? nucleolus ? rRNA metabolism ?  1e-13  BLAST| PF14\_0185 ? ATP-dependent RNA helicase, putative  nucleic acid binding (IEA) ? ATP-dependent RNA helicase activity ? helicase activity (IEA) ? ATP binding (IEA) ? ATP-dependent helicase activity (IEA) ? \*\* also with: YFL002C, clust.pair #15 \*\* also with: YHR065C, clust.pair #15 \*\* also with: YGL078C, clust.pair #15 \*\* also with: YJL033W, clust.pair #15 YMR290C ? RNA binding ? ATP-dependent RNA helicase activity ? nuclear membrane ? nucleolus ? rRNA processing ? RNA-dependent ATPase activity ? ribosome biogenesis and assembly ?  5e-10  BLAST| PF14\_0185 ? ATP-dependent RNA helicase, putative  nucleic acid binding (IEA) ? ATP-dependent RNA helicase activity ? helicase activity (IEA) ? ATP binding (IEA) ? ATP-dependent helicase activity (IEA) ? \*\* also with: YMR290C, clust.pair #15 \*\* also with: YHR065C, clust.pair #15 \*\* also with: YGL078C, clust.pair #15 \*\* also with: YJL033W, clust.pair #15 YFL002C ? ribosomal large subunit assembly and maintenance ? ATP-dependent RNA helicase activity ? nucleolus ? 35S primary transcript processing ? ribosome biogenesis and assembly ?  6e-10  BLAST| PF14\_0185 ? ATP-dependent RNA helicase, putative  nucleic acid binding (IEA) ? ATP-dependent RNA helicase activity ? helicase activity (IEA) ? ATP binding (IEA) ? ATP-dependent helicase activity (IEA) ? \*\* also with: YMR290C, clust.pair #15 \*\* also with: YFL002C, clust.pair #15 \*\* also with: YGL078C, clust.pair #15 \*\* also with: YJL033W, clust.pair #15 YHR065C ? ATP-dependent RNA helicase activity ? nucleolus ? 35S primary transcript processing ? ribosome biogenesis and assembly ?  1e-09  BLAST| PF14\_0185 ? ATP-dependent RNA helicase, putative  nucleic acid binding (IEA) ? ATP-dependent RNA helicase activity ? helicase activity (IEA) ? ATP binding (IEA) ? ATP-dependent helicase activity (IEA) ? \*\* also with: YMR290C, clust.pair #15 \*\* also with: YFL002C, clust.pair #15 \*\* also with: YHR065C, clust.pair #15 \*\* also with: YJL033W, clust.pair #15 YGL078C ? ribosomal large subunit assembly and maintenance ? ATP-dependent RNA helicase activity ? nucleolus ? 35S primary transcript processing ?  2e-08  BLAST| PF14\_0185 ? ATP-dependent RNA helicase, putative  nucleic acid binding (IEA) ? ATP-dependent RNA helicase activity ? helicase activity (IEA) ? ATP binding (IEA) ? ATP-dependent helicase activity (IEA) ? \*\* also with: YMR290C, clust.pair #15 \*\* also with: YFL002C, clust.pair #15 \*\* also with: YHR065C, clust.pair #15 \*\* also with: YGL078C, clust.pair #15 YJL033W ? ATP-dependent RNA helicase activity ? nucleolus ? 35S primary transcript processing ? ribosome biogenesis and assembly ?  2e-08  BLAST| PFI0860c ? ATP-dependant RNA helicase, putative  nucleic acid binding (IEA) ? helicase activity (IEA) ? ATP binding (IEA) ? ATP-dependent helicase activity (IEA) ?  YGL120C ? U2-type spliceosome disassembly ? ATP-dependent RNA helicase activity ? spliceosome complex ? mitochondrion ? rRNA processing ? 35S primary transcript processing ? processing of 27S pre-rRNA ? processing of 20S pre-rRNA ? RNA splicing factor activity, transesterification mechanism ? ribosome biogenesis and assembly ? ribosomal large subunit biogenesis ?  0  BLAST| PF10\_0341 ? hypothetical protein, conserved  metalloendopeptidase activity (IEA) ? pseudouridylate synthase activity (IEA) ? proteolysis and peptidolysis (IEA) ? tRNA processing (IEA) ? pathogenesis (IEA) ? metal ion binding (IEA) ?  YOR243C ? pseudouridine synthesis ? nucleus ? tRNA modification ? pseudouridine synthase activity ? snRNA modification ? ribosome biogenesis and assembly ?  1e-18  BLAST| PF13\_0261 ? ATP binding protein, putative  ATP binding ?  YLR243W ? signal sequence binding ?  2e-24  BLAST| PF13\_0286 ? methyltransferase, putative  rRNA processing ? RNA methyltransferase activity ?  YCL054W ? nucleus ? nucleolus ? rRNA (uridine-2'-O-)-methyltransferase activity ? rRNA (guanine) methyltransferase activity ? processing of 27S pre-rRNA ? rRNA methylation ?  0  BLAST| PF14\_0292 ? hypothetical protein, conserved  GTP binding (IEA) ?  YGL099W ? ribosome-nucleus export ? conjugation with cellular fusion ? GTPase activity ? cytoplasm ? sporulation (sensu Fungi) ? ribosome biogenesis and assembly ?  2e-30  BLAST| PF13\_0177 ? ATP-dependent RNA helicase, putative  nucleic acid binding (IEA) ? helicase activity (IEA) ? ATP binding (IEA) ? ATP-dependent helicase activity (IEA) ? \*\* also with: YHR169W, clust.pair #16 YHR065C ? ATP-dependent RNA helicase activity ? nucleolus ? 35S primary transcript processing ? ribosome biogenesis and assembly ?  0  BLAST| PF14\_0456 ? hypothetical protein, conserved   YLR129W ? small nucleolar ribonucleoprotein complex ? processing of 20S pre-rRNA ? snoRNA binding ? ribosome biogenesis and assembly ?  5.04467e-44  BLAST| PF13\_0035 ? hypothetical protein   YDR449C ? small nucleolar ribonucleoprotein complex ? processing of 20S pre-rRNA ? snoRNA binding ? ribosome biogenesis and assembly ?  4e-07  BLAST| MAL13P1.341 ? hypothetical protein, conserved   YKL009W ? telomere maintenance ? nucleus ? nucleolus ? rRNA processing ? mRNA catabolism ? ribosome biogenesis and assembly ? ribosomal large subunit biogenesis ?  4e-06  BLAST| PF11\_0275 ? hypothetical protein   YLR409C ? nucleus ? nucleolus ? small nucleolar ribonucleoprotein complex ? 35S primary transcript processing ? snoRNA binding ? ribosome biogenesis and assembly ?  0.083  BLAST| MAL7P1.24 ? hypothetical protein, conserved  intracellular (IEA) ?  YER126C ? nucleus ? ribosome biogenesis and assembly ? ribosomal large subunit biogenesis ?  0  BLAST| PF10\_0087 ? diphthine synthase  diphthine synthase activity ? metabolism (IEA) ? methyltransferase activity (IEA) ? peptidyl-diphthamide biosynthesis from peptidyl-histidine (IEA) ?  YLR172C ? diphthine synthase activity ? cytoplasm ? peptidyl-diphthamide biosynthesis from peptidyl-histidine ?  0  BLAST| PFB0860c ? RNA helicase, putative  nucleic acid binding (IEA) ? ATP-dependent RNA helicase activity ? helicase activity (IEA) ? ATP binding (IEA) ? ATP-dependent helicase activity (IEA) ?  YHR065C ? ATP-dependent RNA helicase activity ? nucleolus ? 35S primary transcript processing ? ribosome biogenesis and assembly ?  0  BLAST| PFE0515w ? hypothetical protein   YOL022C ? cytoplasm ?  0.005  BLAST| PFE1310c ? hypothetical protein  \*\* also with: YCR057C, clust.pair #15 \*\* also with: YPR169W, clust.pair #16 YLR129W ? small nucleolar ribonucleoprotein complex ? processing of 20S pre-rRNA ? snoRNA binding ? ribosome biogenesis and assembly ?  0.0006  BLAST| PFE1310c ? hypothetical protein  \*\* also with: YLR129W, clust.pair #15 \*\* also with: YPR169W, clust.pair #16 YCR057C ? cytokinesis ? small nucleolar ribonucleoprotein complex ? cytoplasm ? 35S primary transcript processing ? establishment of cell polarity (sensu Fungi) ? processing of 20S pre-rRNA ? snoRNA binding ? 90S preribosome ? ribosome biogenesis and assembly ?  0.001  BLAST| PF11\_0471 ? hypothetical protein   YCR072C ? ribosomal large subunit assembly and maintenance ? nucleolus ? ribosome ? ribosome biogenesis and assembly ?  2.8026e-45  BLAST| PF13\_0309 ? hypothetical protein   YLR409C ? nucleus ? nucleolus ? small nucleolar ribonucleoprotein complex ? 35S primary transcript processing ? snoRNA binding ? ribosome biogenesis and assembly ?  1e-17  BLAST| PF07\_0121 ? hypothetical protein, conserved   YHR170W ? ribosomal large subunit assembly and maintenance ? ribosomal large subunit-nucleus export ? RNA binding ? protein binding ? cytosol ? cytosolic large ribosomal subunit (sensu Eukaryota) ? ribosome biogenesis and assembly ?  2.94273e-44  BLAST| PF13\_0184 ? hypothetical protein  metalloendopeptidase activity (IEA) ? cytoplasm (IEA) ? proteolysis and peptidolysis (IEA) ? phosphate transport (IEA) ? pathogenesis (IEA) ? membrane ? metal ion binding (IEA) ?  YDR324C ? small nucleolar ribonucleoprotein complex ? processing of 20S pre-rRNA ? snoRNA binding ? ribosome biogenesis and assembly ?  2e-07  BLAST| PFL1345c ? hypothetical protein, conserved  catalytic activity (IEA) ? iron ion binding (IEA) ? membrane ?  YPL086C ? histone acetyltransferase activity ? nucleus ? cytoplasm ? regulation of transcription from RNA polymerase II promoter ? tRNA modification ? transcription elongation factor complex ?  0  BLAST| PF11\_0358 ? DNA-directed RNA polymerase, beta subunit, putative  DNA binding ? DNA-directed RNA polymerase activity ? DNA-directed RNA polymerase I complex ? transcription (IEA) ? transcription from RNA polymerase I promoter ?  YPR010C ? DNA-directed RNA polymerase activity ? DNA-directed RNA polymerase I complex ? transcription from RNA polymerase I promoter ? ribosome biogenesis and assembly ?  0  BLAST| PF13\_0178 ? translation initiation factor 6, putative  translation initiation factor activity (IEA) ? translational initiation (IEA) ?  YPR016C ? nucleus ? cytoplasm ? processing of 27S pre-rRNA ? ribosomal large subunit biogenesis ? ribosomal large subunit binding ?  0  BLAST| PFE1435c ? hypothetical protein   YER006W ? GTPase activity ? nucleus ? nucleolus ? rRNA processing ? ribosome biogenesis and assembly ?  4e-09  BLAST| PF14\_0068 ? fibrillarin, putative  RNA binding (IEA) ? nucleus (IEA) ? small nucleolar ribonucleoprotein complex ? mitochondrion ? rRNA processing ?  YDL014W ? ribosomal large subunit assembly and maintenance ? rRNA modification ? RNA methylation ? nucleolus ? small nucleolar ribonucleoprotein complex ? ribosome ? 35S primary transcript processing ? methyltransferase activity ? processing of 20S pre-rRNA ? snoRNA 3'-end processing ? ribosome biogenesis and assembly ?  0  BLAST| PF14\_0635 ? hypothetical protein, conserved  RNA binding (IEA) ?  YPL211W ? ribosomal large subunit assembly and maintenance ? nucleolus ? cytosolic large ribosomal subunit (sensu Eukaryota) ? rRNA processing ? ribosome biogenesis and assembly ? ribosomal large subunit biogenesis ?  2e-35  BLAST| PF08\_0130 ? wd repeat protein, putative   YCR057C ? cytokinesis ? small nucleolar ribonucleoprotein complex ? cytoplasm ? 35S primary transcript processing ? establishment of cell polarity (sensu Fungi) ? processing of 20S pre-rRNA ? snoRNA binding ? 90S preribosome ? ribosome biogenesis and assembly ?  0  BLAST| PF13\_0341 ? DNA-directed RNA polymerase 2, putative  DNA binding ? DNA-directed RNA polymerase activity ? DNA-directed RNA polymerase II, core complex ? transcription (IEA) ? transcription from RNA polymerase II promoter ?  YBR154C ? DNA-directed RNA polymerase activity ? DNA-directed RNA polymerase II, core complex ? DNA-directed RNA polymerase III complex ? DNA-directed RNA polymerase I complex ? transcription from RNA polymerase I promoter ? transcription from RNA polymerase II promoter ? transcription from RNA polymerase III promoter ?  3e-37  BLAST| PF10\_0200 ? hypothetical protein, conserved   YNL132W ? nucleolus ? ribosome biogenesis and assembly ?  2e-39  BLAST| PF10\_0219 ? hypothetical protein   YPL263C ? cytoplasm ?  3e-29  BLAST| PF11\_0191 ? hypothetical protein   YLR197W ? rRNA modification ? nucleus ? nucleolus ? small nucleolar ribonucleoprotein complex ? 35S primary transcript processing ? processing of 20S pre-rRNA ? box C/D snoRNP complex ? ribosome biogenesis and assembly ?  0  BLAST| MAL7P1.113 ? DEAD box helicase, putative  nucleic acid binding (IEA) ? RNA binding ? ATP-dependent RNA helicase activity ? helicase activity (IEA) ? ATP binding ? ATP-dependent helicase activity (IEA) ? RNA metabolism ? \*\* also with: YKR024C, clust.pair #16 YMR290C ? RNA binding ? ATP-dependent RNA helicase activity ? nuclear membrane ? nucleolus ? rRNA processing ? RNA-dependent ATPase activity ? ribosome biogenesis and assembly ?  1e-24  BLAST| PF13\_0109 ? N2,N2-dimethylguanosine tRNA methyltransferase, putative  RNA binding (IEA) ? tRNA (guanine-N2-)-methyltransferase activity ? tRNA modification ? tRNA processing (IEA) ? apicoplast ?  YDR120C ? tRNA (guanine-N2-)-methyltransferase activity ? nuclear membrane ? nuclear inner membrane ? mitochondrion ? tRNA methylation ? ribosome biogenesis and assembly ?  3e-13  BLAST| PF14\_0194 ? spliceosome-associated protein, putative  nucleic acid binding (IEA) ? spliceosome complex ? RNA splicing ? RNA splicing factor activity, transesterification mechanism ?  YER165W ? nucleus ? cytoplasm ? ribosome ? regulation of translational initiation ? poly(A) binding ?  2e-19  BLAST| PF14\_0174 ? hypothetical protein, conserved  RNA binding (IEA) ? pseudouridylate synthase activity (IEA) ? RNA processing (IEA) ?  YLR175W ? pseudouridylate synthase activity ? nucleolus ? 35S primary transcript processing ? rRNA pseudouridine synthesis ? box H/ACA snoRNP complex ? ribosome biogenesis and assembly ?  0  BLAST| PF08\_0065 ? hypothetical protein, conserved   YMR131C ? nucleolus ? ribosome biogenesis and assembly ?  5e-33  BLAST| PF13\_0219 ? hypothetical protein   YOR206W ? ribosome-nucleus export ? nucleus ? mitochondrion ? Noc1p-Noc2p complex ? Noc2p-Noc3p complex ? ribosome biogenesis and assembly ? ribosome assembly ?  0.074  BLAST | | | | | | | | | | | | | | | | | | | | | | | | | | | | | | | | | | | | | | | | | | | | | | | | | | | | | | | | | | | | | | | | | | | | | | | | | | | | | | | | | | | | | | | | | | | | | | | | | | | | | | | | | | | | | | | | | | | | | | | | | | | | | | | | | | | | | | | | | | | | | | | | | | | | | | | | | | | | | | | | | |

## Cluster Pair #16: 21 gene pairs.

|  |  |  |  |  |  |  |  |  |  |  |  |  |  |  |  |  |  |  |  |  |  |  |  |  |  |  |  |  |  |  |  |  |  |  |  |  |  |  |  |  |  |  |  |  |  |  |  |  |  |  |  |  |  |  |  |  |  |  |  |  |  |  |  |  |  |
| --- | --- | --- | --- | --- | --- | --- | --- | --- | --- | --- | --- | --- | --- | --- | --- | --- | --- | --- | --- | --- | --- | --- | --- | --- | --- | --- | --- | --- | --- | --- | --- | --- | --- | --- | --- | --- | --- | --- | --- | --- | --- | --- | --- | --- | --- | --- | --- | --- | --- | --- | --- | --- | --- | --- | --- | --- | --- | --- | --- | --- | --- | --- | --- | --- | --- |
| P.falciparum S.cerevisiae Blast evalue|  |  |  |  |  |  |  |  |  |  |  |  |  |  |  |  |  |  |  |  |  |  |  |  |  |  |  |  |  |  |  |  |  |  |  |  |  |  |  |  |  |  |  |  |  |  |  |  |  |  |  |  |  |  |  |  |  |  |  |  |  |  |  | | --- | --- | --- | --- | --- | --- | --- | --- | --- | --- | --- | --- | --- | --- | --- | --- | --- | --- | --- | --- | --- | --- | --- | --- | --- | --- | --- | --- | --- | --- | --- | --- | --- | --- | --- | --- | --- | --- | --- | --- | --- | --- | --- | --- | --- | --- | --- | --- | --- | --- | --- | --- | --- | --- | --- | --- | --- | --- | --- | --- | --- | --- | --- | | PFD0515w ? exosome complex exonuclease rrp4, putative  3'-5'-exoribonuclease activity ? exosome (RNase complex) ? nucleic acid binding (IEA) ? RNA binding (IEA) ? rRNA processing ?  YHR069C ? 3'-5'-exoribonuclease activity ? nuclear exosome (RNase complex) ? cytoplasmic exosome (RNase complex) ? RNA binding ? 35S primary transcript processing ? mRNA catabolism ?  4e-33  BLAST| PF14\_0661 ? hypothetical protein, conserved  nucleic acid binding (IEA) ?  YOR145C ? nucleus ? nucleolus ? rRNA processing ? 35S primary transcript processing ? protein complex assembly ? ribosome biogenesis and assembly ? unfolded protein binding ?  0  BLAST| PFL2295w ? hypothetical protein  rRNA processing (IEA) ? ribonucleoprotein complex (IEA) ?  YKL099C ? small nucleolar ribonucleoprotein complex ? processing of 20S pre-rRNA ? snoRNA binding ? ribosome biogenesis and assembly ?  0.095  BLAST| PF14\_0150 ? RNA polymerase small subunit, putative  DNA binding (IEA) ? DNA-directed RNA polymerase activity ? transcription ? protein dimerization activity (IEA) ?  YNL113W ? DNA-directed RNA polymerase activity ? DNA-directed RNA polymerase III complex ? DNA-directed RNA polymerase I complex ? transcription from RNA polymerase I promoter ? transcription from RNA polymerase III promoter ? ribosome biogenesis and assembly ?  1e-18  BLAST| PF14\_0584 ? ribosomal protein S4, putative  RNA binding (IEA) ? structural constituent of ribosome ? mitochondrial small ribosomal subunit ? protein biosynthesis ?  YHR148W ? rRNA modification ? small nucleolar ribonucleoprotein complex ? 35S primary transcript processing ? processing of 20S pre-rRNA ? snoRNA binding ? ribosome biogenesis and assembly ?  1e-35  BLAST| PF13\_0177 ? ATP-dependent RNA helicase, putative  nucleic acid binding (IEA) ? helicase activity (IEA) ? ATP binding (IEA) ? ATP-dependent helicase activity (IEA) ? \*\* also with: YHR065C, clust.pair #15 YHR169W ? ATP-dependent RNA helicase activity ? nucleolus ? 35S primary transcript processing ? ribosome biogenesis and assembly ?  0  BLAST| PFB0175c ? hypothetical protein   YAL025C ? nucleolus ? processing of 27S pre-rRNA ? ribosome biogenesis and assembly ? ribosomal large subunit biogenesis ?  1e-25  BLAST| PF10\_0277 ? hypothetical protein, conserved   YKL172W ? nuclear division ? nucleolus ? rRNA processing ? ribosome biogenesis and assembly ?  2e-07  BLAST| PF10\_0278 ? hypothetical protein, conserved   YKR081C ? ribosomal large subunit assembly and maintenance ? nucleolus ? 5S rRNA binding ? 7S RNA binding ? rRNA binding ? processing of 27S pre-rRNA ? ribosome biogenesis and assembly ?  2e-05  BLAST| PFB0290c ? transcription factor, putative  DNA binding (IEA) ? transcription factor activity ? DNA-directed RNA polymerase III complex ? RNA elongation (IEA) ? regulation of transcription, DNA-dependent (IEA) ? transcription from RNA polymerase III promoter ?  YDR045C ? DNA-directed RNA polymerase activity ? DNA-directed RNA polymerase III complex ? transcription from RNA polymerase III promoter ?  7e-15  BLAST| PFB0370c ? RNA-binding protein, putative   YCL059C ? nucleolus ? small nucleolar ribonucleoprotein complex ? rRNA processing ? 35S primary transcript processing ? ribosome biogenesis and assembly ?  0  BLAST| PF11\_0274 ? hypothetical protein   YFR001W ? mRNA binding ? nucleus ? intracellular mRNA localization ? ribosomal large subunit biogenesis ?  0.002  BLAST| PF07\_0092 ? hypothetical protein, conserved   YER082C ? small nucleolar ribonucleoprotein complex ? rRNA processing ? 35S primary transcript processing ? snoRNA binding ? ribosome biogenesis and assembly ?  0  BLAST| PF07\_0083 ? hypothetical protein, conserved   YNR054C ? nucleolus ? small nucleolar ribonucleoprotein complex ? cytoplasm ? 35S primary transcript processing ? transcription regulator activity ? ribosome biogenesis and assembly ?  6e-15  BLAST| MAL8P1.67 ? hypothetical protein, conserved   YDR339C ? nucleolus ? 35S primary transcript processing ? mitochondrion organization and biogenesis ?  0  BLAST| PFI0920c ? hypothetical protein, conserved  tRNA processing (IEA) ? oxidoreductase activity (IEA) ? apicoplast ? FAD binding (IEA) ?  YML080W ? nucleus ? tRNA modification ? tRNA dihydrouridine synthase activity ?  9e-10  BLAST| PFE1310c ? hypothetical protein  \*\* also with: YLR129W, clust.pair #15 \*\* also with: YCR057C, clust.pair #15 YPR169W ? nucleus ? nucleolus ? protein monoubiquitination ?  0.0001  BLAST| PF13\_0310 ? hypothetical protein   YDL153C ? nucleus ? small nucleolar ribonucleoprotein complex ? establishment and/or maintenance of chromatin architecture ? processing of 20S pre-rRNA ? snoRNA binding ? ribosome biogenesis and assembly ?  2e-06  BLAST| PFI1070c ? hypothetical protein   YHR088W ? ribosomal large subunit assembly and maintenance ? nucleolus ? processing of 27S pre-rRNA ? rRNA primary transcript binding ? ribosome biogenesis and assembly ?  5e-18  BLAST| MAL7P1.113 ? DEAD box helicase, putative  nucleic acid binding (IEA) ? RNA binding ? ATP-dependent RNA helicase activity ? helicase activity (IEA) ? ATP binding ? ATP-dependent helicase activity (IEA) ? RNA metabolism ? \*\* also with: YMR290C, clust.pair #15 YKR024C ? ribosomal large subunit assembly and maintenance ? ATP-dependent RNA helicase activity ? nucleolus ? 35S primary transcript processing ? ribosome biogenesis and assembly ?  5e-26  BLAST| PF14\_0221 ? hypothetical protein, conserved  GTP binding (IEA) ?  YNR053C ? ribosomal large subunit-nucleus export ? GTPase activity ? nucleus ? nucleoplasm ? nucleolus ? ribosome biogenesis and assembly ? ribosome assembly ?  0  BLAST | | | | | | | | | | | | | | | | | | | | | | | | | | | | | | | | | | | | | | | | | | | | | | | | | | | | | | | | | | | | | | | | | |

## Cluster Pair #17: 13 gene pairs.

|  |  |  |  |  |  |  |  |  |  |  |  |  |  |  |  |  |  |  |  |  |  |  |  |  |  |  |  |  |  |  |  |  |  |  |  |  |  |  |  |  |  |
| --- | --- | --- | --- | --- | --- | --- | --- | --- | --- | --- | --- | --- | --- | --- | --- | --- | --- | --- | --- | --- | --- | --- | --- | --- | --- | --- | --- | --- | --- | --- | --- | --- | --- | --- | --- | --- | --- | --- | --- | --- | --- |
| P.falciparum S.cerevisiae Blast evalue|  |  |  |  |  |  |  |  |  |  |  |  |  |  |  |  |  |  |  |  |  |  |  |  |  |  |  |  |  |  |  |  |  |  |  |  |  |  |  | | --- | --- | --- | --- | --- | --- | --- | --- | --- | --- | --- | --- | --- | --- | --- | --- | --- | --- | --- | --- | --- | --- | --- | --- | --- | --- | --- | --- | --- | --- | --- | --- | --- | --- | --- | --- | --- | --- | --- | | PF10\_0357 ? hypothetical protein   YGR159C ? ribosomal small subunit assembly and maintenance ? telomere maintenance ? single-stranded DNA binding ? RNA binding ? nucleus ? nucleolus ? mitochondrion ? rRNA processing ?  0.014  BLAST| PF10\_0028 ? hypothetical protein, conserved  nucleic acid binding (IEA) ? \*\* also with: YER165W, clust.pair #17 \*\* also with: YGR159C, clust.pair #17 YPL043W ? RNA binding ? nucleolus ? rRNA processing ? ribosome biogenesis and assembly ?  2e-09  BLAST| PF10\_0028 ? hypothetical protein, conserved  nucleic acid binding (IEA) ? \*\* also with: YPL043W, clust.pair #17 \*\* also with: YGR159C, clust.pair #17 YER165W ? nucleus ? cytoplasm ? ribosome ? regulation of translational initiation ? poly(A) binding ?  3e-09  BLAST| PF10\_0028 ? hypothetical protein, conserved  nucleic acid binding (IEA) ? \*\* also with: YPL043W, clust.pair #17 \*\* also with: YER165W, clust.pair #17 YGR159C ? ribosomal small subunit assembly and maintenance ? telomere maintenance ? single-stranded DNA binding ? RNA binding ? nucleus ? nucleolus ? mitochondrion ? rRNA processing ?  4e-08  BLAST| PFD0455w ? ribosomal processing protein, putative  rRNA modification ? small nucleolar ribonucleoprotein complex ?  YLL011W ? rRNA modification ? nucleolus ? small nucleolar ribonucleoprotein complex ? 35S primary transcript processing ? processing of 20S pre-rRNA ? snoRNA binding ? small nuclear ribonucleoprotein complex ?  0  BLAST| PFD0450c ? pre-mrna splicing factor, putative  spliceosome complex ? RNA splicing ? RNA splicing factor activity, transesterification mechanism ? \*\* also with: YLR197W, clust.pair #17 YOR310C ? rRNA modification ? small nucleolar ribonucleoprotein complex ? 35S primary transcript processing ? processing of 20S pre-rRNA ? box C/D snoRNP complex ? ribosome biogenesis and assembly ?  9e-18  BLAST| PFD0450c ? pre-mrna splicing factor, putative  spliceosome complex ? RNA splicing ? RNA splicing factor activity, transesterification mechanism ? \*\* also with: YOR310C, clust.pair #17 YLR197W ? rRNA modification ? nucleus ? nucleolus ? small nucleolar ribonucleoprotein complex ? 35S primary transcript processing ? processing of 20S pre-rRNA ? box C/D snoRNP complex ? ribosome biogenesis and assembly ?  2e-16  BLAST| PF10\_0085 ? nucleolar protein NOP5, putative  RNA binding ? rRNA processing ?  YOR310C ? rRNA modification ? small nucleolar ribonucleoprotein complex ? 35S primary transcript processing ? processing of 20S pre-rRNA ? box C/D snoRNP complex ? ribosome biogenesis and assembly ?  0  BLAST| PF11\_0086 ? hypothetical protein  RNA binding (IEA) ?  YGR162W ? translation initiation factor activity ? mitochondrion ? ribosome ? translational initiation ? eukaryotic translation initiation factor 4F complex ? ribosome biogenesis and assembly ?  3e-13  BLAST| PFL1170w ? polyadenylate-binding protein, putative  nucleic acid binding (IEA) ? RNA binding (IEA) ? mRNA binding ? RNA processing ? poly(A) binding ?  YER165W ? nucleus ? cytoplasm ? ribosome ? regulation of translational initiation ? poly(A) binding ?  0  BLAST| PFL0830w ? hypothetical protein  nucleic acid binding (IEA) ?  YPR112C ? nucleolus ? 35S primary transcript processing ? snoRNA binding ? rRNA primary transcript binding ?  1.4013e-45  BLAST| PFL0075w ? XPA binding protein 1, putative  RNA binding (IEA) ? ATP binding ? GTP binding (IEA) ? signal recognition particle (sensu Eukaryota) (IEA) ? SRP-dependent cotranslational protein-membrane targeting (IEA) ?  YJR072C ? cytoplasm ? ATPase activity ?  0  BLAST| PFL0675c ? hypothetical protein   YDR211W ? translation initiation factor activity ? guanyl-nucleotide exchange factor activity ? eukaryotic translation initiation factor 2B complex ? regulation of translational initiation ?  4e-20  BLAST | | | | | | | | | | | | | | | | | | | | | | | | | | | | | | | | | | | | | | | | | |

## Cluster Pair #18: 3 gene pairs.

|  |  |  |  |  |  |  |  |  |  |  |  |
| --- | --- | --- | --- | --- | --- | --- | --- | --- | --- | --- | --- |
| P.falciparum S.cerevisiae Blast evalue|  |  |  |  |  |  |  |  |  | | --- | --- | --- | --- | --- | --- | --- | --- | --- | | PF14\_0139 ? hypothetical protein  nucleic acid binding (IEA) ?  YKL059C ? RNA binding ? mRNA cleavage and polyadenylation specificity factor complex ? mRNA polyadenylylation ? mRNA cleavage ?  3e-05  BLAST| PFB0875c ? hypothetical protein  nucleus ?  YAL032C ? nuclear mRNA splicing, via spliceosome ? nucleus ? spliceosome complex ? transcriptional activator activity ? positive regulation of transcription from RNA polymerase II promoter ?  1e-16  BLAST| PFI1600w ? mRNA processing protein, putative  nucleic acid binding (IEA) ?  YGL044C ? RNA binding ? mRNA cleavage factor complex ? mRNA polyadenylylation ? mRNA cleavage ? protein heterodimerization activity ?  3e-10  BLAST | | | | | | | | | | | |

## Cluster Pair #19: 4 gene pairs.

|  |  |  |  |  |  |  |  |  |  |  |  |  |  |  |
| --- | --- | --- | --- | --- | --- | --- | --- | --- | --- | --- | --- | --- | --- | --- |
| P.falciparum S.cerevisiae Blast evalue|  |  |  |  |  |  |  |  |  |  |  |  | | --- | --- | --- | --- | --- | --- | --- | --- | --- | --- | --- | --- | | PF14\_0436 ? helicase, truncated, putative  nucleic acid binding (IEA) ? helicase activity ? ATP binding (IEA) ? ATP-dependent helicase activity (IEA) ?  YNL112W ? mRNA catabolism, nonsense-mediated decay ? RNA helicase activity ? nucleus ? cytoplasm ? mitochondrion ? 35S primary transcript processing ?  0  BLAST| PF08\_0026 ? hypothetical protein  zinc ion binding (IEA) ?  YNL308C ? nucleolus ? ribosome biogenesis and assembly ?  0.0008  BLAST| PFL1175w ? hypothetical protein   YJL069C ? nucleolus ? small nucleolar ribonucleoprotein complex ? 35S primary transcript processing ? ribosome biogenesis and assembly ?  7e-05  BLAST| PF10\_0266 ? hypothetical protein   YJR002W ? nucleus ? nucleolus ? small nucleolar ribonucleoprotein complex ? 35S primary transcript processing ? processing of 20S pre-rRNA ? ribosome biogenesis and assembly ?  2e-10  BLAST | | | | | | | | | | | | | | |

## Cluster Pair #20: 9 gene pairs.

|  |  |  |  |  |  |  |  |  |  |  |  |  |  |  |  |  |  |  |  |  |  |  |  |  |  |  |  |  |  |
| --- | --- | --- | --- | --- | --- | --- | --- | --- | --- | --- | --- | --- | --- | --- | --- | --- | --- | --- | --- | --- | --- | --- | --- | --- | --- | --- | --- | --- | --- |
| P.falciparum S.cerevisiae Blast evalue|  |  |  |  |  |  |  |  |  |  |  |  |  |  |  |  |  |  |  |  |  |  |  |  |  |  |  | | --- | --- | --- | --- | --- | --- | --- | --- | --- | --- | --- | --- | --- | --- | --- | --- | --- | --- | --- | --- | --- | --- | --- | --- | --- | --- | --- | | PF14\_0208 ? hypothetical protein, conserved  protein targeting to mitochondrion (IEA) ? mitochondrial intermembrane space protein transporter complex (IEA) ? mitochondrial inner membrane protein import (IEA) ?  YHR005C-A ? mitochondrion ? mitochondrial intermembrane space ? protein transporter activity ? mitochondrial intermembrane space protein transporter complex ? mitochondrial inner membrane protein import ? unfolded protein binding ?  0.028  BLAST| PF14\_0485 ? hypothetical protein   YDL192W ? telomere maintenance ? GTPase activity ? Golgi vesicle ? cytosol ? ER to Golgi transport ? intra-Golgi transport ? Golgi to plasma membrane transport ?  0.0002  BLAST| PF13\_0037 ? DEAD box helicase, putative  nucleic acid binding (IEA) ? ATP-dependent RNA helicase activity ? helicase activity (IEA) ? ATP binding (IEA) ? ATP-dependent helicase activity (IEA) ?  YDR194C ? RNA binding ? ATP-dependent RNA helicase activity ? mitochondrion ? mitochondrial matrix ? RNA-dependent ATPase activity ? RNA splicing ? RNA splicing factor activity, transesterification mechanism ?  5.04467e-44  BLAST| PFB0635w ? T-complex protein 1, putative  protein binding (IEA) ? ATP binding (IEA) ? protein folding ? ATPase activity, coupled ? cellular protein metabolism (IEA) ? unfolded protein binding (IEA) ?  YJL008C ? cytoplasm ? chaperonin-containing T-complex ? cytoskeleton ? protein folding ? cytoskeleton organization and biogenesis ? unfolded protein binding ?  0  BLAST| PFI0965w ? conserved protein, putative   YBL036C ? intracellular ? amino acid metabolism ? alanine racemase activity ? pyridoxal phosphate binding ?  3e-24  BLAST| PF11\_0258 ? co-chaperone GrpE, putative  adenyl-nucleotide exchange factor activity (IEA) ? protein binding (IEA) ? mitochondrion ? mitochondrial matrix ? protein folding (IEA) ? mitochondrial matrix protein import ? protein homodimerization activity (IEA) ? unfolded protein binding ? chaperone binding (IEA) ?  YOR232W ? presequence translocase-associated import motor ? mitochondrion ? mitochondrial matrix ? mitochondrial matrix protein import ? unfolded protein binding ?  1e-26  BLAST| PFL1425w ? t-complex protein 1, gamma subunit, putative  protein binding (IEA) ? ATP binding ? chaperonin-containing T-complex ? protein folding ? cellular protein metabolism (IEA) ? unfolded protein binding ?  YJL014W ? cytoplasm ? chaperonin-containing T-complex ? cytoskeleton ? protein folding ? cytoskeleton organization and biogenesis ? unfolded protein binding ?  0  BLAST| PF13\_0358 ? mitochondrial import inner membrane translocase, putative  mitochondrial intermembrane space ? protein targeting to mitochondrion ? protein translocase activity ? mitochondrial intermembrane space protein transporter complex (IEA) ? mitochondrial inner membrane protein import (IEA) ?  YHR005C-A ? mitochondrion ? mitochondrial intermembrane space ? protein transporter activity ? mitochondrial intermembrane space protein transporter complex ? mitochondrial inner membrane protein import ? unfolded protein binding ?  1e-05  BLAST| PFC0900w ? T-complex protein 1 epsilon subunit, putative  protein binding (IEA) ? ATP binding (IEA) ? chaperonin-containing T-complex ? protein folding ? cellular protein metabolism (IEA) ? unfolded protein binding ?  YJR064W ? cytoplasm ? chaperonin-containing T-complex ? cytoskeleton ? protein folding ? cytoskeleton organization and biogenesis ? unfolded protein binding ?  0  BLAST | | | | | | | | | | | | | | | | | | | | | | | | | | | | | |

## Cluster Pair #21: 2 gene pairs.

|  |  |  |  |  |  |  |  |  |
| --- | --- | --- | --- | --- | --- | --- | --- | --- |
| P.falciparum S.cerevisiae Blast evalue|  |  |  |  |  |  | | --- | --- | --- | --- | --- | --- | | PFL0500w ? 50S ribosomal protein L1, putative  structural constituent of ribosome ? intracellular (IEA) ? mitochondrion ? ribosome (IEA) ? protein biosynthesis ? cytosolic large ribosomal subunit (sensu Bacteria) ? membrane ? apicoplast ?  YDR116C ? structural constituent of ribosome ? mitochondrion ? mitochondrial large ribosomal subunit ? protein biosynthesis ? aerobic respiration ?  0.01  BLAST| PF11\_0407 ? adrenodoxin reductase, putative  mitochondrion ? electron transport ? NADPH-adrenodoxin reductase activity ?  YDR376W ? mitochondrion ? mitochondrial inner membrane ? iron ion homeostasis ? NADPH-adrenodoxin reductase activity ?  4e-37  BLAST | | | | | | | | |

## Cluster Pair #22: 2 gene pairs.

|  |  |  |  |  |  |  |  |  |
| --- | --- | --- | --- | --- | --- | --- | --- | --- |
| P.falciparum S.cerevisiae Blast evalue|  |  |  |  |  |  | | --- | --- | --- | --- | --- | --- | | PF10\_0317 ? hypothetical protein  membrane ?  YDR411C ? endoplasmic reticulum ? ER-associated protein catabolism ? unfolded protein response ?  8e-09  BLAST| MAL8P1.17 ? disulfide isomerase precursor, putative  protein disulfide isomerase activity ? endoplasmic reticulum (IEA) ? endoplasmic reticulum lumen ? electron transport (IEA) ? protein folding ? electron carrier activity (IEA) ? isomerase activity (IEA) ?  YCL043C ? protein disulfide isomerase activity ? endoplasmic reticulum lumen ? protein folding ? protein disulfide oxidoreductase activity ?  1.4013e-45  BLAST | | | | | | | | |

## Cluster Pair #23: 16 gene pairs.

|  |  |  |  |  |  |  |  |  |  |  |  |  |  |  |  |  |  |  |  |  |  |  |  |  |  |  |  |  |  |  |  |  |  |  |  |  |  |  |  |  |  |  |  |  |  |  |  |  |  |  |
| --- | --- | --- | --- | --- | --- | --- | --- | --- | --- | --- | --- | --- | --- | --- | --- | --- | --- | --- | --- | --- | --- | --- | --- | --- | --- | --- | --- | --- | --- | --- | --- | --- | --- | --- | --- | --- | --- | --- | --- | --- | --- | --- | --- | --- | --- | --- | --- | --- | --- | --- |
| P.falciparum S.cerevisiae Blast evalue|  |  |  |  |  |  |  |  |  |  |  |  |  |  |  |  |  |  |  |  |  |  |  |  |  |  |  |  |  |  |  |  |  |  |  |  |  |  |  |  |  |  |  |  |  |  |  |  | | --- | --- | --- | --- | --- | --- | --- | --- | --- | --- | --- | --- | --- | --- | --- | --- | --- | --- | --- | --- | --- | --- | --- | --- | --- | --- | --- | --- | --- | --- | --- | --- | --- | --- | --- | --- | --- | --- | --- | --- | --- | --- | --- | --- | --- | --- | --- | --- | | MAL8P1.19 ? hypothetical protein, conserved  nucleic acid binding (IEA) ? helicase activity (IEA) ? ATP binding (IEA) ? ATP-dependent helicase activity (IEA) ? apicoplast ? \*\* also with: YLL008W, clust.pair #23 \*\* also with: YMR290C, clust.pair #23 \*\* also with: YGL078C, clust.pair #23 \*\* also with: YDL031W, clust.pair #23 YGL171W ? ATP-dependent RNA helicase activity ? nucleolus ? 35S primary transcript processing ? ATPase activity ?  8e-34  BLAST| MAL8P1.19 ? hypothetical protein, conserved  nucleic acid binding (IEA) ? helicase activity (IEA) ? ATP binding (IEA) ? ATP-dependent helicase activity (IEA) ? apicoplast ? \*\* also with: YGL171W, clust.pair #23 \*\* also with: YMR290C, clust.pair #23 \*\* also with: YGL078C, clust.pair #23 \*\* also with: YDL031W, clust.pair #23 YLL008W ? ribosomal large subunit assembly and maintenance ? ATP-dependent RNA helicase activity ? nucleolus ? 35S primary transcript processing ? ribosome biogenesis and assembly ?  7e-32  BLAST| MAL8P1.19 ? hypothetical protein, conserved  nucleic acid binding (IEA) ? helicase activity (IEA) ? ATP binding (IEA) ? ATP-dependent helicase activity (IEA) ? apicoplast ? \*\* also with: YGL171W, clust.pair #23 \*\* also with: YLL008W, clust.pair #23 \*\* also with: YGL078C, clust.pair #23 \*\* also with: YDL031W, clust.pair #23 YMR290C ? RNA binding ? ATP-dependent RNA helicase activity ? nuclear membrane ? nucleolus ? rRNA processing ? RNA-dependent ATPase activity ? ribosome biogenesis and assembly ?  2e-31  BLAST| MAL8P1.19 ? hypothetical protein, conserved  nucleic acid binding (IEA) ? helicase activity (IEA) ? ATP binding (IEA) ? ATP-dependent helicase activity (IEA) ? apicoplast ? \*\* also with: YGL171W, clust.pair #23 \*\* also with: YLL008W, clust.pair #23 \*\* also with: YMR290C, clust.pair #23 \*\* also with: YDL031W, clust.pair #23 YGL078C ? ribosomal large subunit assembly and maintenance ? ATP-dependent RNA helicase activity ? nucleolus ? 35S primary transcript processing ?  1e-30  BLAST| MAL8P1.19 ? hypothetical protein, conserved  nucleic acid binding (IEA) ? helicase activity (IEA) ? ATP binding (IEA) ? ATP-dependent helicase activity (IEA) ? apicoplast ? \*\* also with: YGL171W, clust.pair #23 \*\* also with: YLL008W, clust.pair #23 \*\* also with: YMR290C, clust.pair #23 \*\* also with: YGL078C, clust.pair #23 YDL031W ? ribosomal large subunit assembly and maintenance ? ATP-dependent RNA helicase activity ? nucleolus ? 35S primary transcript processing ? ribosome biogenesis and assembly ?  5e-30  BLAST| PF11\_0090 ? hypothetical protein  intracellular (IEA) ? nucleolus (IEA) ? cell proliferation (IEA) ?  YGR103W ? nucleus ? nucleolus ? cell cycle ? cell proliferation ? processing of 20S pre-rRNA ? ribosome biogenesis and assembly ? ribosomal large subunit biogenesis ?  0  BLAST| MAL13P1.333 ? hypothetical protein   YHR197W ? ribosomal large subunit assembly and maintenance ? ribosome-nucleus export ? nucleus ? nucleoplasm ? 35S primary transcript processing ? ribosome biogenesis and assembly ?  0.098  BLAST| PFA0330w ? pfAARP2 protein  plasma membrane ?  YPL217C ? GTP binding ? nucleus ? nucleolus ? cytoplasm ? mitochondrion ? rRNA processing ? 35S primary transcript processing ? ribosome assembly ?  1.4013e-45  BLAST| PF08\_0092 ? hypothetical protein  S-adenosylmethionine-dependent methyltransferase activity (IEA) ?  YBR034C ? nucleus ? mRNA-nucleus export ? protein-arginine N-methyltransferase activity ? peptidyl-arginine modification ? ribosome biogenesis and assembly ?  1e-39  BLAST| PF14\_0429 ? RNA helicase, putative  nucleic acid binding (IEA) ? helicase activity ? ATP binding (IEA) ? ATP-dependent helicase activity (IEA) ? \*\* also with: YJL033W, clust.pair #23 \*\* also with: YMR290C, clust.pair #23 \*\* also with: YHR065C, clust.pair #23 YLL008W ? ribosomal large subunit assembly and maintenance ? ATP-dependent RNA helicase activity ? nucleolus ? 35S primary transcript processing ? ribosome biogenesis and assembly ?  4e-30  BLAST| PF14\_0429 ? RNA helicase, putative  nucleic acid binding (IEA) ? helicase activity ? ATP binding (IEA) ? ATP-dependent helicase activity (IEA) ? \*\* also with: YLL008W, clust.pair #23 \*\* also with: YMR290C, clust.pair #23 \*\* also with: YHR065C, clust.pair #23 YJL033W ? ATP-dependent RNA helicase activity ? nucleolus ? 35S primary transcript processing ? ribosome biogenesis and assembly ?  2e-29  BLAST| PF14\_0429 ? RNA helicase, putative  nucleic acid binding (IEA) ? helicase activity ? ATP binding (IEA) ? ATP-dependent helicase activity (IEA) ? \*\* also with: YLL008W, clust.pair #23 \*\* also with: YJL033W, clust.pair #23 \*\* also with: YHR065C, clust.pair #23 YMR290C ? RNA binding ? ATP-dependent RNA helicase activity ? nuclear membrane ? nucleolus ? rRNA processing ? RNA-dependent ATPase activity ? ribosome biogenesis and assembly ?  8e-29  BLAST| PF14\_0429 ? RNA helicase, putative  nucleic acid binding (IEA) ? helicase activity ? ATP binding (IEA) ? ATP-dependent helicase activity (IEA) ? \*\* also with: YLL008W, clust.pair #23 \*\* also with: YJL033W, clust.pair #23 \*\* also with: YMR290C, clust.pair #23 YHR065C ? ATP-dependent RNA helicase activity ? nucleolus ? 35S primary transcript processing ? ribosome biogenesis and assembly ?  1e-28  BLAST| PF14\_0100 ? cytidine triphosphate synthetase  catalytic activity (IEA) ? CTP synthase activity ? pyrimidine base metabolism ? pyrimidine nucleotide biosynthesis (IEA) ?  YBL039C ? CTP synthase activity ? cytosol ? CTP biosynthesis ? phospholipid biosynthesis ? pyrimidine base biosynthesis ?  0  BLAST| PFE0300c ? 60S ribosomal subunit protein L24, putative  structural constituent of ribosome ? intracellular (IEA) ? ribosome (IEA) ? protein biosynthesis ? large ribosomal subunit ?  YLR009W ? nucleolus ? ribosome biogenesis and assembly ? ribosomal large subunit biogenesis ?  7e-39  BLAST| PF07\_0067 ? hypothetical protein   YGR245C ? nucleus ? traversing start control point of mitotic cell cycle ? actin cytoskeleton organization and biogenesis ? ribosome biogenesis and assembly ? ribosome assembly ?  1e-09  BLAST | | | | | | | | | | | | | | | | | | | | | | | | | | | | | | | | | | | | | | | | | | | | | | | | | | |

## Cluster Pair #24: 55 gene pairs.

|  |  |  |  |  |  |  |  |  |  |  |  |  |  |  |  |  |  |  |  |  |  |  |  |  |  |  |  |  |  |  |  |  |  |  |  |  |  |  |  |  |  |  |  |  |  |  |  |  |  |  |  |  |  |  |  |  |  |  |  |  |  |  |  |  |  |  |  |  |  |  |  |  |  |  |  |  |  |  |  |  |  |  |  |  |  |  |  |  |  |  |  |  |  |  |  |  |  |  |  |  |  |  |  |  |  |  |  |  |  |  |  |  |  |  |  |  |  |  |  |  |  |  |  |  |  |  |  |  |  |  |  |  |  |  |  |  |  |  |  |  |  |  |  |  |  |  |  |  |  |  |  |  |  |  |  |  |  |  |  |  |  |  |  |  |  |  |  |
| --- | --- | --- | --- | --- | --- | --- | --- | --- | --- | --- | --- | --- | --- | --- | --- | --- | --- | --- | --- | --- | --- | --- | --- | --- | --- | --- | --- | --- | --- | --- | --- | --- | --- | --- | --- | --- | --- | --- | --- | --- | --- | --- | --- | --- | --- | --- | --- | --- | --- | --- | --- | --- | --- | --- | --- | --- | --- | --- | --- | --- | --- | --- | --- | --- | --- | --- | --- | --- | --- | --- | --- | --- | --- | --- | --- | --- | --- | --- | --- | --- | --- | --- | --- | --- | --- | --- | --- | --- | --- | --- | --- | --- | --- | --- | --- | --- | --- | --- | --- | --- | --- | --- | --- | --- | --- | --- | --- | --- | --- | --- | --- | --- | --- | --- | --- | --- | --- | --- | --- | --- | --- | --- | --- | --- | --- | --- | --- | --- | --- | --- | --- | --- | --- | --- | --- | --- | --- | --- | --- | --- | --- | --- | --- | --- | --- | --- | --- | --- | --- | --- | --- | --- | --- | --- | --- | --- | --- | --- | --- | --- | --- | --- | --- | --- | --- | --- | --- |
| P.falciparum S.cerevisiae Blast evalue|  |  |  |  |  |  |  |  |  |  |  |  |  |  |  |  |  |  |  |  |  |  |  |  |  |  |  |  |  |  |  |  |  |  |  |  |  |  |  |  |  |  |  |  |  |  |  |  |  |  |  |  |  |  |  |  |  |  |  |  |  |  |  |  |  |  |  |  |  |  |  |  |  |  |  |  |  |  |  |  |  |  |  |  |  |  |  |  |  |  |  |  |  |  |  |  |  |  |  |  |  |  |  |  |  |  |  |  |  |  |  |  |  |  |  |  |  |  |  |  |  |  |  |  |  |  |  |  |  |  |  |  |  |  |  |  |  |  |  |  |  |  |  |  |  |  |  |  |  |  |  |  |  |  |  |  |  |  |  |  |  |  |  |  |  | | --- | --- | --- | --- | --- | --- | --- | --- | --- | --- | --- | --- | --- | --- | --- | --- | --- | --- | --- | --- | --- | --- | --- | --- | --- | --- | --- | --- | --- | --- | --- | --- | --- | --- | --- | --- | --- | --- | --- | --- | --- | --- | --- | --- | --- | --- | --- | --- | --- | --- | --- | --- | --- | --- | --- | --- | --- | --- | --- | --- | --- | --- | --- | --- | --- | --- | --- | --- | --- | --- | --- | --- | --- | --- | --- | --- | --- | --- | --- | --- | --- | --- | --- | --- | --- | --- | --- | --- | --- | --- | --- | --- | --- | --- | --- | --- | --- | --- | --- | --- | --- | --- | --- | --- | --- | --- | --- | --- | --- | --- | --- | --- | --- | --- | --- | --- | --- | --- | --- | --- | --- | --- | --- | --- | --- | --- | --- | --- | --- | --- | --- | --- | --- | --- | --- | --- | --- | --- | --- | --- | --- | --- | --- | --- | --- | --- | --- | --- | --- | --- | --- | --- | --- | --- | --- | --- | --- | --- | --- | --- | --- | --- | --- | --- | --- | | PF11\_0043 ? 60S acidic ribosomal protein p1, putative  structural constituent of ribosome ? intracellular (IEA) ? ribosome (IEA) ? protein biosynthesis ? translational elongation (IEA) ? large ribosomal subunit ?  YDL081C ? telomere maintenance ? structural constituent of ribosome ? cytosolic large ribosomal subunit (sensu Eukaryota) ? protein biosynthesis ? translational elongation ?  2e-06  BLAST| PF08\_0019 ? guanine nucleotide-binding protein, putative  protein kinase C binding ? heterotrimeric G-protein complex ? G-protein coupled receptor protein signaling pathway ?  YMR116C ? telomere maintenance ? cytoplasm ? cytosolic small ribosomal subunit (sensu Eukaryota) ? negative regulation of protein biosynthesis ?  0  BLAST| PF14\_0083 ? ribosomal protein S8e, putative  structural constituent of ribosome ? intracellular (IEA) ? mitochondrion ? cytosolic small ribosomal subunit (sensu Eukaryota) ? protein biosynthesis ? \*\* also with: YER102W, clust.pair #24 YBL072C ? structural constituent of ribosome ? cytosolic small ribosomal subunit (sensu Eukaryota) ? protein biosynthesis ?  0  BLAST| PF14\_0083 ? ribosomal protein S8e, putative  structural constituent of ribosome ? intracellular (IEA) ? mitochondrion ? cytosolic small ribosomal subunit (sensu Eukaryota) ? protein biosynthesis ? \*\* also with: YBL072C, clust.pair #24 YER102W ? structural constituent of ribosome ? cytosolic small ribosomal subunit (sensu Eukaryota) ? protein biosynthesis ?  0  BLAST| PF14\_0448 ? ribosomal protein S2, putative  structural constituent of ribosome ? intracellular (IEA) ? ribosome (IEA) ? cytosolic small ribosomal subunit (sensu Eukaryota) ? protein biosynthesis ? small ribosomal subunit (IEA) ?  YGL123W ? structural constituent of ribosome ? small nucleolar ribonucleoprotein complex ? cytosolic small ribosomal subunit (sensu Eukaryota) ? protein biosynthesis ? regulation of translational fidelity ?  0  BLAST| PF13\_0316 ? 40S ribosomal protein S13  structural constituent of ribosome ? intracellular (IEA) ? ribosome (IEA) ? cytosolic small ribosomal subunit (sensu Eukaryota) ? protein biosynthesis ?  YDR064W ? structural constituent of ribosome ? cytosolic small ribosomal subunit (sensu Eukaryota) ? protein biosynthesis ?  0  BLAST| PF07\_0043 ? 60S ribosomal protein L34-a, putative  structural constituent of ribosome (IEA) ? intracellular (IEA) ? ribosome (IEA) ? protein biosynthesis (IEA) ? \*\* also with: YIL052C, clust.pair #24 YER056C-A ? structural constituent of ribosome ? cytosolic large ribosomal subunit (sensu Eukaryota) ? protein biosynthesis ?  1e-30  BLAST| PF07\_0043 ? 60S ribosomal protein L34-a, putative  structural constituent of ribosome (IEA) ? intracellular (IEA) ? ribosome (IEA) ? protein biosynthesis (IEA) ? \*\* also with: YER056C-A, clust.pair #24 YIL052C ? telomere maintenance ? structural constituent of ribosome ? cytosolic large ribosomal subunit (sensu Eukaryota) ? protein biosynthesis ?  1e-30  BLAST| PF10\_0103 ? eukaryotic translation initiation factor 2, beta, putative  RNA binding ? translation initiation factor activity ? eukaryotic translation initiation factor 2 complex ? translational initiation ?  YPL237W ? translation initiation factor activity ? ribosome ? eukaryotic translation initiation factor 2 complex ? translational initiation ?  6e-32  BLAST| PFD0770c ? ribosomal protein l15, putative  structural constituent of ribosome ? intracellular (IEA) ? ribosome ? protein biosynthesis ? \*\* also with: YLR029C, clust.pair #24 YMR121C ? RNA binding ? structural constituent of ribosome ? cytosolic large ribosomal subunit (sensu Eukaryota) ? protein biosynthesis ?  0  BLAST| PFD0770c ? ribosomal protein l15, putative  structural constituent of ribosome ? intracellular (IEA) ? ribosome ? protein biosynthesis ? \*\* also with: YMR121C, clust.pair #24 YLR029C ? RNA binding ? structural constituent of ribosome ? cytosolic large ribosomal subunit (sensu Eukaryota) ? protein biosynthesis ?  0  BLAST| PF14\_0296 ? ribosomal protein L14, putative  structural constituent of ribosome ? intracellular (IEA) ? ribosome (IEA) ? cytosolic small ribosomal subunit (sensu Eukaryota) ? protein biosynthesis ? apicoplast ? \*\* also with: YHL001W, clust.pair #24 YKL006W ? RNA binding ? structural constituent of ribosome ? cytosolic large ribosomal subunit (sensu Eukaryota) ? protein biosynthesis ?  0.004  BLAST| PF14\_0296 ? ribosomal protein L14, putative  structural constituent of ribosome ? intracellular (IEA) ? ribosome (IEA) ? cytosolic small ribosomal subunit (sensu Eukaryota) ? protein biosynthesis ? apicoplast ? \*\* also with: YKL006W, clust.pair #24 YHL001W ? RNA binding ? structural constituent of ribosome ? cytosolic large ribosomal subunit (sensu Eukaryota) ? protein biosynthesis ?  0.006  BLAST| PF08\_0039 ? ribosomal protein, putative  structural constituent of ribosome ? intracellular (IEA) ? ribosome (IEA) ? cytosolic large ribosomal subunit (sensu Eukaryota) ? protein biosynthesis ?  YLR061W ? structural constituent of ribosome ? cytosolic large ribosomal subunit (sensu Eukaryota) ? protein biosynthesis ?  1e-07  BLAST| PFB0885w ? 40S ribosomal protein S30, putative  structural constituent of ribosome ? intracellular (IEA) ? mitochondrion ? ribosome (IEA) ? cytosolic small ribosomal subunit (sensu Eukaryota) ? protein biosynthesis ?  YOR182C ? telomere maintenance ? structural constituent of ribosome ? cytosolic small ribosomal subunit (sensu Eukaryota) ? protein biosynthesis ?  4e-13  BLAST| PF08\_0075 ? 60S ribosomal protein L13, putative  structural constituent of ribosome ? intracellular (IEA) ? ribosome (IEA) ? cytosolic large ribosomal subunit (sensu Eukaryota) ? protein biosynthesis ? \*\* also with: YMR142C, clust.pair #24 YDL082W ? structural constituent of ribosome ? cytosolic large ribosomal subunit (sensu Eukaryota) ? protein biosynthesis ?  3e-18  BLAST| PF08\_0075 ? 60S ribosomal protein L13, putative  structural constituent of ribosome ? intracellular (IEA) ? ribosome (IEA) ? cytosolic large ribosomal subunit (sensu Eukaryota) ? protein biosynthesis ? \*\* also with: YDL082W, clust.pair #24 YMR142C ? telomere maintenance ? structural constituent of ribosome ? cytosolic large ribosomal subunit (sensu Eukaryota) ? protein biosynthesis ?  3e-18  BLAST| PFC0200w ? 60S Ribosomal protein L44, putative  structural constituent of ribosome ? intracellular (IEA) ? ribosome (IEA) ? cytosolic large ribosomal subunit (sensu Eukaryota) ? protein biosynthesis ? \*\* also with: YNL162W, clust.pair #24 YHR141C ? structural constituent of ribosome ? cytosolic large ribosomal subunit (sensu Eukaryota) ? protein biosynthesis ?  6e-30  BLAST| PFC0200w ? 60S Ribosomal protein L44, putative  structural constituent of ribosome ? intracellular (IEA) ? ribosome (IEA) ? cytosolic large ribosomal subunit (sensu Eukaryota) ? protein biosynthesis ? \*\* also with: YHR141C, clust.pair #24 YNL162W ? structural constituent of ribosome ? cytosolic large ribosomal subunit (sensu Eukaryota) ? protein biosynthesis ?  6e-30  BLAST| PF10\_0264 ? 40S ribosomal protein, putative  structural constituent of ribosome ? intracellular (IEA) ? ribosome (IEA) ? cytosolic small ribosomal subunit (sensu Eukaryota) ? protein biosynthesis ? small ribosomal subunit (IEA) ? \*\* also with: YLR048W, clust.pair #24 YGR214W ? ribosomal small subunit assembly and maintenance ? structural constituent of ribosome ? cytosolic small ribosomal subunit (sensu Eukaryota) ? protein biosynthesis ?  0  BLAST| PF10\_0264 ? 40S ribosomal protein, putative  structural constituent of ribosome ? intracellular (IEA) ? ribosome (IEA) ? cytosolic small ribosomal subunit (sensu Eukaryota) ? protein biosynthesis ? small ribosomal subunit (IEA) ? \*\* also with: YGR214W, clust.pair #24 YLR048W ? ribosomal small subunit assembly and maintenance ? structural constituent of ribosome ? cytosolic small ribosomal subunit (sensu Eukaryota) ? protein biosynthesis ?  0  BLAST| PF10\_0043 ? ribosomal protein L13, putative  structural constituent of ribosome ? intracellular (IEA) ? ribosome (IEA) ? cytosolic large ribosomal subunit (sensu Eukaryota) ? protein biosynthesis ? large ribosomal subunit (IEA) ? \*\* also with: YNL069C, clust.pair #24 YIL133C ? RNA binding ? structural constituent of ribosome ? cytosolic large ribosomal subunit (sensu Eukaryota) ? protein biosynthesis ?  0  BLAST| PF10\_0043 ? ribosomal protein L13, putative  structural constituent of ribosome ? intracellular (IEA) ? ribosome (IEA) ? cytosolic large ribosomal subunit (sensu Eukaryota) ? protein biosynthesis ? large ribosomal subunit (IEA) ? \*\* also with: YIL133C, clust.pair #24 YNL069C ? RNA binding ? structural constituent of ribosome ? cytosolic large ribosomal subunit (sensu Eukaryota) ? protein biosynthesis ?  0  BLAST| PFC0290w ? 40S ribosomal protein S23, putative  nucleic acid binding (IEA) ? structural constituent of ribosome ? intracellular (IEA) ? ribosome (IEA) ? cytosolic small ribosomal subunit (sensu Eukaryota) ? protein biosynthesis ? small ribosomal subunit (IEA) ? \*\* also with: YPR132W, clust.pair #24 YGR118W ? telomere maintenance ? structural constituent of ribosome ? cytosolic small ribosomal subunit (sensu Eukaryota) ? protein biosynthesis ? regulation of translational fidelity ?  0  BLAST| PFC0290w ? 40S ribosomal protein S23, putative  nucleic acid binding (IEA) ? structural constituent of ribosome ? intracellular (IEA) ? ribosome (IEA) ? cytosolic small ribosomal subunit (sensu Eukaryota) ? protein biosynthesis ? small ribosomal subunit (IEA) ? \*\* also with: YGR118W, clust.pair #24 YPR132W ? telomere maintenance ? structural constituent of ribosome ? cytosolic small ribosomal subunit (sensu Eukaryota) ? protein biosynthesis ? regulation of translational fidelity ?  0  BLAST| MAL7P1.81 ? eukaryotic translation initiation factor 3 37.28 kDa subunit, putative  translation initiation factor activity ? eukaryotic translation initiation factor 3 complex ? regulation of translational initiation ?  YMR146C ? translation initiation factor activity ? eukaryotic translation initiation factor 3 complex ? translational initiation ?  0  BLAST| PF10\_0272 ? ribosomal protein L3, putative  structural constituent of ribosome ? intracellular (IEA) ? mitochondrion ? ribosome (IEA) ? cytosolic large ribosomal subunit (sensu Eukaryota) ? protein biosynthesis ?  YOR063W ? ribosomal large subunit assembly and maintenance ? structural constituent of ribosome ? cytosolic large ribosomal subunit (sensu Eukaryota) ? protein biosynthesis ?  0  BLAST| PFE0975c ? 40S ribosomal subunit protein S24, putative  structural constituent of ribosome (IEA) ? intracellular (IEA) ? ribosome (IEA) ? protein biosynthesis (IEA) ?  YER074W ? structural constituent of ribosome ? mitochondrion ? cytosolic small ribosomal subunit (sensu Eukaryota) ? protein biosynthesis ?  3e-33  BLAST| PFE1005w ? 40S ribosomal subunit protein S9, putative  RNA binding (IEA) ? structural constituent of ribosome (IEA) ? intracellular (IEA) ? ribosome (IEA) ? protein biosynthesis (IEA) ? small ribosomal subunit (IEA) ? \*\* also with: YPL081W, clust.pair #24 YBR189W ? structural constituent of ribosome ? small nucleolar ribonucleoprotein complex ? cytosolic small ribosomal subunit (sensu Eukaryota) ? protein biosynthesis ? regulation of translational fidelity ?  0  BLAST| PFE1005w ? 40S ribosomal subunit protein S9, putative  RNA binding (IEA) ? structural constituent of ribosome (IEA) ? intracellular (IEA) ? ribosome (IEA) ? protein biosynthesis (IEA) ? small ribosomal subunit (IEA) ? \*\* also with: YBR189W, clust.pair #24 YPL081W ? structural constituent of ribosome ? small nucleolar ribonucleoprotein complex ? cytoplasm ? cytosolic small ribosomal subunit (sensu Eukaryota) ? protein biosynthesis ? regulation of translational fidelity ?  0  BLAST| PF11\_0260 ? ribosomal protein L35, putative  structural constituent of ribosome ? intracellular (IEA) ? ribosome (IEA) ? protein biosynthesis ? large ribosomal subunit ? \*\* also with: YDL191W, clust.pair #24 YDL136W ? structural constituent of ribosome ? cytosolic large ribosomal subunit (sensu Eukaryota) ? protein biosynthesis ?  3e-08  BLAST| PF11\_0260 ? ribosomal protein L35, putative  structural constituent of ribosome ? intracellular (IEA) ? ribosome (IEA) ? protein biosynthesis ? large ribosomal subunit ? \*\* also with: YDL136W, clust.pair #24 YDL191W ? structural constituent of ribosome ? cytosolic large ribosomal subunit (sensu Eukaryota) ? protein biosynthesis ?  3e-08  BLAST| PF11\_0106 ? 60S Ribosomal protein L36, putative  structural constituent of ribosome ? intracellular (IEA) ? ribosome (IEA) ? cytosolic large ribosomal subunit (sensu Eukaryota) ? protein biosynthesis ? membrane ?  YMR194W ? RNA binding ? structural constituent of ribosome ? cytosolic large ribosomal subunit (sensu Eukaryota) ? protein biosynthesis ?  8e-13  BLAST| PF14\_0141 ? ribosomal protein L10, putative  structural constituent of ribosome ? intracellular (IEA) ? ribosome (IEA) ? cytosolic large ribosomal subunit (sensu Eukaryota) ? protein biosynthesis ?  YLR075W ? ribosomal large subunit assembly and maintenance ? structural constituent of ribosome ? cytosolic large ribosomal subunit (sensu Eukaryota) ? protein biosynthesis ?  0  BLAST| PF11\_0313 ? ribosomal phosphoprotein P0  structural constituent of ribosome ? intracellular (IEA) ? mitochondrion ? ribosome (IEA) ? cytosolic large ribosomal subunit (sensu Eukaryota) ? protein biosynthesis ? translational elongation (IEA) ? ribosome biogenesis and assembly (IEA) ?  YLR340W ? ribosomal large subunit assembly and maintenance ? structural constituent of ribosome ? cytosolic large ribosomal subunit (sensu Eukaryota) ? protein biosynthesis ? translational elongation ?  0  BLAST| PF14\_0627 ? ribosomal protein S3, putative  nucleic acid binding (IEA) ? structural constituent of ribosome ? intracellular (IEA) ? ribosome (IEA) ? cytosolic small ribosomal subunit (sensu Eukaryota) ? protein biosynthesis ? small ribosomal subunit (IEA) ?  YNL178W ? structural constituent of ribosome ? cytosolic small ribosomal subunit (sensu Eukaryota) ? protein biosynthesis ? response to DNA damage stimulus ? nucleolar preribosome, small subunit precursor ?  0  BLAST| PF14\_0585 ? ribosomal protein S28e, putative  structural constituent of ribosome ? intracellular (IEA) ? ribosome (IEA) ? cytosolic small ribosomal subunit (sensu Eukaryota) ? protein biosynthesis ? \*\* also with: YOR167C, clust.pair #24 YLR264W ? telomere maintenance ? structural constituent of ribosome ? cytosolic small ribosomal subunit (sensu Eukaryota) ? protein biosynthesis ?  5e-12  BLAST| PF14\_0585 ? ribosomal protein S28e, putative  structural constituent of ribosome ? intracellular (IEA) ? ribosome (IEA) ? cytosolic small ribosomal subunit (sensu Eukaryota) ? protein biosynthesis ? \*\* also with: YLR264W, clust.pair #24 YOR167C ? structural constituent of ribosome ? cytosolic small ribosomal subunit (sensu Eukaryota) ? protein biosynthesis ?  5e-12  BLAST| MAL13P1.209 ? 60S ribosomal subunit porotein L18, putative  structural constituent of ribosome ? intracellular (IEA) ? ribosome (IEA) ? cytosolic large ribosomal subunit (sensu Eukaryota) ? protein biosynthesis ? \*\* also with: YOL120C, clust.pair #24 YNL301C ? structural constituent of ribosome ? cytosolic large ribosomal subunit (sensu Eukaryota) ? protein biosynthesis ?  8.00001e-42  BLAST| MAL13P1.209 ? 60S ribosomal subunit porotein L18, putative  structural constituent of ribosome ? intracellular (IEA) ? ribosome (IEA) ? cytosolic large ribosomal subunit (sensu Eukaryota) ? protein biosynthesis ? \*\* also with: YNL301C, clust.pair #24 YOL120C ? structural constituent of ribosome ? cytosolic large ribosomal subunit (sensu Eukaryota) ? protein biosynthesis ?  8.00001e-42  BLAST| PF07\_0088 ? 40S ribosomal protein S5, putative  structural constituent of ribosome ? intracellular (IEA) ? ribosome (IEA) ? cytosolic small ribosomal subunit (sensu Eukaryota) ? protein biosynthesis ? small ribosomal subunit (IEA) ?  YJR123W ? structural constituent of ribosome ? cytosolic small ribosomal subunit (sensu Eukaryota) ? protein biosynthesis ?  0  BLAST| PFE0845c ? 60S ribosomal subunit protein L8, putative  nucleic acid binding (IEA) ? structural constituent of ribosome ? intracellular (IEA) ? ribosome (IEA) ? protein biosynthesis ? large ribosomal subunit ? \*\* also with: YIL018W, clust.pair #24 YFR031C-A ? structural constituent of ribosome ? cytosolic large ribosomal subunit (sensu Eukaryota) ? protein biosynthesis ?  0  BLAST| PFE0845c ? 60S ribosomal subunit protein L8, putative  nucleic acid binding (IEA) ? structural constituent of ribosome ? intracellular (IEA) ? ribosome (IEA) ? protein biosynthesis ? large ribosomal subunit ? \*\* also with: YFR031C-A, clust.pair #24 YIL018W ? structural constituent of ribosome ? cytosolic large ribosomal subunit (sensu Eukaryota) ? protein biosynthesis ? response to drug ?  0  BLAST| PF13\_0129 ? ribosomal protein L6 homologue, putative  RNA binding ? structural constituent of ribosome ? intracellular (IEA) ? ribosome (IEA) ? protein biosynthesis ? large ribosomal subunit ? \*\* also with: YNL067W, clust.pair #24 YGL147C ? structural constituent of ribosome ? cytosolic large ribosomal subunit (sensu Eukaryota) ? protein biosynthesis ?  9.99995e-41  BLAST| PF13\_0129 ? ribosomal protein L6 homologue, putative  RNA binding ? structural constituent of ribosome ? intracellular (IEA) ? ribosome (IEA) ? protein biosynthesis ? large ribosomal subunit ? \*\* also with: YGL147C, clust.pair #24 YNL067W ? structural constituent of ribosome ? cytosolic large ribosomal subunit (sensu Eukaryota) ? protein biosynthesis ?  9.99995e-41  BLAST| PF13\_0049 ? 60S ribosomal protein L24, putative  structural constituent of ribosome ? intracellular (IEA) ? ribosome (IEA) ? cytosolic large ribosomal subunit (sensu Eukaryota) ? protein biosynthesis ? \*\* also with: YGL031C, clust.pair #24 YGR148C ? RNA binding ? structural constituent of ribosome ? cytosolic large ribosomal subunit (sensu Eukaryota) ? protein biosynthesis ?  3e-10  BLAST| PF13\_0049 ? 60S ribosomal protein L24, putative  structural constituent of ribosome ? intracellular (IEA) ? ribosome (IEA) ? cytosolic large ribosomal subunit (sensu Eukaryota) ? protein biosynthesis ? \*\* also with: YGR148C, clust.pair #24 YGL031C ? RNA binding ? structural constituent of ribosome ? cytosolic large ribosomal subunit (sensu Eukaryota) ? protein biosynthesis ?  4e-10  BLAST| PFC0400w ? 60S Acidic ribosomal protein P2  structural constituent of ribosome (IEA) ? intracellular (IEA) ? ribosome (IEA) ? cytosolic large ribosomal subunit (sensu Eukaryota) ? translational elongation ? large ribosomal subunit ? \*\* also with: YOL039W, clust.pair #24 YDR382W ? structural constituent of ribosome ? cytosolic large ribosomal subunit (sensu Eukaryota) ? protein biosynthesis ? translational elongation ?  9e-11  BLAST| PFC0400w ? 60S Acidic ribosomal protein P2  structural constituent of ribosome (IEA) ? intracellular (IEA) ? ribosome (IEA) ? cytosolic large ribosomal subunit (sensu Eukaryota) ? translational elongation ? large ribosomal subunit ? \*\* also with: YDR382W, clust.pair #24 YOL039W ? structural constituent of ribosome ? cytosolic large ribosomal subunit (sensu Eukaryota) ? protein biosynthesis ? translational elongation ?  2e-09  BLAST| PF11\_0065 ? ribosomal protein S4, putative  RNA binding ? structural constituent of ribosome ? intracellular (IEA) ? mitochondrion ? ribosome (IEA) ? protein biosynthesis ? small ribosomal subunit ? \*\* also with: YJR145C, clust.pair #24 YHR203C ? telomere maintenance ? structural constituent of ribosome ? cytosolic small ribosomal subunit (sensu Eukaryota) ? protein biosynthesis ?  0  BLAST| PF11\_0065 ? ribosomal protein S4, putative  RNA binding ? structural constituent of ribosome ? intracellular (IEA) ? mitochondrion ? ribosome (IEA) ? protein biosynthesis ? small ribosomal subunit ? \*\* also with: YHR203C, clust.pair #24 YJR145C ? telomere maintenance ? structural constituent of ribosome ? cytoplasm ? cytosolic small ribosomal subunit (sensu Eukaryota) ? protein biosynthesis ? processing of 20S pre-rRNA ?  0  BLAST| PF07\_0079 ? 60S ribosomal protein L11a, putative  structural constituent of ribosome ? intracellular (IEA) ? ribosome (IEA) ? cytosolic large ribosomal subunit (sensu Eukaryota) ? protein biosynthesis ? \*\* also with: YPR102C, clust.pair #24 YGR085C ? ribosomal large subunit assembly and maintenance ? structural constituent of ribosome ? cytosolic large ribosomal subunit (sensu Eukaryota) ? protein biosynthesis ?  0  BLAST| PF07\_0079 ? 60S ribosomal protein L11a, putative  structural constituent of ribosome ? intracellular (IEA) ? ribosome (IEA) ? cytosolic large ribosomal subunit (sensu Eukaryota) ? protein biosynthesis ? \*\* also with: YGR085C, clust.pair #24 YPR102C ? ribosomal large subunit assembly and maintenance ? structural constituent of ribosome ? cytosolic large ribosomal subunit (sensu Eukaryota) ? protein biosynthesis ?  0  BLAST| PFE0350c ? 60S ribosomal subunit protein L4%2FL1, putative  RNA binding ? structural constituent of ribosome ? intracellular (IEA) ? ribosome (IEA) ? protein biosynthesis ? large ribosomal subunit ? \*\* also with: YDR012W, clust.pair #24 YBR031W ? structural constituent of ribosome ? cytosolic large ribosomal subunit (sensu Eukaryota) ? protein biosynthesis ?  0  BLAST| PFE0350c ? 60S ribosomal subunit protein L4%2FL1, putative  RNA binding ? structural constituent of ribosome ? intracellular (IEA) ? ribosome (IEA) ? protein biosynthesis ? large ribosomal subunit ? \*\* also with: YBR031W, clust.pair #24 YDR012W ? structural constituent of ribosome ? cytoplasm ? cytosolic large ribosomal subunit (sensu Eukaryota) ? protein biosynthesis ?  0  BLAST | | | | | | | | | | | | | | | | | | | | | | | | | | | | | | | | | | | | | | | | | | | | | | | | | | | | | | | | | | | | | | | | | | | | | | | | | | | | | | | | | | | | | | | | | | | | | | | | | | | | | | | | | | | | | | | | | | | | | | | | | | | | | | | | | | | | | | | | | | | | | | | | | | | | | | | | | | | | | | | | | | | | | | | |

## Cluster Pair #25: 10 gene pairs.

|  |  |  |  |  |  |  |  |  |  |  |  |  |  |  |  |  |  |  |  |  |  |  |  |  |  |  |  |  |  |  |  |  |
| --- | --- | --- | --- | --- | --- | --- | --- | --- | --- | --- | --- | --- | --- | --- | --- | --- | --- | --- | --- | --- | --- | --- | --- | --- | --- | --- | --- | --- | --- | --- | --- | --- |
| P.falciparum S.cerevisiae Blast evalue|  |  |  |  |  |  |  |  |  |  |  |  |  |  |  |  |  |  |  |  |  |  |  |  |  |  |  |  |  |  | | --- | --- | --- | --- | --- | --- | --- | --- | --- | --- | --- | --- | --- | --- | --- | --- | --- | --- | --- | --- | --- | --- | --- | --- | --- | --- | --- | --- | --- | --- | | PF14\_0125 ? deoxyhypusine synthase  protein biosynthesis ? hypusine biosynthesis from peptidyl-lysine ? membrane ? spermidine catabolism to deoxyhypusine, using deoxyhypusine synthase ?  YHR068W ? cytoplasm ? hypusine biosynthesis from peptidyl-lysine ? transferase activity, transferring alkyl or aryl (other than methyl) groups ?  9.80909e-45  BLAST| PF13\_0214 ? elongation factor 1-gamma, putative  translation elongation factor activity ? glutathione transferase activity ? eukaryotic translation elongation factor 1 complex ? translational elongation ? \*\* also with: YPL048W, clust.pair #11 YKL081W ? translation elongation factor activity ? mitochondrion ? ribosome ? eukaryotic translation elongation factor 1 complex ? translational elongation ?  4e-24  BLAST| PFD1070w ? eukaryotic initiation factor, putative  nucleic acid binding (IEA) ? translation initiation factor activity ? ATP-dependent RNA helicase activity ? helicase activity (IEA) ? ATP binding (IEA) ? regulation of translational initiation ? ATP-dependent helicase activity (IEA) ? eukaryotic translation initiation factor 4F complex ? \*\* also with: YKR059W, clust.pair #25 YJL138C ? RNA helicase activity ? translation initiation factor activity ? cytoplasm ? ribosome ? translational initiation ? regulation of translational initiation ? eukaryotic translation initiation factor 4F complex ?  0  BLAST| PFD1070w ? eukaryotic initiation factor, putative  nucleic acid binding (IEA) ? translation initiation factor activity ? ATP-dependent RNA helicase activity ? helicase activity (IEA) ? ATP binding (IEA) ? regulation of translational initiation ? ATP-dependent helicase activity (IEA) ? eukaryotic translation initiation factor 4F complex ? \*\* also with: YJL138C, clust.pair #25 YKR059W ? telomere maintenance ? translation initiation factor activity ? ATP-dependent RNA helicase activity ? ribosome ? translational initiation ? eukaryotic translation initiation factor 4F complex ?  0  BLAST| PF07\_0117 ? eukaryotic translation initiation factor 2 alpha subunit, putative  nucleic acid binding (IEA) ? RNA binding (IEA) ? translation initiation factor activity ? eukaryotic translation initiation factor 2 complex ? protein biosynthesis (IEA) ? translational initiation ?  YJR007W ? translation initiation factor activity ? cytoplasm ? ribosome ? eukaryotic translation initiation factor 2 complex ? translational initiation ?  0  BLAST| PF14\_0428 ? histidine -- tRNA ligase, putative  tRNA ligase activity (IEA) ? histidine-tRNA ligase activity ? ATP binding (IEA) ? protein biosynthesis (IEA) ? tRNA aminoacylation for protein translation ?  YPR033C ? histidine-tRNA ligase activity ? cytoplasm ? mitochondrion ? histidyl-tRNA aminoacylation ?  0  BLAST| PF08\_0111 ? hypothetical protein  nucleic acid binding (IEA) ? helicase activity (IEA) ? ATP binding (IEA) ? ATP-dependent helicase activity (IEA) ?  YDL084W ? transcription export complex ? U2-type nuclear mRNA branch site recognition ? nuclear mRNA splicing, via spliceosome ? chromosome, telomeric region ? RNA binding ? ATP-dependent RNA helicase activity ? protein binding ? nucleus ? spliceosome complex ? chromatin silencing at telomere ? mRNA-nucleus export ? RNA splicing factor activity, transesterification mechanism ?  3e-18  BLAST| PF13\_0170 ? glutaminyl-tRNA synthetase, putative  glutamate-tRNA ligase activity (IEA) ? glutamine-tRNA ligase activity (IEA) ? ATP binding (IEA) ? glutamyl-tRNA aminoacylation (IEA) ?  YOR168W ? glutamine-tRNA ligase activity ? cytoplasm ? glutaminyl-tRNA aminoacylation ?  0  BLAST| PFB0445c ? helicase, putative  nucleic acid binding (IEA) ? ATP-dependent RNA helicase activity ? helicase activity (IEA) ? ATP binding (IEA) ? ATP-dependent helicase activity (IEA) ?  YDL084W ? transcription export complex ? U2-type nuclear mRNA branch site recognition ? nuclear mRNA splicing, via spliceosome ? chromosome, telomeric region ? RNA binding ? ATP-dependent RNA helicase activity ? protein binding ? nucleus ? spliceosome complex ? chromatin silencing at telomere ? mRNA-nucleus export ? RNA splicing factor activity, transesterification mechanism ?  0  BLAST| PF14\_0230 ? Ribosomal protein family L5, putative  structural constituent of ribosome ? intracellular (IEA) ? mitochondrion ? ribosome (IEA) ? cytosolic large ribosomal subunit (sensu Eukaryota) ? protein biosynthesis ? 5S rRNA binding (IEA) ?  YPL131W ? ribosomal large subunit assembly and maintenance ? RNA binding ? structural constituent of ribosome ? cytosolic large ribosomal subunit (sensu Eukaryota) ? protein biosynthesis ?  0  BLAST | | | | | | | | | | | | | | | | | | | | | | | | | | | | | | | | |
