# Supplementary material for: Assessing functional annotation transfers with inter-species conserved coexpression: application to Plasmodium falciparum
Source: BMC Genomics. 2010 Jan 15;11:35. doi: 10.1186/1471-2164-11-35 (PMC2826313; doi:10.1186/1471-2164-11-35)
Supplement: Additional file 4 — Bozdech - Spellman analysis. This file presents the cluster pairs identified as revealing a conservation of coexpression when comparing the Bozdech and Spellman data. This file also provide additional information on the available functional annotations, as well as links to the BLAST alignments and the different databases (click on the '?'s to access PlasmoDB, SGD, and Amigo databases). Gene functional annotations are as follows. The short description immediately following each P. falciparum gene comes from PlasmoDB (red = functional gene, blue = putative gene, black = hypothetical gene). Other annotations are Gene Ontology annotations (red = Molecular Function, green = Biological Process, blue = Cellular Component). [file 1471-2164-11-35-S4.HTML]

# Bozdech - Spellman co-coexpression analysis

# 36 cluster pairs

## Cluster Pair #0: 4 gene pairs.

|  |  |  |  |  |  |  |  |  |  |  |  |  |  |  |
| --- | --- | --- | --- | --- | --- | --- | --- | --- | --- | --- | --- | --- | --- | --- |
| P.falciparum S.cerevisiae Blast evalue|  |  |  |  |  |  |  |  |  |  |  |  | | --- | --- | --- | --- | --- | --- | --- | --- | --- | --- | --- | --- | | PFL2440w ? DNA repair protein rhp16, putative  repairosome ? ubiquitin ligase complex (IEA) ? nucleic acid binding (IEA) ? DNA binding (IEA) ? helicase activity (IEA) ? ubiquitin-protein ligase activity (IEA) ? ATP binding ? nucleus (IEA) ? nucleotide-excision repair ? ATP-dependent helicase activity ? zinc ion binding (IEA) ? protein ubiquitination (IEA) ?  YBR114W ? repairosome ? nucleotide excision repair factor 4 complex ? nucleotide-excision repair, DNA damage recognition ? damaged DNA binding ? DNA-dependent ATPase activity ?  0  BLAST| PF10\_0310 ? hypothetical protein   YHR156C ? chromatin ? protein binding ? nucleus ? snRNP U5 ?  4e-09  BLAST| PFA0580c ? TatD-like deoxyribonuclease, putative  deoxyribonuclease activity ? DNA metabolism ? apicoplast ?  YBL055C ? endonuclease activity ? cytoplasm ? DNA fragmentation during apoptosis ? response to oxidative stress ? 3'-5'-exodeoxyribonuclease activity ?  6e-38  BLAST| PFC0785c ? proteasome regulatory protein, putative  protein binding (IEA) ? proteasome regulatory particle (sensu Eukaryota) ? ubiquitin-dependent protein catabolism ?  YIL007C ? cytoplasm ? ubiquitin-dependent protein catabolism ?  2e-14  BLAST | | | | | | | | | | | | | | |

## Cluster Pair #1: 10 gene pairs.

|  |  |  |  |  |  |  |  |  |  |  |  |  |  |  |  |  |  |  |  |  |  |  |  |  |  |  |  |  |  |  |  |  |
| --- | --- | --- | --- | --- | --- | --- | --- | --- | --- | --- | --- | --- | --- | --- | --- | --- | --- | --- | --- | --- | --- | --- | --- | --- | --- | --- | --- | --- | --- | --- | --- | --- |
| P.falciparum S.cerevisiae Blast evalue|  |  |  |  |  |  |  |  |  |  |  |  |  |  |  |  |  |  |  |  |  |  |  |  |  |  |  |  |  |  | | --- | --- | --- | --- | --- | --- | --- | --- | --- | --- | --- | --- | --- | --- | --- | --- | --- | --- | --- | --- | --- | --- | --- | --- | --- | --- | --- | --- | --- | --- | | PF14\_0663 ? hypothetical protein  metalloendopeptidase activity (IEA) ? proteolysis and peptidolysis (IEA) ? pathogenesis (IEA) ? metal ion binding (IEA) ?  YDL167C ? cytoplasm ? ribosome biogenesis and assembly ?  3e-05  BLAST| PFI1060w ? hypothetical protein   YDL189W ? cytoplasm ? galactose metabolism ?  0.016  BLAST| PF13\_0278 ? hypothetical protein  nucleic acid binding (IEA) ?  YDL167C ? cytoplasm ? ribosome biogenesis and assembly ?  5e-05  BLAST| MAL8P1.67 ? hypothetical protein, conserved   YDR339C ? nucleolus ? 35S primary transcript processing ? mitochondrion organization and biogenesis ?  0  BLAST| PF10\_0123 ? GMP synthetase  catalytic activity (IEA) ? GMP synthase activity ? GMP synthase (glutamine-hydrolyzing) activity (IEA) ? ATP binding (IEA) ? purine nucleotide biosynthesis ? GMP biosynthesis (IEA) ? glutamine metabolism (IEA) ?  YMR217W ? GMP synthase (glutamine-hydrolyzing) activity ? GMP metabolism ?  0  BLAST| PFI0415c ? ribosomal RNA methyltransferase, putative   YBR061C ? cytoplasm ? protein biosynthesis ? tRNA methyltransferase activity ? tRNA methylation ?  6.00036e-42  BLAST| PFE0885w ? eukaryotic translation initiation factor 3 subunit, putative  nucleic acid binding (IEA) ?  YOR361C ? translation initiation factor activity ? cytoplasm ? eukaryotic translation initiation factor 3 complex ? translational initiation ?  0  BLAST| PFB0375w ? hypothetical protein  metalloendopeptidase activity (IEA) ? proteolysis and peptidolysis (IEA) ? pathogenesis (IEA) ? membrane ? metal ion binding (IEA) ?  YDL167C ? cytoplasm ? ribosome biogenesis and assembly ?  0.0004  BLAST| PF10\_0197 ? hypothetical protein  apicoplast ?  YNL022C ? nucleus ? ribosome biogenesis and assembly ?  9e-06  BLAST| PFL0335c ? eukaryotic translation initiation factor 5, putative  translation initiation factor activity ? translational initiation (IEA) ? regulation of translational initiation ?  YPR041W ? translation initiation factor activity ? GTPase activator activity ? cytosolic small ribosomal subunit (sensu Eukaryota) ? regulation of translational initiation ? mature ribosome assembly ?  6e-33  BLAST | | | | | | | | | | | | | | | | | | | | | | | | | | | | | | | | |

## Cluster Pair #2: 9 gene pairs.

|  |  |  |  |  |  |  |  |  |  |  |  |  |  |  |  |  |  |  |  |  |  |  |  |  |  |  |  |  |  |
| --- | --- | --- | --- | --- | --- | --- | --- | --- | --- | --- | --- | --- | --- | --- | --- | --- | --- | --- | --- | --- | --- | --- | --- | --- | --- | --- | --- | --- | --- |
| P.falciparum S.cerevisiae Blast evalue|  |  |  |  |  |  |  |  |  |  |  |  |  |  |  |  |  |  |  |  |  |  |  |  |  |  |  | | --- | --- | --- | --- | --- | --- | --- | --- | --- | --- | --- | --- | --- | --- | --- | --- | --- | --- | --- | --- | --- | --- | --- | --- | --- | --- | --- | | PF10\_0300 ? hypothetical protein, conserved  RNA binding (IEA) ? RNA processing (IEA) ? RNA methyltransferase activity (IEA) ?  YDL112W ? cytoplasm ? tRNA (guanosine) methyltransferase activity ? tRNA methylation ?  0.002  BLAST| MAL13P1.170 ? hypothetical protein  nucleic acid binding (IEA) ? nucleotidyltransferase activity (IEA) ?  YNL299W ? DNA-directed DNA polymerase activity ? polynucleotide adenylyltransferase activity ? nucleus ? nucleolus ? mitotic sister chromatid cohesion ? rRNA catabolism ? snRNA catabolism ? snoRNA catabolism ?  5e-19  BLAST| PF11\_0316 ? hypothetical protein   YOL125W ? nucleus ? cytoplasm ? tRNA methyltransferase activity ? tRNA methylation ?  2e-06  BLAST| PFA0480w ? phenylalanyl-tRNA synthetase beta chain, putative  tRNA ligase activity (IEA) ? phenylalanine-tRNA ligase activity ? ATP binding (IEA) ? cytoplasm (IEA) ? tRNA aminoacylation for protein translation (IEA) ? phenylalanyl-tRNA aminoacylation ? phenylalanine-tRNA ligase complex ?  YFL022C ? phenylalanine-tRNA ligase activity ? cytoplasm ? phenylalanyl-tRNA aminoacylation ? phenylalanine-tRNA ligase complex ?  0  BLAST| PFI0700c ? Met-10+ like protein, putative  apicoplast ?  YHR070W ? nucleus ? cytoplasm ? tRNA (guanine) methyltransferase activity ? tRNA methylation ? ribosome biogenesis and assembly ?  1e-19  BLAST| MAL13P1.36 ? hypothetical protein, conserved  nucleic acid binding (IEA) ?  YOL142W ? 3'-5'-exoribonuclease activity ? nuclear exosome (RNase complex) ? cytoplasmic exosome (RNase complex) ? RNA binding ? 35S primary transcript processing ? mRNA catabolism ? ribosome assembly ?  3e-11  BLAST| PF13\_0354 ? alanine--tRNA ligase, putative  alanine-tRNA ligase activity ? ATP binding (IEA) ? alanyl-tRNA aminoacylation ? apicoplast ?  YOR335C ? alanine-tRNA ligase activity ? cytoplasm ? mitochondrion ? alanyl-tRNA aminoacylation ?  0  BLAST| PF14\_0620 ? hypothetical protein   YKR079C ? nucleus ? cytoplasm ? mitochondrion ? purine nucleotide binding ? removal of tRNA 3'-trailer sequence ? 3'-tRNA processing endoribonuclease activity ?  9e-23  BLAST| MAL7P1.28 ? hypothetical protein  tRNA 5'-leader removal (IEA) ? ribonuclease P activity (IEA) ?  YNL221C ? ribonuclease MRP activity ? ribonuclease MRP complex ? ribonuclease P activity ? nucleolar ribonuclease P complex ? rRNA processing ? tRNA processing ?  0.014  BLAST | | | | | | | | | | | | | | | | | | | | | | | | | | | | | |

## Cluster Pair #3: 8 gene pairs.

|  |  |  |  |  |  |  |  |  |  |  |  |  |  |  |  |  |  |  |  |  |  |  |  |  |  |  |
| --- | --- | --- | --- | --- | --- | --- | --- | --- | --- | --- | --- | --- | --- | --- | --- | --- | --- | --- | --- | --- | --- | --- | --- | --- | --- | --- |
| P.falciparum S.cerevisiae Blast evalue|  |  |  |  |  |  |  |  |  |  |  |  |  |  |  |  |  |  |  |  |  |  |  |  | | --- | --- | --- | --- | --- | --- | --- | --- | --- | --- | --- | --- | --- | --- | --- | --- | --- | --- | --- | --- | --- | --- | --- | --- | | PF11\_0445 ? DNA-directed RNA polymerase I, putative  DNA binding (IEA) ? DNA-directed RNA polymerase activity (IEA) ? transcription (IEA) ? protein dimerization activity (IEA) ?  YPR110C ? DNA-directed RNA polymerase activity ? DNA-directed RNA polymerase III complex ? DNA-directed RNA polymerase I complex ? transcription from RNA polymerase I promoter ? transcription from RNA polymerase III promoter ? ribosome biogenesis and assembly ?  9.94922e-44  BLAST| PF11\_0439 ? hypothetical protein   YPL239W ? cytoplasm ? response to osmotic stress ? response to oxidative stress ? ribosomal small subunit biogenesis ?  0.003  BLAST| PF14\_0072 ? hypothetical protein, conserved   YNR046W ? tRNA (guanine-N2-)-methyltransferase activity ? nucleus ? nucleolus ? cytoplasm ? zinc ion binding ? tRNA methylation ?  2e-09  BLAST| PFI1235w ? hypothetical protein   YDR083W ? telomere maintenance ? nucleolus ? rRNA processing ? methyltransferase activity ? ribosome biogenesis and assembly ?  6e-24  BLAST| PF10\_0209 ? RNA helicase, putative  nucleic acid binding (IEA) ? RNA helicase activity ? helicase activity (IEA) ? ATP binding (IEA) ? ATP-dependent helicase activity (IEA) ?  YGL171W ? ATP-dependent RNA helicase activity ? nucleolus ? 35S primary transcript processing ? ATPase activity ?  2e-36  BLAST| PFB0826c ? hypothetical protein  \*\* also with: YPL146C, clust.pair #3 YPR143W ? nucleus ? nucleolus ? processing of 27S pre-rRNA ? ribosome biogenesis and assembly ?  0.029  BLAST| PFB0826c ? hypothetical protein  \*\* also with: YPR143W, clust.pair #3 YPL146C ? ribosomal large subunit-nucleus export ? nucleus ? nucleolus ? rRNA binding ? processing of 27S pre-rRNA ? ribosome biogenesis and assembly ?  0.05  BLAST| PF08\_0123 ? hypothetical protein   YPL212C ? nucleus ? tRNA modification ? tRNA-pseudouridine synthase activity ? ribosome biogenesis and assembly ?  2e-17  BLAST | | | | | | | | | | | | | | | | | | | | | | | | | | |

## Cluster Pair #4: 8 gene pairs.

|  |  |  |  |  |  |  |  |  |  |  |  |  |  |  |  |  |  |  |  |  |  |  |  |  |  |  |
| --- | --- | --- | --- | --- | --- | --- | --- | --- | --- | --- | --- | --- | --- | --- | --- | --- | --- | --- | --- | --- | --- | --- | --- | --- | --- | --- |
| P.falciparum S.cerevisiae Blast evalue|  |  |  |  |  |  |  |  |  |  |  |  |  |  |  |  |  |  |  |  |  |  |  |  | | --- | --- | --- | --- | --- | --- | --- | --- | --- | --- | --- | --- | --- | --- | --- | --- | --- | --- | --- | --- | --- | --- | --- | --- | | PF14\_0207 ? RNA polymerase subunit, putative  DNA binding (IEA) ? DNA-directed RNA polymerase activity ? nucleus (IEA) ? transcription (IEA) ? regulation of transcription, DNA-dependent ? membrane ?  YNR003C ? DNA-directed RNA polymerase activity ? nucleus ? DNA-directed RNA polymerase III complex ? cytoplasm ? transcription from RNA polymerase III promoter ? ribosome biogenesis and assembly ?  3e-11  BLAST| PF10\_0150 ? methionine aminopeptidase, putative  methionyl aminopeptidase activity ? regulation of protein biosynthesis ? regulation of translation ? protein modification ? proteolysis and peptidolysis ? metalloexopeptidase activity (IEA) ?  YLR244C ? methionyl aminopeptidase activity ? cytosolic ribosome (sensu Eukaryota) ? proteolysis and peptidolysis ?  0  BLAST| PFE0730c ? ribose 5-phosphate epimerase, putative  ribose-5-phosphate isomerase activity ? pentose-phosphate shunt, non-oxidative branch ?  YOR095C ? ribose-5-phosphate isomerase activity ? nucleus ? cytoplasm ? pentose-phosphate shunt ? pyridoxine biosynthesis ? ribosome biogenesis and assembly ?  2e-25  BLAST| PF11\_0101 ? hypothetical protein   YBR155W ? cytoplasm ? protein folding ? Hsp70 protein binding ?  0.025  BLAST| PFC0350c ? T-complex protein eta subunit, putative  protein binding (IEA) ? ATP binding (IEA) ? chaperonin-containing T-complex ? protein folding ? cellular protein metabolism (IEA) ? unfolded protein binding ?  YJL111W ? cytoplasm ? chaperonin-containing T-complex ? cytoskeleton ? protein folding ? cytoskeleton organization and biogenesis ? unfolded protein binding ?  0  BLAST| PFE1370w ? hsp70 interacting protein, putative   YBR155W ? cytoplasm ? protein folding ? Hsp70 protein binding ?  1e-06  BLAST| PF13\_0205 ? tryptophan--tRNA ligase, putative  tRNA ligase activity (IEA) ? tryptophan-tRNA ligase activity ? ATP binding (IEA) ? tRNA aminoacylation for protein translation (IEA) ? tryptophanyl-tRNA aminoacylation ? apicoplast ?  YOL097C ? tryptophan-tRNA ligase activity ? cytoplasm ? tryptophanyl-tRNA aminoacylation ?  0  BLAST| PFL2150c ? hypothetical protein, conserved   YOR091W ? cytoplasm ? ribosome ? ribosome biogenesis and assembly ?  3e-36  BLAST | | | | | | | | | | | | | | | | | | | | | | | | | | |

## Cluster Pair #5: 9 gene pairs.

|  |  |  |  |  |  |  |  |  |  |  |  |  |  |  |  |  |  |  |  |  |  |  |  |  |  |  |  |  |  |
| --- | --- | --- | --- | --- | --- | --- | --- | --- | --- | --- | --- | --- | --- | --- | --- | --- | --- | --- | --- | --- | --- | --- | --- | --- | --- | --- | --- | --- | --- |
| P.falciparum S.cerevisiae Blast evalue|  |  |  |  |  |  |  |  |  |  |  |  |  |  |  |  |  |  |  |  |  |  |  |  |  |  |  | | --- | --- | --- | --- | --- | --- | --- | --- | --- | --- | --- | --- | --- | --- | --- | --- | --- | --- | --- | --- | --- | --- | --- | --- | --- | --- | --- | | PFL0670c ? Bi-functional aminoacyl-tRNA synthetase, putative  tRNA ligase activity (IEA) ? glutamate-tRNA ligase activity ? proline-tRNA ligase activity ? ATP binding (IEA) ? cytoplasm (IEA) ? protein biosynthesis (IEA) ? tRNA aminoacylation for protein translation ? prolyl-tRNA aminoacylation (IEA) ?  YHR020W ? proline-tRNA ligase activity ? ribosome ? tRNA aminoacylation for protein translation ?  0  BLAST| PF14\_0486 ? elongation factor 2  translation elongation factor activity ? GTP binding ? protein biosynthesis (IEA) ? translational elongation ? \*\* also with: YOR133W, clust.pair #5 YDR385W ? translation elongation factor activity ? ribosome ? translational elongation ?  0  BLAST| PF14\_0486 ? elongation factor 2  translation elongation factor activity ? GTP binding ? protein biosynthesis (IEA) ? translational elongation ? \*\* also with: YDR385W, clust.pair #5 YOR133W ? translation elongation factor activity ? ribosome ? translational elongation ?  0  BLAST| PF14\_0028 ? hypothetical protein, conserved  nucleic acid binding (IEA) ? RNA binding (IEA) ? RNA processing (IEA) ? ATP biosynthesis (IEA) ? ATP synthesis coupled proton transport (IEA) ? proton-transporting two-sector ATPase complex (IEA) ? hydrogen-transporting ATP synthase activity, rotational mechanism (IEA) ? hydrogen-transporting ATPase activity, rotational mechanism (IEA) ?  YER165W ? nucleus ? cytoplasm ? ribosome ? regulation of translational initiation ? poly(A) binding ?  6e-06  BLAST| PF11\_0116 ? hypothetical protein   YBL024W ? nucleus ? tRNA (cytosine-5-)-methyltransferase activity ? tRNA methylation ? ribosome biogenesis and assembly ?  2e-09  BLAST| PFB0550w ? peptide chain release factor subunit 1, putative  translation release factor activity ? cytoplasm (IEA) ? translational termination (IEA) ? regulation of translational termination ? translation release factor activity, codon specific (IEA) ?  YBR143C ? cytokinesis ? cytosol ? translational termination ? translation release factor activity, codon specific ? translation release factor complex ?  0  BLAST| PFE0845c ? 60S ribosomal subunit protein L8, putative  nucleic acid binding (IEA) ? structural constituent of ribosome ? intracellular (IEA) ? ribosome (IEA) ? protein biosynthesis ? large ribosomal subunit ?  YIL018W ? structural constituent of ribosome ? cytosolic large ribosomal subunit (sensu Eukaryota) ? protein biosynthesis ? response to drug ?  0  BLAST| PF07\_0015 ? hypothetical protein  metalloendopeptidase activity (IEA) ? proteolysis and peptidolysis (IEA) ? pathogenesis (IEA) ? metal ion binding (IEA) ?  YBL024W ? nucleus ? tRNA (cytosine-5-)-methyltransferase activity ? tRNA methylation ? ribosome biogenesis and assembly ?  3e-39  BLAST| PF14\_0198 ? glycine -- tRNA ligase, putative  tRNA ligase activity (IEA) ? glycine-tRNA ligase activity ? ATP binding (IEA) ? protein biosynthesis (IEA) ? tRNA aminoacylation for protein translation (IEA) ? glycyl-tRNA aminoacylation ? apicoplast ?  YBR121C ? glycine-tRNA ligase activity ? cytoplasm ? mitochondrion ? transcription termination ? glycyl-tRNA aminoacylation ?  0  BLAST | | | | | | | | | | | | | | | | | | | | | | | | | | | | | |

## Cluster Pair #6: 7 gene pairs.

|  |  |  |  |  |  |  |  |  |  |  |  |  |  |  |  |  |  |  |  |  |  |  |  |
| --- | --- | --- | --- | --- | --- | --- | --- | --- | --- | --- | --- | --- | --- | --- | --- | --- | --- | --- | --- | --- | --- | --- | --- |
| P.falciparum S.cerevisiae Blast evalue|  |  |  |  |  |  |  |  |  |  |  |  |  |  |  |  |  |  |  |  |  | | --- | --- | --- | --- | --- | --- | --- | --- | --- | --- | --- | --- | --- | --- | --- | --- | --- | --- | --- | --- | --- | | MAL8P1.142 ? proteasome beta-subunit  endopeptidase activity ? threonine endopeptidase activity (IEA) ? proteasome core complex (sensu Eukaryota) ? ubiquitin-dependent protein catabolism (IEA) ?  YFR050C ? endopeptidase activity ? ubiquitin-dependent protein catabolism ? proteasome core complex, beta-subunit complex (sensu Eukaryota) ?  5e-26  BLAST| PFC0520w ? 26S proteasome regulatory subunit S14, putative  proteasome regulatory particle (sensu Eukaryota) ? ubiquitin-dependent protein catabolism ?  YFR052W ? endopeptidase activity ? ubiquitin-dependent protein catabolism ? proteasome regulatory particle, lid subcomplex (sensu Eukaryota) ?  4e-14  BLAST| PF14\_0178 ? hypothetical protein  ubiquitin-dependent protein catabolism (IEA) ? membrane ?  YGR048W ? protein binding ? endoplasmic reticulum ? mRNA processing ? ubiquitin-dependent protein catabolism ? protein transport ?  7e-34  BLAST| PF14\_0025 ? proteosome subunit, putative  endopeptidase activity ? proteasome regulatory particle (sensu Eukaryota) ? proteolysis and peptidolysis ?  YDL097C ? structural molecule activity ? ubiquitin-dependent protein catabolism ? proteasome regulatory particle, lid subcomplex (sensu Eukaryota) ?  8e-21  BLAST| PF08\_0071 ? Fe-superoxide dismutase  superoxide dismutase activity (IEA) ? superoxide metabolism ? response to oxidative stress ? iron superoxide dismutase activity ? metal ion binding (IEA) ?  YHR008C ? replicative cell aging ? age-dependent response to reactive oxygen species during chronological cell aging ? age-dependent response to oxidative stress during chronological cell aging ? mitochondrion ? mitochondrial matrix ? oxygen and reactive oxygen species metabolism ? manganese superoxide dismutase activity ?  6e-30  BLAST| PFL2415w ? Hbeta58%2FVps26 protein homolog, putative  intracellular protein transport (IEA) ? retrograde transport, endosome to Golgi ?  YJL053W ? endosome ? protein transporter activity ? retromer complex ? retrograde transport, endosome to Golgi ? protein retention in Golgi ?  0  BLAST| MAL8P1.128 ? proteasome subunit alpha, putative  endopeptidase activity ? threonine endopeptidase activity (IEA) ? proteasome core complex (sensu Eukaryota) ? ubiquitin-dependent protein catabolism ?  YGL011C ? endopeptidase activity ? mitochondrion ? ubiquitin-dependent protein catabolism ? proteasome core complex, alpha-subunit complex (sensu Eukaryota) ?  1e-34  BLAST | | | | | | | | | | | | | | | | | | | | | | | |

## Cluster Pair #7: 5 gene pairs.

|  |  |  |  |  |  |  |  |  |  |  |  |  |  |  |  |  |  |
| --- | --- | --- | --- | --- | --- | --- | --- | --- | --- | --- | --- | --- | --- | --- | --- | --- | --- |
| P.falciparum S.cerevisiae Blast evalue|  |  |  |  |  |  |  |  |  |  |  |  |  |  |  | | --- | --- | --- | --- | --- | --- | --- | --- | --- | --- | --- | --- | --- | --- | --- | | PF13\_0182 ? hypothetical protein  catalytic activity (IEA) ? ubiquitin activating enzyme activity (IEA) ? protein modification (IEA) ? ubiquitin cycle (IEA) ? small protein activating enzyme activity (IEA) ? membrane ? apicoplast ?  YKL210W ? ubiquitin activating enzyme activity ? nucleus ? cytoplasm ? ubiquitin cycle ?  9e-20  BLAST| PFA0400c ? beta3 proteasome subunit, putative  endopeptidase activity ? threonine endopeptidase activity (IEA) ? proteasome core complex (sensu Eukaryota) ? ubiquitin-dependent protein catabolism ?  YER094C ? endopeptidase activity ? ubiquitin-dependent protein catabolism ? proteasome core complex, beta-subunit complex (sensu Eukaryota) ?  0  BLAST| PF07\_0112 ? proteasome subunit alpha type 5, putative  endopeptidase activity ? threonine endopeptidase activity (IEA) ? proteasome core complex (sensu Eukaryota) (IEA) ? ubiquitin-dependent protein catabolism ? proteasome core complex, alpha-subunit complex (sensu Eukaryota) ?  YGR253C ? endopeptidase activity ? ubiquitin-dependent protein catabolism ? response to stress ? proteasome core complex, alpha-subunit complex (sensu Eukaryota) ? sporulation (sensu Fungi) ?  0  BLAST| PFE0915c ? proteasome subunit beta type 1  endopeptidase activity ? threonine endopeptidase activity (IEA) ? proteasome core complex (sensu Eukaryota) ? ubiquitin-dependent protein catabolism ?  YBL041W ? endopeptidase activity ? ubiquitin-dependent protein catabolism ? proteasome core complex, beta-subunit complex (sensu Eukaryota) ?  9e-35  BLAST| PF13\_0282 ? proteasome subunit, putative  endopeptidase activity ? threonine endopeptidase activity (IEA) ? proteasome core complex (sensu Eukaryota) ? ubiquitin-dependent protein catabolism ?  YGR135W ? endopeptidase activity ? ubiquitin-dependent protein catabolism ? proteasome core complex, alpha-subunit complex (sensu Eukaryota) ? filamentous growth ?  0  BLAST | | | | | | | | | | | | | | | | | |

## Cluster Pair #8: 6 gene pairs.

|  |  |  |  |  |  |  |  |  |  |  |  |  |  |  |  |  |  |  |  |  |
| --- | --- | --- | --- | --- | --- | --- | --- | --- | --- | --- | --- | --- | --- | --- | --- | --- | --- | --- | --- | --- |
| P.falciparum S.cerevisiae Blast evalue|  |  |  |  |  |  |  |  |  |  |  |  |  |  |  |  |  |  | | --- | --- | --- | --- | --- | --- | --- | --- | --- | --- | --- | --- | --- | --- | --- | --- | --- | --- | | PFI0155c ? ras family GTP-ase, putative  GTP binding (IEA) ? intracellular protein transport (IEA) ? small GTPase mediated signal transduction (IEA) ? protein transport (IEA) ?  YML001W ? vacuole inheritance ? telomere maintenance ? GTPase activity ? mitochondrial outer membrane ? vacuole ? Golgi to vacuole transport ? endocytosis ? vesicle-mediated transport ? vacuole fusion, non-autophagic ?  0  BLAST| PF10\_0174 ? 26s proteasome subunit p55, putative  endopeptidase activity ? proteasome regulatory particle (sensu Eukaryota) ? ubiquitin-dependent protein catabolism ?  YDL147W ? ubiquitin-dependent protein catabolism ? proteasome regulatory particle, lid subcomplex (sensu Eukaryota) ?  0  BLAST| PF14\_0493 ? sortilin, putative  lipid transporter activity ? intracellular protein transport (IEA) ? endocytosis ? protein transporter activity (IEA) ? membrane ?  YNR065C ?  0  BLAST| PFC0745c ? proteasome component C8, putative  endopeptidase activity ? threonine endopeptidase activity (IEA) ? proteasome core complex (sensu Eukaryota) ? ubiquitin-dependent protein catabolism ?  YOR362C ? endopeptidase activity ? ubiquitin-dependent protein catabolism ? proteasome core complex, alpha-subunit complex (sensu Eukaryota) ?  1e-32  BLAST| PF11\_0184 ? DNA mismatch repair protein MLH1, putative  ATP binding ? mismatch repair ? ATPase activity ?  YMR167W ? meiotic mismatch repair ? DNA binding ? ATP binding ? nucleus ? mitochondrion ? meiotic recombination ? ATPase activity ?  0  BLAST| PFC0845c ? ubiquitin--protein ligase, putative  ubiquitin ligase complex ? ubiquitin-protein ligase activity ? ubiquitin-dependent protein catabolism ? zinc ion binding (IEA) ? protein ubiquitination (IEA) ? apicoplast ?  YOL133W ? G1/S transition of mitotic cell cycle ? G2/M transition of mitotic cell cycle ? nuclear ubiquitin ligase complex ? ubiquitin-protein ligase activity ? protein binding ? nucleus ? cytoplasm ? protein ubiquitination ? SCF ubiquitin ligase complex ? SCF-dependent proteasomal ubiquitin-dependent protein catabolism ?  9e-34  BLAST | | | | | | | | | | | | | | | | | | | | |

## Cluster Pair #9: 4 gene pairs.

|  |  |  |  |  |  |  |  |  |  |  |  |  |  |  |
| --- | --- | --- | --- | --- | --- | --- | --- | --- | --- | --- | --- | --- | --- | --- |
| P.falciparum S.cerevisiae Blast evalue|  |  |  |  |  |  |  |  |  |  |  |  | | --- | --- | --- | --- | --- | --- | --- | --- | --- | --- | --- | --- | | PF10\_0081 ? 26S proteasome regulatory subunit 4, putative  nucleotide binding (IEA) ? endopeptidase activity ? ATP binding (IEA) ? nucleus (IEA) ? cytoplasm (IEA) ? proteasome regulatory particle (sensu Eukaryota) ? proteolysis and peptidolysis ? hydrolase activity (IEA) ? ATPase activity (IEA) ? nucleoside-triphosphatase activity (IEA) ? protein catabolism (IEA) ?  YDL007W ? endopeptidase activity ? nucleus ? ubiquitin-dependent protein catabolism ? proteasome regulatory particle, base subcomplex (sensu Eukaryota) ? ATPase activity ?  0  BLAST| PF11\_0314 ? 26S protease subunit regulatory subunit 6a, putative  nucleotide binding (IEA) ? proteasome complex (sensu Eukaryota) ? endopeptidase activity ? ATP binding (IEA) ? nucleus (IEA) ? cytoplasm (IEA) ? proteolysis and peptidolysis ? hydrolase activity (IEA) ? ATPase activity ? nucleoside-triphosphatase activity (IEA) ? protein catabolism (IEA) ?  YOR117W ? endopeptidase activity ? ubiquitin-dependent protein catabolism ? proteasome regulatory particle, base subcomplex (sensu Eukaryota) ? ATPase activity ?  0  BLAST| PF14\_0632 ? 26S proteasome subunit, putative  regulation of progression through cell cycle (IEA) ? endopeptidase activity ? proteasome regulatory particle (sensu Eukaryota) ? ubiquitin-dependent protein catabolism ?  YIL075C ? endopeptidase activity ? nucleus ? ubiquitin-dependent protein catabolism ? proteasome regulatory particle, base subcomplex (sensu Eukaryota) ? protein binding, bridging ?  0  BLAST| MAL13P1.343 ? proteasome regulatory subunit, putative  proteasome regulatory particle (sensu Eukaryota) ? ubiquitin-dependent protein catabolism ?  YFR004W ? endopeptidase activity ? nucleus ? ubiquitin-dependent protein catabolism ? proteasome regulatory particle, lid subcomplex (sensu Eukaryota) ?  0  BLAST | | | | | | | | | | | | | | |

## Cluster Pair #10: 4 gene pairs.

|  |  |  |  |  |  |  |  |  |  |  |  |  |  |  |
| --- | --- | --- | --- | --- | --- | --- | --- | --- | --- | --- | --- | --- | --- | --- |
| P.falciparum S.cerevisiae Blast evalue|  |  |  |  |  |  |  |  |  |  |  |  | | --- | --- | --- | --- | --- | --- | --- | --- | --- | --- | --- | --- | | PFD0665c ? 26s proteasome aaa-ATPase subunit Rpt3, putative  nucleotide binding (IEA) ? ATP binding (IEA) ? nucleus (IEA) ? cytoplasm (IEA) ? proteasome regulatory particle (sensu Eukaryota) ? ubiquitin-dependent protein catabolism ? hydrolase activity (IEA) ? ATPase activity ? nucleoside-triphosphatase activity (IEA) ? protein catabolism (IEA) ?  YDR394W ? endopeptidase activity ? ubiquitin-dependent protein catabolism ? proteasome regulatory particle, base subcomplex (sensu Eukaryota) ? ATPase activity ?  0  BLAST| PF08\_0109 ? hypothetical protein   YHR200W ? proteasome complex (sensu Eukaryota) ? endopeptidase activity ? ubiquitin-dependent protein catabolism ? proteasome regulatory particle, base subcomplex (sensu Eukaryota) ?  2e-29  BLAST| PF13\_0063 ? 26S proteasome regulatory subunit 7, putative  nucleotide binding (IEA) ? endopeptidase activity ? ATP binding (IEA) ? nucleus (IEA) ? cytoplasm (IEA) ? proteasome regulatory particle (sensu Eukaryota) ? ubiquitin-dependent protein catabolism ? hydrolase activity (IEA) ? ATPase activity (IEA) ? nucleoside-triphosphatase activity (IEA) ? protein catabolism (IEA) ?  YKL145W ? endopeptidase activity ? ubiquitin-dependent protein catabolism ? proteasome regulatory particle, base subcomplex (sensu Eukaryota) ? ATPase activity ?  0  BLAST| PFL2345c ? tat-binding protein homolog  nucleotide binding (IEA) ? proteasome complex (sensu Eukaryota) ? transcription cofactor activity ? ATP binding (IEA) ? nucleus (IEA) ? cytoplasm (IEA) ? ubiquitin-dependent protein catabolism ? hydrolase activity (IEA) ? ATPase activity ? nucleoside-triphosphatase activity (IEA) ? nucleotide kinase activity (IEA) ? protein catabolism (IEA) ?  YGL048C ? endopeptidase activity ? nucleus ? ubiquitin-dependent protein catabolism ? proteasome regulatory particle, base subcomplex (sensu Eukaryota) ? ATPase activity ?  0  BLAST | | | | | | | | | | | | | | |

## Cluster Pair #11: 5 gene pairs.

|  |  |  |  |  |  |  |  |  |  |  |  |  |  |  |  |  |  |
| --- | --- | --- | --- | --- | --- | --- | --- | --- | --- | --- | --- | --- | --- | --- | --- | --- | --- |
| P.falciparum S.cerevisiae Blast evalue|  |  |  |  |  |  |  |  |  |  |  |  |  |  |  | | --- | --- | --- | --- | --- | --- | --- | --- | --- | --- | --- | --- | --- | --- | --- | | PF11\_0105 ? hypothetical protein   YBR247C ? nucleus ? nucleolus ? rRNA processing ? 35S primary transcript processing ? snoRNA binding ? nucleolar preribosome, small subunit precursor ? ribosome biogenesis and assembly ?  1e-37  BLAST| PF14\_0136 ? hypothetical protein, conserved   YIL103W ? cytoplasm ? peptidyl-diphthamide biosynthesis from peptidyl-histidine ?  0  BLAST| PF14\_0183 ? RNA helicase, putative  nucleic acid binding (IEA) ? ATP-dependent RNA helicase activity ? helicase activity (IEA) ? ATP binding (IEA) ? ATP-dependent helicase activity (IEA) ?  YJL033W ? ATP-dependent RNA helicase activity ? nucleolus ? 35S primary transcript processing ? ribosome biogenesis and assembly ?  0  BLAST| PFI0815c ? hypothetical protein, conserved  S-adenosylmethionine-dependent methyltransferase activity (IEA) ?  YIL064W ? cytoplasm ? S-adenosylmethionine-dependent methyltransferase activity ? ribosome biogenesis and assembly ?  1e-16  BLAST| PF08\_0041 ? ribosome biogenesis protein nep1 homologue, putative  nucleus (IEA) ? ribosome biogenesis ? ribosome biogenesis and assembly (IEA) ?  YLR186W ? nucleus ? nucleolus ? small nucleolar ribonucleoprotein complex ? cytoplasm ? nuclear microtubule ? 35S primary transcript processing ? ribosome biogenesis and assembly ? ribosomal small subunit biogenesis ?  3e-32  BLAST | | | | | | | | | | | | | | | | | |

## Cluster Pair #12: 4 gene pairs.

|  |  |  |  |  |  |  |  |  |  |  |  |  |  |  |
| --- | --- | --- | --- | --- | --- | --- | --- | --- | --- | --- | --- | --- | --- | --- |
| P.falciparum S.cerevisiae Blast evalue|  |  |  |  |  |  |  |  |  |  |  |  | | --- | --- | --- | --- | --- | --- | --- | --- | --- | --- | --- | --- | | PF11\_0284 ? methyltransferase, putative  methyltransferase activity ? S-adenosylmethionine-dependent methyltransferase activity (IEA) ? membrane ?  YDL201W ? protein binding ? nucleus ? tRNA (guanine-N7-)-methyltransferase activity ? tRNA methylation ? ribosome biogenesis and assembly ?  5.60519e-45  BLAST| PFE1335c ? hypothetical protein   YGR162W ? translation initiation factor activity ? mitochondrion ? ribosome ? translational initiation ? eukaryotic translation initiation factor 4F complex ? ribosome biogenesis and assembly ?  0.004  BLAST| MAL13P1.344 ? RNAse L inhibitor protein, putative  nucleotide binding (IEA) ? iron ion binding (IEA) ? ATP binding (IEA) ? electron transport (IEA) ? electron carrier activity (IEA) ? ATPase activity (IEA) ? nucleoside-triphosphatase activity (IEA) ?  YDR091C ? ribosome-nucleus export ? iron ion binding ? nucleus ? cytoplasm ? cytosolic ribosome (sensu Eukaryota) ? translational initiation ? ATPase activity ? ribosomal large subunit biogenesis ?  0  BLAST| PFL0310c ? eukaryotic translation initiation factor 3 subunit 8, putative  translation initiation factor activity ? eukaryotic translation initiation factor 3 complex ? translational initiation (IEA) ? regulation of translational initiation ?  YMR309C ? translation initiation factor activity ? cytoplasm ? eukaryotic translation initiation factor 3 complex ? translational initiation ? ribosome biogenesis and assembly ?  9.99995e-41  BLAST | | | | | | | | | | | | | | |

## Cluster Pair #13: 4 gene pairs.

|  |  |  |  |  |  |  |  |  |  |  |  |  |  |  |
| --- | --- | --- | --- | --- | --- | --- | --- | --- | --- | --- | --- | --- | --- | --- |
| P.falciparum S.cerevisiae Blast evalue|  |  |  |  |  |  |  |  |  |  |  |  | | --- | --- | --- | --- | --- | --- | --- | --- | --- | --- | --- | --- | | MAL7P1.122 ? conserved GTP-binding protein, putative  GTP binding ? signal transduction ?  YBR025C ? cytoplasm ?  0  BLAST| PFE0545c ? histamine-releasing factor, putative  cytoplasm (IEA) ?  YKL056C ? cytoplasm ? mitochondrion ? cytosol ? ribosome ? protein biosynthesis ? response to oxidative stress ?  9e-23  BLAST| PF13\_0179 ? isoleucine--tRNA ligase, putative  tRNA ligase activity (IEA) ? isoleucine-tRNA ligase activity (IEA) ? ATP binding (IEA) ? tRNA aminoacylation for protein translation (IEA) ? isoleucyl-tRNA aminoacylation (IEA) ?  YBL076C ? isoleucine-tRNA ligase activity ? cytosol ? protein biosynthesis ?  0  BLAST| PF11\_0447 ? translation initiation factor eIF-1A, putative  nucleic acid binding (IEA) ? RNA binding (IEA) ? translation initiation factor activity ? translational initiation ? eukaryotic 43S preinitiation complex ?  YMR260C ? translation initiation factor activity ? ribosome ? translational initiation ?  2.00386e-43  BLAST | | | | | | | | | | | | | | |

## Cluster Pair #14: 7 gene pairs.

|  |  |  |  |  |  |  |  |  |  |  |  |  |  |  |  |  |  |  |  |  |  |  |  |
| --- | --- | --- | --- | --- | --- | --- | --- | --- | --- | --- | --- | --- | --- | --- | --- | --- | --- | --- | --- | --- | --- | --- | --- |
| P.falciparum S.cerevisiae Blast evalue|  |  |  |  |  |  |  |  |  |  |  |  |  |  |  |  |  |  |  |  |  | | --- | --- | --- | --- | --- | --- | --- | --- | --- | --- | --- | --- | --- | --- | --- | --- | --- | --- | --- | --- | --- | | PF14\_0656 ? U2 snRNP auxiliary factor, putative  nuclear mRNA splicing, via spliceosome ? nucleic acid binding (IEA) ? RNA binding ? nucleus (IEA) ? mRNA processing (IEA) ?  YGL044C ? RNA binding ? mRNA cleavage factor complex ? mRNA polyadenylylation ? mRNA cleavage ? protein heterodimerization activity ?  3e-07  BLAST| PFD0835c ? hypothetical protein  apicoplast ?  YOL027C ? mitochondrion ? mitochondrial inner membrane ? potassium ion transport ? mitochondrion organization and biogenesis ? potassium ion homeostasis ? protein insertion into mitochondrial membrane ?  4e-27  BLAST| PFE0595w ? hypothetical protein   YLR200W ? cytoplasm ? protein folding ? tubulin folding ? tubulin binding ? prefoldin complex ?  0.001  BLAST| PF14\_0022 ? exopolyphosphatase, putative  exopolyphosphatase activity ? polyphosphate metabolism ? membrane ?  YHR201C ? exopolyphosphatase activity ? cytoplasm ? mitochondrial matrix ? cytosol ? plasma membrane ? polyphosphate metabolism ?  1e-16  BLAST| PF07\_0115 ? integral membrane protein, putative  metalloendopeptidase activity (IEA) ? ATP binding (IEA) ? proteolysis and peptidolysis (IEA) ? cation transport (IEA) ? pathogenesis (IEA) ? ATPase activity, coupled to transmembrane movement of ions, phosphorylative mechanism (IEA) ? membrane (IEA) ? integral to membrane ? apicoplast ? metal ion binding (IEA) ?  YEL031W ? mitochondrion ? endoplasmic reticulum membrane ? protein amino acid glycosylation ? calcium ion homeostasis ? ATPase activity, coupled to transmembrane movement of ions, phosphorylative mechanism ?  5e-29  BLAST| PFA0500w ? human hepatopoietin-like protein, putative  cell proliferation ? \*\* also with: YGR029W, clust.pair #14 YPR037C ? microsome ? protein thiol-disulfide exchange ? thiol oxidase activity ?  8e-15  BLAST| PFA0500w ? human hepatopoietin-like protein, putative  cell proliferation ? \*\* also with: YPR037C, clust.pair #14 YGR029W ? mitochondrion ? mitochondrial intermembrane space ? iron ion homeostasis ? response to oxidative stress ? flavin-linked sulfhydryl oxidase activity ? thiol oxidase activity ? mitochondrial intermembrane space protein import ?  4e-14  BLAST | | | | | | | | | | | | | | | | | | | | | | | |

## Cluster Pair #15: 6 gene pairs.

|  |  |  |  |  |  |  |  |  |  |  |  |  |  |  |  |  |  |  |  |  |
| --- | --- | --- | --- | --- | --- | --- | --- | --- | --- | --- | --- | --- | --- | --- | --- | --- | --- | --- | --- | --- |
| P.falciparum S.cerevisiae Blast evalue|  |  |  |  |  |  |  |  |  |  |  |  |  |  |  |  |  |  | | --- | --- | --- | --- | --- | --- | --- | --- | --- | --- | --- | --- | --- | --- | --- | --- | --- | --- | | PF14\_0378 ? triose-phosphate isomerase  triose-phosphate isomerase activity ? gluconeogenesis ? glycolysis ? pentose-phosphate shunt ? fatty acid biosynthesis ? metabolism (IEA) ?  YDR050C ? triose-phosphate isomerase activity ? cytoplasm ? glycolysis ?  0  BLAST| PFI1105w ? Phosphoglycerate kinase  phosphoglycerate kinase activity (IEA) ? glycolysis (IEA) ?  YCR012W ? phosphoglycerate kinase activity ? cytoplasm ? mitochondrion ? gluconeogenesis ? glycolysis ?  0  BLAST| PF14\_0598 ? glyceraldehyde-3-phosphate dehydrogenase  glyceraldehyde-3-phosphate dehydrogenase (phosphorylating) activity ? mitochondrion ? glucose metabolism (IEA) ? gluconeogenesis ? glycolysis ? glyceraldehyde-3-phosphate dehydrogenase activity (IEA) ? NAD binding (IEA) ? \*\* also with: YGR192C, clust.pair #15 \*\* also with: YJR009C, clust.pair #15 YJL052W ? glyceraldehyde-3-phosphate dehydrogenase (phosphorylating) activity ? cytoplasm ? lipid particle ? gluconeogenesis ? glycolysis ? cell wall (sensu Fungi) ?  0  BLAST| PF14\_0598 ? glyceraldehyde-3-phosphate dehydrogenase  glyceraldehyde-3-phosphate dehydrogenase (phosphorylating) activity ? mitochondrion ? glucose metabolism (IEA) ? gluconeogenesis ? glycolysis ? glyceraldehyde-3-phosphate dehydrogenase activity (IEA) ? NAD binding (IEA) ? \*\* also with: YJL052W, clust.pair #15 \*\* also with: YJR009C, clust.pair #15 YGR192C ? glyceraldehyde-3-phosphate dehydrogenase (phosphorylating) activity ? cytoplasm ? mitochondrion ? lipid particle ? gluconeogenesis ? glycolysis ? cell wall (sensu Fungi) ?  0  BLAST| PF14\_0598 ? glyceraldehyde-3-phosphate dehydrogenase  glyceraldehyde-3-phosphate dehydrogenase (phosphorylating) activity ? mitochondrion ? glucose metabolism (IEA) ? gluconeogenesis ? glycolysis ? glyceraldehyde-3-phosphate dehydrogenase activity (IEA) ? NAD binding (IEA) ? \*\* also with: YJL052W, clust.pair #15 \*\* also with: YGR192C, clust.pair #15 YJR009C ? glyceraldehyde-3-phosphate dehydrogenase (phosphorylating) activity ? cytoplasm ? lipid particle ? gluconeogenesis ? glycolysis ? cell wall (sensu Fungi) ?  0  BLAST| PFL0210c ? eukaryotic initiation factor 5a, putative  nucleic acid binding (IEA) ? translation initiation factor activity ? translational initiation ?  YEL034W ? translation initiation factor activity ? protein binding ? cytoplasm ? mitochondrion ? ribosome ? translational initiation ?  0  BLAST | | | | | | | | | | | | | | | | | | | | |

## Cluster Pair #16: 14 gene pairs.

|  |  |  |  |  |  |  |  |  |  |  |  |  |  |  |  |  |  |  |  |  |  |  |  |  |  |  |  |  |  |  |  |  |  |  |  |  |  |  |  |  |  |  |  |  |
| --- | --- | --- | --- | --- | --- | --- | --- | --- | --- | --- | --- | --- | --- | --- | --- | --- | --- | --- | --- | --- | --- | --- | --- | --- | --- | --- | --- | --- | --- | --- | --- | --- | --- | --- | --- | --- | --- | --- | --- | --- | --- | --- | --- | --- |
| P.falciparum S.cerevisiae Blast evalue|  |  |  |  |  |  |  |  |  |  |  |  |  |  |  |  |  |  |  |  |  |  |  |  |  |  |  |  |  |  |  |  |  |  |  |  |  |  |  |  |  |  | | --- | --- | --- | --- | --- | --- | --- | --- | --- | --- | --- | --- | --- | --- | --- | --- | --- | --- | --- | --- | --- | --- | --- | --- | --- | --- | --- | --- | --- | --- | --- | --- | --- | --- | --- | --- | --- | --- | --- | --- | --- | --- | | PF13\_0224 ? 60S ribosomal subunit protein L18, putative  structural constituent of ribosome ? intracellular (IEA) ? ribosome (IEA) ? cytosolic large ribosomal subunit (sensu Eukaryota) ? protein biosynthesis ? \*\* also with: YMR242C, clust.pair #20 YOR312C ? structural constituent of ribosome ? cytosolic large ribosomal subunit (sensu Eukaryota) ? protein biosynthesis ? ribosome biogenesis and assembly ?  4e-34  BLAST| PFD1055w ? ribosomal protein S19s, putative  structural constituent of ribosome ? intracellular (IEA) ? ribosome ? protein biosynthesis ? \*\* also with: YOL121C, clust.pair #16 YNL302C ? ribosomal small subunit-nucleus export ? structural constituent of ribosome ? cytosolic small ribosomal subunit (sensu Eukaryota) ? rRNA processing ? protein biosynthesis ? ribosomal small subunit biogenesis ?  3e-20  BLAST| PFD1055w ? ribosomal protein S19s, putative  structural constituent of ribosome ? intracellular (IEA) ? ribosome ? protein biosynthesis ? \*\* also with: YNL302C, clust.pair #16 YOL121C ? ribosomal small subunit-nucleus export ? telomere maintenance ? structural constituent of ribosome ? cytosolic small ribosomal subunit (sensu Eukaryota) ? rRNA processing ? protein biosynthesis ? ribosomal small subunit biogenesis ?  3e-20  BLAST| PFC0300c ? 60S ribosomal protein L7, putative  structural constituent of ribosome ? intracellular (IEA) ? ribosome (IEA) ? cytosolic large ribosomal subunit (sensu Eukaryota) ? protein biosynthesis ? large ribosomal subunit (IEA) ? transcription regulator activity (IEA) ? \*\* also with: YGL076C, clust.pair #19 YPL198W ? structural constituent of ribosome ? cytosolic large ribosomal subunit (sensu Eukaryota) ? protein biosynthesis ?  1.00053e-42  BLAST| PFE0185c ? 60S ribosomal subunit protein L31, putative  structural constituent of ribosome ? intracellular (IEA) ? ribosome (IEA) ? protein biosynthesis ? large ribosomal subunit ? \*\* also with: YLR406C, clust.pair #18 YDL075W ? structural constituent of ribosome ? cytosolic large ribosomal subunit (sensu Eukaryota) ? protein biosynthesis ?  9e-21  BLAST| PF13\_0171 ? 60S ribosomal protein L23, putative  structural constituent of ribosome (IEA) ? intracellular (IEA) ? ribosome (IEA) ? protein biosynthesis (IEA) ? \*\* also with: YER117W, clust.pair #20 YBL087C ? structural constituent of ribosome ? cytosolic large ribosomal subunit (sensu Eukaryota) ? protein biosynthesis ?  0  BLAST| PFL2055w ? 40S ribosomal protein S17, putative  structural constituent of ribosome ? intracellular (IEA) ? mitochondrion ? ribosome (IEA) ? cytosolic small ribosomal subunit (sensu Eukaryota) ? protein biosynthesis ?  YDR447C ? ribosomal small subunit assembly and maintenance ? telomere maintenance ? structural constituent of ribosome ? cytosolic small ribosomal subunit (sensu Eukaryota) ? protein biosynthesis ?  4e-33  BLAST| PF11\_0438 ? Ribosomal protein, putative  structural constituent of ribosome ? intracellular (IEA) ? ribosome (IEA) ? protein biosynthesis ? large ribosomal subunit ? \*\* also with: YPL143W, clust.pair #16 YOR234C ? structural constituent of ribosome ? cytosolic large ribosomal subunit (sensu Eukaryota) ? protein biosynthesis ?  1e-30  BLAST| PF11\_0438 ? Ribosomal protein, putative  structural constituent of ribosome ? intracellular (IEA) ? ribosome (IEA) ? protein biosynthesis ? large ribosomal subunit ? \*\* also with: YOR234C, clust.pair #16 YPL143W ? structural constituent of ribosome ? cytosolic large ribosomal subunit (sensu Eukaryota) ? protein biosynthesis ?  1e-30  BLAST| PF13\_0228 ? 40S ribosomal subunit protein S6, putative  structural constituent of ribosome ? intracellular (IEA) ? ribosome (IEA) ? cytosolic small ribosomal subunit (sensu Eukaryota) ? protein biosynthesis ? \*\* also with: YPL090C, clust.pair #18 YBR181C ? structural constituent of ribosome ? small nucleolar ribonucleoprotein complex ? cytoplasm ? cytosolic small ribosomal subunit (sensu Eukaryota) ? protein biosynthesis ?  0  BLAST| PF13\_0268 ? ribosomal protein L17, putative  structural constituent of ribosome ? intracellular (IEA) ? ribosome (IEA) ? cytosolic large ribosomal subunit (sensu Eukaryota) ? protein biosynthesis ? large ribosomal subunit (IEA) ? \*\* also with: YJL177W, clust.pair #19 YKL180W ? structural constituent of ribosome ? cytoplasm ? cytosolic large ribosomal subunit (sensu Eukaryota) ? protein biosynthesis ?  4.00001e-40  BLAST| PFC0735w ? 40S ribosomal protein S15A, putative  structural constituent of ribosome ? intracellular (IEA) ? ribosome (IEA) ? cytosolic small ribosomal subunit (sensu Eukaryota) ? protein biosynthesis ? \*\* also with: YJL190C, clust.pair #18 YLR367W ? structural constituent of ribosome ? cytosolic small ribosomal subunit (sensu Eukaryota) ? protein biosynthesis ?  0  BLAST| PFC0775w ? 40S ribosomal protein S11, putative  nucleic acid binding (IEA) ? structural constituent of ribosome ? intracellular (IEA) ? ribosome (IEA) ? cytosolic small ribosomal subunit (sensu Eukaryota) ? protein biosynthesis ? \*\* also with: YDR025W, clust.pair #16 YBR048W ? ribosomal small subunit assembly and maintenance ? telomere maintenance ? structural constituent of ribosome ? cytosolic small ribosomal subunit (sensu Eukaryota) ? protein biosynthesis ? regulation of translational fidelity ?  0  BLAST| PFC0775w ? 40S ribosomal protein S11, putative  nucleic acid binding (IEA) ? structural constituent of ribosome ? intracellular (IEA) ? ribosome (IEA) ? cytosolic small ribosomal subunit (sensu Eukaryota) ? protein biosynthesis ? \*\* also with: YBR048W, clust.pair #16 YDR025W ? ribosomal small subunit assembly and maintenance ? structural constituent of ribosome ? cytosolic small ribosomal subunit (sensu Eukaryota) ? protein biosynthesis ? regulation of translational fidelity ?  0  BLAST | | | | | | | | | | | | | | | | | | | | | | | | | | | | | | | | | | | | | | | | | | | | |

## Cluster Pair #17: 9 gene pairs.

|  |  |  |  |  |  |  |  |  |  |  |  |  |  |  |  |  |  |  |  |  |  |  |  |  |  |  |  |  |  |
| --- | --- | --- | --- | --- | --- | --- | --- | --- | --- | --- | --- | --- | --- | --- | --- | --- | --- | --- | --- | --- | --- | --- | --- | --- | --- | --- | --- | --- | --- |
| P.falciparum S.cerevisiae Blast evalue|  |  |  |  |  |  |  |  |  |  |  |  |  |  |  |  |  |  |  |  |  |  |  |  |  |  |  | | --- | --- | --- | --- | --- | --- | --- | --- | --- | --- | --- | --- | --- | --- | --- | --- | --- | --- | --- | --- | --- | --- | --- | --- | --- | --- | --- | | PF14\_0027 ? ribosomal S27a, putative  structural constituent of ribosome ? intracellular (IEA) ? ribosome (IEA) ? cytosolic small ribosomal subunit (sensu Eukaryota) ? protein biosynthesis (IEA) ? protein modification (IEA) ?  YLR167W ? ribosomal small subunit assembly and maintenance ? structural constituent of ribosome ? cytoplasm ? cytosolic small ribosomal subunit (sensu Eukaryota) ? protein biosynthesis ? protein ubiquitination ? protein tag ? ribosome biogenesis and assembly ?  1e-12  BLAST| PF13\_0132 ? 60S ribosomal protein L23a, putative  structural constituent of ribosome ? cytosolic large ribosomal subunit (sensu Eukaryota) ? protein biosynthesis ? rRNA binding ?  YOL127W ? ribosomal large subunit assembly and maintenance ? RNA binding ? structural constituent of ribosome ? cytosolic large ribosomal subunit (sensu Eukaryota) ? protein biosynthesis ?  2e-21  BLAST| PFB0830w ? Ribosomal protein S26e, putative  structural constituent of ribosome ? intracellular (IEA) ? mitochondrion ? ribosome (IEA) ? cytosolic small ribosomal subunit (sensu Eukaryota) ? protein biosynthesis ? \*\* also with: YGL189C, clust.pair #17 YER131W ? structural constituent of ribosome ? cytosolic small ribosomal subunit (sensu Eukaryota) ? protein biosynthesis ?  2e-26  BLAST| PFB0830w ? Ribosomal protein S26e, putative  structural constituent of ribosome ? intracellular (IEA) ? mitochondrion ? ribosome (IEA) ? cytosolic small ribosomal subunit (sensu Eukaryota) ? protein biosynthesis ? \*\* also with: YER131W, clust.pair #17 YGL189C ? structural constituent of ribosome ? cytosolic small ribosomal subunit (sensu Eukaryota) ? protein biosynthesis ?  2e-26  BLAST| PF07\_0080 ? 40S ribosomal protein S10, putative  structural constituent of ribosome ? cytosolic small ribosomal subunit (sensu Eukaryota) ? protein biosynthesis ?  YOR293W ? structural constituent of ribosome ? cytosolic small ribosomal subunit (sensu Eukaryota) ? protein biosynthesis ?  2e-20  BLAST| PF14\_0579 ? ribosomal protein L27, putative  structural constituent of ribosome ? intracellular (IEA) ? ribosome (IEA) ? cytosolic large ribosomal subunit (sensu Eukaryota) ? protein biosynthesis ? \*\* also with: YDR471W, clust.pair #20 YHR010W ? structural constituent of ribosome ? cytosolic large ribosomal subunit (sensu Eukaryota) ? protein biosynthesis ?  1e-16  BLAST| MAL13P1.92 ? 40S ribosomal protein S15, putative  structural constituent of ribosome ? intracellular (IEA) ? ribosome (IEA) ? cytosolic small ribosomal subunit (sensu Eukaryota) ? protein biosynthesis ? small ribosomal subunit (IEA) ?  YOL040C ? ribosomal small subunit-nucleus export ? structural constituent of ribosome ? cytosolic small ribosomal subunit (sensu Eukaryota) ? protein biosynthesis ?  5e-36  BLAST| PFC0535w ? 60S ribosomal protein L26, putative  structural constituent of ribosome ? intracellular (IEA) ? ribosome (IEA) ? cytosolic large ribosomal subunit (sensu Eukaryota) ? protein biosynthesis ? large ribosomal subunit (IEA) ? \*\* also with: YGR034W, clust.pair #19 YLR344W ? RNA binding ? structural constituent of ribosome ? cytosolic large ribosomal subunit (sensu Eukaryota) ? protein biosynthesis ?  1e-28  BLAST| PFC1020c ? 40S ribosomal protein S3A, putative  structural constituent of ribosome ? intracellular (IEA) ? ribosome (IEA) ? cytosolic small ribosomal subunit (sensu Eukaryota) ? protein biosynthesis ? \*\* also with: YML063W, clust.pair #19 YLR441C ? structural constituent of ribosome ? cytosolic small ribosomal subunit (sensu Eukaryota) ? protein biosynthesis ?  0  BLAST | | | | | | | | | | | | | | | | | | | | | | | | | | | | | |

## Cluster Pair #18: 8 gene pairs.

|  |  |  |  |  |  |  |  |  |  |  |  |  |  |  |  |  |  |  |  |  |  |  |  |  |  |  |
| --- | --- | --- | --- | --- | --- | --- | --- | --- | --- | --- | --- | --- | --- | --- | --- | --- | --- | --- | --- | --- | --- | --- | --- | --- | --- | --- |
| P.falciparum S.cerevisiae Blast evalue|  |  |  |  |  |  |  |  |  |  |  |  |  |  |  |  |  |  |  |  |  |  |  |  | | --- | --- | --- | --- | --- | --- | --- | --- | --- | --- | --- | --- | --- | --- | --- | --- | --- | --- | --- | --- | --- | --- | --- | --- | | PF13\_0014 ? 40S ribosomal protein S7 homologue, putative  structural constituent of ribosome ? intracellular (IEA) ? ribosome (IEA) ? cytosolic small ribosomal subunit (sensu Eukaryota) ? protein biosynthesis ? \*\* also with: YNL096C, clust.pair #20 YOR096W ? structural constituent of ribosome ? small nucleolar ribonucleoprotein complex ? cytosolic small ribosomal subunit (sensu Eukaryota) ? protein biosynthesis ?  6e-29  BLAST| PFE0810c ? 40S ribosomal subunit protein S14, putative  RNA binding ? structural constituent of ribosome (IEA) ? intracellular (IEA) ? ribosome (IEA) ? cytosolic small ribosomal subunit (sensu Eukaryota) ? protein biosynthesis ? \*\* also with: YJL191W, clust.pair #20 YCR031C ? ribosomal small subunit assembly and maintenance ? telomere maintenance ? RNA binding ? structural constituent of ribosome ? small nucleolar ribonucleoprotein complex ? cytosolic small ribosomal subunit (sensu Eukaryota) ? protein biosynthesis ? processing of 20S pre-rRNA ?  5.60519e-45  BLAST| PFE0185c ? 60S ribosomal subunit protein L31, putative  structural constituent of ribosome ? intracellular (IEA) ? ribosome (IEA) ? protein biosynthesis ? large ribosomal subunit ? \*\* also with: YDL075W, clust.pair #16 YLR406C ? structural constituent of ribosome ? cytosolic large ribosomal subunit (sensu Eukaryota) ? protein biosynthesis ?  5e-21  BLAST| PF11\_0272 ? ribosomal protein S18, putative  RNA binding (IEA) ? structural constituent of ribosome ? intracellular (IEA) ? ribosome (IEA) ? protein biosynthesis ? small ribosomal subunit ?  YDR450W ? telomere maintenance ? structural constituent of ribosome ? mitochondrion ? cytosolic small ribosomal subunit (sensu Eukaryota) ? protein biosynthesis ?  0  BLAST| PF08\_0076 ? 40S ribosomal protein S16, putative  structural constituent of ribosome ? intracellular (IEA) ? ribosome (IEA) ? cytosolic small ribosomal subunit (sensu Eukaryota) ? protein biosynthesis ?  YDL083C ? telomere maintenance ? structural constituent of ribosome ? cytosolic small ribosomal subunit (sensu Eukaryota) ? protein biosynthesis ?  9.80909e-45  BLAST| PF14\_0240 ? ribosomal protein L21e, putative  structural constituent of ribosome ? mitochondrion ? cytosolic large ribosomal subunit (sensu Eukaryota) ? protein biosynthesis ? \*\* also with: YPL079W, clust.pair #19 YBR191W ? structural constituent of ribosome ? cytosolic large ribosomal subunit (sensu Eukaryota) ? protein biosynthesis ?  4e-28  BLAST| PF13\_0228 ? 40S ribosomal subunit protein S6, putative  structural constituent of ribosome ? intracellular (IEA) ? ribosome (IEA) ? cytosolic small ribosomal subunit (sensu Eukaryota) ? protein biosynthesis ? \*\* also with: YBR181C, clust.pair #16 YPL090C ? structural constituent of ribosome ? small nucleolar ribonucleoprotein complex ? cytosolic small ribosomal subunit (sensu Eukaryota) ? protein biosynthesis ?  0  BLAST| PFC0735w ? 40S ribosomal protein S15A, putative  structural constituent of ribosome ? intracellular (IEA) ? ribosome (IEA) ? cytosolic small ribosomal subunit (sensu Eukaryota) ? protein biosynthesis ? \*\* also with: YLR367W, clust.pair #16 YJL190C ? telomere maintenance ? structural constituent of ribosome ? cytosolic small ribosomal subunit (sensu Eukaryota) ? protein biosynthesis ?  0  BLAST | | | | | | | | | | | | | | | | | | | | | | | | | | |

## Cluster Pair #19: 9 gene pairs.

|  |  |  |  |  |  |  |  |  |  |  |  |  |  |  |  |  |  |  |  |  |  |  |  |  |  |  |  |  |  |
| --- | --- | --- | --- | --- | --- | --- | --- | --- | --- | --- | --- | --- | --- | --- | --- | --- | --- | --- | --- | --- | --- | --- | --- | --- | --- | --- | --- | --- | --- |
| P.falciparum S.cerevisiae Blast evalue|  |  |  |  |  |  |  |  |  |  |  |  |  |  |  |  |  |  |  |  |  |  |  |  |  |  |  | | --- | --- | --- | --- | --- | --- | --- | --- | --- | --- | --- | --- | --- | --- | --- | --- | --- | --- | --- | --- | --- | --- | --- | --- | --- | --- | --- | | PF10\_0038 ? ribosomal protein S20e, putative  structural constituent of ribosome ? intracellular (IEA) ? ribosome (IEA) ? cytosolic small ribosomal subunit (sensu Eukaryota) ? protein biosynthesis ? small ribosomal subunit (IEA) ?  YHL015W ? structural constituent of ribosome ? cytosolic small ribosomal subunit (sensu Eukaryota) ? protein biosynthesis ?  3e-25  BLAST| PF13\_0213 ? 60S ribosomal subunit protein L6e, putative  structural constituent of ribosome ? intracellular (IEA) ? ribosome (IEA) ? cytosolic large ribosomal subunit (sensu Eukaryota) ? protein biosynthesis ?  YLR448W ? ribosomal large subunit assembly and maintenance ? RNA binding ? structural constituent of ribosome ? cytosolic large ribosomal subunit (sensu Eukaryota) ? protein biosynthesis ?  7e-16  BLAST| PF10\_0187 ? ribosomal protein L30e, putative  structural constituent of ribosome ? cytosolic large ribosomal subunit (sensu Eukaryota) ? protein biosynthesis ?  YGL030W ? structural constituent of ribosome ? cytoplasm ? cytosolic large ribosomal subunit (sensu Eukaryota) ? rRNA processing ? protein biosynthesis ? negative regulation of protein biosynthesis ? negative regulation of nuclear mRNA splicing, via spliceosome ?  2e-26  BLAST| PFC0300c ? 60S ribosomal protein L7, putative  structural constituent of ribosome ? intracellular (IEA) ? ribosome (IEA) ? cytosolic large ribosomal subunit (sensu Eukaryota) ? protein biosynthesis ? large ribosomal subunit (IEA) ? transcription regulator activity (IEA) ? \*\* also with: YPL198W, clust.pair #16 YGL076C ? structural constituent of ribosome ? cytosolic large ribosomal subunit (sensu Eukaryota) ? protein biosynthesis ?  1.00053e-42  BLAST| PF13\_0045 ? 40S ribosomal protein S27, putative  structural constituent of ribosome ? intracellular (IEA) ? ribosome (IEA) ? cytosolic small ribosomal subunit (sensu Eukaryota) ? protein biosynthesis ? \*\* also with: YHR021C, clust.pair #20 YKL156W ? structural constituent of ribosome ? cytoplasm ? cytosolic small ribosomal subunit (sensu Eukaryota) ? protein biosynthesis ?  5e-27  BLAST| PF14\_0240 ? ribosomal protein L21e, putative  structural constituent of ribosome ? mitochondrion ? cytosolic large ribosomal subunit (sensu Eukaryota) ? protein biosynthesis ? \*\* also with: YBR191W, clust.pair #18 YPL079W ? structural constituent of ribosome ? cytosolic large ribosomal subunit (sensu Eukaryota) ? protein biosynthesis ?  5e-28  BLAST| PFC0535w ? 60S ribosomal protein L26, putative  structural constituent of ribosome ? intracellular (IEA) ? ribosome (IEA) ? cytosolic large ribosomal subunit (sensu Eukaryota) ? protein biosynthesis ? large ribosomal subunit (IEA) ? \*\* also with: YLR344W, clust.pair #17 YGR034W ? RNA binding ? structural constituent of ribosome ? cytosolic large ribosomal subunit (sensu Eukaryota) ? protein biosynthesis ?  2e-28  BLAST| PF13\_0268 ? ribosomal protein L17, putative  structural constituent of ribosome ? intracellular (IEA) ? ribosome (IEA) ? cytosolic large ribosomal subunit (sensu Eukaryota) ? protein biosynthesis ? large ribosomal subunit (IEA) ? \*\* also with: YKL180W, clust.pair #16 YJL177W ? structural constituent of ribosome ? cytosolic large ribosomal subunit (sensu Eukaryota) ? protein biosynthesis ?  3e-40  BLAST| PFC1020c ? 40S ribosomal protein S3A, putative  structural constituent of ribosome ? intracellular (IEA) ? ribosome (IEA) ? cytosolic small ribosomal subunit (sensu Eukaryota) ? protein biosynthesis ? \*\* also with: YLR441C, clust.pair #17 YML063W ? structural constituent of ribosome ? cytosolic small ribosomal subunit (sensu Eukaryota) ? protein biosynthesis ?  0  BLAST | | | | | | | | | | | | | | | | | | | | | | | | | | | | | |

## Cluster Pair #20: 13 gene pairs.

|  |  |  |  |  |  |  |  |  |  |  |  |  |  |  |  |  |  |  |  |  |  |  |  |  |  |  |  |  |  |  |  |  |  |  |  |  |  |  |  |  |  |
| --- | --- | --- | --- | --- | --- | --- | --- | --- | --- | --- | --- | --- | --- | --- | --- | --- | --- | --- | --- | --- | --- | --- | --- | --- | --- | --- | --- | --- | --- | --- | --- | --- | --- | --- | --- | --- | --- | --- | --- | --- | --- |
| P.falciparum S.cerevisiae Blast evalue|  |  |  |  |  |  |  |  |  |  |  |  |  |  |  |  |  |  |  |  |  |  |  |  |  |  |  |  |  |  |  |  |  |  |  |  |  |  |  | | --- | --- | --- | --- | --- | --- | --- | --- | --- | --- | --- | --- | --- | --- | --- | --- | --- | --- | --- | --- | --- | --- | --- | --- | --- | --- | --- | --- | --- | --- | --- | --- | --- | --- | --- | --- | --- | --- | --- | | PF13\_0014 ? 40S ribosomal protein S7 homologue, putative  structural constituent of ribosome ? intracellular (IEA) ? ribosome (IEA) ? cytosolic small ribosomal subunit (sensu Eukaryota) ? protein biosynthesis ? \*\* also with: YOR096W, clust.pair #18 YNL096C ? structural constituent of ribosome ? small nucleolar ribonucleoprotein complex ? cytosolic small ribosomal subunit (sensu Eukaryota) ? protein biosynthesis ?  5e-31  BLAST| PF13\_0224 ? 60S ribosomal subunit protein L18, putative  structural constituent of ribosome ? intracellular (IEA) ? ribosome (IEA) ? cytosolic large ribosomal subunit (sensu Eukaryota) ? protein biosynthesis ? \*\* also with: YOR312C, clust.pair #16 YMR242C ? structural constituent of ribosome ? cytosolic large ribosomal subunit (sensu Eukaryota) ? protein biosynthesis ?  4e-34  BLAST| PFE0810c ? 40S ribosomal subunit protein S14, putative  RNA binding ? structural constituent of ribosome (IEA) ? intracellular (IEA) ? ribosome (IEA) ? cytosolic small ribosomal subunit (sensu Eukaryota) ? protein biosynthesis ? \*\* also with: YCR031C, clust.pair #18 YJL191W ? ribosomal small subunit assembly and maintenance ? RNA binding ? structural constituent of ribosome ? small nucleolar ribonucleoprotein complex ? cytosolic small ribosomal subunit (sensu Eukaryota) ? protein biosynthesis ? processing of 20S pre-rRNA ?  5.60519e-45  BLAST| PFI0190w ? ribosomal protein L32, putative  structural constituent of ribosome (IEA) ? intracellular (IEA) ? ribosome (IEA) ? protein biosynthesis (IEA) ?  YBL092W ? structural constituent of ribosome ? cytosolic large ribosomal subunit (sensu Eukaryota) ? protein biosynthesis ?  6e-22  BLAST| PFB0455w ? ribosomal L37ae protein, putative  structural constituent of ribosome ? intracellular (IEA) ? mitochondrion ? ribosome (IEA) ? cytosolic large ribosomal subunit (sensu Eukaryota) ? protein biosynthesis ?  YPR043W ? structural constituent of ribosome ? cytosolic large ribosomal subunit (sensu Eukaryota) ? protein biosynthesis ?  9e-26  BLAST| PFC0295c ? 40S ribosomal protein S12, putative  structural constituent of ribosome ? intracellular (IEA) ? ribosome (IEA) ? cytosolic small ribosomal subunit (sensu Eukaryota) ? protein biosynthesis ?  YOR369C ? structural constituent of ribosome ? cytosolic small ribosomal subunit (sensu Eukaryota) ? protein biosynthesis ?  9e-19  BLAST| PF13\_0045 ? 40S ribosomal protein S27, putative  structural constituent of ribosome ? intracellular (IEA) ? ribosome (IEA) ? cytosolic small ribosomal subunit (sensu Eukaryota) ? protein biosynthesis ? \*\* also with: YKL156W, clust.pair #19 YHR021C ? telomere maintenance ? structural constituent of ribosome ? cytosolic small ribosomal subunit (sensu Eukaryota) ? protein biosynthesis ?  5e-27  BLAST| PF13\_0171 ? 60S ribosomal protein L23, putative  structural constituent of ribosome (IEA) ? intracellular (IEA) ? ribosome (IEA) ? protein biosynthesis (IEA) ? \*\* also with: YBL087C, clust.pair #16 YER117W ? structural constituent of ribosome ? cytosolic large ribosomal subunit (sensu Eukaryota) ? protein biosynthesis ? response to drug ?  0  BLAST| PF14\_0231 ? ribosomal protein L7a, putative  structural constituent of ribosome ? intracellular (IEA) ? ribosome (IEA) ? cytosolic large ribosomal subunit (sensu Eukaryota) ? protein biosynthesis ? ribonucleoprotein complex (IEA) ? ribosome biogenesis and assembly (IEA) ? \*\* also with: YLL045C, clust.pair #20 YHL033C ? structural constituent of ribosome ? cytosolic large ribosomal subunit (sensu Eukaryota) ? protein biosynthesis ?  2.00386e-43  BLAST| PF14\_0231 ? ribosomal protein L7a, putative  structural constituent of ribosome ? intracellular (IEA) ? ribosome (IEA) ? cytosolic large ribosomal subunit (sensu Eukaryota) ? protein biosynthesis ? ribonucleoprotein complex (IEA) ? ribosome biogenesis and assembly (IEA) ? \*\* also with: YHL033C, clust.pair #20 YLL045C ? structural constituent of ribosome ? cytosolic large ribosomal subunit (sensu Eukaryota) ? protein biosynthesis ?  2.99878e-43  BLAST| PF14\_0579 ? ribosomal protein L27, putative  structural constituent of ribosome ? intracellular (IEA) ? ribosome (IEA) ? cytosolic large ribosomal subunit (sensu Eukaryota) ? protein biosynthesis ? \*\* also with: YHR010W, clust.pair #17 YDR471W ? structural constituent of ribosome ? cytosolic large ribosomal subunit (sensu Eukaryota) ? protein biosynthesis ?  5e-16  BLAST| PF14\_0655 ? RNA helicase-1, putative  RNA cap binding ? nucleic acid binding (IEA) ? mRNA binding ? translation initiation factor activity ? helicase activity (IEA) ? ATP binding (IEA) ? regulation of translational initiation ? ATP-dependent helicase activity ? eukaryotic translation initiation factor 4F complex ? \*\* also with: YKR059W, clust.pair #20 YJL138C ? RNA helicase activity ? translation initiation factor activity ? cytoplasm ? ribosome ? translational initiation ? regulation of translational initiation ? eukaryotic translation initiation factor 4F complex ?  0  BLAST| PF14\_0655 ? RNA helicase-1, putative  RNA cap binding ? nucleic acid binding (IEA) ? mRNA binding ? translation initiation factor activity ? helicase activity (IEA) ? ATP binding (IEA) ? regulation of translational initiation ? ATP-dependent helicase activity ? eukaryotic translation initiation factor 4F complex ? \*\* also with: YJL138C, clust.pair #20 YKR059W ? telomere maintenance ? translation initiation factor activity ? ATP-dependent RNA helicase activity ? ribosome ? translational initiation ? eukaryotic translation initiation factor 4F complex ?  0  BLAST | | | | | | | | | | | | | | | | | | | | | | | | | | | | | | | | | | | | | | | | | |

## Cluster Pair #21: 24 gene pairs.

|  |  |  |  |  |  |  |  |  |  |  |  |  |  |  |  |  |  |  |  |  |  |  |  |  |  |  |  |  |  |  |  |  |  |  |  |  |  |  |  |  |  |  |  |  |  |  |  |  |  |  |  |  |  |  |  |  |  |  |  |  |  |  |  |  |  |  |  |  |  |  |  |  |  |  |
| --- | --- | --- | --- | --- | --- | --- | --- | --- | --- | --- | --- | --- | --- | --- | --- | --- | --- | --- | --- | --- | --- | --- | --- | --- | --- | --- | --- | --- | --- | --- | --- | --- | --- | --- | --- | --- | --- | --- | --- | --- | --- | --- | --- | --- | --- | --- | --- | --- | --- | --- | --- | --- | --- | --- | --- | --- | --- | --- | --- | --- | --- | --- | --- | --- | --- | --- | --- | --- | --- | --- | --- | --- | --- | --- |
| P.falciparum S.cerevisiae Blast evalue|  |  |  |  |  |  |  |  |  |  |  |  |  |  |  |  |  |  |  |  |  |  |  |  |  |  |  |  |  |  |  |  |  |  |  |  |  |  |  |  |  |  |  |  |  |  |  |  |  |  |  |  |  |  |  |  |  |  |  |  |  |  |  |  |  |  |  |  |  |  |  |  | | --- | --- | --- | --- | --- | --- | --- | --- | --- | --- | --- | --- | --- | --- | --- | --- | --- | --- | --- | --- | --- | --- | --- | --- | --- | --- | --- | --- | --- | --- | --- | --- | --- | --- | --- | --- | --- | --- | --- | --- | --- | --- | --- | --- | --- | --- | --- | --- | --- | --- | --- | --- | --- | --- | --- | --- | --- | --- | --- | --- | --- | --- | --- | --- | --- | --- | --- | --- | --- | --- | --- | --- | | PFL1820w ? hypothetical protein   YLR196W ? nucleus ? nucleolus ? cytoplasm ? rRNA processing ?  3e-15  BLAST| PFL1470c ? hypothetical protein  \*\* also with: YCR057C, clust.pair #22 YLR222C ? small nucleolar ribonucleoprotein complex ? processing of 20S pre-rRNA ? snoRNA binding ?  0.012  BLAST| PF14\_0185 ? ATP-dependent RNA helicase, putative  nucleic acid binding (IEA) ? ATP-dependent RNA helicase activity ? helicase activity (IEA) ? ATP binding (IEA) ? ATP-dependent helicase activity (IEA) ? \*\* also with: YHR065C, clust.pair #22 \*\* also with: YGL078C, clust.pair #22 \*\* also with: YJL033W, clust.pair #22 \*\* also with: YFL002C, clust.pair #23 YMR290C ? RNA binding ? ATP-dependent RNA helicase activity ? nuclear membrane ? nucleolus ? rRNA processing ? RNA-dependent ATPase activity ? ribosome biogenesis and assembly ?  5e-10  BLAST| PF14\_0584 ? ribosomal protein S4, putative  RNA binding (IEA) ? structural constituent of ribosome ? mitochondrial small ribosomal subunit ? protein biosynthesis ?  YHR148W ? rRNA modification ? small nucleolar ribonucleoprotein complex ? 35S primary transcript processing ? processing of 20S pre-rRNA ? snoRNA binding ? ribosome biogenesis and assembly ?  1e-35  BLAST| PF14\_0274 ? hypothetical protein, conserved   YKL191W ? cytoplasm ? peptidyl-diphthamide biosynthesis from peptidyl-histidine ? ribosome biogenesis and assembly ?  8e-14  BLAST| PF13\_0177 ? ATP-dependent RNA helicase, putative  nucleic acid binding (IEA) ? helicase activity (IEA) ? ATP binding (IEA) ? ATP-dependent helicase activity (IEA) ? \*\* also with: YHR065C, clust.pair #22 YHR169W ? ATP-dependent RNA helicase activity ? nucleolus ? 35S primary transcript processing ? ribosome biogenesis and assembly ?  0  BLAST| PF10\_0278 ? hypothetical protein, conserved   YKR081C ? ribosomal large subunit assembly and maintenance ? nucleolus ? 5S rRNA binding ? 7S RNA binding ? rRNA binding ? processing of 27S pre-rRNA ? ribosome biogenesis and assembly ?  2e-05  BLAST| PF11\_0275 ? hypothetical protein   YLR409C ? nucleus ? nucleolus ? small nucleolar ribonucleoprotein complex ? 35S primary transcript processing ? snoRNA binding ? ribosome biogenesis and assembly ?  0.083  BLAST| PF11\_0274 ? hypothetical protein   YFR001W ? mRNA binding ? nucleus ? intracellular mRNA localization ? ribosomal large subunit biogenesis ?  0.002  BLAST| PF07\_0083 ? hypothetical protein, conserved   YNR054C ? nucleolus ? small nucleolar ribonucleoprotein complex ? cytoplasm ? 35S primary transcript processing ? transcription regulator activity ? ribosome biogenesis and assembly ?  6e-15  BLAST| MAL7P1.24 ? hypothetical protein, conserved  intracellular (IEA) ?  YER126C ? nucleus ? ribosome biogenesis and assembly ? ribosomal large subunit biogenesis ?  0  BLAST| PF13\_0309 ? hypothetical protein   YLR409C ? nucleus ? nucleolus ? small nucleolar ribonucleoprotein complex ? 35S primary transcript processing ? snoRNA binding ? ribosome biogenesis and assembly ?  1e-17  BLAST| PFI0625c ? hypothetical protein  translation initiation factor activity (IEA) ? regulation of translational initiation (IEA) ?  YNL062C ? tRNA binding ? nucleus ? translational initiation ? tRNA (adenine-N1-)-methyltransferase activity ? tRNA methylation ? ribosome biogenesis and assembly ?  5e-07  BLAST| PF07\_0121 ? hypothetical protein, conserved   YHR170W ? ribosomal large subunit assembly and maintenance ? ribosomal large subunit-nucleus export ? RNA binding ? protein binding ? cytosol ? cytosolic large ribosomal subunit (sensu Eukaryota) ? ribosome biogenesis and assembly ?  2.94273e-44  BLAST| PF13\_0184 ? hypothetical protein  metalloendopeptidase activity (IEA) ? cytoplasm (IEA) ? proteolysis and peptidolysis (IEA) ? phosphate transport (IEA) ? pathogenesis (IEA) ? membrane ? metal ion binding (IEA) ?  YDR324C ? small nucleolar ribonucleoprotein complex ? processing of 20S pre-rRNA ? snoRNA binding ? ribosome biogenesis and assembly ?  2e-07  BLAST| PFL1345c ? hypothetical protein, conserved  catalytic activity (IEA) ? iron ion binding (IEA) ? membrane ?  YPL086C ? histone acetyltransferase activity ? nucleus ? cytoplasm ? regulation of transcription from RNA polymerase II promoter ? tRNA modification ? transcription elongation factor complex ?  0  BLAST| PF14\_0635 ? hypothetical protein, conserved  RNA binding (IEA) ?  YPL211W ? ribosomal large subunit assembly and maintenance ? nucleolus ? cytosolic large ribosomal subunit (sensu Eukaryota) ? rRNA processing ? ribosome biogenesis and assembly ? ribosomal large subunit biogenesis ?  2e-35  BLAST| PF10\_0200 ? hypothetical protein, conserved   YNL132W ? nucleolus ? ribosome biogenesis and assembly ?  2e-39  BLAST| MAL7P1.113 ? DEAD box helicase, putative  nucleic acid binding (IEA) ? RNA binding ? ATP-dependent RNA helicase activity ? helicase activity (IEA) ? ATP binding ? ATP-dependent helicase activity (IEA) ? RNA metabolism ? \*\* also with: YMR290C, clust.pair #21 YKR024C ? ribosomal large subunit assembly and maintenance ? ATP-dependent RNA helicase activity ? nucleolus ? 35S primary transcript processing ? ribosome biogenesis and assembly ?  5e-26  BLAST| MAL7P1.113 ? DEAD box helicase, putative  nucleic acid binding (IEA) ? RNA binding ? ATP-dependent RNA helicase activity ? helicase activity (IEA) ? ATP binding ? ATP-dependent helicase activity (IEA) ? RNA metabolism ? \*\* also with: YKR024C, clust.pair #21 YMR290C ? RNA binding ? ATP-dependent RNA helicase activity ? nuclear membrane ? nucleolus ? rRNA processing ? RNA-dependent ATPase activity ? ribosome biogenesis and assembly ?  1e-24  BLAST| PF14\_0494 ? hypothetical protein, conserved  mitochondrion ?  YDL060W ? nucleolus ? cytoplasm ? rRNA processing ? ribosome biogenesis and assembly ? ribonucleoprotein binding ?  2e-18  BLAST| PF13\_0109 ? N2,N2-dimethylguanosine tRNA methyltransferase, putative  RNA binding (IEA) ? tRNA (guanine-N2-)-methyltransferase activity ? tRNA modification ? tRNA processing (IEA) ? apicoplast ?  YDR120C ? tRNA (guanine-N2-)-methyltransferase activity ? nuclear membrane ? nuclear inner membrane ? mitochondrion ? tRNA methylation ? ribosome biogenesis and assembly ?  3e-13  BLAST| PF14\_0221 ? hypothetical protein, conserved  GTP binding (IEA) ?  YNR053C ? ribosomal large subunit-nucleus export ? GTPase activity ? nucleus ? nucleoplasm ? nucleolus ? ribosome biogenesis and assembly ? ribosome assembly ?  0  BLAST| PF08\_0065 ? hypothetical protein, conserved   YMR131C ? nucleolus ? ribosome biogenesis and assembly ?  5e-33  BLAST | | | | | | | | | | | | | | | | | | | | | | | | | | | | | | | | | | | | | | | | | | | | | | | | | | | | | | | | | | | | | | | | | | | | | | | | | | |

## Cluster Pair #22: 28 gene pairs.

|  |  |  |  |  |  |  |  |  |  |  |  |  |  |  |  |  |  |  |  |  |  |  |  |  |  |  |  |  |  |  |  |  |  |  |  |  |  |  |  |  |  |  |  |  |  |  |  |  |  |  |  |  |  |  |  |  |  |  |  |  |  |  |  |  |  |  |  |  |  |  |  |  |  |  |  |  |  |  |  |  |  |  |  |  |  |  |
| --- | --- | --- | --- | --- | --- | --- | --- | --- | --- | --- | --- | --- | --- | --- | --- | --- | --- | --- | --- | --- | --- | --- | --- | --- | --- | --- | --- | --- | --- | --- | --- | --- | --- | --- | --- | --- | --- | --- | --- | --- | --- | --- | --- | --- | --- | --- | --- | --- | --- | --- | --- | --- | --- | --- | --- | --- | --- | --- | --- | --- | --- | --- | --- | --- | --- | --- | --- | --- | --- | --- | --- | --- | --- | --- | --- | --- | --- | --- | --- | --- | --- | --- | --- | --- | --- | --- |
| P.falciparum S.cerevisiae Blast evalue|  |  |  |  |  |  |  |  |  |  |  |  |  |  |  |  |  |  |  |  |  |  |  |  |  |  |  |  |  |  |  |  |  |  |  |  |  |  |  |  |  |  |  |  |  |  |  |  |  |  |  |  |  |  |  |  |  |  |  |  |  |  |  |  |  |  |  |  |  |  |  |  |  |  |  |  |  |  |  |  |  |  |  |  | | --- | --- | --- | --- | --- | --- | --- | --- | --- | --- | --- | --- | --- | --- | --- | --- | --- | --- | --- | --- | --- | --- | --- | --- | --- | --- | --- | --- | --- | --- | --- | --- | --- | --- | --- | --- | --- | --- | --- | --- | --- | --- | --- | --- | --- | --- | --- | --- | --- | --- | --- | --- | --- | --- | --- | --- | --- | --- | --- | --- | --- | --- | --- | --- | --- | --- | --- | --- | --- | --- | --- | --- | --- | --- | --- | --- | --- | --- | --- | --- | --- | --- | --- | --- | | PFD0515w ? exosome complex exonuclease rrp4, putative  3'-5'-exoribonuclease activity ? exosome (RNase complex) ? nucleic acid binding (IEA) ? RNA binding (IEA) ? rRNA processing ?  YHR069C ? 3'-5'-exoribonuclease activity ? nuclear exosome (RNase complex) ? cytoplasmic exosome (RNase complex) ? RNA binding ? 35S primary transcript processing ? mRNA catabolism ?  4e-33  BLAST| PFL2010c ? DEAD%2FDEAH box helicase, putative  nucleic acid binding (IEA) ? ATP-dependent RNA helicase activity ? helicase activity (IEA) ? ATP binding (IEA) ? ATP-dependent helicase activity (IEA) ? RNA metabolism ?  YLR276C ? ribosomal large subunit assembly and maintenance ? ATP-dependent RNA helicase activity ? nucleolus ? 35S primary transcript processing ? ribosome biogenesis and assembly ?  3e-25  BLAST| PFL1470c ? hypothetical protein  \*\* also with: YLR222C, clust.pair #21 YCR057C ? cytokinesis ? small nucleolar ribonucleoprotein complex ? cytoplasm ? 35S primary transcript processing ? establishment of cell polarity (sensu Fungi) ? processing of 20S pre-rRNA ? snoRNA binding ? 90S preribosome ? ribosome biogenesis and assembly ?  0.012  BLAST| PFL1230w ? hypothetical protein   YLR186W ? nucleus ? nucleolus ? small nucleolar ribonucleoprotein complex ? cytoplasm ? nuclear microtubule ? 35S primary transcript processing ? ribosome biogenesis and assembly ? ribosomal small subunit biogenesis ?  3e-12  BLAST| PFL2295w ? hypothetical protein  rRNA processing (IEA) ? ribonucleoprotein complex (IEA) ?  YKL099C ? small nucleolar ribonucleoprotein complex ? processing of 20S pre-rRNA ? snoRNA binding ? ribosome biogenesis and assembly ?  0.095  BLAST| MAL13P1.14 ? ATP-dependent DEAD box helicase, putative  nucleic acid binding (IEA) ? ATP-dependent RNA helicase activity ? helicase activity (IEA) ? ATP binding (IEA) ? ATP-dependent helicase activity (IEA) ?  YMR128W ? RNA helicase activity ? nucleolus ? small nucleolar ribonucleoprotein complex ? mitochondrion ? processing of 20S pre-rRNA ? ribosome biogenesis and assembly ?  9.94922e-44  BLAST| PF14\_0185 ? ATP-dependent RNA helicase, putative  nucleic acid binding (IEA) ? ATP-dependent RNA helicase activity ? helicase activity (IEA) ? ATP binding (IEA) ? ATP-dependent helicase activity (IEA) ? \*\* also with: YMR290C, clust.pair #21 \*\* also with: YGL078C, clust.pair #22 \*\* also with: YJL033W, clust.pair #22 \*\* also with: YFL002C, clust.pair #23 YHR065C ? ATP-dependent RNA helicase activity ? nucleolus ? 35S primary transcript processing ? ribosome biogenesis and assembly ?  1e-09  BLAST| PF14\_0185 ? ATP-dependent RNA helicase, putative  nucleic acid binding (IEA) ? ATP-dependent RNA helicase activity ? helicase activity (IEA) ? ATP binding (IEA) ? ATP-dependent helicase activity (IEA) ? \*\* also with: YMR290C, clust.pair #21 \*\* also with: YHR065C, clust.pair #22 \*\* also with: YJL033W, clust.pair #22 \*\* also with: YFL002C, clust.pair #23 YGL078C ? ribosomal large subunit assembly and maintenance ? ATP-dependent RNA helicase activity ? nucleolus ? 35S primary transcript processing ?  2e-08  BLAST| PF14\_0185 ? ATP-dependent RNA helicase, putative  nucleic acid binding (IEA) ? ATP-dependent RNA helicase activity ? helicase activity (IEA) ? ATP binding (IEA) ? ATP-dependent helicase activity (IEA) ? \*\* also with: YMR290C, clust.pair #21 \*\* also with: YHR065C, clust.pair #22 \*\* also with: YGL078C, clust.pair #22 \*\* also with: YFL002C, clust.pair #23 YJL033W ? ATP-dependent RNA helicase activity ? nucleolus ? 35S primary transcript processing ? ribosome biogenesis and assembly ?  2e-08  BLAST| PFI0860c ? ATP-dependant RNA helicase, putative  nucleic acid binding (IEA) ? helicase activity (IEA) ? ATP binding (IEA) ? ATP-dependent helicase activity (IEA) ?  YGL120C ? U2-type spliceosome disassembly ? ATP-dependent RNA helicase activity ? spliceosome complex ? mitochondrion ? rRNA processing ? 35S primary transcript processing ? processing of 27S pre-rRNA ? processing of 20S pre-rRNA ? RNA splicing factor activity, transesterification mechanism ? ribosome biogenesis and assembly ? ribosomal large subunit biogenesis ?  0  BLAST| PF13\_0261 ? ATP binding protein, putative  ATP binding ?  YOR262W ? cytoplasm ?  4e-22  BLAST| PF14\_0292 ? hypothetical protein, conserved  GTP binding (IEA) ?  YGL099W ? ribosome-nucleus export ? conjugation with cellular fusion ? GTPase activity ? cytoplasm ? sporulation (sensu Fungi) ? ribosome biogenesis and assembly ?  2e-30  BLAST| PF13\_0177 ? ATP-dependent RNA helicase, putative  nucleic acid binding (IEA) ? helicase activity (IEA) ? ATP binding (IEA) ? ATP-dependent helicase activity (IEA) ? \*\* also with: YHR169W, clust.pair #21 YHR065C ? ATP-dependent RNA helicase activity ? nucleolus ? 35S primary transcript processing ? ribosome biogenesis and assembly ?  0  BLAST| PF14\_0456 ? hypothetical protein, conserved   YLR129W ? small nucleolar ribonucleoprotein complex ? processing of 20S pre-rRNA ? snoRNA binding ? ribosome biogenesis and assembly ?  5.04467e-44  BLAST| MAL13P1.341 ? hypothetical protein, conserved   YKL009W ? telomere maintenance ? nucleus ? nucleolus ? rRNA processing ? mRNA catabolism ? ribosome biogenesis and assembly ? ribosomal large subunit biogenesis ?  4e-06  BLAST| PF14\_0157 ? hypothetical protein   YHR040W ? nucleic acid binding ? nucleus ? cytoplasm ? snoRNA metabolism ?  0.006  BLAST| PF10\_0087 ? diphthine synthase  diphthine synthase activity ? metabolism (IEA) ? methyltransferase activity (IEA) ? peptidyl-diphthamide biosynthesis from peptidyl-histidine (IEA) ?  YLR172C ? diphthine synthase activity ? cytoplasm ? peptidyl-diphthamide biosynthesis from peptidyl-histidine ?  0  BLAST| PFI0920c ? hypothetical protein, conserved  tRNA processing (IEA) ? oxidoreductase activity (IEA) ? apicoplast ? FAD binding (IEA) ?  YML080W ? nucleus ? tRNA modification ? tRNA dihydrouridine synthase activity ?  9e-10  BLAST| PFB0860c ? RNA helicase, putative  nucleic acid binding (IEA) ? ATP-dependent RNA helicase activity ? helicase activity (IEA) ? ATP binding (IEA) ? ATP-dependent helicase activity (IEA) ?  YHR065C ? ATP-dependent RNA helicase activity ? nucleolus ? 35S primary transcript processing ? ribosome biogenesis and assembly ?  0  BLAST| PFE1310c ? hypothetical protein  \*\* also with: YCR057C, clust.pair #22 \*\* also with: YPR169W, clust.pair #23 YLR129W ? small nucleolar ribonucleoprotein complex ? processing of 20S pre-rRNA ? snoRNA binding ? ribosome biogenesis and assembly ?  0.0006  BLAST| PFE1310c ? hypothetical protein  \*\* also with: YLR129W, clust.pair #22 \*\* also with: YPR169W, clust.pair #23 YCR057C ? cytokinesis ? small nucleolar ribonucleoprotein complex ? cytoplasm ? 35S primary transcript processing ? establishment of cell polarity (sensu Fungi) ? processing of 20S pre-rRNA ? snoRNA binding ? 90S preribosome ? ribosome biogenesis and assembly ?  0.001  BLAST| PF11\_0471 ? hypothetical protein   YCR072C ? ribosomal large subunit assembly and maintenance ? nucleolus ? ribosome ? ribosome biogenesis and assembly ?  2.8026e-45  BLAST| PF14\_0068 ? fibrillarin, putative  RNA binding (IEA) ? nucleus (IEA) ? small nucleolar ribonucleoprotein complex ? mitochondrion ? rRNA processing ?  YDL014W ? ribosomal large subunit assembly and maintenance ? rRNA modification ? RNA methylation ? nucleolus ? small nucleolar ribonucleoprotein complex ? ribosome ? 35S primary transcript processing ? methyltransferase activity ? processing of 20S pre-rRNA ? snoRNA 3'-end processing ? ribosome biogenesis and assembly ?  0  BLAST| PF08\_0130 ? wd repeat protein, putative   YCR057C ? cytokinesis ? small nucleolar ribonucleoprotein complex ? cytoplasm ? 35S primary transcript processing ? establishment of cell polarity (sensu Fungi) ? processing of 20S pre-rRNA ? snoRNA binding ? 90S preribosome ? ribosome biogenesis and assembly ?  0  BLAST| PFE1115c ? s-adenosylmethionine-dependent methyltransferase, putative  S-adenosylmethionine-dependent methyltransferase activity ?  YCR047C ? bud site selection ? nucleus ? cytoplasm ? S-adenosylmethionine-dependent methyltransferase activity ?  0  BLAST| PF07\_0067 ? hypothetical protein   YGR245C ? nucleus ? traversing start control point of mitotic cell cycle ? actin cytoskeleton organization and biogenesis ? ribosome biogenesis and assembly ? ribosome assembly ?  1e-09  BLAST| PF14\_0156 ? dimethyladenosine transferase, putative  rRNA modification ? rRNA (adenine-N6,N6-)-dimethyltransferase activity (IEA) ? mitochondrion ? rRNA processing (IEA) ? rRNA methyltransferase activity ? S-adenosylmethionine-dependent methyltransferase activity (IEA) ? rRNA (adenine) methyltransferase activity (IEA) ?  YPL266W ? rRNA modification ? rRNA (adenine-N6,N6-)-dimethyltransferase activity ? nucleolus ? 35S primary transcript processing ? ribosome biogenesis and assembly ?  0  BLAST| PF13\_0219 ? hypothetical protein   YOR206W ? ribosome-nucleus export ? nucleus ? mitochondrion ? Noc1p-Noc2p complex ? Noc2p-Noc3p complex ? ribosome biogenesis and assembly ? ribosome assembly ?  0.074  BLAST | | | | | | | | | | | | | | | | | | | | | | | | | | | | | | | | | | | | | | | | | | | | | | | | | | | | | | | | | | | | | | | | | | | | | | | | | | | | | | | | | | | | | | |

## Cluster Pair #23: 18 gene pairs.

|  |  |  |  |  |  |  |  |  |  |  |  |  |  |  |  |  |  |  |  |  |  |  |  |  |  |  |  |  |  |  |  |  |  |  |  |  |  |  |  |  |  |  |  |  |  |  |  |  |  |  |  |  |  |  |  |  |
| --- | --- | --- | --- | --- | --- | --- | --- | --- | --- | --- | --- | --- | --- | --- | --- | --- | --- | --- | --- | --- | --- | --- | --- | --- | --- | --- | --- | --- | --- | --- | --- | --- | --- | --- | --- | --- | --- | --- | --- | --- | --- | --- | --- | --- | --- | --- | --- | --- | --- | --- | --- | --- | --- | --- | --- | --- |
| P.falciparum S.cerevisiae Blast evalue|  |  |  |  |  |  |  |  |  |  |  |  |  |  |  |  |  |  |  |  |  |  |  |  |  |  |  |  |  |  |  |  |  |  |  |  |  |  |  |  |  |  |  |  |  |  |  |  |  |  |  |  |  |  | | --- | --- | --- | --- | --- | --- | --- | --- | --- | --- | --- | --- | --- | --- | --- | --- | --- | --- | --- | --- | --- | --- | --- | --- | --- | --- | --- | --- | --- | --- | --- | --- | --- | --- | --- | --- | --- | --- | --- | --- | --- | --- | --- | --- | --- | --- | --- | --- | --- | --- | --- | --- | --- | --- | | PF14\_0661 ? hypothetical protein, conserved  nucleic acid binding (IEA) ?  YOR145C ? nucleus ? nucleolus ? rRNA processing ? 35S primary transcript processing ? protein complex assembly ? ribosome biogenesis and assembly ? unfolded protein binding ?  0  BLAST| PF07\_0122 ? hypothetical protein, conserved   YOL077C ? ribosomal large subunit assembly and maintenance ? nucleolus ? 5S rRNA binding ? rRNA primary transcript binding ? ribosome biogenesis and assembly ?  1e-35  BLAST| PF14\_0150 ? RNA polymerase small subunit, putative  DNA binding (IEA) ? DNA-directed RNA polymerase activity ? transcription ? protein dimerization activity (IEA) ?  YNL113W ? DNA-directed RNA polymerase activity ? DNA-directed RNA polymerase III complex ? DNA-directed RNA polymerase I complex ? transcription from RNA polymerase I promoter ? transcription from RNA polymerase III promoter ? ribosome biogenesis and assembly ?  1e-18  BLAST| PF10\_0194 ? hypothetical protein  nucleic acid binding (IEA) ?  YOL041C ? RNA binding ? nucleolus ? rRNA metabolism ?  1e-13  BLAST| PF14\_0185 ? ATP-dependent RNA helicase, putative  nucleic acid binding (IEA) ? ATP-dependent RNA helicase activity ? helicase activity (IEA) ? ATP binding (IEA) ? ATP-dependent helicase activity (IEA) ? \*\* also with: YMR290C, clust.pair #21 \*\* also with: YHR065C, clust.pair #22 \*\* also with: YGL078C, clust.pair #22 \*\* also with: YJL033W, clust.pair #22 YFL002C ? ribosomal large subunit assembly and maintenance ? ATP-dependent RNA helicase activity ? nucleolus ? 35S primary transcript processing ? ribosome biogenesis and assembly ?  6e-10  BLAST| PF13\_0286 ? methyltransferase, putative  rRNA processing ? RNA methyltransferase activity ?  YCL054W ? nucleus ? nucleolus ? rRNA (uridine-2'-O-)-methyltransferase activity ? rRNA (guanine) methyltransferase activity ? processing of 27S pre-rRNA ? rRNA methylation ?  0  BLAST| PF07\_0027 ? DNA-directed RNA polymerase 2 8.2 kDa polypeptide, putative  DNA-directed RNA polymerase activity ? DNA-directed RNA polymerase II, core complex ? transcription (IEA) ? transcription from RNA polymerase II promoter ?  YOR210W ? DNA-directed RNA polymerase activity ? DNA-directed RNA polymerase II, core complex ? DNA-directed RNA polymerase III complex ? DNA-directed RNA polymerase I complex ? transcription from RNA polymerase I promoter ? transcription from RNA polymerase II promoter ? transcription from RNA polymerase III promoter ?  2e-17  BLAST| PFB0175c ? hypothetical protein   YAL025C ? nucleolus ? processing of 27S pre-rRNA ? ribosome biogenesis and assembly ? ribosomal large subunit biogenesis ?  1e-25  BLAST| PF10\_0277 ? hypothetical protein, conserved   YKL172W ? nuclear division ? nucleolus ? rRNA processing ? ribosome biogenesis and assembly ?  2e-07  BLAST| PFB0290c ? transcription factor, putative  DNA binding (IEA) ? transcription factor activity ? DNA-directed RNA polymerase III complex ? RNA elongation (IEA) ? regulation of transcription, DNA-dependent (IEA) ? transcription from RNA polymerase III promoter ?  YDR045C ? DNA-directed RNA polymerase activity ? DNA-directed RNA polymerase III complex ? transcription from RNA polymerase III promoter ?  7e-15  BLAST| PFB0370c ? RNA-binding protein, putative   YCL059C ? nucleolus ? small nucleolar ribonucleoprotein complex ? rRNA processing ? 35S primary transcript processing ? ribosome biogenesis and assembly ?  0  BLAST| PF13\_0035 ? hypothetical protein   YDR449C ? small nucleolar ribonucleoprotein complex ? processing of 20S pre-rRNA ? snoRNA binding ? ribosome biogenesis and assembly ?  4e-07  BLAST| PFE0515w ? hypothetical protein   YOL022C ? cytoplasm ?  0.005  BLAST| PFE1310c ? hypothetical protein  \*\* also with: YLR129W, clust.pair #22 \*\* also with: YCR057C, clust.pair #22 YPR169W ? nucleus ? nucleolus ? protein monoubiquitination ?  0.0001  BLAST| PFI1070c ? hypothetical protein   YHR088W ? ribosomal large subunit assembly and maintenance ? nucleolus ? processing of 27S pre-rRNA ? rRNA primary transcript binding ? ribosome biogenesis and assembly ?  5e-18  BLAST| PFE1435c ? hypothetical protein   YER006W ? GTPase activity ? nucleus ? nucleolus ? rRNA processing ? ribosome biogenesis and assembly ?  4e-09  BLAST| PF11\_0191 ? hypothetical protein   YLR197W ? rRNA modification ? nucleus ? nucleolus ? small nucleolar ribonucleoprotein complex ? 35S primary transcript processing ? processing of 20S pre-rRNA ? box C/D snoRNP complex ? ribosome biogenesis and assembly ?  0  BLAST| PF14\_0174 ? hypothetical protein, conserved  RNA binding (IEA) ? pseudouridylate synthase activity (IEA) ? RNA processing (IEA) ?  YLR175W ? pseudouridylate synthase activity ? nucleolus ? 35S primary transcript processing ? rRNA pseudouridine synthesis ? box H/ACA snoRNP complex ? ribosome biogenesis and assembly ?  0  BLAST | | | | | | | | | | | | | | | | | | | | | | | | | | | | | | | | | | | | | | | | | | | | | | | | | | | | | | | | |

## Cluster Pair #24: 4 gene pairs.

|  |  |  |  |  |  |  |  |  |  |  |  |  |  |  |
| --- | --- | --- | --- | --- | --- | --- | --- | --- | --- | --- | --- | --- | --- | --- |
| P.falciparum S.cerevisiae Blast evalue|  |  |  |  |  |  |  |  |  |  |  |  | | --- | --- | --- | --- | --- | --- | --- | --- | --- | --- | --- | --- | | PFD0455w ? ribosomal processing protein, putative  rRNA modification ? small nucleolar ribonucleoprotein complex ?  YLL011W ? rRNA modification ? nucleolus ? small nucleolar ribonucleoprotein complex ? 35S primary transcript processing ? processing of 20S pre-rRNA ? snoRNA binding ? small nuclear ribonucleoprotein complex ?  0  BLAST| PFL0830w ? hypothetical protein  nucleic acid binding (IEA) ?  YPR112C ? nucleolus ? 35S primary transcript processing ? snoRNA binding ? rRNA primary transcript binding ?  1.4013e-45  BLAST| PF10\_0266 ? hypothetical protein   YJR002W ? nucleus ? nucleolus ? small nucleolar ribonucleoprotein complex ? 35S primary transcript processing ? processing of 20S pre-rRNA ? ribosome biogenesis and assembly ?  2e-10  BLAST| PFD0975w ? ROI kinase-like protein   YNL207W ? protein kinase activity ? nucleocytoplasmic transporter activity ? nucleus ? cytoplasm ? cytosol ? processing of 20S pre-rRNA ?  0  BLAST | | | | | | | | | | | | | | |

## Cluster Pair #25: 9 gene pairs.

|  |  |  |  |  |  |  |  |  |  |  |  |  |  |  |  |  |  |  |  |  |  |  |  |  |  |  |  |  |  |
| --- | --- | --- | --- | --- | --- | --- | --- | --- | --- | --- | --- | --- | --- | --- | --- | --- | --- | --- | --- | --- | --- | --- | --- | --- | --- | --- | --- | --- | --- |
| P.falciparum S.cerevisiae Blast evalue|  |  |  |  |  |  |  |  |  |  |  |  |  |  |  |  |  |  |  |  |  |  |  |  |  |  |  | | --- | --- | --- | --- | --- | --- | --- | --- | --- | --- | --- | --- | --- | --- | --- | --- | --- | --- | --- | --- | --- | --- | --- | --- | --- | --- | --- | | PFI0685w ? pseudouridylate synthase, putative  RNA binding (IEA) ? RNA processing (IEA) ? pseudouridine synthase activity (IEA) ? apicoplast ?  YOL066C ? pseudouridylate synthase activity ? cytoplasm ? riboflavin biosynthesis ? tRNA-pseudouridine synthase activity ? DRAP deaminase activity ? tRNA pseudouridine synthesis ?  2e-23  BLAST| PF13\_0042 ? fork head domain protein, putative  protein binding ? nucleus ?  YLR016C ? nuclear mRNA splicing, via spliceosome ? nucleus ? cytoplasm ? mRNA-nucleus export ?  1e-13  BLAST| PFB0635w ? T-complex protein 1, putative  protein binding (IEA) ? ATP binding (IEA) ? protein folding ? ATPase activity, coupled ? cellular protein metabolism (IEA) ? unfolded protein binding (IEA) ?  YJL008C ? cytoplasm ? chaperonin-containing T-complex ? cytoskeleton ? protein folding ? cytoskeleton organization and biogenesis ? unfolded protein binding ?  0  BLAST| PFE1040c ? hypothetical protein   YLR418C ? telomere maintenance ? nucleus ? DNA recombination ? RNA elongation from RNA polymerase II promoter ? transcription elongation factor complex ? histone methylation ? Cdc73/Paf1 complex ? RNA polymerase II transcription elongation factor activity ?  4e-06  BLAST| PFC0270w ? hypothetical protein   YNL281W ? telomere maintenance ? ATPase stimulator activity ? nucleus ? cytoplasm ? protein folding ? response to stress ? chaperone activator activity ? chaperone binding ?  2e-06  BLAST| PF11\_0258 ? co-chaperone GrpE, putative  adenyl-nucleotide exchange factor activity (IEA) ? protein binding (IEA) ? mitochondrion ? mitochondrial matrix ? protein folding (IEA) ? mitochondrial matrix protein import ? protein homodimerization activity (IEA) ? unfolded protein binding ? chaperone binding (IEA) ?  YOR232W ? presequence translocase-associated import motor ? mitochondrion ? mitochondrial matrix ? mitochondrial matrix protein import ? unfolded protein binding ?  1e-26  BLAST| PF14\_0599 ? hypothetical protein   YPR173C ? cytoplasm ? endoplasmic reticulum ? sterol metabolism ? ATPase activity ? protein retention in Golgi ? late endosome to vacuole transport ?  0.007  BLAST| PF13\_0143 ? phosphoribosylpyrophosphate synthetase  ribose phosphate diphosphokinase activity ? pentose-phosphate shunt ? purine ribonucleoside salvage ? nucleoside metabolism (IEA) ? nucleotide biosynthesis (IEA) ?  YER099C ? histidine biosynthesis ? tryptophan biosynthesis ? ribose phosphate diphosphokinase activity ? cytoplasm ? purine ribonucleoside salvage ? 'de novo' IMP biosynthesis ? 'de novo' pyrimidine base biosynthesis ?  0  BLAST| PFA0145c ? aspartyl-tRNA synthetase  nucleic acid binding (IEA) ? tRNA ligase activity (IEA) ? aspartate-tRNA ligase activity ? ATP binding (IEA) ? cytoplasm (IEA) ? tRNA aminoacylation for protein translation (IEA) ? aspartyl-tRNA aminoacylation ?  YLL018C ? RNA binding ? aspartate-tRNA ligase activity ? cytoplasm ? protein biosynthesis ?  0  BLAST | | | | | | | | | | | | | | | | | | | | | | | | | | | | | |

## Cluster Pair #26: 3 gene pairs.

|  |  |  |  |  |  |  |  |  |  |  |  |
| --- | --- | --- | --- | --- | --- | --- | --- | --- | --- | --- | --- |
| P.falciparum S.cerevisiae Blast evalue|  |  |  |  |  |  |  |  |  | | --- | --- | --- | --- | --- | --- | --- | --- | --- | | MAL7P1.147 ? ubiquitin carboxyl-terminal hydrolase, putative  cysteine-type endopeptidase activity (IEA) ? ubiquitin thiolesterase activity ? ubiquitin-dependent protein catabolism (IEA) ? protein deubiquitination ?  YMR304W ? ubiquitin-specific protease activity ? cytoplasm ? protein deubiquitination ?  0  BLAST| PF14\_0276 ? ribosomal protein L15, putative  structural constituent of ribosome ? intracellular (IEA) ? mitochondrion ? mitochondrial large ribosomal subunit ? ribosome (IEA) ? protein biosynthesis ?  YNL284C ? structural constituent of ribosome ? mitochondrion ? mitochondrial large ribosomal subunit ? protein biosynthesis ?  5e-10  BLAST| PF11\_0408 ? hypothetical protein   YNL306W ? structural constituent of ribosome ? mitochondrion ? mitochondrial small ribosomal subunit ? protein biosynthesis ?  0.047  BLAST | | | | | | | | | | | |

## Cluster Pair #27: 7 gene pairs.

|  |  |  |  |  |  |  |  |  |  |  |  |  |  |  |  |  |  |  |  |  |  |  |  |
| --- | --- | --- | --- | --- | --- | --- | --- | --- | --- | --- | --- | --- | --- | --- | --- | --- | --- | --- | --- | --- | --- | --- | --- |
| P.falciparum S.cerevisiae Blast evalue|  |  |  |  |  |  |  |  |  |  |  |  |  |  |  |  |  |  |  |  |  | | --- | --- | --- | --- | --- | --- | --- | --- | --- | --- | --- | --- | --- | --- | --- | --- | --- | --- | --- | --- | --- | | PF07\_0023 ? DNA replication licensing factor mcm7 homologue, putative  nucleotide binding (IEA) ? nucleic acid binding (IEA) ? DNA binding (IEA) ? ATP binding (IEA) ? nucleus (IEA) ? DNA replication initiation ? DNA-dependent ATPase activity ? photosynthesis (IEA) ? chlorophyll biosynthesis (IEA) ? magnesium chelatase activity (IEA) ? ATPase activity (IEA) ? nucleoside-triphosphatase activity (IEA) ?  YBR202W ? chromatin binding ? ATP-dependent DNA helicase activity ? ATP binding ? nucleus ? pre-replicative complex ? cytoplasm ? pre-replicative complex formation and maintenance ? DNA unwinding ? DNA replication initiation ? MCM complex ?  0  BLAST| PF13\_0291 ? replication licensing factor, putative  nucleic acid binding (IEA) ? DNA binding ? ATP binding (IEA) ? nucleus (IEA) ? DNA replication initiation ? DNA-dependent ATPase activity ? ATPase activity (IEA) ?  YGL201C ? chromatin binding ? ATP-dependent DNA helicase activity ? nucleus ? pre-replicative complex ? cytoplasm ? DNA unwinding ? DNA replication initiation ? MCM complex ?  0  BLAST| PFE1345c ? minichromosome maintenance protein 3, putative  nucleotide binding (IEA) ? nucleic acid binding (IEA) ? DNA binding (IEA) ? ATP binding (IEA) ? nucleus (IEA) ? DNA replication initiation (IEA) ? DNA-dependent ATPase activity (IEA) ? photosynthesis (IEA) ? chlorophyll biosynthesis (IEA) ? magnesium chelatase activity (IEA) ? nucleoside-triphosphatase activity (IEA) ?  YEL032W ? chromatin binding ? ATP-dependent DNA helicase activity ? nucleus ? pre-replicative complex ? cytoplasm ? pre-replicative complex formation and maintenance ? DNA unwinding ? DNA replication initiation ? MCM complex ?  0  BLAST| PFL0560c ? minichromosome maintenance protein, putative  nucleic acid binding (IEA) ? DNA binding (IEA) ? ATP-dependent DNA helicase activity ? ATP binding ? pre-replicative complex ? DNA replication initiation ? DNA strand elongation ? DNA-dependent ATPase activity ? \*\* also with: YLR274W, clust.pair #27 YBL023C ? chromatin binding ? ATP-dependent DNA helicase activity ? nucleus ? pre-replicative complex ? cytoplasm ? pre-replicative complex formation and maintenance ? DNA unwinding ? DNA replication initiation ? MCM complex ?  2e-37  BLAST| PFL0560c ? minichromosome maintenance protein, putative  nucleic acid binding (IEA) ? DNA binding (IEA) ? ATP-dependent DNA helicase activity ? ATP binding ? pre-replicative complex ? DNA replication initiation ? DNA strand elongation ? DNA-dependent ATPase activity ? \*\* also with: YBL023C, clust.pair #27 YLR274W ? chromatin binding ? ATP-dependent DNA helicase activity ? pre-replicative complex ? replication fork ? cytoplasm ? pre-replicative complex formation and maintenance ? DNA unwinding ? DNA replication initiation ? establishment of chromatin silencing ? MCM complex ?  1e-33  BLAST| PFL1370w ? NIMA-related protein kinase %28Pfnek-1%29  protein kinase activity (IEA) ? protein serine/threonine kinase activity ? protein-tyrosine kinase activity (IEA) ? ATP binding (IEA) ? protein amino acid phosphorylation ? mitosis ?  YAR018C ? protein kinase activity ? chromosome segregation ?  1e-38  BLAST| PFL0580w ? DNA replication licensing factor mcm5, putative  nucleic acid binding (IEA) ? DNA binding (IEA) ? ATP-dependent DNA helicase activity ? ATP binding ? nucleus (IEA) ? pre-replicative complex ? DNA replication initiation ? DNA strand elongation ? DNA-dependent ATPase activity ?  YLR274W ? chromatin binding ? ATP-dependent DNA helicase activity ? pre-replicative complex ? replication fork ? cytoplasm ? pre-replicative complex formation and maintenance ? DNA unwinding ? DNA replication initiation ? establishment of chromatin silencing ? MCM complex ?  0  BLAST | | | | | | | | | | | | | | | | | | | | | | | |

## Cluster Pair #28: 9 gene pairs.

|  |  |  |  |  |  |  |  |  |  |  |  |  |  |  |  |  |  |  |  |  |  |  |  |  |  |  |  |  |  |
| --- | --- | --- | --- | --- | --- | --- | --- | --- | --- | --- | --- | --- | --- | --- | --- | --- | --- | --- | --- | --- | --- | --- | --- | --- | --- | --- | --- | --- | --- |
| P.falciparum S.cerevisiae Blast evalue|  |  |  |  |  |  |  |  |  |  |  |  |  |  |  |  |  |  |  |  |  |  |  |  |  |  |  | | --- | --- | --- | --- | --- | --- | --- | --- | --- | --- | --- | --- | --- | --- | --- | --- | --- | --- | --- | --- | --- | --- | --- | --- | --- | --- | --- | | PF11\_0488 ? hypothetical protein  protein kinase activity (IEA) ? protein serine/threonine kinase activity (IEA) ? protein-tyrosine kinase activity (IEA) ? ATP binding (IEA) ? protein amino acid phosphorylation (IEA) ?  YPL153C ? DNA replication origin binding ? protein threonine/tyrosine kinase activity ? nucleus ? nucleobase, nucleoside, nucleotide and nucleic acid metabolism ? DNA replication initiation ? DNA repair ? deoxyribonucleoside triphosphate biosynthesis ?  2e-14  BLAST| PFI0530c ? DNA primase, large subunit, putative  DNA primase activity (IEA) ? alpha DNA polymerase:primase complex (IEA) ? DNA replication, synthesis of RNA primer (IEA) ?  YKL045W ? DNA synthesis during DNA repair ? DNA primase activity ? nucleus ? nuclear membrane ? alpha DNA polymerase:primase complex ? DNA replication ? DNA replication, synthesis of RNA primer ? DNA replication initiation ? lagging strand elongation ?  9.80909e-45  BLAST| PF14\_0254 ? DNA mismatch repair protein Msh2p, putative  damaged DNA binding ? ATP binding (IEA) ? DNA metabolism (IEA) ? mismatch repair ?  YOL090W ? nuclear chromosome ? meiotic mismatch repair ? removal of nonhomologous ends ? ATP binding ? nucleus ? mismatch repair ? DNA recombination ? meiotic gene conversion ? mitotic recombination ? ATPase activity ? chromatin silencing at silent mating-type cassette ?  0  BLAST| PFD0470c ? replication factor a protein, putative  nucleic acid binding (IEA) ? DNA replication factor A complex ? DNA replication ? DNA-dependent DNA replication ?  YAR007C ? double-strand break repair via homologous recombination ? chromosome, telomeric region ? damaged DNA binding ? single-stranded DNA binding ? nucleus ? DNA replication factor A complex ? cytoplasm ? DNA unwinding ? DNA replication, synthesis of RNA primer ? DNA strand elongation ? nucleotide-excision repair ? postreplication repair ? DNA recombination ?  9.94922e-44  BLAST| PFI0235w ? replication factor A-related protein, putative  nucleic acid binding (IEA) ?  YAR007C ? double-strand break repair via homologous recombination ? chromosome, telomeric region ? damaged DNA binding ? single-stranded DNA binding ? nucleus ? DNA replication factor A complex ? cytoplasm ? DNA unwinding ? DNA replication, synthesis of RNA primer ? DNA strand elongation ? nucleotide-excision repair ? postreplication repair ? DNA recombination ?  1e-28  BLAST| PF07\_0012 ? hypothetical protein   YAR003W ? telomere maintenance ? chromatin silencing at telomere ? regulation of transcription, DNA-dependent ? histone methylation ? histone lysine N-methyltransferase activity (H3-K4 specific) ? COMPASS complex ?  8e-06  BLAST| MAL13P1.326 ? ferrochelatase, putative  ferrochelatase activity ? heme biosynthesis ? ferrous iron binding ?  YOR176W ? ferrochelatase activity ? mitochondrion ? mitochondrial inner membrane ? heme biosynthesis ?  8e-13  BLAST| PF13\_0328 ? proliferating cell nuclear antigen  DNA binding (IEA) ? nucleus (IEA) ? DNA replication factor C complex (IEA) ? regulation of DNA replication (IEA) ? DNA polymerase processivity factor activity (IEA) ?  YBR088C ? nucleus ? replication fork ? leading strand elongation ? lagging strand elongation ? mutagenesis ? base-excision repair ? nucleotide-excision repair ? mismatch repair ? postreplication repair ? chromatin silencing at telomere ? DNA polymerase processivity factor activity ? chromatin silencing at silent mating-type cassette ?  2e-37  BLAST| PFE0270c ? DNA repair protein, putative  damaged DNA binding ? ATP binding (IEA) ? DNA metabolism (IEA) ? DNA repair ? mismatch repair (IEA) ?  YDR097C ? meiotic mismatch repair ? ATP binding ? nucleus ? mismatch repair ? ATPase activity ?  0  BLAST | | | | | | | | | | | | | | | | | | | | | | | | | | | | | |

## Cluster Pair #29: 7 gene pairs.

|  |  |  |  |  |  |  |  |  |  |  |  |  |  |  |  |  |  |  |  |  |  |  |  |
| --- | --- | --- | --- | --- | --- | --- | --- | --- | --- | --- | --- | --- | --- | --- | --- | --- | --- | --- | --- | --- | --- | --- | --- |
| P.falciparum S.cerevisiae Blast evalue|  |  |  |  |  |  |  |  |  |  |  |  |  |  |  |  |  |  |  |  |  | | --- | --- | --- | --- | --- | --- | --- | --- | --- | --- | --- | --- | --- | --- | --- | --- | --- | --- | --- | --- | --- | | PF13\_0316 ? 40S ribosomal protein S13  structural constituent of ribosome ? intracellular (IEA) ? ribosome (IEA) ? cytosolic small ribosomal subunit (sensu Eukaryota) ? protein biosynthesis ?  YDR064W ? structural constituent of ribosome ? cytosolic small ribosomal subunit (sensu Eukaryota) ? protein biosynthesis ?  0  BLAST| PF14\_0296 ? ribosomal protein L14, putative  structural constituent of ribosome ? intracellular (IEA) ? ribosome (IEA) ? cytosolic small ribosomal subunit (sensu Eukaryota) ? protein biosynthesis ? apicoplast ? \*\* also with: YKL006W, clust.pair #33 YHL001W ? RNA binding ? structural constituent of ribosome ? cytosolic large ribosomal subunit (sensu Eukaryota) ? protein biosynthesis ?  0.006  BLAST| PFC0200w ? 60S Ribosomal protein L44, putative  structural constituent of ribosome ? intracellular (IEA) ? ribosome (IEA) ? cytosolic large ribosomal subunit (sensu Eukaryota) ? protein biosynthesis ? \*\* also with: YHR141C, clust.pair #33 YNL162W ? structural constituent of ribosome ? cytosolic large ribosomal subunit (sensu Eukaryota) ? protein biosynthesis ?  6e-30  BLAST| PFC0290w ? 40S ribosomal protein S23, putative  nucleic acid binding (IEA) ? structural constituent of ribosome ? intracellular (IEA) ? ribosome (IEA) ? cytosolic small ribosomal subunit (sensu Eukaryota) ? protein biosynthesis ? small ribosomal subunit (IEA) ? \*\* also with: YGR118W, clust.pair #32 YPR132W ? telomere maintenance ? structural constituent of ribosome ? cytosolic small ribosomal subunit (sensu Eukaryota) ? protein biosynthesis ? regulation of translational fidelity ?  0  BLAST| PF11\_0260 ? ribosomal protein L35, putative  structural constituent of ribosome ? intracellular (IEA) ? ribosome (IEA) ? protein biosynthesis ? large ribosomal subunit ? \*\* also with: YDL136W, clust.pair #31 YDL191W ? structural constituent of ribosome ? cytosolic large ribosomal subunit (sensu Eukaryota) ? protein biosynthesis ?  3e-08  BLAST| MAL13P1.209 ? 60S ribosomal subunit porotein L18, putative  structural constituent of ribosome ? intracellular (IEA) ? ribosome (IEA) ? cytosolic large ribosomal subunit (sensu Eukaryota) ? protein biosynthesis ? \*\* also with: YNL301C, clust.pair #33 YOL120C ? structural constituent of ribosome ? cytosolic large ribosomal subunit (sensu Eukaryota) ? protein biosynthesis ?  8.00001e-42  BLAST| PF11\_0065 ? ribosomal protein S4, putative  RNA binding ? structural constituent of ribosome ? intracellular (IEA) ? mitochondrion ? ribosome (IEA) ? protein biosynthesis ? small ribosomal subunit ? \*\* also with: YHR203C, clust.pair #32 YJR145C ? telomere maintenance ? structural constituent of ribosome ? cytoplasm ? cytosolic small ribosomal subunit (sensu Eukaryota) ? protein biosynthesis ? processing of 20S pre-rRNA ?  0  BLAST | | | | | | | | | | | | | | | | | | | | | | | |

## Cluster Pair #30: 7 gene pairs.

|  |  |  |  |  |  |  |  |  |  |  |  |  |  |  |  |  |  |  |  |  |  |  |  |
| --- | --- | --- | --- | --- | --- | --- | --- | --- | --- | --- | --- | --- | --- | --- | --- | --- | --- | --- | --- | --- | --- | --- | --- |
| P.falciparum S.cerevisiae Blast evalue|  |  |  |  |  |  |  |  |  |  |  |  |  |  |  |  |  |  |  |  |  | | --- | --- | --- | --- | --- | --- | --- | --- | --- | --- | --- | --- | --- | --- | --- | --- | --- | --- | --- | --- | --- | | PF14\_0083 ? ribosomal protein S8e, putative  structural constituent of ribosome ? intracellular (IEA) ? mitochondrion ? cytosolic small ribosomal subunit (sensu Eukaryota) ? protein biosynthesis ? \*\* also with: YBL072C, clust.pair #31 YER102W ? structural constituent of ribosome ? cytosolic small ribosomal subunit (sensu Eukaryota) ? protein biosynthesis ?  0  BLAST| PF07\_0043 ? 60S ribosomal protein L34-a, putative  structural constituent of ribosome (IEA) ? intracellular (IEA) ? ribosome (IEA) ? protein biosynthesis (IEA) ? \*\* also with: YIL052C, clust.pair #32 YER056C-A ? structural constituent of ribosome ? cytosolic large ribosomal subunit (sensu Eukaryota) ? protein biosynthesis ?  1e-30  BLAST| PFD0770c ? ribosomal protein l15, putative  structural constituent of ribosome ? intracellular (IEA) ? ribosome ? protein biosynthesis ? \*\* also with: YMR121C, clust.pair #33 YLR029C ? RNA binding ? structural constituent of ribosome ? cytosolic large ribosomal subunit (sensu Eukaryota) ? protein biosynthesis ?  0  BLAST| PF08\_0039 ? ribosomal protein, putative  structural constituent of ribosome ? intracellular (IEA) ? ribosome (IEA) ? cytosolic large ribosomal subunit (sensu Eukaryota) ? protein biosynthesis ?  YLR061W ? structural constituent of ribosome ? cytosolic large ribosomal subunit (sensu Eukaryota) ? protein biosynthesis ?  1e-07  BLAST| PFB0885w ? 40S ribosomal protein S30, putative  structural constituent of ribosome ? intracellular (IEA) ? mitochondrion ? ribosome (IEA) ? cytosolic small ribosomal subunit (sensu Eukaryota) ? protein biosynthesis ?  YOR182C ? telomere maintenance ? structural constituent of ribosome ? cytosolic small ribosomal subunit (sensu Eukaryota) ? protein biosynthesis ?  4e-13  BLAST| PF13\_0049 ? 60S ribosomal protein L24, putative  structural constituent of ribosome ? intracellular (IEA) ? ribosome (IEA) ? cytosolic large ribosomal subunit (sensu Eukaryota) ? protein biosynthesis ? \*\* also with: YGL031C, clust.pair #30 YGR148C ? RNA binding ? structural constituent of ribosome ? cytosolic large ribosomal subunit (sensu Eukaryota) ? protein biosynthesis ?  3e-10  BLAST| PF13\_0049 ? 60S ribosomal protein L24, putative  structural constituent of ribosome ? intracellular (IEA) ? ribosome (IEA) ? cytosolic large ribosomal subunit (sensu Eukaryota) ? protein biosynthesis ? \*\* also with: YGR148C, clust.pair #30 YGL031C ? RNA binding ? structural constituent of ribosome ? cytosolic large ribosomal subunit (sensu Eukaryota) ? protein biosynthesis ?  4e-10  BLAST | | | | | | | | | | | | | | | | | | | | | | | |

## Cluster Pair #31: 15 gene pairs.

|  |  |  |  |  |  |  |  |  |  |  |  |  |  |  |  |  |  |  |  |  |  |  |  |  |  |  |  |  |  |  |  |  |  |  |  |  |  |  |  |  |  |  |  |  |  |  |  |
| --- | --- | --- | --- | --- | --- | --- | --- | --- | --- | --- | --- | --- | --- | --- | --- | --- | --- | --- | --- | --- | --- | --- | --- | --- | --- | --- | --- | --- | --- | --- | --- | --- | --- | --- | --- | --- | --- | --- | --- | --- | --- | --- | --- | --- | --- | --- | --- |
| P.falciparum S.cerevisiae Blast evalue|  |  |  |  |  |  |  |  |  |  |  |  |  |  |  |  |  |  |  |  |  |  |  |  |  |  |  |  |  |  |  |  |  |  |  |  |  |  |  |  |  |  |  |  |  | | --- | --- | --- | --- | --- | --- | --- | --- | --- | --- | --- | --- | --- | --- | --- | --- | --- | --- | --- | --- | --- | --- | --- | --- | --- | --- | --- | --- | --- | --- | --- | --- | --- | --- | --- | --- | --- | --- | --- | --- | --- | --- | --- | --- | --- | | PF11\_0043 ? 60S acidic ribosomal protein p1, putative  structural constituent of ribosome ? intracellular (IEA) ? ribosome (IEA) ? protein biosynthesis ? translational elongation (IEA) ? large ribosomal subunit ? \*\* also with: YDL130W, clust.pair #31 YDL081C ? telomere maintenance ? structural constituent of ribosome ? cytosolic large ribosomal subunit (sensu Eukaryota) ? protein biosynthesis ? translational elongation ?  2e-06  BLAST| PF11\_0043 ? 60S acidic ribosomal protein p1, putative  structural constituent of ribosome ? intracellular (IEA) ? ribosome (IEA) ? protein biosynthesis ? translational elongation (IEA) ? large ribosomal subunit ? \*\* also with: YDL081C, clust.pair #31 YDL130W ? structural constituent of ribosome ? cytosolic large ribosomal subunit (sensu Eukaryota) ? protein biosynthesis ? translational elongation ?  2e-05  BLAST| PF08\_0019 ? guanine nucleotide-binding protein, putative  protein kinase C binding ? heterotrimeric G-protein complex ? G-protein coupled receptor protein signaling pathway ?  YMR116C ? telomere maintenance ? cytoplasm ? cytosolic small ribosomal subunit (sensu Eukaryota) ? negative regulation of protein biosynthesis ?  0  BLAST| PF14\_0083 ? ribosomal protein S8e, putative  structural constituent of ribosome ? intracellular (IEA) ? mitochondrion ? cytosolic small ribosomal subunit (sensu Eukaryota) ? protein biosynthesis ? \*\* also with: YER102W, clust.pair #30 YBL072C ? structural constituent of ribosome ? cytosolic small ribosomal subunit (sensu Eukaryota) ? protein biosynthesis ?  0  BLAST| PF14\_0391 ? ribosomal protein L1, putative  structural constituent of ribosome ? intracellular (IEA) ? ribosome (IEA) ? cytosolic large ribosomal subunit (sensu Eukaryota) ? protein biosynthesis ?  YPL220W ? structural constituent of ribosome ? cytosolic large ribosomal subunit (sensu Eukaryota) ? protein biosynthesis ?  0  BLAST| PF10\_0264 ? 40S ribosomal protein, putative  structural constituent of ribosome ? intracellular (IEA) ? ribosome (IEA) ? cytosolic small ribosomal subunit (sensu Eukaryota) ? protein biosynthesis ? small ribosomal subunit (IEA) ? \*\* also with: YGR214W, clust.pair #33 YLR048W ? ribosomal small subunit assembly and maintenance ? structural constituent of ribosome ? cytosolic small ribosomal subunit (sensu Eukaryota) ? protein biosynthesis ?  0  BLAST| PFE1005w ? 40S ribosomal subunit protein S9, putative  RNA binding (IEA) ? structural constituent of ribosome (IEA) ? intracellular (IEA) ? ribosome (IEA) ? protein biosynthesis (IEA) ? small ribosomal subunit (IEA) ? \*\* also with: YPL081W, clust.pair #32 YBR189W ? structural constituent of ribosome ? small nucleolar ribonucleoprotein complex ? cytosolic small ribosomal subunit (sensu Eukaryota) ? protein biosynthesis ? regulation of translational fidelity ?  0  BLAST| PF11\_0260 ? ribosomal protein L35, putative  structural constituent of ribosome ? intracellular (IEA) ? ribosome (IEA) ? protein biosynthesis ? large ribosomal subunit ? \*\* also with: YDL191W, clust.pair #29 YDL136W ? structural constituent of ribosome ? cytosolic large ribosomal subunit (sensu Eukaryota) ? protein biosynthesis ?  3e-08  BLAST| PF11\_0313 ? ribosomal phosphoprotein P0  structural constituent of ribosome ? intracellular (IEA) ? mitochondrion ? ribosome (IEA) ? cytosolic large ribosomal subunit (sensu Eukaryota) ? protein biosynthesis ? translational elongation (IEA) ? ribosome biogenesis and assembly (IEA) ?  YLR340W ? ribosomal large subunit assembly and maintenance ? structural constituent of ribosome ? cytosolic large ribosomal subunit (sensu Eukaryota) ? protein biosynthesis ? translational elongation ?  0  BLAST| PF14\_0585 ? ribosomal protein S28e, putative  structural constituent of ribosome ? intracellular (IEA) ? ribosome (IEA) ? cytosolic small ribosomal subunit (sensu Eukaryota) ? protein biosynthesis ? \*\* also with: YLR264W, clust.pair #33 YOR167C ? structural constituent of ribosome ? cytosolic small ribosomal subunit (sensu Eukaryota) ? protein biosynthesis ?  5e-12  BLAST| PF13\_0304 ? elongation factor 1 alpha  translation elongation factor activity ? GTP binding (IEA) ? cytoplasm (IEA) ? eukaryotic translation elongation factor 1 complex ? protein biosynthesis (IEA) ? translational elongation ? \*\* also with: YPR080W, clust.pair #32 YBR118W ? translation elongation factor activity ? ribosome ? eukaryotic translation elongation factor 1 complex ? tRNA-nucleus export ? translational elongation ?  0  BLAST| PFC0870w ? elongation factor 1 %28EF-1%29, putative  translation elongation factor activity ? eukaryotic translation elongation factor 1 complex ? translational elongation ?  YAL003W ? translation elongation factor activity ? ribosome ? eukaryotic translation elongation factor 1 complex ? translational elongation ?  3e-15  BLAST| PFC0400w ? 60S Acidic ribosomal protein P2  structural constituent of ribosome (IEA) ? intracellular (IEA) ? ribosome (IEA) ? cytosolic large ribosomal subunit (sensu Eukaryota) ? translational elongation ? large ribosomal subunit ? \*\* also with: YOL039W, clust.pair #33 YDR382W ? structural constituent of ribosome ? cytosolic large ribosomal subunit (sensu Eukaryota) ? protein biosynthesis ? translational elongation ?  9e-11  BLAST| PFE0350c ? 60S ribosomal subunit protein L4%2FL1, putative  RNA binding ? structural constituent of ribosome ? intracellular (IEA) ? ribosome (IEA) ? protein biosynthesis ? large ribosomal subunit ? \*\* also with: YDR012W, clust.pair #31 YBR031W ? structural constituent of ribosome ? cytosolic large ribosomal subunit (sensu Eukaryota) ? protein biosynthesis ?  0  BLAST| PFE0350c ? 60S ribosomal subunit protein L4%2FL1, putative  RNA binding ? structural constituent of ribosome ? intracellular (IEA) ? ribosome (IEA) ? protein biosynthesis ? large ribosomal subunit ? \*\* also with: YBR031W, clust.pair #31 YDR012W ? structural constituent of ribosome ? cytoplasm ? cytosolic large ribosomal subunit (sensu Eukaryota) ? protein biosynthesis ?  0  BLAST | | | | | | | | | | | | | | | | | | | | | | | | | | | | | | | | | | | | | | | | | | | | | | | |

## Cluster Pair #32: 14 gene pairs.

|  |  |  |  |  |  |  |  |  |  |  |  |  |  |  |  |  |  |  |  |  |  |  |  |  |  |  |  |  |  |  |  |  |  |  |  |  |  |  |  |  |  |  |  |  |
| --- | --- | --- | --- | --- | --- | --- | --- | --- | --- | --- | --- | --- | --- | --- | --- | --- | --- | --- | --- | --- | --- | --- | --- | --- | --- | --- | --- | --- | --- | --- | --- | --- | --- | --- | --- | --- | --- | --- | --- | --- | --- | --- | --- | --- |
| P.falciparum S.cerevisiae Blast evalue|  |  |  |  |  |  |  |  |  |  |  |  |  |  |  |  |  |  |  |  |  |  |  |  |  |  |  |  |  |  |  |  |  |  |  |  |  |  |  |  |  |  | | --- | --- | --- | --- | --- | --- | --- | --- | --- | --- | --- | --- | --- | --- | --- | --- | --- | --- | --- | --- | --- | --- | --- | --- | --- | --- | --- | --- | --- | --- | --- | --- | --- | --- | --- | --- | --- | --- | --- | --- | --- | --- | | PF14\_0448 ? ribosomal protein S2, putative  structural constituent of ribosome ? intracellular (IEA) ? ribosome (IEA) ? cytosolic small ribosomal subunit (sensu Eukaryota) ? protein biosynthesis ? small ribosomal subunit (IEA) ?  YGL123W ? structural constituent of ribosome ? small nucleolar ribonucleoprotein complex ? cytosolic small ribosomal subunit (sensu Eukaryota) ? protein biosynthesis ? regulation of translational fidelity ?  0  BLAST| PF13\_0214 ? elongation factor 1-gamma, putative  translation elongation factor activity ? glutathione transferase activity ? eukaryotic translation elongation factor 1 complex ? translational elongation ?  YKL081W ? translation elongation factor activity ? mitochondrion ? ribosome ? eukaryotic translation elongation factor 1 complex ? translational elongation ?  4e-24  BLAST| PF07\_0043 ? 60S ribosomal protein L34-a, putative  structural constituent of ribosome (IEA) ? intracellular (IEA) ? ribosome (IEA) ? protein biosynthesis (IEA) ? \*\* also with: YER056C-A, clust.pair #30 YIL052C ? telomere maintenance ? structural constituent of ribosome ? cytosolic large ribosomal subunit (sensu Eukaryota) ? protein biosynthesis ?  1e-30  BLAST| PF10\_0103 ? eukaryotic translation initiation factor 2, beta, putative  RNA binding ? translation initiation factor activity ? eukaryotic translation initiation factor 2 complex ? translational initiation ?  YPL237W ? translation initiation factor activity ? ribosome ? eukaryotic translation initiation factor 2 complex ? translational initiation ?  6e-32  BLAST| PF10\_0043 ? ribosomal protein L13, putative  structural constituent of ribosome ? intracellular (IEA) ? ribosome (IEA) ? cytosolic large ribosomal subunit (sensu Eukaryota) ? protein biosynthesis ? large ribosomal subunit (IEA) ? \*\* also with: YNL069C, clust.pair #33 YIL133C ? RNA binding ? structural constituent of ribosome ? cytosolic large ribosomal subunit (sensu Eukaryota) ? protein biosynthesis ?  0  BLAST| PFC0290w ? 40S ribosomal protein S23, putative  nucleic acid binding (IEA) ? structural constituent of ribosome ? intracellular (IEA) ? ribosome (IEA) ? cytosolic small ribosomal subunit (sensu Eukaryota) ? protein biosynthesis ? small ribosomal subunit (IEA) ? \*\* also with: YPR132W, clust.pair #29 YGR118W ? telomere maintenance ? structural constituent of ribosome ? cytosolic small ribosomal subunit (sensu Eukaryota) ? protein biosynthesis ? regulation of translational fidelity ?  0  BLAST| PFE1005w ? 40S ribosomal subunit protein S9, putative  RNA binding (IEA) ? structural constituent of ribosome (IEA) ? intracellular (IEA) ? ribosome (IEA) ? protein biosynthesis (IEA) ? small ribosomal subunit (IEA) ? \*\* also with: YBR189W, clust.pair #31 YPL081W ? structural constituent of ribosome ? small nucleolar ribonucleoprotein complex ? cytoplasm ? cytosolic small ribosomal subunit (sensu Eukaryota) ? protein biosynthesis ? regulation of translational fidelity ?  0  BLAST| PF14\_0141 ? ribosomal protein L10, putative  structural constituent of ribosome ? intracellular (IEA) ? ribosome (IEA) ? cytosolic large ribosomal subunit (sensu Eukaryota) ? protein biosynthesis ?  YLR075W ? ribosomal large subunit assembly and maintenance ? structural constituent of ribosome ? cytosolic large ribosomal subunit (sensu Eukaryota) ? protein biosynthesis ?  0  BLAST| PF13\_0304 ? elongation factor 1 alpha  translation elongation factor activity ? GTP binding (IEA) ? cytoplasm (IEA) ? eukaryotic translation elongation factor 1 complex ? protein biosynthesis (IEA) ? translational elongation ? \*\* also with: YBR118W, clust.pair #31 YPR080W ? translation elongation factor activity ? ribosome ? eukaryotic translation elongation factor 1 complex ? tRNA-nucleus export ? translational elongation ?  0  BLAST| PF13\_0129 ? ribosomal protein L6 homologue, putative  RNA binding ? structural constituent of ribosome ? intracellular (IEA) ? ribosome (IEA) ? protein biosynthesis ? large ribosomal subunit ? \*\* also with: YNL067W, clust.pair #32 YGL147C ? structural constituent of ribosome ? cytosolic large ribosomal subunit (sensu Eukaryota) ? protein biosynthesis ?  9.99995e-41  BLAST| PF13\_0129 ? ribosomal protein L6 homologue, putative  RNA binding ? structural constituent of ribosome ? intracellular (IEA) ? ribosome (IEA) ? protein biosynthesis ? large ribosomal subunit ? \*\* also with: YGL147C, clust.pair #32 YNL067W ? structural constituent of ribosome ? cytosolic large ribosomal subunit (sensu Eukaryota) ? protein biosynthesis ?  9.99995e-41  BLAST| PF11\_0065 ? ribosomal protein S4, putative  RNA binding ? structural constituent of ribosome ? intracellular (IEA) ? mitochondrion ? ribosome (IEA) ? protein biosynthesis ? small ribosomal subunit ? \*\* also with: YJR145C, clust.pair #29 YHR203C ? telomere maintenance ? structural constituent of ribosome ? cytosolic small ribosomal subunit (sensu Eukaryota) ? protein biosynthesis ?  0  BLAST| PF07\_0079 ? 60S ribosomal protein L11a, putative  structural constituent of ribosome ? intracellular (IEA) ? ribosome (IEA) ? cytosolic large ribosomal subunit (sensu Eukaryota) ? protein biosynthesis ? \*\* also with: YPR102C, clust.pair #32 YGR085C ? ribosomal large subunit assembly and maintenance ? structural constituent of ribosome ? cytosolic large ribosomal subunit (sensu Eukaryota) ? protein biosynthesis ?  0  BLAST| PF07\_0079 ? 60S ribosomal protein L11a, putative  structural constituent of ribosome ? intracellular (IEA) ? ribosome (IEA) ? cytosolic large ribosomal subunit (sensu Eukaryota) ? protein biosynthesis ? \*\* also with: YGR085C, clust.pair #32 YPR102C ? ribosomal large subunit assembly and maintenance ? structural constituent of ribosome ? cytosolic large ribosomal subunit (sensu Eukaryota) ? protein biosynthesis ?  0  BLAST | | | | | | | | | | | | | | | | | | | | | | | | | | | | | | | | | | | | | | | | | | | | |

## Cluster Pair #33: 22 gene pairs.

|  |  |  |  |  |  |  |  |  |  |  |  |  |  |  |  |  |  |  |  |  |  |  |  |  |  |  |  |  |  |  |  |  |  |  |  |  |  |  |  |  |  |  |  |  |  |  |  |  |  |  |  |  |  |  |  |  |  |  |  |  |  |  |  |  |  |  |  |  |
| --- | --- | --- | --- | --- | --- | --- | --- | --- | --- | --- | --- | --- | --- | --- | --- | --- | --- | --- | --- | --- | --- | --- | --- | --- | --- | --- | --- | --- | --- | --- | --- | --- | --- | --- | --- | --- | --- | --- | --- | --- | --- | --- | --- | --- | --- | --- | --- | --- | --- | --- | --- | --- | --- | --- | --- | --- | --- | --- | --- | --- | --- | --- | --- | --- | --- | --- | --- | --- |
| P.falciparum S.cerevisiae Blast evalue|  |  |  |  |  |  |  |  |  |  |  |  |  |  |  |  |  |  |  |  |  |  |  |  |  |  |  |  |  |  |  |  |  |  |  |  |  |  |  |  |  |  |  |  |  |  |  |  |  |  |  |  |  |  |  |  |  |  |  |  |  |  |  |  |  |  | | --- | --- | --- | --- | --- | --- | --- | --- | --- | --- | --- | --- | --- | --- | --- | --- | --- | --- | --- | --- | --- | --- | --- | --- | --- | --- | --- | --- | --- | --- | --- | --- | --- | --- | --- | --- | --- | --- | --- | --- | --- | --- | --- | --- | --- | --- | --- | --- | --- | --- | --- | --- | --- | --- | --- | --- | --- | --- | --- | --- | --- | --- | --- | --- | --- | --- | | PF14\_0125 ? deoxyhypusine synthase  protein biosynthesis ? hypusine biosynthesis from peptidyl-lysine ? membrane ? spermidine catabolism to deoxyhypusine, using deoxyhypusine synthase ?  YHR068W ? cytoplasm ? hypusine biosynthesis from peptidyl-lysine ? transferase activity, transferring alkyl or aryl (other than methyl) groups ?  9.80909e-45  BLAST| PFD1070w ? eukaryotic initiation factor, putative  nucleic acid binding (IEA) ? translation initiation factor activity ? ATP-dependent RNA helicase activity ? helicase activity (IEA) ? ATP binding (IEA) ? regulation of translational initiation ? ATP-dependent helicase activity (IEA) ? eukaryotic translation initiation factor 4F complex ? \*\* also with: YKR059W, clust.pair #33 YJL138C ? RNA helicase activity ? translation initiation factor activity ? cytoplasm ? ribosome ? translational initiation ? regulation of translational initiation ? eukaryotic translation initiation factor 4F complex ?  0  BLAST| PFD1070w ? eukaryotic initiation factor, putative  nucleic acid binding (IEA) ? translation initiation factor activity ? ATP-dependent RNA helicase activity ? helicase activity (IEA) ? ATP binding (IEA) ? regulation of translational initiation ? ATP-dependent helicase activity (IEA) ? eukaryotic translation initiation factor 4F complex ? \*\* also with: YJL138C, clust.pair #33 YKR059W ? telomere maintenance ? translation initiation factor activity ? ATP-dependent RNA helicase activity ? ribosome ? translational initiation ? eukaryotic translation initiation factor 4F complex ?  0  BLAST| PFD0770c ? ribosomal protein l15, putative  structural constituent of ribosome ? intracellular (IEA) ? ribosome ? protein biosynthesis ? \*\* also with: YLR029C, clust.pair #30 YMR121C ? RNA binding ? structural constituent of ribosome ? cytosolic large ribosomal subunit (sensu Eukaryota) ? protein biosynthesis ?  0  BLAST| PF14\_0296 ? ribosomal protein L14, putative  structural constituent of ribosome ? intracellular (IEA) ? ribosome (IEA) ? cytosolic small ribosomal subunit (sensu Eukaryota) ? protein biosynthesis ? apicoplast ? \*\* also with: YHL001W, clust.pair #29 YKL006W ? RNA binding ? structural constituent of ribosome ? cytosolic large ribosomal subunit (sensu Eukaryota) ? protein biosynthesis ?  0.004  BLAST| MAL8P1.40 ? RNA-binding protein, putative  nucleic acid binding (IEA) ? RNA binding ? regulation of protein biosynthesis ? regulation of translation ?  YER165W ? nucleus ? cytoplasm ? ribosome ? regulation of translational initiation ? poly(A) binding ?  4e-13  BLAST| PF08\_0075 ? 60S ribosomal protein L13, putative  structural constituent of ribosome ? intracellular (IEA) ? ribosome (IEA) ? cytosolic large ribosomal subunit (sensu Eukaryota) ? protein biosynthesis ?  YDL082W ? structural constituent of ribosome ? cytosolic large ribosomal subunit (sensu Eukaryota) ? protein biosynthesis ?  3e-18  BLAST| PFC0200w ? 60S Ribosomal protein L44, putative  structural constituent of ribosome ? intracellular (IEA) ? ribosome (IEA) ? cytosolic large ribosomal subunit (sensu Eukaryota) ? protein biosynthesis ? \*\* also with: YNL162W, clust.pair #29 YHR141C ? structural constituent of ribosome ? cytosolic large ribosomal subunit (sensu Eukaryota) ? protein biosynthesis ?  6e-30  BLAST| PF10\_0264 ? 40S ribosomal protein, putative  structural constituent of ribosome ? intracellular (IEA) ? ribosome (IEA) ? cytosolic small ribosomal subunit (sensu Eukaryota) ? protein biosynthesis ? small ribosomal subunit (IEA) ? \*\* also with: YLR048W, clust.pair #31 YGR214W ? ribosomal small subunit assembly and maintenance ? structural constituent of ribosome ? cytosolic small ribosomal subunit (sensu Eukaryota) ? protein biosynthesis ?  0  BLAST| PF10\_0043 ? ribosomal protein L13, putative  structural constituent of ribosome ? intracellular (IEA) ? ribosome (IEA) ? cytosolic large ribosomal subunit (sensu Eukaryota) ? protein biosynthesis ? large ribosomal subunit (IEA) ? \*\* also with: YIL133C, clust.pair #32 YNL069C ? RNA binding ? structural constituent of ribosome ? cytosolic large ribosomal subunit (sensu Eukaryota) ? protein biosynthesis ?  0  BLAST| PF10\_0272 ? ribosomal protein L3, putative  structural constituent of ribosome ? intracellular (IEA) ? mitochondrion ? ribosome (IEA) ? cytosolic large ribosomal subunit (sensu Eukaryota) ? protein biosynthesis ?  YOR063W ? ribosomal large subunit assembly and maintenance ? structural constituent of ribosome ? cytosolic large ribosomal subunit (sensu Eukaryota) ? protein biosynthesis ?  0  BLAST| PFE0975c ? 40S ribosomal subunit protein S24, putative  structural constituent of ribosome (IEA) ? intracellular (IEA) ? ribosome (IEA) ? protein biosynthesis (IEA) ? \*\* also with: YIL069C, clust.pair #33 YER074W ? structural constituent of ribosome ? mitochondrion ? cytosolic small ribosomal subunit (sensu Eukaryota) ? protein biosynthesis ?  3e-33  BLAST| PFE0975c ? 40S ribosomal subunit protein S24, putative  structural constituent of ribosome (IEA) ? intracellular (IEA) ? ribosome (IEA) ? protein biosynthesis (IEA) ? \*\* also with: YER074W, clust.pair #33 YIL069C ? structural constituent of ribosome ? cytosolic small ribosomal subunit (sensu Eukaryota) ? protein biosynthesis ?  3e-33  BLAST| PF14\_0230 ? Ribosomal protein family L5, putative  structural constituent of ribosome ? intracellular (IEA) ? mitochondrion ? ribosome (IEA) ? cytosolic large ribosomal subunit (sensu Eukaryota) ? protein biosynthesis ? 5S rRNA binding (IEA) ?  YPL131W ? ribosomal large subunit assembly and maintenance ? RNA binding ? structural constituent of ribosome ? cytosolic large ribosomal subunit (sensu Eukaryota) ? protein biosynthesis ?  0  BLAST| PF14\_0627 ? ribosomal protein S3, putative  nucleic acid binding (IEA) ? structural constituent of ribosome ? intracellular (IEA) ? ribosome (IEA) ? cytosolic small ribosomal subunit (sensu Eukaryota) ? protein biosynthesis ? small ribosomal subunit (IEA) ?  YNL178W ? structural constituent of ribosome ? cytosolic small ribosomal subunit (sensu Eukaryota) ? protein biosynthesis ? response to DNA damage stimulus ? nucleolar preribosome, small subunit precursor ?  0  BLAST| PF14\_0585 ? ribosomal protein S28e, putative  structural constituent of ribosome ? intracellular (IEA) ? ribosome (IEA) ? cytosolic small ribosomal subunit (sensu Eukaryota) ? protein biosynthesis ? \*\* also with: YOR167C, clust.pair #31 YLR264W ? telomere maintenance ? structural constituent of ribosome ? cytosolic small ribosomal subunit (sensu Eukaryota) ? protein biosynthesis ?  5e-12  BLAST| MAL13P1.209 ? 60S ribosomal subunit porotein L18, putative  structural constituent of ribosome ? intracellular (IEA) ? ribosome (IEA) ? cytosolic large ribosomal subunit (sensu Eukaryota) ? protein biosynthesis ? \*\* also with: YOL120C, clust.pair #29 YNL301C ? structural constituent of ribosome ? cytosolic large ribosomal subunit (sensu Eukaryota) ? protein biosynthesis ?  8.00001e-42  BLAST| PF07\_0088 ? 40S ribosomal protein S5, putative  structural constituent of ribosome ? intracellular (IEA) ? ribosome (IEA) ? cytosolic small ribosomal subunit (sensu Eukaryota) ? protein biosynthesis ? small ribosomal subunit (IEA) ?  YJR123W ? structural constituent of ribosome ? cytosolic small ribosomal subunit (sensu Eukaryota) ? protein biosynthesis ?  0  BLAST| PFL1745c ? clustered-asparagine-rich protein  nucleic acid binding (IEA) ? RNA binding ?  YER165W ? nucleus ? cytoplasm ? ribosome ? regulation of translational initiation ? poly(A) binding ?  4e-08  BLAST| PFL0665c ? RNA polymerase subunit 8c, putative  nucleic acid binding (IEA) ? DNA-directed RNA polymerase activity ? transcription (IEA) ? transcription initiation ?  YOR224C ? DNA-directed RNA polymerase activity ? DNA-directed RNA polymerase II, core complex ? DNA-directed RNA polymerase III complex ? DNA-directed RNA polymerase I complex ? transcription from RNA polymerase I promoter ? transcription from RNA polymerase II promoter ? transcription from RNA polymerase III promoter ?  1e-14  BLAST| PFC0400w ? 60S Acidic ribosomal protein P2  structural constituent of ribosome (IEA) ? intracellular (IEA) ? ribosome (IEA) ? cytosolic large ribosomal subunit (sensu Eukaryota) ? translational elongation ? large ribosomal subunit ? \*\* also with: YDR382W, clust.pair #31 YOL039W ? structural constituent of ribosome ? cytosolic large ribosomal subunit (sensu Eukaryota) ? protein biosynthesis ? translational elongation ?  2e-09  BLAST| PFL0900c ? arginyl-tRNA synthetase, putative  arginine-tRNA ligase activity ? ATP binding (IEA) ? arginyl-tRNA aminoacylation ? apicoplast ?  YDR341C ? arginine-tRNA ligase activity ? cytoplasm ? mitochondrion ? protein biosynthesis ?  3.9937e-43  BLAST | | | | | | | | | | | | | | | | | | | | | | | | | | | | | | | | | | | | | | | | | | | | | | | | | | | | | | | | | | | | | | | | | | | | |

## Cluster Pair #34: 8 gene pairs.

|  |  |  |  |  |  |  |  |  |  |  |  |  |  |  |  |  |  |  |  |  |  |  |  |  |  |  |
| --- | --- | --- | --- | --- | --- | --- | --- | --- | --- | --- | --- | --- | --- | --- | --- | --- | --- | --- | --- | --- | --- | --- | --- | --- | --- | --- |
| P.falciparum S.cerevisiae Blast evalue|  |  |  |  |  |  |  |  |  |  |  |  |  |  |  |  |  |  |  |  |  |  |  |  | | --- | --- | --- | --- | --- | --- | --- | --- | --- | --- | --- | --- | --- | --- | --- | --- | --- | --- | --- | --- | --- | --- | --- | --- | | PF13\_0349 ? nucleoside diphosphate kinase b%3B putative  nucleoside diphosphate kinase activity ? ATP binding ? GTP biosynthesis ? UTP biosynthesis ? CTP biosynthesis ?  YKL067W ? nucleoside diphosphate kinase activity ? mitochondrion ? mitochondrial intermembrane space ? cytosol ? purine nucleotide biosynthesis ? nucleoside diphosphate phosphorylation ? DNA metabolism ? nucleotide metabolism ? nucleoside triphosphate biosynthesis ? RNA metabolism ?  0  BLAST| PF08\_0038 ? hypothetical protein  structural constituent of ribosome (IEA) ? intracellular (IEA) ? ribosome (IEA) ? protein biosynthesis (IEA) ?  YJL115W ? chromatin assembly complex ? DNA replication-dependent nucleosome assembly ? DNA replication-independent nucleosome assembly ? nucleosome disassembly ? chromatin silencing at telomere ? histone acetylation ? chromatin silencing at silent mating-type cassette ? histone binding ?  0.032  BLAST| PF08\_0125 ? tubulin gamma chain  microtubule cytoskeleton organization and biogenesis ? GTPase activity (IEA) ? structural molecule activity (IEA) ? structural constituent of cytoskeleton ? GTP binding (IEA) ? microtubule organizing center ? microtubule (IEA) ? microtubule-based movement (IEA) ? protein complex (IEA) ? protein polymerization (IEA) ?  YLR212C ? gamma-tubulin complex (sensu Saccharomyces) ? structural constituent of cytoskeleton ? inner plaque of spindle pole body ? outer plaque of spindle pole body ? microtubule nucleation ? mitotic spindle organization and biogenesis in nucleus ?  0  BLAST| PF11\_0117 ? replication factor C subunit 5, putative  DNA binding (IEA) ? DNA replication factor C complex ? DNA replication ? DNA strand elongation ? protein complex (IEA) ?  YBR087W ? DNA clamp loader activity ? DNA replication factor C complex ? leading strand elongation ? mismatch repair ? sister chromatid cohesion ?  1e-38  BLAST| PFD0685c ? chromosome associated protein, putative  nuclear chromosome ? protein binding (IEA) ? ATP binding ? chromosome (IEA) ? chromosome organization and biogenesis (sensu Eukaryota) ? ATPase activity (IEA) ? chromosome organization and biogenesis (IEA) ?  YJL074C ? nuclear cohesin complex ? nucleus ? mitotic sister chromatid cohesion ? synaptonemal complex formation ? ATPase activity ? sporulation (sensu Fungi) ?  0  BLAST| PF14\_0602 ? DNA polymerase alpha subunit, putative  DNA binding (IEA) ? DNA-directed DNA polymerase activity (IEA) ? alpha DNA polymerase activity ? nucleus (IEA) ? alpha DNA polymerase:primase complex ? DNA replication (IEA) ? DNA replication, synthesis of RNA primer ?  YBL035C ? alpha DNA polymerase activity ? nucleus ? nuclear membrane ? alpha DNA polymerase:primase complex ? DNA replication initiation ? lagging strand elongation ? telomere capping ?  2e-16  BLAST| MAL8P1.18 ? hypothetical protein  nucleic acid binding (IEA) ? RNA binding (IEA) ? apicoplast ?  YJL074C ? nuclear cohesin complex ? nucleus ? mitotic sister chromatid cohesion ? synaptonemal complex formation ? ATPase activity ? sporulation (sensu Fungi) ?  0.012  BLAST| PF11\_0087 ? Rad51 homolog, putative  recombinase activity ? nucleotide binding (IEA) ? DNA binding ? damaged DNA binding ? ATP binding ? nucleus ? DNA metabolism (IEA) ? DNA repair ? DNA recombination ? DNA-dependent ATPase activity ? nucleoside-triphosphatase activity (IEA) ?  YER095W ? recombinase activity ? nuclear chromosome ? meiotic joint molecule formation ? telomerase-independent telomere maintenance ? condensed nuclear chromosome ? meiotic recombination ? DNA-dependent ATPase activity ? heteroduplex formation ? strand invasion ? double-strand break repair via single-strand annealing ?  0  BLAST | | | | | | | | | | | | | | | | | | | | | | | | | | |

## Cluster Pair #35: 4 gene pairs.

|  |  |  |  |  |  |  |  |  |  |  |  |  |  |  |
| --- | --- | --- | --- | --- | --- | --- | --- | --- | --- | --- | --- | --- | --- | --- |
| P.falciparum S.cerevisiae Blast evalue|  |  |  |  |  |  |  |  |  |  |  |  | | --- | --- | --- | --- | --- | --- | --- | --- | --- | --- | --- | --- | | PF14\_0451 ? mitochondrial ribosomal protein S14 precursor, putative  structural constituent of ribosome ? mitochondrial small ribosomal subunit ? protein biosynthesis ?  YPR166C ? structural constituent of ribosome ? mitochondrion ? mitochondrial small ribosomal subunit ? protein biosynthesis ?  5e-06  BLAST| MAL13P1.281 ? glutamate--tRNA ligase, putative  tRNA ligase activity (IEA) ? glutamate-tRNA ligase activity ? ATP binding (IEA) ? cytoplasm (IEA) ? protein biosynthesis (IEA) ? glutamyl-tRNA aminoacylation ? aminoacyl-tRNA synthetase multienzyme complex (IEA) ? apicoplast ?  YOL033W ? glutamate-tRNA ligase activity ? mitochondrion ? protein biosynthesis ? glutamyl-tRNA aminoacylation ?  0  BLAST| PFL1590c ? elongation factor g, putative  translation elongation factor activity ? GTP binding ? mitochondrion ? protein biosynthesis (IEA) ? translational elongation ?  YLR069C ? translation elongation factor activity ? mitochondrion ? translational elongation ?  0  BLAST| PFL2180w ? 50S ribosomal protein L3, putative  structural constituent of ribosome ? intracellular (IEA) ? mitochondrial large ribosomal subunit ? ribosome (IEA) ? protein biosynthesis ?  YGR220C ? ribosomal large subunit assembly and maintenance ? peptidyltransferase activity ? structural constituent of ribosome ? mitochondrion ? mitochondrial large ribosomal subunit ? protein biosynthesis ?  3e-25  BLAST | | | | | | | | | | | | | | |
