# Supplementary material for: Assessing functional annotation transfers with inter-species conserved coexpression: application to Plasmodium falciparum
Source: BMC Genomics. 2010 Jan 15;11:35. doi: 10.1186/1471-2164-11-35 (PMC2826313; doi:10.1186/1471-2164-11-35)
Supplement: Additional file 5 — Bozdech - Pilot analysis. This file presents the cluster pairs identified as revealing a conservation of coexpression when comparing the Bozdech and Pilot data. This file also provide additional information on the available functional annotations, as well as links to the BLAST alignments and the different databases (click on the '?'s to access PlasmoDB, FlyBase, and Amigo databases). Gene functional annotations are as follows. The short description immediately following each P. falciparum gene comes from PlasmoDB (red = functional gene, blue = putative gene, black = hypothetical gene). Other annotations are Gene Ontology annotations (red = Molecular Function, green = Biological Process, blue = Cellular Component). [file 1471-2164-11-35-S5.HTML]

# Bozdech - Pilot co-coexpression analysis

# 21 cluster pairs

## Cluster Pair #0: 5 gene pairs.

|  |  |  |  |  |  |  |  |  |  |  |  |  |  |  |  |  |  |
| --- | --- | --- | --- | --- | --- | --- | --- | --- | --- | --- | --- | --- | --- | --- | --- | --- | --- |
| P.falciparum D.melanogaster Blast evalue|  |  |  |  |  |  |  |  |  |  |  |  |  |  |  | | --- | --- | --- | --- | --- | --- | --- | --- | --- | --- | --- | --- | --- | --- | --- | | PFA0400c ? beta3 proteasome subunit, putative  endopeptidase activity ? threonine endopeptidase activity (IEA) ? proteasome core complex (sensu Eukaryota) ? ubiquitin-dependent protein catabolism ?  FBgn0026380 ? proteasome complex (sensu Eukaryota) ? endopeptidase activity ? threonine endopeptidase activity (IEA) ? proteasome core complex (sensu Eukaryota) ? ATP-dependent proteolysis ? ubiquitin-dependent protein catabolism (IEA) ?  8.96831e-44  BLAST| PF07\_0112 ? proteasome subunit alpha type 5, putative  endopeptidase activity ? threonine endopeptidase activity (IEA) ? proteasome core complex (sensu Eukaryota) (IEA) ? ubiquitin-dependent protein catabolism ? proteasome core complex, alpha-subunit complex (sensu Eukaryota) ?  FBgn0016697 ? proteasome complex (sensu Eukaryota) ? endopeptidase activity ? threonine endopeptidase activity (IEA) ? proteasome core complex (sensu Eukaryota) ? ATP-dependent proteolysis ? ubiquitin-dependent protein catabolism ?  0  BLAST| PFC0745c ? proteasome component C8, putative  endopeptidase activity ? threonine endopeptidase activity (IEA) ? proteasome core complex (sensu Eukaryota) ? ubiquitin-dependent protein catabolism ?  FBgn0023175 ? endopeptidase activity ? threonine endopeptidase activity (IEA) ? proteasome core complex (sensu Eukaryota) ? proteolysis and peptidolysis (IEA) ? ubiquitin-dependent protein catabolism (IEA) ?  5e-32  BLAST| MAL8P1.128 ? proteasome subunit alpha, putative  endopeptidase activity ? threonine endopeptidase activity (IEA) ? proteasome core complex (sensu Eukaryota) ? ubiquitin-dependent protein catabolism ?  FBgn0026781 ? endopeptidase activity ? threonine endopeptidase activity (IEA) ? proteasome core complex (sensu Eukaryota) ? proteolysis and peptidolysis (IEA) ? ubiquitin-dependent protein catabolism (IEA) ?  6.00036e-42  BLAST| PF13\_0282 ? proteasome subunit, putative  endopeptidase activity ? threonine endopeptidase activity (IEA) ? proteasome core complex (sensu Eukaryota) ? ubiquitin-dependent protein catabolism ?  FBgn0003150 ? proteasome complex (sensu Eukaryota) ? endopeptidase activity ? threonine endopeptidase activity (IEA) ? proteasome core complex (sensu Eukaryota) ? ATP-dependent proteolysis ? ubiquitin-dependent protein catabolism ?  0  BLAST | | | | | | | | | | | | | | | | | |

## Cluster Pair #1: 4 gene pairs.

|  |  |  |  |  |  |  |  |  |  |  |  |  |  |  |
| --- | --- | --- | --- | --- | --- | --- | --- | --- | --- | --- | --- | --- | --- | --- |
| P.falciparum D.melanogaster Blast evalue|  |  |  |  |  |  |  |  |  |  |  |  | | --- | --- | --- | --- | --- | --- | --- | --- | --- | --- | --- | --- | | MAL8P1.142 ? proteasome beta-subunit  endopeptidase activity ? threonine endopeptidase activity (IEA) ? proteasome core complex (sensu Eukaryota) ? ubiquitin-dependent protein catabolism (IEA) ?  FBgn0037314 ? endopeptidase activity ? threonine endopeptidase activity (IEA) ? nucleus ? cytoplasm ? proteasome core complex (sensu Eukaryota) ? ATP-dependent proteolysis ? ubiquitin-dependent protein catabolism ?  2e-26  BLAST| PFC0520w ? 26S proteasome regulatory subunit S14, putative  proteasome regulatory particle (sensu Eukaryota) ? ubiquitin-dependent protein catabolism ?  FBgn0028693 ? endopeptidase activity ? proteasome regulatory particle (sensu Eukaryota) ? proteolysis and peptidolysis ? proteasome regulatory particle, lid subcomplex (sensu Eukaryota) ?  6e-27  BLAST| PFE0915c ? proteasome subunit beta type 1  endopeptidase activity ? threonine endopeptidase activity (IEA) ? proteasome core complex (sensu Eukaryota) ? ubiquitin-dependent protein catabolism ?  FBgn0002284 ? proteasome complex (sensu Eukaryota) ? endopeptidase activity ? threonine endopeptidase activity (IEA) ? proteasome core complex (sensu Eukaryota) ? ATP-dependent proteolysis ? ubiquitin-dependent protein catabolism ?  5e-40  BLAST| PFI1545c ? proteasome precursor, putative  threonine endopeptidase activity (IEA) ? proteasome core complex (sensu Eukaryota) (IEA) ? ubiquitin-dependent protein catabolism (IEA) ?  FBgn0023174 ? endopeptidase activity ? threonine endopeptidase activity (IEA) ? proteasome core complex (sensu Eukaryota) ? proteolysis and peptidolysis (IEA) ? ubiquitin-dependent protein catabolism (IEA) ?  1e-08  BLAST | | | | | | | | | | | | | | |

## Cluster Pair #2: 4 gene pairs.

|  |  |  |  |  |  |  |  |  |  |  |  |  |  |  |
| --- | --- | --- | --- | --- | --- | --- | --- | --- | --- | --- | --- | --- | --- | --- |
| P.falciparum D.melanogaster Blast evalue|  |  |  |  |  |  |  |  |  |  |  |  | | --- | --- | --- | --- | --- | --- | --- | --- | --- | --- | --- | --- | | PF11\_0461 ? rab6  GTP binding ? Golgi apparatus ? intracellular protein transport ? small GTPase mediated signal transduction (IEA) ? protein transport (IEA) ?  FBgn0015797 ? compound eye morphogenesis (sensu Endopterygota) ? GTPase activity ? GTP binding (IEA) ? intracellular protein transport (IEA) ? receptor mediated endocytosis (IEA) ? signal transduction (IEA) ? small GTPase mediated signal transduction (IEA) ? regulation of exocytosis (IEA) ?  0  BLAST| PF10\_0081 ? 26S proteasome regulatory subunit 4, putative  nucleotide binding (IEA) ? endopeptidase activity ? ATP binding (IEA) ? nucleus (IEA) ? cytoplasm (IEA) ? proteasome regulatory particle (sensu Eukaryota) ? proteolysis and peptidolysis ? hydrolase activity (IEA) ? ATPase activity (IEA) ? nucleoside-triphosphatase activity (IEA) ? protein catabolism (IEA) ?  FBgn0015282 ? proteasome complex (sensu Eukaryota) ? endopeptidase activity ? ATP binding (IEA) ? nucleus (IEA) ? cytoplasm (IEA) ? proteasome regulatory particle (sensu Eukaryota) ? proteolysis and peptidolysis ? ATP-dependent proteolysis ? ubiquitin-dependent protein catabolism ? proteasome regulatory particle, base subcomplex (sensu Eukaryota) ? ATPase activity ?  0  BLAST| PF08\_0109 ? hypothetical protein   FBgn0015283 ? proteasome complex (sensu Eukaryota) ? endopeptidase activity ? proteasome regulatory particle (sensu Eukaryota) ? proteolysis and peptidolysis ? ubiquitin-dependent protein catabolism ? chromosome segregation ? proteasome regulatory particle, base subcomplex (sensu Eukaryota) ? protein metabolism (IEA) ?  3e-30  BLAST| MAL13P1.343 ? proteasome regulatory subunit, putative  proteasome regulatory particle (sensu Eukaryota) ? ubiquitin-dependent protein catabolism ?  FBgn0028694 ? endopeptidase activity ? proteasome regulatory particle (sensu Eukaryota) ? proteolysis and peptidolysis ? proteasome regulatory particle, lid subcomplex (sensu Eukaryota) ?  0  BLAST | | | | | | | | | | | | | | |

## Cluster Pair #3: 9 gene pairs.

|  |  |  |  |  |  |  |  |  |  |  |  |  |  |  |  |  |  |  |  |  |  |  |  |  |  |  |  |  |  |
| --- | --- | --- | --- | --- | --- | --- | --- | --- | --- | --- | --- | --- | --- | --- | --- | --- | --- | --- | --- | --- | --- | --- | --- | --- | --- | --- | --- | --- | --- |
| P.falciparum D.melanogaster Blast evalue|  |  |  |  |  |  |  |  |  |  |  |  |  |  |  |  |  |  |  |  |  |  |  |  |  |  |  | | --- | --- | --- | --- | --- | --- | --- | --- | --- | --- | --- | --- | --- | --- | --- | --- | --- | --- | --- | --- | --- | --- | --- | --- | --- | --- | --- | | PF10\_0327 ? Myb2 protein  DNA binding ? nucleus (IEA) ? regulation of transcription, DNA-dependent ?  FBgn0035136 ? regulation of progression through cell cycle (IEA) ? nuclear mRNA splicing, via spliceosome ? DNA binding (IEA) ? spliceosome complex ? regulation of transcription from RNA polymerase II promoter (IEA) ? transcription regulator activity (IEA) ? RNA splicing factor activity, transesterification mechanism ?  0  BLAST| PFE1530c ? hypothetical protein, conserved  nucleus (IEA) ?  FBgn0039557 ? nucleus (IEA) ?  5.00264e-43  BLAST| MAL7P1.81 ? eukaryotic translation initiation factor 3 37.28 kDa subunit, putative  translation initiation factor activity ? eukaryotic translation initiation factor 3 complex ? regulation of translational initiation ?  FBgn0015834 ? translation initiation factor activity ? cytosol ? eukaryotic translation initiation factor 3 complex ? protein biosynthesis ? translational initiation ?  0  BLAST| PF07\_0066 ? hypothetical protein  nucleic acid binding (IEA) ?  FBgn0005411 ? nucleotide binding (IEA) ? spliceosome assembly ? regulation of alternative nuclear mRNA splicing, via spliceosome ? nuclear mRNA splicing, via spliceosome ? mRNA binding ? nucleus ? spliceosome complex ? snRNP U2 ? poly-pyrimidine tract binding ? RNA splicing factor activity, transesterification mechanism ? protein heterodimerization activity ? nuclear export ?  2e-21  BLAST| PFL0815w ? DNA-binding chaperone, putative  DNA binding (IEA) ? nucleus (IEA) ? protein folding (IEA) ? heat shock protein binding (IEA) ? unfolded protein binding (IEA) ?  FBgn0037051 ? nucleic acid binding (IEA) ? DNA binding (IEA) ? nucleus (IEA) ? protein folding ? heat shock protein binding (IEA) ? regulation of transcription (IEA) ? unfolded protein binding ?  1e-19  BLAST| PF11\_0200 ? U2 snRNP auxiliary factor, small subunit, putative  nuclear mRNA splicing, via spliceosome ? nucleic acid binding (IEA) ? RNA binding ? nucleus (IEA) ? snRNP U2 ?  FBgn0017457 ? nucleotide binding (IEA) ? regulation of alternative nuclear mRNA splicing, via spliceosome ? nuclear mRNA splicing, via spliceosome ? mRNA binding ? nucleus ? spliceosome complex ? snRNP U2 ? poly-pyrimidine tract binding ? zinc ion binding (IEA) ?  0  BLAST| PFL1715w ? hypothetical protein  nucleus (IEA) ? nucleobase, nucleoside, nucleotide and nucleic acid metabolism (IEA) ? methyltransferase activity (IEA) ?  FBgn0032016 ? transcription factor activity ? nucleus ? nucleobase, nucleoside, nucleotide and nucleic acid metabolism (IEA) ? mRNA (2'-O-methyladenosine-N6-)-methyltransferase activity ?  0  BLAST| MAL13P1.132 ? microfibril-associated protein homologue, putative  extracellular matrix structural constituent ? extracellular region (IEA) ? extracellular matrix (sensu Metazoa) ?  FBgn0035294 ? microfibril ? structural molecule activity (IEA) ? cytoskeleton organization and biogenesis (IEA) ?  3e-18  BLAST| MAL13P1.261 ? hypothetical protein   FBgn0035249 ?  0.049  BLAST | | | | | | | | | | | | | | | | | | | | | | | | | | | | | |

## Cluster Pair #4: 15 gene pairs.

|  |  |  |  |  |  |  |  |  |  |  |  |  |  |  |  |  |  |  |  |  |  |  |  |  |  |  |  |  |  |  |  |  |  |  |  |  |  |  |  |  |  |  |  |  |  |  |  |
| --- | --- | --- | --- | --- | --- | --- | --- | --- | --- | --- | --- | --- | --- | --- | --- | --- | --- | --- | --- | --- | --- | --- | --- | --- | --- | --- | --- | --- | --- | --- | --- | --- | --- | --- | --- | --- | --- | --- | --- | --- | --- | --- | --- | --- | --- | --- | --- |
| P.falciparum D.melanogaster Blast evalue|  |  |  |  |  |  |  |  |  |  |  |  |  |  |  |  |  |  |  |  |  |  |  |  |  |  |  |  |  |  |  |  |  |  |  |  |  |  |  |  |  |  |  |  |  | | --- | --- | --- | --- | --- | --- | --- | --- | --- | --- | --- | --- | --- | --- | --- | --- | --- | --- | --- | --- | --- | --- | --- | --- | --- | --- | --- | --- | --- | --- | --- | --- | --- | --- | --- | --- | --- | --- | --- | --- | --- | --- | --- | --- | --- | | PFL2395c ? dimethyladenosine transferase, putative  rRNA modification ? rRNA (adenine-N6,N6-)-dimethyltransferase activity ? mitochondrion ? rRNA methyltransferase activity (IEA) ? S-adenosylmethionine-dependent methyltransferase activity (IEA) ? membrane ? apicoplast ?  FBgn0036197 ? rRNA modification (IEA) ? rRNA (adenine-N6,N6-)-dimethyltransferase activity (IEA) ? nucleic acid binding (IEA) ? mitochondrion ? rRNA processing (IEA) ? rRNA metabolism (IEA) ? rRNA (adenine) methyltransferase activity ?  3e-13  BLAST| PFL2445c ? hypothetical protein   FBgn0035411 ?  0.059  BLAST| PF14\_0576 ? ubiquitin carboxyl-terminal hydrolase, putative  ubiquitin thiolesterase activity ? intracellular (IEA) ? ubiquitin-dependent protein catabolism ?  FBgn0010288 ? ubiquitin thiolesterase activity ? intracellular (IEA) ? proteolysis and peptidolysis (IEA) ? ubiquitin-dependent protein catabolism (IEA) ? protein deubiquitination ?  1e-15  BLAST| PF14\_0378 ? triose-phosphate isomerase  triose-phosphate isomerase activity ? gluconeogenesis ? glycolysis ? pentose-phosphate shunt ? fatty acid biosynthesis ? metabolism (IEA) ?  FBgn0003738 ? triose-phosphate isomerase activity ? gluconeogenesis (IEA) ? glycolysis (IEA) ? pentose-phosphate shunt (IEA) ? intramolecular transferase activity (IEA) ?  0  BLAST| PFI1105w ? Phosphoglycerate kinase  phosphoglycerate kinase activity (IEA) ? glycolysis (IEA) ?  FBgn0003075 ? phosphoglycerate kinase activity ? cytoplasm ? glycolysis ? phosphorylation (IEA) ? carbohydrate kinase activity (IEA) ?  0  BLAST| PF14\_0370 ? RNA helicase, putative  nucleotide binding (IEA) ? nucleic acid binding (IEA) ? ATP-dependent RNA helicase activity ? helicase activity (IEA) ? ATP binding (IEA) ? ATP-dependent helicase activity (IEA) ? nucleoside-triphosphatase activity (IEA) ?  FBgn0038344 ? nuclear mRNA splicing, via spliceosome ? nucleic acid binding (IEA) ? RNA helicase activity ? helicase activity ? ATP binding (IEA) ? snRNP U5 ? ATP-dependent helicase activity (IEA) ? small nuclear ribonucleoprotein complex ?  0  BLAST| PF14\_0301 ? hypothetical protein  mitochondrion ?  FBgn0012066 ? nucleotide binding (IEA) ? nucleic acid binding (IEA) ? DNA binding (IEA) ? delta DNA polymerase activity ? phospholipase A2 activity (IEA) ? transporter activity (IEA) ? leading strand elongation ? lagging strand elongation ? DNA repair (IEA) ? DNA recombination (IEA) ? phospholipid metabolism (IEA) ? transport (IEA) ? 3'-5'-exodeoxyribonuclease activity ? single-stranded DNA specific 3'-5' exodeoxyribonuclease activity ? membrane (IEA) ? lipid catabolism (IEA) ?  0.039  BLAST| MAL13P1.345 ? hypothetical protein   FBgn0031848 ?  0.003  BLAST| PF14\_0598 ? glyceraldehyde-3-phosphate dehydrogenase  glyceraldehyde-3-phosphate dehydrogenase (phosphorylating) activity ? mitochondrion ? glucose metabolism (IEA) ? gluconeogenesis ? glycolysis ? glyceraldehyde-3-phosphate dehydrogenase activity (IEA) ? NAD binding (IEA) ? \*\* also with: FBgn0001091, clust.pair #4 FBgn0001092 ? glyceraldehyde-3-phosphate dehydrogenase (phosphorylating) activity ? cytoplasm ? glycolysis ? NAD binding (IEA) ?  0  BLAST| PF14\_0598 ? glyceraldehyde-3-phosphate dehydrogenase  glyceraldehyde-3-phosphate dehydrogenase (phosphorylating) activity ? mitochondrion ? glucose metabolism (IEA) ? gluconeogenesis ? glycolysis ? glyceraldehyde-3-phosphate dehydrogenase activity (IEA) ? NAD binding (IEA) ? \*\* also with: FBgn0001092, clust.pair #4 FBgn0001091 ? glyceraldehyde-3-phosphate dehydrogenase (phosphorylating) activity ? cytoplasm ? glycolysis ? NAD binding (IEA) ?  0  BLAST| PFL1545c ? chaperonin cpn60  protein binding (IEA) ? ATP binding (IEA) ? protein folding ? apicoplast ? cellular protein metabolism (IEA) ? unfolded protein binding ?  FBgn0015245 ? ATP binding (IEA) ? mitochondrion ? mitochondrial matrix ? protein folding ? 'de novo' protein folding ? protein targeting to mitochondrion ? response to stress ? response to heat ? protein refolding ? ATPase activity, coupled (IEA) ? unfolded protein binding ?  0  BLAST| PF13\_0232 ? Casein kinase II regulatory subunit, putative  protein kinase CK2 complex ? protein amino acid phosphorylation ? protein kinase CK2 regulator activity ?  FBgn0000259 ? protein kinase activity ? casein kinase activity ? protein kinase CK2 activity ? receptor signaling protein serine/threonine kinase activity (IEA) ? nucleus ? cytosol ? protein kinase CK2 complex ? protein amino acid phosphorylation ? signal transduction (IEA) ? rhythmic behavior ? circadian rhythm ? eclosion rhythm ? protein kinase CK2 regulator activity ? mushroom body development ?  7e-37  BLAST| PFA0500w ? human hepatopoietin-like protein, putative  cell proliferation ?  FBgn0031068 ? mitochondrion ? electron transport (IEA) ? oxidative phosphorylation (IEA) ? flavin-linked sulfhydryl oxidase activity ?  3e-14  BLAST| PFC0975c ? PFCYP19, cyclophilin, peptidyl-prolyl cis-trans isomerase  peptidyl-prolyl cis-trans isomerase activity ? cytosol ? protein folding ? cyclosporin A binding ?  FBgn0004432 ? peptidyl-prolyl cis-trans isomerase activity ? cytoplasm ? cytosol ? protein folding ? protein targeting (IEA) ? defense response (IEA) ? transcription elongation factor complex b ? cyclin-dependent protein kinase regulator activity ? nuclear cyclin-dependent protein kinase holoenzyme complex ? salivary gland cell death ? autophagic cell death ?  0  BLAST| PFL1710c ? tetQ family GTPase, putative  translation elongation factor activity ? GTP binding (IEA) ? protein biosynthesis (IEA) ? translational elongation ? apicoplast ?  FBgn0031898 ? translation elongation factor activity ? GTP binding (IEA) ? mitochondrion ? translational elongation ?  3e-23  BLAST | | | | | | | | | | | | | | | | | | | | | | | | | | | | | | | | | | | | | | | | | | | | | | | |

## Cluster Pair #5: 4 gene pairs.

|  |  |  |  |  |  |  |  |  |  |  |  |  |  |  |
| --- | --- | --- | --- | --- | --- | --- | --- | --- | --- | --- | --- | --- | --- | --- |
| P.falciparum D.melanogaster Blast evalue|  |  |  |  |  |  |  |  |  |  |  |  | | --- | --- | --- | --- | --- | --- | --- | --- | --- | --- | --- | --- | | PF13\_0334 ? polynucleotide kinase, putative  damaged DNA binding ? response to DNA damage stimulus ?  FBgn0037578 ? nucleobase, nucleoside, nucleotide and nucleic acid metabolism (IEA) ? DNA metabolism (IEA) ? DNA repair (IEA) ? kinase activity (IEA) ? nucleotide kinase activity (IEA) ?  2e-17  BLAST| PF11\_0078 ? hypothetical protein   FBgn0030354 ? mRNA catabolism, nonsense-mediated decay ? nucleic acid binding (IEA) ? RNA helicase activity (IEA) ? helicase activity ? cytoplasm ?  1e-14  BLAST| PFI0650c ? hypothetical protein   FBgn0033373 ?  0.002  BLAST| PF13\_0142 ? u6 snRNA-associated sm-like protein, putative  nucleus (IEA) ? spliceosome complex ? small nucleolar ribonucleoprotein complex (IEA) ? mRNA processing (IEA) ? ribonucleoprotein complex (IEA) ? RNA splicing factor activity, transesterification mechanism ?  FBgn0034564 ? nuclear mRNA splicing, via spliceosome ? spliceosome complex ? snRNP U6 ? small nuclear ribonucleoprotein complex ?  2e-20  BLAST | | | | | | | | | | | | | | |

## Cluster Pair #6: 2 gene pairs.

|  |  |  |  |  |  |  |  |  |
| --- | --- | --- | --- | --- | --- | --- | --- | --- |
| P.falciparum D.melanogaster Blast evalue|  |  |  |  |  |  | | --- | --- | --- | --- | --- | --- | | PFI1680w ? hypothetical protein   FBgn0034068 ? apoptosis (IEA) ? induction of apoptosis (IEA) ? signal transduction (IEA) ? intracellular signaling cascade (IEA) ?  4e-07  BLAST| PF14\_0632 ? 26S proteasome subunit, putative  regulation of progression through cell cycle (IEA) ? endopeptidase activity ? proteasome regulatory particle (sensu Eukaryota) ? ubiquitin-dependent protein catabolism ?  FBgn0028692 ? regulation of progression through cell cycle (IEA) ? endopeptidase activity ? binding (IEA) ? proteasome regulatory particle (sensu Eukaryota) ? proteolysis and peptidolysis ? proteasome regulatory particle, base subcomplex (sensu Eukaryota) ? enzyme regulator activity (IEA) ?  0  BLAST | | | | | | | | |

## Cluster Pair #7: 31 gene pairs.

|  |  |  |  |  |  |  |  |  |  |  |  |  |  |  |  |  |  |  |  |  |  |  |  |  |  |  |  |  |  |  |  |  |  |  |  |  |  |  |  |  |  |  |  |  |  |  |  |  |  |  |  |  |  |  |  |  |  |  |  |  |  |  |  |  |  |  |  |  |  |  |  |  |  |  |  |  |  |  |  |  |  |  |  |  |  |  |  |  |  |  |  |  |  |  |  |
| --- | --- | --- | --- | --- | --- | --- | --- | --- | --- | --- | --- | --- | --- | --- | --- | --- | --- | --- | --- | --- | --- | --- | --- | --- | --- | --- | --- | --- | --- | --- | --- | --- | --- | --- | --- | --- | --- | --- | --- | --- | --- | --- | --- | --- | --- | --- | --- | --- | --- | --- | --- | --- | --- | --- | --- | --- | --- | --- | --- | --- | --- | --- | --- | --- | --- | --- | --- | --- | --- | --- | --- | --- | --- | --- | --- | --- | --- | --- | --- | --- | --- | --- | --- | --- | --- | --- | --- | --- | --- | --- | --- | --- | --- | --- | --- |
| P.falciparum D.melanogaster Blast evalue|  |  |  |  |  |  |  |  |  |  |  |  |  |  |  |  |  |  |  |  |  |  |  |  |  |  |  |  |  |  |  |  |  |  |  |  |  |  |  |  |  |  |  |  |  |  |  |  |  |  |  |  |  |  |  |  |  |  |  |  |  |  |  |  |  |  |  |  |  |  |  |  |  |  |  |  |  |  |  |  |  |  |  |  |  |  |  |  |  |  |  |  |  | | --- | --- | --- | --- | --- | --- | --- | --- | --- | --- | --- | --- | --- | --- | --- | --- | --- | --- | --- | --- | --- | --- | --- | --- | --- | --- | --- | --- | --- | --- | --- | --- | --- | --- | --- | --- | --- | --- | --- | --- | --- | --- | --- | --- | --- | --- | --- | --- | --- | --- | --- | --- | --- | --- | --- | --- | --- | --- | --- | --- | --- | --- | --- | --- | --- | --- | --- | --- | --- | --- | --- | --- | --- | --- | --- | --- | --- | --- | --- | --- | --- | --- | --- | --- | --- | --- | --- | --- | --- | --- | --- | --- | --- | | PF13\_0014 ? 40S ribosomal protein S7 homologue, putative  structural constituent of ribosome ? intracellular (IEA) ? ribosome (IEA) ? cytosolic small ribosomal subunit (sensu Eukaryota) ? protein biosynthesis ?  FBgn0039757 ? nucleic acid binding (IEA) ? structural constituent of ribosome ? cytosolic small ribosomal subunit (sensu Eukaryota) ? protein biosynthesis ?  5e-31  BLAST| PF13\_0224 ? 60S ribosomal subunit protein L18, putative  structural constituent of ribosome ? intracellular (IEA) ? ribosome (IEA) ? cytosolic large ribosomal subunit (sensu Eukaryota) ? protein biosynthesis ?  FBgn0010409 ? nucleic acid binding (IEA) ? structural constituent of ribosome ? ribosome ? cytosolic large ribosomal subunit (sensu Eukaryota) ? protein biosynthesis ?  2e-32  BLAST| PF14\_0027 ? ribosomal S27a, putative  structural constituent of ribosome ? intracellular (IEA) ? ribosome (IEA) ? cytosolic small ribosomal subunit (sensu Eukaryota) ? protein biosynthesis (IEA) ? protein modification (IEA) ?  FBgn0003942 ? nucleic acid binding (IEA) ? structural constituent of ribosome ? protein binding ? nucleus ? cytoplasm ? ribosome ? cytosolic small ribosomal subunit (sensu Eukaryota) ? establishment and/or maintenance of chromatin architecture ? regulation of transcription, DNA-dependent ? protein biosynthesis ? protein modification ? ATP-dependent proteolysis ? ubiquitin-dependent protein catabolism ? ubiquitin cycle ? response to stress ?  2e-14  BLAST| PF10\_0038 ? ribosomal protein S20e, putative  structural constituent of ribosome ? intracellular (IEA) ? ribosome (IEA) ? cytosolic small ribosomal subunit (sensu Eukaryota) ? protein biosynthesis ? small ribosomal subunit (IEA) ?  FBgn0019936 ? nucleic acid binding (IEA) ? structural constituent of ribosome ? ribosome ? cytosolic small ribosomal subunit (sensu Eukaryota) ? protein biosynthesis ?  2e-35  BLAST| PF13\_0132 ? 60S ribosomal protein L23a, putative  structural constituent of ribosome ? cytosolic large ribosomal subunit (sensu Eukaryota) ? protein biosynthesis ? rRNA binding ?  FBgn0026372 ? nucleotide binding (IEA) ? nucleic acid binding (IEA) ? structural constituent of ribosome ? cytosolic large ribosomal subunit (sensu Eukaryota) ? protein biosynthesis ?  2e-24  BLAST| PFE0810c ? 40S ribosomal subunit protein S14, putative  RNA binding ? structural constituent of ribosome (IEA) ? intracellular (IEA) ? ribosome (IEA) ? cytosolic small ribosomal subunit (sensu Eukaryota) ? protein biosynthesis ? \*\* also with: FBgn0004404, clust.pair #7 FBgn0004403 ? nucleic acid binding (IEA) ? structural constituent of ribosome ? ribosome ? cytosolic small ribosomal subunit (sensu Eukaryota) ? protein biosynthesis ?  0  BLAST| PFE0810c ? 40S ribosomal subunit protein S14, putative  RNA binding ? structural constituent of ribosome (IEA) ? intracellular (IEA) ? ribosome (IEA) ? cytosolic small ribosomal subunit (sensu Eukaryota) ? protein biosynthesis ? \*\* also with: FBgn0004403, clust.pair #7 FBgn0004404 ? nucleic acid binding (IEA) ? structural constituent of ribosome ? ribosome ? cytosolic small ribosomal subunit (sensu Eukaryota) ? protein biosynthesis ?  0  BLAST| PFI0190w ? ribosomal protein L32, putative  structural constituent of ribosome (IEA) ? intracellular (IEA) ? ribosome (IEA) ? protein biosynthesis (IEA) ?  FBgn0002626 ? nucleic acid binding (IEA) ? structural constituent of ribosome ? ribosome ? cytosolic large ribosomal subunit (sensu Eukaryota) ? protein biosynthesis ?  4e-28  BLAST| PF13\_0213 ? 60S ribosomal subunit protein L6e, putative  structural constituent of ribosome ? intracellular (IEA) ? ribosome (IEA) ? cytosolic large ribosomal subunit (sensu Eukaryota) ? protein biosynthesis ?  FBgn0039857 ? nucleic acid binding (IEA) ? structural constituent of ribosome (IEA) ? cytosolic large ribosomal subunit (sensu Eukaryota) ? protein biosynthesis (IEA) ?  1e-15  BLAST| PFB0830w ? Ribosomal protein S26e, putative  structural constituent of ribosome ? intracellular (IEA) ? mitochondrion ? ribosome (IEA) ? cytosolic small ribosomal subunit (sensu Eukaryota) ? protein biosynthesis ?  FBgn0004413 ? nucleic acid binding (IEA) ? structural constituent of ribosome ? ribosome ? cytosolic small ribosomal subunit (sensu Eukaryota) ? protein biosynthesis ?  1e-31  BLAST| PF07\_0080 ? 40S ribosomal protein S10, putative  structural constituent of ribosome ? cytosolic small ribosomal subunit (sensu Eukaryota) ? protein biosynthesis ?  FBgn0031035 ? nucleic acid binding (IEA) ? structural constituent of ribosome ? actin binding (IEA) ? structural constituent of cytoskeleton (IEA) ? cytosolic small ribosomal subunit (sensu Eukaryota) ? protein biosynthesis (IEA) ? cytoskeleton organization and biogenesis (IEA) ? microtubule binding (IEA) ? intermediate filament binding (IEA) ?  2e-25  BLAST| PFC0295c ? 40S ribosomal protein S12, putative  structural constituent of ribosome ? intracellular (IEA) ? ribosome (IEA) ? cytosolic small ribosomal subunit (sensu Eukaryota) ? protein biosynthesis ?  FBgn0014027 ? structural constituent of ribosome ? ribosome ? cytosolic small ribosomal subunit (sensu Eukaryota) ? protein biosynthesis ?  2e-22  BLAST| PFC0300c ? 60S ribosomal protein L7, putative  structural constituent of ribosome ? intracellular (IEA) ? ribosome (IEA) ? cytosolic large ribosomal subunit (sensu Eukaryota) ? protein biosynthesis ? large ribosomal subunit (IEA) ? transcription regulator activity (IEA) ?  FBgn0005593 ? mRNA binding ? structural constituent of ribosome ? cysteine-type endopeptidase activity (IEA) ? ribosome ? cytosolic large ribosomal subunit (sensu Eukaryota) ? protein biosynthesis ? proteolysis and peptidolysis (IEA) ? rRNA binding ? transcription regulator activity (IEA) ?  4e-39  BLAST| PF13\_0045 ? 40S ribosomal protein S27, putative  structural constituent of ribosome ? intracellular (IEA) ? ribosome (IEA) ? cytosolic small ribosomal subunit (sensu Eukaryota) ? protein biosynthesis ?  FBgn0039300 ? nucleic acid binding (IEA) ? structural constituent of ribosome ? ribosome (IEA) ? cytosolic small ribosomal subunit (sensu Eukaryota) ? protein biosynthesis (IEA) ?  7e-31  BLAST| PF13\_0171 ? 60S ribosomal protein L23, putative  structural constituent of ribosome (IEA) ? intracellular (IEA) ? ribosome (IEA) ? protein biosynthesis (IEA) ?  FBgn0010078 ? nucleic acid binding (IEA) ? structural constituent of ribosome ? ribosome ? cytosolic large ribosomal subunit (sensu Eukaryota) ? protein biosynthesis ?  0  BLAST| PF14\_0231 ? ribosomal protein L7a, putative  structural constituent of ribosome ? intracellular (IEA) ? ribosome (IEA) ? cytosolic large ribosomal subunit (sensu Eukaryota) ? protein biosynthesis ? ribonucleoprotein complex (IEA) ? ribosome biogenesis and assembly (IEA) ?  FBgn0014026 ? nucleic acid binding (IEA) ? structural constituent of ribosome ? ribosome ? cytosolic large ribosomal subunit (sensu Eukaryota) ? protein biosynthesis ? ribosome biogenesis and assembly (IEA) ?  0  BLAST| PF11\_0272 ? ribosomal protein S18, putative  RNA binding (IEA) ? structural constituent of ribosome ? intracellular (IEA) ? ribosome (IEA) ? protein biosynthesis ? small ribosomal subunit ?  FBgn0010411 ? nucleic acid binding (IEA) ? structural constituent of ribosome ? ribosome ? cytosolic small ribosomal subunit (sensu Eukaryota) ? protein biosynthesis ? translational initiation ?  0  BLAST| PF14\_0579 ? ribosomal protein L27, putative  structural constituent of ribosome ? intracellular (IEA) ? ribosome (IEA) ? cytosolic large ribosomal subunit (sensu Eukaryota) ? protein biosynthesis ?  FBgn0039359 ? nucleic acid binding (IEA) ? structural constituent of ribosome ? cytosolic large ribosomal subunit (sensu Eukaryota) ? protein biosynthesis ?  5e-18  BLAST| PF08\_0076 ? 40S ribosomal protein S16, putative  structural constituent of ribosome ? intracellular (IEA) ? ribosome (IEA) ? cytosolic small ribosomal subunit (sensu Eukaryota) ? protein biosynthesis ?  FBgn0034743 ? nucleic acid binding (IEA) ? structural constituent of ribosome ? cytosolic small ribosomal subunit (sensu Eukaryota) ? protein biosynthesis ?  8.40779e-45  BLAST| PF14\_0240 ? ribosomal protein L21e, putative  structural constituent of ribosome ? mitochondrion ? cytosolic large ribosomal subunit (sensu Eukaryota) ? protein biosynthesis ?  FBgn0032987 ? nucleic acid binding (IEA) ? structural constituent of ribosome (IEA) ? cytosolic large ribosomal subunit (sensu Eukaryota) ? protein biosynthesis ?  8e-33  BLAST| PF14\_0655 ? RNA helicase-1, putative  RNA cap binding ? nucleic acid binding (IEA) ? mRNA binding ? translation initiation factor activity ? helicase activity (IEA) ? ATP binding (IEA) ? regulation of translational initiation ? ATP-dependent helicase activity ? eukaryotic translation initiation factor 4F complex ? \*\* also with: FBgn0037573, clust.pair #7 FBgn0001942 ? RNA cap binding ? regulation of alternative nuclear mRNA splicing, via spliceosome ? larval development (sensu Insecta) ? RNA helicase activity ? mRNA binding ? translation initiation factor activity ? ATP binding (IEA) ? nucleus ? cytosol ? DNA unwinding ? translational initiation ? imaginal disc growth ? dorsal/ventral axis specification ? eukaryotic translation initiation factor 4F complex ? single-stranded DNA-dependent ATP-dependent DNA helicase activity ?  0  BLAST| PF14\_0655 ? RNA helicase-1, putative  RNA cap binding ? nucleic acid binding (IEA) ? mRNA binding ? translation initiation factor activity ? helicase activity (IEA) ? ATP binding (IEA) ? regulation of translational initiation ? ATP-dependent helicase activity ? eukaryotic translation initiation factor 4F complex ? \*\* also with: FBgn0001942, clust.pair #7 FBgn0037573 ? translation initiation factor activity ? ATP-dependent RNA helicase activity ? ATP binding (IEA) ? nucleobase, nucleoside, nucleotide and nucleic acid metabolism (IEA) ? translational initiation ? eukaryotic translation initiation factor 4F complex ?  0  BLAST| PFL2055w ? 40S ribosomal protein S17, putative  structural constituent of ribosome ? intracellular (IEA) ? mitochondrion ? ribosome (IEA) ? cytosolic small ribosomal subunit (sensu Eukaryota) ? protein biosynthesis ?  FBgn0005533 ? nucleic acid binding (IEA) ? structural constituent of ribosome ? ribosome ? cytosolic small ribosomal subunit (sensu Eukaryota) ? protein biosynthesis ?  4e-30  BLAST| PFC0441c ? hypothetical protein   FBgn0034654 ? translation initiation factor activity ? nucleus (IEA) ? eukaryotic translation initiation factor 3 complex ? translational initiation ?  2e-10  BLAST| PF11\_0438 ? Ribosomal protein, putative  structural constituent of ribosome ? intracellular (IEA) ? ribosome (IEA) ? protein biosynthesis ? large ribosomal subunit ?  FBgn0037328 ? nucleic acid binding (IEA) ? structural constituent of ribosome ? cytosolic large ribosomal subunit (sensu Eukaryota) ? protein biosynthesis ?  1e-22  BLAST| MAL13P1.92 ? 40S ribosomal protein S15, putative  structural constituent of ribosome ? intracellular (IEA) ? ribosome (IEA) ? cytosolic small ribosomal subunit (sensu Eukaryota) ? protein biosynthesis ? small ribosomal subunit (IEA) ?  FBgn0034138 ? nucleic acid binding (IEA) ? structural constituent of ribosome ? cytosolic small ribosomal subunit (sensu Eukaryota) ? protein biosynthesis ?  9.94922e-44  BLAST| PF13\_0228 ? 40S ribosomal subunit protein S6, putative  structural constituent of ribosome ? intracellular (IEA) ? ribosome (IEA) ? cytosolic small ribosomal subunit (sensu Eukaryota) ? protein biosynthesis ?  FBgn0004922 ? nucleic acid binding (IEA) ? structural constituent of ribosome ? ribosome ? cytosolic small ribosomal subunit (sensu Eukaryota) ? protein biosynthesis ? immune response ?  0  BLAST| PFC0535w ? 60S ribosomal protein L26, putative  structural constituent of ribosome ? intracellular (IEA) ? ribosome (IEA) ? cytosolic large ribosomal subunit (sensu Eukaryota) ? protein biosynthesis ? large ribosomal subunit (IEA) ?  FBgn0036825 ? nucleic acid binding (IEA) ? structural constituent of ribosome ? cytosolic large ribosomal subunit (sensu Eukaryota) ? protein biosynthesis ?  5e-30  BLAST| PF13\_0268 ? ribosomal protein L17, putative  structural constituent of ribosome ? intracellular (IEA) ? ribosome (IEA) ? cytosolic large ribosomal subunit (sensu Eukaryota) ? protein biosynthesis ? large ribosomal subunit (IEA) ?  FBgn0029897 ? nucleic acid binding (IEA) ? structural constituent of ribosome ? cytosolic large ribosomal subunit (sensu Eukaryota) ? protein biosynthesis ?  0  BLAST| PFC0735w ? 40S ribosomal protein S15A, putative  structural constituent of ribosome ? intracellular (IEA) ? ribosome (IEA) ? cytosolic small ribosomal subunit (sensu Eukaryota) ? protein biosynthesis ?  FBgn0033555 ? nucleic acid binding (IEA) ? structural constituent of ribosome ? cytosolic small ribosomal subunit (sensu Eukaryota) ? protein biosynthesis (IEA) ?  0  BLAST| PFC0775w ? 40S ribosomal protein S11, putative  nucleic acid binding (IEA) ? structural constituent of ribosome ? intracellular (IEA) ? ribosome (IEA) ? cytosolic small ribosomal subunit (sensu Eukaryota) ? protein biosynthesis ?  FBgn0033699 ? nucleic acid binding (IEA) ? structural constituent of ribosome ? cytosolic small ribosomal subunit (sensu Eukaryota) ? protein biosynthesis ?  1.4013e-45  BLAST | | | | | | | | | | | | | | | | | | | | | | | | | | | | | | | | | | | | | | | | | | | | | | | | | | | | | | | | | | | | | | | | | | | | | | | | | | | | | | | | | | | | | | | | | | | | | | | |

## Cluster Pair #8: 11 gene pairs.

|  |  |  |  |  |  |  |  |  |  |  |  |  |  |  |  |  |  |  |  |  |  |  |  |  |  |  |  |  |  |  |  |  |  |  |  |
| --- | --- | --- | --- | --- | --- | --- | --- | --- | --- | --- | --- | --- | --- | --- | --- | --- | --- | --- | --- | --- | --- | --- | --- | --- | --- | --- | --- | --- | --- | --- | --- | --- | --- | --- | --- |
| P.falciparum D.melanogaster Blast evalue|  |  |  |  |  |  |  |  |  |  |  |  |  |  |  |  |  |  |  |  |  |  |  |  |  |  |  |  |  |  |  |  |  | | --- | --- | --- | --- | --- | --- | --- | --- | --- | --- | --- | --- | --- | --- | --- | --- | --- | --- | --- | --- | --- | --- | --- | --- | --- | --- | --- | --- | --- | --- | --- | --- | --- | | PF14\_0661 ? hypothetical protein, conserved  nucleic acid binding (IEA) ?  FBgn0020305 ? mRNA binding (IEA) ? nucleoplasm ? nucleolus ? rRNA processing ?  0.0005  BLAST| PF13\_0177 ? ATP-dependent RNA helicase, putative  nucleic acid binding (IEA) ? helicase activity (IEA) ? ATP binding (IEA) ? ATP-dependent helicase activity (IEA) ?  FBgn0032919 ? nucleic acid binding (IEA) ? ATP-dependent RNA helicase activity ? ATP binding (IEA) ? nucleobase, nucleoside, nucleotide and nucleic acid metabolism (IEA) ? metal ion transport (IEA) ? metal ion binding (IEA) ?  0  BLAST| PFB0370c ? RNA-binding protein, putative   FBgn0020305 ? mRNA binding (IEA) ? nucleoplasm ? nucleolus ? rRNA processing ?  0  BLAST| PF07\_0092 ? hypothetical protein, conserved   FBgn0030000 ?  0  BLAST| PFB0860c ? RNA helicase, putative  nucleic acid binding (IEA) ? ATP-dependent RNA helicase activity ? helicase activity (IEA) ? ATP binding (IEA) ? ATP-dependent helicase activity (IEA) ?  FBgn0032919 ? nucleic acid binding (IEA) ? ATP-dependent RNA helicase activity ? ATP binding (IEA) ? nucleobase, nucleoside, nucleotide and nucleic acid metabolism (IEA) ? metal ion transport (IEA) ? metal ion binding (IEA) ?  0  BLAST| PF14\_0124 ? actin II  microfilament motor activity ? structural constituent of cytoskeleton ? protein binding (IEA) ? actin filament ? cytoskeleton organization and biogenesis ? \*\* also with: FBgn0000043, clust.pair #9 FBgn0000042 ? cytokinesis ? structural constituent of cytoskeleton ? protein binding (IEA) ? ATP binding (IEA) ? actin filament ? cytoskeleton organization and biogenesis ? sperm individualization ?  0  BLAST| PF14\_0113 ? hypothetical protein  RNA binding (IEA) ?  FBgn0032678 ?  0.007  BLAST| PF13\_0341 ? DNA-directed RNA polymerase 2, putative  DNA binding ? DNA-directed RNA polymerase activity ? DNA-directed RNA polymerase II, core complex ? transcription (IEA) ? transcription from RNA polymerase II promoter ?  FBgn0033571 ? nucleic acid binding (IEA) ? DNA binding (IEA) ? DNA-directed RNA polymerase activity ? DNA-directed RNA polymerase II, core complex ? transcription from RNA polymerase II promoter ? mRNA transcription (IEA) ?  0  BLAST| PFI1215w ? splicesome-associated protein, putative  nucleic acid binding (IEA) ? nucleus (IEA) ?  FBgn0014366 ? nuclear mRNA splicing, via spliceosome ? nucleic acid binding (IEA) ? spliceosome complex ? snRNP U2 ? zinc ion binding (IEA) ? fertilization ?  1.96182e-44  BLAST| PFE1115c ? s-adenosylmethionine-dependent methyltransferase, putative  S-adenosylmethionine-dependent methyltransferase activity ?  FBgn0037543 ? nucleobase, nucleoside, nucleotide and nucleic acid metabolism (IEA) ? S-adenosylmethionine-dependent methyltransferase activity ?  0  BLAST| PF11\_0191 ? hypothetical protein   FBgn0038964 ? mRNA binding (IEA) ? nucleolus ? rRNA metabolism (IEA) ? small nuclear ribonucleoprotein complex ?  0  BLAST | | | | | | | | | | | | | | | | | | | | | | | | | | | | | | | | | | | |

## Cluster Pair #9: 12 gene pairs.

|  |  |  |  |  |  |  |  |  |  |  |  |  |  |  |  |  |  |  |  |  |  |  |  |  |  |  |  |  |  |  |  |  |  |  |  |  |  |  |
| --- | --- | --- | --- | --- | --- | --- | --- | --- | --- | --- | --- | --- | --- | --- | --- | --- | --- | --- | --- | --- | --- | --- | --- | --- | --- | --- | --- | --- | --- | --- | --- | --- | --- | --- | --- | --- | --- | --- |
| P.falciparum D.melanogaster Blast evalue|  |  |  |  |  |  |  |  |  |  |  |  |  |  |  |  |  |  |  |  |  |  |  |  |  |  |  |  |  |  |  |  |  |  |  |  | | --- | --- | --- | --- | --- | --- | --- | --- | --- | --- | --- | --- | --- | --- | --- | --- | --- | --- | --- | --- | --- | --- | --- | --- | --- | --- | --- | --- | --- | --- | --- | --- | --- | --- | --- | --- | | PF11\_0250 ? high mobility group-like protein NHP2, putative  structural constituent of ribosome (IEA) ? intracellular (IEA) ? nucleus ? ribosome (IEA) ? protein biosynthesis (IEA) ? ribonucleoprotein complex (IEA) ? ribosome biogenesis and assembly (IEA) ?  FBgn0014026 ? nucleic acid binding (IEA) ? structural constituent of ribosome ? ribosome ? cytosolic large ribosomal subunit (sensu Eukaryota) ? protein biosynthesis ? ribosome biogenesis and assembly (IEA) ?  1e-05  BLAST| PFI0860c ? ATP-dependant RNA helicase, putative  nucleic acid binding (IEA) ? helicase activity (IEA) ? ATP binding (IEA) ? ATP-dependent helicase activity (IEA) ?  FBgn0033160 ? nuclear mRNA splicing, via spliceosome ? RNA helicase activity ? structural constituent of ribosome (IEA) ? ATP-dependent RNA helicase activity ? ATP binding (IEA) ? spliceosome complex ? ribosome (IEA) ? protein biosynthesis (IEA) ? ATP-dependent helicase activity ? RNA splicing factor activity, transesterification mechanism ?  0  BLAST| PF14\_0584 ? ribosomal protein S4, putative  RNA binding (IEA) ? structural constituent of ribosome ? mitochondrial small ribosomal subunit ? protein biosynthesis ?  FBgn0034232 ? structural constituent of ribosome ? mitochondrial ribosome ? protein biosynthesis ?  3e-31  BLAST| PF14\_0274 ? hypothetical protein, conserved   FBgn0038272 ? defense response (IEA) ? peptidyl-diphthamide biosynthesis from peptidyl-histidine ?  2e-14  BLAST| PF07\_0027 ? DNA-directed RNA polymerase 2 8.2 kDa polypeptide, putative  DNA-directed RNA polymerase activity ? DNA-directed RNA polymerase II, core complex ? transcription (IEA) ? transcription from RNA polymerase II promoter ?  FBgn0039218 ? nucleic acid binding (IEA) ? DNA binding (IEA) ? DNA-directed RNA polymerase activity ? nucleus ? DNA-directed RNA polymerase II, core complex ? transcription ? transcription from RNA polymerase II promoter ? regulation of transcription (IEA) ?  1e-16  BLAST| PF14\_0124 ? actin II  microfilament motor activity ? structural constituent of cytoskeleton ? protein binding (IEA) ? actin filament ? cytoskeleton organization and biogenesis ? \*\* also with: FBgn0000042, clust.pair #8 FBgn0000043 ? cytokinesis ? structural constituent of cytoskeleton ? protein binding (IEA) ? ATP binding (IEA) ? actin filament ? cytoskeleton organization and biogenesis ?  0  BLAST| PF10\_0068 ? hypothetical protein  nucleic acid binding (IEA) ?  FBgn0037701 ? nucleotide binding (IEA) ? mRNA binding ?  3e-11  BLAST| PF14\_0068 ? fibrillarin, putative  RNA binding (IEA) ? nucleus (IEA) ? small nucleolar ribonucleoprotein complex ? mitochondrion ? rRNA processing ?  FBgn0003062 ? dense fibrillar component ? mRNA binding ? small nucleolar ribonucleoprotein complex ? 35S primary transcript processing ? RNA processing ? rRNA metabolism (IEA) ? small nuclear ribonucleoprotein complex ?  0  BLAST| PF14\_0635 ? hypothetical protein, conserved  RNA binding (IEA) ?  FBgn0039233 ?  4e-32  BLAST| PFL2310w ? hypothetical protein, conserved  nucleic acid binding (IEA) ?  FBgn0037220 ? nucleotide binding (IEA) ? regulation of alternative nuclear mRNA splicing, via spliceosome ? mRNA binding ? nucleus ? zinc ion binding (IEA) ?  0  BLAST| PF14\_0221 ? hypothetical protein, conserved  GTP binding (IEA) ?  FBgn0034243 ? receptor binding (IEA) ? GTP binding ? nucleus ? intracellular protein transport (IEA) ? cell surface receptor linked signal transduction (IEA) ? intracellular signaling cascade (IEA) ?  0  BLAST| PF14\_0174 ? hypothetical protein, conserved  RNA binding (IEA) ? pseudouridylate synthase activity (IEA) ? RNA processing (IEA) ?  FBgn0023184 ? pseudouridine synthesis ? pseudouridylate synthase activity ? nucleus ? nucleolus ? rRNA processing ? chromosome segregation (IEA) ? mitosis (IEA) ? germ cell development ? rRNA metabolism (IEA) ? centromeric DNA binding (IEA) ?  0  BLAST | | | | | | | | | | | | | | | | | | | | | | | | | | | | | | | | | | | | | | |

## Cluster Pair #10: 12 gene pairs.

|  |  |  |  |  |  |  |  |  |  |  |  |  |  |  |  |  |  |  |  |  |  |  |  |  |  |  |  |  |  |  |  |  |  |  |  |  |  |  |
| --- | --- | --- | --- | --- | --- | --- | --- | --- | --- | --- | --- | --- | --- | --- | --- | --- | --- | --- | --- | --- | --- | --- | --- | --- | --- | --- | --- | --- | --- | --- | --- | --- | --- | --- | --- | --- | --- | --- |
| P.falciparum D.melanogaster Blast evalue|  |  |  |  |  |  |  |  |  |  |  |  |  |  |  |  |  |  |  |  |  |  |  |  |  |  |  |  |  |  |  |  |  |  |  |  | | --- | --- | --- | --- | --- | --- | --- | --- | --- | --- | --- | --- | --- | --- | --- | --- | --- | --- | --- | --- | --- | --- | --- | --- | --- | --- | --- | --- | --- | --- | --- | --- | --- | --- | --- | --- | | PFL2295w ? hypothetical protein  rRNA processing (IEA) ? ribonucleoprotein complex (IEA) ?  FBgn0030063 ? small nucleolar ribonucleoprotein complex ? rRNA processing ? protein biosynthesis ?  7e-05  BLAST| PF07\_0122 ? hypothetical protein, conserved   FBgn0035524 ? nucleolus ? ribosomal large subunit biogenesis ?  7.00005e-41  BLAST| PF14\_0150 ? RNA polymerase small subunit, putative  DNA binding (IEA) ? DNA-directed RNA polymerase activity ? transcription ? protein dimerization activity (IEA) ?  FBgn0032762 ? nucleic acid binding (IEA) ? DNA binding (IEA) ? DNA-directed RNA polymerase activity ? DNA-directed RNA polymerase I complex ? transcription from RNA polymerase I promoter ? transcription from RNA polymerase II promoter (IEA) ? mRNA transcription (IEA) ? protein dimerization activity (IEA) ?  4e-16  BLAST| PFI1085w ? ubiquitin-like protein, putative  protein modification (IEA) ?  FBgn0003942 ? nucleic acid binding (IEA) ? structural constituent of ribosome ? protein binding ? nucleus ? cytoplasm ? ribosome ? cytosolic small ribosomal subunit (sensu Eukaryota) ? establishment and/or maintenance of chromatin architecture ? regulation of transcription, DNA-dependent ? protein biosynthesis ? protein modification ? ATP-dependent proteolysis ? ubiquitin-dependent protein catabolism ? ubiquitin cycle ? response to stress ?  0.002  BLAST| PF10\_0277 ? hypothetical protein, conserved   FBgn0039828 ? nucleolus ? processing of 27S pre-rRNA ?  8e-06  BLAST| PF10\_0278 ? hypothetical protein, conserved   FBgn0038585 ?  1e-09  BLAST| MAL13P1.341 ? hypothetical protein, conserved   FBgn0033485 ? nucleic acid binding (IEA) ? structural constituent of ribosome (IEA) ? protein biosynthesis (IEA) ? protein metabolism (IEA) ? ribosome biogenesis and assembly (IEA) ?  8e-17  BLAST| PFB0865w ? small nuclear ribonucleoprotein, putative  nucleus (IEA) ? spliceosome complex ? small nucleolar ribonucleoprotein complex (IEA) ? mRNA processing (IEA) ? RNA splicing ? ribonucleoprotein complex (IEA) ? RNA splicing factor activity, transesterification mechanism ?  FBgn0037434 ? nuclear mRNA splicing, via spliceosome ? spliceosome complex ? small nuclear ribonucleoprotein complex ? RNA splicing factor activity, transesterification mechanism ?  9e-25  BLAST| PF13\_0310 ? hypothetical protein   FBgn0029755 ?  0.0003  BLAST| PFI1070c ? hypothetical protein   FBgn0032408 ? nucleic acid binding (IEA) ? mRNA binding (IEA) ? nucleobase, nucleoside, nucleotide and nucleic acid metabolism (IEA) ? rRNA metabolism (IEA) ?  6e-31  BLAST| PFE1435c ? hypothetical protein   FBgn0038473 ? receptor binding (IEA) ? hormone activity (IEA) ? GTP binding (IEA) ? intracellular (IEA) ? nucleobase, nucleoside, nucleotide and nucleic acid metabolism (IEA) ? transport (IEA) ? intracellular protein transport (IEA) ? signal transduction (IEA) ? cell surface receptor linked signal transduction (IEA) ? intracellular signaling cascade (IEA) ? nucleobase, nucleoside, nucleotide and nucleic acid transport (IEA) ? protein metabolism (IEA) ?  0.0002  BLAST| PF14\_0194 ? spliceosome-associated protein, putative  nucleic acid binding (IEA) ? spliceosome complex ? RNA splicing ? RNA splicing factor activity, transesterification mechanism ?  FBgn0015818 ? nucleotide binding (IEA) ? nuclear mRNA splicing, via spliceosome ? mRNA binding ? spliceosome complex ? snRNP U2 ? histone mRNA 3'-end processing (IEA) ? RNA splicing factor activity, transesterification mechanism ?  0  BLAST | | | | | | | | | | | | | | | | | | | | | | | | | | | | | | | | | | | | | | |

## Cluster Pair #11: 2 gene pairs.

|  |  |  |  |  |  |  |  |  |
| --- | --- | --- | --- | --- | --- | --- | --- | --- |
| P.falciparum D.melanogaster Blast evalue|  |  |  |  |  |  | | --- | --- | --- | --- | --- | --- | | PF13\_0318 ? hypothetical protein, conserved  nucleic acid binding (IEA) ?  FBgn0037342 ? nucleotide binding (IEA) ? mRNA binding ?  1e-31  BLAST| PF11\_0380 ? hypothetical protein, conserved  protein folding (IEA) ? membrane ? heat shock protein binding (IEA) ? unfolded protein binding (IEA) ?  FBgn0038195 ? protein folding (IEA) ? response to stress (IEA) ? defense response (IEA) ? protein metabolism (IEA) ? heat shock protein binding (IEA) ? unfolded protein binding (IEA) ?  3e-17  BLAST | | | | | | | | |

## Cluster Pair #12: 8 gene pairs.

|  |  |  |  |  |  |  |  |  |  |  |  |  |  |  |  |  |  |  |  |  |  |  |  |  |  |  |
| --- | --- | --- | --- | --- | --- | --- | --- | --- | --- | --- | --- | --- | --- | --- | --- | --- | --- | --- | --- | --- | --- | --- | --- | --- | --- | --- |
| P.falciparum D.melanogaster Blast evalue|  |  |  |  |  |  |  |  |  |  |  |  |  |  |  |  |  |  |  |  |  |  |  |  | | --- | --- | --- | --- | --- | --- | --- | --- | --- | --- | --- | --- | --- | --- | --- | --- | --- | --- | --- | --- | --- | --- | --- | --- | | PF14\_0563 ? DEAD-box RNA helicase, putative  nucleic acid binding (IEA) ? ATP-dependent RNA helicase activity ? helicase activity (IEA) ? ATP binding (IEA) ? ATP-dependent helicase activity (IEA) ? \*\* also with: FBgn0001942, clust.pair #12 FBgn0037573 ? translation initiation factor activity ? ATP-dependent RNA helicase activity ? ATP binding (IEA) ? nucleobase, nucleoside, nucleotide and nucleic acid metabolism (IEA) ? translational initiation ? eukaryotic translation initiation factor 4F complex ?  0  BLAST| PF14\_0563 ? DEAD-box RNA helicase, putative  nucleic acid binding (IEA) ? ATP-dependent RNA helicase activity ? helicase activity (IEA) ? ATP binding (IEA) ? ATP-dependent helicase activity (IEA) ? \*\* also with: FBgn0037573, clust.pair #12 FBgn0001942 ? RNA cap binding ? regulation of alternative nuclear mRNA splicing, via spliceosome ? larval development (sensu Insecta) ? RNA helicase activity ? mRNA binding ? translation initiation factor activity ? ATP binding (IEA) ? nucleus ? cytosol ? DNA unwinding ? translational initiation ? imaginal disc growth ? dorsal/ventral axis specification ? eukaryotic translation initiation factor 4F complex ? single-stranded DNA-dependent ATP-dependent DNA helicase activity ?  0  BLAST| PFL1975c ? hypothetical protein, conserved   FBgn0034274 ? cell proliferation (IEA) ?  5e-11  BLAST| PF14\_0542 ? hypothetical protein, conserved   FBgn0035415 ?  3e-06  BLAST| PF14\_0587 ? hypothetical protein  nucleus (IEA) ? mRNA processing (IEA) ?  FBgn0031493 ? nuclear mRNA splicing, via spliceosome ? spliceosome complex ? snRNP U2 ?  0  BLAST| PFI0930c ? Nucleosome assembly protein  nucleus (IEA) ? nucleosome assembly (IEA) ?  FBgn0014879 ? nucleus ? DNA replication (IEA) ? nucleosome assembly (IEA) ? spermatogenesis (IEA) ? phosphatase inhibitor activity (IEA) ? cyclin binding ?  6e-23  BLAST| MAL13P1.172 ? hypothetical protein, conserved   FBgn0039136 ?  3e-25  BLAST| PFI1435w ? RNA binding function, putative  nucleic acid binding (IEA) ?  FBgn0037701 ? nucleotide binding (IEA) ? mRNA binding ?  0.003  BLAST | | | | | | | | | | | | | | | | | | | | | | | | | | |

## Cluster Pair #13: 5 gene pairs.

|  |  |  |  |  |  |  |  |  |  |  |  |  |  |  |  |  |  |
| --- | --- | --- | --- | --- | --- | --- | --- | --- | --- | --- | --- | --- | --- | --- | --- | --- | --- |
| P.falciparum D.melanogaster Blast evalue|  |  |  |  |  |  |  |  |  |  |  |  |  |  |  | | --- | --- | --- | --- | --- | --- | --- | --- | --- | --- | --- | --- | --- | --- | --- | | PFB0220w ? UbiE-like methlytransferase, putative  mitochondrion ? methyltransferase activity (IEA) ? S-adenosylmethionine-dependent methyltransferase activity (IEA) ? coenzyme biosynthesis (IEA) ? quinone cofactor methyltransferase activity ? quinone cofactor biosynthesis ?  FBgn0030460 ? 2-polyprenyl-6-methoxy-1,4-benzoquinone methyltransferase activity ? S-adenosylmethionine-dependent methyltransferase activity (IEA) ?  0  BLAST| PF08\_0093 ? hypothetical protein  binding (IEA) ? transport (IEA) ? membrane (IEA) ?  FBgn0033391 ? carrier activity ? binding (IEA) ? mitochondrial membrane ? mitochondrial inner membrane ? mitochondrial transport ? folic acid transporter activity ? folic acid transport ?  1e-08  BLAST| PFE1265w ? G-protein coupled receptor, putative  G-protein coupled receptor activity ? integral to plasma membrane ? G-protein coupled receptor protein signaling pathway ?  FBgn0027498 ? G-protein coupled receptor activity ? G-protein coupled receptor protein signaling pathway ? integral to membrane (IEA) ?  8e-20  BLAST| PFE0435c ? single-strand binding protein, putative  nucleic acid binding (IEA) ? single-stranded DNA binding ? DNA replication (IEA) ? apicoplast ?  FBgn0010438 ? mitochondrial genome maintenance ? mitochondrial chromosome ? single-stranded DNA binding ? mitochondrion ? DNA replication (IEA) ? DNA repair (IEA) ?  4e-12  BLAST| PFE1125w ? 50S ribosomal subunit protein L17, putative  structural constituent of ribosome ? intracellular (IEA) ? mitochondrial large ribosomal subunit ? ribosome (IEA) ? protein biosynthesis ?  FBgn0035122 ? structural constituent of ribosome ? mitochondrial large ribosomal subunit ? protein biosynthesis ?  0.023  BLAST | | | | | | | | | | | | | | | | | |

## Cluster Pair #14: 2 gene pairs.

|  |  |  |  |  |  |  |  |  |
| --- | --- | --- | --- | --- | --- | --- | --- | --- |
| P.falciparum D.melanogaster Blast evalue|  |  |  |  |  |  | | --- | --- | --- | --- | --- | --- | | PF13\_0174 ? P. falciparum homologue of human mbp-1 interacting protein-2a%3B putative  intracellular transporter activity (IEA) ? intracellular (IEA) ? transport (IEA) ? ER to Golgi transport ? vesicle-mediated transport ?  FBgn0036573 ? intracellular transporter activity (IEA) ? intracellular (IEA) ? ER to Golgi transport (IEA) ? cell proliferation (IEA) ? transcription regulator activity (IEA) ?  6e-22  BLAST| PF14\_0243 ? dynein-associated protein, putative  microtubule motor activity ? cytoskeleton ? microtubule-based movement ?  FBgn0036195 ? motor activity ? structural constituent of cytoskeleton (IEA) ? cytoplasmic dynein complex ? microtubule associated complex ? RNA localization (IEA) ? intracellular protein transport (IEA) ? microtubule-based movement ? cell cycle (IEA) ? vesicle-mediated transport (IEA) ? ATPase activity, coupled ?  0  BLAST | | | | | | | | |

## Cluster Pair #15: 11 gene pairs.

|  |  |  |  |  |  |  |  |  |  |  |  |  |  |  |  |  |  |  |  |  |  |  |  |  |  |  |  |  |  |  |  |  |  |  |  |
| --- | --- | --- | --- | --- | --- | --- | --- | --- | --- | --- | --- | --- | --- | --- | --- | --- | --- | --- | --- | --- | --- | --- | --- | --- | --- | --- | --- | --- | --- | --- | --- | --- | --- | --- | --- |
| P.falciparum D.melanogaster Blast evalue|  |  |  |  |  |  |  |  |  |  |  |  |  |  |  |  |  |  |  |  |  |  |  |  |  |  |  |  |  |  |  |  |  | | --- | --- | --- | --- | --- | --- | --- | --- | --- | --- | --- | --- | --- | --- | --- | --- | --- | --- | --- | --- | --- | --- | --- | --- | --- | --- | --- | --- | --- | --- | --- | --- | --- | | PFB0635w ? T-complex protein 1, putative  protein binding (IEA) ? ATP binding (IEA) ? protein folding ? ATPase activity, coupled ? cellular protein metabolism (IEA) ? unfolded protein binding (IEA) ?  FBgn0033342 ? ATP binding (IEA) ? chaperonin-containing T-complex ? protein folding ? ATPase activity, coupled ? unfolded protein binding (IEA) ?  0  BLAST| PF10\_0155 ? enolase  phosphopyruvate hydratase complex ? phosphopyruvate hydratase activity ? gluconeogenesis ? glycolysis ?  FBgn0000579 ? phosphopyruvate hydratase complex (IEA) ? phosphopyruvate hydratase activity ? glycolysis ?  0  BLAST| PF14\_0023 ? hypothetical protein, conserved  membrane ?  FBgn0031227 ?  6e-09  BLAST| PF08\_0096 ? RNA helicase, putative  nucleic acid binding (IEA) ? RNA helicase activity ? helicase activity (IEA) ? ATP binding (IEA) ? ATP-dependent helicase activity (IEA) ?  FBgn0003970 ? nucleic acid binding (IEA) ? RNA helicase activity ? ATP binding (IEA) ? mRNA polyadenylylation ? pole cell fate determination ? oocyte fate determination (sensu Insecta) ? oocyte anterior/posterior axis determination ? pole plasm assembly ? pole plasm RNA localization ? ATP-dependent helicase activity ? intracellular mRNA localization ? oogenesis (sensu Insecta) ? polar granule ? karyosome formation ? pole plasm ? regulation of oskar mRNA translation ? positive regulation of oskar mRNA translation ? dorsal appendage formation ? germ-line cyst formation ?  0  BLAST| PF14\_0324 ? hypothetical protein, conserved   FBgn0024352 ? protein folding ? protein complex assembly (IEA) ? response to stress (IEA) ? defense response (IEA) ? unfolded protein binding ?  0  BLAST| PFC0271c ? glutaredoxin, putative  electron transport (IEA) ? response to oxidative stress ? electron carrier activity (IEA) ? glutathione disulfide oxidoreductase activity ? cell redox homeostasis (IEA) ?  FBgn0036820 ?  2e-12  BLAST| PF11\_0258 ? co-chaperone GrpE, putative  adenyl-nucleotide exchange factor activity (IEA) ? protein binding (IEA) ? mitochondrion ? mitochondrial matrix ? protein folding (IEA) ? mitochondrial matrix protein import ? protein homodimerization activity (IEA) ? unfolded protein binding ? chaperone binding (IEA) ?  FBgn0014877 ? adenyl-nucleotide exchange factor activity (IEA) ? mitochondrion ? mitochondrial matrix ? protein folding ? protein targeting to mitochondrion ? protein homodimerization activity (IEA) ? unfolded protein binding ? chaperone binding (IEA) ?  1e-28  BLAST| PF07\_0085 ? ferrodoxin reductase-like protein  electron transport ? electron carrier activity ? disulfide oxidoreductase activity ? oxidoreductase activity (IEA) ?  FBgn0025628 ? electron transport (IEA) ? ferredoxin metabolism (IEA) ? oxygen and reactive oxygen species metabolism (IEA) ? defense response (IEA) ? disulfide oxidoreductase activity (IEA) ?  0  BLAST| PFL1425w ? t-complex protein 1, gamma subunit, putative  protein binding (IEA) ? ATP binding ? chaperonin-containing T-complex ? protein folding ? cellular protein metabolism (IEA) ? unfolded protein binding ?  FBgn0015019 ? ATP binding (IEA) ? cytoplasm ? chaperonin-containing T-complex ? protein folding ? ATPase activity, coupled ? unfolded protein binding ?  0  BLAST| PFA0145c ? aspartyl-tRNA synthetase  nucleic acid binding (IEA) ? tRNA ligase activity (IEA) ? aspartate-tRNA ligase activity ? ATP binding (IEA) ? cytoplasm (IEA) ? tRNA aminoacylation for protein translation (IEA) ? aspartyl-tRNA aminoacylation ?  FBgn0002069 ? mRNA binding (IEA) ? aspartate-tRNA ligase activity ? ATP binding (IEA) ? cytoplasm (IEA) ? aspartyl-tRNA aminoacylation ? growth ?  0  BLAST| PFC0900w ? T-complex protein 1 epsilon subunit, putative  protein binding (IEA) ? ATP binding (IEA) ? chaperonin-containing T-complex ? protein folding ? cellular protein metabolism (IEA) ? unfolded protein binding ?  FBgn0010621 ? ATP binding (IEA) ? chaperonin-containing T-complex ? protein folding ? ATPase activity, coupled ? unfolded protein binding ?  0  BLAST | | | | | | | | | | | | | | | | | | | | | | | | | | | | | | | | | | | |

## Cluster Pair #16: 6 gene pairs.

|  |  |  |  |  |  |  |  |  |  |  |  |  |  |  |  |  |  |  |  |  |
| --- | --- | --- | --- | --- | --- | --- | --- | --- | --- | --- | --- | --- | --- | --- | --- | --- | --- | --- | --- | --- |
| P.falciparum D.melanogaster Blast evalue|  |  |  |  |  |  |  |  |  |  |  |  |  |  |  |  |  |  | | --- | --- | --- | --- | --- | --- | --- | --- | --- | --- | --- | --- | --- | --- | --- | --- | --- | --- | | PF13\_0042 ? fork head domain protein, putative  protein binding ? nucleus ?  FBgn0038640 ? anion exchanger adaptor activity ?  0.005  BLAST| PFL2020c ? hypothetical protein  electron transport (IEA) ? electron carrier activity (IEA) ?  FBgn0033814 ? protein modification (IEA) ? electron carrier activity (IEA) ? protein disulfide oxidoreductase activity (IEA) ? glucosidase activity (IEA) ? flavin-linked sulfhydryl oxidase activity ?  3e-06  BLAST| PF10\_0122 ? phosphoglucomutase, putative  carbohydrate metabolism (IEA) ? intramolecular transferase activity, phosphotransferases (IEA) ?  FBgn0033377 ? phosphomannomutase activity ? monosaccharide metabolism (IEA) ?  0  BLAST| PF13\_0358 ? mitochondrial import inner membrane translocase, putative  mitochondrial intermembrane space ? protein targeting to mitochondrion ? protein translocase activity ? mitochondrial intermembrane space protein transporter complex (IEA) ? mitochondrial inner membrane protein import (IEA) ?  FBgn0030480 ? mitochondrial inner membrane presequence translocase complex ? protein targeting to mitochondrion ? protein transport ? protein translocase activity ? mitochondrial intermembrane space protein transporter complex (IEA) ? mitochondrial inner membrane protein import (IEA) ?  4e-09  BLAST| PFL0210c ? eukaryotic initiation factor 5a, putative  nucleic acid binding (IEA) ? translation initiation factor activity ? translational initiation ?  FBgn0034967 ? translation initiation factor activity ? cytosol ? translational initiation ? salivary gland cell death ? translation regulator activity ? autophagic cell death ?  0  BLAST| PFL2075c ? hypothetical protein, conserved  nucleic acid binding (IEA) ? nucleus (IEA) ? zinc ion binding (IEA) ?  FBgn0035520 ?  5e-15  BLAST | | | | | | | | | | | | | | | | | | | | |

## Cluster Pair #17: 16 gene pairs.

|  |  |  |  |  |  |  |  |  |  |  |  |  |  |  |  |  |  |  |  |  |  |  |  |  |  |  |  |  |  |  |  |  |  |  |  |  |  |  |  |  |  |  |  |  |  |  |  |  |  |  |
| --- | --- | --- | --- | --- | --- | --- | --- | --- | --- | --- | --- | --- | --- | --- | --- | --- | --- | --- | --- | --- | --- | --- | --- | --- | --- | --- | --- | --- | --- | --- | --- | --- | --- | --- | --- | --- | --- | --- | --- | --- | --- | --- | --- | --- | --- | --- | --- | --- | --- | --- |
| P.falciparum D.melanogaster Blast evalue|  |  |  |  |  |  |  |  |  |  |  |  |  |  |  |  |  |  |  |  |  |  |  |  |  |  |  |  |  |  |  |  |  |  |  |  |  |  |  |  |  |  |  |  |  |  |  |  | | --- | --- | --- | --- | --- | --- | --- | --- | --- | --- | --- | --- | --- | --- | --- | --- | --- | --- | --- | --- | --- | --- | --- | --- | --- | --- | --- | --- | --- | --- | --- | --- | --- | --- | --- | --- | --- | --- | --- | --- | --- | --- | --- | --- | --- | --- | --- | --- | | PF14\_0688 ? hypothetical protein, conserved   FBgn0037550 ?  4e-21  BLAST| MAL8P1.19 ? hypothetical protein, conserved  nucleic acid binding (IEA) ? helicase activity (IEA) ? ATP binding (IEA) ? ATP-dependent helicase activity (IEA) ? apicoplast ? \*\* also with: FBgn0032919, clust.pair #17 FBgn0036104 ? nucleic acid binding (IEA) ? ATP-dependent RNA helicase activity ? helicase activity ? ATP binding (IEA) ? nucleobase, nucleoside, nucleotide and nucleic acid metabolism (IEA) ?  1e-27  BLAST| MAL8P1.19 ? hypothetical protein, conserved  nucleic acid binding (IEA) ? helicase activity (IEA) ? ATP binding (IEA) ? ATP-dependent helicase activity (IEA) ? apicoplast ? \*\* also with: FBgn0036104, clust.pair #17 FBgn0032919 ? nucleic acid binding (IEA) ? ATP-dependent RNA helicase activity ? ATP binding (IEA) ? nucleobase, nucleoside, nucleotide and nucleic acid metabolism (IEA) ? metal ion transport (IEA) ? metal ion binding (IEA) ?  7e-27  BLAST| PF14\_0546 ? hypothetical protein, conserved   FBgn0030520 ? nucleic acid binding (IEA) ? translation elongation factor activity (IEA) ? protein biosynthesis (IEA) ? apoptosis (IEA) ? induction of apoptosis (IEA) ? translation factor activity, nucleic acid binding (IEA) ? protein metabolism (IEA) ?  0.028  BLAST| PF11\_0090 ? hypothetical protein  intracellular (IEA) ? nucleolus (IEA) ? cell proliferation (IEA) ?  FBgn0032138 ? nucleic acid binding (IEA) ? mRNA binding (IEA) ? structural constituent of ribosome (IEA) ? intracellular (IEA) ? nucleolus (IEA) ? nucleobase, nucleoside, nucleotide and nucleic acid metabolism (IEA) ? protein complex assembly (IEA) ? cell proliferation (IEA) ? rRNA metabolism (IEA) ? protein metabolism (IEA) ?  0  BLAST| PF13\_0286 ? methyltransferase, putative  rRNA processing ? RNA methyltransferase activity ?  FBgn0030720 ? nucleic acid binding (IEA) ? nucleolus ? rRNA processing ? rRNA methyltransferase activity ? rRNA metabolism (IEA) ?  0  BLAST| PFA0330w ? pfAARP2 protein  plasma membrane ?  FBgn0036686 ?  0  BLAST| PFB0630c ? hypothetical protein   FBgn0032678 ?  4e-08  BLAST| MAL7P1.24 ? hypothetical protein, conserved  intracellular (IEA) ?  FBgn0025366 ?  0  BLAST| PF08\_0054 ? heat shock 70 kDa protein  ATP binding (IEA) ? response to unfolded protein ? response to heat ?  FBgn0001219 ? embryonic development (sensu Insecta) ? ATP binding (IEA) ? nucleus ? mitochondrion ? protein folding ? protein complex assembly (IEA) ? defense response (IEA) ? neurotransmitter secretion ? nervous system development ? axon guidance ? axonal fasciculation ? response to heat ? vesicle-mediated transport ? ATPase activity ? protein refolding ? synaptic vesicle transport (IEA) ? unfolded protein binding ?  0  BLAST| PFC0005w ? PfEMP1  receptor activity (IEA) ? glycosaminoglycan binding (IEA) ? pathogenesis (IEA) ? integral to membrane (IEA) ?  FBgn0025140 ? nucleic acid binding (IEA) ? RNA helicase activity ? ATP-dependent RNA helicase activity ? ATP binding (IEA) ? nucleus ? nucleolus ? nucleobase, nucleoside, nucleotide and nucleic acid metabolism (IEA) ?  0.013  BLAST| PFC0100c ? regulatory protein, putative   FBgn0030365 ? cytosol ? Golgi organization and biogenesis ? signal transduction (IEA) ?  0  BLAST| PF11\_0418 ? hypothetical protein  chromatin (IEA) ? chromatin binding (IEA) ? nucleus (IEA) ? chromatin assembly or disassembly (IEA) ? \*\* also with: FBgn0039019, clust.pair #17 FBgn0030082 ? chromatin (IEA) ? chromatin binding ? nucleus (IEA) ? chromatin assembly or disassembly (IEA) ? regulation of transcription from RNA polymerase II promoter (IEA) ?  0.006  BLAST| PF11\_0418 ? hypothetical protein  chromatin (IEA) ? chromatin binding (IEA) ? nucleus (IEA) ? chromatin assembly or disassembly (IEA) ? \*\* also with: FBgn0030082, clust.pair #17 FBgn0039019 ? chromatin (IEA) ? chromatin binding ? nucleus (IEA) ? chromatin assembly or disassembly (IEA) ? regulation of transcription from RNA polymerase II promoter (IEA) ?  0.013  BLAST| PF14\_0437 ? helicase, truncated, putative  nucleic acid binding ? helicase activity ? ATP binding (IEA) ? ATP-dependent helicase activity (IEA) ?  FBgn0003261 ? regulation of alternative nuclear mRNA splicing, via spliceosome ? mRNA binding ? ATP-dependent RNA helicase activity ? protein binding ? ATP binding (IEA) ? nucleus ? ATP-dependent helicase activity ? RNA interference ? antimicrobial humoral response ?  8e-39  BLAST| PFE0300c ? 60S ribosomal subunit protein L24, putative  structural constituent of ribosome ? intracellular (IEA) ? ribosome (IEA) ? protein biosynthesis ? large ribosomal subunit ?  FBgn0037899 ? nucleic acid binding (IEA) ? structural constituent of ribosome ? cytosolic large ribosomal subunit (sensu Eukaryota) ? protein biosynthesis ? ribosome biogenesis and assembly ?  7e-39  BLAST | | | | | | | | | | | | | | | | | | | | | | | | | | | | | | | | | | | | | | | | | | | | | | | | | | |

## Cluster Pair #18: 10 gene pairs.

|  |  |  |  |  |  |  |  |  |  |  |  |  |  |  |  |  |  |  |  |  |  |  |  |  |  |  |  |  |  |  |  |  |
| --- | --- | --- | --- | --- | --- | --- | --- | --- | --- | --- | --- | --- | --- | --- | --- | --- | --- | --- | --- | --- | --- | --- | --- | --- | --- | --- | --- | --- | --- | --- | --- | --- |
| P.falciparum D.melanogaster Blast evalue|  |  |  |  |  |  |  |  |  |  |  |  |  |  |  |  |  |  |  |  |  |  |  |  |  |  |  |  |  |  | | --- | --- | --- | --- | --- | --- | --- | --- | --- | --- | --- | --- | --- | --- | --- | --- | --- | --- | --- | --- | --- | --- | --- | --- | --- | --- | --- | --- | --- | --- | | PFD0470c ? replication factor a protein, putative  nucleic acid binding (IEA) ? DNA replication factor A complex ? DNA replication ? DNA-dependent DNA replication ?  FBgn0010173 ? single-stranded DNA binding ? nucleus ? DNA replication factor A complex ? DNA-dependent DNA replication ?  4.00001e-40  BLAST| PFB0465c ? hypothetical protein  membrane ?  FBgn0033028 ? cation transport (IEA) ? monocarboxylic acid transporter activity ?  9e-08  BLAST| PF14\_0133 ? ATP-dependent transporter, putative  ATP binding (IEA) ? transport ? membrane ? ATPase activity (IEA) ? apicoplast ? ATPase activity, coupled to transmembrane movement of substances ?  FBgn0030321 ? transporter activity ? ATP binding (IEA) ? transport (IEA) ? ATPase activity, coupled to transmembrane movement of substances ? ATP-binding cassette (ABC) transporter complex ?  1e-07  BLAST| PFB0840w ? replication factor C, subunit 2  nucleotide binding (IEA) ? DNA binding (IEA) ? ATP binding (IEA) ? DNA replication factor C complex ? DNA replication ? nucleoside-triphosphatase activity (IEA) ? protein complex (IEA) ?  FBgn0030871 ? DNA binding ? DNA-directed DNA polymerase activity (IEA) ? ATP binding (IEA) ? DNA replication factor C complex ? DNA replication ? DNA repair (IEA) ? nucleoside-triphosphatase activity (IEA) ?  0  BLAST| PF11\_0361 ? hypothetical protein  membrane ?  FBgn0031662 ? integral to membrane ?  0.042  BLAST| PF10\_0316 ? phosphatidyl inositol glycan, class A, putative  catalytic activity ? GPI anchor biosynthesis ? biosynthesis (IEA) ? membrane ?  FBgn0034270 ? polysaccharide metabolism (IEA) ? GPI anchor biosynthesis ? phosphatidylinositol N-acetylglucosaminyltransferase activity ? integral to endoplasmic reticulum membrane ?  0  BLAST| PF14\_0053 ? ribonucleotide reductase small subunit  ribonucleoside-diphosphate reductase activity ? DNA replication ? deoxyribonucleoside diphosphate metabolism (IEA) ?  FBgn0011704 ? ribonucleoside-diphosphate reductase activity ? ribonucleoside-diphosphate reductase complex ? purine base metabolism (IEA) ? pyrimidine base metabolism (IEA) ? DNA replication ? deoxyribonucleoside diphosphate metabolism (IEA) ?  0  BLAST| MAL13P1.326 ? ferrochelatase, putative  ferrochelatase activity ? heme biosynthesis ? ferrous iron binding ?  FBgn0024891 ? ferrochelatase activity ? mitochondrial inner membrane ? protoporphyrinogen IX biosynthesis ?  5e-19  BLAST| PF14\_0376 ? hypothetical protein  membrane ?  FBgn0028507 ?  3e-31  BLAST| PFE0270c ? DNA repair protein, putative  damaged DNA binding ? ATP binding (IEA) ? DNA metabolism (IEA) ? DNA repair ? mismatch repair (IEA) ?  FBgn0036486 ? damaged DNA binding (IEA) ? ATP binding (IEA) ? DNA repair ? mismatch repair (IEA) ? postreplication repair ?  0  BLAST | | | | | | | | | | | | | | | | | | | | | | | | | | | | | | | | |

## Cluster Pair #19: 39 gene pairs.

|  |  |  |  |  |  |  |  |  |  |  |  |  |  |  |  |  |  |  |  |  |  |  |  |  |  |  |  |  |  |  |  |  |  |  |  |  |  |  |  |  |  |  |  |  |  |  |  |  |  |  |  |  |  |  |  |  |  |  |  |  |  |  |  |  |  |  |  |  |  |  |  |  |  |  |  |  |  |  |  |  |  |  |  |  |  |  |  |  |  |  |  |  |  |  |  |  |  |  |  |  |  |  |  |  |  |  |  |  |  |  |  |  |  |  |  |  |  |  |  |
| --- | --- | --- | --- | --- | --- | --- | --- | --- | --- | --- | --- | --- | --- | --- | --- | --- | --- | --- | --- | --- | --- | --- | --- | --- | --- | --- | --- | --- | --- | --- | --- | --- | --- | --- | --- | --- | --- | --- | --- | --- | --- | --- | --- | --- | --- | --- | --- | --- | --- | --- | --- | --- | --- | --- | --- | --- | --- | --- | --- | --- | --- | --- | --- | --- | --- | --- | --- | --- | --- | --- | --- | --- | --- | --- | --- | --- | --- | --- | --- | --- | --- | --- | --- | --- | --- | --- | --- | --- | --- | --- | --- | --- | --- | --- | --- | --- | --- | --- | --- | --- | --- | --- | --- | --- | --- | --- | --- | --- | --- | --- | --- | --- | --- | --- | --- | --- | --- | --- | --- |
| P.falciparum D.melanogaster Blast evalue|  |  |  |  |  |  |  |  |  |  |  |  |  |  |  |  |  |  |  |  |  |  |  |  |  |  |  |  |  |  |  |  |  |  |  |  |  |  |  |  |  |  |  |  |  |  |  |  |  |  |  |  |  |  |  |  |  |  |  |  |  |  |  |  |  |  |  |  |  |  |  |  |  |  |  |  |  |  |  |  |  |  |  |  |  |  |  |  |  |  |  |  |  |  |  |  |  |  |  |  |  |  |  |  |  |  |  |  |  |  |  |  |  |  |  |  |  | | --- | --- | --- | --- | --- | --- | --- | --- | --- | --- | --- | --- | --- | --- | --- | --- | --- | --- | --- | --- | --- | --- | --- | --- | --- | --- | --- | --- | --- | --- | --- | --- | --- | --- | --- | --- | --- | --- | --- | --- | --- | --- | --- | --- | --- | --- | --- | --- | --- | --- | --- | --- | --- | --- | --- | --- | --- | --- | --- | --- | --- | --- | --- | --- | --- | --- | --- | --- | --- | --- | --- | --- | --- | --- | --- | --- | --- | --- | --- | --- | --- | --- | --- | --- | --- | --- | --- | --- | --- | --- | --- | --- | --- | --- | --- | --- | --- | --- | --- | --- | --- | --- | --- | --- | --- | --- | --- | --- | --- | --- | --- | --- | --- | --- | --- | --- | --- | | PF11\_0043 ? 60S acidic ribosomal protein p1, putative  structural constituent of ribosome ? intracellular (IEA) ? ribosome (IEA) ? protein biosynthesis ? translational elongation (IEA) ? large ribosomal subunit ?  FBgn0002593 ? nucleic acid binding (IEA) ? structural constituent of ribosome ? cytosolic large ribosomal subunit (sensu Eukaryota) ? protein biosynthesis ? translational elongation ?  1e-10  BLAST| PF08\_0019 ? guanine nucleotide-binding protein, putative  protein kinase C binding ? heterotrimeric G-protein complex ? G-protein coupled receptor protein signaling pathway ?  FBgn0020618 ? protein kinase C binding ? protein targeting (IEA) ? signal transduction (IEA) ?  0  BLAST| PF14\_0083 ? ribosomal protein S8e, putative  structural constituent of ribosome ? intracellular (IEA) ? mitochondrion ? cytosolic small ribosomal subunit (sensu Eukaryota) ? protein biosynthesis ?  FBgn0039713 ? nucleic acid binding (IEA) ? structural constituent of ribosome ? cytosolic small ribosomal subunit (sensu Eukaryota) ? protein biosynthesis ?  0  BLAST| PF14\_0448 ? ribosomal protein S2, putative  structural constituent of ribosome ? intracellular (IEA) ? ribosome (IEA) ? cytosolic small ribosomal subunit (sensu Eukaryota) ? protein biosynthesis ? small ribosomal subunit (IEA) ?  FBgn0004867 ? nucleic acid binding (IEA) ? structural constituent of ribosome ? ribosome ? cytosolic small ribosomal subunit (sensu Eukaryota) ? protein biosynthesis ?  0  BLAST| PF14\_0125 ? deoxyhypusine synthase  protein biosynthesis ? hypusine biosynthesis from peptidyl-lysine ? membrane ? spermidine catabolism to deoxyhypusine, using deoxyhypusine synthase ?  FBgn0035854 ? hypusine biosynthesis from peptidyl-lysine ? spermidine catabolism to deoxyhypusine, using deoxyhypusine synthase ?  7.00649e-43  BLAST| PF13\_0316 ? 40S ribosomal protein S13  structural constituent of ribosome ? intracellular (IEA) ? ribosome (IEA) ? cytosolic small ribosomal subunit (sensu Eukaryota) ? protein biosynthesis ?  FBgn0010265 ? nucleic acid binding (IEA) ? structural constituent of ribosome ? ribosome ? cytosolic small ribosomal subunit (sensu Eukaryota) ? protein biosynthesis ?  0  BLAST| PF10\_0326 ? hypothetical protein   FBgn0034908 ?  4e-21  BLAST| PF07\_0043 ? 60S ribosomal protein L34-a, putative  structural constituent of ribosome (IEA) ? intracellular (IEA) ? ribosome (IEA) ? protein biosynthesis (IEA) ?  FBgn0037686 ? nucleic acid binding (IEA) ? structural constituent of ribosome ? cytosolic large ribosomal subunit (sensu Eukaryota) ? protein biosynthesis ?  1e-24  BLAST| PF14\_0296 ? ribosomal protein L14, putative  structural constituent of ribosome ? intracellular (IEA) ? ribosome (IEA) ? cytosolic small ribosomal subunit (sensu Eukaryota) ? protein biosynthesis ? apicoplast ?  FBgn0017579 ? nucleic acid binding (IEA) ? structural constituent of ribosome ? cytosolic ribosome (sensu Eukaryota) ? ribosome ? cytosolic large ribosomal subunit (sensu Eukaryota) ? protein biosynthesis ?  0.005  BLAST| PFE0750c ? hypothetical protein, conserved  nucleic acid binding (IEA) ?  FBgn0037220 ? nucleotide binding (IEA) ? regulation of alternative nuclear mRNA splicing, via spliceosome ? mRNA binding ? nucleus ? zinc ion binding (IEA) ?  2e-06  BLAST| PF08\_0039 ? ribosomal protein, putative  structural constituent of ribosome ? intracellular (IEA) ? ribosome (IEA) ? cytosolic large ribosomal subunit (sensu Eukaryota) ? protein biosynthesis ?  FBgn0015288 ? nucleic acid binding (IEA) ? structural constituent of ribosome ? ribosome ? cytosolic large ribosomal subunit (sensu Eukaryota) ? protein biosynthesis ?  7e-15  BLAST| PFD0180c ? CGI-201 protein, short form  spliceosome assembly ? RNA binding ? intracellular (IEA) ? RNA processing (IEA) ?  FBgn0000377 ? regulation of alternative nuclear mRNA splicing, via spliceosome ? nuclear mRNA splicing, via spliceosome ? nucleus ? spliceosome complex ? neuroblast proliferation ? central nervous system development ? peripheral nervous system development ? Malpighian tubule morphogenesis ? nuclear speck ?  0  BLAST| PFB0885w ? 40S ribosomal protein S30, putative  structural constituent of ribosome ? intracellular (IEA) ? mitochondrion ? ribosome (IEA) ? cytosolic small ribosomal subunit (sensu Eukaryota) ? protein biosynthesis ?  FBgn0038834 ? nucleic acid binding (IEA) ? structural constituent of ribosome ? cytosolic small ribosomal subunit (sensu Eukaryota) ? protein biosynthesis (IEA) ?  1e-15  BLAST| PF08\_0075 ? 60S ribosomal protein L13, putative  structural constituent of ribosome ? intracellular (IEA) ? ribosome (IEA) ? cytosolic large ribosomal subunit (sensu Eukaryota) ? protein biosynthesis ?  FBgn0011272 ? nucleic acid binding (IEA) ? structural constituent of ribosome ? ribosome ? cytosolic large ribosomal subunit (sensu Eukaryota) ? protein biosynthesis ?  3e-28  BLAST| PFC0200w ? 60S Ribosomal protein L44, putative  structural constituent of ribosome ? intracellular (IEA) ? ribosome (IEA) ? cytosolic large ribosomal subunit (sensu Eukaryota) ? protein biosynthesis ?  FBgn0031980 ? nucleic acid binding (IEA) ? structural constituent of ribosome ? cytosolic large ribosomal subunit (sensu Eukaryota) ? protein biosynthesis ?  3e-28  BLAST| PF10\_0264 ? 40S ribosomal protein, putative  structural constituent of ribosome ? intracellular (IEA) ? ribosome (IEA) ? cytosolic small ribosomal subunit (sensu Eukaryota) ? protein biosynthesis ? small ribosomal subunit (IEA) ?  FBgn0003517 ? nucleic acid binding (IEA) ? structural constituent of ribosome ? ribosome ? cytosolic small ribosomal subunit (sensu Eukaryota) ? protein biosynthesis ? protein metabolism (IEA) ?  0  BLAST| PF10\_0043 ? ribosomal protein L13, putative  structural constituent of ribosome ? intracellular (IEA) ? ribosome (IEA) ? cytosolic large ribosomal subunit (sensu Eukaryota) ? protein biosynthesis ? large ribosomal subunit (IEA) ?  FBgn0037351 ? nucleic acid binding (IEA) ? structural constituent of ribosome ? cytosolic large ribosomal subunit (sensu Eukaryota) ? protein biosynthesis ?  0  BLAST| PFC0290w ? 40S ribosomal protein S23, putative  nucleic acid binding (IEA) ? structural constituent of ribosome ? intracellular (IEA) ? ribosome (IEA) ? cytosolic small ribosomal subunit (sensu Eukaryota) ? protein biosynthesis ? small ribosomal subunit (IEA) ?  FBgn0033912 ? nucleic acid binding (IEA) ? structural constituent of ribosome ? cytosolic small ribosomal subunit (sensu Eukaryota) ? protein biosynthesis ?  0  BLAST| PF10\_0272 ? ribosomal protein L3, putative  structural constituent of ribosome ? intracellular (IEA) ? mitochondrion ? ribosome (IEA) ? cytosolic large ribosomal subunit (sensu Eukaryota) ? protein biosynthesis ?  FBgn0020910 ? nucleic acid binding (IEA) ? structural constituent of ribosome ? cytosolic ribosome (sensu Eukaryota) ? cytosolic large ribosomal subunit (sensu Eukaryota) ? protein biosynthesis ?  0  BLAST| PFE0975c ? 40S ribosomal subunit protein S24, putative  structural constituent of ribosome (IEA) ? intracellular (IEA) ? ribosome (IEA) ? protein biosynthesis (IEA) ?  FBgn0034751 ? nucleic acid binding (IEA) ? structural constituent of ribosome ? cytosolic small ribosomal subunit (sensu Eukaryota) ? protein biosynthesis ?  5e-31  BLAST| PFE1005w ? 40S ribosomal subunit protein S9, putative  RNA binding (IEA) ? structural constituent of ribosome (IEA) ? intracellular (IEA) ? ribosome (IEA) ? protein biosynthesis (IEA) ? small ribosomal subunit (IEA) ?  FBgn0010408 ? nucleic acid binding (IEA) ? structural constituent of ribosome ? ribosome ? cytosolic small ribosomal subunit (sensu Eukaryota) ? protein biosynthesis ?  0  BLAST| PF11\_0260 ? ribosomal protein L35, putative  structural constituent of ribosome ? intracellular (IEA) ? ribosome (IEA) ? protein biosynthesis ? large ribosomal subunit ?  FBgn0029785 ? nucleic acid binding (IEA) ? structural constituent of ribosome ? cytosolic large ribosomal subunit (sensu Eukaryota) ? protein biosynthesis ?  2e-08  BLAST| PF11\_0106 ? 60S Ribosomal protein L36, putative  structural constituent of ribosome ? intracellular (IEA) ? ribosome (IEA) ? cytosolic large ribosomal subunit (sensu Eukaryota) ? protein biosynthesis ? membrane ?  FBgn0002579 ? nucleic acid binding (IEA) ? structural constituent of ribosome ? ribosome ? cytosolic large ribosomal subunit (sensu Eukaryota) ? protein biosynthesis ?  4e-12  BLAST| PF11\_0313 ? ribosomal phosphoprotein P0  structural constituent of ribosome ? intracellular (IEA) ? mitochondrion ? ribosome (IEA) ? cytosolic large ribosomal subunit (sensu Eukaryota) ? protein biosynthesis ? translational elongation (IEA) ? ribosome biogenesis and assembly (IEA) ?  FBgn0000100 ? mRNA binding (IEA) ? structural constituent of ribosome ? DNA-(apurinic or apyrimidinic site) lyase activity ? nucleus ? cytoplasm ? cytosolic ribosome (sensu Eukaryota) ? ribosome ? cytosolic large ribosomal subunit (sensu Eukaryota) ? DNA repair ? protein biosynthesis ? translational elongation (IEA) ? ribosome biogenesis and assembly (IEA) ?  0  BLAST| PFB0445c ? helicase, putative  nucleic acid binding (IEA) ? ATP-dependent RNA helicase activity ? helicase activity (IEA) ? ATP binding (IEA) ? ATP-dependent helicase activity (IEA) ?  FBgn0014189 ? regulation of alternative nuclear mRNA splicing, via spliceosome ? nuclear mRNA splicing, via spliceosome ? RNA helicase activity ? ATP-dependent RNA helicase activity ? ATP binding (IEA) ? nucleus ? spliceosome complex ? mRNA-nucleus export ? RNA splicing factor activity, transesterification mechanism ?  0  BLAST| PF14\_0627 ? ribosomal protein S3, putative  nucleic acid binding (IEA) ? structural constituent of ribosome ? intracellular (IEA) ? ribosome (IEA) ? cytosolic small ribosomal subunit (sensu Eukaryota) ? protein biosynthesis ? small ribosomal subunit (IEA) ?  FBgn0002622 ? nucleic acid binding (IEA) ? structural constituent of ribosome ? DNA-(apurinic or apyrimidinic site) lyase activity ? nucleus ? cytoplasm ? ribosome ? cytosolic small ribosomal subunit (sensu Eukaryota) ? DNA repair ? protein biosynthesis ? purine-specific oxidized base lesion DNA N-glycosylase activity ? nuclear matrix ?  0  BLAST| PF14\_0585 ? ribosomal protein S28e, putative  structural constituent of ribosome ? intracellular (IEA) ? ribosome (IEA) ? cytosolic small ribosomal subunit (sensu Eukaryota) ? protein biosynthesis ?  FBgn0030136 ? nucleic acid binding (IEA) ? structural constituent of ribosome ? cytosolic small ribosomal subunit (sensu Eukaryota) ? protein biosynthesis (IEA) ?  2e-09  BLAST| MAL13P1.209 ? 60S ribosomal subunit porotein L18, putative  structural constituent of ribosome ? intracellular (IEA) ? ribosome (IEA) ? cytosolic large ribosomal subunit (sensu Eukaryota) ? protein biosynthesis ?  FBgn0035753 ? nucleic acid binding (IEA) ? structural constituent of ribosome ? cytosolic large ribosomal subunit (sensu Eukaryota) ? protein biosynthesis ?  0  BLAST| PF07\_0088 ? 40S ribosomal protein S5, putative  structural constituent of ribosome ? intracellular (IEA) ? ribosome (IEA) ? cytosolic small ribosomal subunit (sensu Eukaryota) ? protein biosynthesis ? small ribosomal subunit (IEA) ? \*\* also with: FBgn0002590, clust.pair #19 FBgn0038277 ? nucleic acid binding (IEA) ? structural constituent of ribosome ? cytosolic small ribosomal subunit (sensu Eukaryota) ? protein biosynthesis ?  0  BLAST| PF07\_0088 ? 40S ribosomal protein S5, putative  structural constituent of ribosome ? intracellular (IEA) ? ribosome (IEA) ? cytosolic small ribosomal subunit (sensu Eukaryota) ? protein biosynthesis ? small ribosomal subunit (IEA) ? \*\* also with: FBgn0038277, clust.pair #19 FBgn0002590 ? nucleic acid binding (IEA) ? structural constituent of ribosome ? ribosome ? cytosolic small ribosomal subunit (sensu Eukaryota) ? protein biosynthesis ?  0  BLAST| PF10\_0217 ? pre-mRNA splicing factor, putative  nucleic acid binding (IEA) ? mitochondrion ? RNA splicing ? RNA splicing factor activity, transesterification mechanism ?  FBgn0004587 ? nucleotide binding (IEA) ? regulation of alternative nuclear mRNA splicing, via spliceosome ? nuclear mRNA splicing, via spliceosome ? mRNA binding ? protein binding ? nucleus ? spliceosome complex (IEA) ? mRNA splice site selection ? nuclear speck ? RNA splicing factor activity, transesterification mechanism ? omega speckle ? regulation of nuclear mRNA splicing, via spliceosome ?  3e-22  BLAST| PFE0845c ? 60S ribosomal subunit protein L8, putative  nucleic acid binding (IEA) ? structural constituent of ribosome ? intracellular (IEA) ? ribosome (IEA) ? protein biosynthesis ? large ribosomal subunit ?  FBgn0024939 ? mRNA binding (IEA) ? structural constituent of ribosome ? cytosolic large ribosomal subunit (sensu Eukaryota) ? protein biosynthesis ?  0  BLAST| PF13\_0129 ? ribosomal protein L6 homologue, putative  RNA binding ? structural constituent of ribosome ? intracellular (IEA) ? ribosome (IEA) ? protein biosynthesis ? large ribosomal subunit ?  FBgn0015756 ? nucleic acid binding (IEA) ? structural constituent of ribosome ? ribosome ? cytosolic large ribosomal subunit (sensu Eukaryota) ? protein biosynthesis ?  0  BLAST| PF13\_0049 ? 60S ribosomal protein L24, putative  structural constituent of ribosome ? intracellular (IEA) ? ribosome (IEA) ? cytosolic large ribosomal subunit (sensu Eukaryota) ? protein biosynthesis ?  FBgn0032518 ? nucleic acid binding (IEA) ? structural constituent of ribosome ? cytosolic large ribosomal subunit (sensu Eukaryota) ? protein biosynthesis ?  1e-08  BLAST| PFC0400w ? 60S Acidic ribosomal protein P2  structural constituent of ribosome (IEA) ? intracellular (IEA) ? ribosome (IEA) ? cytosolic large ribosomal subunit (sensu Eukaryota) ? translational elongation ? large ribosomal subunit ?  FBgn0003274 ? nucleic acid binding (IEA) ? structural constituent of ribosome ? cytosolic ribosome (sensu Eukaryota) ? cytosolic large ribosomal subunit (sensu Eukaryota) ? protein biosynthesis ? translational elongation ?  3e-11  BLAST| PF11\_0065 ? ribosomal protein S4, putative  RNA binding ? structural constituent of ribosome ? intracellular (IEA) ? mitochondrion ? ribosome (IEA) ? protein biosynthesis ? small ribosomal subunit ?  FBgn0011284 ? nucleic acid binding (IEA) ? structural constituent of ribosome ? ribosome ? cytosolic small ribosomal subunit (sensu Eukaryota) ? protein biosynthesis ?  0  BLAST| PF07\_0079 ? 60S ribosomal protein L11a, putative  structural constituent of ribosome ? intracellular (IEA) ? ribosome (IEA) ? cytosolic large ribosomal subunit (sensu Eukaryota) ? protein biosynthesis ?  FBgn0013325 ? nucleic acid binding (IEA) ? structural constituent of ribosome ? protein binding ? ribosome ? cytosolic large ribosomal subunit (sensu Eukaryota) ? protein biosynthesis ?  0  BLAST| PFL0310c ? eukaryotic translation initiation factor 3 subunit 8, putative  translation initiation factor activity ? eukaryotic translation initiation factor 3 complex ? translational initiation (IEA) ? regulation of translational initiation ?  FBgn0034258 ? translation initiation factor activity ? cytosol ? eukaryotic translation initiation factor 3 complex ? translational initiation ?  0  BLAST| PFE0350c ? 60S ribosomal subunit protein L4%2FL1, putative  RNA binding ? structural constituent of ribosome ? intracellular (IEA) ? ribosome (IEA) ? protein biosynthesis ? large ribosomal subunit ?  FBgn0003279 ? nucleic acid binding (IEA) ? structural constituent of ribosome ? cytosolic large ribosomal subunit (sensu Eukaryota) ? protein biosynthesis ?  0  BLAST | | | | | | | | | | | | | | | | | | | | | | | | | | | | | | | | | | | | | | | | | | | | | | | | | | | | | | | | | | | | | | | | | | | | | | | | | | | | | | | | | | | | | | | | | | | | | | | | | | | | | | | | | | | | | | | | | | | | | | | |

## Cluster Pair #20: 7 gene pairs.

|  |  |  |  |  |  |  |  |  |  |  |  |  |  |  |  |  |  |  |  |  |  |  |  |
| --- | --- | --- | --- | --- | --- | --- | --- | --- | --- | --- | --- | --- | --- | --- | --- | --- | --- | --- | --- | --- | --- | --- | --- |
| P.falciparum D.melanogaster Blast evalue|  |  |  |  |  |  |  |  |  |  |  |  |  |  |  |  |  |  |  |  |  | | --- | --- | --- | --- | --- | --- | --- | --- | --- | --- | --- | --- | --- | --- | --- | --- | --- | --- | --- | --- | --- | | PF14\_0667 ? hypothetical protein   FBgn0036685 ?  0.005  BLAST| PFC0515c ? hypothetical protein   FBgn0024352 ? protein folding ? protein complex assembly (IEA) ? response to stress (IEA) ? defense response (IEA) ? unfolded protein binding ?  7e-06  BLAST| PFD0795w ? hypothetical protein   FBgn0037376 ? histone acetyltransferase activity ? chromatin assembly or disassembly (IEA) ?  4e-11  BLAST| PF07\_0105 ? exonuclease i, putative  DNA binding (IEA) ? nuclease activity (IEA) ? nucleus ? DNA repair (IEA) ? mismatch repair ? DNA recombination ? 5'-3' exonuclease activity ?  FBgn0015553 ? nucleic acid binding (IEA) ? DNA binding (IEA) ? nuclease activity ? DNA repair (IEA) ? exodeoxyribonuclease I activity ?  4e-26  BLAST| PFL0150w ? origin recognition complex 1 protein  nucleotide binding (IEA) ? DNA replication origin binding ? ATP binding (IEA) ? DNA replication initiation ? nucleoside-triphosphatase activity (IEA) ?  FBgn0022772 ? regulation of progression through cell cycle (IEA) ? DNA binding ? DNA replication origin binding (IEA) ? ATP binding (IEA) ? nucleus ? nuclear origin of replication recognition complex ? DNA-dependent DNA replication ? DNA replication initiation ? chromatin silencing ? nucleoside-triphosphatase activity (IEA) ?  0  BLAST| PFC0710w ? inorganic pyrophosphatase, putative  magnesium ion binding (IEA) ? inorganic diphosphatase activity (IEA) ? cytoplasm (IEA) ? phosphate metabolism (IEA) ? metabolism ? pyrophosphatase activity ?  FBgn0016687 ? magnesium ion binding (IEA) ? inorganic diphosphatase activity ? nucleus ? cytoplasm ? chromatin remodeling ? transcription ? polyphosphate catabolism (IEA) ? NURF complex ? ecdysone receptor-mediated signaling pathway ? nucleosome mobilization ?  9.99995e-41  BLAST| PFC0960c ? hypothetical protein   FBgn0020503 ? actin binding ? structural constituent of cytoskeleton (IEA) ? protein binding ? Golgi apparatus ? microtubule associated complex ? spindle microtubule ? cell cortex ? protein targeting (IEA) ? microtubule-based process ? cellularization ? microtubule binding ? vesicle-mediated transport (IEA) ? fusome ?  5e-11  BLAST | | | | | | | | | | | | | | | | | | | | | | | |
