# Supplementary material for: Assessing functional annotation transfers with inter-species conserved coexpression: application to Plasmodium falciparum
Source: BMC Genomics. 2010 Jan 15;11:35. doi: 10.1186/1471-2164-11-35 (PMC2826313; doi:10.1186/1471-2164-11-35)
Supplement: Additional file 6 — Predicted annotations of P. falciparum gene coding for putative ribosomal proteins (structural constituent of ribosomes, GO:0003735). Prediction gained by co-coexpression analyses compared to PlasmoDB 5.4: (0) confirmed annotation; (1) refined annotation of an incomplete or wrong original functional inference; (2) previously hypothetical; (N): no advance based on co-coexpression analyses; (+) functional inference of hypothetical gene products by Smits et al. (2007) [32]; (*) correction of Smits et al. (2007) annotation; (Z) pair also identified in Zhou et al. (2008) [15]; (x): pairing of P. falciparum and S. cerevisiae or P. falciparum and D. melanogaster genes in co-coexpression analyses. [file 1471-2164-11-35-S6.PDF]

P. falciparum genes	Final annotation	prediction gained by co-coexpression analyses	*S. cerevisiae* genes	**Comparative analyses *P. falciparum* vs *S. cerevisiae*** Smits 2007	co-co (this work) LR-GA	co-co (this work) BO-GA	co-co (this work) BO-SP	*D. melanogaster* genes	**Comparative analyses *P. falciparum* vs *D. melanogaster*** Smits 2007	co-co (this work) BO-PI
**Cytosolic large ribosomal proteins**										
PF14_0391	60S ribosomal protein L1, putative	**0**	YPL220W<br>YGL135W		x<br>x		x	FBgn0036213		x
PF10_0272	60S ribosomal protein L3, putative	**0**	YOR063W		x	x	x	FBgn0020910		x
PFE0350c	60S ribosomal protein L4, putative	**0**	YDR012W<br>YBR031W		x<br>x	x<br>x	x<br>x	FBgn0003279		x
PF14_0230	60S ribosomal protein L5, putative	**0**	YPL131W		x	x	x			
PF13_0129	60S ribosomal protein L6, putative	**0**	YNL067W<br>YGL147C		x<br>x	x<br>x	x<br>x	FBgn0015756		x
PF13_0213	60S ribosomal protein L6-2, putative	**0**	YLR448W		x	x	x	FBgn0039857		x
PFC0300c	60S ribosomal protein L7, putative	**0**	YPL198W<br>YGL076C		x<br>x	x<br>x	x<br>x	FBgn0005593		x
MAL13P1.272	60S ribosomal protein L7-2, putative	**N**								
PF14_0231	60S ribosomal protein L7-3, putative	**0**	YHL033C		x	x	x	FBgn0014026		x
PFD0960c	60S ribosomal protein L7Ae/L30e, putative	**N**								
PFE0845c	60S ribosomal protein L8, putative	*	YFR031C-A<br>YEL050C	 x	x	x		FBgn0036135	x	
PF14_0141	60S ribosomal protein L10, putative	**0**	YLR075W		x	x	x			
PF07_0079	60S ribosomal protein L11a, putative	**0**	YGR085C			x	x	FBgn0013325		x
PF10_0043	60S ribosomal protein L13, putative	**0**	YIL133C		x	x	x	FBgn0037351		x
PF08_0075	60S ribosomal protein L13-2, putative	**0**	YDL082W		x	x	x			
PF14_0296	60S ribosomal protein L14, putative	**2**	YKL006W		x	x	x	FBgn0017579		x
PFD0770c	60S ribosomal protein L15, putative	**0**	YMR121C		x	x	x			
PF13_0268	60S ribosomal protein L17, putative	**0**	YJL177W		x	x	x	FBgn0029897		x
PF13_0224	60S ribosomal protein L18, putative	**0**	YOR312C<br>YMR242C		x<br>x	x<br>x	x<br>x	FBgn0010409		x
MAL13P1.209	60S ribosomal protein L18-2, putative	**0**	YOL120C<br>YNL301C		x<br>x	x<br>x	x<br>x	FBgn0035753		x
PFF0700c	60S ribosomal protein L19, putative	**0**	YBL027W		x					
PF14_0240	60S ribosomal protein L21e, putative	**0**	YBR191W		x	x	x			
PF08_0039	60S ribosomal protein L22, putative	**1**	YLR061W		x	x	x			
PF13_0171	60S ribosomal protein L23, putative	**0**	YER117W<br>YBL087C		x<br>x	x<br>x	x<br>x	FBgn0010078		x
PF13_0132	60S ribosomal protein L23a, putative	**0**	YOL127W		x	x	x	FBgn0026372		x
PF13_0049	60S ribosomal protein L24, putative	**0**	YGR148C		x	x	x			
PFE0300c	60S ribosomal protein L24-2, putative	**0**	YLR009W			x				
PFC0535w	60S ribosomal protein L26, putative	**0**	YLR344W<br>YGR034W		x<br>x	x<br>x	x<br>x	FBgn0036825		x
PF14_0579	60S ribosomal protein L27, putative	**0**	YHR010W		x	x	x	FBgn0039359		x
PFF0885w	60S ribosomal protein L27a, putative	**N**								
PF10_0187	60S ribosomal protein L30e, putative	**0**	YGL030W		x	x	x			
PFE0185c	60S ribosomal protein L31, putative	**0**	YLR406C		x	x	x			
PFI0190w	60S ribosomal protein L32, putative	**0**	YBL092W		x	x	x	FBgn0002626		x
PF07_0043	60S ribosomal protein L34a, putative	**0**	YIL052C<br>YER056C-A		x<br>x	x<br>x	x<br>x	FBgn0037686		x
PF11_0260	60S ribosomal protein L35, putative	**N**								
PF11_0438	60S ribosomal protein L35Ae, putative	**1**	YOR234C		x	x	x	FBgn0037328		x
PFB0455w	60S ribosomal protein L37ae, putative	**0**	YJR094W-A		x	x				
PF11_0312	60S ribosomal protein L38e, putative	**N**								
PF13_0346	60S ribosomal protein L40/UBI, putative	**N**								
PFC0200w	60S ribosomal protein L44, putative	**0**	YHR141C		x	x	x			
PF07_0121	60S ribosomal subunit export protein, putative	**2**	YHR170W			x	x			
PF11_0313	60S ribosomal protein P0	**0**	YLR340W		x	x	x	FBgn0000100		x
MAL13P1.341	60S ribosomal protein P0-2, putative	**1, Z**	YKL009W		x	x	x	FBgn0033485		x
PF11_0043	60S ribosomal protein P1, putative	**0**	YDL081C		x	x	x	FBgn0002593		x
PFC0400w	60S ribosomal protein P2	**0**	YDR382W		x	x	x	FBgn0003274		x
**Cytosolic small ribosomal proteins**										
PF14_0448	40S ribosomal protein S2, putative	**0**	YGL123W		x	x	x			
PF10_0264	40S ribosomal protein S2B, putative	**1**	YGR214W		x	x	x	FBgn0003517		x
PF14_0627	40S ribosomal protein S3, putative	**0**	YNL178W		x	x	x	FBgn0002622		x
PFC1020c	40S ribosomal protein S3A, putative	**0**	YLR441C		x	x	x			
PF11_0065	40S ribosomal protein S4, putative	**0**	YJR145C<br>YHR203C		x<br>x	x<br>x	x<br>x	FBgn0011284		x
PF07_0088	40S ribosomal protein S5, putative	**0**	YJR123W		x	x	x	FBgn0002590		x
PF13_0228	40S ribosomal protein S6, putative	**0**	YPL090C<br>YBR181C		x<br>x	x<br>x	x<br>x	FBgn0004922		x
PF13_0014	40S ribosomal protein S7, putative	**0**	YNL096C		x	x	x	FBgn0039757		x
PF14_0083	40S ribosomal protein S8e, putative	**0**	YBL072C		x	x	x			
PFE1005w	40S ribosomal protein S9, putative	**0**	YBR189W		x	x	x	FBgn0010408		x
PF14_0132	40S ribosomal protein S9A, putative	**N**								
PF07_0080	40S ribosomal protein S10, putative	**0**	YMR230W		x	x		FBgn0031035		x
PFC0775w	40S ribosomal protein S11, putative	**0**	YDR025W<br>YBR048W		x<br>x	x<br>x	x<br>x	FBgn0033699		x
PFC0295c	40S ribosomal protein S12, putative	**0**	YOR369C			x	x	FBgn0014027		x
PF13_0316	40S ribosomal protein S13, putative	**0**	YDR064W			x	x	FBgn0010265		x
PFE0810c	40S ribosomal protein S14, putative	**0**	YJL191W		x	x	x	FBgn0004403		x

			YCR031C		x	x	x			
PFC0735w	40S ribosomal protein S15A, putative	**0**	YJL190C		x	x	x			
MAL13P1.92	40S ribosomal protein S15/S19, putative	**0**	YOL040C		x	x	x	FBgn0034138		x
PF08_0076	40S ribosomal protein S16, putative	**0**	YMR143W<br>YDL083C		x<br>x	x<br>x	 x	FBgn0034743		x
PFL2055w	40S ribosomal protein S17, putative	**0**	YML024W<br>YDR447C		x<br>x	x<br>x	 x	FBgn0005533		x
PF11_0272	40S ribosomal protein S18, putative	**0**	YML026C<br>YDR450W		x<br>x	x<br>x	 x	FBgn0010411		x
PFD1055w	40S ribosomal protein S19, putative	**0**	YNL302C		x	x	x			
PF10_0038	40S ribosomal protein S20e, putative	**0**	YHL015W			x	x			
PF11_0454	40S ribosomal protein S21e, putative	**1**	YKR057W		x					
PFC0290w	40S ribosomal protein S23, putative	**0**	YPR132W<br>YGR118W		x<br>x	x<br>x	x<br>x	FBgn0033912		x
PFE0975c	40S ribosomal protein S24, putative	**0**	YER074W		x	x	x			
PFB0830w	40S ribosomal protein S26e, putative	**1**	YGL189C<br>YER131W		x<br>x	x<br>x	x<br>x	FBgn0004413		x
PF13_0045	40S ribosomal protein S27, putative	**0**	YHR021C		x	x	x			
PF14_0585	40S ribosomal protein S28e, putative	**N**								
PFB0885w	40S ribosomal protein S30, putative	**N**								
PF14_0027	40S ribosomal protein S31/UBI, putative	**1**	YLR167W		x	x	x			
**Mitochondrial large ribosomal proteins**										
PF07_0046	mitochondrial ribosomal protein L1 precursor, putative	**N**	YDR116C	x				FBgn0037566	x	
PFL0500w	mitochondrial ribosomal protein L1-2 precursor, putative	**0**	YDR116C	x		x		FBgn0037566	x	
PF11_0337	mitochondrial ribosomal protein L2 precursor	**N**								
PFL2180w	mitochondrial ribosomal protein L3 precursor, putative	**0**	YGR220C	x			x	FBgn0030686	x	
PF08_0038	mitochondrial ribosomal protein L4 precursor, putative	**+**	YML025C	x				FBgn0001995	x	
MAL13P1.318	mitochondrial ribosomal protein L9 precursor, putative	**+**	YML022C	x				FBgn0038319	x	
PF11_0113	mitochondrial ribosomal protein L11 precursor, putative	**0**	YNL185C					FBgn0038234	x	x
PFB0545c	mitochondrial ribosomal protein L12 precursor, putative	**N**	YGL068W	x				FBgn0011787	x	
PFB0645c	mitochondrial ribosomal protein L13 precursor, putative	**N**	YOR150W	x				FBgn0032720	x	
PFE0960w	mitochondrial ribosomal protein L14 precursor, putative	**N**	YKL170W	x				FBgn0040389	x	
PF14_0276	mitochondrial ribosomal protein L15 precursor, putative	**0**	YNL284C	x			x	FBgn0036990	x	
PF14_0041	mitochondrial ribosomal protein L16 precursor, putative	**N**	YBL038W	x				FBgn0023519	x	
PFE1125w	mitochondrial ribosomal protein L17 precursor, putative	**N**	YJL063C	x				FBgn0035122	x	
PF14_0289	mitochondrial ribosomal protein L172 precursor, putative	**N**	YJL063C	x				FBgn0035122	x	
PFF0495w	mitochondrial ribosomal protein L19 precursor, putative	**N**	YCR046C	x				FBgn0037608	x	
PF14_0709	mitochondrial ribosomal protein L20 precursor, putative	**N**						FBgn0036335	x	
PF14_0212	mitochondrial ribosomal protein L21 precursor, putative	**+**	YJL096W	x				FBgn0036853	x	
PF10_0097	mitochondrial ribosomal protein L22/L43, putative	**+**	YPR100W<br>YNL177C	x<br>x				FBgn0034893<br>FBgn0030786	x<br>x	
PFL1895w	mitochondrial ribosomal protein L23 precursor, putative	**N**	YDR405W	x				FBgn0035335	x	
PFF0245w	mitochondrial ribosomal protein L24 precursor, putative	**N**	YPL173W	x				FBgn0031651	x	
PFL1150c	mitochondrial ribosomal protein L242 precursor, putative	**N**								
PFC0701w	mitochondrial ribosomal protein L27 precursor, putative	**N**	YNL005C	x				FBgn0053002	x	
PF14_0539	mitochondrial ribosomal protein L28 precursor, putative	**N**	YMR193W	x				FBgn0031660	x	
PFC0675c	mitochondrial ribosomal protein L29/L47 precursor, putative	**2**	YLR439W	x				FBgn0014023<br>FBgn0014023		x
PFF0205w	mitochondrial ribosomal protein L41 precursor, putative	**N**	YBR282W	x						
PFF1305w	mitochondrial ribosomal protein L46 precursor, putative	**N**	YNL252C	x				FBgn0035272	x	
MAL13P1.200	mitochondrial ribosomal protein L49 precursor, putative	**N**	YCR071C	x				FBgn0030433	x	
**Mitochondrial small ribosomal proteins**										
PF14_0584	mitochondrial ribosomal protein S4/S9 precursor, putative	**1,+**	YHR148w			x	x	FBgn0034232		x
MAL7P1.66	mitochondrial ribosomal protein S5 precursor, putative	**+**	YBR251W	x				FBgn0044510	x	
PFI1585c	mitochondrial ribosomal protein S6 precursor, putative	**N**	YKL003C	x				FBgn0035534	x	
PF14_0606	mitochondrial ribosomal protein S62 precursor, putative	**+**	YKL003C	x				FBgn0035534	x	
MAL7P1.93	mitochondrial ribosomal protein S8 precursor, putative	**N**	YMR158W	x						
PF11_0382	mitochondrial ribosomal protein S9 precursor, putative	**N**	YBR146W	x				FBgn0037529	x	
PF14_0519	mitochondrial ribosomal protein S11 precursor, putative	**N**	YNL306W	x				FBgn0038474	x	
PFD0600c	mitochondrial ribosomal protein S12 precursor, putative	**N**	YNR036C	x				FBgn0003714	x	
PF14_0451	mitochondrial ribosomal protein S14 precursor, putative	**0**	YPR166C	x			x	FBgn0044030	x	
PF13_0059	mitochondrial ribosomal protein S15 precursor, putative	**N**	YDR337W	x				FBgn0026261	x	
PFE1560c	mitochondrial ribosomal protein S16 precursor, putative	**N**	YPL013C	x				FBgn0033907	x	
MAL13P1.327	mitochondrial ribosomal protein S17 precursor, putative	**N**	YMR188C	x						
PFL0570c	mitochondrial ribosomal protein S18 precursor, putative	**N**	YER050C	x						
PF10_0267	mitochondrial ribosomal protein S22 precursor, putative	**N**	YKL155C	x				FBgn0032168	x	
PF14_0430	mitochondrial ribosomal protein S29 precursor, putative	**N**	YGL129C	x				FBgn0034727	x	
PF13_0068	mitochondrial ribosomal protein S35 precursor, putative	**N**	YDR175C	x				FBgn0035374	x	
**Organelle ribosomal proteins**										
PF14_0642	organelle ribosomal protein L22/L17 precursor, putative	**N**								
PFE0145w	organelle ribosomal protein L28 precursor, putative	**N**								
**Apicoplast large ribosomal proteins**										
PFCOMPIRBrpl2	apicoplast ribosomal protein L2	**N**								
PFCOMPIRBrpl4	apicoplast ribosomal protein L4	**N**								
PFCOMPIRBrpl6	apicoplast ribosomal protein L6	**N**								
PFD0675w	apicoplast ribosomal protein L10 precursor, putative	**N**								
PFCOMPIRBrpl14	apicoplast ribosomal protein L14	**N**								
PF14_0270	apicoplast ribosomal protein L15 precursor, putative	**N**								
PFCOMPIRBrpl16	apicoplast ribosomal protein L16	**N**								
PFF0650w	apicoplast ribosomal protein L18 precursor, putative	**N**								
PF08_0014	apicoplast ribosomal protein L21 precursor, putative	**N**								
PFCOMPIRBrpl23	apicoplast ribosomal protein L23	**N**								
PF10_0332	apicoplast ribosomal protein L27 precursor, putative	**N**								
PFL0400w	apicoplast ribosomal protein L29 precursor, putative	**N**								
MAL8P1.110	apicoplast ribosomal protein L33 precursor, putative	**N**								
PFCOMPIRBrpl36	apicoplast ribosomal protein L36	**N**								
PFI0375w	apicoplast ribosomal protein L35 precursor, putative	**N**								

PF11_0106	apicoplast ribosomal protein L36e precursor, putative	**N**
**Apicoplast small ribosomal proteins**		
PFCOMPIRArps2	apicoplast ribosomal protein S2	**N**
PFCOMPIRBrps3	apicoplast ribosomal protein S3	**N**
PFCOMPIRBrps4	apicoplast ribosomal protein S4	**N**
PFCOMPIRBrps5	apicoplast ribosomal protein S5	**N**
PFCOMPIRBrps7	apicoplast ribosomal protein S7	**N**
PFCOMPIRBrps8	apicoplast ribosomal protein S8	**N**
PF14_0581	apicoplast ribosomal protein S10 precursor, putative	**N**
PFCOMPIRBrps11	apicoplast ribosomal protein S11	**N**
PFCOMPIRBrps12	apicoplast ribosomal protein S12	**N**
PF11_0386	apicoplast ribosomal protein S14p/S29e precursor, putative	**N**
PF11_0072	apicoplast ribosomal protein S15 precursor, putative	**N**
PFCOMPIRBrps17	apicoplast ribosomal protein S17	**N**
PF14_0205	apicoplast ribosomal protein S25 precursor, putative	**N**
PFCOMPIRBrps19	apicoplast ribosomal protein S19	**N**
