# Supplementary material for: Assessing functional annotation transfers with inter-species conserved coexpression: application to Plasmodium falciparum
Source: BMC Genomics. 2010 Jan 15;11:35. doi: 10.1186/1471-2164-11-35 (PMC2826313; doi:10.1186/1471-2164-11-35)
Supplement: Additional file 7 — Predicted annotations of P. falciparum gene products involved in ribosome biogenesis and assembly (GO:0042254), rRNA metabolic process (GO:0016072) and tRNA processing (GO:0008033). Prediction gained by co-coexpression analyses when compared to PlasmoDB 5.4: (0) confirmed annotation; (1) refined annotation of an incomplete or wrong original functional inference; (2) previously hypothetical; (Z) pair also identified in Zhou et al. (2008) [15]. (x): pairing of P. falciparum and S. cerevisiae or P. falciparum and D. melanogaster genes in co-coexpression analyses. (§): pairing of P. falciparum and S. cerevisiae genes in co-coexpression with different clustering parameters. (*): Correction of the gene models of PF14_0436 and PF14_0437, forming a unique gene coding for a putative nucleolar DEAD/DEAH box ATP-dependent RNA helicase (see Add. File 8). [file 1471-2164-11-35-S7.PDF]

P. falciparum genes	Final annotation	predicted subcellular localization	prediction gained by co-coexpression analyses	*S. cerevisiae* genes	**Comparative analyses *P. falciparum* vs *S. cerevisiae*** LR-GA	BO-GA	BO-SP	*D. melanogaster* genes	**Comparative analyses *P. falciparum* vs *D. melanogaster*** BO-PI
**DNA-directed RNA polymerase I/III subunits (rRNA synthesis)**									
PF11_0358	DNA-directed RNA polymerase I subunit 1, putative	Nucleus	**1, Z**	YPR010C	x	x		FBgn0003276	x
PF11_0445	DNA-directed RNA polymerase I subunit 2, putative	Nucleus	**1, Z**	YPR110C	x	x	x		
PF14_0150	DNA-directed RNA polymerase I/III subunit, putative	Nucleus	**0**	YNL113W	x	x	x	FBgn0032762	x
PF14_0207	DNA-directed RNA polymerase III subunit C, putative	Nucleus	**1**	YNR003C	x	x	x		
**Proteins involved in ribosomal RNA (rRNA) processing**									
PFB0860c	nucleolar DEAD/DEAH box ATP-dependent RNA helicase 1, putative	Nucleolus	**1, Z**	YHR065C	x	x	x	FBgn0032919	x
PFE1085w	nucleolar DEAD/DEAH box ATP-dependent RNA helicase 2, putative	Nucleolus	**1**	YDL031W		x			
PFF1500c	nucleolar DEAD/DEAH box ATP-dependent RNA helicase 3, putative	Nucleolus	**1, Z**	YMR290C	x				
PFL2010c	nucleolar DEAD/DEAH box ATP-dependent RNA helicase 4, putative	Nucleolus	**1, Z**	YLR276C	x	x	x		
PFL2475w	nucleolar DEAD/DEAH box ATP-dependent RNA helicase 5, putative	Nucleolus	**1**	YLL008W	x				
PF13_0177	nucleolar small subunit rRNA associated DEAD/DEAH box ATP-dependent RNA helicase 6, putative	Nucleolus	**1, Z**	YHR169W/ YHR065C	x	x	x		
MAL7P1.113	nucleolar DEAD/DEAH box ATP-dependent RNA helicase 7, putative	Nucleolus	**1, Z**	YKR024C		x	x		
MAL8P1.19	nucleolar DEAD/DEAH box ATP-dependent RNA helicase 8, putative	Nucleolus	**1**	YGL171W/YLL 008W/YDL031 W	x	x			
MAL13P1.14	nucleolar DEAD/DEAH box ATP-dependent RNA helicase 9, putative	Nucleolus	**1**	YMR128W	x	x	x		
PF14_0183	nucleolar small subunit rRNA associated DEAD/DEAH box ATP-dependent RNA helicase 10, putative	Nucleolus	**1**	YJL033W	x		x		
PF14_0436 fused with PF14_0437*	nucleolar DEAD/DEAH box ATP-dependent RNA helicase 11, putative	Nucleolus	**1**	YNL112W	x	x			
PFL0355c	nucleolar small subunit rRNA processing factor, putative	Nucleolus	**2**	YDR365C			§		
PF14_0661	nucleolar small subunit rRNA processing KH domain protein, putative	Nucleolus	**2, Z**	YOR145C	x	x	x		
PF14_0156	nucleolar small subunit rRNA dimethylase, putative	Nucleolus	**1, Z**	YPL266W	x	x	x		
PF13_0310	nucleolar small subunit rRNA processing factor, putative	Nucleolus	**2**	YDL153C	x	x			
PF10_0266	nucleolar small subunit rRNA processing stabilizing factor, putative	Nucleolus	**2**	YJR002W		x	x		
PF10_0089	nucleolar small subunit rRNA synthesis-associated protein, putative	Nucleolus	**2**	YMR229C			§		
PF10_0085	nucleolar small subunit rRNA processing protein, putative	Nucleolus	**1, Z**	YOR310C	x	x			
PF07_0083	nucleolar small subunit rRNA processing protein, putative	Nucleolus	**2**	YNR054C	x	x	x		
MAL8P1.67	nucleolar small subunit rRNA processing PINc domain protein, putative	Nucleolus	**2**	YDR339C	x	x	x		
PFL1230w	small subunit rRNA processing factor, putative	Nucleus Cytoplasm	**2**	YLR186W	x	x	x		
PFD0975w	small subunit rRNA processing serine kinase, putative	Nucleus Cytoplasm	**1**	YNL207W			x		
PF14_0055	nucleolar large subunit rRNA processing protein, putative	Nucleolus	**2, Z**	YMR049C			§		
PF13_0286	nucleolar large subunit rRNA methyl transferase, putative	Nucleolus	**1**	YCL054W	x	x	x		
PF10_0194	nucleolar large subunit rRNA processing RRM protein, putative	Nucleolus	**2**	YOL041C		x	x		
PF13_0315	rRNA associated RNA binding protein, putative	Nucleolus	**1**	YGR159C	x	x			
PFL1820w	rRNA processing WD-repeat protein, putative	Nucleolus, Nucleus, Cytoplasm	**2**	YLR196W	x	x	x		
PF08_0130	rRNA processing WD-repeat protein, putative	Nucleolus Cytoplasm	**1, Z**	YCR057C		x	x		
PF08_0065	ribosomal biogenesis-associated WD repeat protein, putative	Nucleus	**1**	YMR131C	x	x	x		
PF11_0090	general rRNA processing protein, putative	Nucleolus	**2, Z**	YGR103W	x	x			
PFI1235w	rRNA processing and telomere maintaining methyltransferase, putative	Nucleolus	**1**	YDR083W	x	x	x		
PF10_0162	rRNA processing factor, putative	Nucleolus	**2**	YJR041C			§		
PFE1310c	nucleolar Jumonji domain interacting protein, putative	Nucleolus	**2**	YPR169W	x	x	x		
**Proteins associated to small nucleolar RNA (snoRNA) and small nucleolar ribonucleoprotein (snoRNP)**									
PF07_0092	U3 snoRNA-associated small subunit rRNA processing protein 1, putative	Nucleolus	**2**	YER082C		x		FBgn0030000	x
PFL2295w	U3 snoRNA-associated small subunit rRNA processing protein 2, putative	Nucleolus	**2**	YKL099c		x	x	FBgn0030063	x
PFL0830w	snoRNA-associated small subunit rRNA processing protein, putative	Nucleolus	**2**	YPR112C		x	x		
PF14_0456	U3 snoRNA-associated small subunit rRNA processing protein 3, putative	Nucleolus	**2, Z**	YLR129W	x	x	x	FBgn0038597	x
PF13_0309	U3 snoRNA-associated small subunit rRNA processing protein 4, putative	Nucleolus	**2, Z**	YLR409C	x	x	x		
PF13_0184	U3 snoRNA-associated small subunit rRNA processing associated protein, putative	Nucleolus	**2**	YDR324C		x	x		
PF10_0128	U3 snoRNA-associated small subunit rRNA processing WD-repeat protein, putative	Nucleolus	**1**	YLR222C	x				
PF11_0105	U3/U14 snoRNA-associated small subunit rRNA processing protein, putative	Nucleolus	**2**	YBR247C		x	x		
PF11_0250	U3 snoRNP/U4/U6-U5 tri-snRNP-associated mRNA splicing factor, putative	Nucleolus	**1**	YEL026W	x				
PF11_0191	box C/D snoRNP rRNA 2'-O-methylation factor, putative	Nucleolus	**2**	YLR197W		x	x		

PF14_0068	box C/D snoRNP rRNA methyltransferase fibrillarin, putative	Nucleolus	**0, Z**	YDL014W	x	x	x	FBgn0003062	x
MAL8P1.48	small nuclear ribonucleoprotein (snoRNP) mRNA splicing factor, putative	Nucleolus	**0**					FBgn0030765	x
PF14_0174	small nucleolar ribonucleoprotein (snoRNP) pseudouridine synthase, putative	Nucleolus	**1, Z**	YLR175W	x	x	x	FBgn0023184	x
PF13_0051	small nucleolar ribonucleoprotein (snoRNP) pseudouridylase associated protein, putative	Nucleolus	**1**	YHR089C	x	x		FBgn0011824	x
**Proteins associated to small nuclear RNA (snRNA) and transfer RNAs (tRNA)**									
PF10_0341	U2 snRNA/tRNA pseudouridine synthase, putative	Nucleus	**2**	YOR243C		x			
PF08_0123	U2 snRNA/tRNA pseudouridine synthase, putative	Nucleus	**1**	YPL212C	x	x	x		
MAL7P1.28	rRNA/tRNA ribonuclease MRP/P subunit, putative	Nucleolus	**1**	YNL221C			x		
PFE1240w	tRNA-YW synthesizing protein, putative	Endoplasmic reticulum	**2**	YPL207W	x	x			
PF07_0015	tRNA m5C-methyltransferase, putative	Nucleolus	**2, Z**	YBL024W	x	x	x		
PFI0625c	tRNA 1-methyladenosine methyltransferase subunit, putative	Nucleus	**2**	YNL062C	x	x	x		
PF13_0109	tRNA N2,N2-dimethylguanosine methyltransferase, putative	Nucleus Mitochondria	**0**	YDR120C	x	x	x		
PF13_0087	1-methyladenosine tRNA methyltransferase subunit, putative	Nucleus	**2**	YJL125C		x			
PF14_0072	tRNA m2G10 methyltransferase subunit, putative	Nucleus Cytoplasm	**2**	YNR046W	x	x	x		
PF14_0086	tRNA (Phe) U17 dihydrouridine synthase, putative	Nucleus	**2, Z**	YML080W		x			
PF14_0620	tRNA 3'-trailer sequence RNase, putative	Endoplasmic reticulum	**2**	YKR079C	x	x	x		
PF10_0149	cysteinyl tRNA ligase, putative	Cytoplasm	**0**	YNL247W	x	x			
PF11_0116	tRNA m5C-methyltransferase, putative	Nucleolus	**2**	YBL024W	x	x	x		
MAL6P1.214	mRNA binding Pumilio-homology domain protein, putative	Nucleolus, Nucleus	**2**	YDR496C			§		
**Nucleolar and nuclear proteins involved in the assembly of ribosomal subunits**									
PFB0370c	small ribosomal subunit assembling protein, putative	Nucleolus	**1**	YCL059C	x	x	x		
PFA0330w	small ribosomal subunit assembling AARP2 protein, putative	Nucleolus	**1**	YPL217C	x	x	x		
PFD0455w	small ribosomal subunit processing protein, putative	Nucleolus	**1**	YLL011W		x	x		
PFI1070c	large ribosomal subunit assembling BRIX domain protein, putative	Nucleolus	**1**	YHR088W	x	x	x	FBgn0032408	x
PF07_0122	large ribosomal subunit assembling BRIX domain protein, putative	Nucleolus	**1**	YOL077C	x	x	x	FBgn0035524	x
PFB0175c	large ribosomal subunit assembling protein, putative	Nucleus	**1**	YAL025C	x	x	x		
PF14_0635	large ribosomal subunit assembling protein, putative	Nucleolus	**2**	YPL211W		x	x	FBgn0039233	x
PF14_0185	large ribosomal subunit associated ATP-dependent RNA helicase, putative	Nucleus Nucleolus	**1**	YMR290C/ YFL002C/YHR 065C/YGL078 C/YJL033W	x	x	x		
PF11_0471	Large ribosomal subunit processing WD-repeat protein, putative	Nucleolus	**2, Z**	YCR072C	x	x	x		
PF11_0274	large ribosomal subunit processing factor, putative	Nucleus	**2**	YFR001W		x	x		
PF10_0278	large ribosomal subunit assembly L11-binding factor, putative	Nucleolus	**1, Z**	YKR081C	x	x	x	FBgn0038585	x
PF10_0277	large ribosomal subunit assembling factor, putative	Nucleolus	**2**	YKL172W		x	x		
PF10_0197	large ribosomal subunit associated RNA m(5)C methyltransferase protein, putative	Nucleolus	**2**	YNL061W	x	x			
PF07_0067	actin cytoskeleton and large ribosomal subunit assembling factor, putative	Nucleus	**2**	YGR245C	x	x	x		
MAL7P1.24	large ribosomal subunit processing protein, putative	Nucleolus	**2, Z**	YER126C	x	x	x	FBgn0025366	x
PFL0310c	eukaryotic translation initiation factor 3 subunit 8, putative	Nucleus	**0, Z**	YMR309C	x		x		
PF10_0200	ribosomal processing ATPase, putative	Nucleolus	**1, Z**	YNL132W	x	x	x		
**Proteins involved in the export of ribosomal constituents from nucleus**									
MAL13P1.93	small ribosomal subunit nuclear export protein, putative	Nucleus Nucleolus	**2**	YPR144C	x				
PF14_0550	large ribosomal subunit nuclear export factor, putative	Nucleus	**2**	YDR060W	x	x			
PF14_0221	large ribosomal subunit nuclear export RAN GTPase, putative	Nucleus	**1**	YNR053C		x	x	FBgn0034243	x
PF11_0259	large ribosomal subunit nuclear export L11-binding factor, putative	Nucleus	**2**	YOR294W	x			FBgn0033507	x
PF07_0121	large ribosomal subunit nuclear export protein, putative	Nucleus	**1, Z**	YHR170W	x	x	x		
PFE1435c	large ribosomal subunit nuclear export GTP-binding factor, putative	Nucleus Cytoplasm	**2**	YER006W	x	x	x		
PF08_0041	small ribosomal subunit processing microtubule-associated protein, putative	Nucleus Cytoplasm	**1**	YLR186W			x		
MAL13P1.344	small ribosomal subunit processing ABC ATPase (RNAse L inhibitor), putative	Nucleus Cytoplasm	**1**	YDR091C		x	x		
PF13_0178	large ribosomal subunit processing elf6 like factor, putative	Nucleus Cytoplasm	**1**	YPR016C	x	x			
**Cytoplasmic proteins involved in ribosomal biogenesis and assembly**									
PFD0515w	exosome rRNA processing protein, putative	Nucleus Cytoplasm	**0, Z**	YHR069C		x	x		
PFL2150c	cytoplasmic translation machinery associated protein, putative	Cytoplasm	**2**	YOR091W		x	x		
PFE1215c	cytoplasmic translation machinery associated GTPase, putative	Cytoplasm	**1**	YAL036C	x				
PFI0815c	ribosomal biogenesis associated methyltransferase protein, putative	Cytoplasm	**2**	YIL064W			x		
PF14_0494	small subunit rRNA processing factor, putative	Cytoplasm	**2, Z**	YDL060W	x	x	x		
PF14_0292	large ribosomal subunit associated GTPase, putative	Cytoplasm	**2**	YGL099W	x	x	x		
PF14_0360	elF2A like protein, putative	Cytoplasm	**2**	YGR054W			§		
PF11_0225	GCN20-like elf2 alpha kinase ABC activator, putative	Cytoplasm	**1**	YFR009W			§		
PF14_0136	eukaryotic translation initiation factor 2 (elf2) histidine:diphtamide synthetase subunit 1, putative	Cytoplasm	**2**	YIL103W			x		
PF14_0274	eukaryotic translation initiation factor 2 (elf2) histidine:diphtamide synthetase subunit 2, putative	Cytoplasm	**1**	YKL191W			x		
