# Supplementary material for: Assessing functional annotation transfers with inter-species conserved coexpression: application to Plasmodium falciparum
Source: BMC Genomics. 2010 Jan 15;11:35. doi: 10.1186/1471-2164-11-35 (PMC2826313; doi:10.1186/1471-2164-11-35)
Supplement: Additional file 8 — Correction of the gene structures and functional inference of PF14_0436 and PF14_0437 based on P. falciparum vs. S. cerevisiae co-coexpression analyses. In co-coxpression analyses, both PF14_0436 and PF14_0437 were in the same expression cluster and with the same yeast sequence, YNL112W, involved in ribosome biogenesis and assembly (GO:0042254, see Add. File 7). Sequence comparison of these contiguous predicted open reading frames shows that they align with both extremities of YNL112W, and that their overlapping region is an error of the gene model, corrected in updated versions of PlasmoDB. [file 1471-2164-11-35-S8.PDF]

```
            1                                                         60
PF14_0437   ------------------------------------------------------------
PF14_0436       MRGFNYNNNSNNNNRYSAYPDYNNSYGNYQAYGQQYRANYGTGYVPNYNNNSNNYG
YNL112W     MTYGGRDQQYNKTNYKSRGGDFRGGRNSDRNSYNDRPQGGNYRGGFGGRSNYNQPQELIK

            61                                                       120
PF14_0437   ------------------------------------------------------------
PF14_0436   KNLAPIDWKTINLVPFEKNFYKEHEDISKLSTKEVKEIRDKHKITILEGENVPKPVVSIN
YNL112W     PNW---DEELPKLPTFEKNFYVEHESVRDRSDSEIAQFRKENEMTI-SGHDIPKPITTFD

            121                                                      180
PF14_0437   ------------------------------------------------------------
PF14_0436   KIGFPDYVIKSLKNNNIVAPTPIQIQGWPIALSGKDMIGKAETGSGKTLAFILPAFVHIL
YNL112W     EAGFPDYVLNEVKAEGFDKPTGIQCQGWPMALSGRDMVGIAATGSGKTLSYCLPGIVHIN

            181                                                      240
PF14_0437   ------------------------------------------------------------
PF14_0436   AQPNLKYGDGPIVLVLAPTRELAEQIRQECIKFSTESKIRNTCAYGGVPKSGQIYALKQG
YNL112W     AQPLLAPGDGPIVLVLAPTRELAVQIQTECSKFGHSSRIRNTCVYGGVPKSQQIRDLSRG

            241                                                      300
PF14_0437   ------------------------------------------------------------
PF14_0436   VHILIACPGRLIDLLEQNVTNLMRVTYLVLDEADRMLDMGFELQIRKIVDQIRPDRQTLM
YNL112W     SEIVIATPGRLIDMLEIGKTNLKRVTYLVLDEADRMLDMGFEPQIRKIVDQIRPDRQTLM

            301                                                      360
PF14_0437   --------------------------------------MKKANINEHEKIGNLKSLLQRI
PF14_0436   WSATWPKEVQALAKDLCKEQPIQVNVGSLTLTACRSIKQEIYLLED-SYSSSLQIYK---
YNL112W     WSATWPKEVKQLAADYLND-PIQVQVGSLELSASHNITQIVEVVSDFEKRDRLNKYLETA

            361                                                      420
PF14_0437   FKDND-RIIVEVETKKNADFITKALRLDGMPALCIHGDKKQEERRWVLNEFKTGKSPIMI
PF14_0436   ------------------------------------------------------------
YNL112W     SQDNEYKTLIFASTKRMCDDITKYLREDGWPALAIHGDKDQRERDWVLQEFRNGRSPIMV

            421                                                      480
PF14_0437   ATDVASRGLDIKNVKYVINFDFPNQIEDYVHRIGRTGRAGSHGASFTFLTADKYRLAKDL
PF14_0436   ------------------------------------------------------------
YNL112W     ATDVAARGIDVKGINYVINYDMPGNIEDYVHRIGRTGRAGATGTAISFFTEQNKGLGAKL

            481                                                      540
PF14_0437   VKILRESEQPVPPQLEKISY-SMGNNQRRNPYYSSGRSNNVNNIPLRGNNRFY
PF14_0436   ------------------------------------------------------------
YNL112W     ISIMREANQNIPPELLKYDRRSYGGGHPRYGGGRGGRGGYGRRGGYGGGRGGYGGNRQRD

            541       551
PF14_0437
PF14_0436
YNL112W     GGWGNRGRSN Y
```
