# Supplementary material for: Assessing functional annotation transfers with inter-species conserved coexpression: application to Plasmodium falciparum
Source: BMC Genomics. 2010 Jan 15;11:35. doi: 10.1186/1471-2164-11-35 (PMC2826313; doi:10.1186/1471-2164-11-35)
Supplement: Additional file 9 — Other annotations of P. falciparum gene products based on co-coexpression analyses. Prediction gained by co-coexpression analyses when compared to PlasmoDB 5.4: (0) confirmed annotation; (1) refined annotation of an incomplete or wrong original functional inference; (2) previously hypothetical; (Z) pair also identified in Zhou et al. (2008) [15]. (x): pairing of P. falciparum and S. cerevisiae or P. falciparum and D. melanogaster genes in co-coexpressed analyses. (§): pairing of P. falciparum and S. cerevisiae genes in co-coexpression with different clustering parameters. This table summarizes important functional annotation predicted for P. falciparum genes previously reported as "hypotheticals" or with little indication of a putative function. [file 1471-2164-11-35-S9.PDF]

				**Comparative analyses *P. falciparum* vs *S. cerevisiae***				**Comparative analyses *P. falciparum* vs *D. melanogaster***
P. falciparum genes	Final annotation	prediction gained by co-coexpression analyses	*S. cerevisiae* genes	LR-GA	BO-GA	BO-SP	*D. melanogaster* genes	BO-PI
**Histone modification**								
PFL1345c	histone S-adenosyl methyltransferase, putative	**1, Z**	YPL086C	x	x	x		
PF11_0192	histone acetyltransferase catalytic subunit, putative	**2**	YBL052C<br>YOR244W			§		
**Cytoskeleton dynamics**								
PFC0960c	Microtubule and actin binding protein	**2**					FBgn0020503	x
PFL2105c	microtubule associated katanin, putative	**2**					FBgn0032030	x
PFE0595w	subunit of the heterohexameric Gim/prefoldin protein complex, putative	**2**	YLR200W	x				
PF11_0292	microtubule associated heterohexameric cochaperone prefoldin complex subunit, putative	**2**	YML094W	x				
PF14_0324	Hsp70/Hsp90 organizing protein, putative	**2**					FBgn0024352	x
**Inter-organellar protein transfer machineries**								
PFE0140c	mitochondrial inner membrane TIM10 associated protein, putative	**2**	YHR005C-A	x				
PF08_0087	nuclear protein import karyopherin alpha protein, putative	**1**	YNL189W	x			FBgn0024889	x
**Golgi organisation and biogenesis**								
PFD0880w	Golgi organization and biogenesis factor 1, putative	**2**					FBgn0033902	§
PFC0100c	Golgi organization and biogenesis factor 2, putative	**2**					FBgn0030365	x
**Mitochondrial proteins**								
PFE0670w	mitochondrial matrix protein import constituent, putative	**2**	YLR008C			§		
PFD0145c	mitochondrial FMP25-like protein, putative	**2**	YLR077W			§		
**Proteasome associated proteins**								
PF08_0109	26S proteasome19S regulatory particle non-ATPase base subunit, putative	**2**	YHR200W			x	FBgn0015283	x
PF14_0178	polyubiquitinated protein - 26S proteasome guiding protein, putative	**2**	YGR048W	x	x	x		
**Other functions (unclassified)**								
PF14_0478	DNA polymerase delta interacting protein, putative	**2**	YPR048W			x		
PF14_0587	spliceosome associated protein, putative	**2**					FBgn0031493	x
PF14_0028	polyribosome independent poly-A-RNA-binding protein, putative	**2**	YNL016W	x	x			
PFL0175c	Aerobic respiration associated protein, putative	**2**	YGL169W		x			
PFB0280w	3-dehydroquinate synthase, putative	**2**	YDR127W			§		
PF10_0219	cytoplasmic KELch repeat protein, putative	**2**	YPL263C			x		
PFI1680w	UBX domain protein, putative	**2**	YDL091C			x		
PFE0515w	TSR4-like protein, putative	**2**	YOL022C	x		x		
MAL13P1.21	Chromosome condensation protein, putative	**2**	YBL097W	x				
PF11_0488	Serine/theonine kinase similar to RAD53, putative	**1**	YPL153C			x		
